# Supplementary material for: Nickel-catalyzed switchable arylative/endo-cyclization of 1,6-enynes
Source: Nat Commun. 2024 Apr 4;15:2914. doi: 10.1038/s41467-024-47200-z (PMC10995176; doi:10.1038/s41467-024-47200-z)
Supplement: Supplementary file 1 — Supplementary Information [file 41467_2024_47200_MOESM1_ESM.pdf]

Supplementary Information

Nickel-Catalyzed Switchable Arylative/*endo*-Cyclization of 1,6-Enynes

Wenfeng Liu,[1,‡] Wei Li,[1,‡] Weipeng Xu,[2] Minyan Wang,[2,*] and Wangqing Kong[1,*]

[1]The Institute for Advanced Studies, Wuhan University, Wuhan 430072, China; ✉email: wqkong@whu.edu.cn

[2]State Key Laboratory of Coordination Chemistry, School of Chemistry and Chemical Engineering, Nanjing University, Nanjing 210023, China; ✉email: wangmy@nju.edu.cn

‡These authors contributed equally: Wenfeng Liu, Wei Li.

Contents

1. Supplementary Methods

1.1 General Information 3

1.2 General Information 4

2. Supplementary Discussion

2.1 Synthesis of Starting Materials 7

2.2 Optimization Details 11

2.3 Mechanistic studies 12

2.4 Computational Investigations 20

2.5 Characterization Data of Products 138

2.6 NMR Spectra 221

3. Supplementary References 399

1. Supplementary Methods

1.1 General Information

Nuclear magnetic resonance (NMR) spectroscopy measurements were carried out at room temperature. 1H NMR, ^{13}C NMR, ^{19}F NMR, HSQC, HMBC, COSY and NOESY experiments were carried out using Bruker ADVANCE III (600 MHz) or JNM-ECZ400S/L1 (400 MHz) spectrometers. Chemical shifts (δ) are reported in ppm relative to the residual solvent peak with corresponding coupling constants (J) in Hertz (Hz) and multiplicities (s: singlet, d: doublet, t: triplet, q: quartet, m: multiplet and combinations of these and app.: apparent multiplicities). Gas chromatography were determined with a SHIMADZU Nexis GC 2030 gas chromatography instrument with an FID detector. High-resolution mass spectra (HRMS) were recorded on Thermo Fisher Orbitrap Elite mass spectrometer. Enantiomeric excesses were determined with a SHIMADZU LC-20ADXR system using chiral stationary phase columns (DAICEL) by comparing the samples with the corresponding racemic samples. Column and elution details were specified in each entry.

Materials and Methods: Commercially available reagents and ligands were purchased from Sigma Aldrich, Alfa Aesar, and Strem Chemicals and unless otherwise stated were used without further purification. $NiBr_2{\cdot}DME$, NiI_2 and $Ni(OAc)_2$ were bought from Strem Chemicals. All reactions dealing with air- or moisture-sensitive compounds were performed in the argon-filled glove box or by standard Schlenk techniques in oven-dried reaction vessels under argon atmosphere. Solvents were purchased in HPLC quality, degassed by purging thoroughly with argon and dried over 4 Å activated molecular sieves. More sensitive compounds were stored in a desiccator or in a glove-box if required. Reactions were monitored by thin layer chromatography (TLC) using glass 0.25 mm silica gel plates. Compounds were visualized by UV-light at 254 nm and by dipping the plates in an aqueous potassium permanganate solution followed by heating. Flash column chromatography was performed over silica gel (200-400 mesh).

1.2 General Information

1.2.1 General procedure for the synthesis of 7-membered ring products

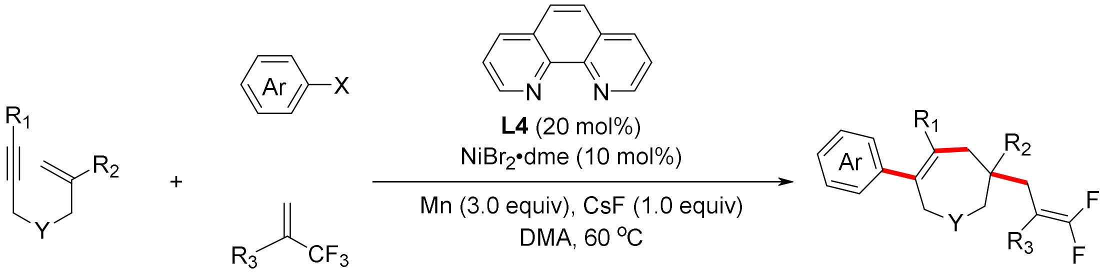

An oven-dried sealed tube equipped with a PTFE-coated stir bar was charged with $NiBr_2$(DME) (10 mol%), **L4** (20 mol%), trifluoromethyl alkene (0.1 mmol), 1,6-enyne (0.2 mmol), CsF (1.0 equiv), Mn powder (3.0 equiv), aryl bromide (2.0 equiv) and anhydrous DMA (2 mL). The sealed tube was sealed and removed from the glovebox. Then the reaction was stirred at 60 °C until the reaction was complete (monitored by TLC). The resulting mixture was quenched with saturated NH_4Cl solution (5 mL) and further diluted with water (10 mL). The aqueous layer was extracted with EtOAc and the combined organic layers were washed with brine, dried over anhydrous Na_2SO_4, filtered, and concentrated under vacuum. The residue was purified by chromatography on silica gel, eluting with PE/EtOAc (50/1~5/1) to afford the desired 7-membered ring products.

1.2.2 General procedure for the synthesis of 6-membered ring products

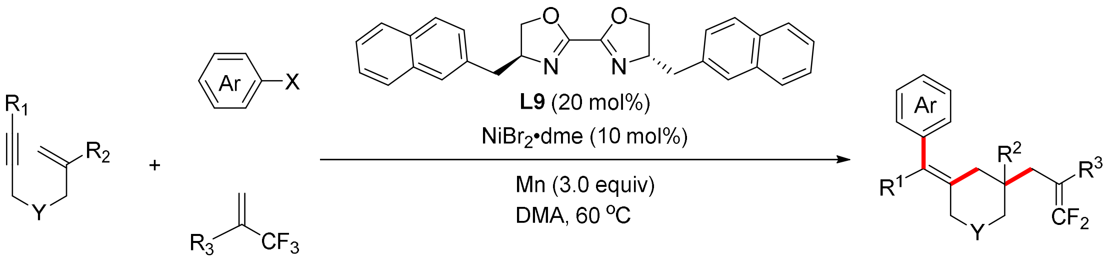

An oven-dried sealed tube equipped with a PTFE-coated stir bar was charged with $NiBr_2$(DME) (10 mol%), **L9** (20 mol%), trifluoromethyl alkene (0.1 mmol), 1,6-enyne (0.2 mmol), Mn powder (3.0 equiv), aryl bromide (2.0 equiv) and anhydrous DMA (2 mL). The sealed tube was sealed and removed from the glovebox. Then the reaction was stirred at 60 °C until the reaction was complete (monitored by TLC). The resulting mixture was

quenched with saturated NH_4Cl solution (5 mL) and further diluted with water (10 mL). The aqueous layer was extracted with EtOAc and the combined organic layers were washed with brine, dried over anhydrous Na_2SO_4, filtered, and concentrated under vacuum. The residue was purified by chromatography on silica gel, eluting with PE/EtOAc (50/1~5/1) to afford the desired 6-membered ring products.

1.2.3 General procedure for the diastereoselective synthesis of 6-membered ring products

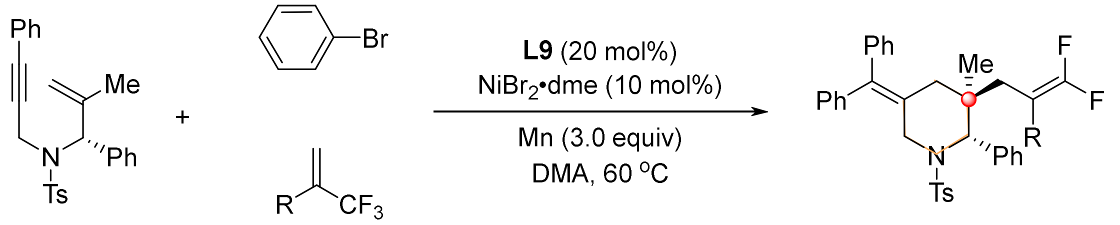

An oven-dried sealed tube equipped with a PTFE-coated stir bar was charged with $NiBr_2$(DME) (10 mol%), **L9** (20 mol%), trifluoromethyl alkene (0.1 mmol), 1,6-enyne (0.2 mmol), Mn powder (3.0 equiv), aryl bromide (2.0 equiv) and anhydrous DMA (2 mL). The sealed tube was sealed and removed from the glovebox. Then the reaction was stirred at 60 °C until the reaction was complete (monitored by TLC). The resulting mixture was quenched with saturated NH_4Cl solution (5 mL) and further diluted with water (10 mL). The aqueous layer was extracted with EtOAc and the combined organic layers were washed with brine, dried over anhydrous Na_2SO_4, filtered, and concentrated under vacuum. The residue was purified by chromatography on silica gel, eluting with PE/EtOAc (50/1~5/1) to afford the chiral 6-membered ring products.

1.2.4 General procedure for the diastereoselective synthesis of 7-membered ring products

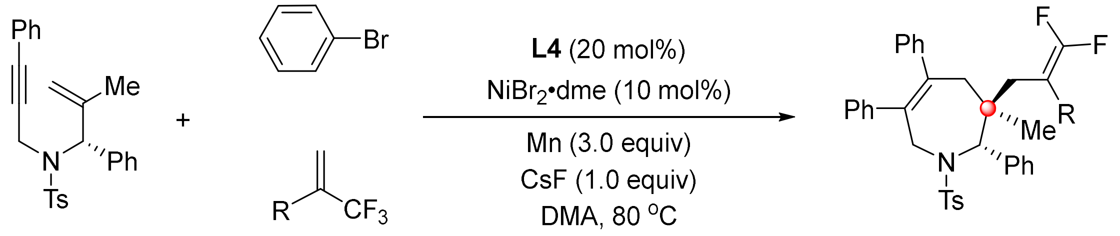

An oven-dried sealed tube equipped with a PTFE-coated stir bar was charged with

$NiBr_2$(DME) (10 mol%), **L4** (20 mol%), trifluoromethyl alkene (0.1 mmol), chiral 1,6-enyne (0.2 mmol), CsF (1.0 equiv), Mn power (3.0 equiv), aryl bromide (2.0 equiv) and anhydrous DMA (2 mL). The sealed tube was sealed and removed from the glovebox. Then the reaction was stirred at 80 °C until the reaction was complete (monitored by TLC). The resulting mixture was quenched with saturated NH_4Cl solution (5 mL) and further diluted with water (10 mL). The aqueous layer was extracted with EtOAc and the combined organic layers were washed with brine, dried over anhydrous Na_2SO_4, filtered, and concentrated under vacuum. The residue was purified by chromatography on silica gel, eluting with PE/EtOAc (50/1~5/1) to afford the chiral 7-membered ring products.

1.2.5 General procedure for the diarylation of 1,6-enynes

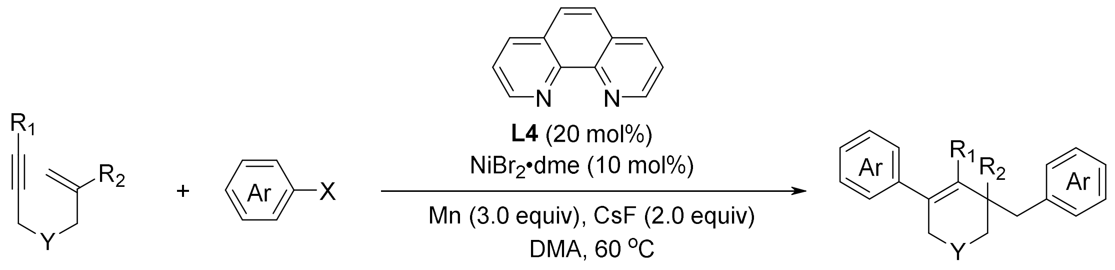

An oven-dried sealed tube equipped with a PTFE-coated stir bar was charged with $NiBr_2$(DME) (10 mol%), **L4** (20 mol%), 1,6-enyne (0.2 mmol), CsF (2.0 equiv), Mn powder (3.0 equiv), aryl bromide (6.0 equiv) and anhydrous DMA (2 mL). The sealed tube was sealed and removed from the glovebox. Then the reaction was stirred at 60 °C until the reaction was complete (monitored by TLC). The resulting mixture was quenched with saturated NH_4Cl solution (5 mL) and further diluted with water (10 mL). The aqueous layer was extracted with EtOAc and the combined organic layers were washed with brine, dried over anhydrous Na_2SO_4, filtered, and concentrated under vacuum. The residue was purified by chromatography on silica gel, eluting with PE/EtOAc (50/1~5/1) to afford the desired diarylated products.

2. Supplementary Discussion

2.1 Synthesis of Starting Materials

2.1.1 Procedure for the synthesis of enantioenriched 1,6-enyne 74

Synthesized according to a modified procedure of M. Lautens and co-workers.[1]

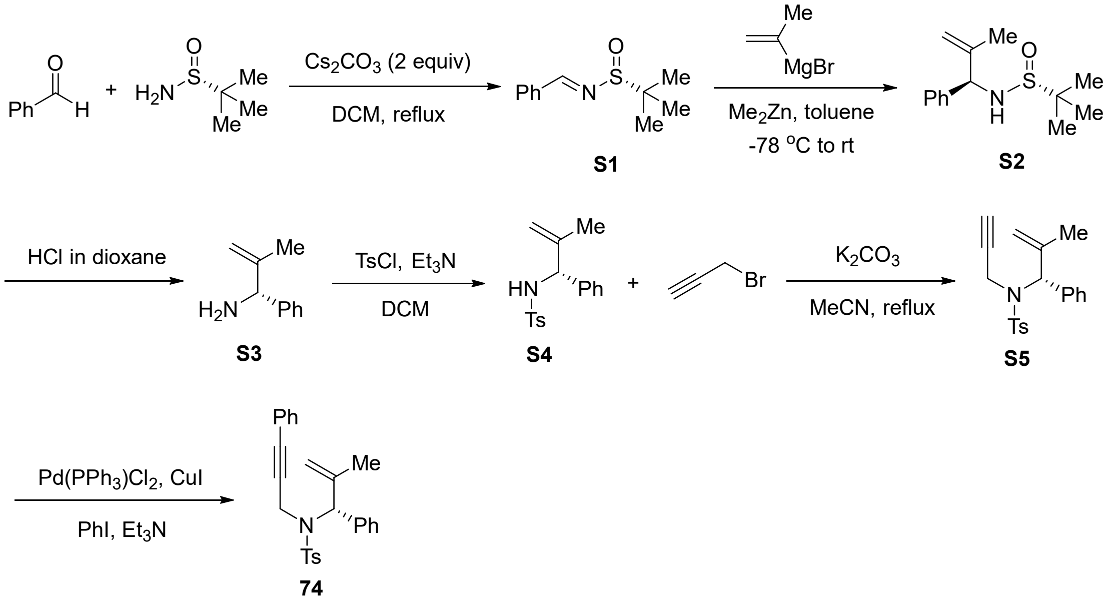

To a mixture of (*R*)-2-methylpropane-2-sulfinamide (1.2 equiv) and anhydrous Cs_2CO_3 (2 equiv) in DCM was added benzaldehyde (1 equiv). The reaction was heated at reflux until complete (monitored by TLC). The mixture was then cooled to room temperature and filtered through with DCM. The collected organic fraction was concentrated under vacuum, and the obtained sulfinamide **S1** was directly used in the next step without purification.

To a solution of isopropenyl magnesium bromide (0.5 M in THF, 1.8 equiv) was added dropwise a solution of dimethylzinc (2 M in toluene, 1.8 equiv) at room temperature. The resulting solution was stirred at this temperature for 30 minutes. The second flask was charged with the sulfinamide (**S1**, 1 equiv) and dry THF under argon at -78 °C. The generated zinc reagent contained in the first flask was added slowly drop-wise via cannula to the second flask under argon. This resulting solution was stirred at -78 °C for 3 hours before slowly warming to room temperature until complete (monitored by TLC). The reaction was then quenched by the addition of a saturated aqueous solution of NH_4Cl at 0 °C. The resulting biphasic mixture was filtered and

extracted with EtOAc. The combined organic layers were washed with brine, dried over $MgSO_4$, filtered, and concentrated in vacuo. The residue was purified by flash column chromatography to give the desired product **S2**.

To a mixture of the sulfinamide **S2** (1 equiv) in Et_2O was added dropwise a solution of anhydrous HCl in 1,4-dioxane (4 M, 2 equiv) under argon at 0 °C. The resulting mixture was stirred at 0 °C for 30 minutes. The white precipitate was filtered and washed with cooled Et_2O. The filter cake was dissolved in distilled water and diluted with Et_2O. The mixture was slowly basified to pH 10 using 1 M aqueous NH_4OH. The layers were separated, and the aqueous layer was extracted with Et_2O. The combined organic layers were washed with brine, dried over Na_2SO_4, filtered and concentrated in vacuo. The chiral amine **S3** was obtained without further purification.

To a solution of **S3** (1.47 g, 10 mmol, 1.0 equiv.) in DCM (20 mL) was added TsCl (2.7g, 10 mmol, 1.0 equiv.) and Et_3N (3.61 g, 2.5 equiv). The mixture was stirred at room temperature for 12 hours until complete (monitored by TLC). The mixture was concentrated under vacuum and purified by flash column chromatography to give the desired product **S4** (2.8 g, 93% yield).

To a solution of **S4** (2.8g, 9.3 mmol, 1.0 equiv.) in MeCN (10 mL) was added 3-bromoprop-1-yne (1.6 g, 1.5 equiv.) and K_2CO_3 (2.6 g, 2.0 equiv). The mixture was refluxed until the reaction was complete (monitored by TLC). The mixture was concentrated under vacuum and purified by flash column chromatography to give the desired product **S5** (2.7 g, 87% yield).

An over-dried Schlenk (50 mL) equipped with a PTFE-coated stir bar was charged with $Pd(PPh_3)Cl_2$ (42.0 mg, 0.02 equiv), CuI (28.5 mg, 0.05 equiv.), **S5** (1.0 g, 1.0 equiv), iodobenzene (0.92 g, 1.5 equiv), and Et_3N (10 mL). The mixture was stirred at 55 °C until the reaction was complete (monitored by TLC). The mixture was concentrated under vacuum and purified by flash column chromatography to give the desired product **74** (0.98 g, 79% yield).

^{1}H NMR (600 MHz, $CDCl_3$) δ 7.8-7.8 (m, 2H), 7.3-7.2 (m, 6H), 7.2-7.1 (m, 4H), 7.1-7.0 (m, 2H), 5.6 (s, 1H), 5.2 (q, *J* = 1.4 Hz, 1H), 5.0 (dt, *J* = 1.9, 1.0 Hz, 1H), 4.4- 4.2 (m, 2H), 2.3 (s, 3H), 1.7 (d, *J* = 1.4 Hz, 3H);

^{13}C NMR (151 MHz, $CDCl_3$) δ 143.2, 142.4, 137.9, 136.5, 131.4, 129.2, 129.2, 128.4, 128.1, 128.1, 127.9, 127.8, 122.7, 115.9, 84.8, 83.9, 66.6, 35.3, 21.6, 21.5.

3.2 Procedure for the synthesis of alkenyl iodide 100

Synthesized according to a modified procedure of H. Liu and co-workers.[2]

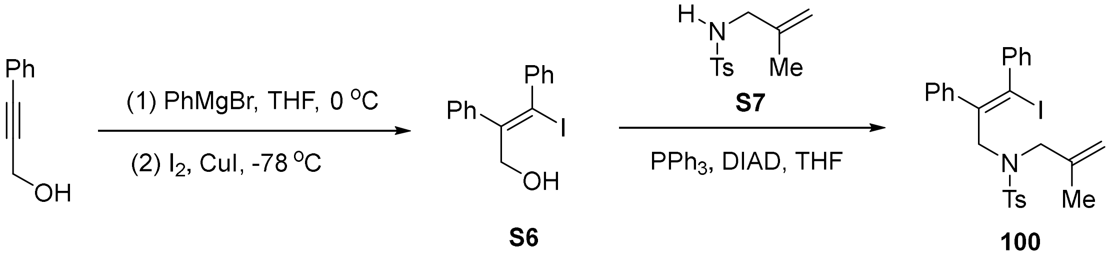

To a stirred solution of 3-phenylprop-2-yn-1-ol (50 mmol, 1.0 equiv) in anhydrous THF (70 mL) was added CuI (10 mol%, 5 mmol, 0.952 g) under Ar. The mixture was then cooled to -78 °C. Grignard reagent (2.5 equiv, 125 mmol), which were freshly prepared in anhydrous THF, was added dropwise to maintain the temperature below -60 °C. Upon complete addition of the Grignard reagent, the dark green suspension allowed to slowly warm to room temperature. The dark green mixture was again cooled to -78 °C and was added dropwise the solution of I_2 (13.0 g, 1.1 equiv.) in anhydrous THF (60 mL) within 2 hours. After warming up to room temperature and stirring at room temperature for 1 h, the reaction was cooled to 0 °C and quenched with saturated aqueous NH_4Cl. The aqueous layer was extracted by ether and the combined organic layers were washed with saturated aqueous $Na_2S_2O_3$, and dried over Na_2SO_4. The mixture was concentrated under vacuum and purified by flash column chromatography to give **S6**.

To the solution of **S6** (3 mmol, 1.0 equiv.) and **S7** (3.3 mmol, 1.1 equiv.) in THF (10 mL) was added PPh_3 (3.3 mmol, 1.1 equiv) under N_2 at room temperature. The solution of DIAD (3.3 mmol, 1.1 equiv) in THF (5 mL) was added dropwise over 10 min. The reaction mixture was stirred at room temperature for overnight. The mixture was concentrated under vacuum and purified by flash column chromatography to give **100** (0.879 g, 54% yield).

^1H NMR (600 MHz, $CDCl_3$) δ 7.65-7.61 (m, 2H), 7.26-7.22 (m, 2H), 7.08-6.94 (m, 8H),

6.86-6.81 (m, 2H), 4.90 (s, 1H), 4.88 (s, 1H), 4.60 (s, 2H), 3.50 (s, 2H), 2.42 (s, 3H), 1.44 (s, 3H); ^{13}C NMR (151 MHz, $CDCl_3$) δ 144.3, 143.5, 143.1, 140.4, 136.8, 136.6, 129.6, 129.4, 129.1, 127.62, 127.57, 127.3, 127.0, 114.6, 102.2, 59.5, 54.6, 21.5, 20.0.

3.3 Procedure for the synthesis of deuterated 1,6-enyne (*Z*)-107-D

Synthesized according to a modified procedure of X. Tong and co-workers.[3]

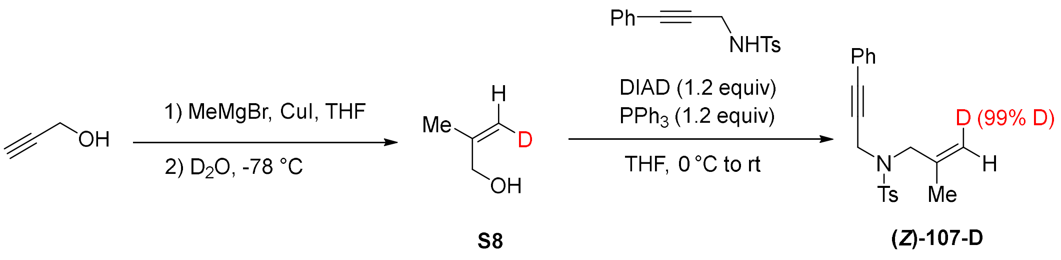

To a stirred solution of propargyl alcohol (2.5 g, 45 mmol, 1.0 equiv) in anhydrous THF (60 mL) was added CuI (0.85 g, 0.1 equiv) under Ar. The mixture was cooled to -78 °C, 50 mL of MeMgBr (3.0 M in THF) was added dropwise, and then was stirred at room temperature for 18 hours. The reaction was again cooled to -78 °C, and treated with D_2O (5 mL) and then allowed to warm to room temperature. After acidified with 1 M HCl (50 mL), the combined organic layers were washed with brine and dried over anhydrous Na_2SO_4. The solvent was evaporated to afford the deuterated allyl alcohol **S8** (2.9 g, 87% yield), which was used directly for the next step without further purification.

To a mixture of deuterated allyl alcohol **S8** (0.44 g, 6.0 mmol) and propargylamide (1.4 g, 5.0 mmol) in THF (20 mL) was added PPh_3 (1.6 g, 6.0 mmol) and DIAD (1.2 g, 6.0 mmol) under Ar at 0 ºC. The mixture was stirred at room temperature for 12 hours. The solvent was evaporated and the residue was purified by column chromatography (petroleum ether/ethyl acetate = 20:1) to give the desired (*Z*)-**107-D** (1.1 g, 65% yield). ^{1}H NMR (600 MHz, $CDCl_3$) δ 7.84-7.76 (m, 2H), 7.33-7.21 (m, 5H), 7.10-7.02 (m, 2H), 5.01 (s, 1H), 4.27 (s, 2H), 3.82 (s, 2H), 2.34 (s, 3H), 1.83 (d, J = 1.5 Hz, 3H);
^{13}C NMR (151 MHz, $CDCl_3$) δ 143.4, 139.2, 136.0, 131.4, 129.5, 128.3, 128.1, 127.8, 122.2, 115.3 (t, J = 23.8 Hz), 85.6, 81.6, 52.6, 36.4, 21.4, 19.7;
HRMS: (ESI) calcd for $C_{20}H_{21}DNO_2S[M+H]^+$ 341.1429; found 341.1427.

2.2 Optimization Details

Supplementary Table 1. Optimization of the enantioselective cyclization/cross-couplings

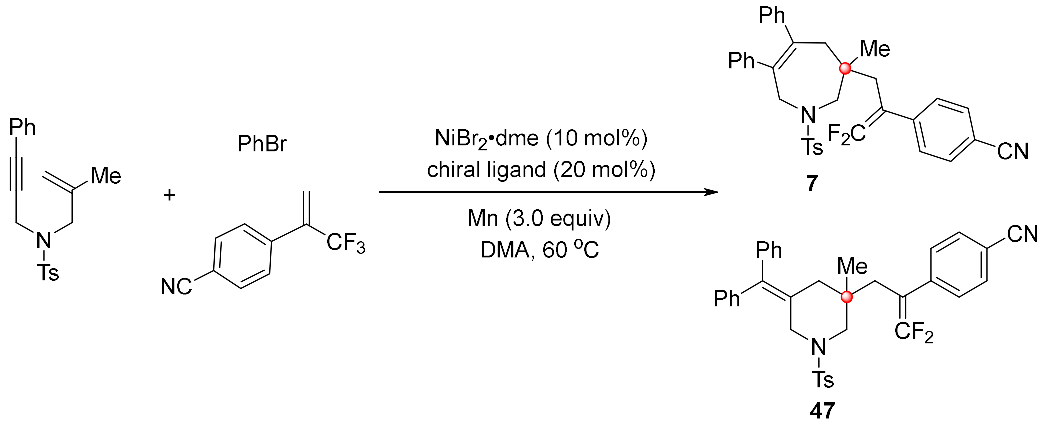

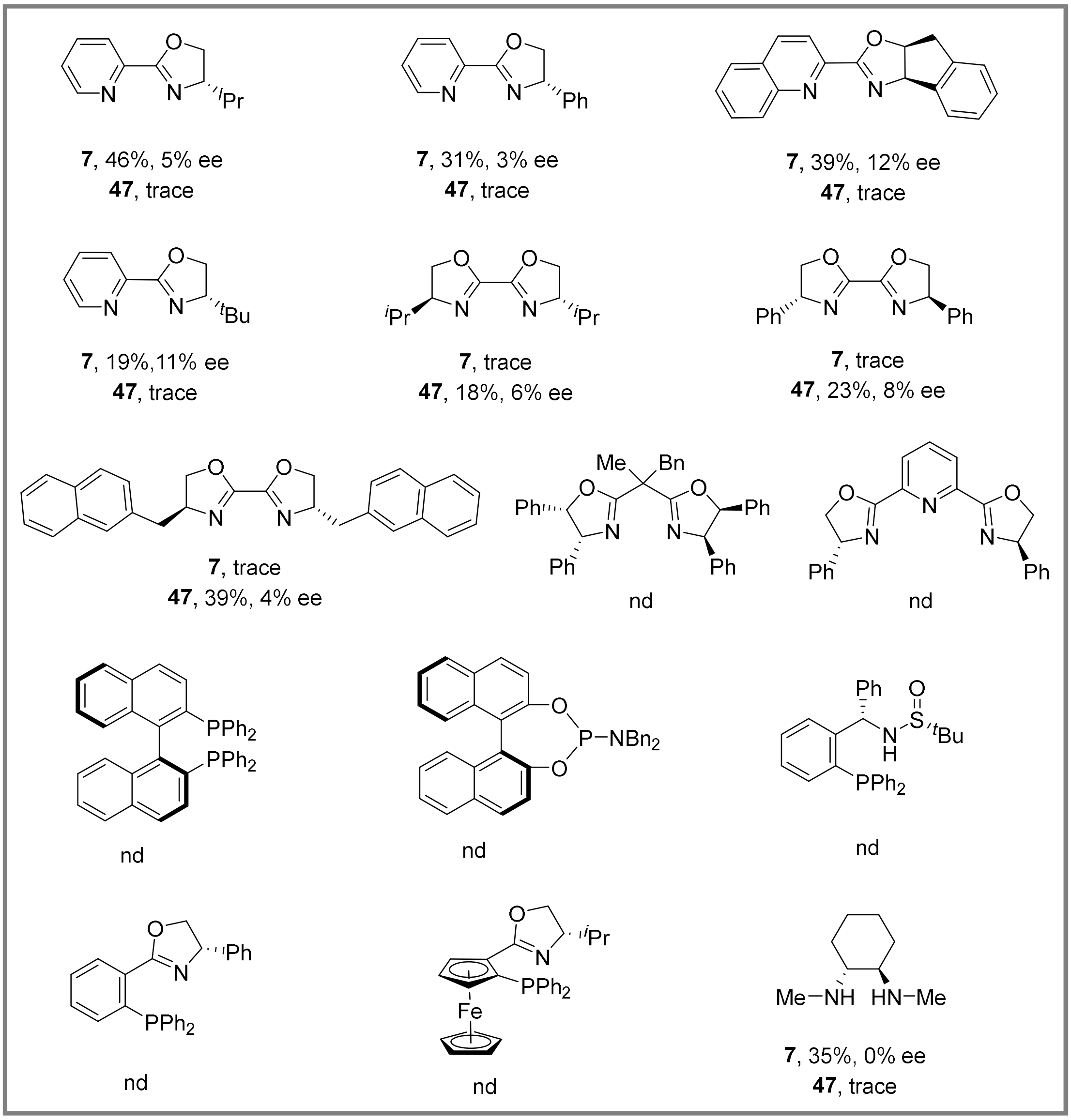

2.3 Mechanistic studies

2.3.1 Stoichiometric reaction with aryl-nickel complex

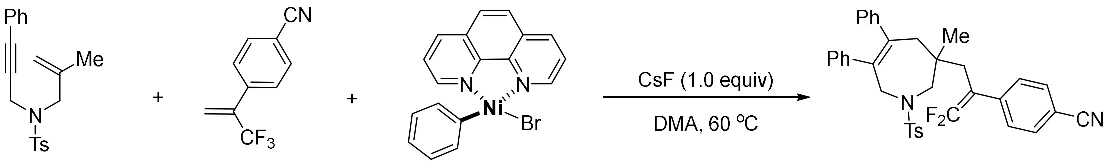

97 3 98 **7**, 31%

The aryl-nickel complex was synthesized according to a modified procedure of T. Diao and co-workers.[4]

An oven-dried sealed tube equipped with a PTFE-coated stir bar was charged with enyne **97** (1.5 equiv, 50.9 mg), **3** (1.5 equiv, 29.6 mg), CsF (1.0 equiv, 15.0 mg), **98** (0.1 mmol, 39.5 mg) and anhydrous DMA (2 mL). The sealed tube was sealed and removed from the glovebox. Then the reaction was stirred at 60 °C for 36 hours. The resulting mixture was quenched with saturated NH_4Cl solution (5 mL) and further diluted with water (10 mL). The aqueous layer was extracted with EtOAc and the combined organic layers were washed with brine, dried over anhydrous Na_2SO_4, filtered, and concentrated under vacuum. The residue was purified by chromatography on silica gel, eluting with PE/EtOAc (10/1) to afford the corresponding product **7** (18.5 mg, 31% yield).

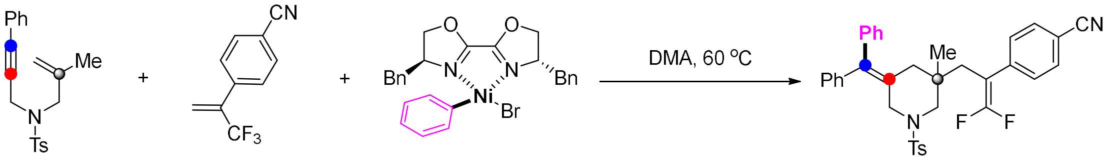

97 3 99 **47**, 30%

An oven-dried sealed tube equipped with a PTFE-coated stir bar was charged with enyne **97** (1.5 equiv, 50.9 mg), trifluoromethyl alkene **3** (1.5 equiv, 29.6 mg), CsF (1.0 equiv, 15.0 mg), **99** (0.1 mmol, 53.5 mg) and anhydrous DMA (2 mL). The sealed tube was sealed and removed from the glovebox. Then the reaction was stirred at 60 °C for 36 hours. The resulting mixture was quenched with saturated NH_4Cl solution (5 mL) and further diluted with water (10 mL). The aqueous layer was extracted with EtOAc and the combined organic layers were washed with brine, dried over anhydrous Na_2SO_4, filtered, and concentrated under vacuum. The residue was purified by chromatography on silica gel, eluting with PE/EtOAc (10/1) to afford the corresponding product **47** (18.0

mg, 30% yield).

2.3.2 Reaction of alkenyl iodide with trifluoromethylalkene

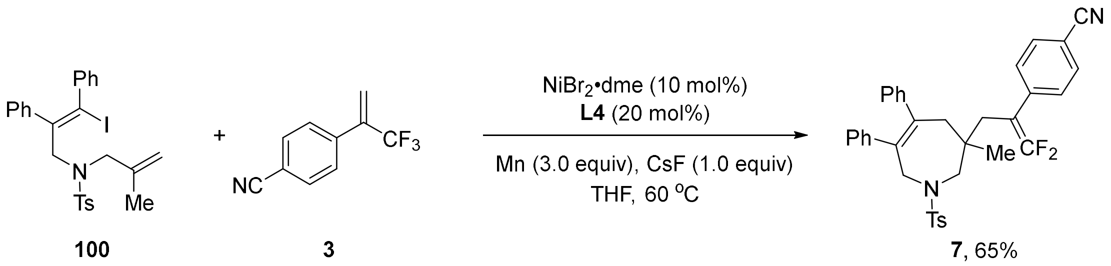

An oven-dried sealed tube equipped with a PTFE-coated stir bar was charged with $NiBr_2$(DME) (10 mol%, 3.2 mg), **L4** (20 mol%, 3.6 mg), **100** (0.1 mmol, 54.4 mg), **3** (2.0 equiv, 39.4 mg), CsF (1.0 equiv, 15.0 mg), Mn powder (3.0 equiv, 16.5 mg) and anhydrous THF (2 mL). The sealed tube was sealed and removed from the glovebox. Then the reaction was stirred at 60 °C for 48 h. The solvent was removed under vacuum, and the resulting residue was purified by chromatography on silica gel, eluting with PE/EtOAc (10/1) to afford the corresponding product **7** (38.6 mg, 65% yield).

2.3.3 Deuterium labelling reaction

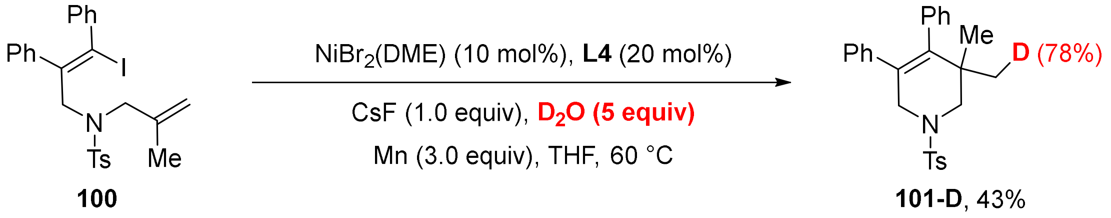

An oven-dried sealed tube equipped with a PTFE-coated stir bar was charged with $NiBr_2$(DME) (10 mol%, 3.2 mg), **L4** (20 mol%, 3.6 mg), **100** (0.1 mmol, 54.4 mg), CsF (1.0 equiv, 15.0 mg), Mn powder (3.0 equiv, 16.5 mg), D_2O (5.0 equiv, 10.0 mg) and anhydrous THF (2 mL). The sealed tube was sealed and removed from the glovebox. Then the reaction was stirred at 60 °C for 48 h. The resulting mixture was quenched with saturated NH_4Cl solution (5 mL) and further diluted with water (10 mL). The aqueous layer was extracted with EtOAc and the combined organic layers were washed with brine, dried over anhydrous Na_2SO_4, filtered, and concentrated under vacuum. The residue was purified by chromatography on silica gel, eluting with PE/EtOAc (10/1) to

afford the corresponding product **101-D** (18.0 mg, 43% yield, 78% D-incorporation).

^{1}H NMR (600 MHz, $CDCl_3$) δ 7.70 (d, *J* = 8.2 Hz, 1H), 7.35 (d, *J* = 8.1 Hz, 1H), 7.11-6.99 (m, 4H), 6.95-6.89 (m, 1H), 6.89-6.84 (m, 1H), 3.81 (s, 1H), 3.07 (s, 1H), 2.45 (s, 2H), 1.13 (s, 5.22H);

^{13}C NMR (151 MHz, $CDCl_3$) δ 143.6, 142.8, 139.3, 138.2, 133.0, 131.1, 130.5, 129.7, 129.0, 127.8, 127.7, 127.1, 126.6, 126.2, 56.2, 50.1, 36.6, 25.9, 21.5.

101-D

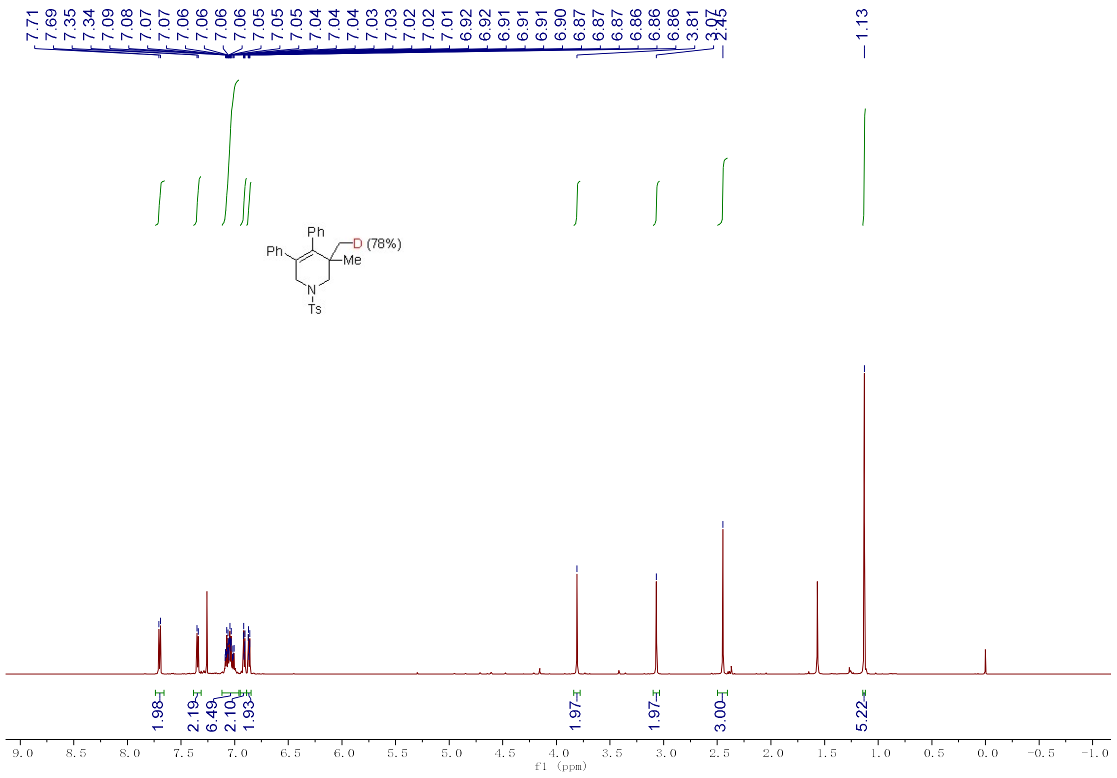

Supplementary Figure 1. ^{1}H NMR of compound **101-D**

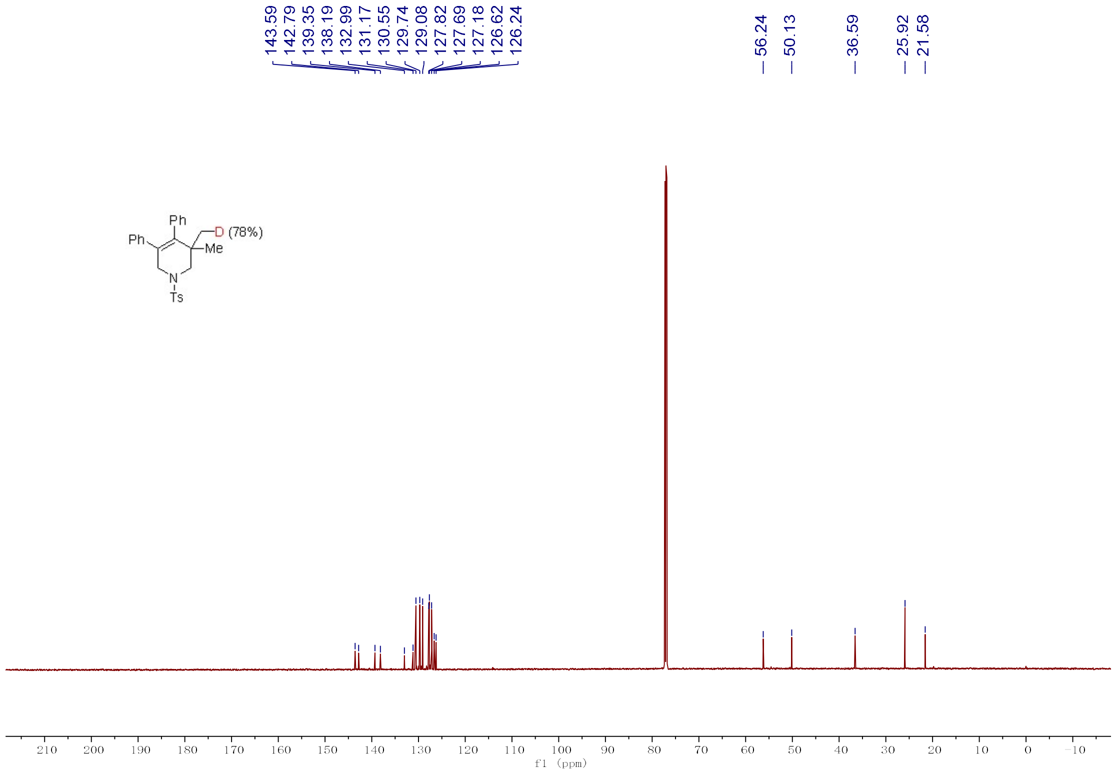

Supplementary Figure 2. ^{13}C NMR of compound **101-D**

2.3.4 Reaction of alkenyl iodide with trifluoromethylalkene in the presence of D_2O

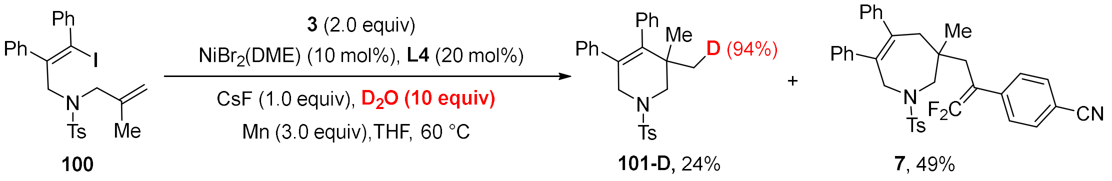

An oven-dried sealed tube equipped with a PTFE-coated stir bar was charged with $NiBr_2$(DME) (10 mol%, 3.2 mg), **L4** (20 mol%, 3.6 mg), **100** (0.1 mmol, 54.4 mg), **3** (2.0 equiv, 39.4 mg), CsF (1.0 equiv, 15.0 mg), Mn powder (3.0 equiv, 16.5 mg), D_2O (10.0 equiv, 20.0 mg) and anhydrous THF (2 mL). The sealed tube was sealed and removed from the glovebox. Then the reaction was stirred at 60 °C for 48 h. The resulting mixture was quenched with saturated NH_4Cl solution (5 mL) and further diluted with water (10 mL). The aqueous layer was extracted with EtOAc and the combined organic layers were washed with brine, dried over anhydrous Na_2SO_4, filtered, and concentrated under vacuum. The residue was purified by chromatography on silica gel, eluting with PE/EtOAc (10/1) to afford the corresponding product **101-D** (10.0 mg, 24% yield, 94% D-incorporation) and **7** (29.1 mg, 49% yield).

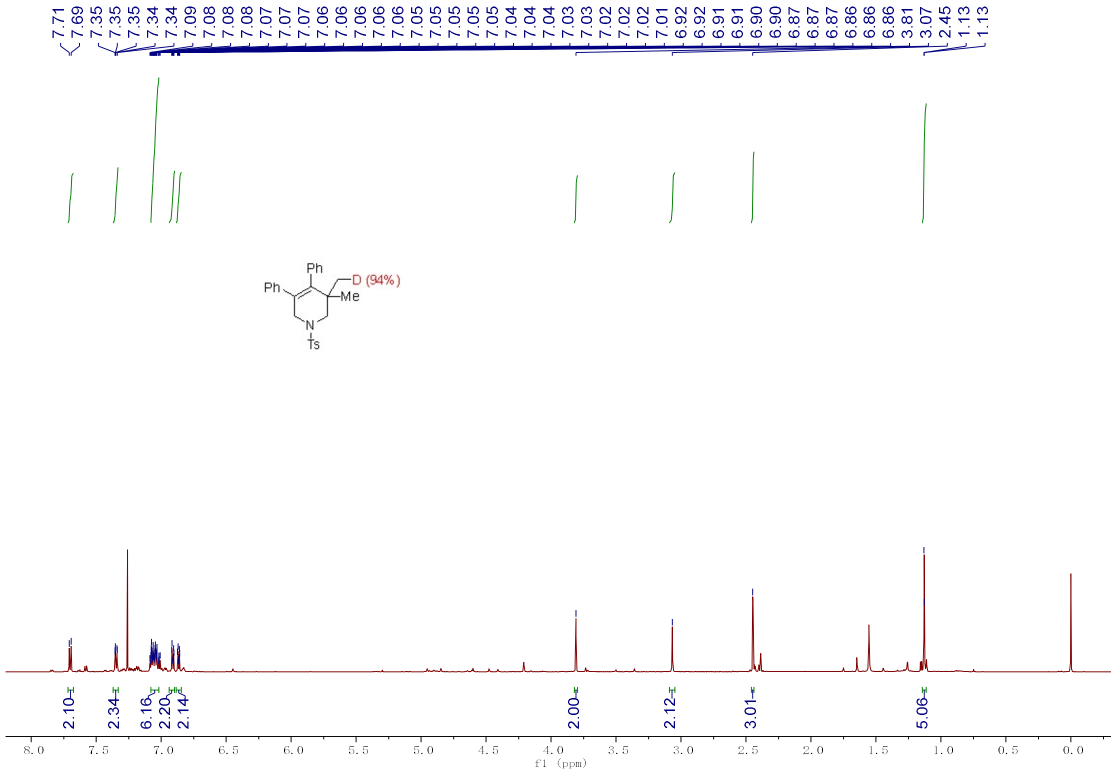

Supplementary Figure 3. ^{1}H NMR of compound **101-D**

2.3.5 Deuterium labelling reaction

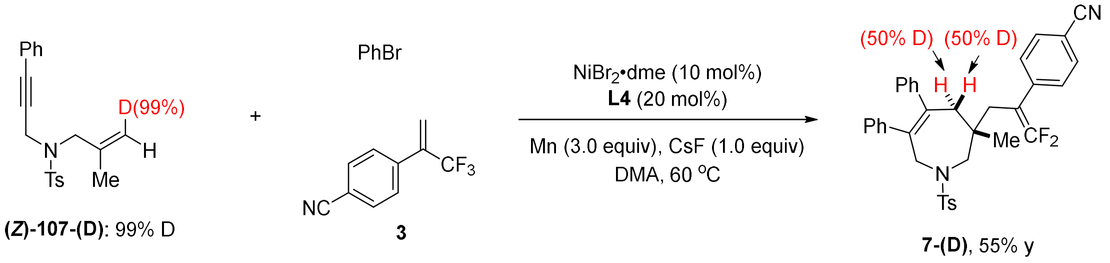

An oven-dried sealed tube equipped with a PTFE-coated stir bar was charged with $NiBr_2$(DME) (10 mol%, 3.2 mg), **L4** (20 mol%, 3.6 mg), **(*Z*)-107-D** (2.0 equiv, 68.0 mg), **3** (0.1 mmol, 19.7 mg), bromobenzene (2.0 equiv, 31.4 mg), CsF (1.0 equiv, 15.0 mg), Mn powder (3.0 equiv, 16.5 mg) and anhydrous DMA (2 mL). The sealed tube was sealed and removed from the glovebox. Then the reaction was stirred at 60 °C for 48 h. The resulting mixture was quenched with saturated NH_4Cl solution (5 mL) and further diluted with water (10 mL). The aqueous layer was extracted with EtOAc and the combined organic layers were washed with brine, dried over anhydrous Na_2SO_4, filtered, and concentrated under vacuum. The residue was purified by chromatography on silica gel, eluting with PE/EtOAc (10/1) to afford the corresponding product **7-D** (32.8 mg, 55% yield).

^{1}H NMR (600 MHz, $CDCl_3$) δ 7.68-7.62 (m, 2H), 7.49-7.44 (m, 2H), 7.43-7.39 (m, 2H), 7.29-7.26 (m, 2H), 7.10-7.03 (m, 6H), 7.03-6.99 (m, 2H), 6.87-6.82 (m, 2H), 4.25 (t, J = 15.2 Hz, 1H), 3.81 (t, J = 15.5 Hz, 1H), 3.25 (dd, J = 18.1, 12.5 Hz, 1H), 2.75-2.63 (m, 1.5H), 2.56-2.49 (m, 1H), 2.48-2.42 (m, 4.5H), 0.94 (d, J = 6.6 Hz, 3H);

^{19}F NMR (565 MHz, $CDCl_3$) δ -84.69 (dd, J = 30.8, 15.7 Hz), -87.54 (d, J = 30.3 Hz);

^{13}C NMR (151 MHz, $CDCl_3$) δ 155.7 (dd, J = 292.3, 290.0 Hz), 144.2, 143.5, 141.6, 140.1 (dd, J = 4.6, 2.9 Hz), 139.28, 139.26, 135.48, 135.46, 134.78, 134.76, 132.3, 129.7, 129.5, 129.2 (t, J = 2.5 Hz), 128.9, 128.8, 127.9, 127.8, 127.13, 127.11, 126.5, 126.2, 118.5, 111.1, 89.0 (dd, J = 22.4, 13.6 Hz), 61.1, 61.0, 54.7, 46.4 (t, J = 18.3 Hz), 38.70, 38.68, 38.2, 38.0, 22.6, 22.4, 21.5.

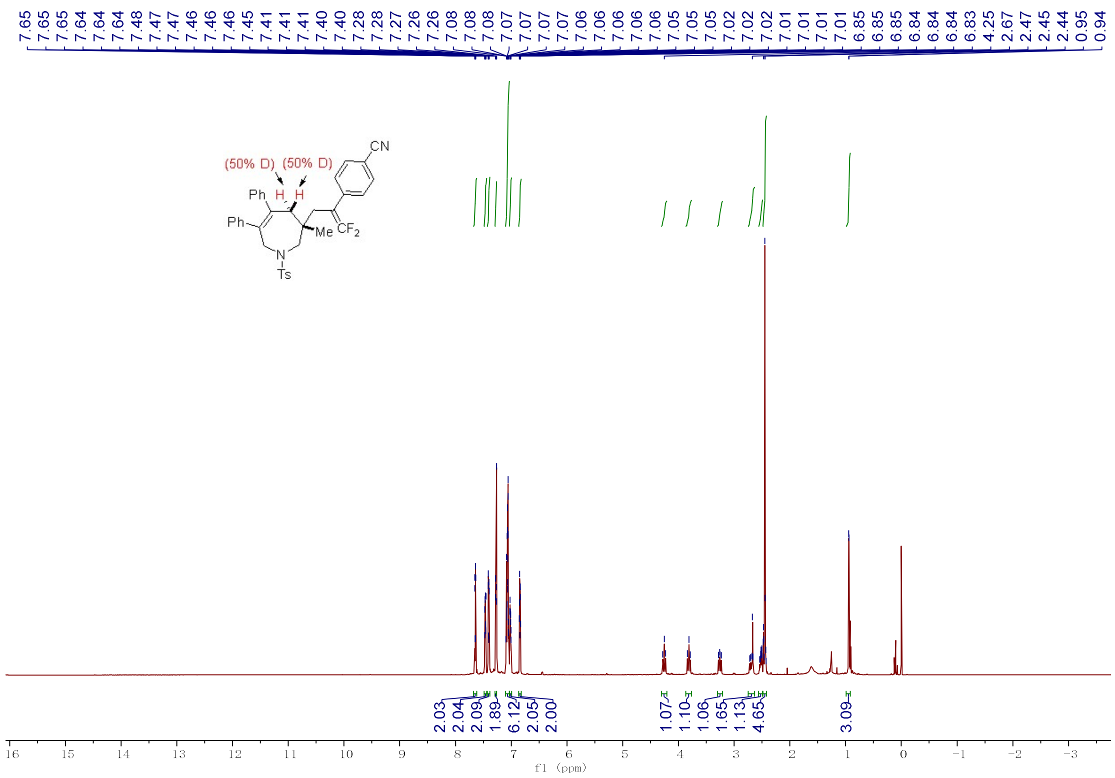

Supplementary Figure 4. ^{1}H NMR of compound **7-D**

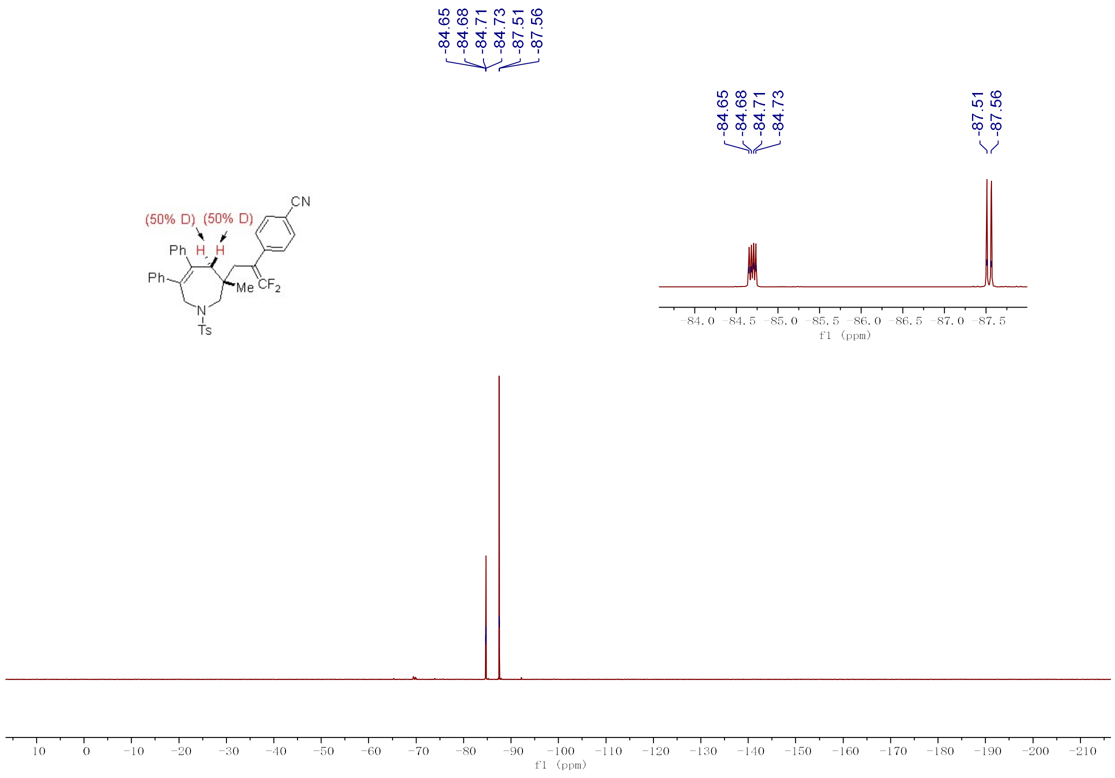

Supplementary Figure 5. ^{19}F NMR of compound **7-D**

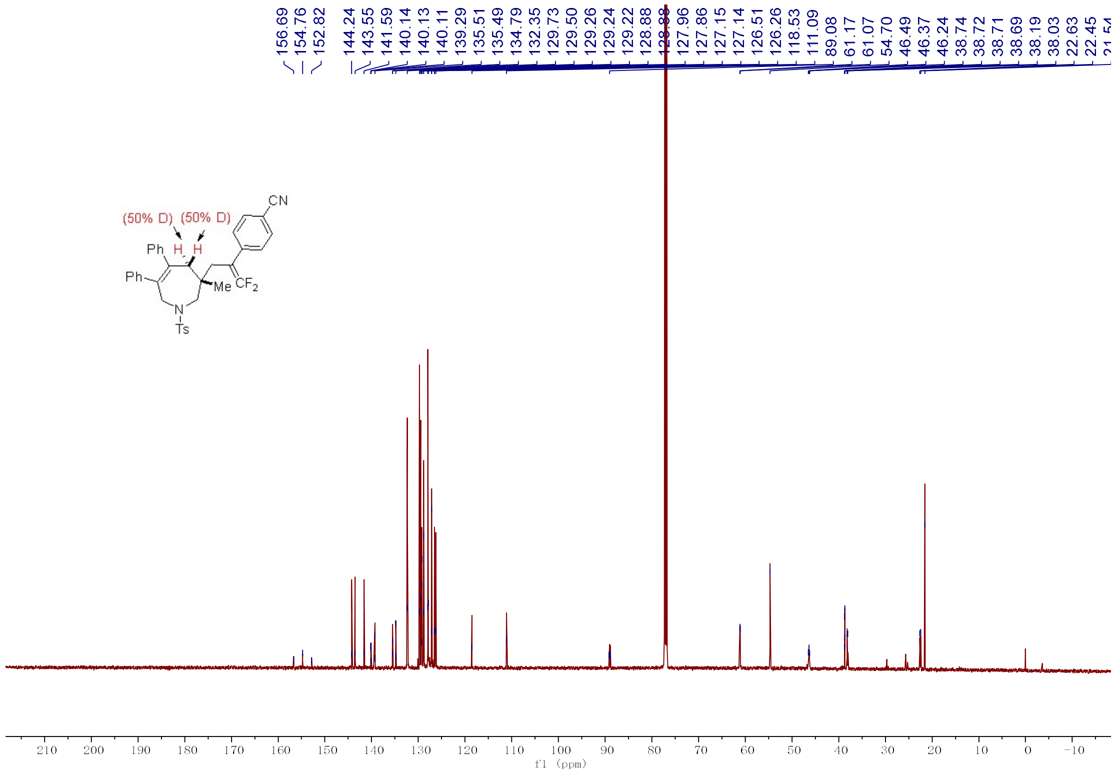

Supplementary Figure 6. ^{13}C NMR of compound **7-D**

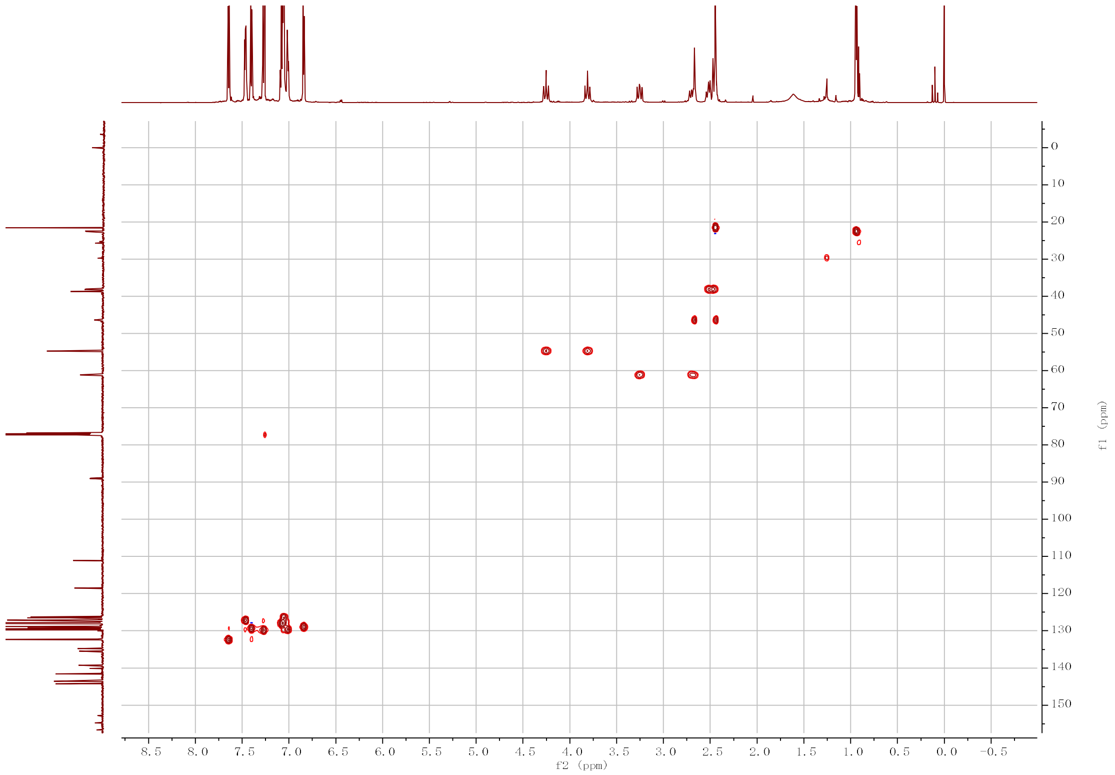

Supplementary Figure 7. HSQC of compound **7-D**

2.4 Computational Investigations

All of the computations were performed with the Gaussian 09 package.[6] Geometry optimizations for all the intermediates and transition structures were calculated with B3LYP[7-8] level of theory with Grimme D3 correction[9] and a mix basis set of LANL08[10-14] for the Ni atom and the 6-31G(d)[15-17] for other atoms. Vibrational frequencies were carried out at the same level to obtain the thermal correction to free energies at 298.15 K and 1 atm pressure and to determine that the optimized structures are either local minimums or transition states. The single-point energies were calculated at the M06-D3[18] functional with the SDD[19-20] basis set for the Ni atom and the 6-311+G(d,p)[21-22] basis set was used for other atoms with SMD[23] solvation model in DMAc. The final Gibbs free energies were determined by the formula $G_{solv} = G_{corr} + E_{solv}$. The frontier molecular orbital analysis was also conducted at B3LYP-D3/6-31G(d)-LANL08 level of theory. Noncovalent Interactions (NCI)[24] was performed with Multiwfn[25] software package. The visualization of NCI Plots is generated using VMD[26] visualization software. The calculated 3D optimized structures are displayed utilizing CYLview visualization program.[27] The key transition states related with regioselectivity have been calculated with SMD implicit solvation model at B3LYP-D3/6-31G(d)-LANL08 level of theory. The corresponding results are shown in the following table. The calculated results indicated the geometry optimizations conducted in gas phase have little influence on the free energy barriers of transition states.

Supplementary Table 2. Extensive computational studies on the migratory insertion process

structure	ΔG≠(optimized in gas) (kcal mol^{-1})	ΔG≠(optimized in solvent) (kcal mol^{-1})
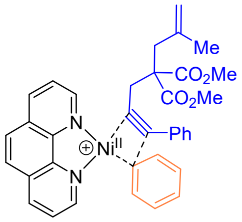 **TS4A-L4**	17.8	17.9
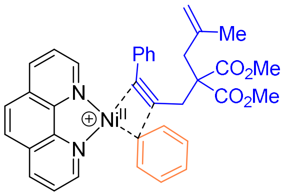 **TS4B-L4**	12.9	13.0
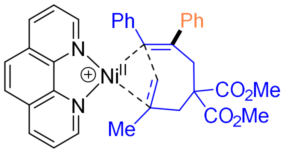 **TS5B-L4-endo**	14.3	14.5
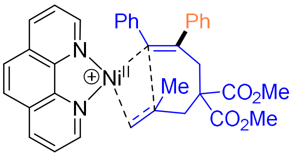 **TS5B-L4-exo**	11.3	11.1

Furthermore, four DFT functionals (M06-D3, B3LYP-D3, ωB97X-D, and PBE0-D3) with SDD for Ni and 6-311+G(d,p) for other atoms was further used to compute the solvation single-point energies in *N,N*-Dimethyl Acetamide with SMD continuum model. All of the energies are calculated as the sum of the solution-phase free energy and the corresponding thermal correction obtained in gas phase. The absolute (in Hartree) single-point energies and relative (in kcal mol^{-1}) Gibbs free energies were shown as follows:

Supplementary Table 3. The absolute (in Hartree) single-point energies and relative (in kcal mol-1) Gibbs free energies.

structure	G_{corr}	$E_{M06\text{-}D3}$	$\Delta G_{M06\text{-}D3}$	$E_{B3LYP\text{-}D3}$	$\Delta G_{B3LYP\text{-}D3}$	$E_{\omega B97X\text{-}D}$	$\Delta G_{\omega B97X\text{-}D}$	$E_{PBE0\text{-}D3}$	$\Delta G_{PBE0\text{-}D3}$
**INT1A-L4**	0.215569	-3547.895181	0.0	-3548.655671	0.0	-3548.335915	0.0	-3547.283435	0.0
**TS2A-L4**	0.215351	-3547.877212	11.1	-3548.648224	4.5	-3548.322682	8.2	-3547.271652	7.3
**INT2A-L4**	0.218248	-3547.946452	-30.5	-3548.721857	-39.9	-3548.400643	-38.9	-3547.344723	-36.8
**INT3A-L4**	0.538278	-1972.174875	-18.4	-1973.533250	-29.2	-1972.811360	-29.1	-1971.298169	-24.5
**TS4A-L4**	0.539319	-1972.147467	-0.6	-1973.503975	-10.2	-1972.784108	-11.3	-1971.274222	-8.8
**INT4A-L4**	0.543161	-1972.216332	-41.4	-1973.574105	-51.8	-1972.857774	-55.1	-1971.347492	-52.3
**TS4B-L4**	0.539307	-1972.155198	-5.5	-1973.509496	-13.7	-1972.788924	-14.3	-1971.280230	-12.6
**INT4B-L4**	0.542542	-1972.193538	-27.5	-1973.553358	-39.2	-1972.835334	-41.4	-1971.326780	-39.7
**INT5B-L4**	0.545795	-1972.214507	-38.6	-1973.575319	-50.9	-1972.856847	-52.9	-1971.348509	-51.3
**TS5B-L4-endo**	0.544448	-1972.190366	-24.3	-1973.541773	-30.7	-1972.828440	-35.9	-1971.321778	-35.4
**INT5B-L4-endo**	0.549674	-1972.221754	-40.7	-1973.581053	-52.1	-1972.869774	-58.6	-1971.362449	-57.6
**TS5B-L4-exo**	0.545092	-1972.195846	-27.3	-1973.551520	-36.4	-1972.835519	-39.9	-1971.329249	-39.7
**INT5B-L4-exo**	0.550387	-1972.233436	-47.6	-1973.590805	-57.8	-1972.882150	-65.9	-1971.371944	-63.2
**TS6B-L4**	0.540696	-1972.203663	-35.0	-1973.555018	-41.4	-1972.848757	-51.0	-1971.338584	-48.3
**INT6B-L4**	0.133390	-3316.441303	-50.6	-3317.018435	-53.7	-3316.797601	-65.6	-3315.932356	-64.6
**INT7B**	0.378910	-1229.957643	0.0	-1230.867773	0.0	-1230.409036	0.0	-1229.409533	0.0
**TS8B**	0.380007	-1229.948685	6.3	-1230.858175	6.7	-1230.398619	7.2	-1229.401715	5.6
**INT8B**	0.382898	-1229.975957	-9.0	-1230.877916	-3.9	-1230.423217	-6.4	-1229.426559	-8.2
**TS8C**	0.501519	-1968.692755	8.1	-1970.045892	10.7	-1969.324287	11.7	-1967.771370	10.6
**INT8C**	0.507023	-1968.750819	-33.9	-1970.102190	-25.0	-1969.389139	-25.5	-1967.835541	-26.2
**TS9B**	0.382100	-1229.952172	5.4	-1230.860833	6.4	-1230.400143	7.6	-1229.404749	5.0
**INT9B**	0.380707	-1229.962467	-1.9	-1230.874114	-2.9	-1230.414296	-2.2	-1229.415450	-2.6
**TS10B**	0.503843	-1968.698592	5.9	-1970.054007	7.1	-1969.330470	9.3	-1967.779360	7.0
**INT10B**	0.510597	-1968.741351	-16.7	-1970.091664	-12.3	-1969.377305	-15.9	-1967.824983	-17.4
**TS11B**	0.508771	-1968.692426	12.9	-1970.050643	12.3	-1969.327740	14.1	-1967.775272	12.7
**INT11B**	0.509611	-1968.885819	-54.6	-1970.234569	-67.6	-1969.517970	-74.9	-1967.966546	-73.2

Note: Input keywords for optimization: #opt freq b3lyp/genecp empiricaldispersion= gd3; Input keywords for solvation single-point energy: #M06/genecp empiricaldispersion = gd3 scrf = (SMD, solvent = DMA) int = ultrafine

The calculated results indicated the solvation single-point energies of key transition states using different functionals are well in agreement with the experimental observed regioselectivity and the reaction trends. Given the advantages of the M06 functional, a hybrid meta functional with good accuracy "across-the-board" for transition metals, main group thermochemistry, medium-range correlation energy, and barrier heights, the single-point calculations were performed with at M06-D3/6-311+G(d,p)-SDD/SMD level of theory. For the Ni-catalyzed system, the DFT calculations at this level are supported by many literatures, such as: (1) Hie, L. Fine Nathel, N. F. Shah, T. K. Baker, E. L. Hong, X. Yang, Y. Liu, P. Houk, K. N. & Garg, N. K. Conversion of amides to esters by the nickel-catalysed activation of amide C-N bonds. *Nature*, **524**, 79 (2015). (2) Hie, L. Fine Nathel, N. F. Hong, X. Yang, Y. Houk, K. N. & Garg, N. K. Nickel-Catalyzed Activation of Acyl C-O Bonds of Methyl Esters. *Angew. Chem. Int. Ed.* **55**, 2810 (2016). (3) Zhang, S. Taylor, B. L. H. Ji, C. Gao, Y. Harris, M. R. Hanna, L. E. Jarvo, E. R. Houk, K. N. & Hong, X. Mechanism and Origins of Ligand-Controlled Stereoselectivity of Ni-Catalyzed Suzuki-Miyaura Coupling with Benzylic Esters: A Computational Study. *J. Am. Chem. Soc.* **139**, 12994 (2017). (4) Omer, H. M. & Liu, P. Computational Study of Ni-Catalyzed C–H Functionalization: Factors That Control the Competition of Oxidative Addition and Radical Pathways. *J. Am. Chem. Soc.* **139**, 9909 (2017). (5) Nohira, I. Liu, S. Bai, R. Lan, Y. & Chatani, N. Nickel-Catalyzed C-F/N-H Annulation of Aromatic Amides with Alkynes: Activation of C-F Bonds under Mild Reaction Conditions. *J. Am. Chem. Soc.* **142**, 17306 (2020). (6) Yao, W. Li, R. Chen, H. Chen, M. Luan, Y. Wang, Y. Yu, Z. & Ye, M. Ni-catalyzed hydroaminoalkylation of alkynes with amines. *Nat. Commun.* **12**, 3800 (2021).

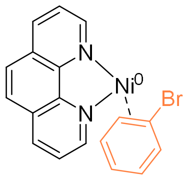

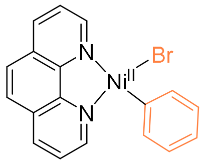

	INT1A-L4<br>multiplicity = 1	INT1A-L4-T<br>multiplicity = 3		INT2A-L4<br>multiplicity = 1	INT2A-L4-T<br>multiplicity = 3
ΔG/ kcal mol^{-1}	0.0	8.1	ΔG/ kcal mol^{-1}	0.0	1.2

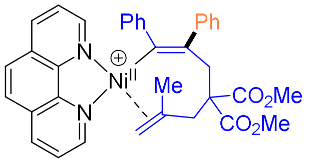

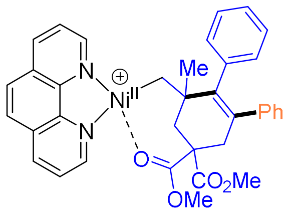

	INT5B-L4<br>multiplicity = 1	INT5B-L4-T<br>multiplicity = 3		INT5B-L4-exo<br>multiplicity = 1	INT5B-L4-exo-T<br>multiplicity = 3
ΔG/ kcal mol^{-1}	0.0	2.5	ΔG/ kcal mol^{-1}	0.0	6.1

Supplementary Figure 8. Comparison of relative stabilities of the key Ni-L4 complexes with different multiplicity.

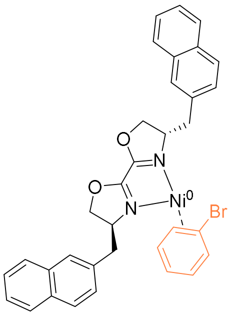

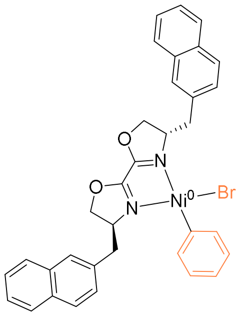

	INT1A-L9<br>multiplicity = 1	INT1A-L9-T<br>multiplicity = 3		INT2A-L9<br>multiplicity = 1	INT2A-L9-T<br>multiplicity = 3
ΔG/ kcal mol^{-1}	0.0	5.3	ΔG/ kcal mol^{-1}	0.0	7.0

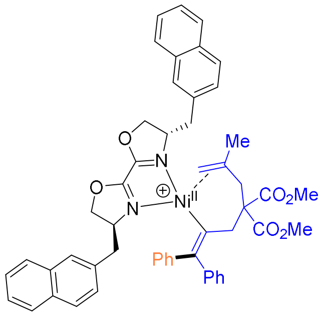

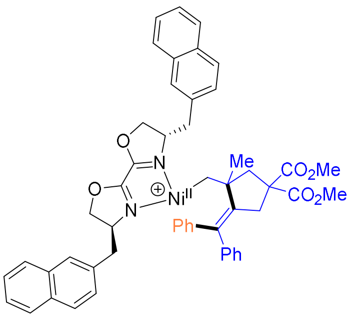

	INT5B-L9<br>multiplicity = 1	INT5B-L9-T<br>multiplicity = 3		INT5B-L9-exo<br>multiplicity = 1	INT5B-L9-exo-T<br>multiplicity = 3
ΔG/ kcal mol^{-1}	0.0	0.6	ΔG/ kcal mol^{-1}	0.0	9.8

Supplementary Figure 9. Comparison of relative stabilities of the key Ni-L9 complexes with different multiplicity.

The resulting intermediate **INT4B-L4** undergoes rapid *cis/trans* isomerization via reversible Ni–C bond homolysis or a η^2-coordinated alkenylnickel species to form intermediate **INT5B-L4**, which facilitates the proximity of the nickel(II) center to the C=C bond for the subsequent cyclization process. The energy barriers of the transition states for these two processes are comparable. Therefore, we cannot exclude either of them. Based on DFT conclusions and the reported literature[28-33], we can conclude that the differences in mechanism can mainly be attributed to the electronic effects of the ligands. When the ligand donates more electrons to the metal center, the charge on the metal center increases, leading to a decrease in the bond energy of the Ni-C bond, making it easier to break. However, electron-deficient ligands weaken the charge on the metal center, facilitating their η^2-coordination with alkenes, thus enabling isomerization reactions.

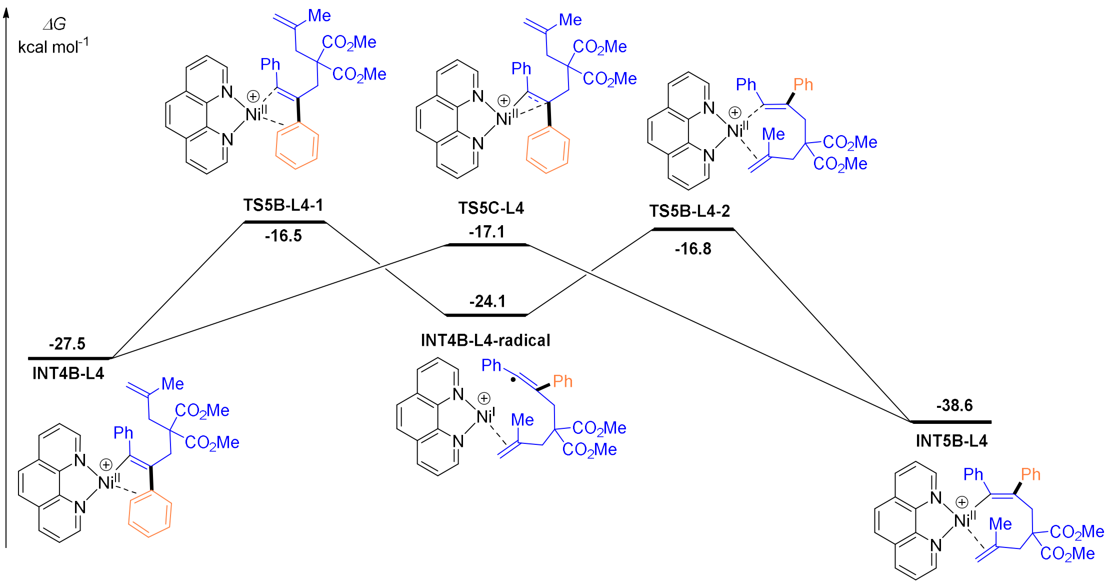

Supplementary Figure 10. cis/trans isomerization of intermediate INT4B-L4.

The previous reported 1,2-alkenyl/Ni(I) dyotropic and 1,2-alkyl/Ni(I) dyotropic rearrangement to form aza-seven membered-ring nickel(I) intermediate **INT6C-L4-exo-Ni(I)** and **INT6D-L4-exo-Ni(I)** has been proposed, respectively (34.7 kcal·mol^{-1} and 55.7 kcal·mol^{-1}) (**Figure 11**). These calculated high energy barriers well excluded the Ni(I)-mediated pathway.

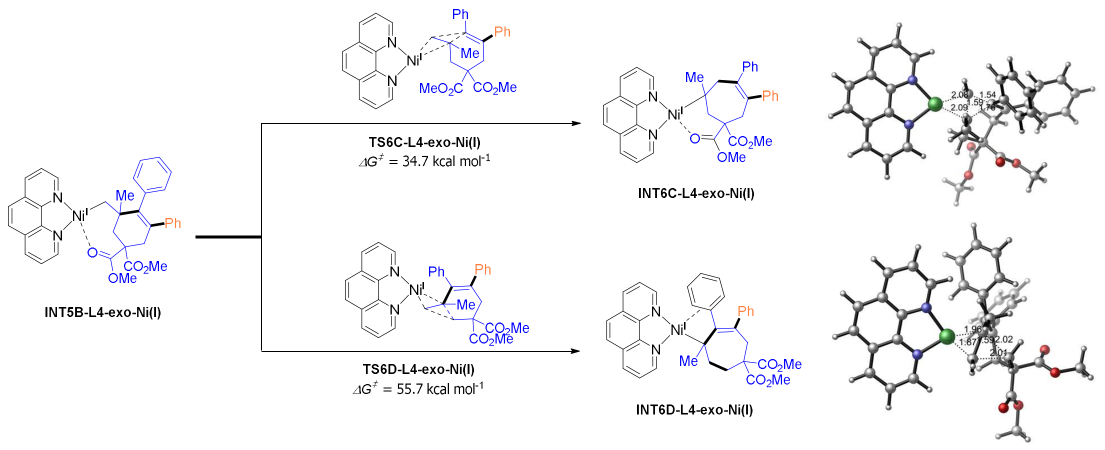

Supplementary Figure 11. The previous reported 1,2-alkenyl/Ni(I) dyotropic and 1,2-alkyl/Ni(I) dyotropic rearrangement in the presence of L4.

Generally, the radicals with high SOMO energy exhibited more nucleophilicity and the radicals with low SOMO energy showed more electrophilicity (**Figure 12**). The CF_3 group increases the electrophilic character of **INT10B**, which should be thermodynamically more favorable to recombine with intermediate **INT6B-L4** to generate Ni(II) complex **INT11C**.

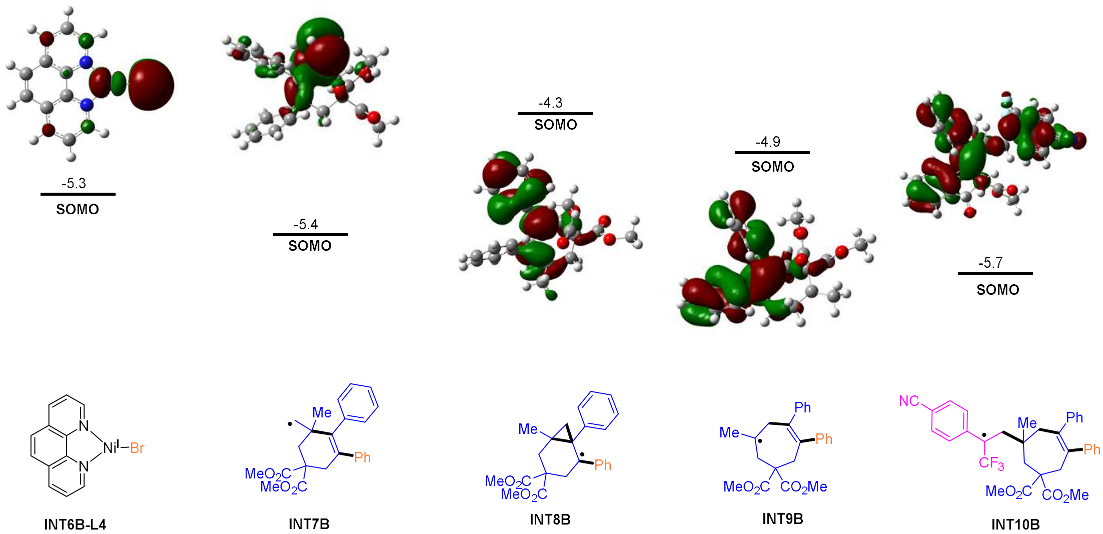

Supplementary Figure 12. The frontier molecular orbitals to analyze the nature of Ni(I) specie INT6B-L4 and the generated radicals (The calculations were conducted at B3LYP level of theory with Grimme D3 correction and a mix basis set of LANL08 for the Ni atom and the 6-31G(d) for another atoms level of theroy).

The recombination of intermediate **INT6B-L4** and radical INT10B is also clauclated. This process is a reversible process (**Figure 13**). If **INT11C** was formed,the energy barrier of the following β-F elimination was 27.0 kcal mol^{-1} (**Figure S10**), which was much higher than that of reductive process from **INT6B-L4** to **INT1A-L4** (**Scheme 6A**). Based on these computational results, we suspect that the recombination of **INT6B-L4** with radical **INT10B** is a reversible process, the radical pathway is the main pathway. The corresponding results have been added in Section 6 of the Supporting Information.

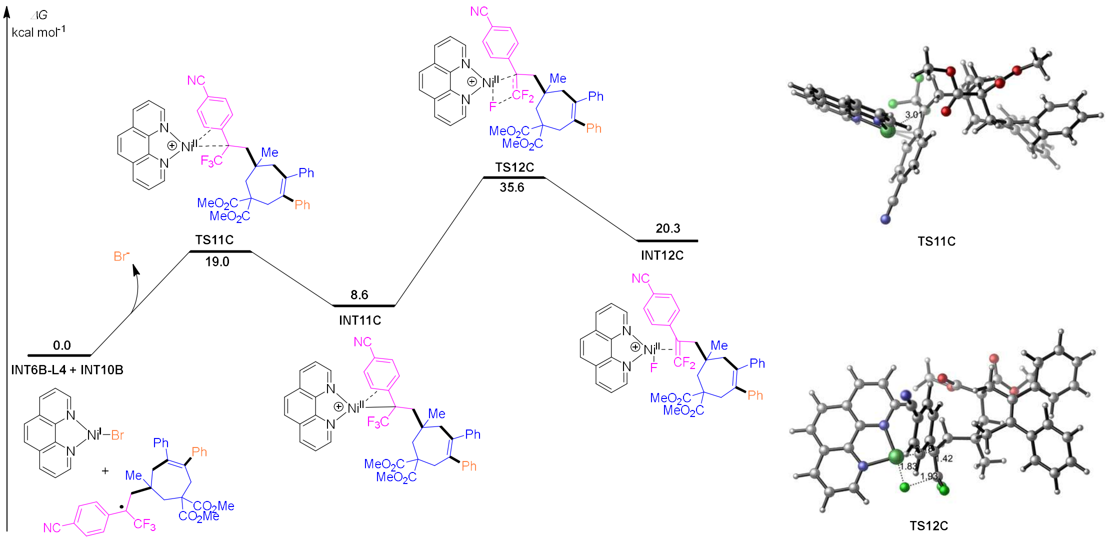

Supplementary Figure 13. The generation of intermediate INT11C and subsequent β-F elimination process.

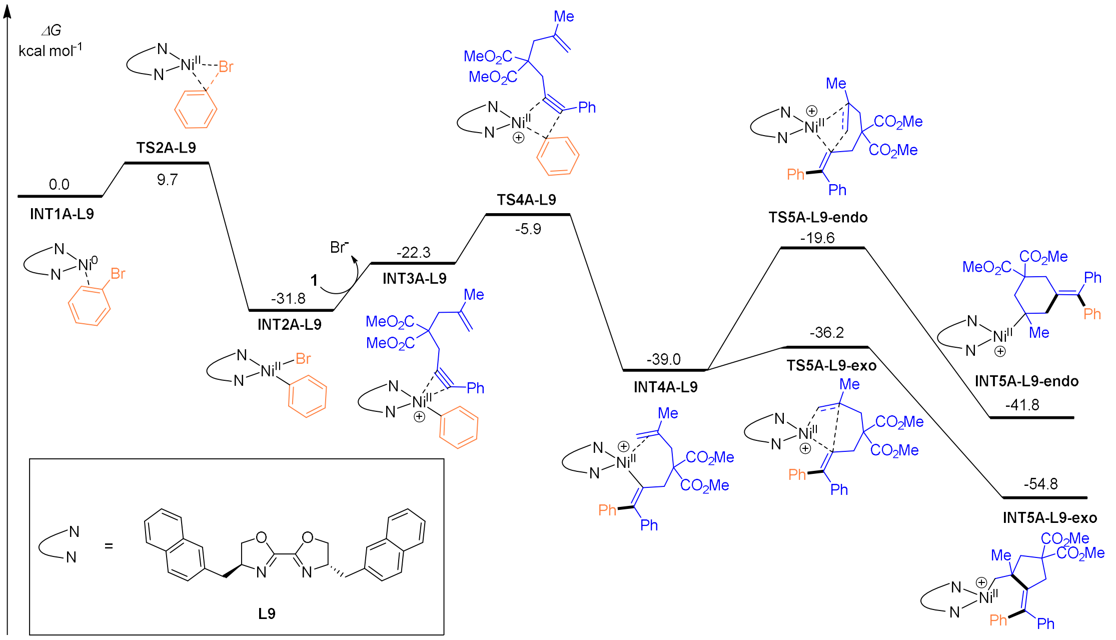

Supplementary Figure 14. The calculated free energy profile of the nickel catalytic cycle with L9 as ligand.

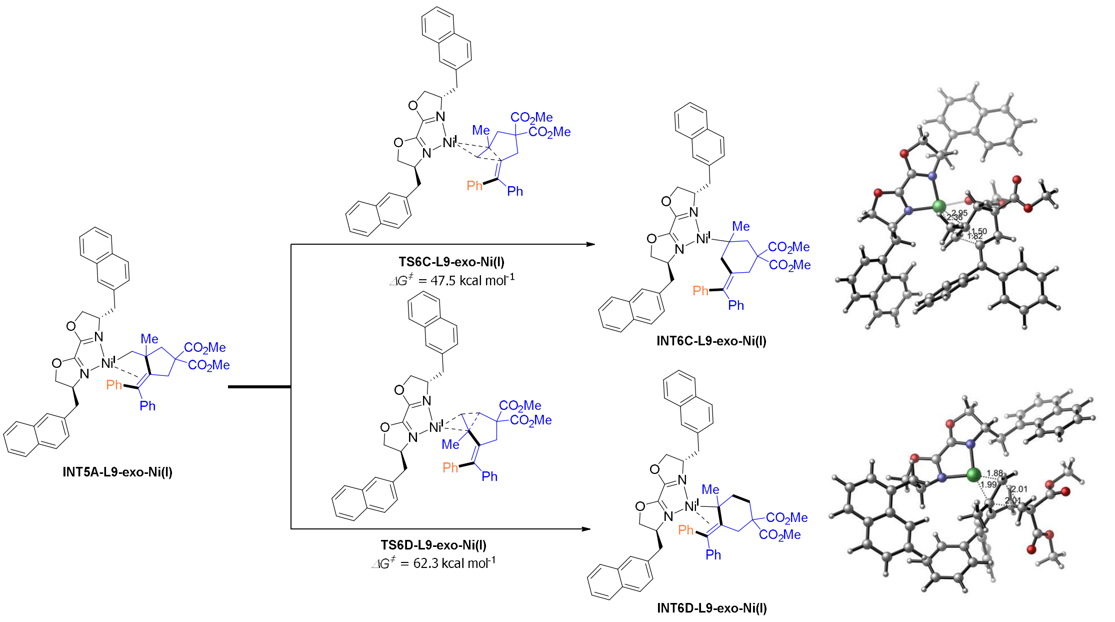

Supplementary Figure 15. The previous reported 1,2-alkenyl/Ni(I) dyotropic and 1,2-alkyl/Ni(I) dyotropic rearrangement in the presence of L9.

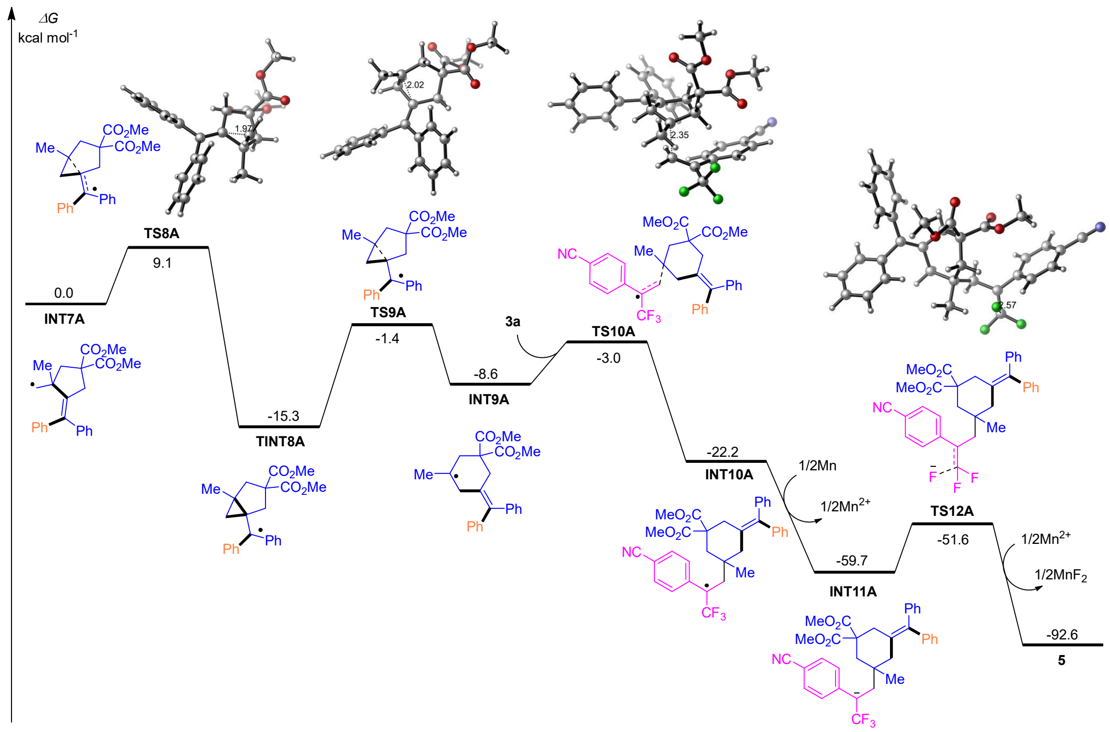

Supplementary Figure 16. The subsequent pathway for the radical INT7A.

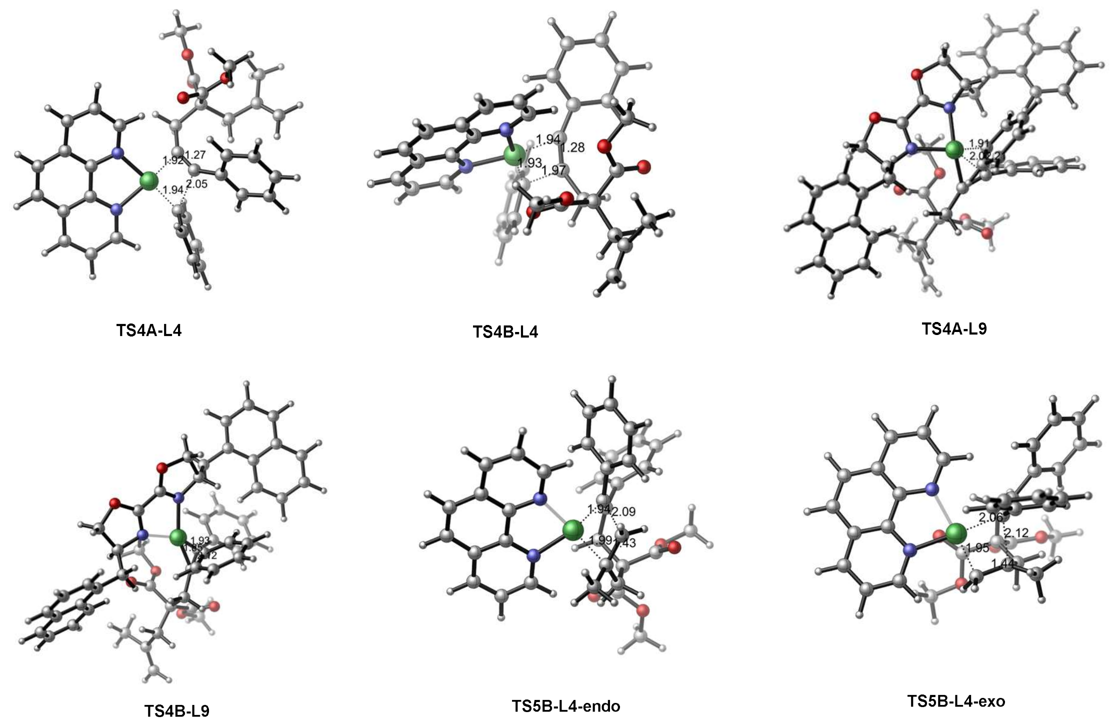

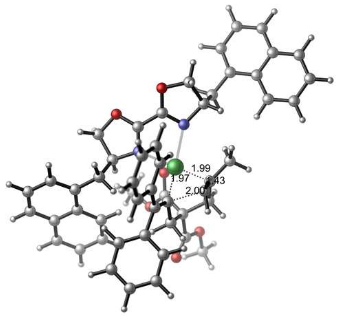

TS5A-L9-endo

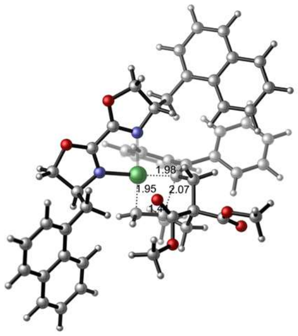

TS5A-L9-exo

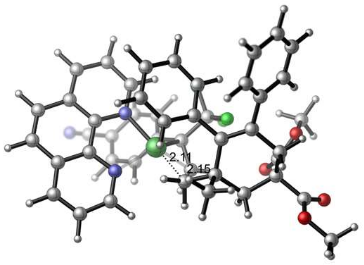

TS6A-L4-exo

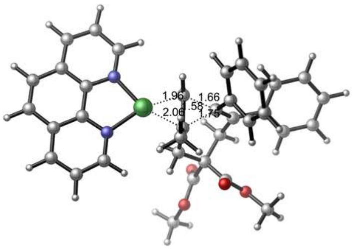

TS6C-L4-exo

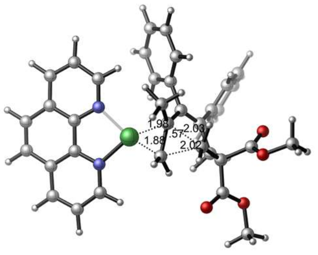

TS6D-L4-exo

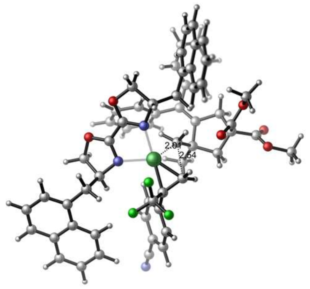

TS6A-L9-exo

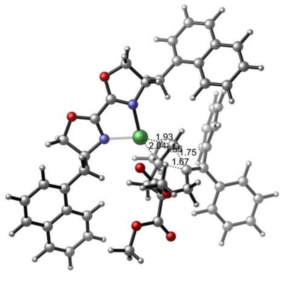

TS6C-L9-exo

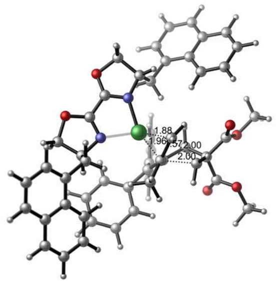

TS6D-L9-exo

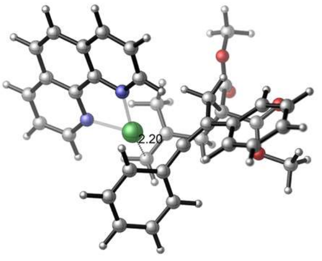

TS4B-L4-1

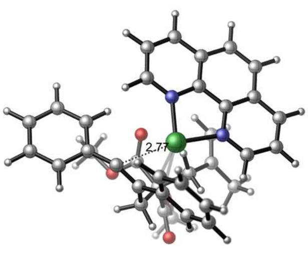

TS4B-L4-2

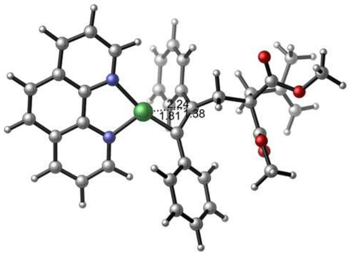

TS5C-L4

Supplementary Figure 17. The key transition states in the catalytic cycle.

Supplementary Table 4. The calculated energies of stationary points (in Hartree/Particle).

Structure	ZPE	H_{corr}	G_{corr}	E_{ele}	H_{sol}	G_{sol}
**1**	0.344546	0.368320	0.290095	-998.416570	-998.048250	-998.126475
**2**	0.090943	0.097570	0.060080	-2805.541956	-2805.444386	-2805.481876
**3**	0.138012	0.150634	0.099174	-738.724592	-738.573958	-738.625418
**INT1A-L4**	0.263593	0.282550	0.215569	-3547.895181	-3547.612631	-3547.679612
**TS2A-L4**	0.263060	0.281737	0.215351	-3547.877212	-3547.595475	-3547.661861
**INT2A-L4**	0.266091	0.284833	0.218248	-3547.946452	-3547.661619	-3547.728204
**INT3A-L4**	0.612361	0.653178	0.538278	-1972.174875	-1971.521697	-1971.636597
Br^-	0.000000	0.002360	-0.016176	-2574.182708	-2574.180348	-2574.198884
**TS4A-L4**	0.612015	0.651991	0.539319	-1972.147467	-1971.495476	-1971.608148
**INT4A-L4**	0.615878	0.655488	0.543161	-1972.216332	-1971.560844	-1971.673171
**TS4B-L4**	0.611757	0.651623	0.539307	-1972.155198	-1971.503575	-1971.615891
**INT4B-L4**	0.614701	0.654499	0.542542	-1972.193538	-1971.539039	-1971.650996
**TS5B-L4-1**	0.611318	0.651579	0.535129	-1972.168684	-1971.517105	-1971.633555
**INT4B-L4-radical**	0.612632	0.653179	0.537178	-1972.182742	-1971.529563	-1971.645564
**TS5B-L4-2**	0.612385	0.652085	0.538344	-1972.172377	-1971.520292	-1971.634033
**TS5C-L4**	0.614846	0.653877	0.545795	-1972.180244	-1971.526367	-1971.634449
**INT5B-L4**	0.616762	0.655744	0.545795	-1972.214507	-1971.558763	-1971.668712
**TS5B-L4-endo**	0.614680	0.653368	0.544448	-1972.190366	-1971.536998	-1971.645918
**INT5B-L4-endo**	0.619078	0.656935	0.549674	-1972.221754	-1971.564819	-1971.672080
**TS5B-L4-exo**	0.615046	0.653360	0.545092	-1972.195846	-1971.542486	-1971.650754
**INT5B-L4-exo**	0.617878	0.655021	0.550387	-1972.233436	-1971.578415	-1971.683049
**TS6B-L4-exo**	0.613207	0.652080	0.540696	-1972.203663	-1971.551583	-1971.662967
**INT6B-L4**	0.174124	0.187071	0.133390	-3316.441303	-3316.254232	-3316.307913
**INT7B**	0.437142	0.464769	0.378910	-1229.957643	-1229.492874	-1229.578733
Mn	0.000000	0.002360	-0.017354	-104.341466	-104.339106	-104.358820
$MnBr_2$	0.001671	0.007441	-0.028702	-5252.532789	-5252.525348	-5252.561491
**TS6A-L4-exo**	0.758400	0.808792	0.675227	-2710.945387	-2710.136595	-2710.270160

**INT6A-L4-exo**	0.761087	0.812020	0.674635	-2711.012141	-2710.200121	-2710.337506
**TS6C-L4-exo**	0.613871	0.652458	0.543106	-1972.159948	-1971.507490	-1971.616842
**INT6C-L4-exo**	0.618355	0.656412	0.548367	-1972.226305	-1971.569893	-1971.677938
**TS6D-L4-exo**	0.613702	0.652540	0.542011	-1972.141985	-1971.489445	-1971.599974
**INT6D-L4-exo**	0.618288	0.656489	0.548300	-1972.224359	-1971.567870	-1971.676059
**INT5B-L4-exo-Ni(I)**	0.614504	0.652762	0.543422	-1972.342128	-1971.689366	-1971.798706
**TS6C-L4-exo-Ni(I)**	0.609731	0.648660	0.537567	-1972.280916	-1971.632256	-1971.743349
**TS6D-L4-exo-Ni(I)**	0.609225	0.648465	0.535966	-1972.245938	-1971.597473	-1971.709972
**TS8B**	0.436818	0.463627	0.380007	-1229.948685	-1229.485058	-1229.568678
**INT8B**	0.439596	0.466316	0.382898	-1229.975957	-1229.509641	-1229.593059
**TS9B**	0.438050	0.464622	0.382100	-1229.952172	-1229.487550	-1229.570072
**INT9B**	0.438791	0.466169	0.380707	-1229.962467	-1229.496298	-1229.581760
**TS10B**	0.579049	0.618692	0.503843	-1968.698592	-1968.079900	-1968.194749
**INT10B**	0.583637	0.622575	0.510597	-1968.741351	-1968.118776	-1968.230754
**TS11B**	0.581337	0.620320	0.508771	-1968.692426	-1968.072106	-1968.183655
**INT11B**	0.581831	0.620773	0.509611	-1968.885819	-1968.265046	-1968.376208
Mn^{2+}	0.000000	0.002360	-0.017354	-104.171363	-104.169003	-104.188717
**TS12B**	0.580313	0.619255	0.508264	-1968.868656	-1968.249401	-1968.360392
MnF_2	0.003636	0.008646	-0.022588	-304.206927	-304.198281	-304.229515
**4**	0.580672	0.618957	0.507419	-1868.899308	-1868.280351	-1868.391889
**TS11C**	0.758535	0.809253	0.670238	-2710.979382	-2710.170129	-2710.309144
**INT11C**	0.761397	0.812087	0.674472	-2711.000475	-2710.188388	-2710.326003
**TS12C**	0.760480	0.810667	0.675028	-2710.958064	-2710.147397	-2710.283036
**INT12C**	0.761110	0.811935	0.675693	-2710.983067	-2710.171132	-2710.307374
**INT1A-L9**	0.552585	0.587052	0.482666	-4317.339238	-4316.752186	-4316.856572
**TS2A-L9**	0.551619	0.585803	0.482633	-4317.323692	-4316.737889	-4316.841059
**INT2A-L9**	0.555832	0.589517	0.490431	-4317.397630	-4316.808113	-4316.907199
**INT3A-L9**	0.900943	0.957689	0.805341	-2741.624976	-2740.667287	-2740.819635
**TS4A-L9**	0.900375	0.956070	0.807316	-2741.600830	-2740.644760	-2740.793514

**INT4A-L9**	0.905181	0.960633	0.811476	-2741.657744	-2740.697111	-2740.846268
**TS4B-L9**	0.899918	0.955956	0.804424	-2741.589293	-2740.633337	-2740.784869
**INT4B-L9**	0.905723	0.960264	0.815118	-2741.663034	-2740.702770	-2740.847916
**TS5A-L9-endo**	0.902956	0.957455	0.811456	-2741.626844	-2740.669389	-2740.815388
**INT5A-L9-endo**	0.906682	0.960722	0.815366	-2741.666139	-2740.705417	-2740.850773
**TS5A-L9-exo**	0.903522	0.957642	0.812740	-2741.654642	-2740.697000	-2740.841902
**INT5A-L9-exo**	0.906790	0.960450	0.817872	-2741.689438	-2740.728988	-2740.871566
**INT6A-L9**	0.462969	0.492380	0.397689	-4085.881602	-4085.389222	-4085.483913
**INT7A**	0.436576	0.464393	0.377222	-1229.950163	-1229.485770	-1229.572941
**TS6A-L9-exo**	1.044544	1.111661	0.937830	-3480.382215	-3479.270554	-3479.444385
**INT6A-L9-exo**	1.049770	1.116486	0.944151	-3480.454700	-3479.338214	-3479.510549
**TS6B-L9-exo**	0.901878	0.956484	0.808936	-2741.605360	-2740.648876	-2740.796424
**INT6B-L9-exo**	0.906358	0.960559	0.814635	-2741.671529	-2740.710970	-2740.856894
**TS6C-L9-exo**	0.902381	0.956824	0.809821	-2741.589469	-2740.632645	-2740.779648
**INT6C-L9-exo**	0.906708	0.961372	0.814582	-2741.703321	-2740.741949	-2740.888739
**INT5A-L9-exo-Ni(I)**	0.903789	0.957650	0.815357	-2741.805253	-2740.847603	-2740.989896
**TS6C-L9-exo-Ni(I)**	0.898742	0.953674	0.804562	-2741.718773	-2740.765099	-2740.914211
**TS6D-L9-exo-Ni(I)**	0.899507	0.954046	0.804956	-2741.695631	-2740.741585	-2740.890675
**TS8A**	0.436131	0.463400	0.376312	-1229.934753	-1229.471353	-1229.558441
**INT8A**	0.439630	0.466448	0.382182	-1229.979480	-1229.513032	-1229.597298
**TS9A**	0.437171	0.464287	0.378674	-1229.953768	-1229.489481	-1229.575094
**INT9A**	0.437795	0.465595	0.377830	-1229.964552	-1229.498957	-1229.586722
**TS10A**	0.577986	0.617992	0.502646	-1968.705848	-1968.087856	-1968.203202
**INT10A**	0.582750	0.621999	0.507961	-1968.741726	-1968.119727	-1968.233765
**INT11A**	0.580351	0.619745	0.506542	-1968.885137	-1968.265392	-1968.378595
**TS12A**	0.580009	0.618918	0.508397	-1968.874103	-1968.255185	-1968.365706
**5**	0.580177	0.618520	0.508488	-1868.906166	-1868.287646	-1868.397678

Note: ZPE = zero-point vibrational energy in the gas phase; H_{corr} = thermal correction to enthalpy in the gas phase; G_{corr} = thermal correction to Gibbs free energy in the gas

phase; E_{ele} = the electronic energies in solvent; $H_{sol} = H_{corr} + E_{ele}$; $G_{sol} = G_{corr} + E_{ele}$.

DFT-Computed Energies and Cartesian Coordinate (unit: angstrom)

1

C	-4.564450	0.312294	-1.332872
C	-3.405018	0.133422	-0.556523
C	-3.514082	0.094464	0.848705
C	-4.759747	0.232328	1.456093
C	-5.908214	0.409788	0.678473
C	-5.806028	0.449061	-0.714686
H	-4.480121	0.342638	-2.414982
H	-2.612750	-0.042746	1.439833
H	-4.835290	0.202407	2.539815
H	-6.878251	0.517677	1.156191
H	-6.696592	0.587195	-1.321948
C	-2.114773	-0.010772	-1.149681
C	-0.985195	-0.141321	-1.569038
C	0.433527	-0.321782	-1.857734
H	0.630956	-1.353915	-2.169346
H	0.781324	0.324048	-2.670768
C	1.290866	-0.011015	-0.586287
C	1.187202	1.507343	-0.233724
H	0.203698	1.658898	0.222781
H	1.199080	2.048575	-1.184743
C	2.279568	2.068104	0.658455
C	2.244802	1.724515	2.126337
H	3.003748	2.285090	2.681894
H	1.259410	1.937924	2.556092
H	2.421532	0.655402	2.280272
C	3.232364	2.850042	0.135854
C	0.750705	-0.888114	0.549062
O	0.182888	-0.490151	1.543090
O	0.921446	-2.192743	0.260812
C	2.752770	-0.319381	-0.906680
O	3.248424	-0.196005	-2.004801
O	3.439751	-0.665679	0.197500
C	4.864132	-0.771381	0.028935
H	5.274215	0.200878	-0.258240
H	5.250575	-1.081160	1.000059
H	5.106312	-1.509343	-0.740059
C	0.364640	-3.110743	1.217134
H	-0.715891	-2.962471	1.297975
H	0.590321	-4.105944	0.833421
H	0.821147	-2.961081	2.199421
H	4.019142	3.278675	0.752272
H	3.258953	3.092760	-0.923683

2

C	-2.181843	-1.208130	0.000002
C	-0.785384	-1.216383	0.000007
C	-0.103070	-0.000017	-0.000021
C	-0.785378	1.216378	-0.000004
C	-2.181813	1.208148	0.000012
C	-2.882349	0.000003	-0.000006
H	-2.719343	-2.152443	0.000004
H	-0.233765	-2.150180	0.000007
H	-0.233714	2.150150	-0.000005

H	-2.719333	2.152450	0.000017	N	1.234449	-1.350225	-0.376521
H	-3.968523	0.000029	-0.000011	Ni	-0.506142	-0.436236	-0.162420
Br	1.811249	0.000000	0.000001	Br	-3.080389	1.380845	-0.967870
				C	-2.330955	-0.169676	0.118981
**3**				C	-2.148005	-1.469827	-0.529784
C	1.509990	0.728697	0.213594	C	-2.730998	-0.150690	1.503315
C	2.015847	1.910887	0.585243	C	-2.451325	-2.657054	0.226450
H	3.084584	2.089249	0.601953	H	-2.226496	-1.534962	-1.615562
H	1.371479	2.726824	0.895966	C	-2.902859	-1.322291	2.198842
C	2.459008	-0.394839	-0.144659	H	-2.889095	0.809821	1.985483
F	3.726933	0.028435	-0.308248	C	-2.783273	-2.591945	1.555775
F	2.081878	-1.010261	-1.284983	H	-2.443789	-3.616249	-0.289104
F	2.483363	-1.345884	0.826575	H	-3.161247	-1.281248	3.254602
C	0.056397	0.441066	0.133180	H	-2.993515	-3.498027	2.118072
C	-0.464710	-0.829963	0.436052	C	2.960935	1.805659	0.389269
C	-0.837057	1.456646	-0.252438	C	2.550173	3.131183	0.659470
C	-1.832346	-1.071636	0.376990	H	3.295117	3.899081	0.850468
H	0.200207	-1.632311	0.730918	C	1.198985	3.429791	0.670574
C	-2.205616	1.226240	-0.310402	H	0.851074	4.438685	0.867578
H	-0.450891	2.431047	-0.534350	C	3.633341	-0.911100	-0.182533
C	-2.715100	-0.044001	0.007339	C	3.889288	-2.270929	-0.471348
H	-2.223467	-2.054423	0.619099	H	4.913810	-2.630997	-0.512378
H	-2.883287	2.017679	-0.613509	C	2.824067	-3.125287	-0.695631
C	-4.125447	-0.290661	-0.055406	H	2.985947	-4.175357	-0.916661
N	-5.270570	-0.489042	-0.105305	C	1.508688	-2.627069	-0.639477
				H	0.648214	-3.267117	-0.804755
**INT1A-L4**				C	0.256194	2.412959	0.424587
C	1.943165	0.856047	0.149922	H	-0.811676	2.607209	0.408134
C	2.280319	-0.506111	-0.144539	C	4.329821	1.370528	0.342452
N	0.614149	1.153581	0.179140	H	5.111810	2.101733	0.529542

C	4.651403	0.071075	0.071569
H	5.691491	-0.242619	0.042078

TS2A-L4

C	2.634981	-3.286577	-0.606786
C	3.726152	-2.482898	-0.323397
C	3.515893	-1.124475	-0.001004
C	2.178681	-0.664567	0.020088
C	1.342119	-2.736445	-0.575338
C	4.569602	-0.191433	0.291022
C	1.894101	0.703292	0.339924
C	2.949215	1.609299	0.596129
C	4.298915	1.117333	0.569377
C	2.592595	2.949767	0.861091
H	3.366860	3.683778	1.067844
C	1.255868	3.309949	0.839228
C	0.276646	2.334347	0.577021
H	5.595355	-0.549830	0.275882
H	2.761156	-4.333500	-0.862438
H	4.736556	-2.881875	-0.349833
H	0.467610	-3.336708	-0.803602
H	5.107548	1.813092	0.776998
H	0.949502	4.334606	1.022881
H	-0.778091	2.578784	0.517159
N	0.582554	1.058676	0.358310
N	1.107803	-1.458615	-0.271467
Ni	-0.612487	-0.491499	-0.069190
Br	-2.082609	1.116311	-1.829178
C	-2.318498	-0.068135	0.284402
C	-2.587491	-1.443056	0.028679
C	-2.962577	0.614896	1.335503
C	-3.393449	-2.154706	0.951205
H	-2.383329	-1.880422	-0.945966
C	-3.749776	-0.113046	2.213063
H	-2.797326	1.677837	1.481743
C	-3.955444	-1.500585	2.035213
H	-3.628006	-3.200099	0.761665
H	-4.202995	0.388724	3.065346
H	-4.591590	-2.041488	2.730466

INT2A-L4

C	0.619442	3.650093	0.000039
C	1.997698	3.552185	0.000034
C	2.593459	2.271595	0.000018
C	1.725732	1.156157	0.000007
C	-0.167339	2.484103	0.000025
C	4.011902	2.041440	0.000015
C	2.265108	-0.168574	-0.000003
C	3.663458	-0.372232	-0.000005
C	4.525172	0.776377	0.000003
C	4.112433	-1.712149	-0.000014
H	5.178647	-1.923074	-0.000017
C	3.183614	-2.736115	-0.000018
C	1.807836	-2.431677	-0.000013
H	4.673581	2.903271	0.000023
H	0.124926	4.615715	0.000053
H	2.624211	4.440087	0.000044
H	-1.248823	2.527975	0.000025
H	5.600255	0.618885	0.000001
H	3.495564	-3.775295	-0.000024

H	1.035651	-3.195337	-0.000015
N	1.360426	-1.180439	-0.000006
N	0.364006	1.259617	0.000006
Ni	-0.564489	-0.470188	-0.000005
Br	-1.565312	-2.546625	0.000001
C	-2.266506	0.330639	-0.000016
C	-2.888052	0.690858	1.204444
C	-2.887966	0.691018	-1.204474
C	-4.085607	1.414554	1.206664
H	-2.435262	0.401333	2.150166
C	-4.085522	1.414711	-1.206683
H	-2.435104	0.401625	-2.150202
C	-4.686562	1.782644	-0.000007
H	-4.552238	1.685409	2.151806
H	-4.552088	1.685690	-2.151822
H	-5.618489	2.342831	-0.000004

INT3A-L4

C	-1.517768	3.889319	2.183407
C	-0.938987	4.722453	1.245029
C	-0.512066	4.183170	0.010721
C	-0.698107	2.797103	-0.186510
C	-1.651536	2.516801	1.908185
C	0.083170	4.960046	-1.041171
C	-0.326129	2.198146	-1.429902
C	0.240348	2.983130	-2.457356
C	0.446637	4.384683	-2.223954
C	0.561271	2.322174	-3.665006
H	1.003806	2.881120	-4.484691
C	0.298449	0.970734	-3.789222
C	-0.263815	0.274494	-2.703088
H	0.232690	6.023015	-0.876932
H	-1.869537	4.271683	3.135331
H	-0.816892	5.784080	1.440910
H	-2.081708	1.834563	2.629313
H	0.891221	4.983056	-3.013591
H	0.523458	0.435395	-4.705105
H	-0.470880	-0.787881	-2.763893
N	-0.559378	0.867274	-1.550768
N	-1.239983	1.975225	0.759625
Ni	-1.177440	0.084627	0.243036
C	-1.890253	-0.488587	1.888793
C	-1.148950	-0.542400	3.074642
C	-3.254378	-0.801929	1.915234
C	-1.768587	-0.899932	4.278573
H	-0.090947	-0.298544	3.069644
C	-3.873500	-1.150679	3.121119
H	-3.841988	-0.783337	1.000492
C	-3.131765	-1.200950	4.303940
H	-1.183298	-0.942095	5.193871
H	-4.934002	-1.389115	3.131775
H	-3.612555	-1.477672	5.237804
O	2.021777	1.057015	0.033983
C	2.112705	0.100651	0.776517
C	2.230265	-1.361482	0.334035
O	2.076520	0.198325	2.117229
C	0.953818	-2.141801	0.770469
C	2.430399	-1.487262	-1.208980
C	3.430248	-2.045650	1.010206
C	2.022871	1.536676	2.649759

C	-0.282062	-1.817226	0.046912
H	0.783804	-2.007097	1.843085
H	1.160429	-3.207789	0.618990
H	1.738997	-0.780035	-1.673227
H	2.108380	-2.496843	-1.483165
C	3.832860	-1.258391	-1.740536
O	3.471875	-3.228651	1.260685
O	4.439091	-1.185621	1.197951
H	1.087776	2.021278	2.359288
H	2.075203	1.420246	3.731732
H	2.868205	2.121623	2.279894
C	-1.356603	-1.825728	-0.568339
C	4.333511	0.162194	-1.822975
C	4.574566	-2.300409	-2.134038
C	5.683417	-1.771229	1.640748
C	-2.512226	-2.189616	-1.342317
H	5.296677	0.211937	-2.339685
H	3.616234	0.801322	-2.352821
H	4.455482	0.595501	-0.825009
H	5.576012	-2.166351	-2.534695
H	4.211187	-3.323041	-2.069904
H	6.050764	-2.465735	0.881161
H	6.368807	-0.933627	1.764779
H	5.537578	-2.302011	2.584120
C	-3.409036	-1.222183	-1.832577
C	-2.746731	-3.553308	-1.606922
C	-4.519764	-1.613764	-2.575933
H	-3.229499	-0.171483	-1.624389
C	-3.859518	-3.933158	-2.352352
H	-2.059163	-4.299795	-1.221717
C	-4.746954	-2.967578	-2.837532
H	-5.209758	-0.862797	-2.948951
H	-4.036759	-4.985629	-2.552099
H	-5.614795	-3.270617	-3.415558

TS4A-L4

C	1.511807	-3.723179	1.494853
C	2.841309	-4.073563	1.359327
C	3.751423	-3.131921	0.826921
C	3.224424	-1.881404	0.435650
C	1.070231	-2.460711	1.053959
C	5.161648	-3.362681	0.680982
C	4.097979	-0.844546	-0.014736
C	5.482623	-1.090835	-0.147101
C	5.989812	-2.388221	0.203144
C	6.283482	-0.020420	-0.605836
H	7.353655	-0.160765	-0.729137
C	5.688673	1.194201	-0.889745
C	4.302381	1.351993	-0.706209
H	5.559409	-4.332719	0.964187
H	0.790707	-4.406656	1.929677
H	3.197694	-5.050582	1.673572
H	0.032631	-2.162775	1.162603
H	7.054235	-2.574613	0.095457
H	6.272854	2.035800	-1.245633
H	3.815154	2.296627	-0.910896
N	3.522181	0.364077	-0.269025
N	1.898256	-1.573643	0.499225
Ni	1.526362	0.283419	-0.111333
C	-0.360486	0.053044	-0.401429

C	-0.420445	1.210378	0.123931
C	-1.115521	-1.037618	-1.073388
C	-1.207383	2.176461	0.876931
C	1.300274	2.178587	-0.442983
H	-1.001591	-0.918867	-2.157373
H	-0.681806	-2.009622	-0.829828
C	-2.648326	-1.056533	-0.767969
C	-1.840506	1.729618	2.051476
C	-1.435824	3.483481	0.414409
C	1.802010	3.095840	0.499719
C	1.147230	2.579160	-1.779719
C	-3.298307	0.257190	-1.319467
C	-3.186551	-2.253729	-1.575804
C	-2.851720	-1.284966	0.732396
C	-2.705976	2.578041	2.740371
H	-1.656013	0.719024	2.399778
C	-2.308466	4.319944	1.105458
H	-0.937390	3.829110	-0.484795
C	2.173777	4.378049	0.100997
H	1.891370	2.801438	1.541581
C	1.547572	3.857426	-2.183679
H	0.706397	1.892084	-2.496209
H	-3.132218	0.211311	-2.403924
H	-2.723611	1.101840	-0.940145
C	-4.767559	0.517199	-1.062396
O	-3.185957	-2.277332	-2.786022
O	-3.596217	-3.266625	-0.796899
O	-1.961476	-1.597516	1.507250
O	-4.114659	-1.106240	1.105820
C	-2.944958	3.870553	2.267757

H	-3.193295	2.229930	3.646636
H	-2.493325	5.325003	0.738151
C	2.055830	4.757908	-1.243448
H	2.555786	5.083493	0.833826
H	1.441247	4.155333	-3.223078
C	-5.757580	-0.500843	-1.569492
C	-5.146049	1.646158	-0.454438
C	-4.114776	-4.419472	-1.498234
C	-4.409837	-1.307641	2.500535
H	-3.621794	4.529033	2.804259
H	2.344853	5.758681	-1.551120
H	-6.785952	-0.140909	-1.472428
H	-5.572119	-0.748120	-2.621997
H	-5.677170	-1.434769	-0.998254
H	-6.195954	1.887213	-0.308504
H	-4.424826	2.369794	-0.081831
H	-3.350123	-4.836616	-2.157967
H	-4.391365	-5.130580	-0.720629
H	-4.986171	-4.132894	-2.092034
H	-3.852527	-0.591055	3.109510
H	-5.481756	-1.138504	2.593997
H	-4.144992	-2.324409	2.800825

INT4A-L4

Ni	-0.434996	-0.691209	-0.606090
C	0.959806	0.578787	-0.441686
C	0.540057	1.860674	-0.554533
C	2.408240	0.223163	-0.157673
C	1.433125	3.052302	-0.417021
C	-0.912488	2.159977	-0.751200

H	3.047236	0.825312	-0.818472	H	-3.473900	3.733079	0.868770
H	2.668960	0.520399	0.861287	C	0.469725	-3.495689	-1.536183
C	2.848737	-1.252116	-0.301315	C	0.249349	-1.251079	-2.665927
C	1.486745	4.001906	-1.450535	C	6.234433	-0.886405	1.201365
C	2.186253	3.281053	0.744536	C	2.445893	-4.235994	1.872026
C	-1.657020	1.599505	-1.803156	H	3.672886	6.227431	-0.088924
C	-1.587126	2.959351	0.189065	H	-4.780869	2.663885	-0.959460
C	2.462681	-1.864214	-1.669138	H	-0.599438	-3.600564	-1.724230
C	4.392455	-1.255394	-0.227450	H	1.003845	-4.254855	-2.122619
C	2.342692	-2.094416	0.874384	H	0.660158	-3.721574	-0.484265
C	2.293870	5.133013	-1.337388	H	-0.708925	-1.527807	-3.091918
H	0.887917	3.847288	-2.344370	H	0.713257	-0.359467	-3.076101
C	2.986758	4.418090	0.864024	H	6.662089	-0.112458	0.559336
H	2.129947	2.574072	1.567897	H	6.378546	-0.642408	2.253304
C	-3.042727	1.777269	-1.878262	H	6.689295	-1.849564	0.957604
H	-1.142092	1.050106	-2.583785	H	1.360069	-4.349700	1.936529
C	-2.967541	3.129105	0.120731	H	2.921411	-5.188240	1.640141
H	-1.017971	3.423891	0.989239	H	2.822166	-3.830939	2.814313
H	3.002824	-2.808454	-1.781624	C	-4.379599	-2.387149	-1.911765
H	2.851490	-1.187905	-2.434859	C	-5.182393	-1.904374	-0.896781
C	0.990268	-2.130511	-1.916124	C	-4.589444	-1.224655	0.189487
O	5.127104	-1.464172	-1.163884	C	-3.182315	-1.068108	0.168210
O	4.802302	-0.951603	1.015143	C	-2.989857	-2.186415	-1.839015
O	1.641111	-1.696320	1.780368	C	-5.336742	-0.698190	1.295896
O	2.793510	-3.357539	0.780565	C	-2.528049	-0.383367	1.244197
C	3.047681	5.343863	-0.179471	C	-3.282202	0.093221	2.340765
H	2.330866	5.853111	-2.149973	C	-4.707918	-0.072223	2.331114
H	3.558057	4.583072	1.773449	C	-2.574727	0.711410	3.395312
C	-3.704562	2.526979	-0.907210	H	-3.116803	1.092655	4.256147
H	-3.598443	1.334799	-2.700909	C	-1.199774	0.810677	3.319609

C	-0.531144	0.332873	2.179766
H	-6.416004	-0.818608	1.296440
H	-4.798086	-2.919070	-2.759236
H	-6.259514	-2.043558	-0.922671
H	-2.348421	-2.563776	-2.627421
H	-5.278411	0.311401	3.171875
H	-0.622061	1.259556	4.120085
H	0.541634	0.393388	2.086196
N	-1.177952	-0.227135	1.158082
N	-2.395314	-1.547307	-0.835328

TS4B-L4

C	-0.087772	-2.109212	3.034920
C	-1.237144	-2.876769	3.033049
C	-2.316817	-2.500398	2.202140
C	-2.147612	-1.337614	1.416254
C	-0.021717	-0.952541	2.236974
C	-3.555680	-3.222066	2.112121
C	-3.175364	-0.931791	0.509162
C	-4.395406	-1.640975	0.454969
C	-4.557066	-2.802300	1.284755
C	-5.382752	-1.151802	-0.430814
H	-6.340517	-1.659129	-0.504876
C	-5.114512	-0.026940	-1.187451
C	-3.854984	0.593797	-1.089929
H	-3.683811	-4.109195	2.725338
H	0.764507	-2.374959	3.650703
H	-1.317981	-3.764191	3.654558
H	0.864717	-0.335968	2.219592
H	-5.494529	-3.348271	1.234883
H	-5.853901	0.381755	-1.867487
H	-3.607876	1.461862	-1.687882
N	-2.900198	0.144150	-0.277362
N	-1.030115	-0.559456	1.460931
Ni	-1.058118	0.845236	0.017655
C	0.623877	1.701296	0.484997
C	0.722624	1.469016	-0.771283
C	1.659841	0.971040	-1.821427
C	1.141283	2.230221	1.718781
C	-1.013989	1.952810	-1.567513
H	1.151320	0.865620	-2.778145
H	2.462611	1.706872	-1.944350
C	2.321123	-0.389298	-1.439188
C	0.353537	2.282780	2.885584
C	2.456342	2.745050	1.754983
C	-1.310367	1.379206	-2.820698
C	-1.310187	3.308921	-1.340018
C	3.209204	-0.826613	-2.662500
C	3.238737	-0.106942	-0.236004
C	1.233776	-1.447007	-1.230834
C	0.872796	2.813626	4.063624
H	-0.665803	1.910162	2.849147
C	2.964002	3.279210	2.936489
H	3.074115	2.699719	0.863936
C	-1.902211	2.151974	-3.818031
H	-1.085094	0.330674	-2.986568
C	-1.947767	4.063699	-2.327013
H	-1.027200	3.767169	-0.396741
H	3.991133	-0.061626	-2.745690
H	2.579115	-0.755116	-3.555214

C	3.841887	-2.200137	-2.600736
O	4.180947	0.651486	-0.326646
O	2.892740	-0.725060	0.903659
O	0.084679	-1.317285	-1.618045
O	1.687800	-2.561150	-0.654903
C	2.178386	3.312401	4.093915
H	0.254921	2.850329	4.956159
H	3.976691	3.671714	2.953607
C	-2.235062	3.490225	-3.569462
H	-2.119943	1.708685	-4.786126
H	-2.190053	5.105745	-2.138096
C	4.840554	-2.456674	-1.499153
C	3.535675	-3.123005	-3.518650
C	3.797525	-0.528113	2.014698
C	0.748065	-3.646803	-0.549300
H	2.579366	3.732956	5.011296
H	-2.701094	4.087258	-4.347966
H	5.367899	-3.402608	-1.651997
H	5.581606	-1.650290	-1.438827
H	4.337149	-2.510460	-0.525564
H	4.008774	-4.101456	-3.521777
H	2.814622	-2.931270	-4.309775
H	3.779330	0.514217	2.338138
H	3.429127	-1.185538	2.802756
H	4.812580	-0.806988	1.723427
H	0.379053	-3.925886	-1.539015
H	1.306291	-4.467199	-0.099626
H	-0.093944	-3.357619	0.085888

INT4B-L4

C	3.918365	3.126831	-0.499205
C	4.810692	2.368246	-1.235762
C	4.588534	0.979635	-1.374636
C	3.442799	0.446701	-0.744659
C	2.793125	2.514089	0.079663
C	5.449943	0.086077	-2.099467
C	3.178217	-0.955879	-0.797188
C	4.039875	-1.817364	-1.509290
C	5.184691	-1.251869	-2.168513
C	3.709385	-3.192033	-1.511295
H	4.336375	-3.897397	-2.049196
C	2.590075	-3.621326	-0.821421
C	1.798556	-2.686819	-0.127895
H	6.324925	0.498553	-2.592991
H	4.065330	4.192848	-0.364481
H	5.679153	2.825698	-1.700944
H	2.061834	3.084882	0.638245
H	5.845050	-1.914506	-2.720054
H	2.311502	-4.669281	-0.802468
H	0.925173	-2.990193	0.436431
N	2.080784	-1.386486	-0.117166
N	2.550019	1.209601	-0.049091
Ni	1.108207	0.167711	0.666494
C	-0.273726	1.299125	1.039156
C	-0.888998	0.225602	1.623521
C	-2.346930	-0.137952	1.394050
C	-0.714731	2.616606	0.655568
C	-0.010438	-0.675703	2.432835
H	-2.653063	-0.927940	2.081741
H	-2.967156	0.733674	1.623105

C	-2.710184	-0.609767	-0.057287
C	-1.525595	3.371884	1.532122
C	-0.305105	3.202217	-0.562379
C	-0.278637	-2.057624	2.616689
C	1.119525	-0.131058	3.109843
C	-1.676208	-1.637971	-0.614038
C	-4.086773	-1.291155	0.023404
C	-2.759627	0.624008	-0.968656
C	-1.897532	4.673566	1.206754
H	-1.843033	2.928209	2.471322
C	-0.676734	4.503763	-0.881874
H	0.276157	2.605605	-1.256698
C	0.583685	-2.859153	3.350968
H	-1.169747	-2.497799	2.184980
C	1.998709	-0.959994	3.818881
H	1.246600	0.945686	3.161524
H	-1.453086	-2.336834	0.196547
H	-0.764642	-1.075040	-0.828313
C	-2.081920	-2.440941	-1.839136
O	-4.476375	-1.913347	0.989293
O	-4.759995	-1.187791	-1.128570
O	-1.957088	0.882853	-1.840556
O	-3.774515	1.428324	-0.617374
C	-1.467339	5.245606	0.004686
H	-2.517482	5.245257	1.890939
H	-0.363256	4.939188	-1.826235
C	1.744768	-2.323368	3.930249
H	0.351405	-3.912413	3.481657
H	2.855125	-0.519296	4.320813
C	-2.729257	-3.778161	-1.564438
C	-1.825636	-2.009815	-3.077749
C	-6.037714	-1.855682	-1.173312
C	-3.946162	2.620452	-1.408889
H	-1.753494	6.263351	-0.244453
H	2.412938	-2.960844	4.501214
H	-3.080997	-4.255599	-2.483561
H	-2.015072	-4.460518	-1.080671
H	-3.577505	-3.691442	-0.875204
H	-2.107787	-2.596883	-3.948248
H	-1.355267	-1.048935	-3.257602
H	-6.696702	-1.462724	-0.395219
H	-6.437436	-1.646927	-2.165014
H	-5.908929	-2.931203	-1.026654
H	-4.047173	2.358127	-2.464951
H	-4.859000	3.085112	-1.036771
H	-3.092549	3.286903	-1.276964

TS5B-L4-1

C	-0.544056	-3.798871	-2.247428
C	-1.638519	-3.361479	-2.969787
C	-2.471224	-2.354715	-2.429929
C	-2.122865	-1.840904	-1.159933
C	-0.262739	-3.211477	-1.001147
C	-3.635957	-1.838846	-3.093376
C	-2.952727	-0.847985	-0.534818
C	-4.096154	-0.361148	-1.208759
C	-4.411571	-0.879922	-2.509960
C	-4.864626	0.622179	-0.546623
H	-5.748498	1.029609	-1.029218
C	-4.479132	1.053377	0.708781

C	-3.326394	0.506353	1.301402	C	-1.317238	0.859607	4.323311
H	-3.889072	-2.230510	-4.074191	H	-0.109746	2.068180	3.042475
H	0.107048	-4.578316	-2.627905	C	-0.990831	-1.527665	4.219262
H	-1.869675	-3.785953	-3.942706	H	0.637987	-2.191905	2.970864
H	0.602105	-3.511883	-0.420465	H	-0.723238	2.056009	0.890154
H	-5.289065	-0.495774	-3.021757	H	-0.461670	0.700625	-0.201110
H	-5.048879	1.805219	1.244181	C	-1.158756	2.483699	-1.156263
H	-2.988721	0.823064	2.281474	O	1.540384	4.264723	0.785206
N	-2.581515	-0.417925	0.700840	O	1.518566	3.933915	-1.447959
N	-1.017882	-2.248344	-0.476693	O	1.316442	0.393135	-1.894386
Ni	-0.737270	-1.157034	1.171560	O	3.144772	1.605653	-1.361341
C	2.027602	-0.948573	1.231625	C	5.158982	-3.048309	-0.653422
C	1.423888	0.120180	1.740480	H	6.222857	-2.771141	1.206324
C	1.839875	1.518229	1.269815	H	3.872366	-3.128361	-2.386438
C	3.065944	-1.598962	0.569713	C	-1.660065	-0.431602	4.751621
C	0.334400	-0.041833	2.765451	H	-1.810585	1.723016	4.761142
H	1.612639	2.265110	2.031559	H	-1.207498	-2.531350	4.572077
H	2.925559	1.527567	1.143971	C	-1.739254	3.821141	-0.762391
C	1.202113	2.015861	-0.071756	C	-1.415210	1.910336	-2.336572
C	4.281834	-1.868666	1.271315	C	1.696701	5.351715	-1.648010
C	2.940673	-2.075443	-0.770238	C	3.876491	0.991492	-2.441994
C	-0.348544	1.055872	3.345409	H	5.962411	-3.607302	-1.123213
C	0.006485	-1.346410	3.244319	H	-2.421754	-0.570566	5.512550
C	-0.335029	1.779864	-0.093593	H	-2.233420	4.314645	-1.604363
C	1.469807	3.525490	-0.173828	H	-2.480541	3.693307	0.039525
C	1.860202	1.251746	-1.228328	H	-0.975688	4.500226	-0.365406
C	5.303698	-2.579046	0.660555	H	-2.024549	2.409389	-3.086037
H	4.390045	-1.510310	2.290412	H	-0.999087	0.943327	-2.599110
C	3.976747	-2.785093	-1.360606	H	2.621369	5.686866	-1.172095
H	2.052491	-1.820640	-1.334455	H	1.742572	5.487069	-2.728012

H	0.853278	5.903409	-1.224592
H	3.333470	1.117459	-3.381842
H	4.833390	1.511748	-2.474156
H	4.022840	-0.071569	-2.240297

INT4B-L4-radical

C	-0.586879	-2.530485	-1.889925
C	-1.732941	-3.205013	-1.516718
C	-2.848158	-2.474168	-1.045723
C	-2.722833	-1.067680	-0.999689
C	-0.554832	-1.124414	-1.809132
C	-4.068875	-3.080495	-0.593563
C	-3.812684	-0.267206	-0.513086
C	-4.998689	-0.889971	-0.061548
C	-5.097502	-2.322008	-0.115468
C	-6.028746	-0.048791	0.417801
H	-6.957001	-0.485854	0.775019
C	-5.843889	1.321379	0.424587
C	-4.631448	1.851922	-0.049389
H	-4.155784	-4.162440	-0.633363
H	0.297938	-3.062479	-2.221553
H	-1.782251	-4.289326	-1.565024
H	0.340315	-0.566810	-2.059415
H	-6.014040	-2.791169	0.229829
H	-6.615814	1.992292	0.785113
H	-4.454638	2.922783	-0.060567
N	-3.641248	1.085135	-0.506066
N	-1.596499	-0.412293	-1.384937
Ni	-1.838391	1.615277	-1.167374
C	2.658520	-1.456890	1.088509
C	1.930670	-0.557271	1.713886
C	2.458134	0.875737	1.820424
C	3.184419	-2.547125	0.433885
C	0.554253	-0.887183	2.213202
H	2.081722	1.396345	2.702475
H	3.546682	0.865453	1.900399
C	2.071979	1.735868	0.570529
C	3.928837	-3.555527	1.126994
C	3.023047	-2.688169	-0.986450
C	-0.078796	-0.128467	3.211736
C	-0.153565	-1.971141	1.661718
C	0.524863	1.709740	0.392767
C	2.526453	3.180413	0.802705
C	2.767456	1.121717	-0.650568
C	4.449319	-4.640153	0.439729
H	4.067938	-3.458822	2.198995
C	3.559693	-3.780663	-1.648930
H	2.505841	-1.903689	-1.528571
C	-1.379417	-0.425309	3.625818
H	0.442073	0.697705	3.684044
C	-1.447168	-2.272091	2.079945
H	0.319455	-2.575133	0.894089
H	0.084027	1.853562	1.381372
H	0.279972	0.692000	0.091910
C	-0.119039	2.712682	-0.553724
O	2.592054	3.707747	1.890283
O	2.753288	3.813953	-0.361909
O	2.203962	0.553292	-1.570075
O	4.092378	1.219455	-0.543211
C	4.270043	-4.767622	-0.946768

H	5.004133	-5.399629	0.983495
H	3.436931	-3.867617	-2.725714
C	-2.074574	-1.493136	3.056858
H	-1.842095	0.175073	4.404395
H	-1.970049	-3.116881	1.641653
C	-0.630648	3.983883	0.096099
C	-0.116309	2.572137	-1.926021
C	3.156517	5.196359	-0.254749
C	4.871492	0.596406	-1.588754
H	4.687111	-5.619603	-1.474669
H	-3.084741	-1.727265	3.380953
H	-1.113691	4.648488	-0.626551
H	-1.337491	3.761387	0.904582
H	0.202493	4.528758	0.557633
H	-0.446109	3.397784	-2.555838
H	0.460565	1.791470	-2.409557
H	4.065901	5.278166	0.345033
H	3.333361	5.526802	-1.277698
H	2.364773	5.788382	0.212626
H	4.586851	1.001629	-2.562635
H	5.908019	0.836227	-1.355388
H	4.711129	-0.484396	-1.576219

TS5B-L4-2

C	-5.522250	-0.055047	-1.839579
C	-6.070763	0.457917	-0.680428
C	-5.256432	0.607636	0.464426
C	-3.894715	0.231604	0.350228
C	-4.154893	-0.382202	-1.860217
C	-5.748041	1.101219	1.718969
C	-3.036277	0.295878	1.503290
C	-3.559509	0.763377	2.734845
C	-4.931304	1.180333	2.807122
C	-2.693548	0.773643	3.850973
H	-3.057225	1.129239	4.810959
C	-1.400047	0.314570	3.704335
C	-0.971242	-0.137242	2.444264
H	-6.789393	1.402131	1.786117
H	-6.121473	-0.201850	-2.731621
H	-7.119988	0.735799	-0.633993
H	-3.698836	-0.780955	-2.759495
H	-5.310580	1.546966	3.756481
H	-0.709091	0.291810	4.540092
H	0.034617	-0.512363	2.302934
N	-1.751599	-0.138490	1.362698
N	-3.353128	-0.227038	-0.809570
Ni	-1.286354	-0.591939	-0.637179
C	1.094185	-1.371491	0.137463
C	0.470380	-2.421902	-0.491179
C	1.914808	-0.488987	0.671749
C	-0.768472	-2.988706	-0.006782
C	1.001634	-2.946322	-1.727977
C	1.616509	0.997253	0.443790
C	3.088955	-0.906811	1.496166
C	-1.437421	-3.956339	-0.752691
H	-1.126119	-2.715284	0.980027
C	0.315445	-3.911315	-2.436018
H	1.955175	-2.564665	-2.078745
H	0.542434	1.165347	0.553122
H	2.101593	1.609367	1.203835

C	2.027554	1.519816	-0.963572
C	4.089132	0.002349	1.881704
C	3.225955	-2.252600	1.888034
C	-0.918992	-4.410288	-1.970945
H	-2.363138	-4.374442	-0.367491
H	0.736036	-4.296076	-3.360905
C	1.300056	0.798502	-2.123709
C	1.817095	3.041356	-1.107582
C	3.556211	1.346601	-1.072907
C	5.196296	-0.427110	2.617779
H	4.039375	1.044525	1.590033
C	4.325803	-2.676251	2.626197
H	2.458401	-2.969806	1.610318
H	-1.448386	-5.166888	-2.541245
H	1.654728	-0.228945	-2.175412
H	1.629401	1.290054	-3.048480
C	-0.220648	0.805876	-2.182560
O	1.864138	3.606167	-2.178094
O	1.575564	3.656233	0.058180
O	4.340679	2.167006	-0.647476
O	3.906867	0.164834	-1.597466
C	5.320349	-1.763945	2.994269
H	5.960954	0.292738	2.895316
H	4.407792	-3.719532	2.917698
C	-0.955398	2.105346	-1.944377
C	-0.850096	-0.263610	-2.767493
C	1.464209	5.094665	-0.017456
C	5.324875	-0.123169	-1.585089
H	6.179520	-2.093730	3.570905
H	-2.033177	1.978275	-2.073443
H	-0.776199	2.494814	-0.936189
H	-0.595413	2.867083	-2.646209
H	-1.853889	-0.154176	-3.163799
H	-0.288724	-1.126978	-3.112798
H	0.636386	5.376073	-0.673422
H	1.282551	5.425370	1.004733
H	2.392722	5.519133	-0.405865
H	5.673974	-0.194656	-0.552094
H	5.428363	-1.079751	-2.096184
H	5.874207	0.662812	-2.107475

TS5C-L4

Ni	-1.571800	-0.035799	-0.234876
C	-0.357416	-0.974942	2.143110
C	0.197061	-1.324355	0.889696
C	0.299764	-2.697122	0.564630
C	-0.140160	-3.671325	1.452861
C	-0.706546	-3.308096	2.682505
C	-0.812859	-1.960057	3.022400
H	-0.383807	0.070356	2.434569
H	0.728437	-2.995673	-0.387171
H	-0.043201	-4.720719	1.189319
H	-1.043787	-4.074367	3.373974
H	-1.225503	-1.669682	3.984152
C	1.842108	-0.486671	-0.966662
H	1.790182	0.214805	-1.801616
H	1.748619	-1.485178	-1.401542
C	0.653979	-0.244896	-0.044422
C	-0.057637	0.938301	-0.070641
C	0.260147	2.339656	0.005483

C	-0.412369	3.283645	-0.805960
C	1.171694	2.803132	0.979011
C	-0.158620	4.642805	-0.665921
H	-1.118346	2.922753	-1.548772
C	1.399823	4.169053	1.132909
H	1.693475	2.090211	1.605118
C	0.744216	5.088796	0.309193
H	-0.662367	5.358615	-1.308919
H	2.102534	4.511607	1.886353
H	0.934231	6.151994	0.424843
C	-4.405155	2.876298	1.053613
C	-4.201192	-0.913536	-0.666467
C	-5.183833	-2.930044	-1.541942
C	-3.902175	-3.423909	-1.706100
N	-2.943507	-1.402817	-0.841431
H	-6.048978	-3.526304	-1.817891
H	-3.730832	-4.414691	-2.112529
C	-4.302746	0.394764	-0.093587
C	-5.571012	0.969297	0.141562
C	-5.597121	2.253101	0.732855
H	-6.549577	2.735448	0.933291
H	-4.390751	3.858533	1.513192
C	-5.366952	-1.636086	-1.001698
C	-6.743605	0.221162	-0.220412
H	-7.716983	0.669029	-0.043497
C	-6.646610	-1.026072	-0.767583
H	-7.541919	-1.581396	-1.031091
C	-3.185844	2.230554	0.780770
H	-2.234797	2.697312	1.007124
C	-2.800688	-2.627488	-1.340240

H	-1.782150	-2.985981	-1.442983
C	3.284056	-0.411590	-0.362375
C	3.398431	-1.161248	1.002769
C	4.214097	-1.095733	-1.383840
C	3.686962	1.060873	-0.203410
H	2.831560	-2.091440	0.904030
H	2.882263	-0.545801	1.744945
C	4.800206	-1.501755	1.485558
O	3.880693	-2.023353	-2.091659
O	5.450802	-0.588978	-1.338013
O	3.927301	1.617254	0.846327
O	3.682434	1.686042	-1.393888
C	5.311335	-2.869902	1.096669
C	5.506456	-0.670917	2.256803
C	6.424174	-1.215984	-2.197931
C	4.077666	3.071947	-1.379327
H	6.354435	-3.011870	1.394263
H	4.711755	-3.654726	1.579527
H	5.234318	-3.051040	0.017544
H	6.499064	-0.938902	2.610280
H	5.124612	0.302547	2.543765
H	6.103287	-1.154138	-3.240738
H	7.347926	-0.660509	-2.040113
H	6.552415	-2.265373	-1.920468
H	5.067936	3.175865	-0.928940
H	4.093459	3.379364	-2.424831
H	3.356136	3.664636	-0.812091
N	-3.130909	1.024165	0.216858

INT5B-L4

C	5.015765	-1.498883	-2.275322	C	-0.157857	4.821865	-0.068503
C	5.828170	-1.267148	-1.183548	H	-0.959130	3.250397	1.156597
C	5.272108	-0.689748	-0.020718	C	0.764707	4.148422	-2.194407
C	3.889783	-0.380956	-0.043387	H	0.696312	2.051997	-2.634512
C	3.652136	-1.165796	-2.197448	H	-0.395095	-1.193960	0.972948
C	6.036164	-0.405599	1.160273	H	-1.903124	-0.975321	1.834667
C	3.279728	0.228644	1.101352	C	-2.139865	-1.879472	-0.105820
C	4.054665	0.494289	2.255293	C	-3.482336	1.249288	1.721776
C	5.450429	0.159566	2.253763	C	-3.344159	1.995575	-0.568571
C	3.400087	1.085118	3.359014	C	0.460252	5.155949	-1.278352
H	3.960538	1.304515	4.263322	H	-0.401725	5.599159	0.650437
C	2.053528	1.373762	3.267820	H	1.242125	4.395677	-3.138498
C	1.362744	1.084210	2.079223	C	-1.716454	-1.642818	-1.573724
H	7.093947	-0.651015	1.164524	C	-1.900117	-3.351448	0.273281
H	5.405960	-1.934104	-3.188805	C	-3.665298	-1.705113	0.092084
H	6.884727	-1.518305	-1.208893	C	-4.693080	1.928137	1.868709
H	3.006375	-1.350309	-3.047285	H	-3.084247	0.691987	2.564312
H	6.033436	0.370342	3.145350	C	-4.554079	2.669850	-0.424832
H	1.515049	1.824965	4.093934	H	-2.824710	2.012458	-1.521609
H	0.309308	1.301675	1.971564	H	0.700743	6.191007	-1.502436
N	1.951663	0.530990	1.017850	H	-2.147656	-0.701870	-1.911075
N	3.087591	-0.630465	-1.117065	H	-2.163301	-2.439211	-2.182426
Ni	1.124168	0.052405	-0.720358	C	-0.235854	-1.657906	-1.881783
C	-0.432303	1.054974	-0.332209	O	-2.157110	-4.271333	-0.474552
C	-0.179201	2.462181	-0.696876	O	-1.393763	-3.500037	1.503748
C	-1.493303	0.547093	0.330723	O	-4.202342	-1.948279	1.150216
C	-0.465680	3.496635	0.221743	O	-4.289898	-1.244949	-0.994726
C	0.452662	2.819118	-1.906749	C	-5.232972	2.642445	0.797415
C	-1.436082	-0.881824	0.851841	H	-5.215226	1.893526	2.820563
C	-2.781598	1.280806	0.504468	H	-4.971417	3.212482	-1.268460

C	0.542079	-2.902391	-1.540221
C	0.294957	-0.633855	-2.632834
C	-1.256390	-4.865900	1.954533
C	-5.695010	-0.948395	-0.820441
H	-6.177171	3.167355	0.910573
H	1.548394	-2.889844	-1.960704
H	0.629937	-3.020232	-0.453631
H	0.003253	-3.783998	-1.904184
H	1.222272	-0.755552	-3.181957
H	-0.360607	0.154975	-2.989285
H	-0.582896	-5.417587	1.293515
H	-0.847142	-4.798482	2.962030
H	-2.233153	-5.354887	1.963076
H	-5.810513	-0.160527	-0.072893
H	-6.040151	-0.610811	-1.796956
H	-6.231633	-1.844786	-0.502174
H	-0.650025	-1.400018	0.998949
H	-2.142991	-0.812858	1.667515
C	-2.348444	-1.516391	-0.337899
C	-2.024803	1.572248	2.664781
C	-2.619838	2.716538	0.621586
C	1.156030	5.102100	-0.911512
H	0.563018	4.936465	-2.978162
H	1.608270	4.964277	1.195699
C	-1.504784	-2.077333	-1.522189
C	-2.987732	-2.765258	0.292372
C	-3.442750	-0.553616	-0.816777
C	-2.760413	2.502336	3.398823
H	-1.500775	0.773612	3.183731
C	-3.364954	3.638497	1.354542
C	-0.281472	-1.319511	-2.008176
H	-2.571274	2.795127	-0.459838
H	1.534559	6.114812	-1.014363

TS5B-L4-endo

Ni	0.979068	-0.382791	-0.782418
C	-0.263908	1.069943	-0.455485
C	0.173076	2.470402	-0.649218
C	-1.176953	0.664461	0.466173
C	0.126234	3.135643	-1.885728
C	0.716684	3.155989	0.456252
C	-1.529057	-0.796102	0.767056
C	-1.938437	1.669415	1.266195
C	0.614380	4.438887	-2.013900
H	-0.307652	2.651357	-2.753044
C	1.197227	4.455656	0.328235
H	0.757325	2.651920	1.416382
H	-2.179598	-2.224457	-2.375086
H	-1.174351	-3.083553	-1.237666
O	-2.570269	-3.317280	1.287377
O	-4.001518	-3.215033	-0.460356
O	-3.476168	-0.012186	-1.903149
O	-4.333050	-0.346688	0.159957
C	-3.434861	3.537216	2.746504
H	-2.807556	2.417906	4.480801
H	-3.891869	4.436033	0.838095
C	-0.382870	0.076525	-2.288227
C	0.621622	-2.159456	-2.901258
C	-4.646151	-4.420319	0.006165
C	-5.399821	0.581858	-0.130559

H	-4.011928	4.258103	3.318293
H	1.502174	-1.601140	-3.235768
H	0.965812	-3.067535	-2.396885
H	0.068924	-2.473080	-3.798716
H	0.322466	0.514259	-2.994494
H	-1.363802	0.534859	-2.305921
H	-5.065658	-4.259808	1.002272
H	-5.433192	-4.626865	-0.718165
H	-3.927322	-5.242853	0.045589
H	-5.928540	0.276428	-1.036284
H	-6.056229	0.545517	0.738011
H	-4.990226	1.585970	-0.258294
C	4.556433	2.180865	-0.534956
C	5.407499	1.362033	0.179904
C	4.961164	0.080052	0.575317
C	3.642627	-0.282348	0.215695
C	3.246962	1.745448	-0.815729
C	5.768073	-0.857535	1.304657
C	3.159141	-1.596674	0.519204
C	3.966601	-2.497725	1.252223
C	5.287013	-2.089159	1.641055
C	3.414602	-3.763131	1.553832
H	3.997213	-4.483706	2.120778
C	2.135904	-4.064659	1.124843
C	1.421026	-3.117520	0.372152
H	6.774639	-0.563695	1.587524
H	4.868346	3.163440	-0.871416
H	6.413992	1.684010	0.432133
H	2.557887	2.381960	-1.354673
H	5.901738	-2.787888	2.200627
H	1.675600	-5.020816	1.347948
H	0.421983	-3.335275	0.017704
N	1.915715	-1.918508	0.061531
N	2.786121	0.556921	-0.430375

INT5B-L4-endo

C	2.040082	1.316480	-0.395070
C	2.901042	2.494288	-0.115031
C	2.516620	0.139583	-0.866603
C	3.869905	2.917078	-1.042012
C	2.755474	3.226144	1.076544
C	1.527533	-0.972712	-1.139772
C	3.956213	-0.231807	-0.933955
C	4.673556	4.024146	-0.779231
H	3.988438	2.367864	-1.970474
C	3.567320	4.328319	1.344934
H	2.016454	2.917255	1.811429
H	0.606114	-0.589722	-1.591492
H	1.941194	-1.738394	-1.797997
C	1.157885	-1.667773	0.225048
C	4.495104	-0.844336	-2.075947
C	4.787103	-0.052261	0.185544
C	4.528911	4.732514	0.416816
H	5.413244	4.336408	-1.511102
H	3.449101	4.870115	2.279387
C	0.774887	-0.635781	1.305498
C	-0.063762	-2.500992	-0.039318
C	2.365377	-2.561173	0.603609
C	5.828543	-1.250741	-2.106186
H	3.868072	-0.996880	-2.950684

C	6.118560	-0.465232	0.159091
H	4.380562	0.415958	1.076607
H	5.158025	5.594037	0.621451
H	1.703772	-0.202712	1.688590
H	0.327463	-1.169886	2.150439
C	-0.153657	0.514880	0.871385
O	-1.215257	-2.044782	0.091740
O	0.132935	-3.729527	-0.452309
O	2.908002	-3.308281	-0.176037
O	2.746174	-2.381357	1.875043
C	6.644483	-1.065608	-0.987975
H	6.229672	-1.716070	-3.002065
H	6.745816	-0.316268	1.033918
C	-0.539340	1.251028	2.166834
C	0.538429	1.483532	-0.154419
C	-1.016736	-4.542697	-0.780995
C	3.955283	-3.078688	2.254237
H	7.681583	-1.387822	-1.009646
H	-1.160464	2.132630	1.993196
H	-1.088569	0.593076	2.851560
H	0.360191	1.589794	2.704726
H	0.037696	1.412374	-1.131009
H	0.390349	2.514501	0.177126
H	-1.652266	-4.023009	-1.501704
H	-0.603446	-5.453644	-1.210294
H	-1.579679	-4.769514	0.128297
H	4.787594	-2.718729	1.643859
H	4.107820	-2.838284	3.305978
H	3.833610	-4.154801	2.111187
Ni	-1.861880	-0.234361	0.237507
N	-2.884050	1.449473	-0.049105
N	-3.733419	-1.063588	0.011348
C	-4.230946	1.237343	-0.189398
C	-2.457346	2.709065	-0.163989
C	-4.690423	-0.115333	-0.134271
C	-4.108489	-2.336677	0.086296
C	-5.168026	2.277793	-0.394148
C	-3.311254	3.802727	-0.386156
H	-1.396604	2.866584	-0.074772
C	-6.068823	-0.413831	-0.237928
C	-5.456113	-2.734611	0.009248
H	-3.307741	-3.054264	0.209677
C	-6.563465	1.952785	-0.501163
C	-4.671040	3.595633	-0.489063
H	-2.881489	4.795209	-0.465117
C	-6.437403	-1.775802	-0.158592
C	-6.999115	0.662920	-0.420079
H	-5.706953	-3.787659	0.077817
H	-7.269742	2.764286	-0.649294
H	-5.356230	4.423528	-0.647152
H	-7.484727	-2.055545	-0.229422
H	-8.057009	0.430840	-0.499088

TS5B-L4-exo

C	1.175961	-0.633638	-0.704127
C	1.669777	-1.874577	-1.363818
C	1.792651	-0.031383	0.360473
C	0.775015	-2.914622	-1.675865
C	3.023783	-2.049225	-1.717152
C	1.202925	1.218159	0.992861

C	2.997547	-0.564268	1.057920	H	5.990268	0.556258	2.269359
C	1.210344	-4.081045	-2.308294	C	-0.524551	0.705196	-2.408096
H	-0.275344	-2.814919	-1.411499	C	1.849515	0.456026	-3.203441
C	3.459526	-3.209617	-2.349785	C	-2.501183	3.889353	-0.810317
H	3.740207	-1.270451	-1.478159	C	3.402210	4.842512	1.043371
H	0.303315	0.916057	1.539292	H	6.208202	-1.847921	2.872662
H	1.892534	1.597417	1.745820	H	1.573188	-0.507125	-3.636044
C	0.790241	2.378946	0.034872	H	1.800271	1.210899	-4.001201
C	3.147114	-1.924065	1.386867	H	2.880095	0.406038	-2.847732
C	4.038604	0.321776	1.398940	H	-0.745768	0.119431	-3.305618
C	2.554437	-4.232605	-2.649257	H	-1.160322	1.584120	-2.297989
H	0.497073	-4.869052	-2.533256	H	-2.736745	4.320292	0.165291
H	4.508680	-3.316536	-2.610526	H	-2.616137	4.630913	-1.600129
C	1.333267	2.150675	-1.396494	H	-3.144852	3.023527	-0.991518
C	-0.734200	2.610545	0.079083	H	3.223515	4.889899	2.120385
C	1.396289	3.674086	0.613814	H	4.460579	4.697397	0.829527
C	4.287031	-2.379101	2.045289	H	3.031446	5.757562	0.575933
H	2.376842	-2.634750	1.107451	C	-5.017026	0.334464	-1.843140
C	5.191269	-0.140368	2.032280	C	-5.657878	-0.075574	-0.689467
C	0.879974	0.902626	-2.136440	C	-4.897283	-0.623035	0.367382
H	3.955800	1.374248	1.140565	C	-3.497849	-0.711599	0.181954
H	2.895668	-5.137426	-3.143692	C	-3.619215	0.213811	-1.934865
H	2.422966	2.142924	-1.328075	C	-5.467576	-1.091065	1.599590
H	1.050911	3.008181	-2.020202	C	-2.674815	-1.279211	1.209508
O	-1.499089	2.079794	0.857571	C	-3.262044	-1.739504	2.410518
O	-1.113941	3.496921	-0.851584	C	-4.683512	-1.625114	2.579525
O	0.758657	4.542256	1.163593	C	-2.397089	-2.293073	3.381756
O	2.735728	3.689424	0.478685	H	-2.804596	-2.657614	4.320486
C	5.316108	-1.490479	2.366873	C	-1.042306	-2.361672	3.120919
H	4.375880	-3.431465	2.299044	C	-0.551394	-1.876775	1.894702

H	-6.542257	-1.012829	1.734767
H	-5.570684	0.748291	-2.678916
H	-6.735984	0.011880	-0.588718
H	-3.092268	0.533114	-2.825973
H	-5.124819	-1.977104	3.507306
H	-0.349139	-2.779668	3.842742
H	0.505044	-1.902983	1.665919
N	-1.338706	-1.350372	0.963323
N	-2.872227	-0.283974	-0.951176
Ni	-0.862924	-0.406240	-0.839860

INT5B-L4-exo

C	-0.928307	1.430368	-0.757621
C	0.343634	2.153789	-1.091701
C	-1.355687	1.388238	0.525896
C	1.545317	1.766862	-0.474459
C	0.386797	3.249082	-1.969373
C	-2.586970	0.637182	0.994400
C	-0.667975	2.119551	1.631724
C	2.749779	2.407508	-0.755287
H	1.518159	0.968298	0.257744
C	1.590548	3.899073	-2.251270
H	-0.528627	3.619167	-2.417125
H	-2.363119	0.071148	1.903761
H	-3.335752	1.383808	1.293532
C	-3.239698	-0.302058	-0.043191
C	-0.153803	1.411280	2.729376
C	-0.524762	3.514264	1.606391
C	2.779718	3.473772	-1.656877
H	3.660550	2.083363	-0.258119
H	1.593973	4.747517	-2.929832
C	-3.103845	0.331222	-1.442161
C	-2.553788	-1.649963	-0.087576
C	-4.681206	-0.540288	0.427271
C	0.508916	2.074857	3.761580
H	-0.256168	0.329692	2.762765
C	0.129050	4.180721	2.642242
C	-1.644154	0.617920	-1.863130
H	-0.917174	4.071154	0.761095
H	3.714316	3.981874	-1.877172
H	-3.662023	1.271803	-1.414624
H	-3.587648	-0.307573	-2.188171
O	-1.405655	-1.858934	0.342150
O	-3.249200	-2.587465	-0.681192
O	-4.944377	-1.295541	1.338682
O	-5.567107	0.226492	-0.214770
C	0.651717	3.463733	3.721278
H	0.910857	1.508970	4.598039
H	0.230802	5.261695	2.606552
C	-0.830906	-0.691208	-2.142593
C	-1.758722	1.377576	-3.206829
C	-2.616448	-3.882832	-0.849887
C	-6.933429	0.120610	0.249438
H	1.162183	3.984112	4.526600
H	-0.777265	1.613869	-3.623278
H	-2.287866	0.749443	-3.932742
H	-2.322643	2.311540	-3.098773
H	-0.144504	-0.485130	-2.964059
H	-1.503954	-1.485016	-2.503641
H	-2.415247	-4.320914	0.129420

H	-3.337543	-4.478864	-1.405536
H	-1.683198	-3.763208	-1.405924
H	-6.994733	0.402725	1.303306
H	-7.503343	0.809168	-0.373133
H	-7.291401	-0.904494	0.128615
C	3.672022	-0.646950	-3.074237
C	4.680205	-0.742599	-2.137157
C	4.346938	-1.076659	-0.805692
C	2.981551	-1.286372	-0.512152
C	2.337291	-0.858308	-2.686519
C	5.312012	-1.208103	0.251272
C	2.592673	-1.639433	0.816663
C	3.554979	-1.767031	1.842218
C	4.934924	-1.536305	1.521605
C	3.076470	-2.117242	3.125940
H	3.775872	-2.227114	3.949883
C	1.721170	-2.317691	3.315770
C	0.838847	-2.163382	2.229544
H	6.358631	-1.039813	0.015438
H	3.884426	-0.399935	-4.108421
H	5.717276	-0.568559	-2.409145
H	1.541209	-0.768726	-3.411500
H	5.676228	-1.631085	2.309437
H	1.325395	-2.590139	4.288098
H	-0.231099	-2.303294	2.334149
N	1.264644	-1.830656	1.014890
N	1.980894	-1.157037	-1.438385
Ni	0.205580	-1.357326	-0.631111

TS6B-L4

C	-1.294892	1.346549	-1.068535
C	-0.036442	1.946436	-1.620663
C	-1.590122	1.478630	0.243209
C	1.202375	1.472276	-1.160082
C	-0.034994	2.983549	-2.566412
C	-2.768655	0.798475	0.912239
C	-0.780157	2.332651	1.160231
C	2.402614	1.991113	-1.641933
H	1.209249	0.709681	-0.389620
C	1.165116	3.509611	-3.049144
H	-0.975183	3.403438	-2.908554
H	-2.486652	0.451690	1.909941
H	-3.562537	1.542179	1.064556
C	-3.364001	-0.376109	0.112827
C	-0.209069	1.772781	2.313328
C	-0.566134	3.694337	0.900442
C	2.388128	3.010483	-2.596400
H	3.346491	1.608160	-1.262201
H	1.142558	4.318208	-3.774328
C	-3.477263	0.049629	-1.366009
C	-2.493226	-1.620545	0.227579
C	-4.707046	-0.738660	0.762179
C	0.580681	2.541424	3.167273
H	-0.371506	0.720907	2.525106
C	0.213012	4.468279	1.759757
C	-2.120921	0.436797	-2.003864
H	-1.006397	4.140972	0.014774
H	3.319500	3.423407	-2.973469
H	-4.148316	0.912273	-1.395907
H	-3.948224	-0.744098	-1.954508

O	-1.415650	-1.662658	0.832233
O	-3.001559	-2.658366	-0.409469
O	-4.803993	-1.545581	1.662434
O	-5.717063	-0.011549	0.273697
C	0.794328	3.894160	2.892944
H	1.026871	2.085625	4.047456
H	0.366342	5.521839	1.543950
C	-1.287621	-0.777342	-2.323964
C	-2.465688	1.129570	-3.357026
C	-2.275141	-3.904709	-0.349000
C	-7.002446	-0.228653	0.901649
H	1.402609	4.498294	3.560048
H	-1.569231	1.339559	-3.943133
H	-3.114010	0.474847	-3.949413
H	-2.999437	2.071257	-3.180847
H	-0.235122	-0.633593	-2.543768
H	-1.766576	-1.684241	-2.685932
H	-2.125231	-4.199104	0.691580
H	-2.897022	-4.625994	-0.876207
H	-1.304179	-3.795061	-0.845587
H	-6.946540	0.015506	1.965233
H	-7.691980	0.437042	0.384054
H	-7.304554	-1.272148	0.785816
C	3.406203	-1.884980	-2.999518
C	4.514597	-1.446219	-2.300316
C	4.401720	-1.183884	-0.915730
C	3.135790	-1.384278	-0.319786
C	2.185598	-2.051055	-2.321789
C	5.490739	-0.726245	-0.097650
C	2.960221	-1.138134	1.085048
C	4.051550	-0.704634	1.871056
C	5.323560	-0.496828	1.237439
C	3.812683	-0.508509	3.251152
H	4.621801	-0.175235	3.894868
C	2.552519	-0.751671	3.765422
C	1.524457	-1.168654	2.899313
H	6.458240	-0.569543	-0.565506
H	3.459176	-2.099075	-4.061332
H	5.468233	-1.302434	-2.800140
H	1.297094	-2.385858	-2.846506
H	6.156441	-0.155722	1.845261
H	2.341680	-0.622301	4.821437
H	0.519617	-1.359351	3.262313
N	1.717236	-1.345130	1.595633
N	2.042728	-1.803617	-1.020265
Ni	0.418241	-1.770661	0.118222

INT6B-L4

C	-1.184763	3.456936	-0.000061
C	-2.450446	2.894326	-0.000098
C	-2.579821	1.487838	-0.000111
C	-1.384652	0.734283	-0.000077
C	-0.052468	2.622237	-0.000026
C	-3.833172	0.784208	-0.000156
C	-1.432001	-0.696081	-0.000087
C	-2.675297	-1.367247	-0.000131
C	-3.878531	-0.580641	-0.000164
C	-2.642843	-2.779289	-0.000139
H	-3.572433	-3.341955	-0.000171
C	-1.417990	-3.426096	-0.000104

C	-0.232037	-2.670813	-0.000061
H	-4.752188	1.364106	-0.000180
H	-1.051387	4.533718	-0.000057
H	-3.339718	3.518867	-0.000125
H	0.955120	3.025787	0.000006
H	-4.834151	-1.097959	-0.000195
H	-1.357479	-4.509433	-0.000109
H	0.744261	-3.144871	-0.000031
N	-0.229244	-1.336683	-0.000051
N	-0.143057	1.292476	-0.000035
Ni	1.313731	-0.078680	0.000036
Br	3.588964	-0.016964	0.000221

INT7B

C	0.667866	-0.671327	-0.517691
C	2.070917	-1.145496	-0.295090
C	0.378206	0.640055	-0.405619
C	2.582160	-1.178898	1.012067
C	2.904312	-1.545898	-1.349502
C	-1.031758	1.190679	-0.479195
C	1.428663	1.669428	-0.154711
C	3.886844	-1.606298	1.258721
H	1.945402	-0.850732	1.828873
C	4.211302	-1.971152	-1.105290
H	2.531720	-1.504428	-2.368947
H	-1.136292	2.030434	0.212494
H	-1.218778	1.588254	-1.486660
C	-2.096741	0.127737	-0.157130
C	1.401301	2.405890	1.040938
C	2.442470	1.929120	-1.086702
C	4.705732	-2.005678	0.200143
H	4.266035	-1.620600	2.277258
H	4.845225	-2.269008	-1.936577
C	-1.797021	-1.121524	-1.009088
C	-2.069375	-0.183242	1.346959
C	-3.466976	0.741188	-0.452208
C	2.375531	3.369617	1.300254
H	0.625618	2.192230	1.772027
C	3.412883	2.898285	-0.829633
C	-0.414562	-1.752668	-0.710613
H	2.470636	1.359350	-2.010658
H	5.723665	-2.335458	0.390262
H	-1.836087	-0.813299	-2.058102
H	-2.578431	-1.874953	-0.868617
O	-1.350401	0.347801	2.164366
O	-2.971066	-1.137793	1.649016
O	-4.126585	1.358406	0.355980
O	-3.826736	0.583985	-1.742626
C	3.383166	3.620652	0.365000
H	2.350557	3.922602	2.235820
H	4.193292	3.087447	-1.562276
C	-0.476634	-2.613227	0.524665
C	-0.072043	-2.672006	-1.921364
C	-3.021017	-1.511684	3.034993
C	-5.065628	1.215113	-2.112804
H	4.140603	4.373566	0.566892
H	0.843922	-3.240329	-1.743950
H	-0.888650	-3.384084	-2.087503
H	0.057481	-2.076704	-2.834576
H	0.430696	-2.871851	1.058128

H	-1.382278	-3.167355	0.749820
H	-3.254131	-0.642756	3.655965
H	-3.808880	-2.262017	3.108661
H	-2.058690	-1.928729	3.346377
H	-5.008388	2.294705	-1.947894
H	-5.201818	0.992317	-3.171463
H	-5.891707	0.810217	-1.521852

TS6A-L4-exo

C	2.098737	1.007524	0.538423
C	0.982017	1.814363	-0.060208
C	3.354520	1.089986	0.033988
C	0.840065	1.890353	-1.463737
C	0.107089	2.616043	0.706577
C	4.476451	0.246446	0.602732
C	3.774006	2.044319	-1.030262
C	-0.070480	2.756946	-2.059898
H	1.480355	1.284493	-2.089293
C	-0.813925	3.482270	0.108296
H	0.179607	2.625001	1.786362
H	5.285988	0.162510	-0.124343
H	4.906342	0.742463	1.482472
C	4.000728	-1.156945	1.027788
C	4.403131	1.580059	-2.197229
C	3.582611	3.425423	-0.868738
C	-0.899068	3.569065	-1.278563
H	-0.125070	2.805139	-3.144100
H	-1.447013	4.101952	0.737667
C	2.837205	-1.030955	2.027674
C	3.586593	-2.048940	-0.156724
C	5.189345	-1.906769	1.660079
C	4.801948	2.474665	-3.190033
H	4.560198	0.513086	-2.326767
C	3.992607	4.319801	-1.856401
C	1.860562	0.187191	1.820474
H	3.105434	3.792159	0.035498
H	-1.600158	4.253819	-1.746726
H	3.271276	-0.934064	3.024012
H	2.295327	-1.977626	2.028496
O	2.776988	-2.949294	-0.061048
O	4.242382	-1.754487	-1.282132
O	5.707450	-2.891906	1.184334
O	5.604405	-1.306033	2.789989
C	4.598251	3.846914	-3.023066
H	5.275925	2.100797	-4.093454
H	3.842591	5.386337	-1.713623
C	0.394700	-0.254637	2.029733
C	2.157031	1.142577	3.030042
C	3.999723	-2.644792	-2.393950
C	6.748034	-1.915003	3.429207
H	4.916732	4.543365	-3.793459
H	1.579221	2.068301	2.995011
H	1.944107	0.640679	3.982025
H	3.216533	1.416008	3.025027
H	-0.168665	0.603295	2.387446
H	0.341630	-1.015879	2.809196
H	4.335478	-3.652125	-2.137358
H	2.935616	-2.663490	-2.635059
H	4.583948	-2.240329	-3.220033
H	6.944861	-1.306639	4.311562

H	6.519440	-2.946385	3.708880
H	7.605837	-1.908783	2.752255
C	-3.673456	1.515474	-2.475082
C	-4.734160	2.035772	-1.764834
C	-4.730203	1.935780	-0.354957
C	-3.620975	1.301399	0.249522
C	-2.619500	0.884612	-1.789071
C	-5.791502	2.428808	0.476802
C	-3.563038	1.181022	1.672120
C	-4.633434	1.651436	2.471012
C	-5.750613	2.286535	1.832917
C	-4.532489	1.455395	3.866227
H	-5.332275	1.795597	4.517884
C	-3.415289	0.826265	4.382197
C	-2.392615	0.417380	3.508065
H	-6.639265	2.911132	-0.000705
H	-3.636032	1.571480	-3.557429
H	-5.568767	2.513541	-2.269943
H	-1.791615	0.455359	-2.325806
H	-6.565442	2.650086	2.451750
H	-3.307835	0.650896	5.447095
H	-1.496274	-0.054997	3.895852
N	-2.451365	0.594173	2.189689
N	-2.587469	0.768770	-0.463554
Ni	-1.173528	-0.051083	0.636967
C	-0.147020	-1.876607	0.725598
C	-0.808506	-1.645726	-0.527749
C	0.140535	-1.570758	-1.710785
C	-2.164671	-2.225436	-0.784864
C	-2.622383	-2.531580	-2.080265
C	-3.057731	-2.436481	0.288238
C	-3.916923	-2.993886	-2.297654
H	-1.973812	-2.416249	-2.937427
C	-4.351249	-2.890984	0.081110
H	-2.752533	-2.210314	1.304492
C	-4.800569	-3.164802	-1.222432
H	-4.247261	-3.221790	-3.305796
H	-5.021324	-3.034719	0.922523
C	-6.146357	-3.600562	-1.445284
N	-7.245566	-3.937362	-1.620918
H	-0.648793	-2.500468	1.459076
H	0.918050	-2.053143	0.674978
F	1.313715	-0.992901	-1.392045
F	0.414356	-2.803677	-2.189276
F	-0.343736	-0.858893	-2.769924

INT6A-L4-exo

C	-2.192259	1.496774	0.599571
C	-1.636839	2.708902	1.289801
C	-2.199706	0.300971	1.227426
C	-0.284432	2.771540	1.667722
C	-2.456847	3.802086	1.611002
C	-2.750075	-0.950530	0.582256
C	-1.596465	0.070613	2.570506
C	0.240017	3.898103	2.301751
H	0.351066	1.905415	1.512371
C	-1.937772	4.928959	2.251488
H	-3.515209	3.762037	1.374512
H	-1.950996	-1.542397	0.126271
H	-3.204895	-1.577403	1.354107

C	-3.794887	-0.634336	-0.494846	H	-3.523326	2.920600	-2.328583
C	-0.563761	-0.876904	2.682061	H	-4.352949	2.803142	-0.771927
C	-2.009609	0.750689	3.723529	H	-0.715334	2.786218	-1.392724
C	-0.584323	4.988025	2.588285	H	-1.313004	1.797405	-2.716655
H	1.287724	3.914854	2.592178	H	-5.423165	-4.182672	-1.741390
H	-2.595511	5.759198	2.492877	H	-6.813454	-3.248994	-2.391243
C	-3.202986	0.364217	-1.512978	H	-5.238850	-3.262215	-3.258310
C	-4.085003	-1.928534	-1.269744	H	-6.871494	1.656962	0.942200
C	-5.076350	-0.138165	0.198041	H	-7.462641	1.826249	-0.747818
C	0.057933	-1.119448	3.906988	H	-7.692288	0.288053	0.153037
H	-0.239925	-1.420479	1.798226	C	-0.397569	-3.709437	-0.439124
C	-1.393739	0.503558	4.950785	C	0.148747	-4.274311	0.696651
C	-2.510950	1.631219	-0.906318	C	1.379369	-3.783888	1.192855
H	-2.810211	1.479398	3.650153	C	1.974790	-2.712339	0.494154
H	-0.181164	5.865724	3.085070	C	0.261395	-2.634016	-1.064586
H	-3.989585	0.679066	-2.201637	C	2.046043	-4.302210	2.355300
H	-2.483716	-0.202181	-2.110765	C	3.235226	-2.189864	0.917608
O	-3.209732	-2.690614	-1.641230	C	3.880748	-2.723125	2.054347
O	-5.383281	-2.104635	-1.524696	C	3.243025	-3.792262	2.770775
O	-5.418645	-0.489313	1.305207	C	5.128069	-2.158437	2.407800
O	-5.780544	0.710705	-0.569311	H	5.663627	-2.533506	3.275277
C	-0.355734	-0.426843	5.047430	C	5.651511	-1.134875	1.640099
H	0.860301	-1.851261	3.969862	C	4.934491	-0.670080	0.522165
H	-1.727136	1.038699	5.835440	H	1.576939	-5.116597	2.899149
C	-1.134673	1.803308	-1.635418	H	-1.346624	-4.033566	-0.849520
C	-3.369325	2.868753	-1.244667	H	-0.354285	-5.087373	1.212021
C	-5.730269	-3.284297	-2.282536	H	-0.163838	-2.171946	-1.943915
C	-7.036083	1.146665	-0.010327	H	3.737659	-4.194949	3.649526
H	0.121186	-0.614306	6.005356	H	6.606551	-0.681319	1.881492
H	-2.887789	3.798624	-0.933764	H	5.318137	0.134263	-0.092886

N	3.756791	-1.178932	0.165853
N	1.405105	-2.126147	-0.600561
Ni	2.416691	-0.573446	-1.158910
C	-0.138067	0.693597	-1.251071
C	1.093269	0.494304	-2.117525
C	0.781684	0.078429	-3.540032
C	2.254381	1.362706	-1.899039
C	2.267581	2.404288	-0.919203
C	3.510804	0.960283	-2.450677
C	3.442347	3.016572	-0.543689
H	1.337765	2.736263	-0.475057
C	4.703584	1.605115	-2.071783
H	3.547656	0.263371	-3.280612
C	4.678286	2.614299	-1.113554
H	3.428857	3.816544	0.189432
H	5.641401	1.315267	-2.534599
C	5.899201	3.229481	-0.683392
N	6.893653	3.702684	-0.310835
H	-0.675437	-0.252300	-1.236694
H	0.191943	0.837470	-0.222633
F	-0.334435	-0.696092	-3.586942
F	0.553673	1.142371	-4.337997
F	1.769069	-0.651231	-4.126016

TS6C-L4-exo

C	-1.516904	-0.993250	-0.068793
C	-1.993724	-2.051771	0.886227
C	-2.441713	-0.327140	-0.871421
C	-1.577918	-3.388995	0.836143
C	-2.927527	-1.676143	1.866550
C	-2.058580	0.967936	-1.534604
C	-3.811881	-0.825737	-1.089330
C	-2.082226	-4.328201	1.737273
H	-0.885603	-3.724514	0.068519
C	-3.428046	-2.611532	2.769044
H	-3.252330	-0.641865	1.914916
H	-1.267316	0.823888	-2.284103
H	-2.897024	1.403124	-2.076225
C	-1.521182	1.965159	-0.481322
C	-4.903808	0.065702	-1.164303
C	-4.069242	-2.199124	-1.288308
C	-3.006608	-3.942023	2.709002
H	-1.757083	-5.362637	1.673222
H	-4.147253	-2.300287	3.521099
C	-0.197899	1.415049	0.082464
C	-1.218740	3.307715	-1.155662
C	-2.550831	2.164279	0.633511
C	-6.197032	-0.401382	-1.385915
H	-4.744001	1.128376	-1.023826
C	-5.359065	-2.659781	-1.529561
C	-0.144607	-0.072780	0.496830
H	-3.248314	-2.905774	-1.271254
H	-3.397110	-4.672316	3.411618
H	0.079344	1.998073	0.962831
H	0.558024	1.607252	-0.688635
O	-1.161030	3.479591	-2.351337
O	-0.967096	4.253104	-0.232783
O	-2.435626	1.756000	1.769764
O	-3.625167	2.829317	0.179149
C	-6.432637	-1.765567	-1.569096

H	-7.022498	0.303940	-1.420767
H	-5.526733	-3.720576	-1.691931
C	0.042237	-1.178886	-0.617751
C	-0.027822	-0.351901	2.003269
C	-0.666629	5.566665	-0.753239
C	-4.684674	3.043325	1.138578
H	-7.440249	-2.127832	-1.750527
H	0.152170	-1.407568	2.208875
H	0.811470	0.215100	2.416190
H	-0.933927	-0.035434	2.523924
H	0.026947	-0.912295	-1.671844
H	0.253961	-2.216102	-0.377757
H	-1.506258	5.936269	-1.346970
H	-0.503913	6.195520	0.121562
H	0.227981	5.529987	-1.380191
H	-4.296297	3.558138	2.019925
H	-5.422863	3.655426	0.621728
H	-5.116127	2.083679	1.435597
C	4.505876	-2.901724	-2.372415
C	5.724234	-2.376258	-1.983542
C	5.754976	-1.315726	-1.049405
C	4.513980	-0.850472	-0.559082
C	3.321036	-2.371494	-1.830811
C	6.962018	-0.692507	-0.581929
C	4.471857	0.226070	0.388725
C	5.673857	0.827588	0.826389
C	6.922929	0.334762	0.315533
C	5.564149	1.892017	1.750153
H	6.461122	2.384294	2.115114
C	4.311282	2.292918	2.174557
C	3.171973	1.628529	1.684009
H	7.912647	-1.055551	-0.961470
H	4.447087	-3.714255	-3.088517
H	6.654122	-2.766512	-2.387288
H	2.349592	-2.760538	-2.118001
H	7.842235	0.799962	0.659139
H	4.190381	3.107688	2.880294
H	2.177635	1.923092	2.003120
N	3.239096	0.619018	0.819053
N	3.315767	-1.373452	-0.947436
Ni	1.790030	-0.503026	-0.053880

INT6C-L4-exo

C	-1.799377	1.450316	0.224018
C	-2.700590	2.628970	0.319502
C	-2.140891	0.211978	0.654726
C	-3.348771	2.950407	1.523886
C	-2.902698	3.464213	-0.792753
C	-1.107848	-0.901900	0.647078
C	-3.516647	-0.159627	1.091271
C	-4.181127	4.064410	1.609821
H	-3.197024	2.316628	2.391921
C	-3.746410	4.571895	-0.710781
H	-2.417593	3.229773	-1.737248
H	-0.164493	-0.565255	1.093567
H	-1.479504	-1.736222	1.245238
C	-0.754779	-1.480825	-0.793131
C	-4.626227	0.169026	0.292924
C	-3.739834	-0.877763	2.276932
C	-4.387463	4.876942	0.491838

H	-4.669610	4.299132	2.551461	Ni	1.932222	-0.777343	-0.567134
H	-3.904024	5.195470	-1.586503	N	2.976997	0.832478	-1.029827
C	0.239170	-0.564443	-1.606811	N	3.412929	-0.999437	0.836255
C	0.058004	-2.709129	-0.485135	C	3.980815	1.082004	-0.130791
C	-2.097778	-1.772497	-1.498671	C	2.883415	1.643342	-2.083214
C	-5.915417	-0.199729	0.673393	C	4.195238	0.107081	0.893936
H	-4.467369	0.713626	-0.632274	C	3.581140	-1.952847	1.746539
C	-5.029975	-1.246164	2.660055	C	4.828143	2.210078	-0.206472
H	-2.899923	-1.134162	2.918550	C	3.698796	2.775503	-2.256474
H	-5.040373	5.742534	0.558478	H	2.131417	1.389985	-2.814956
C	-0.298876	0.863109	-1.748570	C	5.200466	0.294304	1.868088
O	1.263133	-2.544650	-0.185291	C	4.545212	-1.851158	2.768062
O	-0.487289	-3.894553	-0.487407	H	2.931180	-2.816054	1.651132
O	-2.452551	-1.278897	-2.546197	C	5.830445	2.394444	0.807405
O	-2.848695	-2.601568	-0.764566	C	4.651620	3.081211	-1.304790
C	-6.123190	-0.909779	1.858880	H	3.556903	3.396742	-3.133847
H	-6.758564	0.065328	0.041271	C	5.354152	-0.730720	2.830253
H	-5.181532	-1.791695	3.587488	C	6.004474	1.482218	1.807858
C	-0.454023	1.675904	-0.443793	H	4.646977	-2.655615	3.488365
C	0.356914	-5.026637	-0.147029	H	6.459462	3.278035	0.754036
C	-4.158208	-2.909376	-1.290709	H	5.278039	3.963198	-1.402840
H	-7.127755	-1.196795	2.156251	H	6.110044	-0.633546	3.604438
H	0.365575	1.416969	0.244100	H	6.769960	1.632838	2.563237
H	-0.335851	2.741032	-0.660518	H	-1.270707	0.812675	-2.255845
H	0.690502	-4.937764	0.889276	H	0.344826	1.414548	-2.436700
H	-0.280246	-5.899353	-0.276001	C	0.550022	-1.165807	-2.986625
H	1.218144	-5.063294	-0.817334	H	0.889975	-2.207016	-2.918732
H	-4.066739	-3.391310	-2.267143	H	-0.327625	-1.140697	-3.642198
H	-4.607289	-3.580817	-0.560058	H	1.353673	-0.606091	-3.479275
H	-4.746327	-1.994892	-1.379206				

TS6D-L4-exo

C	-0.847489	0.852350	-0.434976
C	-0.161949	2.180552	-0.598916
C	-1.740941	0.698016	0.581066
C	0.681373	2.628842	0.431122
C	-0.346212	3.005752	-1.716988
C	-2.413642	-0.616500	0.893297
C	-2.131167	1.822558	1.477688
C	1.327472	3.860480	0.344649
H	0.818518	2.001309	1.305784
C	0.311083	4.235750	-1.812770
H	-1.024173	2.702931	-2.508386
H	-1.704264	-1.306168	1.368440
H	-3.221916	-0.459358	1.606803
C	-3.024372	-1.322234	-0.352462
C	-2.724540	2.987787	0.971449
C	-1.947736	1.704462	2.864208
C	1.150833	4.666252	-0.783516
H	1.969237	4.190129	1.156739
H	0.151260	4.863207	-2.685069
C	-2.143728	-1.257584	-1.589839
C	-3.310222	-2.787298	0.018948
C	-4.353021	-0.621803	-0.717574
C	-3.104231	4.018878	1.828606
H	-2.893815	3.075222	-0.096536
C	-2.320643	2.739654	3.721952
C	-0.399538	-0.236319	-1.365553
H	-1.495790	0.802696	3.271687
H	1.651960	5.627424	-0.854111
H	-2.375637	-0.400452	-2.212559
H	-2.168685	-2.165024	-2.184624
O	-2.694197	-3.742425	-0.403661
O	-4.299018	-2.850846	0.916475
O	-4.760731	0.399861	-0.222354
O	-4.964935	-1.303661	-1.700646
C	-2.898755	3.901132	3.205476
H	-3.567094	4.913411	1.421509
H	-2.160736	2.638429	4.791823
C	-0.289592	-1.746817	-0.962787
C	-0.122256	0.097492	-2.832144
C	-4.658918	-4.175448	1.371006
C	-6.232665	-0.763667	-2.140548
H	-3.193824	4.706358	3.872136
H	0.721920	0.784838	-2.912429
H	0.134307	-0.808810	-3.386820
H	-0.975583	0.572870	-3.334177
H	-0.116996	-2.467579	-1.760366
H	-0.714555	-2.186624	-0.069555
H	-3.804102	-4.650482	1.858702
H	-5.475436	-4.026019	2.076091
H	-4.979723	-4.787057	0.524437
H	-6.099119	0.253751	-2.516385
H	-6.572417	-1.430832	-2.931770
H	-6.941279	-0.751335	-1.308979
C	3.394852	-4.019092	1.434306
C	4.662136	-3.479792	1.553863
C	4.921927	-2.193017	1.029233
C	3.846182	-1.519238	0.406981
C	2.387147	-3.277307	0.792016
C	6.203616	-1.547797	1.087156

C	4.040457	-0.211832	-0.153565
C	5.317526	0.391339	-0.099975
C	6.393084	-0.310843	0.542868
C	5.455500	1.664865	-0.699629
H	6.418888	2.166840	-0.682233
C	4.360138	2.248664	-1.304235
C	3.123597	1.575103	-1.297241
H	7.025179	-2.069501	1.569200
H	3.164415	-5.005252	1.822566
H	5.459502	-4.033347	2.041656
H	1.384425	-3.673937	0.673860
H	7.368805	0.164511	0.584786
H	4.426812	3.222893	-1.775684
H	2.248109	2.026155	-1.746939
N	2.956876	0.382068	-0.731455
N	2.599381	-2.060683	0.290846
Ni	1.320258	-0.854442	-0.599032

INT6D-L4-exo

C	2.318176	0.982954	-0.551447
C	2.864385	2.292230	-0.102756
C	3.037436	-0.164503	-0.625720
C	2.588915	3.470644	-0.816557
C	3.620793	2.391073	1.078568
C	2.334659	-1.476829	-0.963732
C	4.518756	-0.217742	-0.492732
C	3.065383	4.705421	-0.373848
H	2.023176	3.417138	-1.743959
C	4.087995	3.624665	1.526962
H	3.846430	1.488566	1.638244
H	1.928229	-1.399188	-1.979781
H	3.066172	-2.284142	-0.999787
C	1.172634	-1.936397	0.005260
C	5.329532	0.697525	-1.187180
C	5.140514	-1.191279	0.308433
C	3.813263	4.787704	0.802297
H	2.857121	5.602364	-0.951076
H	4.669218	3.678256	2.443304
C	0.698883	-0.865729	1.013189
C	-0.013089	-2.386991	-0.812751
C	1.631808	-3.143057	0.851878
C	6.716627	0.655920	-1.067218
H	4.863481	1.443476	-1.823210
C	6.529995	-1.225988	0.432835
H	4.535556	-1.918705	0.839535
H	4.181376	5.748642	1.150183
H	1.585628	-0.573424	1.589154
H	0.012080	-1.335952	1.726752
C	0.042885	0.406617	0.428389
O	-1.141319	-1.874388	-0.761019
O	0.242731	-3.389090	-1.626085
O	2.777246	-3.422963	1.110138
O	0.565367	-3.816828	1.326460
C	7.323098	-0.303314	-0.251733
H	7.325293	1.369939	-1.615157
H	6.992380	-1.978600	1.065751
C	-0.011532	1.405900	1.593094
C	0.817149	0.929336	-0.800690
C	-0.834042	-3.877220	-2.462021
C	0.869500	-4.935399	2.192034

H	8.404951	-0.335408	-0.157737
H	-0.444991	2.370163	1.329216
H	-0.597180	0.997058	2.425441
H	0.999311	1.616127	1.968164
H	0.602771	0.289534	-1.666508
H	0.451665	1.923056	-1.069093
H	-1.249153	-3.059595	-3.055360
H	-0.378382	-4.632744	-3.099501
H	-1.613563	-4.318502	-1.836027
H	1.410965	-4.589030	3.075487
H	-0.095575	-5.358203	2.469327
H	1.479090	-5.668531	1.658552
Ni	-1.749849	-0.150804	-0.105609
N	-2.683436	1.573840	-0.120052
N	-3.662606	-0.887481	-0.116835
C	-4.039718	1.441798	0.014663
C	-2.205372	2.802833	-0.322281
C	-4.563458	0.111214	0.055667
C	-4.093008	-2.144400	-0.105761
C	-4.926922	2.540735	0.065104
C	-3.015334	3.951740	-0.320538
H	-1.140511	2.886135	-0.480827
C	-5.948117	-0.113193	0.222468
C	-5.449749	-2.473024	0.078699
H	-3.333251	-2.904094	-0.254141
C	-6.330426	2.291788	0.251269
C	-4.372560	3.829868	-0.096521
H	-2.553958	4.919799	-0.481502
C	-6.377996	-1.460184	0.240081
C	-6.820832	1.020992	0.338050
H	-5.751929	-3.514762	0.082438
H	-6.999919	3.144544	0.313900
H	-5.015805	4.704527	-0.063576
H	-7.432024	-1.687434	0.372497
H	-7.884166	0.848899	0.475873

INT6B-L4-exo-Ni(I)

C	2.319693	0.763723	0.156008
C	2.972811	2.077470	0.417829
C	3.022859	-0.306145	-0.275852
C	2.643588	3.209590	-0.344979
C	3.919262	2.214545	1.443764
C	2.336592	-1.616350	-0.615579
C	4.486094	-0.287579	-0.556054
C	3.248982	4.441059	-0.092893
H	1.917929	3.115179	-1.148069
C	4.525502	3.445177	1.697038
H	4.186260	1.341579	2.032152
H	2.057163	-1.572015	-1.676815
H	3.036431	-2.448599	-0.531405
C	1.070438	-1.950605	0.233814
C	5.319451	-1.252550	0.036865
C	5.065448	0.662304	-1.411791
C	4.192562	4.563590	0.929948
H	2.986373	5.304582	-0.698809
H	5.259798	3.529526	2.494016
C	0.625975	-0.758443	1.110683
C	-0.033675	-2.393540	-0.710856
C	1.373854	-3.106294	1.199345
C	6.693020	-1.253645	-0.204792

H	4.884360	-1.993257	0.704031
C	6.438199	0.657272	-1.657579
C	0.810586	0.620527	0.434498
H	4.432682	1.411551	-1.876553
H	4.664975	5.522407	1.126178
H	1.235959	-0.769528	2.021591
H	-0.415167	-0.904219	1.416263
O	-1.069674	-1.788436	-0.985641
O	0.243319	-3.557654	-1.290415
O	2.476081	-3.480171	1.529634
O	0.226967	-3.605582	1.703426
C	7.258074	-0.298836	-1.053889
H	7.322831	-2.000109	0.272525
H	6.867440	1.401535	-2.323440
C	-0.005426	0.727201	-0.881565
C	0.307642	1.665877	1.449139
C	-0.707758	-4.049243	-2.259330
C	0.380596	-4.644950	2.688156
H	8.328052	-0.301103	-1.244618
H	0.396452	2.682481	1.059209
H	-0.750160	1.482274	1.662916
H	0.875256	1.621759	2.386259
H	0.472266	0.131601	-1.673633
H	0.019657	1.760768	-1.241038
H	-0.873380	-3.302250	-3.039185
H	-0.256408	-4.951722	-2.669797
H	-1.657017	-4.279642	-1.769269
H	0.939977	-4.270503	3.549633
H	-0.632426	-4.924406	2.977468
H	0.912809	-5.498486	2.259570

C	-3.059208	4.052852	-0.263211
C	-4.424395	3.882840	0.054453
C	-4.937370	2.589146	0.121180
C	-4.057559	1.494078	-0.130116
C	-2.259471	2.946015	-0.493407
C	-6.311272	2.271984	0.426902
C	-4.523083	0.179774	-0.081919
C	-5.885105	-0.125872	0.218940
C	-6.763351	0.986383	0.473232
C	-6.262798	-1.467787	0.246454
H	-7.290824	-1.738793	0.472423
C	-5.297041	-2.460411	-0.020987
C	-3.989194	-2.080067	-0.303956
H	-6.991755	3.097576	0.622053
H	-2.619860	5.042418	-0.331614
H	-5.067552	4.738357	0.240742
H	-1.213443	3.071677	-0.732458
H	-7.805307	0.779173	0.705859
H	-5.558769	-3.513274	-0.007261
H	-3.220330	-2.817778	-0.510693
N	-3.582093	-0.801443	-0.339855
N	-2.704164	1.669312	-0.433398
Ni	-1.800755	0.008441	-0.666016

TS6C-L4-exo-Ni(I)

C	-1.469587	-1.026926	-0.157867
C	-1.906768	-2.071640	0.832896
C	-2.483318	-0.335923	-0.905321
C	-1.424278	-3.387620	0.823266
C	-2.847981	-1.718386	1.814691

C	-2.110597	0.976560	-1.541238	H	-7.050916	0.260475	-1.590347
C	-3.838166	-0.844602	-1.091576	H	-5.588378	-3.779998	-1.402957
C	-1.871859	-4.325553	1.755851	C	-0.028633	-1.159136	-0.689871
H	-0.715249	-3.703121	0.062757	C	0.046187	-0.339795	1.939252
C	-3.292262	-2.650124	2.749200	C	-0.863606	5.594222	-0.602660
H	-3.220632	-0.700186	1.836348	C	-4.807821	2.727073	1.172983
H	-1.316079	0.869374	-2.295887	H	-7.501248	-2.194482	-1.660140
H	-2.951198	1.422191	-2.071223	H	0.285623	-1.388565	2.124767
C	-1.579454	1.961721	-0.472124	H	0.864227	0.264142	2.345950
C	-4.943354	0.029872	-1.272167	H	-0.861861	-0.087148	2.494013
C	-4.127423	-2.233669	-1.158592	H	0.071510	-0.828678	-1.723494
C	-2.806733	-3.960320	2.725138	H	0.400017	-2.137001	-0.477111
H	-1.491602	-5.343239	1.719297	H	-1.696473	5.958221	-1.210837
H	-4.019053	-2.351908	3.500276	H	-0.742351	6.206938	0.291188
C	-0.222924	1.432512	0.048425	H	0.048065	5.606426	-1.206826
C	-1.329310	3.333986	-1.088632	H	-4.476260	3.204058	2.098455
C	-2.595862	2.082300	0.661907	H	-5.589819	3.313801	0.690259
C	-6.235122	-0.449101	-1.469778	H	-5.168347	1.717271	1.388766
H	-4.789518	1.101442	-1.226831	C	4.608856	-2.972666	-2.241612
C	-5.419414	-2.706675	-1.354426	C	5.825910	-2.400518	-1.861374
C	-0.135107	-0.049875	0.443785	C	5.819133	-1.301480	-0.987798
H	-3.318147	-2.948378	-1.074693	C	4.560823	-0.826187	-0.531999
H	-3.153594	-4.688253	3.453700	C	3.416817	-2.457730	-1.727833
H	0.064708	2.018438	0.924291	C	7.002501	-0.635352	-0.513367
H	0.504510	1.651888	-0.742403	C	4.477533	0.302738	0.319225
O	-1.257941	3.569612	-2.274144	C	5.657667	0.932574	0.796283
O	-1.133802	4.264908	-0.126547	C	6.925363	0.419176	0.348793
O	-2.440974	1.700259	1.802063	C	5.512046	2.027770	1.662700
O	-3.730029	2.666028	0.221859	H	6.390962	2.537524	2.047518
C	-6.492151	-1.822100	-1.506592	C	4.225573	2.446435	2.011524

C	3.117591	1.789410	1.473350
H	7.969631	-0.999130	-0.851215
H	4.576735	-3.827344	-2.909487
H	6.768489	-2.794614	-2.231184
H	2.458309	-2.899189	-1.987696
H	7.830976	0.902235	0.707137
H	4.072347	3.293349	2.672537
H	2.110974	2.121535	1.705062
N	3.209661	0.747367	0.631632
N	3.367902	-1.423437	-0.874532
Ni	1.839538	-0.462067	-0.111150

TS6D-L4-exo-Ni(I)

C	-0.780226	0.861361	-0.466377
C	-0.168516	2.222323	-0.640722
C	-1.596858	0.654943	0.605360
C	0.779157	2.668103	0.291761
C	-0.545315	3.083933	-1.679235
C	-2.266104	-0.668509	0.876570
C	-1.895456	1.718582	1.606407
C	1.343978	3.937238	0.184015
H	1.081097	1.999147	1.090095
C	0.023794	4.354589	-1.793547
H	-1.299843	2.768235	-2.393824
H	-1.542013	-1.405535	1.246463
H	-3.021092	-0.552736	1.654120
C	-2.971742	-1.264580	-0.378675
C	-2.559411	2.901377	1.249715
C	-1.552050	1.522419	2.953239
C	0.970877	4.784353	-0.862145
H	2.085315	4.258101	0.910102
H	-0.280762	5.009878	-2.605491
C	-2.144179	-1.150513	-1.656445
C	-3.310372	-2.737160	-0.094643
C	-4.290858	-0.504547	-0.615929
C	-2.849513	3.871079	2.207799
H	-2.846550	3.051895	0.214694
C	-1.840288	2.493099	3.913603
C	-0.389600	-0.171887	-1.466872
H	-1.036930	0.609631	3.244028
H	1.414173	5.772586	-0.950656
H	-2.413871	-0.266211	-2.226532
H	-2.239049	-2.025586	-2.291966
O	-2.837930	-3.695681	-0.664596
O	-4.191004	-2.821171	0.918139
O	-4.655988	0.486509	-0.031377
O	-4.984988	-1.097049	-1.611542
C	-2.488447	3.672924	3.542955
H	-3.362968	4.782253	1.911853
H	-1.553912	2.329096	4.949320
C	-0.291801	-1.730725	-1.146612
C	-0.157563	0.227492	-2.919177
C	-4.571630	-4.156420	1.302195
C	-6.243384	-0.477559	-1.938822
H	-2.713296	4.430807	4.288756
H	0.657191	0.950630	-2.980079
H	0.133020	-0.645696	-3.509219
H	-1.037790	0.683969	-3.398151
H	-0.144566	-2.383448	-2.004778
H	-0.719532	-2.236672	-0.290845

H	-3.695440	-4.719558	1.634489
H	-5.283845	-4.032147	2.117704
H	-5.032400	-4.676586	0.458213
H	-6.086930	0.556333	-2.258524
H	-6.660572	-1.074857	-2.749591
H	-6.906475	-0.484909	-1.069574
C	3.126089	-4.046359	1.409759
C	4.429550	-3.528494	1.583347
C	4.734049	-2.269344	1.065751
C	3.705350	-1.551740	0.384069
C	2.177990	-3.292838	0.732368
C	6.023546	-1.632466	1.164715
C	3.941678	-0.287515	-0.168856
C	5.231329	0.321292	-0.082919
C	6.258361	-0.404451	0.620214
C	5.417895	1.560811	-0.694631
H	6.387537	2.050051	-0.648663
C	4.338378	2.155242	-1.377850
C	3.108594	1.506715	-1.407645
H	6.818470	-2.161756	1.685401
H	2.855861	-5.022732	1.798421
H	5.190563	-4.100343	2.107687
H	1.169216	-3.667188	0.580050
H	7.242952	0.050838	0.701656
H	4.445422	3.113273	-1.875305
H	2.264055	1.964560	-1.910261
N	2.870259	0.322900	-0.816123
N	2.425231	-2.068989	0.224232
Ni	1.294286	-0.840720	-0.713849

TS8B

C	-0.699524	-0.890666	-0.667840
C	-2.000648	-1.226076	-0.002386
C	-0.317582	0.440958	-0.855920
C	-3.121201	-1.723876	-0.679068
C	-2.081447	-1.046857	1.387969
C	1.124847	0.735747	-1.196684
C	-1.248553	1.576534	-0.730983
C	-4.295661	-2.030806	0.011082
H	-3.076804	-1.855739	-1.755883
C	-3.254617	-1.350431	2.078057
H	-1.209298	-0.680675	1.921056
H	1.334079	0.557928	-2.261414
H	1.371140	1.782927	-1.018941
C	2.056967	-0.175986	-0.375550
C	-0.868408	2.738399	-0.026245
C	-2.534552	1.552721	-1.306324
C	-4.367131	-1.843887	1.392354
H	-5.156100	-2.410610	-0.533845
H	-3.298321	-1.204432	3.154304
C	1.809631	-1.642626	-0.775965
C	3.518942	0.172963	-0.650721
C	1.755002	0.019624	1.114892
C	-1.741497	3.814970	0.112986
H	0.111953	2.780553	0.439378
C	-3.404970	2.631762	-1.169741
C	0.305555	-2.030469	-0.858333
H	-2.840906	0.684466	-1.877982
H	-5.281550	-2.080305	1.929939
H	2.315028	-2.289912	-0.055566

H	2.276406	-1.811626	-1.753093
O	3.916616	0.833433	-1.583859
O	4.328962	-0.398378	0.266275
O	1.364413	-0.834711	1.880570
O	1.956140	1.305376	1.475103
C	-3.016575	3.768681	-0.456811
H	-1.426577	4.692437	0.672513
H	-4.388970	2.586959	-1.629651
C	-0.276295	-2.069912	-2.227966
C	0.039707	-3.286317	-0.024521
C	5.735391	-0.158972	0.082268
C	1.636653	1.621276	2.841770
H	-3.696718	4.609479	-0.350431
H	-0.994267	-3.628677	-0.115717
H	0.697831	-4.093232	-0.367938
H	0.248580	-3.083200	1.029636
H	0.127676	-1.451432	-3.020848
H	-1.091214	-2.743715	-2.462314
H	5.947708	0.913083	0.121569
H	6.230650	-0.681410	0.901053
H	6.066276	-0.551048	-0.883518
H	2.195509	0.976402	3.524687
H	1.919700	2.665817	2.973819
H	0.564934	1.489674	3.016559

INT8B

C	-0.780357	-0.996660	-0.878930
C	-2.045806	-1.256946	-0.098381
C	-0.305360	0.418565	-0.948663
C	-3.194325	-1.801070	-0.685461
C	-2.073607	-0.956223	1.271651
C	1.152324	0.629000	-1.263197
C	-1.172227	1.565998	-0.777929
C	-4.346624	-2.031013	0.069927
H	-3.196572	-2.030868	-1.747540
C	-3.221498	-1.185559	2.028221
H	-1.181958	-0.553394	1.740920
H	1.375168	0.353578	-2.305319
H	1.441705	1.674862	-1.174905
C	2.049414	-0.244342	-0.361607
C	-0.674826	2.785783	-0.242413
C	-2.545512	1.543971	-1.138890
C	-4.364909	-1.722686	1.430643
H	-5.229465	-2.448199	-0.407860
H	-3.222218	-0.948223	3.089033
C	1.793631	-1.730252	-0.682702
C	3.521442	0.074577	-0.616804
C	1.707749	0.047043	1.102840
C	-1.497971	3.891915	-0.065270
H	0.359424	2.845245	0.078748
C	-3.359707	2.657184	-0.968415
C	0.308198	-2.109924	-0.767506
H	-2.964264	0.649223	-1.580737
H	-5.259865	-1.901088	2.020819
H	2.277917	-2.337604	0.085819
H	2.286441	-1.957939	-1.636640
O	3.950090	0.690560	-1.566685
O	4.301456	-0.468102	0.342498
O	1.263933	-0.748598	1.901775
O	1.934752	1.346159	1.390576

C	-2.848374	3.840707	-0.425902
H	-1.083849	4.801271	0.363733
H	-4.403305	2.602735	-1.268179
C	-0.433803	-1.921826	-2.057170
C	-0.019536	-3.336710	0.068283
C	5.714865	-0.252416	0.184272
C	1.549552	1.766353	2.711656
H	-3.489294	4.707316	-0.289398
H	-1.056229	-3.659153	-0.053813
H	0.632016	-4.169284	-0.227587
H	0.152118	-3.121381	1.128012
H	0.083720	-1.468802	-2.899190
H	-1.158407	-2.680278	-2.341619
H	5.939403	0.817879	0.187956
H	6.183542	-0.748578	1.034435
H	6.064176	-0.684997	-0.757410
H	2.027219	1.139365	3.468558
H	1.881349	2.801315	2.797066
H	0.462976	1.704023	2.820283

TS9B

C	-0.992516	-0.985223	-0.964448
C	-2.270953	-1.146774	-0.210184
C	-0.350277	0.273726	-1.071978
C	-3.387848	-1.772390	-0.784432
C	-2.365672	-0.686172	1.114040
C	1.124588	0.290425	-1.417492
C	-1.025647	1.551077	-0.813469
C	-4.577512	-1.910218	-0.067009
H	-3.333585	-2.134217	-1.808211
C	-3.550134	-0.829715	1.832617
H	-1.493678	-0.228375	1.569759
H	1.327429	-0.214924	-2.370184
H	1.490029	1.308040	-1.551094
C	1.996330	-0.422022	-0.340075
C	-0.321698	2.692415	-0.356332
C	-2.407306	1.728054	-1.078213
C	-4.663011	-1.437949	1.244132
H	-5.436746	-2.385569	-0.533203
H	-3.604326	-0.471265	2.857285
C	1.742606	-1.958852	-0.347569
C	3.475578	-0.190516	-0.649265
C	1.672067	0.142515	1.045073
C	-0.963570	3.909483	-0.137166
H	0.738887	2.624121	-0.150049
C	-3.044070	2.944092	-0.859920
C	0.301698	-2.394657	-0.393194
H	-2.981998	0.901309	-1.476940
H	-5.587011	-1.548834	1.805412
H	2.230764	-2.384737	0.533224
H	2.262580	-2.356902	-1.233782
O	3.916417	0.289179	-1.669710
O	4.249592	-0.650566	0.358436
O	1.023970	-0.414895	1.903694
O	2.182303	1.384021	1.188562
C	-2.331748	4.047341	-0.378686
H	-0.386033	4.756557	0.225742
H	-4.104928	3.034679	-1.079984
C	-0.459177	-2.220842	-1.655087
C	-0.204979	-3.266836	0.715113

C	5.666243	-0.499360	0.165446
C	1.843505	2.055234	2.416451
H	-2.831233	4.997221	-0.208974
H	-1.267160	-3.499150	0.604507
H	0.346703	-4.221999	0.728941
H	-0.053968	-2.784215	1.687326
H	0.138600	-2.036715	-2.551247
H	-1.216313	-2.981378	-1.854298
H	5.923981	0.557251	0.050514
H	6.129962	-0.914378	1.060771
H	5.991570	-1.042747	-0.726169
H	2.123613	1.442321	3.276467
H	2.406783	2.988597	2.402650
H	0.768826	2.256383	2.443572

INT9B

C	-1.221371	-1.190262	-0.697001
C	-2.660581	-1.229055	-0.322453
C	-0.521359	-0.046228	-0.875884
C	-3.613953	-0.456842	-1.007782
C	-3.107183	-2.071141	0.710783
C	0.921787	-0.107079	-1.346257
C	-1.057212	1.304165	-0.544072
C	-4.962846	-0.512991	-0.662632
H	-3.286246	0.191118	-1.814554
C	-4.455897	-2.121002	1.063574
H	-2.388196	-2.676043	1.257453
H	1.048609	-0.788880	-2.192086
H	1.238771	0.878763	-1.688695
C	1.935904	-0.554510	-0.240607
C	-1.650252	1.547592	0.707089
C	-0.948520	2.374534	-1.446516
C	-5.390207	-1.342333	0.377598
H	-5.682703	0.090341	-1.209592
H	-4.776232	-2.768757	1.875560
C	1.875548	-2.037007	0.099426
C	3.341580	-0.236280	-0.783551
C	1.663663	0.295574	1.021253
C	-2.132963	2.813206	1.036964
H	-1.718991	0.734539	1.422234
C	-1.433308	3.641192	-1.118889
H	-0.493345	2.210352	-2.419898
H	-6.441947	-1.384375	0.647839
C	0.566688	-2.767718	0.232545
O	3.644128	-0.255322	-1.955620
O	4.217178	0.029369	0.207372
O	1.290700	-0.135633	2.090091
O	1.850257	1.602441	0.757186
C	-2.028457	3.866294	0.124377
H	-2.587742	2.977411	2.010541
H	-1.347428	4.452561	-1.837111
C	-0.517142	-2.527954	-0.842636
C	5.567303	0.284487	-0.214869
C	1.581134	2.506489	1.842593
H	-2.404509	4.853166	0.380642
H	-0.063839	-2.616044	-1.839405
H	-1.253622	-3.334115	-0.774048
H	5.600624	1.147820	-0.885004
H	6.126937	0.482769	0.699555
H	5.975226	-0.585966	-0.736909

H	2.207481	2.261314	2.704770
H	1.818239	3.498893	1.458474
H	0.527482	2.450267	2.123995
H	0.132663	-2.527565	1.219984
H	0.789790	-3.842655	0.255766
C	3.055630	-2.639642	0.809111
H	3.978352	-2.586186	0.216079
H	3.259790	-2.113284	1.754206
H	2.874814	-3.693231	1.044993

TS10B

C	-2.550852	1.423427	-1.289461
C	-3.383265	0.413346	-0.895079
C	-4.078025	-0.374984	-1.972831
C	-3.508635	-0.051301	0.494230
C	-3.071784	0.764061	1.565279
C	-4.031981	-1.325102	0.814819
C	-3.130428	0.324278	2.878722
H	-2.673309	1.753599	1.378284
C	-4.099567	-1.766861	2.129041
H	-4.372146	-1.989002	0.031337
C	-3.644899	-0.950288	3.177986
H	-2.781640	0.967433	3.680222
H	-4.497088	-2.752275	2.349945
C	-3.703771	-1.408991	4.532267
N	-3.748855	-1.782566	5.633691
H	-2.585504	1.799440	-2.302639
H	-2.080568	2.057699	-0.554394
F	-4.031404	0.232374	-3.178453
F	-3.500090	-1.604347	-2.144534
F	-5.380155	-0.604098	-1.692510
C	0.947724	-1.210182	-0.322736
C	1.489971	-2.527494	-0.740166
C	1.580420	-0.354689	0.518499
C	2.050916	-3.409893	0.200446
C	1.422131	-2.944058	-2.080927
C	0.861798	0.916006	0.932213
C	2.991748	-0.499751	0.969209
C	2.544538	-4.652605	-0.189766
H	2.094479	-3.111358	1.242820
C	1.923758	-4.184960	-2.474093
H	0.983861	-2.285806	-2.825488
H	-0.194588	0.728962	1.138774
H	1.280127	1.338869	1.847810
C	0.943321	2.012278	-0.172008
C	3.352434	-0.276045	2.308247
C	4.013148	-0.797511	0.048935
C	2.488176	-5.044796	-1.530050
H	2.969356	-5.319375	0.555886
H	1.870387	-4.480369	-3.518722
C	0.643345	1.462976	-1.596702
C	-0.016448	3.153022	0.194769
C	2.353109	2.625051	-0.053707
C	4.683878	-0.362760	2.718174
H	2.583623	-0.044750	3.040429
C	5.342631	-0.880893	0.455383
H	3.752635	-0.954728	-0.992335
H	2.873113	-6.014503	-1.833649
H	1.581247	1.023839	-1.963604
H	0.440018	2.309271	-2.260570

C	-0.413815	0.395304	-1.788949
O	-0.785702	3.156385	1.133527
O	0.077055	4.164170	-0.686441
O	2.650880	3.393895	0.835917
O	3.222574	2.162334	-0.963874
C	5.685318	-0.664468	1.793651
H	4.936845	-0.192943	3.761228
H	6.114397	-1.109485	-0.275133
C	-0.694622	0.117432	-3.244679
C	-0.426950	-0.819893	-0.869990
C	-0.755493	5.306214	-0.411583
C	4.594613	2.553573	-0.755720
H	6.722658	-0.727536	2.110941
H	-1.550968	-0.548803	-3.373085
H	-0.882493	1.037478	-3.810298
H	0.176916	-0.368311	-3.716789
H	-1.090531	-0.640125	-0.015820
H	-0.879633	-1.663486	-1.397302
H	-0.513927	5.721040	0.570242
H	-0.534670	6.024415	-1.201145
H	-1.811545	5.021939	-0.431279
H	4.953227	2.147444	0.193023
H	5.147351	2.120450	-1.589178
H	4.682105	3.642675	-0.749440

INT10B

C	1.773824	-1.174671	-1.300848
C	3.028123	-0.369670	-1.120083
C	3.686947	0.177950	-2.355613
C	3.575386	-0.040442	0.159474
C	3.121062	-0.687954	1.350069
C	4.583508	0.960155	0.317636
C	3.632504	-0.361635	2.592496
H	2.370508	-1.468493	1.305088
C	5.087597	1.289395	1.561410
H	4.950997	1.498648	-0.545221
C	4.622004	0.633788	2.719338
H	3.272346	-0.876880	3.477090
H	5.843223	2.062980	1.653049
C	5.145844	0.974946	4.003950
N	5.572966	1.253172	5.050864
H	1.868974	-1.816132	-2.183106
H	1.672128	-1.837609	-0.444574
F	3.223201	-0.402307	-3.483333
F	3.474811	1.522336	-2.491573
F	5.030246	0.005494	-2.355015
C	-1.005240	1.256215	0.033634
C	-1.383926	2.664372	-0.256226
C	-1.796578	0.383294	0.714284
C	-2.071004	3.430962	0.705609
C	-1.016117	3.300071	-1.455649
C	-1.201102	-0.942119	1.149732
C	-3.252918	0.566561	0.957015
C	-2.404666	4.760601	0.464079
H	-2.335779	2.974789	1.653363
C	-1.355095	4.631314	-1.701431
H	-0.464167	2.751732	-2.210092
H	-0.184712	-0.811029	1.526490
H	-1.767943	-1.386487	1.970642
C	-1.144375	-1.986344	-0.008462

C	-3.841644	0.232797	2.188140
C	-4.096957	0.999423	-0.082929
C	-2.054349	5.368025	-0.745059
H	-2.931528	5.327714	1.226968
H	-1.066301	5.092392	-2.642366
C	-0.769068	-1.339458	-1.368513
C	-0.167857	-3.105578	0.374769
C	-2.540512	-2.635417	-0.035660
C	-5.220550	0.341131	2.379348
H	-3.216085	-0.102595	3.010740
C	-5.472277	1.105449	0.104899
H	-3.659756	1.242342	-1.045841
H	-2.313203	6.406493	-0.932889
H	-1.652149	-0.790270	-1.696104
H	-0.617784	-2.140117	-2.100798
C	0.433506	-0.358373	-1.450326
O	0.568589	-3.104441	1.341012
O	-0.188750	-4.092531	-0.536461
O	-2.878500	-3.493323	0.752529
O	-3.365615	-2.077857	-0.934369
C	-6.042608	0.776548	1.338793
H	-5.650483	0.084060	3.343748
H	-6.102520	1.438219	-0.715780
C	0.354477	0.263850	-2.860581
C	0.384598	0.788097	-0.379944
C	0.685191	-5.204067	-0.268545
C	-4.750371	-2.458859	-0.820269
H	-7.116367	0.855904	1.485283
H	1.144597	1.000021	-3.025345
H	0.442175	-0.503596	-3.637484
H	-0.609134	0.766003	-2.997488
H	0.909542	0.457474	0.521586
H	0.976646	1.628698	-0.753070
H	0.429000	-5.663399	0.689478
H	0.526130	-5.904111	-1.088723
H	1.726792	-4.871159	-0.239854
H	-5.137127	-2.151654	0.154724
H	-5.263463	-1.922971	-1.618250
H	-4.859588	-3.539806	-0.936924

TS11B

C	1.764505	-0.868429	-1.401938
C	2.971166	0.002351	-1.162281
C	3.511936	0.697890	-2.305370
C	3.624498	0.119655	0.113178
C	3.069843	-0.481726	1.279066
C	4.865507	0.807359	0.259666
C	3.669581	-0.341171	2.517748
H	2.170312	-1.081481	1.222981
C	5.461195	0.948480	1.498861
H	5.381101	1.165488	-0.619697
C	4.864082	0.391962	2.647286
H	3.222793	-0.804114	3.391352
H	6.405650	1.475027	1.589112
C	5.479103	0.546580	3.928764
N	5.977569	0.674785	4.972866
H	1.890054	-1.397359	-2.353524
H	1.746702	-1.634089	-0.629520
F	3.016046	0.441751	-3.491223
F	3.840783	1.968533	-2.206510

F	5.267711	-0.059660	-2.477916	C	0.362852	-0.145924	-1.444217
C	-1.176836	1.247340	0.153549	O	0.721598	-3.019213	1.163966
C	-1.634192	2.651910	-0.018398	O	0.061799	-3.961492	-0.773248
C	-1.924298	0.277724	0.746828	O	-2.675394	-3.715890	0.471590
C	-2.371236	3.287790	0.999987	O	-3.301482	-2.165021	-1.042614
C	-1.299457	3.413590	-1.151926	C	-6.192562	0.383716	1.324601
C	-1.254872	-1.036367	1.098069	H	-5.788191	-0.427217	3.281936
C	-3.391798	0.358210	0.976943	H	-6.263462	1.187255	-0.677962
C	-2.784226	4.610680	0.873835	C	0.190658	0.596821	-2.786598
H	-2.612833	2.734745	1.901229	C	0.242210	0.893381	-0.276463
C	-1.717661	4.738264	-1.282371	C	1.039513	-5.000149	-0.569734
H	-0.713267	2.970461	-1.948249	C	-4.652514	-2.660417	-0.957133
H	-0.264207	-0.866390	1.523722	H	-7.270875	0.392318	1.457990
H	-1.814713	-1.576424	1.864631	H	0.901143	1.420792	-2.897744
C	-1.086399	-1.989771	-0.127034	H	0.326532	-0.079941	-3.637056
C	-3.976137	-0.095906	2.170669	H	-0.816951	1.019032	-2.855164
C	-4.244856	0.817138	-0.043498	H	0.777272	0.517779	0.599894
C	-2.465863	5.343145	-0.272725	H	0.786249	1.797303	-0.567231
H	-3.348540	5.074532	1.678488	H	0.826970	-5.541090	0.355581
H	-1.452711	5.297822	-2.175695	H	0.947754	-5.658334	-1.433462
C	-0.757113	-1.226131	-1.437595	H	2.044147	-4.571575	-0.513928
C	-0.012166	-3.035933	0.196040	H	-5.037438	-2.504059	0.053736
C	-2.419749	-2.753025	-0.219429	H	-5.221000	-2.070741	-1.675398
C	-5.361073	-0.078627	2.345442	H	-4.685397	-3.724195	-1.204238
H	-3.343531	-0.452570	2.978920				

C	-5.626056	0.831630	0.127414
H	-3.809697	1.154647	-0.978531
H	-2.786830	6.376545	-0.370951
H	-1.680791	-0.730503	-1.736504
H	-0.537783	-1.962044	-2.218889

INT11B

C	1.796504	-1.209784	-1.238899
C	3.055460	-0.389859	-1.084895
C	3.684898	0.105350	-2.308651
C	3.564299	-0.002129	0.174115

C	3.073437	-0.546432	1.418951	C	-3.837328	0.261340	2.190937
C	4.613034	0.978091	0.334757	C	-4.125929	0.993418	-0.084559
C	3.568940	-0.167529	2.648356	C	-2.123177	5.290253	-0.930622
H	2.301559	-1.309523	1.409585	H	-2.950760	5.324180	1.063398
C	5.097762	1.354316	1.565707	H	-1.171278	4.945198	-2.835894
H	5.022427	1.458454	-0.545136	C	-0.759254	-1.398404	-1.363445
C	4.596785	0.797510	2.770856	C	-0.158293	-3.124610	0.440295
H	3.163294	-0.625575	3.547942	C	-2.517521	-2.692987	-0.026359
H	5.879937	2.108736	1.621534	C	-5.210849	0.393216	2.407671
C	5.099427	1.191239	4.035623	H	-3.199524	-0.069168	3.006007
N	5.516932	1.516190	5.080171	C	-5.494998	1.126422	0.128919
H	1.883696	-1.916943	-2.075012	H	-3.703722	1.209764	-1.060155
H	1.684354	-1.829296	-0.346309	H	-2.393434	6.319482	-1.153771
F	3.244100	-0.538657	-3.435062	H	-1.645091	-0.860584	-1.705983
F	3.470086	1.464729	-2.592609	H	-0.595864	-2.213203	-2.077114
F	5.054505	0.004759	-2.350830	C	0.449994	-0.428406	-1.447101
C	-1.022669	1.216770	-0.020991	O	0.414776	-3.180263	1.506283
C	-1.418833	2.612327	-0.349236	O	-0.001192	-4.044605	-0.534085
C	-1.814458	0.361080	0.682208	O	-2.830264	-3.643075	0.662620
C	-2.088448	3.413044	0.596573	O	-3.392377	-2.039934	-0.816141
C	-1.081991	3.202419	-1.580743	C	-6.047987	0.826058	1.378330
C	-1.215539	-0.954318	1.143352	H	-5.624338	0.157974	3.385477
C	-3.264557	0.564567	0.943803	H	-6.135452	1.456948	-0.685547
C	-2.438987	4.729869	0.310156	C	0.396522	0.151978	-2.876971
H	-2.324773	2.992888	1.568366	C	0.372493	0.758260	-0.420267
C	-1.438853	4.519656	-1.871716	C	0.947577	-5.079794	-0.239958
H	-0.535343	2.626326	-2.317945	C	-4.764628	-2.432800	-0.669943
H	-0.200681	-0.805217	1.517891	H	-7.117818	0.924991	1.544584
H	-1.779280	-1.385444	1.974381	H	1.194703	0.879794	-3.036161
C	-1.135614	-2.025113	0.005597	H	0.515860	-0.639870	-3.625685

H	-0.568024	0.642498	-3.062537
H	0.909714	0.473914	0.489667
H	0.954338	1.591035	-0.820953
H	0.645883	-5.635293	0.652667
H	0.953717	-5.729453	-1.116398
H	1.938864	-4.647556	-0.074309
H	-5.097403	-2.248083	0.355309
H	-5.321903	-1.802685	-1.363145

TS12B

C	1.759115	-0.667551	-1.417104
C	2.936402	0.245613	-1.140286
C	3.494503	0.947951	-2.212064
C	3.630136	0.225375	0.120345
C	3.083622	-0.369465	1.295817
C	4.957211	0.746162	0.249802
C	3.750151	-0.359913	2.509176
H	2.123068	-0.871008	1.259759
C	5.616439	0.762713	1.460931
H	5.473468	1.056159	-0.650434
C	5.022567	0.231244	2.630862
H	3.286652	-0.817196	3.379676
H	6.625502	1.163991	1.516928
C	5.701059	0.259881	3.881188
N	6.257321	0.284876	4.908485
H	1.885185	-1.166646	-2.387857
H	1.778040	-1.470834	-0.675795
F	2.900219	0.869849	-3.408778
F	3.961411	2.195107	-2.048108
F	5.208519	0.246351	-2.718331
C	-1.283918	1.204254	0.236728
C	-1.786807	2.601272	0.140896
C	-2.018364	0.181628	0.755365
C	-2.566334	3.154152	1.176275
C	-1.448633	3.441211	-0.935406
C	-1.318558	-1.131080	1.050333
C	-3.490186	0.217216	0.967569
C	-3.019975	4.468753	1.118876
H	-2.807462	2.541478	2.038259
C	-1.907786	4.757192	-0.997297
H	-0.822881	3.064276	-1.734983
H	-0.353000	-0.949469	1.525765
H	-1.886724	-1.732528	1.764818
C	-1.050104	-2.009177	-0.218247
C	-4.083112	-0.312256	2.125933
C	-4.342799	0.713681	-0.036275
C	-2.700224	5.278144	0.025739
H	-3.616131	4.865946	1.936951
H	-1.635648	5.377854	-1.847521
C	-0.753813	-1.156179	-1.480644
C	0.081607	-2.995254	0.099297
C	-2.318157	-2.854733	-0.394734
C	-5.470476	-0.333808	2.282805
H	-3.452017	-0.699010	2.921459
C	-5.725999	0.692100	0.116736
H	-3.901619	1.108347	-0.945623
H	-3.051029	6.306209	-0.018898
H	-1.702417	-0.694047	-1.758530
H	-0.489660	-1.837702	-2.296236
C	0.322340	-0.035545	-1.418061

O	0.653907	-3.089341	1.163139
O	0.385904	-3.744433	-0.977447
O	-2.480888	-3.959756	0.079962
O	-3.292862	-2.175979	-1.032600
C	-6.300048	0.167206	1.278991
H	-5.900975	-0.742477	3.193875
H	-6.359886	1.079937	-0.677247
C	0.105349	0.781535	-2.709084
C	0.150463	0.923143	-0.189708
C	1.499053	-4.636445	-0.801120
C	-4.597188	-2.772451	-0.986709
H	-7.380475	0.146394	1.397610
H	0.803825	1.618213	-2.772947
H	0.261863	0.158854	-3.597245
H	-0.918163	1.174698	-2.751955
H	0.687693	0.506834	0.666282
H	0.680996	1.851584	-0.412233
H	1.294232	-5.348651	0.002923
H	1.614167	-5.150936	-1.755766
H	2.401780	-4.068509	-0.558643
H	-4.912626	-2.904321	0.052028
H	-5.255101	-2.064468	-1.490507
H	-4.596939	-3.742206	-1.492347

4

C	1.835711	-1.503384	-1.056639
C	3.690709	-0.030137	0.031986
C	3.455504	-0.500920	1.339382
C	4.406829	1.172976	-0.118037
C	3.921038	0.195094	2.448794
H	2.900202	-1.418777	1.504049
C	4.876659	1.874777	0.984616
H	4.586442	1.569971	-1.110241
C	4.637330	1.391546	2.281852
H	3.731583	-0.184310	3.447696
H	5.419900	2.804511	0.849256
C	5.113704	2.115727	3.422323
N	5.501571	2.704482	4.347905
H	1.852352	-2.372190	-1.723342
H	1.738052	-1.895757	-0.045379
C	-0.824131	1.223425	-0.187337
C	-1.147774	2.582448	-0.696056
C	-1.648998	0.502825	0.618586
C	-1.763328	3.531591	0.142529
C	-0.802505	2.986034	-1.997941
C	-1.118713	-0.776198	1.237160
C	-3.088312	0.804929	0.845941
C	-2.049796	4.816183	-0.311617
H	-2.011085	3.252966	1.161218
C	-1.095415	4.270814	-2.456958
H	-0.307637	2.288404	-2.664132
H	-0.096996	-0.645278	1.600660
H	-1.707620	-1.074931	2.107223
C	-1.109137	-1.973469	0.234831
C	-3.664298	0.712406	2.123858
C	-3.930764	1.113877	-0.237934
C	-1.723562	5.191806	-1.617769
H	-2.522015	5.529015	0.359209
H	-0.828137	4.551432	-3.472541
C	-0.698845	-1.542842	-1.199065

C	-0.187263	-3.071820	0.783527
C	-2.532732	-2.555067	0.282568
C	-5.029356	0.935164	2.315764
H	-3.038071	0.477423	2.980262
C	-5.292598	1.332882	-0.049260
H	-3.504073	1.171099	-1.233857
H	-1.946750	6.194329	-1.972574
H	-1.545530	-0.990666	-1.608637
H	-0.594566	-2.445119	-1.811376
C	0.566582	-0.661206	-1.389013
O	0.518377	-2.980005	1.766882
O	-0.222136	-4.161707	-0.003757
O	-2.906446	-3.312362	1.153381
O	-3.337677	-2.050045	-0.665415
C	-5.850207	1.245049	1.230240
H	-5.449221	0.865898	3.315734
H	-5.922708	1.566452	-0.903650
C	0.590647	-0.282719	-2.883833
C	0.546164	0.645079	-0.517006
C	0.594016	-5.263848	0.429997
C	-4.736447	-2.359428	-0.515750
H	-6.913485	1.413635	1.377788
H	1.444561	0.361918	-3.116748
H	0.662755	-1.174913	-3.515957
H	-0.323603	0.250543	-3.164587
H	1.057922	0.442399	0.429130
H	1.164392	1.400237	-1.010613
H	0.286919	-5.592822	1.426056
H	0.433620	-6.053027	-0.304699
H	1.647288	-4.969722	0.458805
H	-5.105405	-1.956591	0.430908
H	-5.231369	-1.870804	-1.354505
H	-4.892640	-3.440739	-0.540953
C	3.163222	-0.772049	-1.139846
C	3.852772	-0.834970	-2.282836
F	3.437259	-1.485897	-3.366227
F	5.045711	-0.297708	-2.513841

TS11C

C	-6.519460	-1.931166	-1.446051
C	-7.288923	-0.782689	-1.466193
C	-6.781521	0.408076	-0.895577
C	-5.492366	0.347082	-0.320772
C	-5.243884	-1.896387	-0.853827
C	-7.494908	1.654698	-0.871058
C	-4.912568	1.522146	0.268699
C	-5.632754	2.737478	0.274089
C	-6.944554	2.770923	-0.310306
C	-4.998981	3.856764	0.864015
H	-5.516269	4.811635	0.896080
C	-3.727357	3.722620	1.388869
C	-3.083163	2.471306	1.334278
H	-8.487103	1.692280	-1.310840
H	-6.879886	-2.857142	-1.880380
H	-8.277045	-0.783832	-1.917584
H	-4.605216	-2.771704	-0.831198
H	-7.493654	3.707781	-0.297987
H	-3.213832	4.563637	1.841994
H	-2.070536	2.343051	1.697649
N	-3.663183	1.400619	0.795985

N	-4.745795	-0.792184	-0.300847	H	5.434268	-1.548967	2.021071
Ni	-3.024326	-0.504092	0.656517	C	5.458081	-4.081042	-0.904136
C	0.000039	-0.376411	-1.441766	H	3.828534	-2.954811	-1.713921
C	-0.768442	-1.590352	-1.017770	H	1.808233	0.727901	1.319179
C	-1.142229	-2.602344	-2.066521	H	2.929568	2.041175	1.540870
C	-1.168291	-1.830846	0.331824	C	2.156312	1.892224	-0.470794
C	-1.051165	-0.795749	1.341849	C	5.497753	1.623958	1.944948
C	-1.771363	-3.064253	0.761766	C	6.163681	0.638188	-0.152848
C	-1.605744	-0.969179	2.614034	C	6.377405	-4.216477	0.136434
H	-0.502596	0.118913	1.148391	H	7.058249	-3.402004	2.015693
C	-2.247251	-3.246042	2.050937	H	5.454477	-4.792503	-1.725471
H	-1.843688	-3.888280	0.065272	C	2.183473	0.903085	-1.668494
C	-2.208547	-2.194028	2.990637	C	0.755600	2.475465	-0.258674
H	-1.531757	-0.164479	3.337640	C	3.081780	3.105691	-0.691348
H	-2.679816	-4.199433	2.335410	C	6.764660	2.195954	2.078194
C	-2.777481	-2.346281	4.289699	H	4.759389	1.784531	2.725990
N	-3.254080	-2.458785	5.345527	C	7.425822	1.210917	-0.023594
H	-0.338726	-0.060189	-2.433266	H	5.925879	0.040738	-1.026896
H	-0.256982	0.430940	-0.760161	H	7.092534	-5.034111	0.133608
F	-0.910509	-2.160375	-3.312318	H	3.234608	0.770182	-1.925440
F	-0.462153	-3.767434	-1.912899	H	1.716410	1.389586	-2.531751
F	-2.470379	-2.925329	-2.009590	C	1.570183	-0.514217	-1.499485
C	3.532677	-0.975358	0.179585	O	0.053888	2.306845	0.726147
C	4.522166	-2.084065	0.141002	O	0.351646	3.162119	-1.333494
C	3.809453	0.275626	0.638618	O	2.908726	4.160818	-0.118092
C	5.444652	-2.249294	1.192865	O	4.128899	2.826277	-1.471836
C	4.535924	-3.033646	-0.896248	C	7.733432	1.993557	1.093953
C	2.655737	1.236815	0.854975	H	6.992910	2.798136	2.953204
C	5.176574	0.829626	0.831739	H	8.170407	1.051364	-0.798778
C	6.360444	-3.297489	1.189496	C	1.962023	-1.298313	-2.768496

C	2.098358	-1.281834	-0.237282
C	-0.948769	3.769093	-1.256004
C	5.176202	3.821885	-1.494423
H	8.717811	2.441711	1.194597
H	1.580572	-2.323273	-2.744423
H	1.572622	-0.814776	-3.671122
H	3.051223	-1.346830	-2.862004
H	1.450819	-1.061916	0.617422
H	1.975762	-2.354642	-0.414202
H	-1.004932	4.435723	-0.391768
H	-1.064815	4.330077	-2.182783
H	-1.722476	2.999256	-1.172571
H	4.778797	4.782776	-1.828339
H	5.603600	3.921605	-0.493761
H	5.921017	3.438433	-2.190127

INT11C

C	4.465034	-1.816268	-3.258003
C	5.658690	-2.201452	-2.679785
C	5.968503	-1.755691	-1.374403
C	5.016511	-0.934749	-0.731573
C	3.578526	-0.988086	-2.545335
C	7.181917	-2.082625	-0.678307
C	5.265899	-0.449763	0.588236
C	6.471860	-0.769105	1.249316
C	7.425275	-1.607286	0.578071
C	6.658451	-0.227203	2.542068
H	7.570100	-0.443248	3.091758
C	5.676007	0.577466	3.086906
C	4.501053	0.830239	2.355102
H	7.909416	-2.715602	-1.177467
H	4.195942	-2.133493	-4.259465
H	6.359906	-2.835487	-3.214799
H	2.651852	-0.659133	-2.989393
H	8.349538	-1.855460	1.091092
H	5.789007	1.016261	4.072170
H	3.713381	1.451743	2.761081
N	4.292315	0.326699	1.140404
N	3.839004	-0.555091	-1.311275
Ni	2.773813	0.531318	-0.104704
C	0.252244	-0.298198	-1.211332
C	1.126119	0.954452	-1.122120
C	1.319339	1.715033	-2.415162
C	1.126223	1.776493	0.089110
C	1.123157	1.079642	1.338727
C	1.382269	3.186572	0.121263
C	1.294730	1.775634	2.551779
H	0.800119	0.044896	1.413923
C	1.573249	3.847329	1.312313
H	1.393331	3.750437	-0.801068
C	1.531955	3.144908	2.546624
H	1.222507	1.233838	3.489324
H	1.744867	4.918687	1.318354
C	1.746934	3.843804	3.779090
N	1.950141	4.399012	4.780177
H	0.659395	-0.947958	-1.992356
H	0.403935	-0.841233	-0.283445
F	1.309259	0.893645	-3.493971
F	0.348714	2.641208	-2.627463
F	2.500246	2.384665	-2.449027

C	-3.351627	0.887107	-0.207783
C	-4.323155	1.877055	-0.741904
C	-3.667182	-0.080278	0.695229
C	-5.301989	2.441257	0.099442
C	-4.264699	2.328547	-2.072484
C	-2.541125	-0.880923	1.324396
C	-5.050554	-0.521449	1.021010
C	-6.202221	3.389209	-0.378751
H	-5.348758	2.133254	1.138421
C	-5.171244	3.273486	-2.554939
H	-3.512149	1.933829	-2.745820
H	-1.695155	-0.238406	1.579555
H	-2.855895	-1.347011	2.260227
C	-2.018764	-2.009383	0.384505
C	-5.439446	-0.806382	2.340377
C	-5.982664	-0.747824	-0.008686
C	-6.146870	3.806098	-1.711777
H	-6.945092	3.809723	0.293446
H	-5.111516	3.593919	-3.591596
C	-1.939816	-1.550389	-1.098221
C	-0.659180	-2.507584	0.890123
C	-2.993141	-3.188897	0.585511
C	-6.719577	-1.286165	2.624154
H	-4.742974	-0.638448	3.157461
C	-7.258066	-1.230029	0.272065
H	-5.691369	-0.548297	-1.034689
H	-6.850203	4.545158	-2.084808
H	-2.967800	-1.523166	-1.459332
H	-1.435377	-2.333514	-1.675530
C	-1.295562	-0.176221	-1.450204
O	0.025729	-1.972658	1.745106
O	-0.265655	-3.598917	0.218907
O	-2.942556	-3.901710	1.565381
O	-3.934170	-3.266707	-0.360655
C	-7.633658	-1.501727	1.591537
H	-7.000666	-1.490746	3.653458
H	-7.959478	-1.399882	-0.540341
C	-1.589522	0.029778	-2.951956
C	-1.893650	1.020102	-0.631695
C	0.989853	-4.172423	0.624517
C	-5.011743	-4.190362	-0.089524
H	-8.628194	-1.879490	1.811183
H	-1.215779	0.986920	-3.319741
H	-1.137070	-0.766280	-3.554924
H	-2.668815	0.002703	-3.131187
H	-1.303718	1.158956	0.278625
H	-1.745088	1.937414	-1.207707
H	0.968354	-4.416936	1.689079
H	1.106070	-5.073453	0.022765
H	1.808241	-3.470285	0.431654
H	-5.549344	-3.871043	0.806414
H	-5.660532	-4.139071	-0.962945
H	-4.618789	-5.199556	0.052290

TS12C

C	-7.406630	-1.308089	-1.671563
C	-7.976622	-0.205401	-1.058037
C	-7.149468	0.732466	-0.394830
C	-5.765812	0.470477	-0.411453
C	-6.010098	-1.489362	-1.630265

C	-7.603850	1.910568	0.290452	H	-2.650015	-4.027119	2.709968
C	-4.840684	1.341964	0.227859	C	-1.744989	-2.680634	4.851063
C	-5.305229	2.487441	0.905233	N	-1.894949	-2.965169	5.968615
C	-6.719911	2.747262	0.913365	H	-0.626524	0.475320	-2.007901
C	-4.332318	3.298432	1.536757	H	-0.270586	0.754342	-0.330152
H	-4.638101	4.190867	2.075358	F	-1.208217	-1.804055	-2.955361
C	-3.002046	2.933852	1.459776	F	-1.125199	-3.414831	-1.541197
C	-2.634776	1.770641	0.757815	F	-3.228645	-2.298623	-1.724612
H	-8.668196	2.124278	0.309791	C	3.412715	-1.049632	0.016766
H	-8.019446	-2.041348	-2.184030	C	4.308475	-2.211969	-0.221921
H	-9.052283	-0.055512	-1.079311	C	3.823124	0.123105	0.569076
H	-5.510340	-2.336872	-2.086678	C	5.271193	-2.575336	0.739234
H	-7.077570	3.631060	1.433025	C	4.192291	-3.014792	-1.370165
H	-2.212436	3.514369	1.922932	C	2.773820	1.149857	0.957339
H	-1.597913	1.490851	0.704771	C	5.243427	0.545826	0.704459
N	-3.520656	0.986546	0.148473	C	6.100539	-3.676590	0.545538
N	-5.220701	-0.615589	-1.014120	H	5.360997	-1.987059	1.646242
Ni	-3.278142	-0.697631	-0.835143	C	5.028029	-4.114467	-1.569219
C	-0.212779	0.003502	-1.110143	H	3.451645	-2.775834	-2.125451
C	-1.146891	-1.153717	-0.680677	H	1.904736	0.671255	1.411896
C	-1.306363	-2.136610	-1.699725	H	3.155709	1.853782	1.699232
C	-1.231849	-1.602358	0.751808	C	2.290466	1.974862	-0.270176
C	-0.720166	-0.800019	1.792643	C	5.709196	1.189774	1.862802
C	-1.943314	-2.767028	1.122586	C	6.140411	0.376623	-0.366577
C	-0.880323	-1.150763	3.128056	C	5.988002	-4.449362	-0.613627
H	-0.189204	0.119476	1.580953	H	6.832286	-3.936770	1.305294
C	-2.103407	-3.125189	2.454886	H	4.926174	-4.710076	-2.472417
H	-2.408869	-3.385012	0.365908	C	2.046964	1.080858	-1.517911
C	-1.570020	-2.321841	3.475761	C	1.034109	2.771114	0.091923
H	-0.467515	-0.516079	3.905327	C	3.391592	3.035058	-0.502321

C	7.029117	1.634385	1.955865
H	5.041275	1.331954	2.707972
C	7.456252	0.822583	-0.276712
H	5.791354	-0.103368	-1.274898
H	6.635596	-5.308247	-0.764818
H	3.035900	0.863763	-1.919989
H	1.537220	1.679535	-2.281662
C	1.311132	-0.288821	-1.378170
O	0.327329	2.587585	1.070124
O	0.763790	3.685040	-0.848497
O	3.521901	3.984580	0.240614
O	4.210100	2.748916	-1.518233
C	7.908019	1.453901	0.886533
H	7.368912	2.121756	2.865352
H	8.129832	0.682812	-1.117869
C	1.512481	-0.966205	-2.753318
C	1.926090	-1.224827	-0.276745
C	-0.341828	4.569999	-0.600199
C	5.380525	3.591094	-1.625286
H	8.934176	1.803277	0.956751
H	1.140736	-1.994243	-2.773829
H	1.019939	-0.407413	-3.556935
H	2.579626	-1.012855	-2.989678
H	1.394135	-1.083987	0.664428
H	1.724261	-2.263710	-0.556211
H	-0.218528	5.064996	0.366388
H	-0.319400	5.298367	-1.410128
H	-1.285673	4.016640	-0.611417
H	5.998987	3.469765	-0.733200
H	5.908924	3.236495	-2.509370
H	5.084314	4.636549	-1.735269

INT12C

C	5.762044	2.159445	-0.211689
C	6.266360	1.732236	1.004120
C	5.809219	0.511678	1.552173
C	4.843520	-0.193627	0.806749
C	4.786990	1.393965	-0.875998
C	6.248282	-0.052449	2.800104
C	4.342416	-1.436882	1.279680
C	4.763015	-1.971686	2.510516
C	5.745160	-1.237894	3.261305
C	4.159902	-3.191191	2.905813
H	4.442335	-3.651693	3.848214
C	3.203436	-3.775272	2.094096
C	2.849317	-3.174438	0.868917
H	6.991465	0.488307	3.378115
H	6.097742	3.086504	-0.662612
H	7.007339	2.322179	1.535802
H	4.358905	1.721682	-1.812606
H	6.083046	-1.643470	4.210133
H	2.709566	-4.695545	2.384955
H	2.085061	-3.570032	0.214522
N	3.426855	-2.041460	0.478726
N	4.321254	0.250385	-0.374095
C	0.139527	-0.388164	-1.353485
C	1.326505	0.492385	-1.708456
C	1.975657	0.273563	-2.894509
C	1.611950	1.707520	-0.894465
C	1.672278	1.603671	0.506406

C	1.804009	2.962802	-1.490508	C	-5.361114	4.820973	-0.077001
C	1.960654	2.710256	1.291854	H	-5.701775	4.570953	2.039004
H	1.523297	0.637554	0.978301	H	-4.825800	4.801293	-2.166498
C	2.083401	4.082891	-0.711660	C	-2.289985	-0.949040	-1.548705
H	1.738858	3.068114	-2.567958	C	-1.181012	-2.853585	-0.259081
C	2.179162	3.959576	0.683534	C	-3.622362	-2.732535	-0.247551
H	2.021350	2.619176	2.371174	C	-6.187980	-0.892581	3.220286
H	2.229962	5.051396	-1.178084	H	-4.048581	-0.784082	3.388989
C	2.518066	5.096055	1.488006	C	-7.122263	-0.186921	1.108967
N	2.818030	6.007212	2.144875	H	-5.706222	0.472301	-0.371924
H	0.214041	-1.293600	-1.950970	H	-5.962006	5.722727	-0.152918
H	0.256863	-0.706793	-0.316256	H	-3.283968	-0.525848	-1.694877
F	1.604579	-0.608706	-3.791095	H	-2.093649	-1.581386	-2.420004
F	2.922783	1.068054	-3.396269	C	-1.278273	0.231036	-1.539212
C	-2.973113	1.249181	0.229639	O	-0.296910	-2.894546	0.576149
C	-3.806288	2.475250	0.121077	O	-1.245293	-3.668390	-1.319916
C	-3.344012	0.139289	0.917183	O	-3.740071	-3.646044	0.540750
C	-4.448956	3.014063	1.250100	O	-4.571961	-2.349269	-1.108397
C	-3.944814	3.150521	-1.103751	C	-7.301120	-0.681156	2.404476
C	-2.366389	-1.017123	1.024144	H	-6.315744	-1.276695	4.228462
C	-4.712657	-0.107766	1.448230	H	-7.981579	-0.024207	0.463993
C	-5.217110	4.171876	1.152252	C	-1.364743	0.895834	-2.926992
H	-4.341421	2.515214	2.207754	C	-1.590188	1.290709	-0.414477
C	-4.721044	4.305292	-1.205265	C	-0.202375	-4.656072	-1.446400
H	-3.455178	2.760574	-1.991548	C	-5.827928	-3.053798	-0.993670
H	-1.348773	-0.666239	1.217838	H	-8.298877	-0.902074	2.772870
H	-2.622189	-1.688667	1.845784	H	-0.722529	1.782956	-2.986251
C	-2.352590	-1.853516	-0.287431	H	-1.062672	0.200712	-3.719350
C	-4.906449	-0.615598	2.743194	H	-2.388572	1.217533	-3.142411
C	-5.842436	0.095776	0.636463	H	-0.860518	1.169882	0.393321

H	-1.406145	2.293260	-0.809863
H	-0.128494	-5.245168	-0.528389
H	-0.502589	-5.288180	-2.281787
H	0.745051	-4.156608	-1.659533
H	-6.272919	-2.851573	-0.016681
H	-6.454347	-2.655939	-1.791623
H	-5.671354	-4.127868	-1.115613
Ni	2.986798	-1.008796	-1.058339
F	2.103479	-2.347641	-1.754535

INT1A-L9

C	1.726698	-1.641543	-2.365037
C	1.724711	-0.135426	-1.969107
C	0.395926	-1.299391	-0.620485
H	1.230380	-1.827338	-3.321718
H	2.709525	-2.110807	-2.356386
H	1.388588	0.491818	-2.800649
C	-1.823032	-0.884318	2.191158
C	-1.925764	-2.420648	1.956709
C	-0.506205	-1.505505	0.512794
H	-1.490877	-0.663661	3.210963
H	-1.490216	-3.002647	2.773601
H	-2.930764	-2.778045	1.736443
N	0.704773	-0.083746	-0.908691
N	-0.760793	-0.484564	1.254544
O	0.927745	-2.300485	-1.328318
O	-1.111485	-2.669851	0.764805
C	3.064957	0.444547	-1.446449
H	2.853988	1.477067	-1.152966
H	3.764637	0.490743	-2.287391
C	-3.102293	-0.050281	1.915548
H	-2.802325	0.997492	2.024723
H	-3.823912	-0.255892	2.712700
C	-3.698308	-0.258413	0.538090
C	-4.722539	-1.231180	0.282172
C	-3.206628	0.492662	-0.515991
C	-5.343292	-2.009976	1.301901
C	-5.167246	-1.430587	-1.071166
C	-3.650909	0.298995	-1.842840
H	-2.451582	1.251643	-0.330102
C	-6.317784	-2.939171	1.008445
H	-5.056228	-1.864596	2.338527
C	-6.171241	-2.400429	-1.339029
C	-4.603997	-0.652851	-2.117742
H	-3.229132	0.914208	-2.632563
C	-6.734323	-3.144436	-0.327842
H	-6.774677	-3.513728	1.809767
H	-6.491337	-2.541628	-2.368812
H	-4.950857	-0.816150	-3.135432
H	-7.501605	-3.880979	-0.549379
C	3.644504	-0.295652	-0.258865
C	4.595094	-1.361377	-0.401334
C	3.207300	0.052479	1.008590
C	5.160851	-1.750932	-1.650215
C	5.022719	-2.072618	0.773430
C	3.636436	-0.644556	2.160395
H	2.514018	0.881244	1.121923
C	6.064744	-2.787011	-1.741837
H	4.889459	-1.213205	-2.553000
C	5.953064	-3.139391	0.642773

C	4.516696	-1.694526	2.045167
H	3.263352	-0.338452	3.133953
C	6.462004	-3.497125	-0.584396
H	6.481163	-3.056289	-2.708811
H	6.260485	-3.669974	1.541071
H	4.848718	-2.242041	2.924217
H	7.172982	-4.314422	-0.667303
Ni	-0.003911	1.270769	0.455436
Br	-0.855137	3.671076	-1.478699
C	0.304423	3.089207	0.077408
C	-0.339599	2.964053	1.389604
C	1.662996	3.569594	0.004620
C	0.475170	3.237410	2.548634
H	-1.414872	3.120757	1.485033
C	2.394617	3.750398	1.150799
H	2.094635	3.770478	-0.971625
C	1.801353	3.571941	2.439957
H	0.000442	3.206247	3.527864
H	3.435665	4.055795	1.079126
H	2.395101	3.760373	3.330480

TS2A-L9

C	1.513786	-1.731237	-2.367046
C	1.750448	-0.228090	-2.046668
C	0.288522	-1.103669	-0.629411
H	0.941426	-1.879117	-3.288242
H	2.416999	-2.339380	-2.385501
H	1.536108	0.400423	-2.916687
C	-1.924377	-0.237419	2.074047
C	-2.008154	-1.788453	2.108613
C	-0.622683	-1.125583	0.505634
H	-1.627696	0.167182	3.046540
H	-1.503177	-2.214065	2.981170
H	-3.015801	-2.192513	2.023366
N	0.740530	0.043958	-1.007084
N	-0.837265	-0.004598	1.108681
O	0.691231	-2.215068	-1.260632
O	-1.265419	-2.221492	0.924178
C	3.170907	0.131141	-1.532152
H	3.167278	1.204696	-1.328778
H	3.874445	-0.032594	-2.355344
C	-3.198868	0.509485	1.596491
H	-2.898185	1.551426	1.436779
H	-3.924976	0.512277	2.415148
C	-3.784938	-0.048365	0.315825
C	-4.833268	-1.029233	0.316327
C	-3.250926	0.369363	-0.891478
C	-5.475606	-1.496413	1.499733
C	-5.273698	-1.574478	-0.939299
C	-3.691625	-0.167030	-2.122525
H	-2.478098	1.132084	-0.896121
C	-6.472449	-2.446842	1.453004
H	-5.184275	-1.093012	2.464231
C	-6.303546	-2.554090	-0.951590
C	-4.676430	-1.125866	-2.146874
H	-3.244942	0.191214	-3.045975
C	-6.891163	-2.987467	0.214549
H	-6.944573	-2.779583	2.373537
H	-6.621594	-2.957439	-1.910248
H	-5.019518	-1.548132	-3.088475

H	-7.677222	-3.736970	0.188291
C	3.580439	-0.604891	-0.273751
C	4.323364	-1.832383	-0.299343
C	3.180935	-0.084051	0.946044
C	4.846102	-2.413242	-1.491192
C	4.578766	-2.515526	0.940367
C	3.442276	-0.754639	2.161788
H	2.642651	0.858336	0.970293
C	5.546021	-3.599943	-1.469926
H	4.707698	-1.905901	-2.440510
C	5.299369	-3.740798	0.926156
C	4.115278	-1.953734	2.159280
H	3.105070	-0.310250	3.094352
C	5.770067	-4.279119	-0.249159
H	5.935160	-4.013928	-2.396302
H	5.476557	-4.247099	1.872330
H	4.314787	-2.482634	3.088377
H	6.320048	-5.216048	-0.243095
Ni	0.100607	1.548859	0.302237
Br	-0.990823	3.534842	-1.102032
C	0.921887	3.124723	0.004112
C	0.758018	3.273458	1.420276
C	2.140378	3.465771	-0.632312
C	1.922079	3.536309	2.189945
H	-0.219116	3.432068	1.866336
C	3.242681	3.726432	0.160425
H	2.207129	3.448325	-1.715467
C	3.146529	3.731422	1.575113
H	1.829833	3.648981	3.268276
H	4.205726	3.913180	-0.309455
H	4.029999	3.944680	2.170088

INT2A-L9

C	1.172685	-0.588820	-3.294292
C	1.522678	0.532116	-2.289310
C	0.233587	-1.006152	-1.336934
H	0.423526	-0.277583	-4.028613
H	2.030036	-1.041073	-3.789789
H	1.297151	1.525951	-2.678302
C	-1.330547	-1.649406	1.837015
C	-1.132127	-3.094842	1.302453
C	-0.436738	-1.627704	-0.196231
H	-0.808961	-1.488315	2.783518
H	-0.303236	-3.612441	1.793333
H	-2.031839	-3.708303	1.311961
N	0.602196	0.221481	-1.179195
N	-0.652669	-0.855093	0.805114
O	0.553522	-1.633219	-2.465418
O	-0.742135	-2.920938	-0.105744
C	2.998376	0.522029	-1.783464
H	3.078109	1.307760	-1.027673
H	3.622585	0.830292	-2.631017
C	-2.808936	-1.205901	2.011813
H	-2.785791	-0.196579	2.429698
H	-3.248053	-1.859215	2.776096
C	-3.622608	-1.290225	0.737799
C	-3.652707	-0.211801	-0.210386
C	-4.302282	-2.459395	0.441467
C	-3.073079	1.062554	0.046089
C	-4.313887	-0.411021	-1.470870

C	-4.973105	-2.644866	-0.789384
H	-4.324072	-3.262133	1.175545
C	-3.109594	2.067278	-0.897081
H	-2.590884	1.262822	0.994406
C	-4.318806	0.641317	-2.427476
C	-4.961480	-1.647264	-1.735022
H	-5.488894	-3.581340	-0.982990
C	-3.725955	1.853328	-2.152759
H	-2.654658	3.025696	-0.666600
H	-4.815325	0.472852	-3.380529
H	-5.460551	-1.784368	-2.691455
H	-3.744541	2.651747	-2.889981
C	3.450585	-0.835876	-1.284513
C	3.183560	-1.284659	0.054655
C	4.036429	-1.718894	-2.176251
C	2.718900	-0.423277	1.086005
C	3.391705	-2.669365	0.379597
C	4.300022	-3.065254	-1.834941
H	4.288174	-1.373619	-3.176970
C	2.388696	-0.904718	2.334676
H	2.615741	0.636440	0.892629
C	3.037529	-3.138851	1.674966
C	3.952066	-3.539643	-0.592283
H	4.755313	-3.724787	-2.568412
C	2.531875	-2.281472	2.628015
H	1.988516	-0.217902	3.074095
H	3.186598	-4.192029	1.902437
H	4.114228	-4.582800	-0.331854
H	2.266626	-2.654399	3.614035
Ni	0.102522	1.051753	0.529006
Br	-0.398242	1.708479	2.708806
C	0.830622	2.723091	0.033089
C	1.901618	3.327852	0.709992
C	0.337133	3.365825	-1.115333
C	2.479552	4.511786	0.239615
H	2.272362	2.879349	1.627762
C	0.899220	4.560197	-1.580625
H	-0.496206	2.924262	-1.658312
C	1.980692	5.133262	-0.908072
H	3.312835	4.956400	0.779943
H	0.492675	5.040776	-2.468697
H	2.424524	6.057870	-1.268902

INT3A-L9

C	-1.177279	-4.337980	-1.136579
C	-1.458312	-2.896904	-0.619976
C	0.680782	-3.117894	-1.206638
H	-1.252580	-5.109120	-0.368114
H	-1.779124	-4.616263	-2.002850
H	-1.802444	-2.906999	0.417059
C	3.808980	-1.307647	-1.004974
C	4.234446	-2.530891	-1.854399
C	2.073429	-2.665148	-1.363803
H	4.061837	-0.365649	-1.492288
H	4.420123	-2.295883	-2.904913
H	5.072519	-3.088070	-1.436945
N	-0.118945	-2.276009	-0.655747
N	2.336908	-1.464195	-0.965897
O	0.237418	-4.307383	-1.570907
O	3.048789	-3.419204	-1.825776

C	-2.459530	-2.103674	-1.485120	C	-4.868376	-1.354741	2.071066
H	-2.138015	-2.162435	-2.531991	H	-3.126480	-1.148012	0.877957
H	-2.391517	-1.054056	-1.189887	C	-6.697510	-2.639603	1.154247
C	4.366863	-1.325337	0.438818	C	-6.482955	-3.671331	-1.088495
H	4.029863	-2.251928	0.920385	H	-6.165164	-4.490400	-3.040719
H	3.905388	-0.495868	0.983416	C	-6.173465	-1.888066	2.182321
C	5.876633	-1.254025	0.462668	H	-4.451167	-0.762024	2.880397
C	6.560131	-0.028243	0.169695	H	-7.701704	-3.048963	1.229556
C	6.611489	-2.398266	0.716453	H	-7.488082	-4.072671	-0.988509
C	5.880849	1.197931	-0.070974	H	-6.760296	-1.700642	3.076719
C	7.995263	-0.031281	0.122035	Ni	0.820476	-0.478819	-0.265910
C	8.026183	-2.395080	0.683327	C	1.811306	1.107236	-0.056418
H	6.092644	-3.323787	0.958422	C	2.255636	1.781267	-1.200707
C	6.578368	2.350231	-0.362125	C	2.069286	1.655761	1.205706
H	4.800064	1.244351	-0.009494	C	2.927155	3.005411	-1.086949
C	8.681481	1.174995	-0.181624	H	2.068764	1.365087	-2.188908
C	8.702114	-1.236261	0.383556	C	2.768306	2.862228	1.320471
H	8.569477	-3.311488	0.893689	H	1.714646	1.154377	2.102660
C	7.991345	2.341411	-0.424540	C	3.184901	3.546158	0.174456
H	6.033427	3.273092	-0.536958	H	3.254580	3.526178	-1.982918
H	9.767914	1.160844	-0.215916	H	2.976908	3.272095	2.305383
H	9.788398	-1.224157	0.347723	H	3.713374	4.490872	0.266155
H	8.528642	3.256690	-0.655366	O	-2.823971	3.011140	-3.008398
C	-3.872592	-2.619978	-1.337162	C	-2.882297	3.395307	-1.860851
C	-4.621464	-2.357787	-0.141848	C	-2.441044	2.547321	-0.662351
C	-4.441139	-3.375588	-2.345212	O	-3.405665	4.562336	-1.469039
C	-4.115981	-1.583084	0.939370	C	-1.293980	1.614024	-1.158263
C	-5.946534	-2.896496	-0.024316	C	-3.661745	1.696139	-0.193136
C	-5.748396	-3.904858	-2.226771	C	-1.854090	3.392080	0.475178
H	-3.879541	-3.556677	-3.259411	C	-4.020350	5.351867	-2.509727

C	-0.857163	0.718017	-0.081012
H	-0.445909	2.218950	-1.492713
H	-1.652135	1.052287	-2.027013
H	-3.409609	1.277530	0.786135
H	-3.755411	0.859966	-0.892740
C	-4.998104	2.407495	-0.112051
O	-2.131407	3.268037	1.650312
O	-0.912197	4.223059	0.010433
H	-4.859525	4.800551	-2.941377
H	-4.365220	6.260417	-2.017456
H	-3.292153	5.581908	-3.290853
C	-0.746428	0.120383	0.996036
C	-5.200075	3.401001	1.003546
C	-5.956177	2.122613	-1.001353
C	-0.218860	5.008777	1.002441
C	-0.891058	-0.392571	2.327549
H	-6.234697	3.755457	1.034532
H	-4.950807	2.956556	1.974412
H	-4.543885	4.268941	0.880982
H	-6.932786	2.597650	-0.956756
H	-5.804472	1.399243	-1.798825
H	0.301916	4.350228	1.700943
H	0.495207	5.612187	0.443520
H	-0.927462	5.639510	1.544546
C	-0.366614	-1.641955	2.706850
C	-1.618681	0.379267	3.257319
C	-0.579436	-2.121001	3.996849
H	0.200966	-2.223329	1.986518
C	-1.828153	-0.117598	4.542129
H	-2.001964	1.350239	2.960159
C	-1.314284	-1.363720	4.913865
H	-0.173548	-3.085204	4.288086
H	-2.389926	0.475186	5.258026
H	-1.481102	-1.741787	5.918062

TS4A-L9

C	1.836004	1.532905	1.037402
C	-1.923689	1.607000	1.630185
H	2.041955	2.072059	1.967016
H	2.655764	0.839853	0.850809
C	1.818039	2.582566	-0.118782
C	-2.373104	2.365740	0.531815
C	-2.662043	1.606284	2.823989
C	-2.339825	-1.546464	2.412311
C	-0.074925	-1.452286	3.273166
C	3.252773	3.204137	-0.204398
C	1.494229	1.818007	-1.406682
C	0.833051	3.696027	0.261508
C	-3.546873	3.110206	0.633298
H	-1.786955	2.372282	-0.381282
C	-3.829713	2.361341	2.917516
H	-2.314697	1.018929	3.666697
C	-2.734105	-2.273712	3.536311
H	-3.077482	-1.262466	1.675429
C	-0.471319	-2.199545	4.387698
H	0.945218	-1.089780	3.197656
H	3.910803	2.412013	-0.582044
H	3.559893	3.434343	0.821122
Ni	-0.147445	-0.848381	0.587395
C	0.556948	0.802302	1.183218

C	-0.682699	0.871098	1.477106	N	-1.277826	-2.076144	-0.531286
C	3.393343	4.445053	-1.060642	N	1.306439	-1.567552	-0.625019
O	2.323602	1.184511	-2.030007	C	-2.700335	-2.197246	-0.925319
C	-1.003170	-1.169887	2.263246	C	-0.596721	-2.682070	-1.442800
O	0.191340	1.832624	-1.730291	C	2.786074	-1.615785	-0.713818
O	0.539952	3.947318	1.410811	C	0.854859	-2.459560	-1.441557
O	0.409802	4.399528	-0.794551	C	-2.645475	-3.362240	-1.939073
C	-4.277888	3.108075	1.823855	H	-3.312951	-2.450151	-0.060036
H	-3.894211	3.686694	-0.219241	C	-3.163456	-0.850034	-1.538760
H	-4.391359	2.367493	3.847134	O	-1.232893	-3.385460	-2.368645
C	-1.798933	-2.610902	4.519975	C	3.013537	-2.517000	-1.956183
H	-3.773998	-2.570775	3.642777	H	3.159579	-0.611388	-0.908160
H	0.253964	-2.431414	5.162655	C	3.378151	-2.174578	0.602617
C	3.162273	4.294769	-2.543789	O	1.690472	-3.117928	-2.222775
C	3.724652	5.613492	-0.500604	H	-2.855940	-4.336637	-1.490560
C	-0.169476	1.078498	-2.902061	H	-3.260102	-3.214143	-2.825724
C	-0.399210	5.553466	-0.481990	H	-2.582248	-0.684093	-2.453744
H	-5.195819	3.683210	1.898851	H	-2.886160	-0.049882	-0.844691
H	-2.109811	-3.170948	5.396961	C	-4.640990	-0.853249	-1.855047
H	3.444138	5.203305	-3.083722	H	3.287640	-1.951985	-2.849141
H	3.731597	3.451205	-2.951725	H	3.716476	-3.333437	-1.791949
H	2.102838	4.099542	-2.753073	H	2.852225	-3.105154	0.846421
H	3.859137	6.512084	-1.096998	H	3.142897	-1.467247	1.407267
H	3.881564	5.714647	0.570566	C	4.864419	-2.446517	0.497482
H	-0.005830	0.012504	-2.723713	C	-5.615718	-0.604351	-0.832667
H	-1.227013	1.284097	-3.063994	C	-5.065107	-1.184544	-3.129612
H	0.425758	1.398292	-3.760085	C	5.824008	-1.382026	0.442827
H	0.177389	6.259989	0.119936	C	5.301611	-3.754674	0.386620
H	-0.662776	5.989879	-1.444912	C	-5.276398	-0.174469	0.480227
H	-1.291645	5.247339	0.069789	C	-7.006443	-0.778469	-1.146709

C	-6.437294	-1.324335	-3.444460
H	-4.327732	-1.339812	-3.914892
C	5.476735	-0.010740	0.585010
C	7.208811	-1.704936	0.241451
C	6.668553	-4.071934	0.206447
H	4.577307	-4.564556	0.442714
C	-6.244750	0.020431	1.441496
H	-4.247015	0.056313	0.726694
C	-7.978882	-0.572441	-0.131235
C	-7.386350	-1.143454	-2.466574
H	-6.731591	-1.583972	-4.456932
C	6.425510	0.985494	0.509524
H	4.447182	0.261189	0.778641
C	8.163590	-0.655027	0.165318
C	7.601221	-3.065600	0.125679
H	6.971293	-5.111837	0.128689
C	-7.609764	-0.194350	1.140204
H	-5.953369	0.361320	2.430319
H	-9.027460	-0.714832	-0.379920
H	-8.442311	-1.267268	-2.692186
C	7.784892	0.662651	0.289070
H	6.129780	2.024431	0.626689
H	9.207792	-0.913869	0.009747
H	8.652752	-3.296745	-0.022964
H	-8.364174	-0.041631	1.906415
H	8.525906	1.454103	0.228586

INT4A-L9

C	-4.009112	-2.039098	-1.656420
C	-3.679769	-0.646258	-1.047086
C	-1.824761	-1.640624	-1.758901
H	-4.430933	-2.742387	-0.937828
H	-4.642632	-1.988990	-2.543993
H	-3.856678	-0.656715	0.029679
C	1.807341	-1.542310	-1.613694
C	1.557861	-2.622393	-2.702222
C	-0.380186	-1.860466	-1.922054
H	2.336398	-0.684586	-2.020715
H	1.853062	-2.312486	-3.706488
H	2.003476	-3.590078	-2.467588
N	-2.215445	-0.532238	-1.235832
N	0.428595	-1.099073	-1.272025
O	-2.706066	-2.577101	-2.070908
O	0.095487	-2.799288	-2.723468
C	-4.480477	0.502340	-1.688760
H	-4.261648	0.529017	-2.762623
H	-4.139100	1.457530	-1.275668
C	2.548238	-2.091750	-0.379499
H	1.975196	-2.940786	0.007160
H	2.528787	-1.325264	0.397749
C	3.959826	-2.544199	-0.686587
C	5.010404	-1.598729	-0.931561
C	4.247244	-3.896052	-0.730128
C	4.805263	-0.192658	-0.911402
C	6.333988	-2.082826	-1.204663
C	5.550075	-4.372682	-1.010989
H	3.457684	-4.616065	-0.524917
C	5.840508	0.688945	-1.134807
H	3.813756	0.202982	-0.745314
C	7.380774	-1.148415	-1.429877

C	6.572407	-3.483468	-1.241672	H	2.164821	1.969413	1.970045
H	5.735351	-5.442488	-1.033581	H	2.838128	1.137576	0.591367
C	7.145110	0.208366	-1.394231	C	1.708873	2.876230	0.072303
H	5.641145	1.756801	-1.113295	C	0.978432	-0.657771	4.546048
H	8.379579	-1.526982	-1.632118	C	2.935637	-0.363133	3.164926
H	7.577889	-3.839156	-1.451314	C	-1.862787	-0.673782	2.342886
H	7.957405	0.908256	-1.568476	C	-0.427285	-2.517115	1.748908
C	-5.965314	0.308752	-1.461710	C	0.302938	3.497727	0.258676
C	-6.537034	0.467502	-0.155046	C	2.750743	3.910830	0.541101
C	-6.770721	-0.097001	-2.509482	C	1.980141	2.598546	-1.406605
C	-5.796948	0.917982	0.975136	C	1.791819	-0.826451	5.664390
C	-7.927783	0.163062	0.032365	H	-0.100774	-0.717518	4.653951
C	-8.145646	-0.376421	-2.325888	C	3.749836	-0.541618	4.283647
H	-6.340955	-0.201077	-3.503278	H	3.402362	-0.216867	2.197146
C	-6.382739	1.033618	2.218034	C	-2.979379	-1.513860	2.271999
H	-4.755259	1.198733	0.862732	H	-1.985468	0.358166	2.649875
C	-8.500527	0.291858	1.326562	C	-1.538884	-3.352502	1.652594
C	-8.708742	-0.258506	-1.077786	H	0.568306	-2.915621	1.588283
H	-8.746985	-0.688141	-3.174564	H	0.310524	4.495097	-0.192187
C	-7.747569	0.709587	2.400482	H	0.159807	3.637931	1.333098
H	-5.796679	1.389018	3.061344	C	-0.864899	2.724419	-0.323321
H	-9.553925	0.055735	1.453608	O	2.499578	4.927982	1.141714
H	-9.761136	-0.481273	-0.922749	O	3.991631	3.489099	0.231848
H	-8.199031	0.803562	3.383563	O	2.136586	1.504372	-1.908284
Ni	-0.537843	0.362327	-0.217463	O	1.996348	3.743761	-2.105579
C	0.807720	0.615021	1.070547	C	3.182338	-0.767253	5.538581
C	0.633346	-0.243379	2.100825	H	1.339783	-1.008695	6.635351
C	1.928954	1.618635	0.955712	H	4.829929	-0.513269	4.169514
C	1.534145	-0.407800	3.277070	C	-2.821907	-2.850894	1.904444
C	-0.571797	-1.153500	2.060006	H	-3.966233	-1.120693	2.503672

H	-1.406174	-4.400916	1.399724
C	-1.165466	2.875566	-1.796762
C	-1.746592	2.097495	0.522838
C	5.070600	4.341950	0.675993
C	2.283317	3.625542	-3.515062
H	3.816744	-0.904772	6.409384
H	-3.685361	-3.508148	1.847664
H	-2.064659	2.323362	-2.077288
H	-1.310695	3.937203	-2.033322
H	-0.342501	2.514381	-2.418607
H	-2.719190	1.764911	0.180484
H	-1.600245	2.157584	1.594957
H	5.030212	4.465478	1.760742
H	5.985075	3.829867	0.378169
H	4.996777	5.321289	0.196935
H	1.492952	3.059741	-4.016446
H	2.320931	4.647702	-3.889380
H	3.239889	3.118704	-3.662206

TS4B-L9

C	1.360689	1.652019	1.426165
C	-1.989545	2.115218	-0.350115
H	1.129375	2.129886	2.382300
H	2.089370	0.857963	1.603738
C	2.000971	2.713991	0.478852
C	-2.517076	1.996837	-1.651952
C	-2.552200	3.049885	0.545352
C	-1.931795	-0.428083	2.387520
C	0.337045	-1.236887	2.715017
C	3.461234	2.972838	0.978983
C	2.015759	2.061500	-0.909970
C	1.155910	3.994809	0.553788
C	-3.597141	2.779929	-2.045041
H	-2.061315	1.291366	-2.340387
C	-3.640098	3.821862	0.146267
H	-2.114329	3.164200	1.530183
C	-2.307329	-0.996324	3.606239
H	-2.656742	0.126665	1.803492
C	-0.046770	-1.791238	3.940597
H	1.360607	-1.317404	2.377118
H	3.993075	2.020706	0.848968
H	3.402155	3.166221	2.055706
Ni	-0.373506	-0.516411	0.013594
C	-0.907149	1.279860	0.075155
C	0.125936	1.067528	0.839284
C	4.249862	4.074945	0.304993
O	2.684454	1.076295	-1.161100
C	-0.619217	-0.593403	1.928082
O	1.152185	2.605786	-1.772010
O	0.251697	4.157755	1.349253
O	1.561063	4.922246	-0.314908
C	-4.167149	3.684735	-1.142175
H	-4.000833	2.685030	-3.048594
H	-4.076540	4.535416	0.838794
C	-1.367070	-1.680541	4.382695
H	-3.331849	-0.886403	3.951240
H	0.692465	-2.306086	4.548169
C	4.475459	3.954080	-1.181851
C	4.753413	5.081972	1.025026
C	1.002811	1.915058	-3.027156

C	0.851691	6.176739	-0.269798	H	-3.756421	-1.748450	-2.681972
H	-5.018758	4.286441	-1.445397	H	-3.598364	-0.315475	-1.675562
H	-1.657658	-2.107745	5.337823	C	-5.422186	-1.432543	-1.344875
H	5.200714	4.691885	-1.536616	H	2.949725	-3.626139	-2.345608
H	4.836601	2.953967	-1.450985	H	2.956675	-4.418700	-0.748473
H	3.539160	4.117627	-1.729582	H	2.654734	-2.852942	1.393500
H	5.364114	5.857129	0.569753	H	3.149244	-1.188363	1.073304
H	4.581739	5.167083	2.095292	C	4.633784	-2.692356	0.598063
H	0.625813	0.902930	-2.847112	C	-5.960611	-0.656097	-0.265216
H	0.281463	2.501594	-3.594688	C	-6.242332	-2.315355	-2.021124
H	1.960787	1.859217	-3.548999	C	5.664071	-1.875409	0.026541
H	0.965868	6.638568	0.713949	C	4.949898	-3.957847	1.060384
H	1.308065	6.794118	-1.042581	C	-5.199029	0.310012	0.450922
H	-0.209941	6.014856	-0.473705	C	-7.332043	-0.849220	0.110997
N	-1.668836	-1.881491	-0.774368	C	-7.600413	-2.489048	-1.661463
N	0.992566	-2.019464	-0.473900	H	-5.839653	-2.889380	-2.852984
C	-3.117145	-2.146828	-0.656020	C	5.432402	-0.554652	-0.448843
C	-1.089277	-3.010923	-0.984250	C	6.996379	-2.405499	-0.061586
C	2.448236	-2.250205	-0.657367	C	6.262297	-4.477876	0.970540
C	0.383502	-3.026645	-0.995034	H	4.173459	-4.568415	1.516956
C	-3.206118	-3.689975	-0.843304	C	-5.748349	1.022853	1.494987
H	-3.415423	-1.852754	0.354666	H	-4.178163	0.520429	0.154890
C	-3.949452	-1.349720	-1.678717	C	-7.865937	-0.100880	1.194906
O	-1.815795	-4.114561	-1.086550	C	-8.129689	-1.779165	-0.609867
C	2.491019	-3.641179	-1.356458	H	-8.216374	-3.189486	-2.217571
H	2.829570	-1.458966	-1.307357	C	6.458027	0.195112	-0.984511
C	3.200292	-2.213597	0.690304	H	4.441877	-0.119498	-0.400170
O	1.077737	-4.014293	-1.537864	C	8.027283	-1.603817	-0.620939
H	-3.551870	-4.227819	0.040460	C	7.263499	-3.716755	0.416232
H	-3.796006	-3.989301	-1.711409	H	6.470696	-5.475537	1.345363

C	-7.092898	0.809758	1.880112
H	-5.146914	1.765753	2.011432
H	-8.905026	-0.258433	1.472832
H	-9.168141	-1.914732	-0.318967
C	7.767935	-0.330376	-1.074662
H	6.258928	1.201811	-1.340620
H	9.031326	-2.016152	-0.681411
H	8.276137	-4.104796	0.342213
H	-7.515980	1.375050	2.705416
H	8.565579	0.272865	-1.498397

INT4B-L9

C	-1.134736	-4.055049	-0.435708
C	-1.464223	-2.547377	-0.347096
C	0.710361	-2.862235	-0.715572
H	-1.135135	-4.558717	0.534297
H	-1.747562	-4.603550	-1.149404
H	-1.981501	-2.303959	0.580332
C	3.936051	-1.242141	-0.658749
C	4.261715	-2.535008	-1.444948
C	2.118105	-2.486049	-0.891621
H	4.264849	-0.355675	-1.203907
H	4.414167	-2.370699	-2.514787
H	5.089890	-3.108923	-1.030371
N	-0.107566	-1.945343	-0.319997
N	2.452722	-1.278697	-0.592044
O	0.259459	-4.085300	-0.925710
O	3.037617	-3.343394	-1.306250
C	-2.256361	-2.020006	-1.568723
H	-1.655702	-2.199940	-2.468747
H	-2.355749	-0.937496	-1.464627
C	4.539663	-1.239879	0.768115
H	4.202147	-2.149892	1.278723
H	4.120777	-0.400198	1.331834
C	6.050907	-1.190808	0.737168
C	6.743826	0.012102	0.375882
C	6.775687	-2.337241	1.007839
C	6.085259	1.245923	0.112096
C	8.176300	-0.019234	0.278921
C	8.187838	-2.361370	0.924654
H	6.250305	-3.243780	1.301101
C	6.792167	2.374976	-0.242201
H	5.006681	1.315785	0.208457
C	8.874522	1.161489	-0.091085
C	8.871718	-1.226594	0.559246
H	8.722507	-3.279346	1.149105
C	8.201600	2.333713	-0.351847
H	6.265084	3.305947	-0.431766
H	9.958539	1.123173	-0.161319
H	9.955966	-1.234848	0.484911
H	8.748298	3.228908	-0.632642
C	-3.601338	-2.697508	-1.708586
C	-4.684356	-2.375698	-0.827258
C	-3.775155	-3.684323	-2.662406
C	-4.606517	-1.344088	0.147696
C	-5.910828	-3.113929	-0.935061
C	-4.991192	-4.398425	-2.780047
H	-2.960117	-3.916227	-3.345078
C	-5.664129	-1.072690	0.989242
H	-3.708506	-0.741479	0.221844

C	-6.980924	-2.812666	-0.049934	C	-2.069351	4.024585	-0.788493
C	-6.033285	-4.125101	-1.925942	C	-5.117382	2.627443	1.522977
H	-5.091676	-5.162833	-3.544757	H	-4.345145	1.654277	-0.221101
C	-6.861478	-1.820652	0.897551	C	-3.567532	2.944419	3.345507
H	-5.580354	-0.267086	1.712680	H	-1.572074	2.217991	3.020229
H	-7.903637	-3.380555	-0.138274	H	-0.897108	-2.338663	5.736311
H	-6.967882	-4.674830	-2.001200	H	-0.014370	3.181855	0.767670
H	-7.688381	-1.600639	1.566413	H	0.738492	4.094562	-0.534600
Ni	0.726435	-0.230284	0.128654	C	1.359485	2.086699	-0.448008
C	-0.816548	0.345440	1.060646	O	0.057481	4.195219	-2.944508
C	-0.848118	-0.328655	2.374288	O	-1.272414	2.448902	-3.468449
C	-1.740165	1.169940	0.519544	O	-3.083473	4.143951	-1.440272
C	0.320620	-0.489067	3.149032	O	-1.766419	4.744995	0.294247
C	-2.034650	-0.927797	2.852476	C	-4.851613	3.093810	2.811472
C	-1.621336	1.566113	-0.945908	H	-6.106768	2.745654	1.090395
C	-2.825050	1.826011	1.310233	H	-3.343731	3.318750	4.340523
C	0.302699	-1.202961	4.347830	C	1.942518	1.869912	-1.820744
H	1.253157	-0.044056	2.816182	C	1.917982	1.523950	0.673851
C	-2.051125	-1.639317	4.048633	C	-1.103372	2.768599	-4.867293
H	-2.950960	-0.817996	2.284671	C	-2.788329	5.673588	0.724872
H	-0.993881	0.841106	-1.471599	H	-5.632466	3.578450	3.390544
H	-2.590530	1.556689	-1.450108	H	2.809222	1.207918	-1.790847
C	-0.993238	2.970869	-1.139763	H	1.202699	1.434076	-2.501931
C	-4.112400	2.008020	0.777053	H	2.231477	2.834059	-2.253704
C	-2.568213	2.322186	2.602362	H	2.887497	1.042400	0.627849
C	-0.882358	-1.782825	4.803425	H	1.579698	1.836539	1.657477
H	1.215945	-1.300879	4.928384	H	-0.044843	2.739232	-5.138560
H	-2.981028	-2.083161	4.393451	H	-1.666771	2.008695	-5.407774
C	0.279458	3.131032	-0.279909	H	-1.500377	3.765685	-5.071018
C	-0.667181	3.280944	-2.612135	H	-3.700150	5.125144	0.970024

H	-2.378471	6.161880	1.608181
H	-2.992783	6.399435	-0.065420

TS5A-L9-endo

C	-4.177355	-2.464638	-0.157656
C	-3.926956	-0.943407	-0.113950
C	-2.101041	-2.002587	-0.754546
H	-4.185215	-2.931549	0.832105
H	-5.063091	-2.757038	-0.719677
H	-4.235068	-0.522394	0.843142
C	1.432604	-1.763856	-1.483567
C	0.972899	-2.864536	-2.479148
C	-0.721341	-2.205623	-1.200110
H	1.762368	-0.869393	-2.013682
H	0.971890	-2.547945	-3.524237
H	1.526869	-3.799165	-2.377530
N	-2.440839	-0.857366	-0.270862
N	0.186183	-1.409403	-0.770044
O	-2.998720	-2.976816	-0.865848
O	-0.423494	-3.127580	-2.109354
C	-4.621498	-0.198009	-1.284999
H	-4.221519	-0.606859	-2.220513
H	-4.326362	0.853845	-1.257263
C	2.534082	-2.235492	-0.522404
H	2.240661	-3.198956	-0.090629
H	2.564005	-1.527484	0.304329
C	3.873243	-2.343872	-1.215430
C	4.595332	-1.160381	-1.588097
C	4.402448	-3.582382	-1.524954
C	4.135837	0.152139	-1.288272
C	5.845962	-1.293896	-2.278290
C	5.634592	-3.712794	-2.209849
H	3.866756	-4.480795	-1.226434
C	4.862308	1.271012	-1.635671
H	3.206131	0.276405	-0.747851
C	6.568904	-0.122462	-2.632932
C	6.338937	-2.592059	-2.581906
H	6.019246	-4.703094	-2.434806
C	6.092974	1.131988	-2.319949
H	4.489102	2.257454	-1.374575
H	7.516566	-0.235456	-3.153529
H	7.286520	-2.683797	-3.106368
H	6.663470	2.015871	-2.589864
C	-6.127074	-0.356830	-1.268069
C	-6.942922	0.339386	-0.316310
C	-6.724893	-1.229840	-2.159695
C	-6.423238	1.284664	0.611001
C	-8.356250	0.084059	-0.296541
C	-8.119302	-1.468843	-2.148367
H	-6.109169	-1.744901	-2.894157
C	-7.241421	1.924137	1.517165
H	-5.367684	1.528269	0.593584
C	-9.171182	0.757676	0.652662
C	-8.916424	-0.830932	-1.228227
H	-8.550585	-2.158822	-2.867460
C	-8.629686	1.654457	1.545598
H	-6.819813	2.646653	2.210622
H	-10.238850	0.553456	0.658077
H	-9.987764	-1.012554	-1.204304
H	-9.264863	2.162916	2.264874

Ni	-0.534697	0.241805	0.232012
C	1.039721	0.652036	1.338908
C	1.697006	-0.237633	2.154665
C	1.728999	1.956699	0.941439
C	3.063178	0.178576	2.643503
C	1.162767	-1.503070	2.709309
H	1.884818	2.595770	1.819936
H	2.738121	1.759896	0.574953
C	0.972976	2.829550	-0.096766
C	3.119789	1.091300	3.709423
C	4.265416	-0.290060	2.097653
C	-0.118425	-2.014361	2.405399
C	1.963481	-2.271815	3.582597
C	-0.473992	3.140733	0.377384
C	1.767078	4.134244	-0.292401
C	0.911482	2.107362	-1.442284
C	4.343828	1.537655	4.206361
H	2.193665	1.446311	4.156606
C	5.491940	0.163919	2.587826
H	4.259398	-1.012010	1.290544
C	-0.568843	-3.219327	2.933566
H	-0.774818	-1.465893	1.741738
C	1.515277	-3.483240	4.106739
H	2.949711	-1.916845	3.855890
H	-1.034852	3.576004	-0.452978
H	-0.407888	3.921023	1.149791
C	-1.235377	1.952877	0.956609
O	2.889790	4.324724	0.116377
O	1.045945	5.029728	-0.985355
O	0.062895	1.268204	-1.725430
O	1.893412	2.460865	-2.263847
C	5.535820	1.079611	3.639668
H	4.366590	2.241267	5.033710
H	6.412398	-0.203044	2.142908
C	0.247408	-3.968545	3.785971
H	-1.562140	-3.579011	2.675502
H	2.162543	-4.046855	4.772530
C	-2.731760	2.057029	0.811864
C	-0.669987	1.331074	2.113105
C	1.715188	6.277048	-1.272923
C	1.956485	1.822087	-3.559782
H	6.490867	1.430934	4.019111
H	-0.101614	-4.912745	4.193639
H	-3.240569	1.247754	1.336289
H	-3.076365	3.005073	1.250610
H	-3.028344	2.055758	-0.239754
H	-1.274229	0.618964	2.671888
H	-0.011328	1.904027	2.759727
H	2.610036	6.091484	-1.872505
H	0.991819	6.873651	-1.827704
H	2.001681	6.775847	-0.343893
H	2.311137	0.796254	-3.442672
H	0.974746	1.838690	-4.036752
H	2.682537	2.401925	-4.127237

INT5A-L9-endo

C	-0.541613	3.016572	-0.949617
C	-1.954650	3.282647	-1.348952
C	-0.192853	1.923675	-0.230605
C	-2.519098	4.552175	-1.138797

C	-2.748400	2.292447	-1.953049	C	2.305392	6.001921	-2.175842
C	1.236717	1.521107	0.046421	H	2.684819	6.273801	-0.070143
C	0.451874	4.045210	-1.376255	H	1.693386	5.523625	-4.188981
C	-3.846455	4.807683	-1.480471	C	-1.858701	-0.073550	2.614920
H	-1.909338	5.336775	-0.699827	C	-1.210066	0.958751	0.353108
C	-4.075285	2.547000	-2.299719	C	4.124577	-0.758993	3.152204
H	-2.313343	1.321068	-2.168633	C	2.546283	4.610894	3.341605
H	1.382749	0.510856	-0.365113	H	3.023966	6.754920	-2.486641
H	1.963805	2.168393	-0.442137	H	-2.613095	-0.700227	2.144678
C	1.536881	1.472771	1.591934	H	-1.482155	-0.610599	3.493039
C	1.191306	4.763273	-0.424956	H	-2.371000	0.833838	2.973667
C	0.631459	4.345234	-2.736028	H	-1.457104	0.196661	-0.393831
C	-4.631230	3.804807	-2.056607	H	-2.150698	1.493394	0.548037
H	-4.268841	5.792127	-1.299410	H	4.700019	-1.207202	2.340851
H	-4.672193	1.764835	-2.761527	H	4.790375	-0.357706	3.914253
C	0.207577	1.275496	2.372022	H	3.420488	-1.476689	3.576823
C	2.297501	0.226926	1.972857	H	2.606559	5.299822	2.495284
C	2.363064	2.712440	1.962314	H	1.975425	5.048260	4.159670
C	2.116015	5.730922	-0.820148	H	3.556266	4.346523	3.663395
H	1.020057	4.568306	0.629446	Ni	0.136371	-1.326386	0.969342
C	1.556944	5.309176	-3.132670	N	1.077119	-2.717688	-0.236654
H	0.041793	3.814775	-3.478776	N	-1.520889	-2.344674	0.266562
H	-5.664770	4.005873	-2.323672	C	2.401650	-2.930101	-0.856901
H	-0.292532	2.250374	2.433859	C	0.246402	-3.475891	-0.849664
H	0.418325	0.971953	3.403680	C	-3.019434	-2.417603	0.332988
C	-0.725697	0.286155	1.661606	C	-1.167498	-3.339619	-0.481841
O	1.824934	-0.906087	1.763650	C	2.119399	-4.045028	-1.906942
O	3.412877	0.397745	2.634934	H	2.677413	-1.993441	-1.350815
O	3.386852	3.013451	1.388462	C	3.481287	-3.289383	0.178842
O	1.819028	3.421121	2.959429	O	0.666663	-4.281662	-1.814855

C	-3.304474	-3.840038	-0.202649
H	-3.343751	-2.333465	1.369864
C	-3.667763	-1.299519	-0.527729
O	-2.069814	-4.205174	-0.908255
H	2.332030	-3.756189	-2.936499
H	2.617058	-4.989869	-1.676915
H	3.316590	-4.318455	0.519472
H	3.325765	-2.645613	1.047458
C	4.878600	-3.135223	-0.382466
H	-3.448051	-4.580805	0.588401
H	-4.124564	-3.889248	-0.917331
H	-3.172578	-1.301975	-1.506321
H	-3.451283	-0.333098	-0.071666
C	-5.158174	-1.476734	-0.718822
C	5.439520	-1.829185	-0.585824
C	5.621753	-4.250661	-0.717126
C	-6.083388	-1.191651	0.338653
C	-5.635904	-1.948912	-1.928649
C	4.749078	-0.625196	-0.267128
C	6.767336	-1.718369	-1.118375
C	6.925415	-4.138791	-1.257491
H	5.204102	-5.241141	-0.549464
C	-5.687738	-0.660639	1.597602
C	-7.481174	-1.440714	0.120575
C	-7.014923	-2.178415	-2.146447
H	-4.935054	-2.147261	-2.737146
C	5.333031	0.613655	-0.429563
H	3.737885	-0.680636	0.115661
C	7.339057	-0.428418	-1.290052
C	7.485924	-2.898600	-1.451898
H	7.480053	-5.038199	-1.507754
C	-6.610331	-0.411955	2.590716
H	-4.646920	-0.418176	1.778054
C	-8.405357	-1.177155	1.166977
C	-7.916688	-1.938173	-1.137241
H	-7.351460	-2.546855	-3.110892
C	6.645404	0.711889	-0.949158
H	4.789470	1.509968	-0.142182
H	8.346558	-0.357922	-1.692306
H	8.489657	-2.803543	-1.857899
C	-7.983122	-0.679645	2.379005
H	-6.282808	-0.000371	3.541309
H	-9.459893	-1.372949	0.990223
H	-8.977521	-2.119238	-1.289542
H	7.102078	1.688772	-1.077386
H	-8.700611	-0.481846	3.169834

TS5A-L9-exo

C	-3.738431	-3.494679	-0.041725
C	-3.506019	-1.985328	0.175398
C	-1.612969	-2.982565	-0.404842
H	-3.837744	-4.061950	0.888127
H	-4.561254	-3.725174	-0.717627
H	-3.907062	-1.636589	1.128378
C	1.863669	-2.258416	-1.100939
C	1.705238	-3.652418	-1.775915
C	-0.200097	-3.033125	-0.806170
H	2.041008	-1.481103	-1.849509
H	1.828461	-3.640944	-2.859440
H	2.349942	-4.418200	-1.339256

N	-2.026240	-1.907626	0.176400	C	-5.641624	0.645628	0.797676
N	0.530047	-2.016296	-0.515261	C	-7.706021	-0.189309	-0.225113
O	-2.487044	-3.940480	-0.684912	C	-7.649217	-1.821166	-2.023127
O	0.310171	-4.039588	-1.502674	H	-5.682223	-2.461067	-2.653083
C	-4.034300	-1.126863	-1.000705	C	-6.379400	1.457351	1.632875
H	-3.652009	-1.567238	-1.929377	H	-4.560940	0.659528	0.874426
H	-3.570979	-0.140462	-0.940199	C	-8.436210	0.657063	0.651945
C	2.972811	-2.200435	-0.037201	C	-8.368593	-1.017422	-1.171197
H	2.805551	-3.007677	0.685694	H	-8.157425	-2.446006	-2.751465
H	2.855563	-1.263455	0.508923	C	-7.791983	1.464000	1.562751
C	4.353385	-2.314424	-0.642662	H	-5.873088	2.097541	2.350271
C	4.923812	-1.220319	-1.375309	H	-9.521426	0.657699	0.588962
C	5.068120	-3.490564	-0.514496	H	-9.454529	-0.999666	-1.212831
C	4.267095	0.031014	-1.538951	H	-8.363567	2.106573	2.225841
C	6.225383	-1.380148	-1.958363	Ni	-0.624777	-0.540956	0.563985
C	6.349535	-3.647590	-1.094620	C	0.812661	0.810006	0.783129
H	4.643539	-4.313481	0.056584	C	1.868016	0.738982	1.671369
C	4.856325	1.064686	-2.232467	C	0.873519	1.522137	-0.561513
H	3.300472	0.193847	-1.079929	C	3.170634	1.437563	1.463704
C	6.803999	-0.295605	-2.671635	C	1.770180	-0.047545	2.924134
C	6.913907	-2.614080	-1.803821	H	1.843672	1.993620	-0.727038
H	6.881978	-4.586043	-0.971648	H	0.723460	0.809579	-1.378468
C	6.137708	0.901606	-2.808592	C	-0.254323	2.597929	-0.637247
H	4.339920	2.016282	-2.321980	C	4.374912	0.741229	1.684933
H	7.791110	-0.428141	-3.107366	C	3.244950	2.787514	1.071548
H	7.898144	-2.724254	-2.251772	C	2.349352	0.448737	4.111921
H	6.595038	1.722639	-3.353076	C	1.080853	-1.273626	2.990593
C	-5.542116	-1.053522	-1.050245	C	-0.655448	2.943336	0.812494
C	-6.271804	-0.202132	-0.156039	C	0.337885	3.838022	-1.318249
C	-6.235754	-1.832907	-1.958232	C	-1.465688	2.095630	-1.438238

C	5.607090	1.353025	1.469347
H	4.342322	-0.293665	2.010563
C	4.479848	3.402452	0.869900
H	2.341693	3.371933	0.933277
C	2.195988	-0.224446	5.319531
H	2.895617	1.385884	4.084021
C	0.931638	-1.951860	4.200964
H	0.702558	-1.722428	2.078134
H	-1.595177	3.507354	0.824684
H	0.116891	3.596961	1.222915
C	-0.805494	1.717084	1.698733
O	0.885403	4.744976	-0.729613
O	0.256262	3.737763	-2.652340
O	-1.567698	1.001115	-1.955699
O	-2.430480	3.026897	-1.451746
C	5.664347	2.684733	1.054595
H	6.521136	0.786194	1.618492
H	4.512120	4.447034	0.573849
C	1.480309	-1.425797	5.371156
H	2.633400	0.187722	6.224137
H	0.402773	-2.900808	4.224405
C	-0.503719	1.962194	3.154692
C	-1.825750	0.738146	1.408447
C	0.860844	4.815168	-3.401575
C	-3.645919	2.703814	-2.162590
H	6.624565	3.163104	0.885534
H	1.367755	-1.953925	6.313558
H	-0.487439	1.037022	3.733997
H	0.437817	2.494209	3.305415
H	-1.318227	2.588213	3.545660
H	-2.295452	0.259461	2.271430
H	-2.535040	0.994818	0.621176
H	1.927887	4.879287	-3.173594
H	0.701270	4.563898	-4.449470
H	0.380758	5.763857	-3.149885
H	-4.259395	2.024358	-1.563727
H	-4.163894	3.652897	-2.297014
H	-3.414161	2.242985	-3.124743

INT5A-L9-exo

C	-3.939813	-2.619877	-1.213536
C	-3.471764	-1.156604	-0.972078
C	-1.781790	-2.417957	-1.722813
H	-4.260048	-3.136767	-0.308343
H	-4.709559	-2.707737	-1.981752
H	-3.518327	-0.913439	0.091281
C	1.785370	-2.289945	-1.902709
C	1.554686	-3.710430	-2.462674
C	-0.380112	-2.716027	-2.045576
H	2.487729	-1.709891	-2.502689
H	1.717926	-3.788127	-3.541146
H	2.100255	-4.493698	-1.937231
N	-2.040068	-1.209066	-1.345289
N	0.425966	-1.715563	-1.986581
O	-2.741139	-3.324297	-1.708779
O	0.111265	-3.936259	-2.228520
C	-4.276860	-0.118199	-1.776336
H	-4.221613	-0.376454	-2.840443
H	-3.801625	0.858831	-1.660968
C	2.186303	-2.278629	-0.406468

H	1.572665	-3.031815	0.103671	H	-8.786071	-1.227190	-2.308658
H	1.884905	-1.314041	0.011757	C	-6.844553	1.421271	2.513999
C	3.647876	-2.543243	-0.145703	H	-4.773426	2.045404	2.698269
C	4.597534	-1.471953	-0.196308	H	-8.813705	0.728376	2.030376
C	4.080057	-3.817751	0.168662	H	-9.422919	-0.365219	-0.074685
C	4.228870	-0.129206	-0.480368	H	-7.129090	1.798520	3.491930
C	5.975894	-1.748122	0.084082	Ni	-0.512742	-0.019001	-1.302277
C	5.443202	-4.092935	0.431895	C	0.611290	1.658369	1.228736
H	3.355527	-4.626248	0.243198	C	0.613224	0.593637	2.066489
C	5.156682	0.891110	-0.481509	C	1.866067	2.405285	0.820899
H	3.187979	0.094883	-0.674796	C	1.866198	0.097462	2.721430
C	6.912761	-0.679241	0.065443	C	-0.633855	-0.165858	2.418736
C	6.371055	-3.078557	0.389693	H	2.067062	3.251861	1.488234
H	5.747446	-5.106131	0.677116	H	2.772113	1.798776	0.825432
C	6.516738	0.611122	-0.207865	C	1.542572	3.034187	-0.560922
H	4.842494	1.913813	-0.674164	C	2.792272	0.985888	3.296672
H	7.955333	-0.899062	0.281112	C	2.157388	-1.277401	2.782105
H	7.418511	-3.279789	0.598769	C	-1.450908	0.251565	3.479747
H	7.243013	1.418399	-0.207368	C	-0.985896	-1.338424	1.734329
C	-5.718781	-0.101614	-1.313049	C	0.048437	3.395924	-0.408114
C	-6.070683	0.437478	-0.030117	C	2.450162	4.233419	-0.848111
C	-6.698899	-0.681044	-2.097380	C	1.731755	2.003391	-1.644956
C	-5.136490	1.084759	0.826599	C	3.992079	0.527793	3.839926
C	-7.429434	0.321781	0.419600	H	2.564126	2.045530	3.335506
C	-8.042632	-0.771032	-1.661801	C	3.361852	-1.736832	3.311699
H	-6.435465	-1.073312	-3.077440	H	1.440478	-1.996983	2.403859
C	-5.510287	1.558077	2.065736	C	-2.615105	-0.447145	3.806056
H	-4.113208	1.229881	0.502546	H	-1.172144	1.135336	4.046105
C	-7.781992	0.823619	1.701363	C	-2.136945	-2.052799	2.071793
C	-8.396458	-0.289539	-0.424074	H	-0.357697	-1.683352	0.920120

H	-0.463212	3.488663	-1.369253
H	0.003374	4.379220	0.070721
C	-0.595823	2.314826	0.511826
O	3.514622	4.426656	-0.304240
O	1.908778	5.033861	-1.777787
O	1.053367	0.977671	-1.780583
O	2.741061	2.250744	-2.457539
C	4.291893	-0.835169	3.831909
H	4.690355	1.238863	4.272210
H	3.576235	-2.801500	3.310168
C	-2.966226	-1.600372	3.099925
H	-3.244954	-0.095189	4.617955
H	-2.382130	-2.965316	1.532322
C	-1.533771	3.037068	1.512235
C	-1.444331	1.333667	-0.316709
C	2.704374	6.181075	-2.149627
C	3.030875	1.308945	-3.520078
H	5.231987	-1.192774	4.241462
H	-3.869325	-2.145395	3.358316
H	-2.077993	2.322431	2.132918
H	-0.967542	3.705657	2.172163
H	-2.268781	3.640619	0.965609
H	-2.081975	0.757991	0.357752
H	-2.090557	1.901841	-1.000615
H	3.662915	5.854573	-2.561175
H	2.118024	6.710028	-2.900172
H	2.884449	6.814793	-1.277888
H	2.111137	1.026252	-4.035278
H	3.710672	1.837652	-4.186033
H	3.522196	0.433790	-3.090570

INT6A-L9

C	1.743455	-1.553701	-2.038657
C	1.769878	0.002487	-1.940412
C	0.326375	-0.854634	-0.476256
H	1.311081	-1.910211	-2.977280
H	2.704545	-2.034290	-1.862047
H	1.483870	0.460869	-2.892776
C	-2.026247	0.235440	2.024395
C	-2.127596	-1.309437	2.182375
C	-0.642358	-0.791193	0.613745
H	-1.749862	0.712531	2.969811
H	-1.710949	-1.663099	3.129535
H	-3.128347	-1.714026	2.037746
N	0.709556	0.275365	-0.959355
N	-0.911259	0.384625	1.075078
O	0.844957	-1.984870	-0.960057
O	-1.285970	-1.849579	1.111134
C	3.095578	0.661747	-1.475512
H	2.863896	1.723042	-1.329526
H	3.814885	0.604666	-2.298611
C	-3.280353	0.956975	1.464112
H	-2.982194	1.998461	1.297635
H	-4.046000	0.973370	2.245618
C	-3.797315	0.365440	0.169176
C	-4.836209	-0.624762	0.139390
C	-3.211062	0.762381	-1.021065
C	-5.534824	-1.068920	1.299693
C	-5.208893	-1.202172	-1.123817
C	-3.584025	0.195616	-2.260567

H	-2.436875	1.525694	-1.001886
C	-6.519530	-2.029948	1.224983
H	-5.299622	-0.637798	2.267428
C	-6.227821	-2.192511	-1.164871
C	-4.557338	-0.774188	-2.310429
H	-3.095464	0.535675	-3.169466
C	-6.869347	-2.604380	-0.019685
H	-7.036117	-2.344438	2.127773
H	-6.493921	-2.620765	-2.128428
H	-4.849268	-1.220639	-3.257964
H	-7.646450	-3.361991	-0.067426
C	3.653668	0.085186	-0.191422
C	4.602901	-0.991858	-0.182180
C	3.203671	0.595941	1.014475
C	5.172504	-1.548723	-1.364190
C	5.021424	-1.539342	1.079965
C	3.621364	0.057212	2.252595
H	2.516619	1.438499	1.007717
C	6.076759	-2.587155	-1.309679
H	4.901253	-1.142443	-2.333293
C	5.953411	-2.612494	1.100225
C	4.503845	-0.996617	2.285597
H	3.241273	0.488287	3.174782
C	6.469858	-3.131802	-0.064491
H	6.496450	-2.986356	-2.229151
H	6.255562	-3.016645	2.063658
H	4.828645	-1.421203	3.232601
H	7.182358	-3.951310	-0.032227
Ni	0.036301	1.863080	0.128174
Br	0.744217	4.012172	-0.143621

INT7A

C	-0.168162	-0.332121	-0.610939
C	-1.397558	0.143162	-0.324913
C	1.066341	0.514880	-0.826945
C	-1.720455	1.600639	-0.315638
C	-2.554460	-0.752300	-0.004680
H	1.282374	0.661094	-1.893242
H	1.015961	1.502640	-0.365886
C	2.200016	-0.356866	-0.248738
C	-2.508767	2.146633	0.713514
C	-1.286897	2.458313	-1.341068
C	-3.538479	-1.008643	-0.969818
C	-2.709636	-1.298693	1.278500
C	1.810956	-1.777616	-0.719923
C	3.567698	0.036459	-0.794910
C	2.191434	-0.256626	1.278425
C	-2.816949	3.505515	0.736490
H	-2.879777	1.495769	1.499753
C	-1.600120	3.817927	-1.322710
H	-0.716532	2.050027	-2.169552
C	-4.634953	-1.819440	-0.671148
H	-3.435727	-0.570020	-1.958955
C	-3.812043	-2.099027	1.582849
H	-1.952187	-1.097981	2.030152
H	2.223267	-2.541864	-0.059550
H	2.219974	-1.938246	-1.723784
C	0.237591	-1.823242	-0.775491
O	3.761143	0.679068	-1.803198
O	4.554706	-0.489020	-0.039230

O	1.903756	-1.143528	2.051962	H	2.172167	-4.681536	0.319490
O	2.514405	0.994330	1.666419	H	2.527819	-3.402086	1.511098
C	-2.361661	4.349102	-0.280501	H	1.854963	-3.083617	-1.440258
H	-3.418365	3.906649	1.548306	C	-3.534164	-1.546061	0.654916
H	-1.255323	4.459504	-2.129512	C	-3.379915	-2.200548	2.039262
C	-4.774174	-2.367131	0.606428	C	-1.504766	-2.402536	0.889388
H	-5.382412	-2.019727	-1.434453	H	-3.928610	-0.532739	0.718041
H	-3.915554	-2.517607	2.580534	H	-3.191202	-1.484307	2.844399
C	-0.232688	-2.299963	-2.169578	H	-4.184873	-2.883401	2.304766
C	-0.284974	-2.747502	0.280963	N	0.520370	-1.990747	-0.268071
C	5.891632	-0.214825	-0.492076	N	-2.117276	-1.517197	0.177424
C	2.509066	1.220654	3.085723	O	0.510364	-3.750709	1.152789
H	-2.607279	5.407604	-0.265473	O	-2.142519	-2.990164	1.891000
H	-5.629784	-2.994950	0.840668	C	3.032833	-1.612185	-0.387049
H	-1.326002	-2.334370	-2.210452	H	2.944511	-1.020228	0.530388
H	0.120785	-1.625923	-2.958624	H	2.944561	-0.918241	-1.227280
H	0.153503	-3.305257	-2.377481	C	-4.404037	-2.405605	-0.296589
H	0.105769	-2.682481	1.289178	H	-3.984436	-3.418966	-0.314606
H	-1.111417	-3.418155	0.075729	H	-4.313419	-2.010725	-1.306897
H	6.549986	-0.703666	0.226514	C	-5.842675	-2.455282	0.169055
H	6.045898	-0.620034	-1.496111	C	-6.716013	-1.330110	-0.001805
H	6.073173	0.863435	-0.512735	C	-6.308089	-3.583616	0.820430
H	3.233233	0.566325	3.579245	C	-6.339469	-0.148461	-0.700837
H	2.782578	2.267974	3.216278	C	-8.043083	-1.398510	0.544812
H	1.515218	1.026740	3.498930	C	-7.620853	-3.653923	1.343087
				H	-5.652072	-4.445284	0.927442
**TS6A-L9-exo**				C	-7.218553	0.904801	-0.838572
C	1.896873	-3.685744	0.664180	H	-5.359754	-0.075707	-1.159663
C	1.861031	-2.608545	-0.453785	C	-8.915778	-0.287421	0.395960
C	-0.103877	-2.723324	0.580937	C	-8.465720	-2.577349	1.216753

H	-7.951644	-4.559336	1.843167	C	3.169516	2.969137	-0.676158
C	-8.514698	0.844140	-0.277040	C	4.104198	2.465742	3.852182
H	-6.916093	1.787688	-1.391251	C	4.016385	0.056875	3.767342
H	-9.914729	-0.350674	0.819962	C	0.172599	1.442963	4.042005
H	-9.473795	-2.615450	1.621446	C	0.826930	-0.671624	3.089832
H	-9.190138	1.687313	-0.386716	C	1.644773	3.199477	-0.661921
C	4.371547	-2.316318	-0.402883	C	3.867501	4.266606	-1.115355
C	4.868607	-2.931687	-1.599753	C	3.587703	1.852940	-1.636985
C	5.114518	-2.393305	0.761214	C	5.264659	2.396559	4.623783
C	4.206483	-2.836480	-2.857449	H	3.676396	3.433523	3.607767
C	6.107724	-3.655430	-1.540570	C	5.183166	-0.013775	4.527900
C	6.341657	-3.095465	0.812383	H	3.521194	-0.860916	3.465846
H	4.759168	-1.886821	1.655741	C	-0.885477	0.815813	4.702618
C	4.723021	-3.443126	-3.982620	H	0.336589	2.509004	4.167901
H	3.296298	-2.251511	-2.947549	C	-0.216364	-1.307753	3.765933
C	6.605814	-4.276123	-2.717511	H	1.498828	-1.257552	2.469957
C	6.819634	-3.725283	-0.312225	H	1.228172	3.095060	-1.661733
H	6.898770	-3.133287	1.743737	H	1.463619	4.230373	-0.337824
C	5.928797	-4.179628	-3.912451	C	1.023807	2.254529	0.408035
H	4.204181	-3.350044	-4.932437	O	4.313610	5.102524	-0.364060
H	7.541399	-4.826410	-2.658776	O	3.844522	4.379361	-2.453770
H	7.756490	-4.275419	-0.281429	O	2.836721	1.200286	-2.340642
H	6.322612	-4.656969	-4.804788	O	4.912780	1.682342	-1.595273
C	2.219611	2.033856	1.390689	C	5.813105	1.156788	4.957138
C	2.220304	1.377374	2.574507	H	5.736790	3.312563	4.967051
C	3.485769	2.631345	0.794447	H	5.594830	-0.983561	4.793806
C	3.470605	1.298096	3.400260	C	-1.082587	-0.561403	4.568881
C	1.036254	0.711996	3.212616	H	-1.544450	1.401059	5.337737
H	3.739713	3.574058	1.289038	H	-0.347023	-2.380755	3.668507
H	4.356190	1.979921	0.904227	C	-0.199780	2.926107	1.043726

C	4.435484	5.583464	-2.990417
C	5.477080	0.718850	-2.511696
H	6.717735	1.102477	5.555599
H	-1.887192	-1.053331	5.108577
H	-0.720764	2.256726	1.730668
H	0.099076	3.819511	1.601605
H	-0.905467	3.232851	0.266008
H	5.488158	5.645332	-2.703638
H	4.331117	5.500480	-4.071750
H	3.907347	6.463072	-2.614058
H	5.112755	0.904181	-3.524653
H	6.555246	0.862980	-2.451768
H	5.213527	-0.294857	-2.205821
C	-2.386153	-0.280923	-3.091326
F	-1.619391	-1.399767	-3.143067
F	-2.376868	0.258593	-4.331791
F	-3.655548	-0.666117	-2.846395
Ni	-0.724744	-0.319228	-0.791671
C	-1.864332	0.715738	-2.068119
C	-0.507980	1.068704	-2.231262
H	0.127305	0.591211	-2.974082
H	-0.208416	2.064598	-1.951394
C	0.757620	0.818985	-0.045316
H	1.586198	0.421007	-0.610726
H	0.514532	0.213720	0.832640
C	-2.885847	1.686361	-1.554452
C	-3.647898	2.439687	-2.463512
C	-3.109170	1.857038	-0.182729
C	-4.602082	3.343835	-2.009593
H	-3.494620	2.312805	-3.530005
C	-4.062085	2.756925	0.284621
H	-2.525102	1.277895	0.523543
C	-4.817700	3.504854	-0.629961
H	-5.186552	3.923350	-2.716670
H	-4.232183	2.875466	1.349432
C	-5.831456	4.402173	-0.159387
N	-6.664593	5.117247	0.223006

INT6A-L9-exo

C	4.122488	-1.098121	-2.949117
C	3.955329	-0.726619	-1.450192
C	2.119758	-1.729747	-2.214860
H	4.299420	-0.244577	-3.603068
H	4.880539	-1.865248	-3.124059
H	3.877584	0.355211	-1.334641
C	-1.251198	-2.647471	-1.219855
C	-1.150895	-3.283493	-2.631272
C	0.749046	-2.254731	-2.135395
H	-1.490199	-3.399362	-0.469365
H	-1.081006	-4.373381	-2.613097
H	-1.936245	-2.965448	-3.315766
N	2.626762	-1.277643	-1.124067
N	0.143526	-2.167408	-0.997528
O	2.818252	-1.675798	-3.334907
O	0.130654	-2.787912	-3.168780
C	5.055400	-1.266095	-0.528831
H	5.147179	-2.348350	-0.678214
H	4.724022	-1.118281	0.501907
C	-2.230900	-1.451901	-1.117908
H	-1.748999	-0.587019	-1.586156

H	-2.361313	-1.210121	-0.063176	C	7.079814	3.499455	0.261321
C	-3.571322	-1.680793	-1.779268	H	5.063200	3.478619	1.059931
C	-4.575558	-2.510443	-1.181257	H	9.021649	3.214069	-0.595961
C	-3.833480	-1.067004	-2.992094	H	9.810833	1.122403	-1.585782
C	-4.412082	-3.131277	0.087334	H	7.259576	4.536112	0.529776
C	-5.823992	-2.691678	-1.868835	C	-1.808782	2.831887	0.564617
C	-5.067073	-1.240081	-3.663873	C	-1.368789	2.864409	-0.713900
H	-3.085974	-0.411194	-3.431043	C	-3.176464	3.339269	0.974383
C	-5.414423	-3.901407	0.636848	C	-2.304162	3.165781	-1.846480
H	-3.503147	-2.972758	0.651748	C	0.025662	2.592031	-1.175666
C	-6.833357	-3.497803	-1.276723	H	-3.072076	4.315309	1.468962
C	-6.039530	-2.042281	-3.114869	H	-3.885208	3.471150	0.158858
H	-5.235600	-0.736444	-4.611260	C	-3.655611	2.340454	2.041571
C	-6.635306	-4.094354	-0.052045	C	-2.097934	4.254400	-2.705411
H	-5.269375	-4.357984	1.611809	C	-3.367376	2.287661	-2.112842
H	-7.772171	-3.630026	-1.808585	C	1.108896	3.349094	-0.699694
H	-6.989815	-2.185510	-3.622783	C	0.264895	1.681875	-2.222594
H	-7.415770	-4.705439	0.391712	C	-2.352720	2.047018	2.813159
C	6.381379	-0.584361	-0.785756	C	-4.699109	2.948017	2.978822
C	6.583912	0.788941	-0.421618	C	-4.216768	1.087459	1.370764
C	7.399909	-1.278455	-1.410224	C	-2.954364	4.477834	-3.784155
C	5.595663	1.581508	0.228853	H	-1.264285	4.927029	-2.522319
C	7.845269	1.404681	-0.722176	C	-4.219075	2.504264	-3.196474
C	8.643083	-0.669345	-1.703020	H	-3.517718	1.429318	-1.464703
H	7.250856	-2.323632	-1.672214	C	2.390958	3.189914	-1.232031
C	5.835223	2.898385	0.560770	H	0.926968	4.106682	0.054572
H	4.632170	1.150760	0.474924	C	1.539359	1.529214	-2.765899
C	8.059830	2.763356	-0.365563	H	-0.571700	1.131063	-2.642913
C	8.859022	0.645593	-1.367333	H	-2.406726	1.096228	3.341454
H	9.422309	-1.248827	-2.188735	H	-2.232146	2.830586	3.569203

C	-1.163688	2.140896	1.793429
O	-4.905256	4.132616	3.110988
O	-5.308639	1.984780	3.694707
O	-3.682673	-0.006436	1.363782
O	-5.367551	1.369515	0.748517
C	-4.016876	3.604278	-4.033009
H	-2.791392	5.333664	-4.433271
H	-5.034191	1.810693	-3.385218
C	2.608477	2.287835	-2.274629
H	3.213150	3.795474	-0.861433
H	1.693089	0.841551	-3.594342
C	-0.028027	2.939989	2.469920
C	-6.293353	2.444025	4.643493
C	-6.026424	0.290799	0.052576
H	-4.679553	3.779110	-4.875849
H	3.595906	2.206527	-2.722073
H	0.881926	2.944576	1.864631
H	-0.326236	3.979433	2.644247
H	0.215152	2.502780	3.446929
H	-7.092472	2.982385	4.127411
H	-6.678542	1.543855	5.122036
H	-5.831388	3.107888	5.378886
H	-5.996795	-0.625690	0.644527
H	-7.051227	0.627549	-0.101880
H	-5.535828	0.117264	-0.907479
C	-0.324604	-2.833607	2.315707
F	-1.595551	-2.787474	1.843160
F	-0.399264	-3.162253	3.622380
F	0.267535	-3.885223	1.679291
Ni	1.342953	-1.412935	0.403729
C	0.385967	-1.518947	2.090942
C	-0.495351	-0.308498	2.464006
H	-1.500039	-0.662621	2.696305
H	-0.102285	0.140578	3.383629
C	-0.630339	0.747517	1.350192
H	-1.290277	0.340853	0.589147
H	0.330897	0.914336	0.847856
C	1.808040	-1.458660	2.499652
C	2.450824	-0.185969	2.501418
C	2.627803	-2.602293	2.747771
C	3.809111	-0.055503	2.792030
H	1.869102	0.713295	2.346914
C	3.974600	-2.469797	3.031598
H	2.186644	-3.589202	2.744809
C	4.577565	-1.193376	3.058889
H	4.273261	0.923960	2.810570
H	4.576361	-3.348659	3.237698
C	5.981210	-1.065403	3.313795
N	7.124722	-0.961791	3.495690

TS6C-L9-exo

C	-2.267597	-4.742905	0.304252
C	-2.366260	-3.201992	0.437362
C	-0.330062	-3.795108	-0.233637
H	-2.252463	-5.262154	1.265822
H	-3.027938	-5.180619	-0.344024
H	-2.696633	-2.909130	1.435819
C	2.890546	-2.372167	-1.014024
C	3.084060	-3.825680	-1.510470
C	1.039588	-3.554769	-0.696673

H	3.147063	-1.647550	-1.790299	C	-4.729574	-2.660015	-0.359819
H	3.084208	-3.917719	-2.600153	C	-5.348695	-1.868998	0.664244
H	3.958662	-4.323677	-1.092285	C	-5.498932	-3.545669	-1.089689
N	-0.966293	-2.775488	0.239493	C	-4.633003	-0.932570	1.463394
N	1.432775	-2.326026	-0.746772	C	-6.758949	-2.008863	0.891531
O	-0.956580	-4.965645	-0.327026	C	-6.886812	-3.692863	-0.854192
O	1.887663	-4.527336	-1.018761	H	-5.032446	-4.133385	-1.877257
C	-3.251629	-2.530895	-0.637469	C	-5.270748	-0.172268	2.420525
H	-3.005860	-2.977002	-1.608873	H	-3.565953	-0.804089	1.316888
H	-2.957462	-1.478528	-0.705043	C	-7.385690	-1.208675	1.885137
C	3.669900	-2.063015	0.289135	C	-7.503569	-2.937882	0.114724
H	3.389879	-2.821470	1.029817	H	-7.459125	-4.398520	-1.448937
H	3.316078	-1.112948	0.696301	C	-6.662741	-0.307764	2.634282
C	5.169587	-2.068885	0.091147	H	-4.701873	0.536607	3.016518
C	5.841459	-0.976733	-0.553184	H	-8.455143	-1.321254	2.043916
C	5.910327	-3.156937	0.515855	H	-8.570098	-3.038033	0.298433
C	5.167947	0.191051	-1.007175	H	-7.155534	0.297226	3.389490
C	7.262623	-1.050233	-0.747120	Ni	-0.044836	-1.060488	-0.059352
C	7.310866	-3.227674	0.325116	C	-0.700878	2.190761	0.101119
H	5.406946	-3.977378	1.022832	C	-1.647458	2.966133	-0.556952
C	5.848246	1.219994	-1.622559	C	0.200252	2.744685	1.206880
H	4.101175	0.286388	-0.854677	C	-1.604731	4.446165	-0.526723
C	7.934761	0.028805	-1.382528	C	-2.794145	2.399129	-1.325331
C	7.972452	-2.196290	-0.297093	H	0.426666	3.796902	1.056718
H	7.855634	-4.099261	0.675678	H	-0.301063	2.668706	2.177477
C	7.247359	1.140514	-1.815408	C	1.522294	1.940897	1.188051
H	5.306942	2.100159	-1.960040	C	-2.792244	5.180039	-0.337205
H	9.010602	-0.040209	-1.522293	C	-0.410545	5.155590	-0.768460
H	9.047604	-2.240609	-0.450149	C	-2.913458	2.622123	-2.706748
H	7.774511	1.955958	-2.301902	C	-3.828047	1.712905	-0.664075

C	1.531920	1.264069	-0.190812
C	2.762549	2.843438	1.267144
C	1.586245	0.915845	2.322601
C	-2.776642	6.572632	-0.342536
H	-3.725747	4.649651	-0.175865
C	-0.400668	6.547861	-0.786306
H	0.509606	4.612704	-0.963926
C	-4.000614	2.112607	-3.415954
H	-2.142320	3.187410	-3.221824
C	-4.922855	1.213334	-1.370824
H	-3.792267	1.588096	0.413820
H	2.204688	0.410416	-0.212643
H	1.889784	1.992978	-0.932012
C	0.095001	0.904865	-0.597415
O	2.769799	4.039835	1.092857
O	3.854262	2.085970	1.463025
O	1.564471	-0.292394	2.184145
O	1.641213	1.533953	3.507443
C	-1.581574	7.261869	-0.565140
H	-3.699639	7.121129	-0.178233
H	0.528778	7.074801	-0.980963
C	-5.003564	1.399258	-2.752512
H	-4.068825	2.279454	-4.487152
H	-5.705851	0.684084	-0.836470
C	-0.131651	0.816775	-2.102475
C	-1.054613	0.507993	0.416939
C	5.121215	2.781517	1.487382
C	1.731794	0.678968	4.668019
H	-1.572183	8.347822	-0.577345
H	-5.850527	1.005849	-3.306987
H	-1.133700	0.465641	-2.351012
H	0.010963	1.801044	-2.568336
H	0.588984	0.117144	-2.537760
H	-0.938632	0.493744	1.498130
H	-2.053667	0.395132	0.024951
H	5.875152	2.001557	1.579339
H	5.261067	3.339858	0.559557
H	5.151619	3.467425	2.337416
H	2.633980	0.065075	4.610540
H	1.774930	1.354284	5.521536
H	0.854585	0.029654	4.728851

INT6C-L9-exo

C	-1.352026	2.811167	-0.680639
C	-2.831269	2.718302	-0.878809
C	-0.694020	1.832938	-0.022183
C	-3.695174	3.665442	-0.309967
C	-3.381545	1.677004	-1.643308
C	0.808954	1.722422	0.041823
C	-0.693530	4.023713	-1.244929
C	-5.076976	3.548556	-0.468920
H	-3.277743	4.489128	0.263003
C	-4.761953	1.566671	-1.814199
H	-2.714899	0.957264	-2.112359
H	1.092583	0.765648	-0.422447
H	1.305770	2.507140	-0.526730
C	1.329709	1.724315	1.525836
C	0.000594	4.917234	-0.412655
C	-0.815542	4.328729	-2.610074
C	-5.613989	2.497684	-1.217112

H	-5.735315	4.281152	-0.010155	H	5.032426	-0.287918	1.939642
H	-5.171585	0.749715	-2.401345	H	5.083650	0.608895	3.490073
C	0.201385	1.245058	2.479173	H	3.928419	-0.754508	3.280633
C	2.370181	0.665203	1.771624	H	3.753049	4.876725	2.339668
C	1.841669	3.121149	1.892180	H	4.212821	4.904369	0.601196
C	0.577067	6.075165	-0.939368	H	2.634082	5.567904	1.133569
H	0.056519	4.717835	0.653629	Ni	0.456111	-1.304096	1.000807
C	-0.225328	5.476153	-3.137490	N	1.547911	-2.690382	-0.103068
H	-1.379002	3.659450	-3.254614	N	-1.055139	-2.582937	0.453528
H	-6.689620	2.403653	-1.333373	C	2.877591	-2.860927	-0.727600
H	-0.454740	2.104048	2.669939	C	0.836479	-3.698003	-0.448432
H	0.625340	0.967215	3.451041	C	-2.541868	-2.798102	0.452792
C	-0.614434	0.104283	1.856179	C	-0.575802	-3.674532	-0.052329
O	2.114369	-0.540751	1.587359	C	2.733824	-4.213350	-1.488563
O	3.491571	1.057128	2.320171	H	3.007603	-2.034082	-1.431889
O	1.384773	3.811828	2.774294	C	4.019206	-2.827526	0.306407
O	2.836595	3.493471	1.069842	O	1.353772	-4.650499	-1.211730
C	0.474164	6.353246	-2.303399	C	-2.634971	-4.331283	0.402498
H	1.088356	6.772132	-0.280350	H	-2.990402	-2.398567	1.358796
H	-0.319861	5.692925	-4.197909	C	-3.179275	-2.152479	-0.805465
C	-1.478235	-0.517847	2.949141	O	-1.371660	-4.712232	-0.252369
C	-1.432040	0.717867	0.702055	H	2.829779	-4.129053	-2.571767
C	4.449090	0.069349	2.789004	H	3.401775	-4.993383	-1.117106
C	3.392578	4.802765	1.310970	H	4.063225	-3.795424	0.819646
H	0.920545	7.255311	-2.712212	H	3.749491	-2.087766	1.061624
H	-2.181029	-1.255944	2.565307	C	5.357000	-2.503958	-0.323503
H	-0.860075	-1.008959	3.709472	H	-2.638547	-4.806829	1.387839
H	-2.074577	0.256362	3.457814	H	-3.456046	-4.706635	-0.206592
H	-1.723130	-0.056635	-0.009338	H	-2.727845	-2.621127	-1.688528
H	-2.372449	1.122984	1.102998	H	-2.913755	-1.095163	-0.843048

C	-4.681993	-2.318550	-0.847463
C	5.645436	-1.169536	-0.770249
C	6.312361	-3.489523	-0.477841
C	-5.532487	-1.526417	-0.006715
C	-5.246432	-3.245373	-1.704945
C	4.722443	-0.090618	-0.651286
C	6.929378	-0.892806	-1.348720
C	7.571557	-3.218184	-1.065192
H	6.100810	-4.497568	-0.127908
C	-5.038457	-0.537962	0.888058
C	-6.953090	-1.719440	-0.082436
C	-6.646521	-3.439743	-1.771176
H	-4.601908	-3.830038	-2.358263
C	5.051801	1.189126	-1.045434
H	3.739271	-0.274357	-0.236735
C	7.233654	0.433576	-1.758895
C	7.873792	-1.945962	-1.489018
H	8.297308	-4.019191	-1.169011
C	-5.891240	0.219481	1.659997
H	-3.974796	-0.348457	0.947256
C	-7.805482	-0.923220	0.729137
C	-7.480723	-2.692311	-0.974231
H	-7.053748	-4.175403	-2.458263
C	6.322572	1.455470	-1.606583
H	4.334481	1.995081	-0.912976
H	8.210450	0.630483	-2.193392
H	8.841657	-1.726365	-1.932008
C	-7.290101	0.026410	1.582707
H	-5.486546	0.981367	2.319901
H	-8.879538	-1.076487	0.660936
H	-8.557887	-2.830466	-1.020213
H	6.575978	2.464399	-1.918502
H	-7.954099	0.630529	2.193960

TS6D-L9-exo

C	-3.723502	-2.903607	1.105087
C	-3.343924	-1.665507	0.262974
C	-1.576773	-2.966230	0.562336
H	-3.863963	-2.675803	2.165716
H	-4.578044	-3.461277	0.722961
H	-3.653486	-0.741031	0.749645
C	1.930090	-3.183091	-0.241807
C	1.679785	-4.601078	0.319610
C	-0.199376	-3.418509	0.338332
H	2.422866	-3.209102	-1.215444
H	1.667604	-5.377652	-0.449794
H	2.352896	-4.884307	1.129101
N	-1.863034	-1.750901	0.233621
N	0.555165	-2.657848	-0.383639
O	-2.537898	-3.771220	1.004152
O	0.316671	-4.520680	0.873572
C	-3.871940	-1.715161	-1.192872
H	-3.462502	-2.614710	-1.668204
H	-3.449723	-0.861341	-1.733303
C	2.704166	-2.259791	0.728085
H	2.248037	-2.352667	1.721540
H	2.524851	-1.228579	0.405607
C	4.177102	-2.578434	0.793663
C	5.057921	-2.174219	-0.263245
C	4.679834	-3.301604	1.858935

C	4.629568	-1.408151	-1.383124	H	-8.419007	2.602131	-0.516420
C	6.442371	-2.547439	-0.190499	Ni	-0.213933	-0.869577	-0.738470
C	6.045648	-3.665531	1.929924	C	0.428929	1.535854	0.196002
H	4.014006	-3.590810	2.669428	C	-0.180210	1.771135	1.390891
C	5.508223	-1.041821	-2.381320	C	1.914172	1.774477	0.013203
H	3.592426	-1.099418	-1.463241	C	0.621789	1.929933	2.635285
C	7.322912	-2.154115	-1.233844	C	-1.650385	1.873477	1.610410
C	6.906720	-3.299187	0.923034	H	2.339970	2.348623	0.832502
H	6.405553	-4.232919	2.782966	H	2.486686	0.844355	-0.063158
C	6.869624	-1.421441	-2.308308	C	2.063797	2.565339	-1.318264
H	5.151008	-0.442019	-3.211732	C	1.609055	0.988261	2.974256
H	8.368434	-2.444539	-1.168286	C	0.370613	2.991371	3.520922
H	7.956794	-3.576333	0.966180	C	-2.323675	0.923325	2.392952
H	7.553877	-1.131396	-3.100441	C	-2.352290	3.002752	1.162076
C	-5.381715	-1.742890	-1.276768	C	0.948293	2.148473	-2.274411
C	-6.167471	-0.563285	-1.057854	C	1.912016	4.074105	-1.050574
C	-6.020239	-2.940989	-1.542698	C	3.438255	2.257603	-1.923488
C	-5.600137	0.714046	-0.795339	C	2.343922	1.116414	4.152415
C	-7.599584	-0.664938	-1.116314	H	1.782659	0.144196	2.312479
C	-7.430571	-3.039359	-1.596427	C	1.113334	3.125918	4.691945
H	-5.423862	-3.831969	-1.727997	H	-0.400833	3.716031	3.279007
C	-6.394824	1.824489	-0.608189	C	-3.680807	1.074656	2.679207
H	-4.523672	0.828800	-0.740451	H	-1.773092	0.068120	2.774623
C	-8.389021	0.498347	-0.912376	C	-3.707668	3.159123	1.457662
C	-8.203462	-1.923183	-1.383891	H	-1.819656	3.763862	0.598636
H	-7.893299	-3.998104	-1.810690	H	1.308794	1.909963	-3.271556
C	-7.803668	1.719590	-0.665222	H	0.123612	2.852746	-2.345990
H	-5.931981	2.786896	-0.412954	C	-0.217815	1.059168	-1.069703
H	-9.471145	0.406614	-0.960119	O	1.537181	4.562122	-0.011035
H	-9.287843	-1.985393	-1.424179	O	2.201496	4.762508	-2.166353

O	3.618248	1.547141	-2.891921
O	4.400348	2.816389	-1.189062
C	2.101384	2.189857	5.012027
H	3.099976	0.376571	4.400843
H	0.919844	3.961366	5.358629
C	-4.378191	2.190538	2.207350
H	-4.194096	0.328797	3.280589
H	-4.236443	4.042624	1.110809
C	-1.615580	1.459851	-1.517477
C	0.666267	0.179566	-2.025786
C	2.088624	6.200285	-2.063516
C	5.762588	2.553190	-1.603571
H	2.672861	2.293396	5.929914
H	-5.434681	2.305865	2.427823
H	-2.349472	1.066506	-0.814951
H	-1.757151	2.547117	-1.551159
H	-1.831242	1.046988	-2.507112
H	0.261567	-0.028488	-3.016824
H	1.748276	0.102708	-1.977157
H	2.773958	6.575094	-1.299543
H	2.356053	6.582792	-3.047747
H	1.065632	6.480554	-1.799970
H	5.901102	2.854306	-2.644598
H	6.384023	3.151453	-0.938626
H	5.983650	1.489207	-1.495053

INT6D-L9-exo

C	-1.028173	1.253711	0.462832
C	-1.604598	1.370725	1.849004
C	0.372350	1.541065	0.270462
C	-1.346111	2.525700	2.604987
C	-2.451580	0.393639	2.388752
C	0.925888	2.326670	-0.904935
C	-1.987510	1.541557	-0.652438
C	-1.926042	2.692004	3.864322
H	-0.686409	3.292486	2.211032
C	-3.031960	0.557132	3.646248
H	-2.632652	-0.511599	1.825798
H	1.436576	1.686010	-1.629560
H	0.132360	2.834985	-1.450021
C	1.959842	3.377499	-0.406709
C	-1.686149	1.200241	-1.987568
C	-3.229844	2.149650	-0.399002
C	-2.772422	1.712453	4.388385
H	-1.714739	3.591937	4.434979
H	-3.684074	-0.216059	4.042697
C	2.962618	2.742403	0.599275
C	2.733849	3.908531	-1.619245
C	1.161675	4.510185	0.257077
C	-2.581399	1.458357	-3.021325
H	-0.752062	0.692675	-2.211164
C	-4.136434	2.392824	-1.432573
H	-3.493708	2.426955	0.615350
H	-3.222814	1.845397	5.367705
H	2.792976	3.185382	1.584133
H	3.993652	2.974013	0.316064
C	2.770963	1.216242	0.686423
O	2.792055	3.360419	-2.698113
O	3.398017	5.029433	-1.298552
O	1.069381	4.692770	1.453034

O	0.495899	5.221184	-0.664231	H	-3.404033	-4.175347	-1.173074
C	-3.817515	2.051525	-2.747310	H	-3.103485	-1.811550	-2.298865
H	-2.322463	1.179942	-4.038912	H	-3.423028	-0.476396	-1.209823
H	-5.095724	2.847866	-1.204305	C	-5.011244	-1.938817	-1.307472
C	1.337933	0.910567	1.092647	H	3.060979	-3.309644	-2.353570
C	4.190688	5.613176	-2.354021	H	3.493960	-4.268712	-0.906846
C	-0.363568	6.262991	-0.155953	H	2.844016	-3.034666	1.535985
H	-4.524524	2.239506	-3.549769	H	3.473175	-1.398107	1.461853
H	3.551939	5.881708	-3.199185	C	4.887019	-2.943919	0.895636
H	4.648223	6.499899	-1.916460	C	-5.888860	-1.428258	-0.294047
H	4.954172	4.906853	-2.690347	C	-5.503871	-2.835286	-2.238280
H	0.212606	6.967645	0.448173	C	6.001087	-2.178631	0.416508
H	-0.777646	6.752251	-1.036855	C	5.084518	-4.259230	1.277505
H	-1.159311	5.827634	0.454904	C	-5.479742	-0.468745	0.672947
Ni	0.070314	-0.456607	0.173598	C	-7.245189	-1.896104	-0.248704
N	-1.301708	-1.924538	-0.232548	C	-6.847716	-3.278521	-2.205783
N	1.386778	-1.901752	-0.308339	H	-4.846169	-3.205828	-3.022040
C	-2.756362	-2.271080	-0.219285	C	5.901928	-0.814333	0.026732
C	-0.687810	-3.001377	-0.586243	C	7.288594	-2.810036	0.329200
C	2.841230	-2.188825	-0.444047	C	6.355288	-4.875908	1.200038
C	0.773598	-2.960397	-0.713860	H	4.242721	-4.836505	1.653949
C	-2.740533	-3.813833	-0.388089	C	-6.343853	-0.020876	1.648569
H	-3.173499	-2.000948	0.750925	H	-4.481856	-0.052876	0.631046
C	-3.548702	-1.550128	-1.331806	C	-8.111746	-1.413147	0.769027
O	-1.360225	-4.120219	-0.790706	C	-7.696691	-2.826621	-1.223526
C	2.845753	-3.484693	-1.296743	H	-7.198762	-3.979450	-2.957323
H	3.322717	-1.371743	-0.982506	C	6.998143	-0.117701	-0.434269
C	3.488043	-2.365666	0.953282	H	4.952243	-0.300794	0.105978
O	1.459274	-3.970646	-1.218719	C	8.398111	-2.064230	-0.151913
H	-2.928270	-4.361300	0.538550	C	7.433455	-4.165894	0.728662

H	6.469600	-5.909092	1.513825
C	-7.672895	-0.502585	1.704330
H	-5.999751	0.712007	2.373638
H	-9.135947	-1.776682	0.794698
H	-8.727103	-3.170129	-1.182556
C	8.260563	-0.747909	-0.530297
H	6.893762	0.924875	-0.722025
H	9.365915	-2.555220	-0.213329
H	8.413629	-4.630338	0.659413
H	-8.346969	-0.144391	2.476985
H	9.117704	-0.190121	-0.895495
C	1.196037	0.455279	2.534323
H	1.431789	1.300404	3.196784
H	0.205589	0.100404	2.811721
H	1.919261	-0.339560	2.739106
H	3.475009	0.808571	1.416202
H	3.007006	0.764897	-0.284081

INT5A-L9-exo-Ni(I)

C	-3.976654	-2.425414	-1.577055
C	-3.473825	-1.012849	-1.199126
C	-1.774636	-2.313336	-1.904100
H	-4.595122	-2.894234	-0.810127
H	-4.523831	-2.419752	-2.529235
H	-3.452664	-0.913021	-0.107051
C	1.748860	-2.141012	-2.018681
C	1.529082	-3.527342	-2.656714
C	-0.431506	-2.596640	-2.169300
H	2.486996	-1.547546	-2.566225
H	1.660510	-3.500841	-3.746115
H	2.142431	-4.323197	-2.228917
N	-2.099920	-1.024242	-1.707726
N	0.410685	-1.563121	-2.116831
O	-2.776416	-3.219610	-1.740074
O	0.137054	-3.823027	-2.364710
C	-4.346875	0.109192	-1.785895
H	-4.356886	0.002935	-2.876928
H	-3.871277	1.068680	-1.568092
C	2.133808	-2.239117	-0.517664
H	1.486809	-3.003850	-0.071339
H	1.845374	-1.294019	-0.052411
C	3.576626	-2.559255	-0.223480
C	4.560276	-1.517232	-0.175622
C	3.970310	-3.859992	0.030184
C	4.235828	-0.152519	-0.403220
C	5.919150	-1.843177	0.145205
C	5.314535	-4.185872	0.330869
H	3.223368	-4.650775	0.022132
C	5.186911	0.841602	-0.316149
H	3.210209	0.104240	-0.633693
C	6.880111	-0.798632	0.223675
C	6.269907	-3.198142	0.389933
H	5.583006	-5.220650	0.526187
C	6.526581	0.513585	0.001333
H	4.904303	1.878211	-0.479561
H	7.907488	-1.056483	0.471057
H	7.302717	-3.438568	0.630843
H	7.271646	1.301089	0.073845
C	-5.759312	0.055111	-1.242545
C	-6.048898	0.441438	0.110248

C	-6.782543	-0.438861	-2.030272	C	1.809303	2.100883	-1.570498
C	-5.066226	0.984259	0.985354	C	3.975017	0.306685	3.843091
C	-7.383410	0.275351	0.614791	H	2.574298	1.866294	3.390755
C	-8.100960	-0.586283	-1.537137	C	3.316420	-1.922375	3.212863
H	-6.568249	-0.725168	-3.057396	H	1.401471	-2.110257	2.269508
C	-5.371855	1.315518	2.287325	C	-2.575225	-0.738498	3.725186
H	-4.056632	1.144955	0.628769	H	-1.125428	0.813338	4.098368
C	-7.665879	0.631472	1.961395	C	-2.153727	-2.130065	1.799498
C	-8.394284	-0.243484	-0.238777	H	-0.418408	-1.622908	0.637726
H	-8.876019	-0.977482	-2.190799	H	-0.424247	3.517068	-1.266431
C	-6.683462	1.133891	2.785095	H	-0.004453	4.376266	0.211543
H	-4.595629	1.716049	2.933409	C	-0.585529	2.285956	0.571797
H	-8.679839	0.497282	2.331661	O	3.413476	4.596249	0.021177
H	-9.401778	-0.362558	0.153098	O	1.963322	5.066819	-1.646991
H	-6.914026	1.397412	3.813992	O	1.116660	1.122545	-1.830563
Ni	-0.536780	0.084782	-1.476748	O	2.908897	2.367852	-2.273482
C	0.618009	1.596156	1.258743	C	4.255998	-1.058708	3.778629
C	0.611717	0.489765	2.039630	H	4.681867	0.989363	4.307477
C	1.873447	2.376409	0.917029	H	3.518362	-2.988197	3.157244
C	1.852166	-0.042178	2.688502	C	-2.949271	-1.800675	2.898919
C	-0.633281	-0.301611	2.324095	H	-3.184469	-0.479375	4.587086
H	2.050824	3.187788	1.633744	H	-2.427865	-2.952643	1.143726
H	2.785857	1.778981	0.906015	C	-1.494401	2.960187	1.630341
C	1.569055	3.071436	-0.434762	C	-1.441263	1.361276	-0.320500
C	2.786127	0.805535	3.311039	C	2.733565	6.243534	-1.948033
C	2.123385	-1.422713	2.694736	C	3.227829	1.482757	-3.371726
C	-1.419205	-0.005873	3.447387	H	5.190776	-1.447152	4.173159
C	-1.007432	-1.384996	1.515542	H	-3.853696	-2.364430	3.109976
C	0.065486	3.407629	-0.296253	H	-2.011929	2.209261	2.231990
C	2.437690	4.310622	-0.637621	H	-0.920348	3.607981	2.307013

H	-2.253650	3.575187	1.130737
H	-2.074847	0.745343	0.322444
H	-2.110719	2.001684	-0.915851
H	3.753103	5.966875	-2.231360
H	2.218612	6.725597	-2.779290
H	2.773757	6.907568	-1.080200
H	2.346228	1.321554	-3.995301
H	4.017075	1.991467	-3.924027
H	3.590219	0.531272	-2.978952

TS6C-L9-exo-Ni(I)

C	-1.394054	-4.920906	-0.396576
C	-1.656424	-3.484499	0.114890
C	0.395050	-3.652531	-0.784305
H	-1.191194	-5.618054	0.426351
H	-2.196446	-5.321056	-1.021218
H	-1.975277	-3.506072	1.161500
C	3.529868	-1.977008	-0.901773
C	3.787372	-3.137538	-1.891270
C	1.695650	-3.238560	-1.122329
H	3.844791	-1.020347	-1.331087
H	3.818082	-2.788483	-2.930978
H	4.689838	-3.714412	-1.674806
N	-0.338933	-2.863194	0.005155
N	2.080620	-2.026078	-0.736485
O	-0.199975	-4.805245	-1.205788
O	2.640681	-4.011296	-1.733436
C	-2.714022	-2.726853	-0.726467
H	-2.466153	-2.862727	-1.785899
H	-2.601992	-1.659704	-0.523456
C	4.209849	-2.170920	0.479518
H	3.936734	-3.169472	0.841243
H	3.743623	-1.454766	1.164109
C	5.711067	-2.020870	0.462843
C	6.324142	-0.724953	0.389540
C	6.520048	-3.142177	0.502945
C	5.572834	0.483607	0.327591
C	7.757138	-0.628776	0.380590
C	7.931560	-3.047125	0.478683
H	6.057453	-4.124441	0.568898
C	6.193692	1.714170	0.276603
H	4.489645	0.444050	0.301779
C	8.366288	0.654278	0.330968
C	8.538557	-1.814940	0.422975
H	8.529959	-3.953611	0.513498
C	7.605880	1.800972	0.283610
H	5.587478	2.610734	0.210930
H	9.452572	0.712687	0.328834
H	9.622660	-1.729477	0.412238
H	8.087275	2.774630	0.243672
C	-4.129228	-3.173047	-0.456945
C	-4.781189	-2.829471	0.774859
C	-4.813937	-3.932994	-1.386486
C	-4.156717	-2.045403	1.786799
C	-6.131456	-3.262748	0.996743
C	-6.139211	-4.374340	-1.159752
H	-4.325010	-4.186845	-2.324154
C	-4.824737	-1.700794	2.941799
H	-3.141494	-1.693580	1.643154
C	-6.792836	-2.888385	2.198287

C	-6.786877	-4.043736	0.006752	H	-3.855765	0.866562	1.215866
H	-6.642519	-4.968244	-1.917895	H	1.429772	0.643318	-1.336589
C	-6.158879	-2.123554	3.151531	H	0.830326	2.125751	-2.105778
H	-4.326676	-1.094551	3.693684	C	-0.670629	1.169575	-0.940250
H	-7.818158	-3.217753	2.350442	O	3.445936	3.164579	-0.636207
H	-7.808370	-4.369938	0.187209	O	1.680895	4.520163	-0.993022
H	-6.678825	-1.842502	4.063203	O	1.875918	0.333662	1.172106
Ni	0.614676	-1.157836	0.342467	O	2.556578	2.307040	1.981331
C	-1.249581	2.273576	-0.103178	C	-3.331547	7.068316	0.388836
C	-2.554100	2.866452	-0.181293	H	-5.074734	6.402886	1.473929
C	-0.081784	3.012149	0.574911	H	-1.550359	7.398677	-0.783512
C	-2.782197	4.305125	0.030036	C	-6.143566	0.751354	-1.295421
C	-3.768706	2.094905	-0.557422	H	-6.107120	2.003528	-3.054220
H	-0.103154	4.075580	0.363259	H	-5.922306	-0.309034	0.568025
H	-0.126199	2.921944	1.665128	C	-1.369938	0.598892	-2.158523
C	1.243734	2.393938	0.009094	C	-1.036331	0.524092	0.368587
C	-3.927018	4.745655	0.734418	C	2.568716	5.453547	-1.637935
C	-1.944576	5.303224	-0.523228	C	3.245678	1.612028	3.046361
C	-4.449480	2.417884	-1.748652	H	-3.539731	8.125418	0.529783
C	-4.327099	1.100687	0.266502	H	-7.052704	0.228631	-1.579913
C	0.789920	1.519901	-1.192448	H	-2.419110	0.372252	-1.963021
C	2.259783	3.397081	-0.533053	H	-1.327341	1.305673	-2.995473
C	1.930403	1.557464	1.081743	H	-0.866219	-0.330569	-2.448277
C	-4.192375	6.099851	0.915479	H	-0.486048	0.592229	1.299970
H	-4.604090	4.003282	1.146881	H	-1.995915	0.036267	0.419990
C	-2.210646	6.658088	-0.338060	H	1.938724	6.289476	-1.941463
H	-1.100350	5.008259	-1.139779	H	3.343270	5.783230	-0.940760
C	-5.612302	1.747050	-2.120702	H	3.043584	4.987756	-2.505620
H	-4.043713	3.199833	-2.384644	H	4.102624	1.073187	2.635640
C	-5.503992	0.440551	-0.094784	H	3.575943	2.394567	3.728484

H	2.568827	0.914644	3.545356

TS6D-L9-exo-Ni(I)

C	-3.172359	-2.462340	2.136896
C	-2.958544	-1.734841	0.796404
C	-1.096250	-2.803961	1.407611
H	-3.125965	-1.763851	2.983316
H	-4.087556	-3.053700	2.196231
H	-3.371547	-0.722906	0.808756
C	2.230951	-3.240504	0.232349
C	2.063481	-4.507417	1.099417
C	0.168595	-3.326447	1.122999
H	2.659825	-3.488368	-0.743691
H	1.933211	-5.405644	0.482412
H	2.874454	-4.670169	1.814304
N	-1.498078	-1.719696	0.720806
N	0.852060	-2.788657	0.096308
O	-2.043889	-3.370173	2.216693
O	0.843843	-4.272967	1.843524
C	-3.541074	-2.526722	-0.411414
H	-3.116987	-3.536694	-0.369708
H	-3.143740	-2.066539	-1.322206
C	3.092322	-2.144552	0.919207
H	2.789994	-2.091907	1.971924
H	2.803916	-1.188195	0.472669
C	4.582997	-2.337254	0.809714
C	5.262123	-2.062530	-0.425486
C	5.321969	-2.767463	1.895856
C	4.580046	-1.631591	-1.598101
C	6.689105	-2.202118	-0.489195
C	6.726097	-2.931926	1.824748
H	4.812384	-2.967505	2.835630
C	5.261578	-1.329531	-2.757556
H	3.501049	-1.545613	-1.584444
C	7.363340	-1.881774	-1.699079
C	7.398241	-2.647098	0.659456
H	7.269728	-3.271404	2.702290
C	6.670732	-1.449819	-2.808771
H	4.716462	-0.983026	-3.630637
H	8.445595	-1.984670	-1.732858
H	8.478533	-2.756057	0.600164
H	7.201347	-1.203317	-3.724146
C	-5.048326	-2.609964	-0.433887
C	-5.853023	-1.538643	-0.948422
C	-5.680248	-3.728542	0.080949
C	-5.294328	-0.361389	-1.516828
C	-7.284896	-1.654164	-0.910109
C	-7.089913	-3.837827	0.127876
H	-5.074135	-4.551279	0.453680
C	-6.092950	0.636758	-2.030631
H	-4.218023	-0.242818	-1.547096
C	-8.080603	-0.601227	-1.436809
C	-7.877735	-2.820004	-0.355307
H	-7.543323	-4.734180	0.542616
C	-7.501778	0.519450	-1.989166
H	-5.634432	1.525197	-2.453162
H	-9.163189	-0.701021	-1.400637
H	-8.962294	-2.895258	-0.327895
H	-8.123392	1.313813	-2.394039
Ni	-0.029178	-1.131160	-0.546177

C	0.063385	1.547953	-0.044780	C	0.328421	4.048443	4.429060
C	-0.835029	2.261891	0.685921	H	0.984620	2.069612	4.985089
C	1.546231	1.678847	0.213560	H	-0.441794	5.886043	3.598572
C	-0.433639	2.876287	1.984194	C	-4.905265	3.255544	-0.266552
C	-2.249054	2.547333	0.311043	H	-5.443833	1.894847	1.318131
H	1.794766	2.324138	1.051477	H	-4.063561	4.541006	-1.781010
H	2.025107	0.717639	0.400763	C	-1.421981	0.836538	-2.123036
C	2.093443	2.279824	-1.125923	C	0.997546	-0.220561	-1.832285
C	0.154578	2.103146	2.999662	C	2.577784	5.735292	0.125400
C	-0.657322	4.243525	2.225769	C	5.653519	1.464365	-0.374015
C	-3.310221	2.007490	1.054659	H	0.622785	4.501549	5.371916
C	-2.534300	3.470629	-0.706684	H	-5.934214	3.518112	-0.495432
C	1.240329	1.727257	-2.268951	H	-2.296877	0.650056	-1.498719
C	1.864804	3.798025	-1.007662	H	-1.556158	1.825055	-2.581117
C	3.584312	2.007345	-1.375074	H	-1.406955	0.081801	-2.914248
C	0.534371	2.684680	4.210284	H	0.854680	-0.563910	-2.856879
H	0.301487	1.039870	2.828067	H	2.041768	-0.177729	-1.538396
C	-0.270475	4.825411	3.432376	H	1.684946	5.797547	0.754510
H	-1.133109	4.844605	1.455895	H	3.463093	6.040046	0.683128
C	-4.629596	2.343703	0.756522	H	2.444196	6.353874	-0.765260
H	-3.086933	1.323404	1.868496	H	6.132997	2.437125	-0.516723
C	-3.855986	3.821389	-0.993069	H	5.963358	1.012454	0.567527
H	-1.709521	3.917712	-1.255130	H	5.889610	0.808194	-1.211576
H	1.821243	1.434292	-3.138867				
H	0.452254	2.410103	-2.574311	**TS8A**			
C	-0.176292	0.724197	-1.262237	C	-0.265690	-0.416132	0.096383
O	0.910967	4.394418	-1.458812	C	-1.561311	0.116658	0.049511
O	2.812088	4.360937	-0.242774	C	0.961914	0.471974	0.294163
O	4.103482	2.118616	-2.466139	C	-1.827540	1.578974	0.114051
O	4.225830	1.646413	-0.257695	C	-2.794693	-0.719073	-0.043186

H	0.929736	1.342582	-0.356903
H	1.030845	0.855985	1.315746
C	2.210102	-0.373003	-0.089988
C	-2.747853	2.087825	1.050656
C	-1.248380	2.484896	-0.795887
C	-3.534219	-0.738788	-1.238127
C	-3.289886	-1.441066	1.054912
C	1.654939	-1.690291	-0.701301
C	3.050561	0.339893	-1.155152
C	3.097857	-0.632515	1.128377
C	-3.043879	3.448693	1.104582
H	-3.231040	1.401601	1.740538
C	-1.547267	3.846048	-0.745029
H	-0.578192	2.113296	-1.565695
C	-4.702491	-1.491486	-1.346695
H	-3.174923	-0.162504	-2.086410
C	-4.463959	-2.190763	0.950913
H	-2.757129	-1.398368	1.999867
H	2.264630	-2.548535	-0.408253
H	1.664063	-1.619784	-1.795342
C	0.199256	-1.826400	-0.234370
O	2.697858	1.280503	-1.831209
O	4.241030	-0.278181	-1.299721
O	3.302821	-1.703523	1.656147
O	3.616794	0.530160	1.577012
C	-2.440837	4.336619	0.209867
H	-3.749072	3.816802	1.845624
H	-1.088649	4.522293	-1.461919
C	-5.169242	-2.226415	-0.253005
H	-5.250972	-1.504132	-2.285120
H	-4.829108	-2.742053	1.813721
C	-0.619820	-2.738504	-1.139864
C	5.103292	0.268434	-2.312501
C	4.468634	0.422048	2.730086
H	-2.673787	5.397379	0.248978
H	-6.080233	-2.812969	-0.336480
H	-1.620741	-2.922781	-0.746766
H	-0.723150	-2.300809	-2.139705
H	-0.109602	-3.704651	-1.235461
H	6.007046	-0.340874	-2.286774
H	4.624901	0.209409	-3.294177
H	5.334516	1.314334	-2.092244
H	5.326268	-0.220259	2.511170
H	4.793660	1.439254	2.950039
H	3.916626	0.001348	3.575298
C	0.034113	-2.000071	1.229612
H	-0.893508	-2.407311	1.606480
H	0.858190	-1.866261	1.917901

INT8A

C	0.340962	-0.522459	0.270393
C	1.688202	0.037911	-0.045770
C	-0.813871	-0.224969	-0.692057
C	1.828361	1.470872	-0.227234
C	2.832742	-0.863252	-0.159324
H	-0.701497	0.766845	-1.133655
H	-0.854632	-0.943404	-1.517350
C	-2.131792	-0.254158	0.143461
C	2.909957	2.029579	-0.961098
C	0.858754	2.382733	0.269444

C	4.110016	-0.510535	0.338704
C	2.694597	-2.147025	-0.738938
C	-1.701996	-0.139471	1.640136
C	-3.030987	0.939251	-0.191711
C	-2.913358	-1.543844	-0.119078
C	3.025858	3.400697	-1.154216
H	3.645671	1.367592	-1.404767
C	0.978891	3.752818	0.070760
H	0.004166	2.008515	0.818380
C	5.187586	-1.387221	0.250465
H	4.238604	0.453889	0.820267
C	3.775845	-3.017180	-0.833974
H	1.726432	-2.445363	-1.128927
H	-2.328577	-0.782032	2.265257
H	-1.835438	0.893133	1.987263
C	-0.226723	-0.502245	1.708262
O	-2.689035	1.950844	-0.761455
O	-4.267863	0.749966	0.315686
O	-3.100920	-2.444720	0.668946
O	-3.352148	-1.569477	-1.395636
C	2.065737	4.277019	-0.635962
H	3.864012	3.789578	-1.727076
H	0.215734	4.416608	0.468369
C	5.030537	-2.644695	-0.340208
H	6.152890	-1.092037	0.653916
H	3.641409	-3.991192	-1.297639
C	0.565676	0.023540	2.883522
C	-5.190031	1.834753	0.114473
C	-4.101977	-2.738473	-1.766455
H	2.156856	5.348411	-0.791331
H	5.872572	-3.327834	-0.409726
H	1.629785	-0.210287	2.774342
H	0.471347	1.112529	2.975306
H	0.213763	-0.426941	3.820059
H	-6.123383	1.515023	0.578599
H	-4.815886	2.747377	0.586953
H	-5.332315	2.020994	-0.953696
H	-4.994834	-2.831977	-1.141939
H	-4.375299	-2.591490	-2.811697
H	-3.491310	-3.638229	-1.649286
C	0.182303	-1.800040	1.071234
H	1.089719	-2.259599	1.451666
H	-0.581806	-2.514649	0.777396

TS9A

C	0.376598	-0.631713	-0.213803
C	1.621112	-0.001765	-0.274302
C	-0.895084	-0.007556	-0.761525
C	1.742664	1.471539	-0.372975
C	2.881676	-0.771816	-0.177228
H	-0.797131	1.068913	-0.891827
H	-1.083430	-0.422266	-1.760332
C	-2.137887	-0.288895	0.141395
C	2.709473	2.062605	-1.210616
C	0.923891	2.327917	0.389671
C	3.956139	-0.307705	0.610048
C	3.062619	-1.978791	-0.883390
C	-1.670477	-0.617587	1.594367
C	-3.042719	0.946560	0.217937
C	-2.964049	-1.448393	-0.428429

C	2.835431	3.447165	-1.298849
H	3.359871	1.420569	-1.797808
C	1.045780	3.713040	0.298055
H	0.192029	1.895527	1.065547
C	5.143496	-1.030201	0.707119
H	3.844916	0.627175	1.151482
C	4.250504	-2.701081	-0.787165
H	2.262667	-2.334109	-1.527003
H	-2.420443	-1.275690	2.055189
H	-1.653366	0.305508	2.189559
C	-0.304679	-1.229643	1.593938
O	-2.736427	2.076192	-0.089537
O	-4.234626	0.616852	0.760752
O	-3.119468	-2.535980	0.082079
O	-3.490090	-1.105611	-1.623140
C	2.001189	4.280947	-0.548203
H	3.583791	3.877856	-1.959443
H	0.396857	4.348887	0.894561
C	5.297153	-2.233402	0.012232
H	5.951629	-0.654150	1.329481
H	4.364563	-3.625532	-1.347805
C	0.610093	-1.057567	2.764900
C	-5.155257	1.707288	0.938119
C	-4.286635	-2.118759	-2.260333
H	2.098219	5.361021	-0.618932
H	6.224254	-2.795382	0.086087
H	1.658981	-1.054592	2.445414
H	0.415390	-0.121375	3.302984
H	0.495181	-1.880807	3.488962
H	-6.049316	1.266190	1.379511
H	-4.728447	2.463704	1.602751
H	-5.386623	2.170790	-0.024927
H	-5.136090	-2.389199	-1.626938
H	-4.627791	-1.676549	-3.196689
H	-3.686925	-3.013256	-2.450894
C	0.151099	-1.995951	0.396756
H	1.054386	-2.574857	0.578901
H	-0.601063	-2.617421	-0.095373

INT9A

C	-1.603787	-0.104075	-0.317539
C	-2.926458	-0.734090	-0.038743
C	-0.469762	-0.811159	-0.524414
C	-3.737023	-0.245783	1.001307
C	-3.418838	-1.789414	-0.824791
C	0.830679	-0.186573	-0.971720
C	-1.624864	1.392566	-0.354074
C	-4.980294	-0.816408	1.267416
H	-3.380949	0.586136	1.602348
C	-4.666873	-2.356960	-0.564215
H	-2.821721	-2.152495	-1.656167
H	1.147740	-0.695828	-1.889964
H	0.719731	0.869858	-1.216759
C	1.991461	-0.381014	0.060386
C	-0.954038	2.146961	0.620855
C	-2.368255	2.068729	-1.334103
C	-5.450382	-1.876010	0.486297
H	-5.586035	-0.430952	2.083486
H	-5.029673	-3.169766	-1.188141
C	1.651045	-1.537626	1.052396

C	3.289741	-0.745671	-0.672917
C	2.211308	0.925150	0.828502
C	-1.009180	3.543025	0.602439
H	-0.394800	1.636661	1.397953
C	-2.413022	3.462344	-1.360160
H	-2.910190	1.490955	-2.078139
H	-6.422830	-2.316375	0.689913
H	1.006060	-1.095490	1.827960
H	2.561513	-1.868914	1.559972
C	0.953895	-2.655541	0.346803
O	3.384155	-1.119606	-1.820110
O	4.344424	-0.650423	0.166150
O	1.942493	1.119505	1.994542
O	2.701684	1.875396	0.007181
C	-1.732337	4.205276	-0.391236
H	-0.496521	4.110144	1.375680
H	-2.983384	3.969716	-2.133941
C	1.313444	-4.087089	0.562878
C	-0.340005	-2.312355	-0.343100
C	5.617592	-1.000006	-0.401245
C	2.869215	3.178945	0.589175
H	-1.774854	5.291161	-0.406149
H	1.098841	-4.693548	-0.328204
H	2.375332	-4.208026	0.809167
H	0.738701	-4.541819	1.391151
H	-0.372261	-2.817690	-1.325181
H	-1.196935	-2.716106	0.219232
H	5.850018	-0.347711	-1.247751
H	6.343055	-0.863954	0.401369
H	5.611547	-2.038305	-0.745038
H	3.482573	3.121154	1.492156
H	3.360469	3.779499	-0.176920
H	1.892945	3.603389	0.838699

TS10A

C	-2.545179	0.258625	-0.305809
C	-3.784093	-0.523523	-0.598446
C	-1.465327	-0.310882	0.277435
C	-4.476667	-1.187174	0.426208
C	-4.306141	-0.568945	-1.901208
C	-0.247978	0.495946	0.689031
C	-2.619656	1.706951	-0.657260
C	-5.647056	-1.897116	0.152249
H	-4.095527	-1.126542	1.441848
C	-5.470276	-1.284640	-2.177977
H	-3.790706	-0.035399	-2.695543
H	0.431455	0.618407	-0.160729
H	-0.543914	1.499388	0.998688
C	0.587544	-0.164986	1.808710
C	-3.744961	2.460980	-0.279651
C	-1.608550	2.349602	-1.389343
C	-6.144181	-1.952691	-1.151466
H	-6.173647	-2.400911	0.958728
H	-5.855372	-1.317757	-3.193857
C	1.036240	-1.587462	1.391245
C	1.829442	0.707269	2.064800
C	-0.224928	-0.224679	3.109167
C	-3.838206	3.815140	-0.595594
H	-4.545289	1.974959	0.270845
C	-1.701699	3.704005	-1.710947

H	-0.745930	1.780032	-1.715307
H	-7.054771	-2.505703	-1.365995
H	1.306357	-2.158630	2.291056
H	1.970729	-1.504765	0.829987
C	0.010098	-2.349737	0.588955
O	2.980530	0.362479	1.915275
O	1.462004	1.945365	2.444979
O	-1.419056	-0.047389	3.208609
O	0.570696	-0.541125	4.152314
C	-2.815639	4.444089	-1.311607
H	-4.711663	4.381550	-0.283261
H	-0.904871	4.179990	-2.277359
C	0.155666	-3.845287	0.645654
C	-1.392399	-1.803137	0.556718
C	2.536100	2.887233	2.631002
C	-0.088471	-0.635922	5.427394
H	-2.889776	5.499297	-1.560052
H	-0.512466	-4.352481	-0.059032
H	1.184958	-4.153057	0.436074
H	-0.096504	-4.210814	1.655545
H	-2.001030	-2.363028	-0.160681
H	-1.846215	-2.003723	1.544616
H	3.039092	3.078527	1.678855
H	2.063779	3.797655	2.999554
H	3.261020	2.502666	3.352986
H	-0.855783	-1.414922	5.403465
H	0.694512	-0.887194	6.143044
H	-0.558112	0.317426	5.684451
C	0.538117	-2.041033	-1.684319
C	1.877382	-1.762297	-1.788200
H	-0.192979	-1.245025	-1.769409
H	0.175758	-3.043927	-1.869396
C	2.410508	-0.394130	-1.768887
C	1.691155	0.652130	-2.388233
C	3.599550	-0.065067	-1.087242
C	2.087751	1.975345	-2.260687
H	0.807847	0.415933	-2.973214
C	4.002230	1.256078	-0.951488
H	4.174278	-0.839866	-0.596422
C	3.239039	2.291997	-1.516331
H	1.511402	2.768683	-2.725999
H	4.887425	1.494001	-0.371885
C	3.614321	3.656931	-1.306040
N	3.910110	4.765105	-1.106836
C	2.851412	-2.902955	-1.718661
F	2.287779	-4.086215	-2.044793
F	3.915435	-2.718604	-2.528537
F	3.360222	-3.063968	-0.454248

INT10A

C	2.928946	-0.037897	-0.501196
C	3.954843	1.021246	-0.273179
C	1.751173	-0.081285	0.165768
C	4.415115	1.337758	1.015243
C	4.520368	1.699629	-1.366978
C	0.774512	-1.230327	-0.020878
C	3.302377	-1.057713	-1.527666
C	5.389656	2.317990	1.208545
H	4.013976	0.796949	1.867283
C	5.487630	2.684515	-1.175582

H	4.192589	1.446677	-2.371445	C	1.567281	-1.817051	4.472287
H	0.152895	-1.088374	-0.909358	H	4.347820	-3.686995	-4.215433
H	1.302776	-2.171142	-0.191384	H	-0.113837	3.264066	1.915853
C	-0.179500	-1.413127	1.182478	H	-1.637874	2.446517	2.285206
C	4.516800	-1.755399	-1.416758	H	-0.167298	2.009789	3.170731
C	2.485607	-1.312489	-2.639914	H	1.808826	1.944739	0.913300
C	5.925459	2.999095	0.114129	H	1.592814	0.726573	2.156782
H	5.735012	2.543667	2.214169	H	-2.669944	-4.648405	1.183240
H	5.903985	3.205000	-2.034070	H	-3.482957	-3.930357	2.616154
C	-0.931645	-0.099468	1.571935	H	-3.778190	-3.267730	0.973284
C	-1.244659	-2.453223	0.806856	H	2.586068	-2.003177	4.121678
C	0.652186	-2.037103	2.317461	H	1.559403	-1.069645	5.265930
C	4.886700	-2.700563	-2.371955	H	1.133890	-2.758358	4.819973
H	5.167995	-1.550477	-0.571487	C	-0.662458	1.712052	-0.287150
C	2.857722	-2.252280	-3.602317	C	-2.103444	2.109841	-0.452092
H	1.559502	-0.756988	-2.756287	H	-0.409789	0.944129	-1.020425
H	6.683961	3.762860	0.263103	H	-0.029772	2.571759	-0.525280
H	-1.064678	-0.100712	2.654698	C	-3.149714	1.207519	-0.816934
H	-1.941468	-0.121883	1.157221	C	-2.860704	-0.135181	-1.202545
C	-0.226481	1.217387	1.147676	C	-4.531606	1.576784	-0.784285
O	-1.475032	-2.848564	-0.315948	C	-3.855848	-1.049973	-1.489493
O	-1.956166	-2.815586	1.888581	H	-1.837621	-0.476215	-1.263271
O	1.181336	-3.122395	2.212963	C	-5.528467	0.666678	-1.079395
O	0.767124	-1.260485	3.412356	H	-4.818519	2.579815	-0.500854
C	4.057509	-2.953123	-3.468476	C	-5.209157	-0.663144	-1.427021
H	5.824306	-3.239241	-2.262835	H	-3.584955	-2.068419	-1.744959
H	2.211929	-2.432792	-4.457548	H	-6.569444	0.970165	-1.032755
C	-0.557138	2.301218	2.189022	C	-6.244513	-1.608953	-1.699536
C	1.303882	0.998746	1.132913	N	-7.087482	-2.383389	-1.913439
C	-3.043167	-3.728170	1.639620	C	-2.463579	3.551222	-0.195828

F	-1.381949	4.330604	0.013973
F	-3.142529	4.099210	-1.234593
F	-3.265661	3.695399	0.898477

INT11A

C	-1.647843	-1.468979	0.105504
C	-1.090602	-2.462821	1.075981
C	-1.184086	-0.201029	0.075247
C	-1.434176	-2.396311	2.434218
C	-0.238029	-3.489699	0.646322
C	-1.831720	0.893711	-0.739901
C	-2.697663	-1.994573	-0.808644
C	-0.943043	-3.334285	3.344405
H	-2.087850	-1.594693	2.769958
C	0.260544	-4.422651	1.555655
H	0.057898	-3.523099	-0.396144
H	-1.261410	1.114600	-1.645312
H	-2.838839	0.618615	-1.058163
C	-1.914573	2.193443	0.101318
C	-3.773822	-2.750839	-0.313121
C	-2.605254	-1.796350	-2.197166
C	-0.093245	-4.351799	2.905900
H	-1.219804	-3.267702	4.394261
H	0.934763	-5.202564	1.210407
C	-0.502646	2.693115	0.543639
C	-2.570898	3.294171	-0.730387
C	-2.876049	1.886393	1.255983
C	-4.747046	-3.259205	-1.171700
H	-3.840189	-2.935703	0.755798
C	-3.578465	-2.307258	-3.056918
H	-1.745238	-1.265286	-2.591609
H	0.297343	-5.080299	3.612426
H	-0.607389	3.198136	1.507249
H	-0.171768	3.462720	-0.165844
C	0.601559	1.597523	0.607743
O	-2.783666	3.261839	-1.921009
O	-2.839985	4.376166	0.041839
O	-4.071473	1.739954	1.104913
O	-2.264246	1.730471	2.448462
C	-4.656031	-3.035786	-2.548664
H	-5.577968	-3.832435	-0.766494
H	-3.486700	-2.144157	-4.128102
C	1.571181	1.955288	1.748152
C	0.000135	0.214477	0.931439
C	-3.434212	5.482301	-0.648465
C	-3.120234	1.307466	3.518156
H	-5.413301	-3.434946	-3.219344
H	2.401186	1.243884	1.789860
H	1.997111	2.956452	1.603830
H	1.053102	1.946048	2.716316
H	0.793089	-0.534266	0.835737
H	-0.302632	0.208807	1.984972
H	-4.391790	5.189961	-1.090018
H	-3.580647	6.256921	0.106052
H	-2.774556	5.840866	-1.444650
H	-3.603327	0.358758	3.265900
H	-2.468534	1.189936	4.384883
H	-3.894047	2.056114	3.712219
C	1.393989	1.597254	-0.762090
C	2.327328	0.446183	-1.042809

H	1.941327	2.550825	-0.792944
H	0.664758	1.678951	-1.572712
C	3.688114	0.423122	-0.663403
C	4.356898	1.561461	-0.086044
C	4.534242	-0.735867	-0.825351
C	5.689417	1.551008	0.264974
H	3.799530	2.477622	0.072239
C	5.862653	-0.742252	-0.468169
H	4.108162	-1.646017	-1.228555
C	6.493454	0.399806	0.089057
H	6.137764	2.448245	0.686883
H	6.446900	-1.649587	-0.606618
C	7.861177	0.388323	0.459108
N	8.991520	0.381061	0.764092
C	1.787923	-0.690319	-1.794794
F	0.517103	-0.473089	-2.270222
F	2.515846	-1.067089	-2.899669
F	1.681886	-1.892236	-1.082977

TS12A

C	3.183143	0.116919	-0.714212
C	4.033575	1.274367	-0.301738
C	1.867003	0.253469	-1.004772
C	3.705304	2.027662	0.837493
C	5.213579	1.590318	-0.993852
C	0.898440	-0.890598	-1.124492
C	3.895134	-1.193924	-0.717374
C	4.514267	3.086834	1.251995
H	2.809104	1.768471	1.392842
C	6.022864	2.649052	-0.582208
H	5.490316	0.996511	-1.861387
H	0.234396	-0.747260	-1.981965
H	1.362571	-1.869453	-1.242672
C	0.006019	-0.912390	0.163510
C	4.704365	-1.561295	0.372582
C	3.827176	-2.072870	-1.811416
C	5.674305	3.403795	0.541626
H	4.237220	3.661549	2.132202
H	6.927089	2.885851	-1.138376
C	-0.299776	0.521640	0.695709
C	-1.266573	-1.713198	-0.140485
C	0.787106	-1.786927	1.162151
C	5.384117	-2.778304	0.387159
H	4.789870	-0.879624	1.213870
C	4.506852	-3.291461	-1.800046
H	3.237639	-1.787254	-2.677847
H	6.305151	4.228877	0.863450
H	0.438520	0.728297	1.472806
H	-1.271174	0.612998	1.200095
C	-0.183037	1.681297	-0.324838
O	-1.613320	-2.089357	-1.240486
O	-1.938327	-1.958256	0.996043
O	0.786274	-3.001609	1.129083
O	1.551855	-1.088338	2.026067
C	5.284474	-3.653035	-0.698309
H	5.993339	-3.044819	1.247821
H	4.432156	-3.956641	-2.656861
C	-0.139839	2.968253	0.524768
C	1.152126	1.572783	-1.155506
C	-3.029666	-2.885096	0.911333

C	2.338152	-1.889339	2.918709	F	-2.495190	4.214051	-0.477788
H	5.813337	-4.602735	-0.687974	F	-4.382595	3.361592	0.143316
H	-0.129427	3.860654	-0.111464	F	-2.572861	2.205920	1.997096
H	-1.008811	2.973731	1.204588				
H	0.782976	2.977002	1.121833	**5**			
H	0.911875	1.665448	-2.227460	C	0.699757	1.686724	0.459437
H	1.808737	2.412861	-0.923479	C	-0.592428	2.132078	1.060565
H	-2.632378	-3.905410	0.954967	C	1.007443	0.383649	0.264406
H	-3.663999	-2.683390	1.774079	C	-1.070231	1.622733	2.279263
H	-3.589426	-2.741415	-0.011155	C	-1.364005	3.103704	0.398663
H	3.015759	-2.535424	2.354763	C	2.334999	-0.052645	-0.334103
H	2.899520	-1.178475	3.527445	C	1.625237	2.797388	0.080153
H	1.690402	-2.507696	3.547102	C	-2.296940	2.039143	2.799846
C	-1.376876	1.725072	-1.346895	H	-0.468305	0.899879	2.821766
C	-2.772547	1.892580	-0.783600	C	-2.591990	3.516924	0.913248
H	-1.359509	0.804606	-1.944058	H	-0.999755	3.518389	-0.536189
H	-1.162130	2.548783	-2.042319	H	2.345465	0.077136	-1.421076
C	-3.716366	0.765561	-0.597675	H	3.153159	0.558016	0.055091
C	-4.085648	-0.057488	-1.674537	C	2.629434	-1.543330	-0.067896
C	-4.311595	0.565610	0.668417	C	1.997543	3.746230	1.047628
C	-5.088587	-1.010270	-1.532068	C	2.112184	2.950006	-1.226725
H	-3.602097	0.067762	-2.639701	C	-3.066412	2.982665	2.115435
C	-5.318107	-0.380571	0.809220	H	-2.647981	1.630147	3.743637
H	-3.887828	1.184302	1.486526	H	-3.180845	4.253957	0.373967
C	-5.732604	-1.162274	-0.290302	C	1.510437	-2.433269	-0.678218
H	-5.383823	-1.632004	-2.372202	C	3.931132	-1.927074	-0.785516
H	-5.792327	-0.535697	1.775298	C	2.860640	-1.746894	1.437020
C	-6.770225	-2.134911	-0.137102	C	2.854634	4.796999	0.725902
N	-7.612357	-2.931002	-0.008719	H	1.610180	3.649526	2.058260
C	-3.180991	3.080910	-0.336413	C	2.965493	4.005811	-1.552067

H	1.803320	2.248668	-1.993288	H	2.138499	-2.294845	3.925551
H	-4.021966	3.306502	2.519089	H	2.005089	-4.046311	3.544185
H	1.615241	-3.446259	-0.285048	H	3.600719	-3.219236	3.507885
H	1.697937	-2.494994	-1.756720	C	-0.488402	-1.350496	-1.800999
C	0.062691	-1.888785	-0.435879	H	-0.760986	-2.216100	-2.420768
O	4.345320	-1.399368	-1.792936	H	0.322203	-0.845894	-2.331279
O	4.526468	-2.980526	-0.190893	C	-2.974586	-0.792398	-1.181589
O	3.501262	-0.976791	2.118239	C	-3.588734	-1.981362	-1.609845
O	2.289974	-2.868751	1.922756	C	-3.617147	-0.016486	-0.200440
C	3.343645	4.929780	-0.576716	C	-4.806805	-2.389118	-1.078833
H	3.139874	5.514181	1.491176	H	-3.105401	-2.593774	-2.364920
H	3.329754	4.107688	-2.571008	C	-4.834139	-0.415956	0.338580
C	-0.808707	-3.038577	0.103351	H	-3.152861	0.897686	0.151284
C	0.073322	-0.754645	0.614094	C	-5.438664	-1.607822	-0.095534
C	5.736574	-3.442066	-0.819184	H	-5.274875	-3.307779	-1.417658
C	2.528732	-3.116884	3.318898	H	-5.313668	0.186507	1.103320
H	4.009233	5.750763	-0.829700	C	-6.688422	-2.028720	0.464955
H	-1.823805	-2.707417	0.337723	N	-7.702286	-2.371099	0.921739
H	-0.879758	-3.853879	-0.627462	C	-1.655800	-0.384804	-1.726274
H	-0.362562	-3.445042	1.017844	C	-1.489954	0.871912	-2.142300
H	-0.946594	-0.395154	0.756810	F	-0.357316	1.371208	-2.631699
H	0.365551	-1.194078	1.577739	F	-2.430022	1.809191	-2.157525
H	6.483016	-2.643521	-0.837533				
H	6.080368	-4.278834	-0.210878				
H	5.534400	-3.764823	-1.844246				

2.5 Characterization Data of Products

Dimethyl-6-(2-(4-cyanophenyl)-3,3-difluoroallyl)-6-methyl-3,4-diphenylcyclohept-3-ene-1,1-dicarboxylate (4)

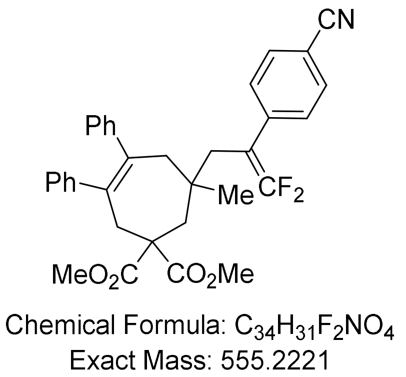

4 was prepared according to general procedure **2.1** using dimethyl 2-(2-methylallyl)-2-(3-phenylprop-2-yn-1-yl)malonate (0.2 mmol, 60.7 mg), bromobenzene (0.2 mmol, 31.4 mg) and 4-(3,3,3-trifluoroprop-1-en-2-yl)benzonitrile (0.1 mmol, 19.7 mg), and was purified by silica gel column chromatography (PE/EtOAc = 15/1) to obtain **4** as colorless oil (35.0 mg, 63% yield).

^{1}H NMR (600 MHz, $CDCl_3$) δ 7.63-7.58 (m, 2H), 7.37-7.33 (m, 2H), 7.07-7.03 (m, 5H), 7.02-6.99 (m, 1H), 6.93-6.88 (m, 2H), 6.87-6.82 (m, 2H), 3.70 (s, 3H), 3.58 (s, 3H), 3.34 (d, *J* = 15.4 Hz, 1H), 3.07 (d, *J* = 15.4 Hz, 1H), 2.56 (d, *J* = 14.4 Hz, 1H), 2.45 (ddd, *J* = 14.2, 2.8, 1.2 Hz, 1H), 2.40 (d, *J* = 2.9 Hz, 1H), 2.36 (d, *J* = 14.5 Hz, 1H), 2.27 (d, *J* = 14.5 Hz, 1H), 2.15 (d, *J* = 14.5 Hz, 1H), 0.86 (s, 3H);

^{19}F NMR (565 MHz, $CDCl_3$) δ -85.32 (d, *J* = 31.6 Hz), -88.12 (d, *J* = 31.2 Hz);

^{13}C NMR (151 MHz, $CDCl_3$) δ 172.7, 172.0, 154.8 (dd, *J* = 293.3, 291.1 Hz), 144.3, 143.7, 140.3 (dd, *J* = 5.0, 2.8 Hz), 138.5, 136.3, 132.3, 129.4, 129.21, 129.20 (t, *J* = 2.6 Hz), 127.8, 127.6, 126.0, 125.9, 118.6, 111.0, 89.6 (dd, *J* = 22.3, 13.1 Hz), 54.0, 53.0, 52.4, 46.5, 44.6, 42.1, 39.5, 36.5 (t, *J* = 2.2 Hz), 25.4;

HRMS: (ESI) calcd for $C_{34}H_{32}F_2NO_4^+$[M+H]$^+$ 556.2294; found 556.2306.

dimethyl 3-(2-(4-cyanophenyl)-3,3-difluoroallyl)-5-(diphenylmethylene)-3-methyl cyclohexane-1,1-dicarboxylate (5)

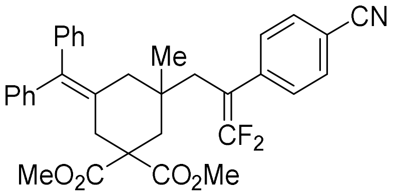

Chemical Formula: $C_{34}H_{31}F_2NO_4$
Exact Mass: 555.2221

5 was prepared according to general procedure **2.2** using dimethyl 2-(2-methylallyl)-2-(3-phenylprop-2-yn-1-yl)malonate (0.2 mmol, 60.6 mg), bromobenzene (0.2 mmol, 31.4 mg) and 4-(3,3,3-trifluoroprop-1-en-2-yl)benzonitrile (0.1 mmol, 19.7 mg), and was purified by silica gel column chromatography (PE/EtOAc = 10/1) to obtain **5** as colorless oil (39.4 mg, 71% yield);

^{1}H NMR (600 MHz, $CDCl_3$) δ 7.52-7.47 (m, 2H), 7.30-7.26 (m, 2H), 7.26-7.22 (m, 3H), 7.20-7.14 (m, 5H), 6.98-6.93 (m, 2H), 3.63 (s, 3H), 3.61 (s, 3H), 3.25 (dt, J = 14.3, 1.8 Hz, 1H), 2.42 (dd, J = 14.3, 2.6 Hz, 1H), 2.28 (dt, J = 14.3, 3.1 Hz, 1H), 2.20 (dt, J = 14.1, 2.1 Hz, 1H), 2.07 (dd, J = 14.3, 1.3 Hz, 1H), 1.93 (dd, J = 13.9, 3.3 Hz, 2H), 1.48 (d, J = 13.7 Hz, 1H), 0.71 (s, 3H);

^{19}F NMR (565 MHz, $CDCl_3$) δ -86.20 (d, J = 32.3 Hz), -88.72 (d, J = 32.8 Hz);

^{13}C NMR (151 MHz, $CDCl_3$) δ 172.5, 171.7, 154.5 (dd, J = 292.8, 290.5 Hz), 142.5, 142.0, 140.2, 132.2, 129.5, 129.1, 129.0 (t, J = 2.7 Hz), 128.7, 128.1, 127.9, 126.4, 126.2, 118.6, 110.8, 89.3 (dd, J = 22.6, 13.5 Hz), 54.7, 52.9, 52.3, 42.2, 42.0, 36.9 (t, J = 2.5 Hz), 34.7, 22.8;

HRMS: (ESI) calcd for $C_{34}H_{32}F_2NO_4^+$[M+H]$^+$ 556.2294; found 556.2306.

4-(3-(6,6-bis(methoxymethyl)-1-methyl-3,4-diphenylcyclohept-3-en-1-yl)-1,1-difluoroprop-1-en-2-yl)benzonitrile (6)

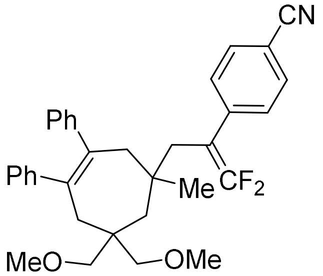

Chemical Formula: $C_{34}H_{35}F_2NO_2$
Exact Mass: 527.2636

6 was prepared according to general procedure **2.1** using (3,3-bis(methoxymethyl)-5-methylhex-5-en-1-yn-1-yl)benzene (0.2 mmol, 51.8 mg), bromobenzene (0.2 mmol, 31.4 mg) and 4-(3,3,3-trifluoroprop-1-en-2-yl)benzonitrile (0.1 mmol, 19.7 mg), and was purified by silica gel column chromatography (PE/EtOAc = 20/1) to obtain **6** as colorless oil (29.5 mg, 56% yield).

^{1}H NMR (600 MHz, $CDCl_3$) δ 7.66-7.60 (m, 2H), 7.43-7.39 (m, 2H), 7.08-6.96 (m, 6H), 6.95-6.91 (m, 2H), 6.88-6.83 (m, 2H), 3.40 (s, 2H), 3.26 (s, 3H), 3.20 (s, 3H), 3.06 (s, 2H), 2.71 (d, *J* = 11.3 Hz, 1H), 2.69 (d, *J* = 10.7 Hz, 1H), 2.51 (d, *J* = 14.5 Hz, 1H), 2.45 (d, *J* = 14.4 Hz, 1H), 2.39 (d, *J* = 14.3 Hz, 1H), 2.30 (d, *J* = 14.0 Hz, 1H), 1.65 (d, *J* = 14.1 Hz, 1H), 1.32 (d, *J* = 14.1 Hz, 1H), 0.99 (s, 3H);

^{19}F NMR (565 MHz, $CDCl_3$) δ -85.65 (d, *J* = 32.2 Hz), -88.60 (d, *J* = 32.5 Hz);

^{13}C NMR (151 MHz, $CDCl_3$) δ 154.7 (dd, *J* = 292.3, 290.7 Hz), 145.2, 144.8, 140.9 (dd, *J* = 5.2, 2.7 Hz), 138.5, 138.2, 132.2, 129.5, 129.4, 129.3 (t, *J* = 2.9 Hz), 127.6, 127.6, 125.7, 125.6, 118.7, 110.8, 90.0 (dd, *J* = 22.5, 12.8 Hz), 79.9, 74.9, 59.2, 58.7, 46.9, 46.3, 44.1, 41.3, 40.1, 37.8 (t, *J* = 2.1 Hz), 25.7;

HRMS: (ESI) calcd for $C_{34}H_{36}F_2NO_2^+$[M+H]$^+$ 528.2709; found 528.2700.

4-(1,1-difluoro-3-(3-methyl-5,6-diphenyl-1-tosyl-2,3,4,7-tetrahydro-1*H*-azepin-3-yl)prop-1-en-2-yl)benzonitrile (7)

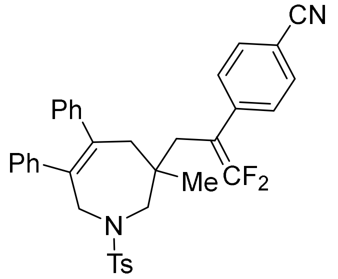

Chemical Formula: $C_{36}H_{32}F_2N_2O_2S$
Exact Mass: 594.2153

7 was prepared according to general procedure **2.1** using 4-methyl-*N*-(2-methylallyl)-*N*-(3-phenylprop-2-yn-1-yl)benzenesulfonamide (0.2 mmol, 67.9 mg), bromobenzene (0.2 mmol, 31.4 mg) and 4-(3,3,3-trifluoroprop-1-en-2-yl)benzonitrile (0.1 mmol, 19.7 mg), and was purified by silica gel column chromatography (PE/EtOAc = 10/1) to obtain **7** as colorless oil (44.6 mg, 75% yield).

^{1}H NMR (600 MHz, CDCl$_3$) δ 7.68-7.61 (m, 2H), 7.49-7.44 (m, 2H), 7.44-7.38 (m, 2H), 7.29-7.26 (m, 2H), 7.13-6.98 (m, 8H), 6.88-6.81 (m, 2H), 4.26 (d, *J* = 15.4 Hz, 1H), 3.81 (d, *J* = 15.4 Hz, 1H), 3.26 (d, *J* = 12.5 Hz, 1H), 2.69 (d, *J* = 13.8 Hz, 2H), 2.52 (d, *J* = 14.5 Hz, 1H), 2.49-2.41 (m, 5H), 0.94 (s, 3H);

^{19}F NMR (565 MHz, CDCl$_3$) δ -84.68 (d, *J* = 30.4 Hz), -87.53 (d, *J* = 30.4 Hz);

^{13}C NMR (151 MHz, CDCl$_3$) δ 154.7 (dd, *J* = 293.6, 291.3 Hz), 144.2, 143.5, 141.6, 140.1 (dd, *J* = 4.9, 2.9 Hz), 139.3, 135.5, 134.8, 132.3, 129.7, 129.5, 129.2 (t, *J* = 2.5 Hz), 128.9, 127.9, 127.8, 127.1, 126.5, 126.2, 118.5, 111.1, 89.0 (dd, *J* = 22.5, 13.7 Hz), 61.1, 54.7, 46.7, 38.8 (t, *J* = 2.2 Hz), 38.1, 22.5, 21.5;

HRMS: (ESI) calcd for $C_{36}H_{33}F_2N_2O_2S^+$[M+H]$^+$ 595.2225; found 595.2233.

4-(1,1-difluoro-3-(5-(4-methoxyphenyl)-3-methyl-6-phenyl-1-tosyl-2,3,4,7-tetrahydro-1*H*-azepin-3-yl)prop-1-en-2-yl)benzonitrile (8)

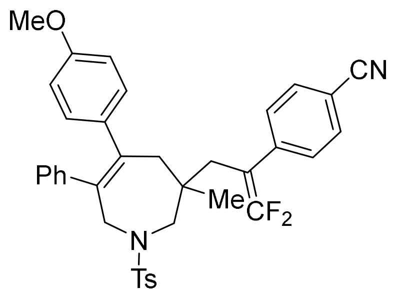

Chemical Formula: $C_{37}H_{34}F_2N_2O_3S$
Exact Mass: 624.2258

8 was prepared according to general procedure **2.1** using *N*-(3-(4-methoxyphenyl)prop-2-yn-1-yl)-4-methyl-*N*-(2-methylallyl)benzenesulfonamide (0.2 mmol, 73.9 mg), bromobenzene (0.2 mmol, 31.4 mg) and 4-(3,3,3-trifluoroprop-1-en-2-yl)benzonitrile (0.1 mmol, 19.7 mg), and was purified by silica gel column chromatography (PE/EtOAc = 5/1) to obtain **8** as white solid (43.1 mg, 69% yield).

^{1}H NMR (600 MHz, CDCl$_3$) δ 7.65 (d, *J* = 8.5 Hz, 2H), 7.46 (d, *J* = 8.2 Hz, 2H), 7.43-7.39 (m, 2H), 7.28-7.26 (m, 2H), 7.10-7.03 (m, 3H), 7.03-6.98 (m, 2H), 6.79-6.74 (m, 2H), 6.64-6.59 (m, 2H), 4.24 (d, *J* = 15.3 Hz, 1H), 3.80 (d, *J* = 15.3 Hz, 1H), 3.72 (s, 3H), 3.25 (d, *J* = 12.5 Hz, 1H), 2.68 (d, *J* = 12.6 Hz, 1H), 2.65 (d, *J* = 13.9 Hz, 1H), 2.51 (d, *J* = 14.6 Hz, 1H), 2.48-2.38 (m, 5H), 0.93 (s, 3H);

^{19}F NMR (565 MHz, $CDCl_3$) δ -84.68 (d, J = 30.3 Hz), -87.56 (d, J = 30.4 Hz);

^{13}C NMR (151 MHz, $CDCl_3$) δ 157.9, 154.8 (dd, J = 293.7, 291.5 Hz), 143.5, 141.9, 140.2 (dd, J = 5.0, 2.9 Hz), 138.9, 136.5, 134.8, 134.7, 132.3, 130.1, 129.7, 129.5, 129.2 (t, J = 2.5 Hz), 127.9, 127.1, 126.3, 118.5, 113.3, 111.1, 89.1 (dd, J = 22.6, 13.5 Hz), 61.1, 55.1, 54.7, 46.8, 38.7 (t, J = 2.2 Hz), 38.1, 22.5, 21.5;

HRMS: (ESI) calcd for $C_{37}H_{35}F_2N_2O_3S^+$[M+H]$^+$ 625.2331; found 625.2336.

4-(1,1-difluoro-3-(3-methyl-6-phenyl-1-tosyl-5-(4-(trifluoromethyl)phenyl)-2,3,4,7-tetrahydro-1*H*-azepin-3-yl)prop-1-en-2-yl)benzonitrile (9)

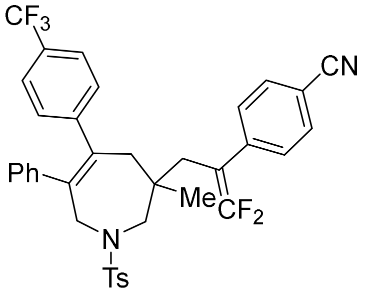

Chemical Formula: $C_{37}H_{31}F_5N_2O_2S$
Exact Mass: 662.2026

9 was prepared according to general procedure **2.1** using 4-methyl-*N*-(2-methylallyl)-*N*-(3-(4-(trifluoromethyl)phenyl)prop-2-yn-1-yl)benzenesulfonamide (0.2 mmol, 81.4 mg), bromobenzene (0.2 mmol, 31.4 mg) and 4-(3,3,3-trifluoroprop-1-en-2-yl)benzonitrile (0.1 mmol, 19.7 mg), and was purified by silica gel column chromatography (PE/EtOAc = 20/1) to obtain **9** as white solid (42.4 mg, 64% yield).

^{1}H NMR (600 MHz, $CDCl_3$) δ 7.66 (d, J = 8.4 Hz, 2H), 7.45 (d, J = 8.3 Hz, 2H), 7.44-7.40 (m, 2H), 7.34 (d, J = 8.1 Hz, 2H), 7.27 (d, J = 8.1 Hz, 2H), 7.13-7.06 (m, 3H), 7.06-7.02 (m, 2H), 6.97 (d, J = 8.0 Hz, 2H), 4.28 (d, J = 15.5 Hz, 1H), 3.80 (d, J = 15.6 Hz, 1H), 3.27 (d, J = 12.5 Hz, 1H), 2.74 (d, J = 14.0 Hz, 1H), 2.67 (d, J = 12.6 Hz, 1H), 2.52 (d, J = 14.6 Hz, 1H), 2.49-2.44 (m, 4H), 2.42 (d, J = 14.1 Hz, 1H), 0.94 (s, 3H);

^{19}F NMR (565 MHz, $CDCl_3$) δ -62.47, -84.49 (d, J = 30.0 Hz), -87.27 (d, J = 30.0 Hz);

^{13}C NMR (151 MHz, $CDCl_3$) δ 154.8 (dd, J = 294.2, 291.3 Hz), 148.1, 143.7, 140.9, 140.0 (dd, J = 4.6, 2.9 Hz), 137.9, 137.5, 134.7, 132.4, 129.8, 129.5, 129.22, 129.1 (d, J = 2.5 Hz), 128.3 (q, J = 32.2 Hz), 128.1, 127.1, 127.0, 125.0 (q, J = 3.6 Hz), 124.1 (q,

J = 272.1 Hz), 118.5, 111.2, 88.9 (dd, J = 22.4, 13.7 Hz), 61.3, 54.8, 46.7, 38.9 (t, J = 2.6 Hz), 38.3, 22.5, 21.6;

HRMS: (ESI) calcd for $C_{37}H_{32}F_5N_2O_2S^+[M+H]^+$ 663.2099; found 663.2103.

4-(6-(2-(4-cyanophenyl)-3,3-difluoroallyl)-6-methyl-3-phenyl-1-tosyl-2,5,6,7-tetrahydro-1*H*-azepin-4-yl)benzonitrile (10)

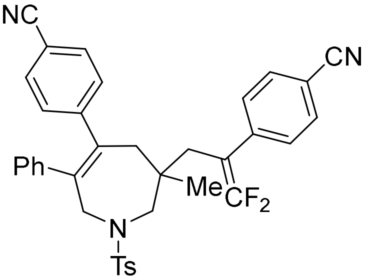

Chemical Formula: $C_{37}H_{31}F_2N_3O_2S$
Exact Mass: 619.2105

10 was prepared according to general procedure **2.1** using *N*-(3-(4-cyanophenyl)prop-2-yn-1-yl)-4-methyl-*N*-(2-methylallyl)benzenesulfonamide (0.2 mmol, 72.8 mg), bromobenzene (0.2 mmol, 31.4 mg) and 4-(3,3,3-trifluoroprop-1-en-2-yl)benzonitrile (0.1 mmol, 19.7 mg), and was purified by silica gel column chromatography (PE/EtOAc = 3/1) to obtain **10** as white solid (30.4 mg, 49% yield).

^{1}H NMR (600 MHz, $CDCl_3$) δ 7.70-7.64 (m, 2H), 7.47-7.40 (m, 4H), 7.40-7.35 (m, 2H), 7.28-7.26 (m, 2H), 7.14-7.07 (m, 3H), 7.06-7.00 (m, 2H), 7.00-6.93 (m, 2H), 4.27 (d, J = 15.6 Hz, 1H), 3.79 (d, J = 15.7 Hz, 1H), 3.27 (d, J = 12.6 Hz, 1H), 2.75 (d, J = 14.1 Hz, 1H), 2.64 (d, J = 12.7 Hz, 1H), 2.52 (d, J = 14.6 Hz, 1H), 2.49-2.43 (m, 4H), 2.39 (d, J = 14.0 Hz, 1H), 0.93 (s, 3H);

^{19}F NMR (565 MHz, $CDCl_3$) δ -84.37 (d, J = 29.8 Hz), -87.12 (d, J = 29.8 Hz);

^{13}C NMR (151 MHz, $CDCl_3$) δ 154.8 (dd, J = 294.4, 291.3 Hz), 149.3, 143.7, 140.6, 139.9 (dd, J = 4.8, 2.8 Hz), 138.4, 137.5, 134.6, 132.4, 131.8, 129.8, 129.6, 129.4, 129.2 (t, J = 2.5 Hz), 128.2, 127.2, 127.0, 118.7, 118.4, 111.2, 109.9, 88.8 (dd, J = 22.4, 13.6 Hz), 61.3, 54.7, 46.5, 38.8 (t, J = 2.6 Hz), 38.3, 22.4, 21.5;

HRMS: (ESI) calcd for $C_{37}H_{32}F_2N_3O_2S^+[M+H]^+$ 620.2178; found 620.2190.

4-(3-(5-(4-chlorophenyl)-3-methyl-6-phenyl-1-tosyl-2,3,4,7-tetrahydro-1*H*-

azepin-3-yl)-1,1-difluoroprop-1-en-2-yl)benzonitrile (11)

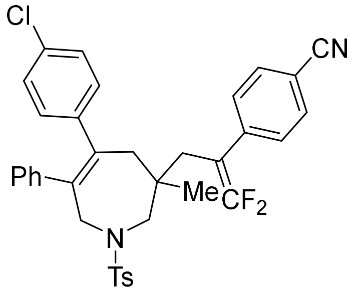

Chemical Formula: $C_{36}H_{31}ClF_2N_2O_2S$
Exact Mass: 628.1763

11 was prepared according to general procedure **2.1** using *N*-(3-(4-chlorophenyl)prop-2-yn-1-yl)-4-methyl-*N*-(2-methylallyl)benzenesulfonamide (0.2 mmol, 74.8 mg), bromobenzene (0.2 mmol, 31.4 mg) and 4-(3,3,3-trifluoroprop-1-en-2-yl)benzonitrile (0.1 mmol, 19.7 mg), and was purified by silica gel column chromatography (PE/EtOAc = 10/1) to obtain **11** as white solid (42.8 mg, 68% yield).

^{1}H NMR (600 MHz, $CDCl_3$) δ 7.69-7.63 (m, 2H), 7.48-7.43 (m, 2H), 7.43-7.39 (m, 2H), 7.29-7.26 (m, 2H), 7.12-7.07 (m, 3H), 7.07-7.04 (m, 2H), 7.04-7.00 (m, 2H), 6.81-6.75 (m, 2H), 4.25 (d, J = 15.4 Hz, 1H), 3.79 (d, J = 15.5 Hz, 1H), 3.26 (d, J = 12.6 Hz, 1H), 2.71-2.63 (m, 2H), 2.51 (d, J = 14.6 Hz, 1H), 2.48-2.42 (m, 4H), 2.39 (d, J = 13.9 Hz, 1H), 0.93 (s, 3H);

^{19}F NMR (565 MHz, $CDCl_3$) δ -84.55 (d, J = 30.2 Hz), -87.36 (d, J = 30.1 Hz);

^{13}C NMR (151 MHz, $CDCl_3$) δ 154.8 (dd, J = 293.9, 291.3 Hz), 143.6, 142.7, 141.2, 140.1 (dd, J = 4.6, 2.9 Hz), 138.0, 136.4, 134.7, 132.4, 132.1, 130.2, 129.8, 129.4, 129.2 (t, J = 2.5 Hz), 128.2, 128.1, 127.1, 126.8, 118.5, 111.2, 89.0 (dd, J = 22.4, 13.5 Hz), 61.2, 54.7, 46.7, 38.8 (t, J = 2.1 Hz), 38.2, 22.5, 21.5;

HRMS: (ESI) calcd for $C_{36}H_{32}ClF_2N_2O_2S^+$ $[M+H]^+$ 629.1836; found 629.1841.

4-(1,1-difluoro-3-(3-methyl-5-((8*R*,9*S*,13*S*,14*S*)-13-methyl-17-oxo-7,8,9,11,12,13,14,15,16,17-decahydro-6*H*-cyclopenta[*a*]phenanthren-3-yl)-6-phenyl-1-tosyl-2,3,4,7-tetrahydro-1*H*-azepin-3-yl)prop-1-en-2-yl)benzonitrile (12)

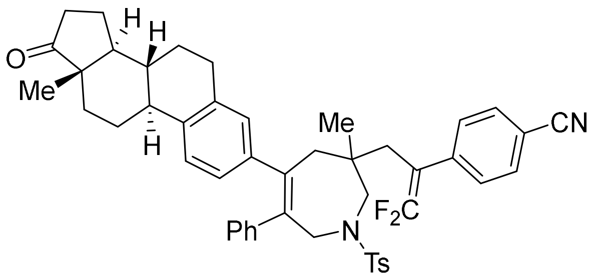

Chemical Formula: $C_{48}H_{48}F_2N_2O_3S$
Exact Mass: 770.3354

12 was prepared according to general procedure **2.1** using 4-methyl-*N*-(3-((8*R*,9*S*,13*S*,14*S*)-13-methyl-17-oxo-7,8,9,11,12,13,14,15,16,17-decahydro-6*H*-cyclo penta[*a*]phenanthren-3-yl)prop-2-yn-1-yl)-*N*-(2-methylallyl)benzenesulfonamide (0.2 mmol, 103.1 mg), bromobenzene (0.2 mmol, 31.4 mg) and 4-(3,3,3-trifluoroprop-1-en-2-yl)benzonitrile (0.1 mmol, 19.7 mg), and was purified by silica gel column chromatography (PE/EtOAc = 3/1) to obtain **12** as white solid (54.7 mg, 71% yield, d.r. = 1/1);

^{1}H NMR (600 MHz, DMSO-*d*$_6$) δ 7.93-7.85 (m, 2H), 7.65-7.57 (m, 2H), 7.40 (dd, *J* = 8.2, 4.4 Hz, 2H), 7.35 (d, *J* = 7.9 Hz, 2H), 7.11 (dd, *J* = 8.2, 6.4 Hz, 2H), 7.07 (qd, *J* = 6.6, 5.9, 3.3 Hz, 3H), 6.97 (dd, *J* = 12.6, 8.2 Hz, 1H), 6.66-6.52 (m, 2H), 4.25-4.15 (m, 1H), 3.83-3.72 (m, 1H), 3.17-3.06 (m, 1H), 2.85 (dd, *J* = 17.4, 14.3 Hz, 1H), 2.67-2.63 (m, 1H), 2.62-2.53 (m, 2H), 2.52-2.48 (m, 2H), 2.47-2.42 (m, 1H), 2.41 (s, 3H), 2.37-2.32 (m, 1H), 2.29-2.22 (m, 1H), 2.18-2.09 (m, 1H), 2.05 (dt, *J* = 18.4, 8.9 Hz, 1H), 1.98-1.83 (m, 2H), 1.74-1.70 (m, 1H), 1.57-1.49 (m, 1H), 1.49-1.40 (m, 2H), 1.38-1.25 (m, 3H), 0.86 (d, *J* = 6.9 Hz, 3H), 0.80 (d, *J* = 4.5 Hz, 3H);

^{19}F NMR (376 MHz, DMSO-*d*$_6$) δ -85.89 (d, *J* = 33.6 Hz), -88.46 (dd, *J* = 33.7, 9.1 Hz);

^{13}C NMR (151 MHz, DMSO-*d*$_6$) δ 220.02, 220.01, 154.5 (dd, *J* = 290.1, 289.9 Hz), 143.7, 142.60, 142.60, 141.75, 141.72, 140.1 (dd, *J* = 4.6, 2.9 Hz), 137.8, 135.98, 135.96, 135.6, 135.0, 133.0, 130.3, 129.0 (t, *J* = 2.5 Hz), 129.5, 129.4, 129.3, 128.3, 126.8, 126.73, 126.71, 125.10, 125.03, 119.1, 110.7, 89.9 (dd, *J* = 22.1, 12.8 Hz), 61.70, 61.63, 54.59, 54.55, 50.1, 47.7, 46.8, 46.7, 44.12, 44.10, 38.3, 38.0, 35.8, 31.8, 29.3, 29.2, 26.4, 25.6, 25.5, 22.6, 22.4, 21.6, 21.5, 14.0;

HRMS: (ESI) calcd for $C_{48}H_{49}F_2N_2O_3S^+$[M+H]$^+$ 771.3427; found 771.3427.

4-(1,1-difluoro-3-(3,5,6-triphenyl-1-tosyl-2,3,4,7-tetrahydro-1*H*-azepin-3-yl)prop-1-en-2-yl)benzonitrile (13)

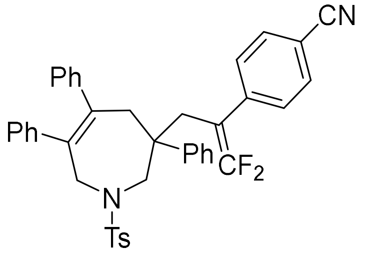

Chemical Formula: $C_{41}H_{34}F_2N_2O_2S$
Exact Mass: 656.2309

13 was prepared according to general procedure **2.1** using 4-methyl-*N*-(2-phenylallyl)-*N*-(3-phenylprop-2-yn-1-yl)benzenesulfonamide (0.2 mmol, 80.2 mg), bromobenzene (0.2 mmol, 31.4 mg) and 4-(3,3,3-trifluoroprop-1-en-2-yl)benzonitrile (0.1 mmol, 19.7 mg), and was purified by silica gel column chromatography (PE/EtOAc = 10/1) to obtain **13** as white solid (25.0 mg, 38% yield).

^{1}H NMR (600 MHz, $CDCl_3$) δ 7.59-7.54 (m, 2H), 7.43-7.38 (m, 2H), 7.33 (d, *J* = 8.1 Hz, 2H), 7.05-6.92 (m, 11H), 6.89 (dd, *J* = 8.1, 6.6 Hz, 2H), 6.87-6.82 (m, 2H), 6.55-6.48 (m, 2H), 4.14 (d, *J* = 15.5 Hz, 1H), 4.05 (d, *J* = 13.0 Hz, 1H), 4.00 (d, *J* = 15.5 Hz, 1H), 3.22 (d, *J* = 13.0 Hz, 1H), 3.16 (d, *J* = 14.2 Hz, 1H), 3.07 (d, *J* = 14.2 Hz, 1H), 2.93 (dt, *J* = 14.6, 2.4 Hz, 1H), 2.82 (dt, *J* = 14.7, 1.8 Hz, 1H), 2.47 (s, 3H);

^{19}F NMR (565 MHz, $CDCl_3$) δ -85.63 (d, *J* = 30.4 Hz), -87.81 (d, *J* = 30.6 Hz);

^{13}C NMR (151 MHz, $CDCl_3$) δ 154.5 (dd, *J* = 294.4, 291.3 Hz), 143.9, 143.8, 141.3, 140.9, 139.2 (dd, *J* = 4.9, 2.6 Hz), 138.9, 135.2, 134.1, 131.7, 129.8, 129.3, 129.2 (t, *J* = 2.3 Hz), 128.9, 127.7, 127.6, 127.6, 127.5, 126.4, 126.4, 126.1, 118.6, 110.4, 89.0 (dd, *J* = 21.8, 14.7 Hz), 58.8, 54.8, 45.8 (t, *J* = 2.7 Hz), 44.8, 40.8, 21.6;

HRMS: (ESI) calcd for $C_{41}H_{35}F_2N_2O_2S^+$[M+H]$^+$ 657.2382; found 657.2382.

4-(1,1-difluoro-3-(6-(4-methoxyphenyl)-3-methyl-5-phenyl-1-tosyl-2,3,4,7-tetrahydro-1*H*-azepin-3-yl)prop-1-en-2-yl)benzonitrile (14)

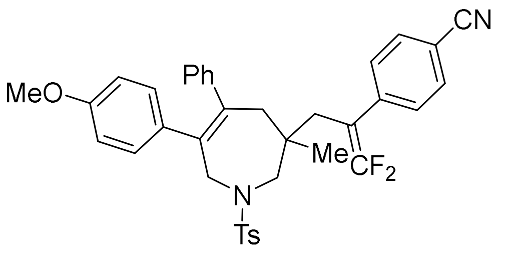

Chemical Formula: $C_{37}H_{34}F_2N_2O_3S$
Exact Mass: 624.2258

14 was prepared according to general procedure **2.1** using 4-methyl-*N*-(2-methylallyl)-*N*-(3-phenylprop-2-yn-1-yl)benzenesulfonamide (0.2 mmol, 67.9 mg), 1-bromo-4-methoxybenzene (0.2 mmol, 37.4 mg) and 4-(3,3,3-trifluoroprop-1-en-2-yl)benzonitrile (0.1 mmol, 19.7 mg), and was purified by silica gel column chromatography (PE/EtOAc = 5/1) to obtain **14** as white solid (43.1 mg, 69% yield).

^{1}H NMR (600 MHz, $CDCl_3$) δ 7.67-7.62 (m, 2H), 7.49-7.44 (m, 2H), 7.42-7.38 (m, 2H), 7.30-7.26 (m, 2H), 7.12-7.08 (m, 2H), 7.08-7.04 (m, 1H), 7.00-6.95 (m, 2H), 6.88-6.83 (m, 2H), 6.64-6.58 (m, 2H), 4.24 (d, *J* = 15.4 Hz, 1H), 3.77 (d, *J* = 15.4 Hz, 1H), 3.71 (s, 3H), 3.25 (d, *J* = 12.5 Hz, 1H), 2.70-2.62 (m, 2H), 2.52 (d, *J* = 14.6 Hz, 1H), 2.48-2.41 (m, 5H), 0.93 (s, 3H);

^{19}NMR (376 MHz, $CDCl_3$) δ -84.54 (d, *J* = 30.3 Hz), -87.44 (d, *J* = 30.2 Hz);

^{13}C NMR (151 MHz, $CDCl_3$) δ 158.0, 154.7 (dd, *J* = 293.8, 291.2 Hz), 144.5, 143.5, 140.1 (dd, *J* = 4.9, 2.9 Hz), 138.4, 135.1, 134.8, 133.9, 132.3, 130.7, 129.7, 129.2 (t, *J* = 2.5 Hz), 128.9, 128.0, 127.1, 126.0, 118.5, 113.2, 111.0, 89.0 (dd, *J* = 22.4, 13.5 Hz), 61.3, 55.0, 54.8, 46.8, 38.7 (t, *J* = 2.0 Hz), 38.1, 22.4, 21.5;

HRMS: (ESI) calcd for $C_{37}H_{35}F_2N_2O_3S^+$[M+H]$^+$ 625.2331; found 625.2331.

4-(3-(6-([1,1'-biphenyl]-4-yl)-3-methyl-5-phenyl-1-tosyl-2,3,4,7-tetrahydro-1*H*-azepin-3-yl)-1,1-difluoroprop-1-en-2-yl)benzonitrile (15)

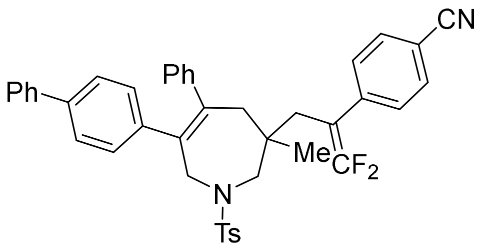

Chemical Formula: $C_{42}H_{36}F_2N_2O_2S$
Exact Mass: 670.2466

15 was prepared according to general procedure **2.1** using 4-methyl-*N*-(2-methylallyl)-*N*-(3-phenylprop-2-yn-1-yl)benzenesulfonamide (0.2 mmol, 67.9 mg), 4-bromo-1,1'-biphenyl (0.2 mmol, 46.6 mg) and 4-(3,3,3-trifluoroprop-1-en-2-yl)benzonitrile (0.1 mmol, 19.7 mg), and was purified by silica gel column chromatography (PE/EtOAc = 10/1) to obtain **15** as white solid (55.0 mg, 82% yield).

^{1}H NMR (600 MHz, $CDCl_3$) δ 7.70-7.63 (m, 2H), 7.54-7.47 (m, 4H), 7.44-7.36 (m, 4H), 7.35-7.27 (m, 5H), 7.15-7.07 (m, 5H), 6.93-6.88 (m, 2H), 4.32 (d, *J* = 15.4 Hz, 1H), 3.83 (d, *J* = 15.4 Hz, 1H), 3.29 (d, *J* = 12.5 Hz, 1H), 2.72 (d, *J* = 14.0 Hz, 1H), 2.69 (d, *J* = 12.9 Hz, 1H), 2.54 (d, *J* = 14.6 Hz, 1H), 2.51-2.44 (m, 5H), 0.97 (s, 3H);

^{19}F NMR (565 MHz, $CDCl_3$) δ -84.60 (d, *J* = 30.4 Hz), -87.47 (d, *J* = 30.3 Hz);

^{13}C NMR (151 MHz, $CDCl_3$) δ 154.8 (dd, *J* = 293.8, 291.3 Hz), 144.3, 143.6, 140.6, 140.5, 140.2 (dd, *J* = 5.3, 2.8 Hz), 139.7, 139.0, 135.2, 134.9, 132.4, 130.0, 129.8, 129.3 (t, *J* = 2.3 Hz), 128.9, 128.7, 128.1, 127.3, 127.2, 126.9, 126.5, 126.4, 118.6, 111.1, 89.1 (dd, *J* = 22.4, 13.6 Hz), 61.4, 54.6, 47.0, 38.7 (t, *J* = 2.0 Hz), 38.2, 22.5, 21.6;

HRMS: (ESI) calcd for $C_{42}H_{37}F_2N_2O_2S^+[M+H]^+$ 671.2538; found 671.2564.

4-(3-(6-(4-(dimethylamino)phenyl)-3-methyl-5-phenyl-1-tosyl-2,3,4,7-tetrahydro-1*H*-azepin-3-yl)-1,1-difluoroprop-1-en-2-yl)benzonitrile (16)

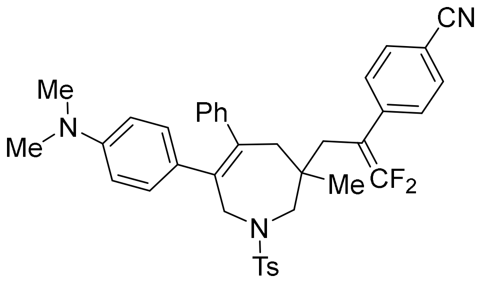

Chemical Formula: $C_{38}H_{37}F_2N_3O_2S$
Exact Mass: 637.2575

16 was prepared according to general procedure **2.1** using 4-methyl-*N*-(2-methylallyl)-*N*-(3-phenylprop-2-yn-1-yl)benzenesulfonamide (0.2 mmol, 67.9 mg), 4-bromo-*N*,*N*-dimethylaniline (0.2 mmol, 40.0 mg) and 4-(3,3,3-trifluoroprop-1-en-2-yl)benzonitrile (0.1 mmol, 19.7 mg), and was purified by silica gel column chromatography (PE/EtOAc = 3/1) to obtain **16** as white solid (43.4 mg, 68% yield).

^{1}H NMR (600 MHz, $CDCl_3$) δ 7.66-7.62 (m, 2H), 7.50-7.45 (m, 2H), 7.43-7.38 (m, 2H), 7.30-7.26 (m, 2H), 7.14-7.09 (m, 2H), 7.07-7.03 (m, 1H), 6.95-6.87 (m, 4H), 6.47-6.41 (m, 2H), 4.26 (d, *J* = 15.4 Hz, 1H), 3.76 (d, *J* = 15.4 Hz, 1H), 3.24 (d, *J* = 12.4 Hz, 1H), 2.86 (s, 6H), 2.69-2.58 (m, 2H), 2.51 (d, *J* = 14.4 Hz, 1H), 2.47-2.41 (m, 5H), 0.92 (s, 3H);

^{19}F NMR (376 MHz, $CDCl_3$) δ -84.61 (d, *J* = 30.9 Hz), -87.55 (d, *J* = 30.3 Hz);

^{13}C NMR (151 MHz, $CDCl_3$) δ 154.7 (dd, *J* = 293.7, 291.1 Hz), 148.8, 145.0, 143.3, 140.1 (dd, *J* = 5.0, 2.8 Hz), 136.8, 135.3, 134.8, 132.2, 130.4, 129.6, 129.3, 129.2 (t, *J* = 2.3 Hz), 128.9, 127.9, 127.1, 125.7, 111.6, 110.9, 89.1 (dd, *J* = 22.5, 13.4 Hz), 61.1, 54.8, 46.8, 40.3, 38.7 (t, *J* = 2.6 Hz), 38.0, 22.4, 21.5;

HRMS: (ESI) calcd for $C_{38}H_{38}F_2N_3O_2S^+[M+H]^+$ 638.2647; found 638.2639.

4-(3-(6-(4-chlorophenyl)-3-methyl-5-phenyl-1-tosyl-2,3,4,7-tetrahydro-1*H*-azepin-3-yl)-1,1-difluoroprop-1-en-2-yl)benzonitrile (17)

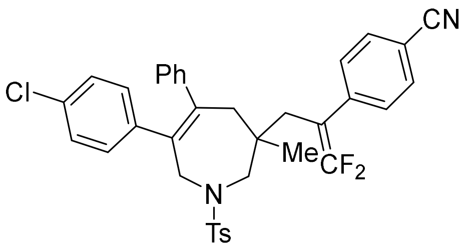

Chemical Formula: $C_{36}H_{31}ClF_2N_2O_2S$
Exact Mass: 628.1763

17 was prepared according to general procedure **2.1** using 4-methyl-*N*-(2-methylallyl)-*N*-(3-phenylprop-2-yn-1-yl)benzenesulfonamide (0.2 mmol, 67.9 mg), 1-bromo-4-chlorobenzene (0.2 mmol, 38.2 mg) and 4-(3,3,3-trifluoroprop-1-en-2-yl)benzonitrile (0.1 mmol, 19.7 mg), and was purified by silica gel column chromatography (PE/EtOAc = 10/1) to obtain **17** as white solid (34.6 mg, 55% yield).

^{1}H NMR (400 MHz, $CDCl_3$) δ 7.68-7.61 (m, 2H), 7.47-7.37 (m, 4H), 7.27 (d, *J* = 8.3 Hz, 2H), 7.11 (pd, *J* = 4.9, 1.7 Hz, 3H), 7.06-6.96 (m, 4H), 6.88-6.80 (m, 2H), 4.22 (d, *J* = 15.3 Hz, 1H), 3.75 (d, *J* = 15.4 Hz, 1H), 3.27 (d, *J* = 12.5 Hz, 1H), 2.69 (d, *J* = 14.0 Hz, 1H), 2.63 (d, *J* = 12.5 Hz, 1H), 2.52 (d, *J* = 14.6 Hz, 1H), 2.47-2.39 (m, 5H), 0.94 (s, 3H);

^{19}F NMR (376 MHz, $CDCl_3$) δ -84.39 (d, *J* = 30.1 Hz), -87.25 (d, *J* = 30.0 Hz);

^{13}C NMR (151 MHz, $CDCl_3$) δ 154.7 (dd, *J* = 294.0, 291.2 Hz), 143.9, 143.6, 140.3, 140.0 (dd, *J* = 4.8, 2.8 Hz), 140.0, 134.7, 134.6, 132.3, 132.3, 130.8, 129.7, 129.2 (t, *J* = 2.5 Hz), 128.7, 128.1, 128.0, 127.0, 126.5, 118.5, 111.1, 88.9 (dd, *J* = 22.4, 13.6 Hz), 61.6, 54.4, 47.0, 38.5 (t, *J* = 2.3 Hz), 38.2, 22.4, 21.5;

HRMS: (ESI) calcd for $C_{36}H_{32}Cl_1F_2N_2O_2S^+$[M+H]$^+$ 629.1836; found 629.1844.

4-(1,1-difluoro-3-(6-(4-fluorophenyl)-3-methyl-5-phenyl-1-tosyl-2,3,4,7-tetrahydro-1*H*-azepin-3-yl)prop-1-en-2-yl)benzonitrile (18)

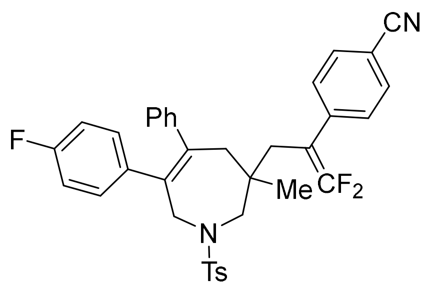

Chemical Formula: $C_{36}H_{31}F_3N_2O_2S$
Exact Mass: 612.2058

18 was prepared according to general procedure **2.1** using 4-methyl-*N*-(2-methylallyl)-*N*-(3-phenylprop-2-yn-1-yl)benzenesulfonamide (0.2 mmol, 67.9 mg), 1-bromo-4-fluorobenzene (0.2 mmol, 35.0 mg) and 4-(3,3,3-trifluoroprop-1-en-2-yl)benzonitrile (0.1 mmol, 19.7 mg), and was purified by silica gel column chromatography (PE/EtOAc = 10/1) to obtain **18** as white solid (45.3 mg, 74% yield).

^{1}H NMR (600 MHz, $CDCl_3$) δ 7.67-7.62 (m, 2H), 7.48-7.44 (m, 2H), 7.42-7.39 (m, 2H), 7.29-7.26 (m, 2H), 7.13-7.05 (m, 3H), 7.05-7.00 (m, 2H), 6.86-6.81 (m, 2H), 6.80-6.73 (m, 2H), 4.23 (d, *J* = 15.4 Hz, 1H), 3.76 (d, *J* = 15.4 Hz, 1H), 3.27 (d, *J* = 12.5 Hz, 1H), 2.69 (d, *J* = 14.0 Hz, 1H), 2.64 (d, *J* = 12.5 Hz, 1H), 2.52 (d, *J* = 14.6 Hz, 1H), 2.48-2.42 (m, 5H), 0.94 (s, 3H);

^{19}F NMR (565 MHz, $CDCl_3$) δ -84.57 (d, *J* = 30.1 Hz), -87.42 (d, *J* = 30.2 Hz), -115.06--115.66 (m);

^{13}C NMR (151 MHz, $CDCl_3$) δ 162.2, 160.6, 154.8 (dd, *J* = 293.8, 291.1 Hz), 144.1, 143.6, 140.1 (dd, *J* = 4.7, 2.9 Hz), 139.8, 137.5 (d, *J* = 3.4 Hz), 134.8, 132.4, 131.2 (d, *J* = 7.7 Hz), 129.8, 129.2 (t, *J* = 2.5 Hz), 128.8, 128.1, 127.1, 126.4, 118.5, 114.8 (d, *J* = 21.3 Hz), 111.1, 89.0 (dd, *J* = 22.4, 13.6 Hz), 61.6, 54.6, 47.0, 38.6 (t, *J* = 2.3 Hz), 38.2, 22.5, 21.6;

HRMS: (ESI) calcd for $C_{36}H_{32}F_3N_2O_2S^+$[M+H]$^+$ 613.2131; found 613.2138.

ethyl 4-(6-(2-(4-cyanophenyl)-3,3-difluoroallyl)-6-methyl-4-phenyl-1-tosyl-2,5,6,7-tetrahydro-1*H*-azepin-3-yl)benzoate (19)

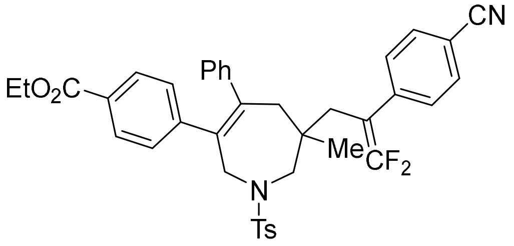

Chemical Formula: $C_{39}H_{36}F_2N_2O_4S$
Exact Mass: 666.2364

19 was prepared according to general procedure **2.1** using 4-methyl-*N*-(2-methylallyl)-*N*-(3-phenylprop-2-yn-1-yl)benzenesulfonamide (0.2 mmol, 67.9 mg), ethyl 4-bromobenzoate (0.2 mmol, 45.8 mg) and 4-(3,3,3-trifluoroprop-1-en-2-yl)benzonitrile (0.1 mmol, 19.7 mg), and was purified by silica gel column chromatography (PE/EtOAc = 5/1) to obtain **19** as white solid (29.3 mg, 44% yield).

^{1}H NMR (600 MHz, $CDCl_3$) δ 7.77-7.72 (m, 2H), 7.67-7.63 (m, 2H), 7.47-7.42 (m, 2H), 7.43-7.39 (m, 2H), 7.29-7.26 (m, 2H), 7.13-7.05 (m, 5H), 6.87-6.80 (m, 2H), 4.31 (q, *J* = 7.1 Hz, 2H), 4.25 (d, *J* = 15.4 Hz, 1H), 3.79 (d, *J* = 15.4 Hz, 1H), 3.29 (d, *J* = 12.5 Hz, 1H), 2.72 (d, *J* = 14.0 Hz, 1H), 2.67 (d, *J* = 12.5 Hz, 1H), 2.52 (d, *J* = 14.6 Hz, 1H), 2.49-2.42 (m, 5H), 1.34 (t, *J* = 7.1 Hz, 3H), 0.95 (s, 3H);

^{19}F NMR (376 MHz, $CDCl_3$) δ -84.40 (d, *J* = 30.0 Hz), -87.25 (d, *J* = 30.0 Hz);

^{13}C NMR (151 MHz, $CDCl_3$) δ 166.3, 154.7 (dd, *J* = 294.0, 291.3 Hz), 146.3, 143.7, 143.6, 141.1, 140.0 (dd, *J* = 4.7, 2.9 Hz), 135.0, 134.7, 132.3, 129.7, 129.4, 129.2 (t, *J* = 2.5 Hz), 129.1, 128.7, 128.3, 128.1, 127.0, 126.6, 118.4, 111.1, 88.9 (dd, *J* = 22.5, 13.7 Hz), 61.4, 60.8, 54.2, 47.0, 38.6 (t, *J* = 2.0 Hz), 38.2, 22.5, 21.5, 14.3;

HRMS: (ESI) calcd for $C_{39}H_{37}F_2N_2O_4S^+$[M+H]$^+$ 667.2437; found 667.2437.

4-(3-(6-(3,5-dimethylphenyl)-3-methyl-5-phenyl-1-tosyl-2,3,4,7-tetrahydro-1*H*-azepin-3-yl)-1,1-difluoroprop-1-en-2-yl)benzonitrile (20)

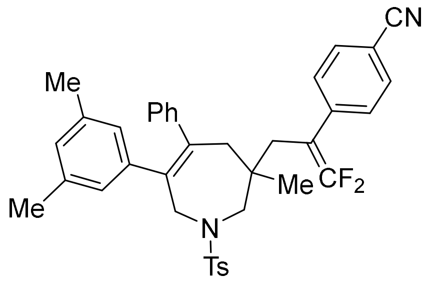

Chemical Formula: $C_{38}H_{36}F_2N_2O_2S$
Exact Mass: 622.2466

20 was prepared according to general procedure **2.1** using 4-methyl-*N*-(2-methylallyl)-*N*-(3-phenylprop-2-yn-1-yl)benzenesulfonamide (0.2 mmol, 67.9 mg), 1-bromo-3,5-dimethylbenzene (0.2 mmol, 37.1 mg) and 4-(3,3,3-trifluoroprop-1-en-2-yl)benzonitrile (0.1 mmol, 19.7 mg), and was purified by silica gel column chromatography (PE/EtOAc = 10/1) to obtain **20** as white solid (41.1 mg, 66% yield).

^{1}H NMR (400 MHz, $CDCl_3$) δ 7.68-7.61 (m, 2H), 7.53-7.46 (m, 2H), 7.45-7.38 (m, 2H), 7.30-7.26 (m, 2H), 7.12-7.04 (m, 3H), 6.88-6.81 (m, 2H), 6.68 (s, 2H), 6.55 (s, 3H), 4.24 (d, *J* = 15.3 Hz, 1H), 3.83 (d, *J* = 15.3 Hz, 1H), 3.25 (d, *J* = 12.6 Hz, 1H), 2.74 (d, *J* = 12.6 Hz, 1H), 2.64 (d, *J* = 13.9 Hz, 1H), 2.50 (t, *J* = 2.3 Hz, 2H), 2.47-2.42 (m, 4H), 2.07 (s, 6H), 0.94 (s, 3H);

^{F}19 NMR (376 MHz, $CDCl_3$) δ -84.68 (d, *J* = 31.0 Hz), -87.53 (d, *J* = 31.0 Hz);

^{13}C NMR (151 MHz, $CDCl_3$) δ 154.7 (dd, *J* = 293.7, 291.2 Hz), 144.2, 143.4, 141.3, 140.1 (dd, *J* = 4.7, 2.9 Hz), 138.8, 137.1, 135.1, 134.9, 132.3, 129.7, 129.2 (t, *J* = 2.5 Hz), 128.7, 128.1, 127.8, 127.18, 127.16, 126.1, 118.5, 111.0, 89.1 (dd, *J* = 22.5, 13.5 Hz), 60.3, 54.6, 46.4, 38.9 (t, *J* = 2.5 Hz), 38.0, 22.6, 21.5, 21.1;

HRMS: (ESI) calcd for $C_{38}H_{37}F_2N_2O_2S^+[M+H]^+$ 623.2538; found 623.2533.

4-(1,1-difluoro-3-(3-methyl-5-phenyl-1-tosyl-6-(3,4,5-trimethoxyphenyl)-2,3,4,7-tetrahydro-1*H*-azepin-3-yl)prop-1-en-2-yl)benzonitrile (21)

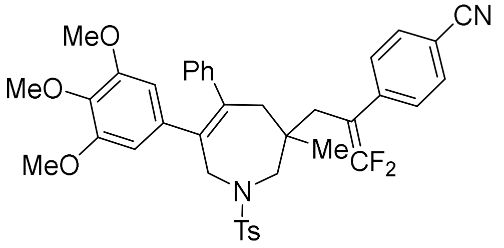

Chemical Formula: $C_{39}H_{38}F_2N_2O_5S$
Exact Mass: 684.2469

21 was prepared according to general procedure **2.1** using 4-methyl-*N*-(2-methylallyl)-*N*-(3-phenylprop-2-yn-1-yl)benzenesulfonamide (0.2 mmol, 67.9 mg), 5-bromo-1,2,3-trimethoxybenzene (0.2 mmol, 49.4 mg) and 4-(3,3,3-trifluoroprop-1-en-2-yl)benzonitrile (0.1 mmol, 19.7 mg), and was purified by silica gel column chromatography (PE/EtOAc = 3/1) to obtain **21** as white solid (49.9 mg, 73% yield).

^{1}H NMR (600 MHz, $CDCl_3$) δ 7.66-7.62 (m, 2H), 7.47-7.43 (m, 2H), 7.42-7.38 (m, 2H), 7.27 (d, *J* = 8.1 Hz, 2H), 7.16-7.10 (m, 2H), 7.11-7.06 (m, 1H), 6.92-6.87 (m, 2H), 6.44 (s, 2H), 4.33 (d, *J* = 15.3 Hz, 1H), 3.76 (s, 3H), 3.71 (d, *J* = 15.2 Hz, 1H), 3.56 (s, 6H), 3.27 (d, *J* = 12.3 Hz, 1H), 2.69 (d, *J* = 14.0 Hz, 1H), 2.58 (d, *J* = 12.0 Hz, 1H), 2.53 (d, *J* = 14.8 Hz, 1H), 2.48-2.42 (m, 4H), 2.39 (dt, *J* = 14.6, 2.4 Hz, 1H), 0.93 (s, 3H);

^{19}F NMR (565 MHz, $CDCl_3$) δ -84.42 (d, *J* = 30.0 Hz), -87.34 (d, *J* = 29.6 Hz);

^{13}C NMR (151 MHz, $CDCl_3$) δ 154.7 (dd, *J* = 294.1, 291.3 Hz), 152.4, 144.7, 143.6, 140.1 (dd, *J* = 5.0, 2.8 Hz), 139.5, 136.7, 136.4, 135.9, 134.7, 132.3, 129.7, 129.2 (t, *J* = 2.4 Hz), 128.5, 128.1, 127.0, 126.3, 118.5, 111.1, 107.0, 88.9 (dd, *J* = 22.5, 13.7 Hz), 62.4, 60.8, 55.9, 54.4, 47.4, 38.3 (t, *J* = 2.3 Hz), 38.2, 22.3, 21.5;

HRMS: (ESI) calcd for $C_{39}H_{39}F_2N_2O_5S^+$[M+H]$^+$ 685.2542; found 685.2534.

4-(3-(6-(benzo[*d*][1,3]dioxol-5-yl)-3-methyl-5-phenyl-1-tosyl-2,3,4,7-tetrahydro-1*H*-azepin-3-yl)-1,1-difluoroprop-1-en-2-yl)benzonitrile (22)

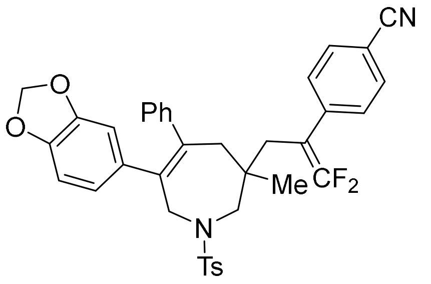

Chemical Formula: $C_{37}H_{32}F_2N_2O_4S$
Exact Mass: 638.2051

22 was prepared according to general procedure **2.1** using 4-methyl-*N*-(2-methylallyl)-*N*-(3-phenylprop-2-yn-1-yl)benzenesulfonamide (0.2 mmol, 67.9 mg), 5-bromobenzo[*d*][1,3]dioxole (0.2 mmol, 40.2 mg) and 4-(3,3,3-trifluoroprop-1-en-2-yl)benzonitrile (0.1 mmol, 19.7 mg), and was purified by silica gel column chromatography (PE/EtOAc = 4/1) to obtain **22** as white solid (49.8 mg, 78% yield).

^{1}H NMR (400 MHz, $CDCl_3$) δ 7.69-7.60 (m, 2H), 7.50-7.43 (m, 2H), 7.43-7.38 (m, 2H), 7.30-7.26 (m, 2H), 7.15-7.04 (m, 3H), 6.92-6.82 (m, 2H), 6.57-6.46 (m, 3H), 5.88-5.80 (m, 2H), 4.21 (d, *J* = 15.3 Hz, 1H), 3.76 (d, *J* = 15.4 Hz, 1H), 3.24 (d, *J* = 12.5 Hz, 1H), 2.65 (d, *J* = 14.0 Hz, 2H), 2.51 (d, *J* = 14.6 Hz, 1H), 2.47-2.38 (m, 5H), 0.92 (s, 3H);

^{19}F NMR (376 MHz, $CDCl_3$) δ -84.53 (d, *J* = 30.1 Hz), -87.41 (d, *J* = 30.1 Hz).

^{13}C NMR (151 MHz, $CDCl_3$) δ 154.7 (dd, *J* = 293.8, 291.3 Hz), 147.0, 146.0, 144.3, 143.5, 140.1 (dd, *J* = 4.9, 2.9 Hz), 138.9, 135.5, 135.1, 134.8, 132.3, 129.7, 129.2 (t, *J* = 2.5 Hz), 128.7, 128.0, 127.1, 126.2, 123.2, 118.5, 111.0, 109.9, 107.7, 100.7, 89.0 (dd, *J* = 22.5, 13.6 Hz), 61.1, 54.7, 46.7, 38.7 (t, *J* = 2.4 Hz), 38.1, 22.5, 21.5;

HRMS: (ESI) calcd for $C_{37}H_{33}F_2N_2O_4S^+$[M+H]$^+$ 639.2124; found 639.2102.

4-(1,1-difluoro-3-(3-methyl-6-(naphthalen-2-yl)-5-phenyl-1-tosyl-2,3,4,7-tetrahydro-1*H*-azepin-3-yl)prop-1-en-2-yl)benzonitrile (23)

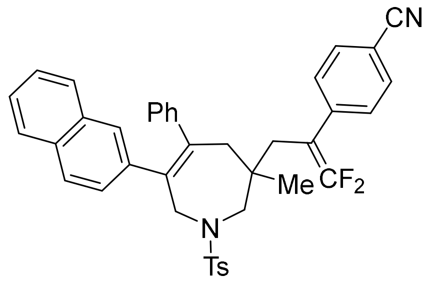

Chemical Formula: $C_{40}H_{34}F_2N_2O_2S$
Exact Mass: 644.2309

23 was prepared according to general procedure **2.1** using 4-methyl-*N*-(2-methylallyl)-*N*-(3-phenylprop-2-yn-1-yl)benzenesulfonamide (0.2 mmol, 67.9 mg), 2-bromonaphthalene (0.2 mmol, 41.4 mg) and 4-(3,3,3-trifluoroprop-1-en-2-yl)benzonitrile (0.1 mmol, 19.7 mg), and was purified by silica gel column chromatography (PE/EtOAc = 4/1) to obtain **23** as white solid (53.5 mg, 83% yield).

^{1}H NMR (600 MHz, $CDCl_3$) δ 7.70-7.61 (m, 5H), 7.52-7.46 (m, 3H), 7.45-7.41 (m, 2H), 7.40-7.37 (m, 2H), 7.29-7.26 (m, 2H), 7.08-7.00 (m, 4H), 6.93-6.88 (m, 2H), 4.38 (d, *J* = 15.4 Hz, 1H), 3.91 (d, *J* = 15.4 Hz, 1H), 3.33 (d, *J* = 12.5 Hz, 1H), 2.75 (d, *J* = 13.7 Hz, 2H), 2.56 (d, *J* = 14.6 Hz, 1H), 2.53-2.47 (m, 2H), 2.45 (s, 3H), 0.99 (s, 3H);

^{19}F NMR (376 MHz, $CDCl_3$) δ -84.48 (d, *J* = 30.0 Hz), -87.35 (d, *J* = 30.0 Hz);

^{13}C NMR (151 MHz, $CDCl_3$) δ 154.7 (dd, *J* = 293.8, 291.1 Hz), 144.0, 143.5, 140.1 (dd, *J* = 5.3, 2.7 Hz), 139.8, 139.1, 135.2, 134.8, 133.1, 132.3, 131.9, 129.7, 129.2 (t, *J* = 2.5 Hz), 128.9, 128.2, 128.03, 128.00, 127.9, 127.4, 127.1, 127.0, 126.3, 125.8, 125.8, 118.5, 111.0, 89.0 (dd, *J* = 22.4, 13.6 Hz), 61.0, 54.8, 46.8, 38.7 (t, *J* = 2.0 Hz), 38.1, 22.5, 21.5;

HRMS: (ESI) calcd for $C_{40}H_{35}F_2N_2O_2S^+$[M+H]$^+$ 645.2382; found 645.2361.

4-(1,1-difluoro-3-(6-(6-methoxypyridin-3-yl)-3-methyl-5-phenyl-1-tosyl-2,3,4,7-tetrahydro-1*H*-azepin-3-yl)prop-1-en-2-yl)benzonitrile (24)

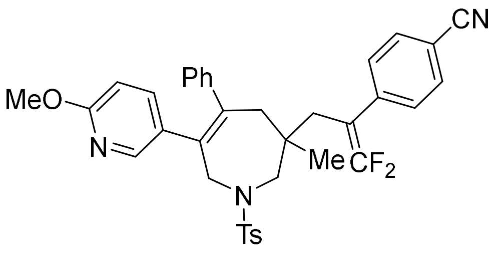

24 was prepared according to general procedure **2.1** using 4-methyl-*N*-(2-methylallyl)-*N*-(3-phenylprop-2-yn-1-yl)benzenesulfonamide (0.2 mmol, 67.9 mg), 5-bromo-2-methoxypyridine (0.2 mmol, 37.6 mg) and 4-(3,3,3-trifluoroprop-1-en-2-yl)benzonitrile (0.1 mmol, 19.7 mg) and was purified by silica gel column chromatography (PE/EtOAc = 5/1) to obtain **24** as white solid (41.9 mg,67% yield).

^{1}H NMR (600 MHz, $CDCl_3$) δ 7.68 (d, *J* = 2.5 Hz, 1H), 7.67-7.61 (m, 2H), 7.50-7.42 (m, 3H), 7.43-7.36 (m, 2H), 7.27 (d, *J* = 8.0 Hz, 2H), 7.16-7.05 (m, 3H), 6.89-6.82 (m, 2H), 6.53 (d, *J* = 8.6 Hz, 1H), 4.18 (d, *J* = 15.5 Hz, 1H), 3.81 (s, 3H), 3.74 (d, *J* = 15.5 Hz, 1H), 3.27 (d, *J* = 12.5 Hz, 1H), 2.70 (d, *J* = 14.1 Hz, 1H), 2.63 (d, *J* = 12.6 Hz, 1H), 2.51 (dt, *J* = 14.6, 2.2 Hz, 1H), 2.44 (s, 5H), 0.94 (s, 3H);

^{19}F NMR (565 MHz, $CDCl_3$) δ -84.58 (d, *J* = 30.1 Hz), -87.40 (d, *J* = 30.2 Hz);

^{13}C NMR (151 MHz, $CDCl_3$) δ 162.3, 154.7 (dd, *J* = 293.8, 291.3 Hz), 147.4, 143.9, 143.6, 140.3, 140.0 (dd, *J* = 4.7, 2.9 Hz), 139.6, 134.8, 132.4, 132.3, 130.4, 129.7, 129.2 (t, *J* = 2.4 Hz), 128.8, 128.3, 127.0, 126.5, 118.5, 111.1, 110.1, 88.9 (dd, *J* = 22.3, 13.7 Hz), 61.6, 54.3, 53.3, 47.0, 38.6 (t, *J* = 2.1 Hz), 38.2, 22.4, 21.5;

HRMS: (ESI) calcd for $C_{36}H_{34}F_2N_3O_3S^+$[M+H]$^+$ 626.2284; found 626.2267.

4-(1,1-difluoro-3-(3-methyl-5-phenyl-6-(thiophen-3-yl)-1-tosyl-2,3,4,7-tetrahydro-1*H*-azepin-3-yl)prop-1-en-2-yl)benzonitrile (25)

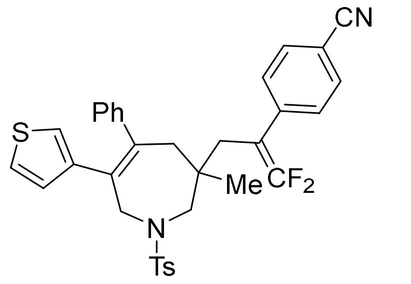

Chemical Formula: $C_{34}H_{30}F_2N_2O_2S_2$
Exact Mass: 600.1717

25 was prepared according to general procedure **2.1** using 4-methyl-*N*-(2-methylallyl)-*N*-(3-phenylprop-2-yn-1-yl)benzenesulfonamide (0.2 mmol, 67.9 mg), 3-bromothiophene (0.2 mmol, 32.6 mg) and 4-(3,3,3-trifluoroprop-1-en-2-yl)benzonitrile (0.1 mmol, 19.7 mg), and was purified by silica gel column chromatography (PE/EtOAc = 10/1) to obtain **25** as white solid (27.6 mg, 46% yield).

^{1}H NMR (600 MHz, $CDCl_3$) δ 7.68-7.61 (m, 2H), 7.50-7.44 (m, 2H), 7.41-7.37 (m, 2H), 7.30-7.26 (m, 2H), 7.22 (dd, *J* = 3.0, 1.3 Hz, 1H), 7.20-7.10 (m, 3H), 6.97-6.89 (m, 3H), 6.50 (dd, *J* = 5.1, 1.3 Hz, 1H), 4.33 (d, *J* = 15.4 Hz, 1H), 3.71 (d, *J* = 15.4 Hz, 1H), 3.29 (d, *J* = 12.4 Hz, 1H), 2.69 (d, *J* = 14.0 Hz, 1H), 2.57 (d, *J* = 12.8 Hz, 1H), 2.49 (d, *J* = 14.6 Hz, 1H), 2.45 (s, 3H), 2.43-2.36 (m, 2H), 0.92 (s, 3H);

^{19}F NMR (376 MHz, $CDCl_3$) δ -84.40 (d, *J* = 29.7 Hz), -87.32 (d, *J* = 30.5 Hz);

^{13}C NMR (151 MHz, $CDCl_3$) δ 154.7 (dd, *J* = 293.9, 291.4 Hz), 144.6, 143.6, 141.7, 140.1 (dd, *J* = 4.9, 2.8 Hz), 138.9, 135.0, 132.3, 130.9, 129.7, 129.2 (t, *J* = 2.6 Hz), 128.9, 128.5, 128.2, 127.0, 126.6, 124.10, 124.01, 118.5, 111.1, 88.9 (dd, *J* = 22.4, 13.6 Hz), 61.9, 54.4, 47.2, 38.5 (t, *J* = 2.3 Hz), 38.3, 22.1, 21.5;

HRMS: (ESI) calcd for $C_{34}H_{31}F_2N_2O_2S_2^+$[M+H]$^+$ 601.1790; found 601.1768.

4-(3-(6-(dibenzo[*b*,*d*]furan-2-yl)-3-methyl-5-phenyl-1-tosyl-2,3,4,7-tetrahydro-1*H*-azepin-3-yl)-1,1-difluoroprop-1-en-2-yl)benzonitrile (26)

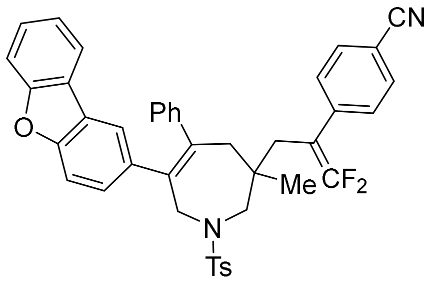

Chemical Formula: $C_{42}H_{34}F_2N_2O_3S$
Exact Mass: 684.2258

26 was prepared according to general procedure **2.1** using 4-methyl-*N*-(2-methylallyl)-*N*-(3-phenylprop-2-yn-1-yl)benzenesulfonamide (0.2 mmol, 67.9 mg), 2-bromodibenzo[*b*,*d*]furan (0.2 mmol, 49.4 mg) and 4-(3,3,3-trifluoroprop-1-en-2-yl)benzonitrile (0.1 mmol, 19.7 mg), and was purified by silica gel column chromatography (PE/EtOAc = 5/1) to obtain **26** as white solid (49.9 mg,73% yield).

^{1}H NMR (400 MHz, $CDCl_3$) δ 7.85-7.77 (m, 2H), 7.69-7.63 (m, 2H), 7.52-7.46 (m, 3H), 7.45-7.39 (m, 3H), 7.33-7.26 (m, 3H), 7.24-7.18 (m, 1H), 7.08-7.00 (m, 4H), 6.92-6.85 (m, 2H), 4.37 (d, *J* = 15.3 Hz, 1H), 3.88 (d, *J* = 15.3 Hz, 1H), 3.32 (d, *J* = 12.5 Hz, 1H), 2.78-2.63 (m, 2H), 2.60-2.45 (m, 3H), 2.44 (s, 3H), 0.99 (s, 3H);

^{19}F NMR (376 MHz, $CDCl_3$) δ -84.44 (d, *J* = 30.7 Hz), -87.33 (d, *J* = 30.7 Hz);

^{13}C NMR (151 MHz, $CDCl_3$) δ 156.4, 154.8, 154.76 (dd, *J* = 294.3, 291.3 Hz), 144.2, 143.6, 140.1 (dd, *J* = 4.7, 2.9 Hz), 139.5, 136.5, 135.5, 134.9, 132.4, 129.7, 129.2 (t, *J* = 2.8 Hz), 129.1, 128.9, 128.1, 127.13, 127.08, 126.3, 124.2, 124.0, 122.7, 121.5, 120.7, 118.5, 111.6, 111.1, 110.8, 89.0 (dd, *J* = 22.4, 13.6 Hz), 61.4, 55.1, 46.9, 38.8 (t, *J* = 2.2 Hz), 38.2, 22.5, 21.5;

HRMS: (ESI) calcd for $C_{42}H_{35}F_2N_2O_3S^+$[M+H]$^+$ 685.2331; found 685.2365.

4-(3-(6-(dibenzo[*b*,*d*]thiophen-2-yl)-3-methyl-5-phenyl-1-tosyl-2,3,4,7-tetrahydro-1*H*-azepin-3-yl)-1,1-difluoroprop-1-en-2-yl)benzonitrile (27)

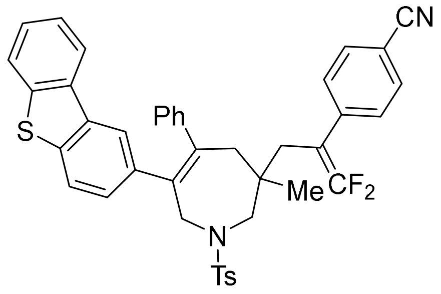

Chemical Formula: $C_{42}H_{34}F_2N_2O_2S_2$
Exact Mass: 700.2030

27 was prepared according to general procedure **2.1** using 4-methyl-*N*-(2-methylallyl)-*N*-(3-phenylprop-2-yn-1-yl)benzenesulfonamide (0.2 mmol, 67.9 mg), 2-bromodibenzo[*b*,*d*]thiophene (0.2 mmol, 52.6 mg) and 4-(3,3,3-trifluoroprop-1-en-2-yl)benzonitrile (0.1 mmol, 19.7 mg) and was purified by silica gel column chromatography (PE/EtOAc = 5/1) to obtain **27** as white solid (54.6 mg,78% yield).

^{1}H NMR (600 MHz, $CDCl_3$) δ 8.22 (d, *J* = 1.8 Hz, 1H), 8.08-8.01 (m, 1H), 7.82-7.77 (m, 1H), 7.66 (d, *J* = 8.2 Hz, 2H), 7.51-7.39 (m, 7H), 7.27 (d, *J* = 6.5 Hz, 2H), 7.11-7.02 (m, 3H), 6.97 (dd, *J* = 8.3, 1.8 Hz, 1H), 6.93-6.88 (m, 2H), 4.41 (d, *J* = 15.4 Hz, 1H), 3.86 (d, *J* = 15.4 Hz, 1H), 3.34 (d, *J* = 12.4 Hz, 1H), 2.75 (d, *J* = 13.8 Hz, 1H), 2.67 (d, *J* = 11.9 Hz, 1H), 2.56 (d, *J* = 14.5 Hz, 1H), 2.51 (d, *J* = 13.8 Hz, 1H), 2.43 (s, 4H), 0.99 (s, 3H);

^{19}F NMR (565 MHz, $CDCl_3$) δ -84.48 (d, *J* = 30.1 Hz), -87.37 (d, *J* = 30.2 Hz);

^{13}C NMR (151 MHz, $CDCl_3$) δ 154.8 (dd, *J* = 294.0, 291.3 Hz), 144.3, 143.6, 140.2 (dd, *J* = 4.6, 2.9 Hz), 139.9, 139.6, 138.1, 137.6, 135.6, 135.6, 135.5, 134.9, 132.4, 129.8, 129.3 (t, *J* = 2.9 Hz), 129.0, 128.6, 128.2, 127.1, 126.7, 126.4, 124.5, 122.7, 122.5, 121.8, 118.6, 111.1, 89.0 (dd, *J* = 22.5, 13.6 Hz), 61.9, 55.0, 47.2, 38.6 (t, *J* = 2.6 Hz), 38.3, 22.5, 21.6;

HRMS: (ESI) calcd for $C_{42}H_{35}F_2N_2O_2S_2^+$[M+H]$^+$ 701.2103; found 701.2118.

4-(3-(6-(benzo[*b*]thiophen-5-yl)-3-methyl-5-phenyl-1-tosyl-2,3,4,7-tetrahydro-1*H*-azepin-3-yl)-1,1-difluoroprop-1-en-2-yl)benzonitrile (28)

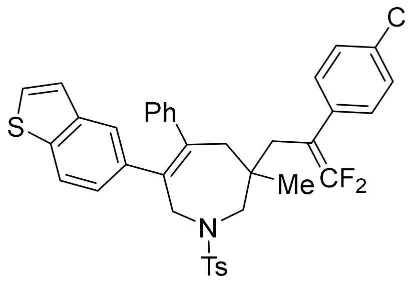

Chemical Formula: $C_{38}H_{32}F_2N_2O_2S_2$
Exact Mass: 650.1873

28 was prepared according to general procedure **2.1** using 4-methyl-*N*-(2-methylallyl)-*N*-(3-phenylprop-2-yn-1-yl)benzenesulfonamide (0.2 mmol, 67.9 mg), 5-bromobenzo[*b*]thiophene (0.2 mmol, 42.6 mg) and 4-(3,3,3-trifluoroprop-1-en-2-yl)benzonitrile (0.1 mmol, 19.7 mg), and was purified by silica gel column chromatography (PE/EtOAc = 5/1) to obtain **28** as white solid (41.7 mg, 64% yield).

^{1}H NMR (600 MHz, $CDCl_3$) δ 7.68-7.63 (m, 2H), 7.61 (d, *J* = 1.7 Hz, 1H), 7.51 (d, *J* = 8.4 Hz, 1H), 7.49-7.45 (m, 2H), 7.43-7.39 (m, 2H), 7.35 (d, *J* = 5.4 Hz, 1H), 7.28-7.26 (m, 2H), 7.15 (dd, *J* = 5.4, 0.8 Hz, 1H), 7.08-7.00 (m, 3H), 6.92-6.84 (m, 3H), 4.32 (d, *J* = 15.4 Hz, 1H), 3.87 (d, *J* = 15.4 Hz, 1H), 3.29 (d, *J* = 12.5 Hz, 1H), 2.72 (d, *J* = 14.0 Hz, 2H), 2.54 (d, *J* = 14.5 Hz, 1H), 2.50-2.45 (m, 2H), 2.44 (s, 3H), 0.97 (s, 3H);

^{19}F NMR (565 MHz, $CDCl_3$) δ -84.63 (d, *J* = 30.3 Hz), -87.49 (d, *J* = 30.2 Hz).

^{13}C NMR (151 MHz, $CDCl_3$) δ 154.7 (dd, *J* = 293.9, 291.3 Hz), 144.2, 143.5, 140.1 (dd, *J* = 4.5, 2.9 Hz), 139.5, 139.5, 137.9, 137.8, 135.4, 134.8, 132.3, 129.7, 129.2 (t, *J* = 2.5 Hz), 128.9, 128.0, 127.1, 126.4, 126.24, 126.21, 124.3, 124.0, 121.6, 118.5, 111.1, 89.0 (dd, *J* = 22.5, 13.6 Hz), 61.2, 55.0, 46.9, 38.8 (t, *J* = 2.5 Hz), 38.1, 22.6, 21.5;

HRMS: (ESI) calcd for $C_{38}H_{33}F_2N_2O_2S_2^+$[M+H]$^+$ 651.1946; found 651.1959.

4-(1,1-difluoro-3-(3-methyl-6-(1-methyl-1*H*-indol-5-yl)-5-phenyl-1-tosyl-2,3,4,7-tetrahydro-1*H*-azepin-3-yl)prop-1-en-2-yl)benzonitrile (29)

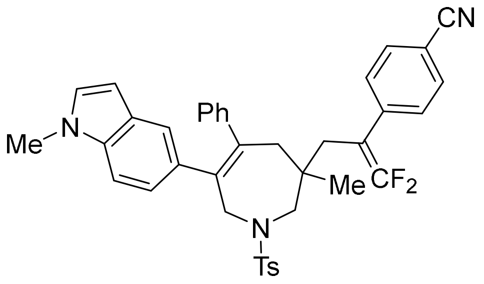

Chemical Formula: $C_{39}H_{35}F_2N_3O_2S$
Exact Mass: 647.2418

29 was prepared according to general procedure **2.1** using 4-methyl-*N*-(2-methylallyl)-*N*-(3-phenylprop-2-yn-1-yl)benzenesulfonamide (0.2 mmol, 67.9 mg), 5-bromo-1-methyl-1*H*-indole (0.2 mmol, 42.0 mg) and 4-(3,3,3-trifluoroprop-1-en-2-yl)benzonitrile (0.1 mmol, 19.7 mg), and was purified by silica gel column chromatography (PE/EtOAc = 5/1) to obtain **29** as white solid (46.0 mg, 71% yield).

^{1}H NMR (600 MHz, $CDCl_3$) δ 7.69-7.61 (m, 2H), 7.52-7.46 (m, 2H), 7.42 (dd, *J* = 8.4, 1.5 Hz, 2H), 7.32-7.28 (m, 2H), 7.27 (s, 1H), 7.07-6.99 (m, 3H), 6.99-6.93 (m, 2H), 6.92-6.85 (m, 2H), 6.80 (dd, *J* = 8.5, 1.7 Hz, 1H), 6.30 (dd, *J* = 3.1, 0.8 Hz, 1H), 4.33 (d, *J* = 15.4 Hz, 1H), 3.91 (d, *J* = 15.4 Hz, 1H), 3.68 (s, 3H), 3.27 (d, *J* = 12.6 Hz, 1H), 2.76 (d, *J* = 12.6 Hz, 1H), 2.68 (d, *J* = 13.8 Hz, 1H), 2.58-2.47 (m, 3H), 2.46 (s, 3H), 0.96 (s, 3H);

^{19}F NMR (376 MHz, $CDCl_3$) δ -84.69 (d, *J* = 31.0 Hz), -87.59 (d, *J* = 31.0 Hz);

^{13}C NMR (151 MHz, $CDCl_3$) δ 154.7 (dd, *J* = 293.6, 291.1 Hz), 144.8, 143.4, 140.2 (dd, *J* = 4.9, 2.8 Hz), 137.5, 136.0, 135.3, 134.9, 132.8, 132.3, 129.6, 129.2 (t, *J* = 2.8 Hz), 129.0, 128.9, 128.0, 127.8, 127.2, 125.8, 123.8, 121.6, 118.5, 111.0, 108.4, 101.1, 89.1 (dd, *J* = 22.6, 13.4 Hz), 60.5, 55.4, 46.5, 39.0 (t, *J* = 2.6 Hz), 38.0, 32.7, 22.7, 21.5;

HRMS: (ESI) calcd for $C_{39}H_{36}F_2N_3O_2S^+$[M+H]$^+$ 648.2491; found 648.2465.

4-(1,1-difluoro-3-(3-methyl-5-phenyl-6-(9-phenyl-9H-carbazol-3-yl)-1-tosyl-2,3,4,7-tetrahydro-1*H*-azepin-3-yl)prop-1-en-2-yl)benzonitrile (30)

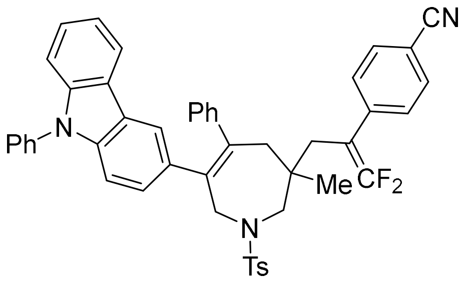

Chemical Formula: $C_{48}H_{39}F_2N_3O_2S$
Exact Mass: 759.2731

30 was prepared according to general procedure **2.1** using 4-methyl-*N*-(2-methylallyl)-*N*-(3-phenylprop-2-yn-1-yl)benzenesulfonamide (0.2 mmol, 67.9 mg), 3-bromo-9-phenyl-9*H*-carbazole (0.2 mmol, 62.4 mg) and 4-(3,3,3-trifluoroprop-1-en-2-yl)benzonitrile (0.1 mmol, 19.7 mg), and was purified by silica gel column chromatography (PE/EtOAc = 5/1) to obtain **30** as white solid (60.7 mg, 80% yield).

^{1}H NMR (600 MHz, $CDCl_3$) δ 8.01-7.94 (m, 2H), 7.67 (d, *J* = 8.4 Hz, 2H), 7.58-7.53 (m, 2H), 7.53-7.47 (m, 4H), 7.45-7.41 (m, 3H), 7.39-7.35 (m, 2H), 7.29 (d, *J* = 8.0 Hz, 2H), 7.26-7.22 (m, 1H), 7.09-7.04 (m, 3H), 7.04-6.97 (m, 2H), 6.96-6.90 (m, 2H), 4.42 (d, *J* = 15.4 Hz, 1H), 3.93 (d, *J* = 15.4 Hz, 1H), 3.33 (d, *J* = 12.5 Hz, 1H), 2.79-2.68 (m, 2H), 2.56 (d, *J* = 14.5 Hz, 1H), 2.53-2.48 (m, 2H), 2.45 (s, 3H), 1.00 (s, 3H).

^{19}F NMR (376 MHz, $CDCl_3$) δ -84.53 (d, *J* = 30.2 Hz), -87.43 (d, *J* = 30.5 Hz);

^{13}C NMR (151 MHz, $CDCl_3$) δ 154.7 (dd, *J* = 293.7, 291.3 Hz), 144.6, 143.4, 140.9, 140.2 (dd, *J* = 4.6, 2.8 Hz), 139.3, 138.3, 137.5, 135.8, 134.9, 133.5, 132.3, 129.74, 129.69, 129.2 (t, *J* = 2.9 Hz), 129.0, 127.96, 128.02, 127.3, 127.1, 126.8, 126.0, 125.9, 123.3, 123.0, 121.1, 120.3, 119.9, 118.5, 111.0, 109.7, 108.9, 89.1 (dd, *J* = 22.4, 13.5 Hz), 61.1, 55.3, 46.8, 38.8 (t, *J* = 2.6 Hz), 38.1, 22.5, 21.5;

HRMS: (ESI) calcd for $C_{48}H_{40}F_2N_3O_2S^+$[M+H]$^+$ 760.2804; found 760.2795.

methyl 4-(1,1-difluoro-3-(3-methyl-5,6-diphenyl-1-tosyl-2,3,4,7-tetrahydro-1*H*-azepin-3-yl)prop-1-en-2-yl)benzoate (31)

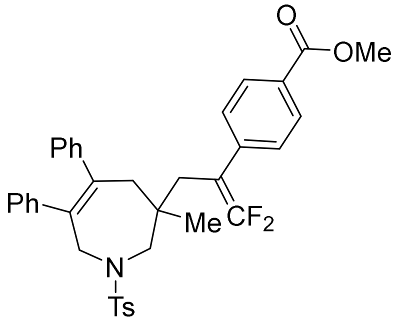

Chemical Formula: $C_{37}H_{35}F_2NO_4S$
Exact Mass: 627.2255

31 was prepared according to general procedure **2.1** using 4-methyl-*N*-(2-methylallyl)-*N*-(3-phenylprop-2-yn-1-yl)benzenesulfonamide (0.2 mmol, 67.9 mg), bromobenzene (0.2 mmol, 31.4 mg) and methyl 4-(3,3,3-trifluoroprop-1-en-2-yl)benzoate (0.1 mmol, 23.0 mg), and was purified by silica gel column chromatography (PE/EtOAc = 10/1) to obtain **31** as white solid (46.5 mg, 74% yield).

^{1}H NMR (600 MHz, $CDCl_3$) δ 8.08-8.02 (m, 2H), 7.43-7.35 (m, 4H), 7.20 (d, *J* = 8.0 Hz, 2H), 7.10-7.00 (m, 8H), 6.88-6.84 (m, 2H), 4.31 (d, *J* = 15.3 Hz, 1H), 3.97 (s, 3H), 3.71 (d, *J* = 15.3 Hz, 1H), 3.33 (d, *J* = 12.5 Hz, 1H), 2.71 (d, *J* = 14.0 Hz, 1H), 2.57 (d, *J* = 12.5 Hz, 1H), 2.53-2.44 (m, 3H), 2.41 (s, 3H), 0.98 (s, 3H);

^{19}F NMR (565 MHz, $CDCl_3$) δ -85.87 (d, *J* = 32.4 Hz), -88.39 (d, *J* = 32.9 Hz);

^{13}C NMR (151 MHz, $CDCl_3$) δ 166.6, 154.6 (dd, *J* = 293.1, 290.3 Hz), 144.3, 143.2, 141.7, 140.0 (dd, *J* = 4.5, 2.8 Hz), 139.6, 135.4, 134.7, 129.9, 129.6, 129.5, 129.0, 128.9, 128.5 (t, *J* = 2.3 Hz), 127.9, 127.8, 127.1, 126.4, 126.2, 89.3 (dd, *J* = 21.9, 14.0 Hz), 61.4, 54.6, 52.2, 46.9, 38.8, 38.7 (t, *J* = 2.1 Hz), 22.2, 21.5;

HRMS: (ESI) calcd for $C_{37}H_{36}F_2NO_4S^+[M+H]^+$ 628.2328; found 628.2303.

4-(1,1-difluoro-3-(3-methyl-5,6-diphenyl-1-tosyl-2,3,4,7-tetrahydro-1*H*-azepin-3-yl)prop-1-en-2-yl)-N,N-dimethylbenzamide (32)

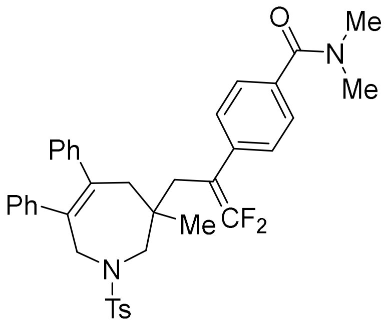

Chemical Formula: $C_{38}H_{38}F_2N_2O_3S$
Exact Mass: 640.2571

32 was prepared according to general procedure **2.1** using 4-methyl-*N*-(2-methylallyl)-*N*-(3-phenylprop-2-yn-1-yl)benzenesulfonamide (0.2 mmol, 67.9 mg), bromobenzene (0.2 mmol, 31.4 mg) and *N*,*N*-dimethyl-4-(3,3,3-trifluoroprop-1-en-2-yl)benzamide (0.1 mmol, 24.3 mg), and was purified by silica gel column chromatography (PE/EtOAc = 5/1) to obtain **32** as white solid (39.0 mg, 61% yield).

^{1}H NMR (600 MHz, $CDCl_3$) δ 7.48-7.42 (m, 4H), 7.37-7.28 (m, 4H), 7.10-6.99 (m, 8H), 6.89-6.83 (m, 2H), 4.26 (d, *J* = 15.3 Hz, 1H), 3.75 (d, *J* = 15.3 Hz, 1H), 3.37 (d, *J* = 12.5 Hz, 1H), 3.13 (s, 3H), 3.00 (s, 3H), 2.73-2.66 (m, 2H), 2.53-2.44 (m, 3H), 2.43 (s, 3H), 0.96 (s, 3H);

^{19}F NMR (565 MHz, $CDCl_3$) δ -86.68 (d, *J* = 34.9 Hz), -89.35 (d, *J* = 34.9 Hz);

^{13}C NMR (151 MHz, $CDCl_3$) δ 171.0, 154.6 (dd, *J* = 292.0, 289.6 Hz), 144.3, 143.4, 141.7, 139.7, 136.4 (dd, *J* = 4.4, 2.5 Hz), 135.3, 135.2, 134.7, 129.8, 129.5, 128.9, 128.4 (t, *J* = 2.9 Hz), 127.8, 127.8, 127.5, 127.1, 126.4, 126.1, 89.2 (dd, *J* = 21.7, 14.1 Hz), 61.3, 54.5, 46.9, 39.6, 38.7 (t, *J* = 2.7 Hz), 38.6, 35.4, 22.3, 21.5;

HRMS: (ESI) calcd for $C_{38}H_{39}F_2N_2O_3S^+$[M+H]$^+$ 641.2644; found 641.2626.

3-(3,3-difluoro-2-(4-(methylsulfonyl)phenyl)allyl)-3-methyl-5,6-diphenyl-1-tosyl-2,3,4,7-tetrahydro-1*H*-azepine (33)

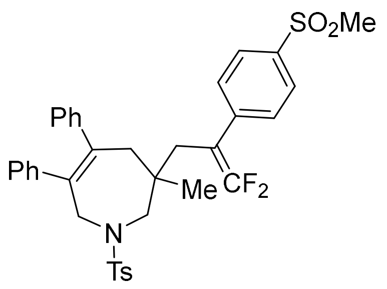

Chemical Formula: $C_{36}H_{35}F_2NO_4S_2$
Exact Mass: 647.1976

33 was prepared according to general procedure **2.1** using 4-methyl-*N*-(2-methylallyl)-*N*-(3-phenylprop-2-yn-1-yl)benzenesulfonamide (0.2 mmol, 67.9 mg), bromobenzene (0.2 mmol, 31.4 mg) and 1-(methylsulfonyl)-4-(3,3,3-trifluoroprop-1-en-2-yl)benzene (0.1 mmol, 25.0 mg), and was purified by silica gel column chromatography (PE/EtOAc = 2/1) to obtain **33** as white solid (42.8 mg, 66% yield).

^{1}H NMR (600 MHz, $CDCl_3$) δ 7.97-7.92 (m, 2H), 7.53-7.49 (m, 2H), 7.47-7.42 (m, 2H), 7.31-7.26 (m, 2H), 7.11-7.03 (m, 6H), 7.04-7.00 (m, 2H), 6.87-6.83 (m, 2H), 4.20 (d, *J* = 15.4 Hz, 1H), 3.83 (d, *J* = 15.4 Hz, 1H), 3.23 (d, *J* = 12.5 Hz, 1H), 3.08 (s, 3H), 2.77-2.64 (m, 2H), 2.57 (d, *J* = 14.5 Hz, 1H), 2.52-2.44 (m, 2H), 2.43 (s, 3H), 0.95 (s, 3H);

^{19}F NMR (376 MHz, $CDCl_3$) δ -84.47 (d, *J* = 30.2 Hz), -87.58 (d, *J* = 30.9 Hz);

^{13}C NMR (151 MHz, $CDCl_3$) δ 154.8 (dd, *J* = 293.6, 291.1 Hz), 144.2, 143.6, 141.6, 141.0 (dd, *J* = 5.0, 2.9 Hz), 139.3, 135.5, 134.6, 129.8, 129.5, 129.4 (t, *J* = 2.8 Hz), 128.9, 127.9, 127.8, 127.7, 127.1, 126.5, 126.2, 89.0 (dd, *J* = 22.4, 13.6 Hz), 61.0, 54.6, 46.9, 44.5, 38.8 (t, *J* = 2.4 Hz), 38.0, 22.7, 21.4;

HRMS: (ESI) calcd for $C_{36}H_{36}F_2NO_4S_2^+$[M+H]$^+$ 648.2048; found 648.2030.

1-(4-(1,1-difluoro-3-(3-methyl-5,6-diphenyl-1-tosyl-2,3,4,7-tetrahydro-1*H*-azepin-3-yl)prop-1-en-2-yl)phenyl)ethan-1-one (34)

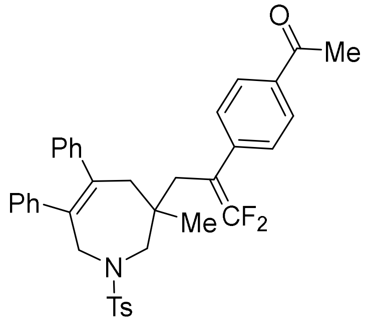

Chemical Formula: $C_{37}H_{35}F_2NO_3S$
Exact Mass: 611.2306

34 was prepared according to general procedure **2.1** using 4-methyl-*N*-(2-methylallyl)-*N*-(3-phenylprop-2-yn-1-yl)benzenesulfonamide (0.2 mmol, 67.9 mg), bromobenzene (0.2 mmol, 31.4 mg) and 1-(4-(3,3,3-trifluoroprop-1-en-2-yl)phenyl)ethan-1-one (0.1 mmol, 21.4 mg), and was purified by silica gel column chromatography (PE/EtOAc = 2/1) to obtain **34** as white solid (41.0 mg, 67% yield).

^{1}H NMR (600 MHz, $CDCl_3$) δ 7.99-7.95 (m, 2H), 7.44-7.38 (m, 4H), 7.22-7.16 (m, 2H), 7.11-7.01 (m, 8H), 6.88-6.83 (m, 2H), 4.29 (d, J = 15.3 Hz, 1H), 3.73 (d, J = 15.3 Hz, 1H), 3.33 (d, J = 12.5 Hz, 1H), 2.72 (d, J = 14.0 Hz, 1H), 2.54-2.44 (m, 3H), 2.41 (s, 3H), 0.97 (s, 3H);

^{19}F NMR (376 MHz, $CDCl_3$) δ -84.69 (d, J = 31.0 Hz), -87.59 (d, J = 31.0 Hz);

^{13}C NMR (151 MHz, $CDCl_3$) δ 197.5, 154.6 (dd, J = 293.0, 290.4 Hz), 144.3, 143.3, 141.6, 140.1 (dd, J = 4.4, 2.8 Hz), 139.6, 135.8, 135.4, 134.6, 129.6, 129.5, 128.9, 128.7 (t, J = 2.9 Hz), 128.6, 127.9, 127.8, 127.1, 126.4, 126.2, 89.2 (dd, J = 21.9, 13.8 Hz), 61.4, 54.6, 46.9, 38.6 (t, J = 2.5 Hz), 38.5, 26.7, 22.2, 21.5;

HRMS: (ESI) calcd for $C_{37}H_{36}F_2NO_3S^+[M+H]^+$ 612.2379; found 612.2359.

3-(3,3-difluoro-2-(4-(trifluoromethoxy)phenyl)allyl)-3-methyl-5,6-diphenyl-1-tosyl-2,3,4,7-tetrahydro-1*H*-azepine (35)

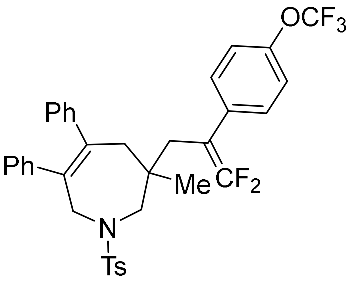

Chemical Formula: $C_{36}H_{32}F_5NO_3S$
Exact Mass: 653.2023

35 was prepared according to general procedure **2.1** using 4-methyl-*N*-(2-methylallyl)-*N*-(3-phenylprop-2-yn-1-yl)benzenesulfonamide (0.2 mmol, 67.9 mg), bromobenzene (0.2 mmol, 31.4 mg) and 1-(trifluoromethoxy)-4-(3,3,3-trifluoroprop-1-en-2-yl)benzene (0.1 mmol, 25.6 mg), and was purified by silica gel column chromatography (PE/EtOAc = 2/1) to obtain **35** as white solid (45.1 mg, 69% yield).

^{1}H NMR (600 MHz, $CDCl_3$) δ 7.49-7.44 (m, 2H), 7.33-7.29 (m, 2H), 7.26-7.23 (m, 2H), 7.23-7.20 (m, 2H), 7.11-7.02 (m, 8H), 6.87-6.83 (m, 2H), 4.32 (d, *J* = 15.4 Hz, 1H), 3.74 (d, *J* = 15.4 Hz, 1H), 3.37 (d, *J* = 12.5 Hz, 1H), 2.71 (d, *J* = 13.9 Hz, 1H), 2.63 (d, *J* = 12.5 Hz, 1H), 2.50-2.37 (m, 6H), 0.99 (s, 3H);

^{19}F NMR (376 MHz, $CDCl_3$) δ -57.63, -86.82 (d, *J* = 35.6 Hz), -89.41 (d, *J* = 35.6 Hz);

^{13}C NMR (151 MHz, $CDCl_3$) δ 154.6 (dd, *J* = 291.7, 289.4 Hz), 148.2, 144.4, 143.3, 141.7, 139.6, 135.4, 134.9, 133.7 (dd, *J* = 4.5, 2.4 Hz), 130.0 (t, *J* = 2.8 Hz), 129.6, 129.5, 128.9, 127.9, 127.8, 127.1, 126.4, 126.2, 120.9, 120.4 (q, *J* = 257.5 Hz), 88.7 (dd, *J* = 22.0, 14.4 Hz), 61.4, 54.6, 46.8, 38.9, 38.7 (t, *J* = 2.2 Hz), 22.2, 21.5;

HRMS: (ESI) calcd for $C_{36}H_{33}F_5NO_3S^+$[M+H]$^+$ 654.2096; found 654.2070.

3-(2-([1,1'-biphenyl]-4-yl)-3,3-difluoroallyl)-3-methyl-5,6-diphenyl-1-tosyl-2,3,4,7-tetrahydro-1*H*-azepine (36)

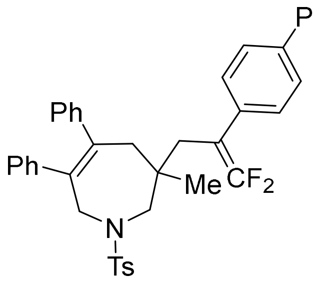

Chemical Formula: $C_{41}H_{37}F_2NO_2S$
Exact Mass: 645.2513

36 was prepared according to general procedure **2.1** using 4-methyl-*N*-(2-methylallyl)-*N*-(3-phenylprop-2-yn-1-yl)benzenesulfonamide (0.2 mmol, 67.9 mg), bromobenzene (0.2 mmol, 31.4 mg) and 4-(3,3,3-trifluoroprop-1-en-2-yl)-1,1'-biphenyl (0.1 mmol, 24.8 mg), and was purified by silica gel column chromatography (PE/EtOAc = 10/1) to obtain **36** as white solid (42.6 mg, 66% yield).

^{1}H NMR (600 MHz, $CDCl_3$) δ 7.71-7.62 (m, 4H), 7.52-7.47 (m, 2H), 7.42-7.35 (m, 5H), 7.13-7.02 (m, 10H), 6.92-6.87 (m, 2H), 4.36 (d, *J* = 15.3 Hz, 1H), 3.66 (d, *J* = 15.4 Hz, 1H), 3.46 (d, *J* = 12.5 Hz, 1H), 2.77 (d, *J* = 14.0 Hz, 1H), 2.56-2.50 (m, 2H), 2.49-2.47 (m, 2H), 2.28 (s, 3H), 1.07 (s, 3H);

^{19}F NMR (565 MHz, $CDCl_3$) δ -87.48 (d, *J* = 36.5 Hz), -89.91 (d, *J* = 36.2 Hz);

^{13}C NMR (151 MHz, $CDCl_3$) δ 154.6 (dd, *J* = 289.4 Hz, *J* = 289.3 Hz), 144.5, 143.1, 141.8, 140.2, 139.94, 139.87, 135.4, 134.8, 134.0 (dd, *J* = 4.2, 2.6 Hz), 129.6, 129.5, 129.0, 128.93, 128.90 (t, *J* = 2.8 Hz), 127.9, 127.8, 127.6, 127.05, 127.11, 126.9, 126.4, 126.1, 89.2 (dd, *J* = 21.3, 14.3 Hz), 61.7, 54.6, 47.1, 39.2, 38.7 (t, *J* = 2.2 Hz), 21.9, 21.3;

HRMS: (ESI) calcd for $C_{41}H_{38}F_2NO_2S^+$[M+H]$^+$ 646.2586; found 646.2570.

3-(3,3-difluoro-2-(naphthalen-2-yl)allyl)-3-methyl-5,6-diphenyl-1-tosyl-2,3,4,7-tetrahydro-1*H*-azepine (37)

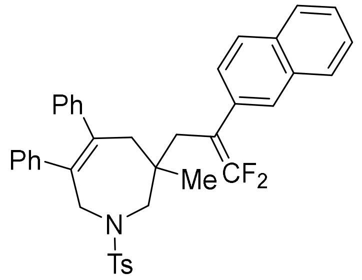

Chemical Formula: $C_{39}H_{35}F_2NO_2S$
Exact Mass: 619.2357

37 was prepared according to general procedure **2.1** using 4-methyl-*N*-(2-methylallyl)-*N*-(3-phenylprop-2-yn-1-yl)benzenesulfonamide (0.2 mmol, 67.9 mg), bromobenzene (0.2 mmol, 31.4 mg) and 2-(3,3,3-trifluoroprop-1-en-2-yl)naphthalene (0.1 mmol, 22.2 mg), and was purified by silica gel column chromatography (PE/EtOAc = 10/1) to obtain **37** as white solid (42.1 mg, 68% yield).

^{1}H NMR (400 MHz, $CDCl_3$) δ 7.96-7.82 (m, 3H), 7.78 (s, 1H), 7.61-7.52 (m, 2H), 7.48-7.41 (m, 1H), 7.15-7.01 (m, 10H), 6.91-6.84 (m, 2H), 6.73 (d, *J* = 8.2 Hz, 2H), 4.34 (d, *J* = 15.2 Hz, 1H), 3.56 (d, *J* = 15.3 Hz, 1H), 3.43 (d, *J* = 12.4 Hz, 1H), 2.80 (d, *J* = 14.0 Hz, 1H), 2.58-2.49 (m, 3H), 2.38 (d, *J* = 12.4 Hz, 1H), 2.27 (s, 3H), 1.09 (s, 3H);

^{19}F NMR (376 MHz, $CDCl_3$) δ -87.12 (d, *J* = 35.8 Hz), -89.81 (d, *J* = 36.1 Hz);

^{13}C NMR (151 MHz, $CDCl_3$) δ 154.8 (dd, *J* = 291.7, 289.3 Hz), 144.5, 142.9, 141.8, 139.9, 135.5, 134.4, 133.3, 132.5 (t, *J* = 3.7 Hz), 129.5, 129.3, 129.0, 128.3, 128.1, 127.9, 127.7, 127.7, 127.5 (t, *J* = 2.9 Hz), 126.9, 126.5, 126.3, 126.3, 126.1, 89.6 (dd, *J* = 21.3, 14.2 Hz), 62.0, 54.6, 47.2, 39.5, 38.7 (t, *J* = 2.6 Hz), 21.8, 21.3;

HRMS: (ESI) calcd for $C_{39}H_{36}F_2NO_2S^+$[M+H]$^+$ 620.2429; found 620.2406.

3-(2-(4-chlorophenyl)-3,3-difluoroallyl)-3-methyl-5,6-diphenyl-1-tosyl-2,3,4,7-tetrahydro-1*H*-azepine (38)

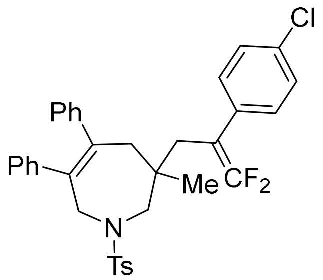

Chemical Formula: $C_{35}H_{32}ClF_2NO_2S$
Exact Mass: 603.1810

38 was prepared according to general procedure **2.1** using 4-methyl-*N*-(2-methylallyl)-*N*-(3-phenylprop-2-yn-1-yl)benzenesulfonamide (0.2 mmol, 67.9 mg), bromobenzene (0.2 mmol, 31.4 mg) and 1-chloro-4-(3,3,3-trifluoroprop-1-en-2-yl)benzene (0.1 mmol, 20.6 mg), and was purified by silica gel column chromatography (PE/EtOAc = 10/1) to obtain **38** as white solid (46.5 mg, 77% yield).

^{1}H NMR (600 MHz, $CDCl_3$) δ 7.42-7.38 (m, 2H), 7.38-7.34 (m, 2H), 7.27-7.22 (m, 4H), 7.12-7.02 (m, 8H), 6.90-6.86 (m, 2H), 4.34 (d, *J* = 15.3 Hz, 1H), 3.68 (d, *J* = 15.3 Hz, 1H), 3.35 (d, *J* = 12.5 Hz, 1H), 2.74 (d, *J* = 13.9 Hz, 1H), 2.52-2.45 (m, 2H), 2.43 (d, *J* = 12.6 Hz, 5H), 1.02 (s, 3H);

^{19}F NMR (376 MHz, $CDCl_3$) δ -86.95 (d, *J* = 35.6 Hz), -89.42 (d, *J* = 35.5 Hz);

^{13}C NMR (151 MHz, $CDCl_3$) δ 154.6 (dd, *J* = 291.9, 289.6 Hz), 144.4, 143.4, 141.8, 139.7, 135.5, 134.8, 133.6 (dd, *J* = 4.6, 2.4 Hz), 133.2, 129.9 (t, *J* = 2.8 Hz), 129.7, 129.6, 129.0, 128.9, 127.9, 127.8, 127.1, 126.5, 126.2, 88.8 (dd, *J* = 21.8, 14.2 Hz), 61.6, 54.7, 47.0, 39.1, 38.7 (t, *J* = 2.1 Hz), 22.0, 21.6;

HRMS: (ESI) calcd for $C_{35}H_{33}ClF_2NO_2S^+$[M+H]$^+$ 604.1883; found 604.1863.

3-(2-(benzo[*d*][1,3]dioxol-5-yl)-3,3-difluoroallyl)-3-methyl-5,6-diphenyl-1-tosyl-2,3,4,7-tetrahydro-1*H*-azepine (39)

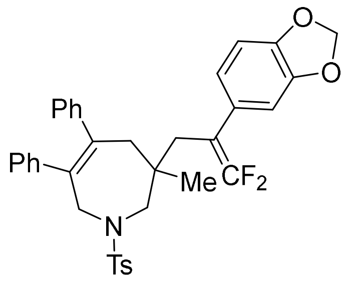

Chemical Formula: $C_{36}H_{33}F_2NO_4S$
Exact Mass: 613.2098

39 was prepared according to general procedure **2.1** using 4-methyl-*N*-(2-methylallyl)-*N*-(3-phenylprop-2-yn-1-yl)benzenesulfonamide (0.2 mmol, 67.9 mg), bromobenzene (0.2 mmol, 31.4 mg) and 5-(3,3,3-trifluoroprop-1-en-2-yl)benzo[*d*][1,3]dioxole (0.1 mmol, 21.6 mg), and was purified by silica gel column chromatography (PE/EtOAc = 5/1) to obtain **39** as white solid (34.3 mg, 56% yield).

^{1}H NMR (400 MHz, $CDCl_3$) δ 7.48 (d, *J* = 8.3 Hz, 2H), 7.25 (d, *J* = 7.6 Hz, 2H), 7.14-7.01 (m, 8H), 6.92-6.85 (m, 2H), 6.82 (d, *J* = 8.6 Hz, 1H), 6.78-6.72 (m, 2H), 6.04-5.97 (m, 2H), 4.36 (d, *J* = 15.2 Hz, 1H), 3.70 (d, *J* = 15.3 Hz, 1H), 3.42 (d, *J* = 12.5 Hz, 1H), 2.74 (d, *J* = 14.0 Hz, 1H), 2.58 (d, *J* = 12.5 Hz, 1H), 2.49 (d, *J* = 14.2 Hz, 1H), 2.43 (s, 3H), 2.40-2.33 (m, 2H), 1.00 (s, 3H);

^{19}F NMR (376 MHz, $CDCl_3$) δ -88.30 (d, *J* = 38.6 Hz), -90.24 (d, *J* = 38.6 Hz);

^{13}C NMR (151 MHz, $CDCl_3$) δ 155.4 (dd, *J* = 290.0, 289.9 Hz), 147.7, 146.8, 144.4, 143.2, 141.8, 139.9, 135.4, 134.9, 129.6, 129.5, 129.0, 128.6 (dd, *J* = 4.6, 2.3 Hz), 127.8, 127.8, 127.2, 126.4, 126.1, 122.1 (t, *J* = 2.3 Hz), 109.0 (t, *J* = 2.5 Hz), 108.4, 101.2, 89.2 (dd, *J* = 21.6, 14.6 Hz), 61.6, 54.6, 46.9, 39.4, 38.5 (t, *J* = 2.4 Hz), 22.1, 21.5;

HRMS: (ESI) calcd for $C_{36}H_{34}F_2NO_4S^+$[M+H]$^+$ 614.2171; found 614.2147.

3-(2-(4-(benzyloxy)phenyl)-3,3-difluoroallyl)-3-methyl-5,6-diphenyl-1-tosyl-2,3,4,7-tetrahydro-1*H*-azepine (40)

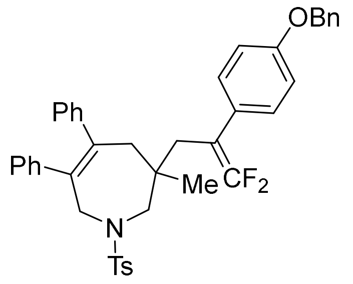

Chemical Formula: $C_{42}H_{39}F_2NO_3S$
Exact Mass: 675.2619

40 was prepared according to general procedure **2.1** using 4-methyl-*N*-(2-methylallyl)-*N*-(3-phenylprop-2-yn-1-yl)benzenesulfonamide (0.2 mmol, 67.9 mg), bromobenzene (0.2 mmol, 31.4 mg) and 1-(benzyloxy)-4-(3,3,3-trifluoroprop-1-en-2-yl)benzene (0.1 mmol, 27.8 mg), and was purified by silica gel column chromatography (PE/EtOAc = 5/1) to obtain **40** as white solid (40.5 mg, 60% yield).

^{1}H NMR (600 MHz, $CDCl_3$) δ 7.51-7.46 (m, 2H), 7.45-7.39 (m, 4H), 7.39-7.34 (m, 1H), 7.25-7.20 (m, 4H), 7.11-7.03 (m, 8H), 7.01 (d, *J* = 8.8 Hz, 2H), 6.91-6.86 (m, 2H), 5.11 (s, 2H), 4.35 (d, *J* = 15.3 Hz, 1H), 3.65 (d, *J* = 15.3 Hz, 1H), 3.45 (d, *J* = 12.5 Hz, 1H), 2.74 (d, *J* = 14.0 Hz, 1H), 2.55-2.46 (m, 2H), 2.43-2.37 (m, 5H), 1.03 (s, 3H);

^{19}F NMR (376 MHz, $CDCl_3$) δ -88.67 (d, *J* = 39.2 Hz), -91.02 (d, *J* = 39.1 Hz);

^{13}C NMR (151 MHz, $CDCl_3$) δ 158.0, 154.5 (dd, *J* = 289.4, 289.2 Hz), 144.5, 143.1, 141.8, 139.9, 136.7, 135.3, 134.9, 129.7 (t, *J* = 2.7 Hz), 129.6, 129.5, 129.0, 128.7, 128.1, 127.84, 127.76, 127.5, 127.4 (dd, *J* = 4.2, 2.4 Hz), 127.2, 126.3, 126.1, 114.9, 88.9 (dd, *J* = 21.1, 14.7 Hz), 70.1, 61.8, 54.5, 47.1, 39.4, 38.5 (t, *J* = 2.2 Hz), 21.9, 21.5;

HRMS: (ESI) calcd for $C_{42}H_{40}F_2NO_3S^+$[M+H]$^+$ 676.2692; found 676.2673.

3-(1,1-difluoro-3-(3-methyl-5,6-diphenyl-1-tosyl-2,3,4,7-tetrahydro-1*H*-azepin-3-yl)prop-1-en-2-yl)quinoline (41)

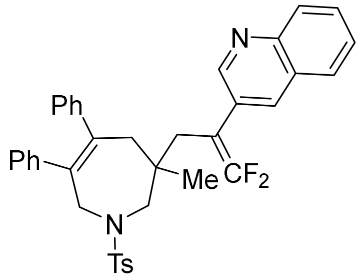

Chemical Formula: $C_{38}H_{34}F_2N_2O_2S$
Exact Mass: 620.2309

41 was prepared according to general procedure **2.1** using 4-methyl-*N*-(2-methylallyl)-*N*-(3-phenylprop-2-yn-1-yl)benzenesulfonamide (0.2 mmol, 67.9 mg), bromobenzene (0.2 mmol, 31.4 mg) and 3-(3,3,3-trifluoroprop-1-en-2-yl)quinoline (0.1 mmol, 22.3 mg), and was purified by silica gel column chromatography (PE/EtOAc = 3/1) to obtain **41** as white solid (54.0 mg, 87% yield).

^{1}H NMR (600 MHz, $CDCl_3$) δ 8.86 (t, *J* = 1.9 Hz, 1H), 8.17 (dd, *J* = 8.4, 1.1 Hz, 1H), 8.09 (d, *J* = 2.2 Hz, 1H), 7.85 (dd, *J* = 8.2, 1.4 Hz, 1H), 7.82-7.75 (m, 1H), 7.65-7.59 (m, 1H), 7.29-7.25 (m, 2H), 7.10-7.00 (m, 8H), 6.92 (d, *J* = 7.9 Hz, 2H), 6.89-6.84 (m, 2H), 4.28 (d, *J* = 15.3 Hz, 1H), 3.73 (d, *J* = 15.3 Hz, 1H), 3.33 (d, *J* = 12.5 Hz, 1H), 2.78 (d, *J* = 14.0 Hz, 1H), 2.66-2.55 (m, 3H), 2.53 (d, *J* = 13.9 Hz, 1H), 2.31 (s, 3H), 1.04 (s, 3H);

^{19}F NMR (376 MHz, $CDCl_3$) δ -84.99 (d, *J* = 32.7 Hz), -88.52 (d, *J* = 32.4 Hz);

^{13}C NMR (151 MHz, $CDCl_3$) δ 154.2 (dd, *J* = 292.2, 291.8 Hz), 150.1 (t, *J* = 3.1 Hz), 147.1, 144.3, 143.1, 141.6, 139.5, 135.5, 135.1 (t, *J* = 2.8 Hz), 134.5, 129.8, 129.5, 129.5, 129.3, 128.9, 128.2 (dd, *J* = 3.2, 3.0 Hz), 127.9, 127.9, 127.8, 127.6, 127.2, 126.9, 126.4, 126.2, 87.1 (dd, *J* = 22.7, 14.4 Hz), 61.5, 54.6, 47.0, 38.8 (t, *J* = 2.0 Hz), 38.6, 22.4, 21.4;

HRMS: (ESI) calcd for $C_{38}H_{35}F_2N_2O_2S^+$[M+H]$^+$ 621.2382; found 621.2358.

3-(2-(dibenzo[*b*,*d*]thiophen-2-yl)-3,3-difluoroallyl)-3-methyl-5,6-diphenyl-1-tosyl-2,3,4,7-tetrahydro-1*H*-azepine (42)

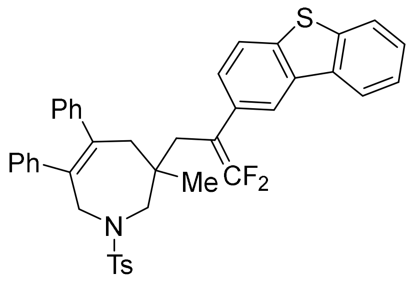

Chemical Formula: $C_{41}H_{35}F_2NO_2S_2$
Exact Mass: 675.2077

42 was prepared according to general procedure **2.1** using 4-methyl-*N*-(2-methylallyl)-*N*-(3-phenylprop-2-yn-1-yl)benzenesulfonamide (0.2 mmol, 67.9 mg), bromobenzene (0.2 mmol, 31.4 mg) and 2-(3,3,3-trifluoroprop-1-en-2-yl)dibenzo[*b*,*d*]thiophene (0.1 mmol, 27.8 mg), and was purified by silica gel column chromatography (PE/EtOAc = 3/1) to obtain **42** as white solid (55.4 mg, 82% yield).

^{1}H NMR (600 MHz, $CDCl_3$) δ 8.21-8.15 (m, 1H), 8.07 (t, *J* = 1.5 Hz, 1H), 7.95-7.90 (m, 1H), 7.87 (d, *J* = 8.3 Hz, 1H), 7.55-7.47 (m, 2H), 7.44-7.38 (m, 1H), 7.23-7.17 (m, 2H), 7.09-7.00 (m, 8H), 6.92-6.85 (m, 2H), 6.83-6.79 (m, 2H), 4.33 (d, *J* = 15.2 Hz, 1H), 3.60 (d, *J* = 15.3 Hz, 1H), 3.42 (d, *J* = 12.5 Hz, 1H), 2.80 (d, *J* = 14.0 Hz, 1H), 2.59-2.51 (m, 3H), 2.45 (d, *J* = 12.5 Hz, 1H), 2.18 (s, 3H), 1.10 (s, 3H);

^{19}F NMR (376 MHz, $CDCl_3$) δ -87.43 (d, *J* = 36.9 Hz), -90.00 (d, *J* = 36.6 Hz);

^{13}C NMR (151 MHz, $CDCl_3$) δ 155.7 (dd, *J* = 289.9, 289.5 Hz), 144.5, 143.0, 141.8, 139.8, 139.7, 138.4, 135.9, 135.5, 135.2, 134.4, 131.5 (dd, *J* = 4.4, 2.3 Hz), 129.5, 129.3, 128.9, 127.9, 127.8, 127.2, 127.1 (t, *J* = 2.6 Hz), 126.9, 126.3, 126.1, 124.6, 123.0, 122.9, 121.9, 121.4 (t, *J* = 2.8 Hz), 89.5 (dd, *J* = 21.4, 14.6 Hz), 61.9, 54.7, 47.1, 39.8, 38.7 (t, *J* = 2.3 Hz), 22.0, 21.2;

HRMS: (ESI) calcd for $C_{41}H_{36}F_2NO_2S_2^+$ $[M+H]^+$ 676.2150; found 676.2123.

3-(1,1-difluoro-3-(3-methyl-5,6-diphenyl-1-tosyl-2,3,4,7-tetrahydro-1*H*-azepin-3-yl)prop-1-en-2-yl)-9-phenyl-9*H*-carbazole (43)

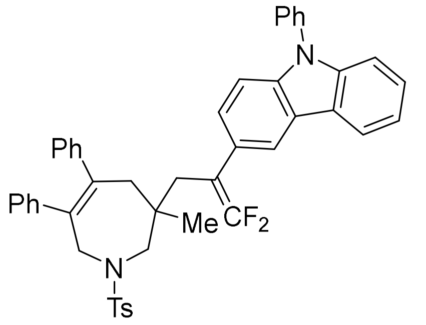

Chemical Formula: $C_{47}H_{40}F_2N_2O_2S$
Exact Mass: 734.2779

43 was prepared according to general procedure **2.1** using 4-methyl-*N*-(2-methylallyl)-*N*-(3-phenylprop-2-yn-1-yl)benzenesulfonamide (0.2 mmol, 67.9 mg), bromobenzene (0.2 mmol, 31.4 mg) and 9-phenyl-3-(3,3,3-trifluoroprop-1-en-2-yl)-9*H*-carbazole (0.1 mmol, 33.7 mg), and was purified by silica gel column chromatography (PE/EtOAc = 2/1) to obtain **43** as white solid (45.6 mg, 62% yield).

^{1}H NMR (600 MHz, $CDCl_3$) δ 8.16 (d, *J* = 7.7 Hz, 1H), 8.05 (d, *J* = 1.4 Hz, 1H), 7.67-7.62 (m, 2H), 7.62-7.58 (m, 2H), 7.53-7.44 (m, 4H), 7.39-7.32 (m, 2H), 7.20-7.14 (m, 2H), 7.11-7.01 (m, 8H), 6.93-6.87 (m, 2H), 6.69 (d, *J* = 7.9 Hz, 2H), 4.34 (d, *J* = 15.2 Hz, 1H), 3.57 (d, *J* = 15.3 Hz, 1H), 3.46 (d, *J* = 12.5 Hz, 1H), 2.83 (d, *J* = 14.0 Hz, 1H), 2.60-2.51 (m, 3H), 2.44 (d, *J* = 12.5 Hz, 1H), 2.10 (s, 3H), 1.11 (s, 3H);

^{19}F NMR (565 MHz, $CDCl_3$) δ -88.96 (d, *J* = 39.6 Hz), -91.45 (d, *J* = 39.4 Hz);

^{13}C NMR (151 MHz, $CDCl_3$) δ 155.6 (dd, *J* = 296.7, 289.0 Hz), 144.5, 142.9, 141.9, 141.2, 140.1, 139.9, 137.4, 135.4, 134.5, 130.0, 129.5, 129.2, 129.0, 127.8, 127.7, 127.7, 126.92, 126.90, 126.7 (dd, *J* = 4.0, 2.4 Hz), 126.6 (t, *J* = 3.9 Hz), 126.4, 126.3, 126.1, 123.6, 123.1, 120.6, 120.3, 120.1 (t, *J* = 2.9 Hz), 110.0, 109.9, 89.8 (dd, *J* = 21.0, 14.9 Hz), 62.2, 54.6, 47.3, 40.2, 38.7 (t, *J* = 2.5 Hz), 21.8, 21.2;

HRMS: (ESI) calcd for $C_{47}H_{41}F_2N_2O_2S^+$[M+H]$^+$ 735.2851; found 735.2831.

methyl 3-(3-methyl-5,6-diphenyl-1-tosyl-2,3,4,7-tetrahydro-1H-azepin-3-yl) propanoate (44)

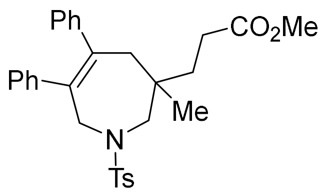

Chemical Formula: $C_{30}H_{33}NO_4S$
Exact Mass: 503.2130

44 was prepared according to general procedure **2.1** using 4-methyl-N-(2-methylallyl)-N-(3-phenylprop-2-yn-1-yl)benzenesulfonamide (0.1 mmol, 33.9 mg), bromobenzene (0.2 mmol, 31.4 mg) and methyl acrylate (0.2 mmol, 19.7 mg) and was purified by silica gel column chromatography (PE/EtOAc = 10/1) to obtain **44** as colorless oil (15.1 mg, 30% yield).

^{1}H NMR (600 MHz, $CDCl_3$) δ 7.71 – 7.66 (m, 2H), 7.31 (d, *J* = 8.0 Hz, 2H), 7.11 – 7.03 (m, 8H), 6.96 – 6.92 (m, 2H), 4.21 (d, *J* = 15.4 Hz, 1H), 4.05 (d, *J* = 15.4 Hz, 1H), 3.62 (s, 3H), 3.32 (d, *J* = 12.7 Hz, 1H), 3.05 – 3.00 (m, 1H), 2.70 – 2.61 (m, 2H), 2.44 (s, 3H), 2.31 (ddd, *J* = 15.6, 11.3, 5.6 Hz, 1H), 2.10 (ddd, *J* = 15.9, 11.2, 5.2 Hz, 1H), 1.83 (ddd, *J* = 13.9, 11.2, 5.6 Hz, 1H), 1.75 (ddd, *J* = 14.0, 11.3, 5.2 Hz, 1H), 0.95 (s, 3H).

^{13}C NMR (151 MHz, $CDCl_3$) δ 174.1, 144.4, 143.4, 141.8, 139.8, 135.6, 135.2, 129.8, 129.6, 129.0, 127.9, 127.8, 127.3, 126.4, 126.2, 61.1, 54.7, 51.6, 46.6, 36.2, 33.0, 28.8, 23.4, 21.5.

HRMS: (ESI) calcd for $C_{30}H_{34}NO_4S^+$[M+H]$^+$ 504.2203; found 504.2210.

3-methyl-5,6-diphenyl-3-(2-(phenylsulfonyl)ethyl)-1-tosyl-2,3,4,7-tetrahydro-1*H*-azepine (45)

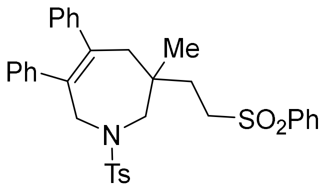

Chemical Formula: $C_{34}H_{35}NO_4S_2$
Exact Mass: 585.2007

45 was prepared according to general procedure **2.1** using 4-methyl-*N*-(2-methylallyl)-*N*-(3-phenylprop-2-yn-1-yl)benzenesulfonamide (0.2 mmol, 67.9 mg), bromobenzene

(0.2 mmol, 31.4 mg) and (vinylsulfonyl)benzene (0.1 mmol, 16.8 mg) and was purified by silica gel column chromatography (PE/EtOAc = 4/1) to obtain **45** as white solid (17.6 mg, 30% yield).

^{1}H NMR (600 MHz, $CDCl_3$) δ 7.91-7.85 (m, 2H), 7.69-7.62 (m, 3H), 7.58 (t, *J* = 7.8 Hz, 2H), 7.31 (d, *J* = 8.1 Hz, 2H), 7.12-7.01 (m, 6H), 6.94 (dd, *J* = 6.7, 3.0 Hz, 2H), 6.78-6.69 (m, 2H), 4.26 (d, *J* = 15.4 Hz, 1H), 3.73 (d, *J* = 15.4 Hz, 1H), 3.49 (d, *J* = 12.9 Hz, 1H), 3.40-3.32 (m, 1H), 2.92-2.85 (m, 1H), 2.72 (d, *J* = 14.2 Hz, 1H), 2.68 (d, *J* = 12.9 Hz, 1H), 2.50 (d, *J* = 14.2 Hz, 1H), 2.43 (s, 3H), 1.94-1.85 (m, 1H), 1.70-1.64 (m, 1H), 0.93 (s, 3H);

^{13}C NMR (151 MHz, $CDCl_3$) δ 143.9, 143.6, 141.6, 139.1, 138.5, 136.1, 134.9, 133.6, 129.9, 129.5, 129.2, 128.8, 128.3, 128.0, 127.7, 127.2, 126.5, 126.3, 60.7, 54.7, 51.3, 47.5, 35.9, 29.7, 24.4, 21.5;

HRMS: (ESI) calcd for $C_{34}H_{36}NO_4S_2^+$[M+H]$^+$ 586.2083; found 586.2072.

4-(3-(5-(diphenylmethylene)-3,3-bis(methoxymethyl)-1-methylcyclohexyl)-1,1-difluoroprop-1-en-2-yl)benzonitrile (46)

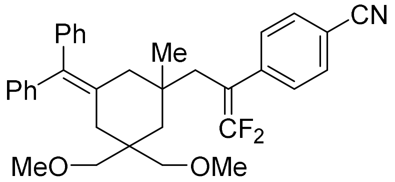

Chemical Formula: $C_{34}H_{35}F_2NO_2$
Exact Mass: 527.2636

46 was prepared according to general procedure **2.2** using (4,4-bis(methoxymethyl)-6-methylhept-6-en-1-yn-1-yl)benzene (0.2 mmol, 54.4 mg), bromobenzene (0.2 mmol, 31.4 mg) and 4-(3,3,3-trifluoroprop-1-en-2-yl)benzonitrile (0.1 mmol, 19.7 mg), and was purified by silica gel column chromatography (PE/EtOAc = 20/1) to obtain **46** as colorless oil (33.2 mg, 63% yield).

^{1}H NMR (600 MHz, $CDCl_3$) δ 7.54-7.45 (m, 2H), 7.27-7.23 (m, 4H), 7.22 (d, *J* = 7.0 Hz, 1H), 7.18-7.13 (m, 3H), 7.09-7.05 (m, 2H), 7.00-6.97 (m, 2H), 3.31 (d, J = 9.2 Hz, 1H), 3.25 (s, 3H), 3.23 (s, 3H), 3.10 (d, *J* = 9.2 Hz, 1H), 3.04 (d, *J* = 8.9 Hz, 2H), 2.97

(d, J = 8.9 Hz, 1H), 2.39 (dd, *J* = 14.2, 2.5 Hz, 1H), 2.27-2.21 (m, 2H), 1.92 (m, 1H), 1.82 (d, *J* = 14.2 Hz, 1H), 1.51-1.46 (m, 2H), 1.24 (d, *J* = 14.5 Hz, 2H), 0.86 (s, 3H);

^{19}F NMR (565 MHz, $CDCl_3$) δ -86.77 (d, *J* = 33.7 Hz), -89.30 (d, *J* = 33.7 Hz);

^{13}C NMR (151 MHz, $CDCl_3$) δ 154.3 (dd, *J* = 292.6 Hz, 290.8 Hz), 142.5, 140.6 (dd, *J* = 5.3, 2.6 Hz), 138.9, 132.9, 132.0, 129.1 (t, *J* = 2.7 Hz), 129.0 (d, *J* = 1.9 Hz), 128.0 (d, *J* = 3.2 Hz), 126.2, 126.1, 118.7, 110.5, 89.8 (dd, *J* = 22.5, 13.0 Hz), 79.7, 75.3, 59.1, 58.7, 43.2, 42.7, 41.3, 40.3, 37.7 (t, *J* = 2.1 Hz), 34.5, 24.8;

HRMS: (ESI) calcd for $C_{34}H_{36}F_2NO_2^+$[M+H]$^+$ 528.2639; found 528.2639.

4-(3-(5-(diphenylmethylene)-3-methyl-1-tosylpiperidin-3-yl)-1,1-difluoroprop-1-en-2-yl)benzonitrile (47)

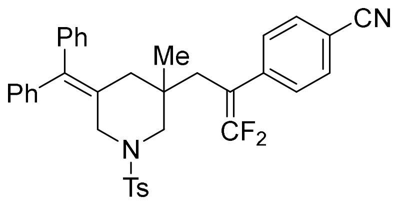

Chemical Formula: $C_{36}H_{32}F_2N_2O_2S$
Exact Mass: 594.2153

47 was prepared according to general procedure **2.2** using 4-methyl-*N*-(2-methylallyl)-*N*-(3-phenylprop-2-yn-1-yl)benzenesulfonamide (0.2 mmol, 67.9 mg), bromobenzene (0.2 mmol, 31.4 mg) and 4-(3,3,3-trifluoroprop-1-en-2-yl)benzonitrile (0.1 mmol, 19.7 mg), and was purified by silica gel column chromatography (PE/EtOAc = 10/1) to obtain **47** as white solid (34.4 mg, 58% yield).

^{1}H NMR (600 MHz, $CDCl_3$) δ 7.58-7.53 (m, 2H), 7.44-7.40 (m, 2H), 7.34-7.30 (m, 2H), 7.30-7.27 (m, 3H), 7.26-7.24 (m, 3H), 7.17 (ddd, *J* = 8.0, 2.8, 1.2 Hz, 4H), 7.00-6.94 (m, 2H), 3.85 (d, *J* = 12.3 Hz, 1H), 3.26 (d, *J* = 12.3 Hz, 1H), 2.82 (d, *J* = 11.2 Hz, 1H), 2.46 (d, *J* = 11.1 Hz, 1H), 2.45-2.40 (m, 4H), 2.37 (dt, *J* = 14.6, 2.3 Hz, 1H), 1.92 (d, *J* = 13.6 Hz, 1H), 1.82 (d, *J* = 13.6 Hz, 1H), 0.77 (s, 3H);

^{19}F NMR (565 MHz, $CDCl_3$) δ -87.26 (d, *J* = 34.8 Hz), -89.53 (d, *J* = 34.8 Hz);

^{13}C NMR (151 MHz, $CDCl_3$) δ 154.4 (dd, *J* = 292.8, 291.4 Hz), 143.5, 141.5, 141.5, 140.7, 139.8 (dd, *J* = 4.9, 2.8 Hz), 132.7, 132.2, 129.6, 129.5, 129.4, 129.1 (t, *J* = 2.8 Hz), 128.3, 128.2, 127.7, 127.3, 127.2, 126.9, 118.5, 111.0, 88.9 (dd, *J* = 22.3, 13.8 Hz),

56.4, 49.8, 41.8, 37.6 (t, J = 2.6 Hz), 36.7, 23.3, 21.5;

HRMS: (ESI) calcd for $C_{36}H_{33}F_2N_2O_2S^+$[M+H]$^+$ 595.2226; found 595.2222.

Dimethyl 3-(2-(4-cyanophenyl)-3,3-difluoroallyl)-5-(diphenylmethylene)-3-phenylcyclohexane-1,1-dicarboxylate (48)

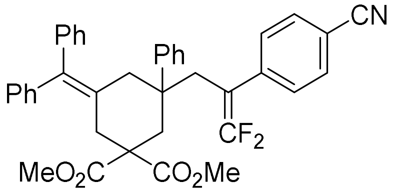

Chemical Formula: $C_{39}H_{33}F_2NO_4$
Exact Mass: 617.2378

48 was prepared according to general procedure **2.2** using dimethyl 2-(2-phenylallyl)-2-(3-phenylprop-2-yn-1-yl)malonate (0.2 mmol, 71.4 mg), bromobenzene (0.2 mmol, 31.4 mg) and 4-(3,3,3-trifluoroprop-1-en-2-yl)benzonitrile (0.1 mmol, 19.7 mg), and was purified by silica gel column chromatography (PE/EtOAc = 5/1) to obtain **48** as colorless oil (14.2 mg, 23% yield).

^{1}H NMR (600 MHz, $CDCl_3$) δ 7.33-7.30 (m, 2H), 7.20-7.16 (m, 4H), 7.14-7.10 (m, 4H), 7.06-6.98 (m, 3H), 6.88 (dd, J = 7.8, 1.7 Hz, 2H), 6.73-6.67 (m, 2H), 6.68-6.64 (m, 2H), 3.75 (s, 3H), 3.53 (dd, J = 17.0, 1.6 Hz, 1H), 3.19 (s, 3H), 3.14 (d, J = 14.8 Hz, 1H), 2.79 (s, 2H), 2.79-2.75 (m, 1H), 2.73 (d, J = 16.7 Hz, 1H), 2.68-2.64 (m, 1H), 2.58 (dt, J = 14.6, 2.6 Hz, 1H);

^{19}F NMR (565 MHz, $CDCl_3$) δ -86.20 (d, J = 30.9 Hz), -88.82 (d, J = 30.3 Hz);

^{13}C NMR (151 MHz, $CDCl_3$) δ 172.5, 171.8, 154.5 (dd, J = 292.6, 290.8 Hz), 142.5, 140.9, 140.8, 140.1 (dd, J = 4.9, 2.7 Hz), 139.8, 139.0, 132.2, 129.7, 129.5, 129.0 (t, J = 2.9 Hz), 128.8, 128.7, 128.1, 127.1, 126.9, 126.7, 126.3, 118.6, 110.7, 89.2 (dd, J = 22.4, 13.5 Hz), 54.7, 53.0, 52.4, 42.2, 42.0, 41.9, 36.9 (t, J = 2.3 Hz), 34.8;

HRMS: (ESI) calcd for $C_{39}H_{34}F_2NO_4^+$[M+H]$^+$ 618.2385; found 618.2384.

Trimethyl-3-(2-(4-cyanophenyl)-3,3-difluoroallyl)-5-(diphenylmethylene) cyclohexane-1,1,3-tricarboxylate (49)

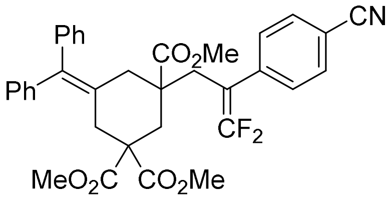

Chemical Formula: $C_{35}H_{31}F_2NO_6$
Exact Mass: 599.2119

49 was prepared according to general procedure **2.2** using trimethyl 7-phenylhept-1-en-6-yne-2,4,4-tricarboxylate (0.2 mmol, 68.8 mg), bromobenzene (0.2 mmol, 31.4 mg) and 4-(3,3,3-trifluoroprop-1-en-2-yl)benzonitrile (0.1 mmol, 19.7 mg), and was purified by silica gel column chromatography (PE/EtOAc = 5/1) to obtain **49** as colorless oil (34.7 mg, 58% yield).

^{1}H NMR (600 MHz, $CDCl_3$) δ 7.63-7.55 (m, 2H), 7.29 (dd, J = 8.3, 1.3 Hz, 2H), 7.10-6.95 (m, 6H), 6.91-6.86 (m, 2H), 6.81-6.77 (m, 2H), 3.72 (s, 3H), 3.68 (s, 3H), 3.42 (dd, J = 16.8, 1.7 Hz, 1H), 3.23 (s, 3H), 3.12 (d, J = 15.9 Hz, 1H), 3.01 (d, J = 16.6 Hz, 1H), 2.79-2.73 (m, 2H), 2.71 (d, J = 14.8 Hz, 1H), 2.64 (m, 1H), 2.45 (d, J = 14.8 Hz, 1H);

^{19}F NMR (565 MHz, $CDCl_3$) δ -85.72 (d, J = 29.1 Hz), -86.88 (d, J = 29.2 Hz);

^{13}C NMR (151 MHz, $CDCl_3$) δ 174.6, 172.6, 171.6, 154.8 (dd, J = 292.6, 290.8 Hz), 143.9, 143.2, 138.30 (dd, J = 4.3, 2.8 Hz), 137.0, 135.5, 132.0, 129.5 (t, J = 2.7 Hz), 129.03, 129.01, 127.9, 127.7, 126.2, 126.0, 118.5, 111.1, 88.6 (dd, J = 21.9, 15.1 Hz), 54.5, 52.9, 52.8, 51.7, 40.9, 39.6, 47.0 (t, J = 2.5 Hz), 38.4, 38.1;

HRMS: (ESI) calcd for $C_{35}H_{32}F_2NO_6^+$[M+H]$^+$ 600.2121; found 600.2117.

dimethyl 5-(bis(4-methoxyphenyl)methylene)-3-(2-(4-cyanophenyl)-3,3-difluoroallyl)-3-methylcyclohexane-1,1-dicarboxylate (50)

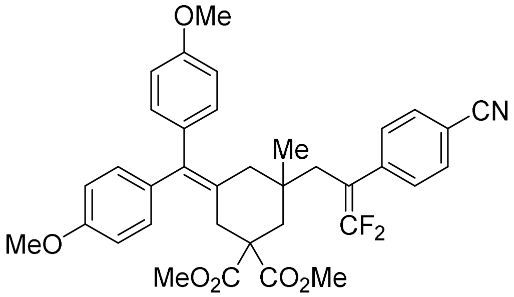

Chemical Formula: $C_{36}H_{35}F_2NO_6$
Exact Mass: 615.2432

50 was prepared according to general procedure **2.2** using dimethyl 2-(3-(4-methoxyphenyl)prop-2-yn-1-yl)-2-(2-methylallyl)malonate (0.2 mmol, 66.1 mg), 1-bromo-4-methoxybenzene (0.2 mmol, 37.4 mg) and 4-(3,3,3-trifluoroprop-1-en-2-yl)benzonitrile (0.1 mmol, 19.7 mg), and was purified by silica gel column chromatography (PE/EtOAc = 5/1) to obtain **50** as colorless oil (35.1 mg, 57% yield).

^{1}H NMR (600 MHz, $CDCl_3$) δ 7.54-7.47 (m, 2H), 7.22-7.18 (m, 2H), 7.10-7.04 (m, 2H), 6.85-6.79 (m, 4H), 6.79-6.75 (m, 2H), 3.83 (s, 3H), 3.76 (s, 3H), 3.63 (s, 3H), 3.62 (s, 3H), 3.31 – 3.24 (m, 1H), 2.42 (dd, J = 14.4, 2.5 Hz, 1H), 2.32-2.25 (m, 1H), 2.22-2.14 (m, 1H), 2.04 (d, J = 14.3 Hz, 1H), 1.96-1.89 (m, 2H), 1.47 (d, J = 13.8 Hz, 1H), 0.70 (s, 3H);

^{19}F NMR (565 MHz, $CDCl_3$) δ -86.22 (d, J = 32.8 Hz), -88.71 (dd, J = 32.4, 2.9 Hz);

^{13}C NMR (151 MHz, $CDCl_3$) δ 172.5, 171.8, 157.93, 157.89, 154.5 (dd, J = 292.9, 290.4 Hz), 140.1 (dd, J = 5.0, 2.7 Hz), 139.3, 135.2, 134.8, 132.1, 130.1, 129.8, 129.1, 129.0 (t, J = 3.1 Hz), 118.6, 113.4, 113.3, 110.7, 89.3 (dd, J = 22.5, 13.4 Hz), 55.2, 55.1, 54.7, 52.9, 52.3, 42.2, 42.1, 41.9, 36.9 (t, J = 2.6 Hz), 34.9, 22.8.

HRMS: (ESI) calcd for $C_{36}H_{36}F_2NO_6^+$ $[M+H]^+$ 616.2505; found 616.2510.

dimethyl 5-(bis(4-chlorophenyl)methylene)-3-(2-(4-cyanophenyl)-3,3-difluoroallyl) -3-methylcyclohexane-1,1-dicarboxylate (51)

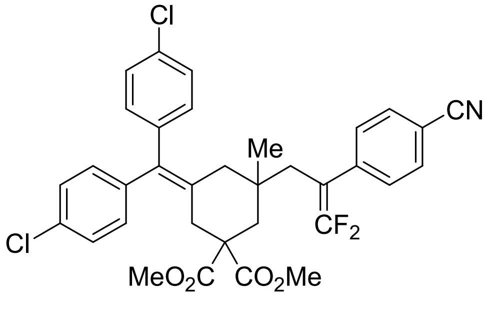

Chemical Formula: $C_{34}H_{29}Cl_2F_2NO_4$
Exact Mass: 623.1442

51 was prepared according to general procedure **2.2** using dimethyl 2-(3-(4-chlorophenyl)prop-2-yn-1-yl)-2-(2-methylallyl)malonate (0.2 mmol, 66.8 mg), 1- 1-bromo-4-chlorobenzene (0.2 mmol, 38.2 mg) and 4-(3,3,3-trifluoroprop-1-en-2-yl)benzonitrile (0.1 mmol, 19.7 mg), and was purified by silica gel column

chromatography (PE/EtOAc = 15/1) to obtain **51** as colorless oil (38.1 mg, 61% yield).
^{1}H NMR (600 MHz, $CDCl_3$) δ 7.55 (d, *J* = 8.3 Hz, 2H), 7.28-7.25 (m, 2H), 7.24-7.21 (m, 2H), 7.21-7.17 (m, 2H), 7.15-7.10 (m, 2H), 6.85 (d, *J* = 8.4 Hz, 2H), 3.64 (s, 3H), 3.63 (s, 3H), 3.22-3.17 (m, 1H), 2.43 (dd, *J* = 14.4, 2.5 Hz, 1H), 2.33-2.27 (m, 1H), 2.21-2.16 (m, 1H), 2.02 (dd, *J* = 14.3, 1.3 Hz, 1H), 1.94 (d, *J* = 14.2 Hz, 1H), 1.84-1.80 (m, 1H), 1.46 (d, *J* = 13.8 Hz, 1H), 0.68 (s, 3H);
^{19}F NMR (565 MHz, $CDCl_3$) δ -85.91 (d, *J* = 32.0 Hz), -88.38 (dd, *J* = 32.0, 3.5 Hz);
^{13}C NMR (151 MHz, $CDCl_3$) δ 172.2, 171.7, 154.6 (dd, *J* = 293.5, 290.7 Hz), 140.5, 139.9 (dd, *J* = 4.6, 2.9 Hz), 139.8, 137.8, 132.6, 132.5, 132.2, 131.1, 130.4, 130.1, 128.9 (t, *J* = 2.5 Hz), 128.4, 128.3, 118.5, 110.9, 89.1 (dd, *J* = 22.4, 13.5 Hz), 54.5, 53.0, 52.4, 42.1, 42.0, 41.8, 36.9 (t, *J* = 2.6 Hz), 34.8, 22.5.
HRMS: (ESI) calcd for $C_{34}H_{30}Cl_2F_2NO_4^+$[M+H]$^+$ 624.1514; found 624.1510.

Dimethyl-3-(2-(4-cyanophenyl)-3,3-difluoroallyl)-5-((4-methoxyphenyl)(phenyl)methylene)-3-methylcyclohexane-1,1-dicarboxylate (52)

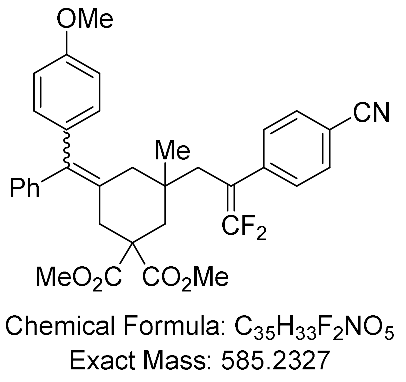

52 was prepared according to general procedure **2.2** using dimethyl 2-(2-methylallyl)-2-(3-phenylprop-2-yn-1-yl)malonate (0.2 mmol, 60.0 mg), 1-bromo-4-methoxybenzene (0.2 mmol, 37.4 mg) and 4-(3,3,3-trifluoroprop-1-en-2-yl)benzonitrile (0.1 mmol, 19.7 mg), and was purified by silica gel column chromatography (PE/EtOAc = 10/1) to obtain **52** as colorless oil (38.0 mg, 65% yield).
^{1}H NMR (600 MHz, $CDCl_3$) δ 7.49 (d, *J* = 8.4 Hz, 2H), 7.26-7.19 (m, 3H), 7.18-7.13 (m, 2H), 7.10 (d, *J* = 8.8 Hz, 2H), 6.92 (dd, *J* = 7.8, 1.7 Hz, 2H), 6.82 (d, *J* = 8.8 Hz, 2H), 3.77 (s, 3H), 3.64 (s, 3H), 3.62 (s, 3H), 3.29 (m, 1H), 2.42 (dd, *J* = 14.3, 2.5 Hz,

1H), 2.30-2.24 (m, 1H), 2.19 (m, 1H), 2.04 (dd, *J* = 14.3, 1.3 Hz, 1H), 1.96-1.88 (m, 2H), 1.46 (d, *J* = 13.7 Hz, 1H), 0.70 (s, 3H);

^{19}F NMR (565 MHz, $CDCl_3$) δ -86.22 (d, *J* = 32.7 Hz), -88.76 (d, *J* = 32.4 Hz);

^{13}C NMR (151 MHz, $CDCl_3$) δ 172.6, 171.9, 158.0, 154.5 (dd, *J* = 292.8, 290.7 Hz), 142.9, 140.2 (dd, *J* = 4.9, 2.7 Hz), 139.8, 134.5, 132.2, 130.2, 129.2, 129.0 (t, *J* = 2.8 Hz), 128.8, 128.1, 126.2, 118.7, 113.4, 110.7, 89.3 (dd, *J* = 22.6, 13.4 Hz), 55.1, 54.7, 53.0, 52.4, 42.2, 42.1, 42.0, 36.9 (t, *J* = 2.5 Hz), 34.8, 22.8;

HRMS: (ESI) calcd for $C_{35}H_{34}F_2NO_5^+$[M+H]$^+$ 586.2332; found 586.2341.

Dimethyl-5-([1,1'-biphenyl]-4-yl(phenyl)methylene)-3-(2-(4-cyanophenyl)-3,3-**difluoroallyl)-3-methylcyclohexane-1,1-dicarboxylate (53)**

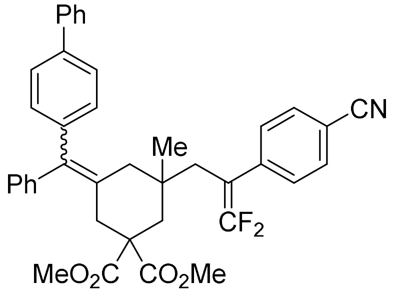

Chemical Formula: $C_{40}H_{35}F_2NO_4$
Exact Mass: 631.2534

53 was prepared according to general procedure **2.2** using dimethyl 2-(2-methylallyl)-2-(3-phenylprop-2-yn-1-yl)malonate (0.2 mmol, 60.0 mg), 4-bromo-1,1'-biphenyl (0.2 mmol, 46.6 mg) and 4-(3,3,3-trifluoroprop-1-en-2-yl)benzonitrile (0.1 mmol, 19.7 mg), and was purified by silica gel column chromatography (PE/EtOAc = 10/1) to obtain **53** as colorless oil (48.0 mg, 76% yield).

^{1}H NMR (600 MHz, $CDCl_3$) δ 7.59-7.55 (m, 2H), 7.55-7.47 (m, 4H), 7.41 (t, *J* = 7.8 Hz, 2H), 7.34-7.29 (m, 1H), 7.27 (s, 4H), 7.26-7.23 (m, 1H), 7.16 (dd, *J* = 8.4, 1.4 Hz, 2H), 6.98 (dd, *J* = 7.9, 1.7 Hz, 2H), 3.66 (s, 3H), 3.63 (s, 3H), 3.33 (m, 1H), 2.43 (dd, *J* = 14.3, 2.5 Hz, 1H), 2.32-2.27 (m, 1H), 2.21 (m, 1H), 2.10 (dd, *J* = 14.3, 1.3 Hz, 1H), 1.94 (m, 2H), 1.49 (d, *J* = 13.8 Hz, 1H), 0.72 (s, 3H);

^{19}F NMR (565 MHz, $CDCl_3$) δ -86.16 (d, *J* = 32.6 Hz), -88.69 (d, *J* = 32.6 Hz);

^{13}C NMR (151 MHz, $CDCl_3$) δ 172.5, 171.8, 154.5 (dd, *J* = 292.7, 290.9 Hz), 142.5,

140.9, 140.8, 140.1 (dd, J = 4.9, 2.7 Hz), 139.8, 139.0, 132.2, 129.7, 129.5, 129.0 (t, J = 3.0 Hz), 128.8, 128.7, 128.1, 127.1, 126.9, 126.7, 126.3, 118.6, 110.7, 89.2 (dd, J = 22.4, 13.5 Hz), 54.7, 53.0, 52.4, 42.2, 42.0, 41.9, 36.9 (t, J = 2.6 Hz), 34.8, 22.7;

HRMS: (ESI) calcd for $C_{40}H_{36}F_2NO_4^+$[M+H]$^+$ 632.2542; found 632.2548.

Dimethyl-5-((4-chlorophenyl)(phenyl)methylene)-3-(2-(4-cyanophenyl)-3,3-difluoroallyl)-3-methylcyclohexane-1,1-dicarboxylate (54)

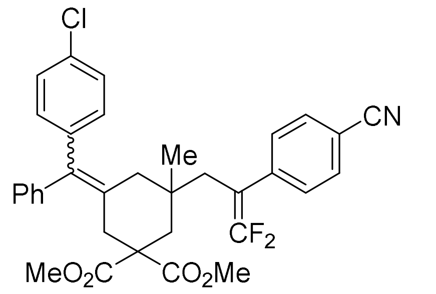

Chemical Formula: $C_{34}H_{30}ClF_2NO_4$
Exact Mass: 589.1831

54was prepared according to general procedure **2.2** using dimethyl 2-(2-methylallyl)-2-(3-phenylprop-2-yn-1-yl)malonate (0.2 mmol, 60.0 mg), 1-bromo-4-chlorobenzene (0.2 mmol, 38.2 mg) and 4-(3,3,3-trifluoroprop-1-en-2-yl)benzonitrile (0.1 mmol, 19.7 mg), and was purified by silica gel column chromatography (PE/EtOAc = 10/1) to obtain **54** as colorless oil (38.9 mg, 66% yield).

^{1}H NMR (600 MHz, $CDCl_3$) δ 7.55 (d, J = 8.4 Hz, 2H), 7.29 (t, J = 7.6 Hz, 2H), 7.20 (m, 5H), 7.17-7.13 (m, 2H), 6.87 (d, J = 8.3 Hz, 2H), 3.63 (s, 3H), 3.61 (s, 3H), 3.24 (dd, J = 14.3, 1.8 Hz, 1H), 2.43 (dd, J = 14.3, 2.5 Hz, 1H), 2.32-2.26 (m, 1H), 2.19 (m, 1H), 2.04 (dd, J = 14.3, 1.3 Hz, 1H), 1.93 (d, J = 14.1 Hz, 1H), 1.82 (m, 1H), 1.47 (d, J = 13.7 Hz, 1H), 0.69 (s, 3H);

^{19}F NMR (565 MHz, $CDCl_3$) δ -85.97 (d, J = 32.1 Hz), -88.49 (d, J = 32.1 Hz);

^{13}C NMR (151 MHz, $CDCl_3$) δ 172.3, 171.7, 154.6 (dd, J = 293.3, 291.1 Hz), 141.4, 140.9, 140.0 (dd, J = 4.9, 2.7 Hz), 139.0, 132.2, 132.2, 130.3, 130.1, 129.0, 128.9 (t, J = 2.8 Hz),128.3, 128.1, 126.7, 118.5, 110.8, 89.2 (dd, J = 22.3, 13.3 Hz), 54.6, 53.0, 52.3, 42.1, 42.1, 41.8, 36.9 (t, J = 2.5 Hz), 34.7, 22.6;

HRMS: (ESI) calcd for $C_{34}H_{31}ClF_2NO_4^+$[M+H]$^+$ 590.1833; found 590.1833.

Dimethyl-3-(2-(4-cyanophenyl)-3,3-difluoroallyl)-5-((3,5-dimethylphenyl)(phenyl)methylene)-3-methylcyclohexane-1,1-dicarboxylate (55)

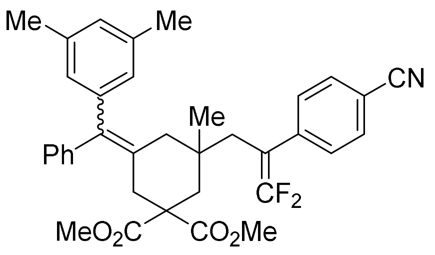

55 was prepared according to general procedure **2.2** using dimethyl 2-(2-methylallyl)-2-(3-phenylprop-2-yn-1-yl)malonate (0.2 mmol, 60.0 mg), 1-bromo-3,5-dimethylbenzene (0.2 mmol, 37.0 mg) and 4-(3,3,3-trifluoroprop-1-en-2-yl)benzonitrile (0.1 mmol, 19.7 mg), and was purified by silica gel column chromatography (PE/EtOAc = 10/1) to obtain **55** as colorless oil (38.5 mg, 66% yield, *Z*/*E* = 1/1).

^{1}H NMR (600 MHz, $CDCl_3$) δ 7.49 (dd, *J* = 8.4, 2.1 Hz, 2H), 7.28 (dd, *J* = 8.7, 6.8 Hz, 1H), 7.25-7.21 (m, 1.5H), 7.19-7.14 (m, 3.5H), 6.96-6.93 (m, 1H), 6.86 (s, 0.5H), 6.82 (s, 0.5H), 6.77 (d, *J* = 1.6 Hz, 1H), 6.57 (d, *J* = 1.7 Hz, 1H), 3.64 (s, 1.5H), 3.63 (s, 1.5 H), 3.62 (s, 1.5H), 3.61 (s, 1.5H), 3.24 (m, 1H), 2.42 (m, 1H), 2.27 (s, 3H), 2.26 (s, 3H), 2.22-2.17 (m, 1H), 2.05 (m, 1H), 1.94-1.87 (m, 2H), 1.45 (dd, *J* = 13.7, 5.2 Hz, 1H), 0.71 (s, 1.5H), 0.70 (s, 1.5H);

^{19}F NMR (565 MHz, $CDCl_3$) δ -86.18 (d, *J* = 29.5 Hz), -86.24 (d, *J* = 28.3 Hz), -88.78 (d, *J* = 32.9 Hz), -88.86 (d, *J* = 32.8 Hz);

^{13}C NMR (151 MHz, $CDCl_3$) δ 172.6, 172.5, 171.77, 171.75, 154.5 (dd, *J* = 292.9, 290.8 Hz), 142.7, 142.4, 142.0, 141.8, 140.34, 140.33, 140.2 (dd, *J* = 4.7, 2.6 Hz), 137.4, 137.2, 132.2, 132.1, 129.09, 129.07, 128.96 (q, *J* = 3.5, 3.0 Hz), 128.6, 128.2, 128.0, 127.9, 127.8, 126.7, 126.4, 126.3, 126.1, 118.6, 118.6, 110.7, 110.7, 89.3 (dd, *J* = 22.5, 13.4 Hz), 54.8, 54.7, 52.93, 52.91, 52.3, 42.18, 42.16, 42.01, 42.95, 36.9 (dd, *J* = 5.4, 2.6 Hz), 34.71, 34.68, 22.91, 22.87, 21.4, 21.3;

HRMS: (ESI) calcd for $C_{36}H_{36}F_2NO_4^+$[M+H]$^+$ 584.2543; found 584.2539.

Dimethyl-3-(2-(4-cyanophenyl)-3,3-difluoroallyl)-3-methyl-5-(phenyl(3,4,5-trimethoxyphenyl)methylene)cyclohexane-1,1-dicarboxylate (56)

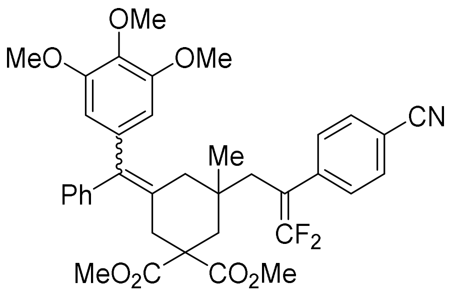

Chemical Formula: $C_{37}H_{37}F_2NO_7$
Exact Mass: 645.2538

56 was prepared according to general procedure **2.2** using dimethyl 2-(2-methylallyl)-2-(3-phenylprop-2-yn-1-yl)malonate (0.2 mmol, 60.0 mg), 5-bromo-1,2,3-trimethoxybenzene (0.2 mmol, 49.4 mg) and 4-(3,3,3-trifluoroprop-1-en-2-yl)benzonitrile (0.1 mmol, 19.7 mg), and was purified by silica gel column chromatography (PE/EtOAc = 5/1) to obtain **56** as colorless oil (36.8 mg, 57% yield).

^{1}H NMR (600 MHz, $CDCl_3$) δ 7.58-7.52 (m, 2H), 7.31 (dd, *J* = 8.1, 7.1 Hz, 2H), 7.22 (dd, *J* = 7.7, 6.3 Hz, 3H), 7.19-7.15 (m, 2H), 6.15 (s, 2H), 3.86 (s, 3H), 3.76 (s, 6H), 3.64 (s, 3H), 3.61 (s, 3H), 3.24 (d, *J* = 14.2 Hz, 1H), 2.43 (dd, *J* = 14.3, 2.5 Hz, 1H), 2.31-2.26 (m, 1H), 2.20 (m, 1H), 2.02 (dd, *J* = 14.2, 1.3 Hz, 1H), 1.95-1.89 (m, 2H), 1.42 (d, *J* = 13.7 Hz, 1H), 0.71 (s, 3H);

^{19}F NMR (565 MHz, $CDCl_3$) δ -85.93 (d, *J* = 31.9 Hz), -88.55 (d, *J* = 32.2 Hz);

^{13}C NMR (151 MHz, $CDCl_3$) δ 172.4, 171.8, 154.5 (dd, *J* = 291.9, 289.6 Hz), 152.9, 141.5, 140.2 (dd, *J* = 4.9, 2.4 Hz), 140.1, 138.2, 136.3, 132.2, 129.8, 129.0 (t, *J* = 3.1 Hz), 128.8, 128.0, 126.6, 118.5, 110.8, 105.5, 89.2 (dd, *J* = 22.7, 13.5 Hz), 60.8, 56.0, 54.7, 53.0, 52.3, 42.3, 42.0, 41.9, 37.0 (d, *J* = 2.6 Hz), 34.6, 22.5;

HRMS: (ESI) calcd for $C_{37}H_{38}F_2NO_7^+$[M+H]$^+$ 646.2546; found 646.2541.

Dimethyl-3-(2-(4-cyanophenyl)-3,3-difluoroallyl)-3-methyl-5-(phenyl(4-(trifluoromethyl)phenyl)methylene)cyclohexane-1,1-dicarboxylate (58)

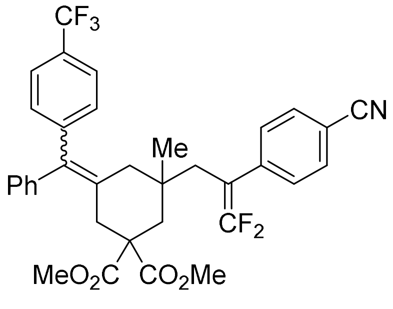

Chemical Formula: $C_{35}H_{30}F_5NO_4$
Exact Mass: 623.2095

58 was prepared according to general procedure **2.2** using dimethyl 2-(2-methylallyl)-2-(3-phenylprop-2-yn-1-yl)malonate (0.2 mmol, 60.0 mg), 1-bromo-4-(trifluoromethyl)benzene (0.2 mmol, 55.0 mg) and 4-(3,3,3-trifluoroprop-1-en-2-yl)benzonitrile (0.1 mmol, 19.7 mg), and was purified by silica gel column chromatography (PE/EtOAc = 5/1) to obtain **58** as colorless oil (36.8 mg, 59% yield).

^{1}H NMR (600 MHz, $CDCl_3$) δ 7.51 (dd, J = 8.3, 4.0 Hz, 4H), 7.31 (t, J = 7.6 Hz, 2H), 7.24-7.20 (m, 1H), 7.18 (m, 4H), 7.08 (d, J = 8.0 Hz, 2H), 3.65 (s, 3H), 3.62 (s, 3H), 3.26 (m, 1H), 2.44 (dd, J = 14.4, 2.5 Hz, 1H), 2.33-2.27 (m, 1H), 2.21 (dt, J = 14.2, 2.1 Hz, 1H), 2.06 (dd, J = 14.3, 1.3 Hz, 1H), 1.94 (d, J = 14.2 Hz, 1H), 1.81 (dt, J = 13.7, 2.0 Hz, 1H), 1.51 (d, J = 13.8 Hz, 1H), 0.71 (s, 3H);

^{13}C NMR (151 MHz, $CDCl_3$) δ 172.3, 171.6, 154.6 (dd, J = 292.8, 290.7 Hz), 146.2, 141.0, 140.1 (dd, J = 4.9, 2.7 Hz), 138.9, 132.2, 130.8, 129.1, 129.0, 128.8 (t, J = 2.8 Hz), 128.5 (q J = 32.5 Hz), 128.2, 126.9, 125.1 (q, J = 3.7 Hz), 123.3, 118.4, 110.9, 89.1 (dd, J = 22.5, 13.3 Hz), 54.6, 53.0, 52.4, 42.0, 41.8, 37.0 (t, J = 2.6 Hz), 34.7, 22.7;

^{19}F NMR (565 MHz, $CDCl_3$) δ -62.26, -85.71 (d, J = 31.6 Hz), -88.34 (d, J = 31.7 Hz);

HRMS: (ESI) calcd for $C_{35}H_{31}F_5NO_4^+$[M+H]$^+$ 624.2104; found 624.2110.

Dimethyl-3-(2-(4-cyanophenyl)-3,3-difluoroallyl)-3-methyl-5-(phenyl(9-phenyl-9*H*-carbazol-3-yl)methylene)cyclohexane-1,1-dicarboxylate (59)

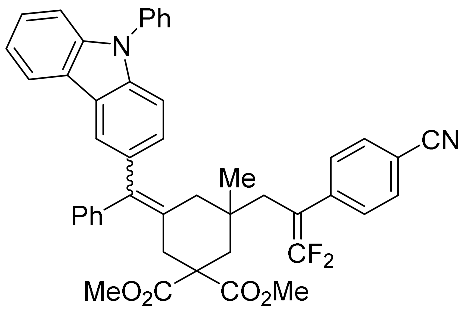

Chemical Formula: $C_{46}H_{38}F_2N_2O_4$
Exact Mass: 720.2800

59 was prepared according to general procedure **2.2** using dimethyl 2-(2-methylallyl)-2-(3-phenylprop-2-yn-1-yl)malonate (0.2 mmol, 60.0 mg), 2-bromo-9-phenyl-9*H*-carbazole (0.2 mmol, 64.2 mg) and 4-(3,3,3-trifluoroprop-1-en-2-yl)benzonitrile (0.1 mmol, 19.7 mg), and was purified by silica gel column chromatography (PE/EtOAc = 5/1) to obtain **59** as colorless oil (51.1 mg, 71% yield).

^{1}H NMR (600 MHz, $CDCl_3$) δ 8.03 (m, 1H), 7.73 (dd, *J* = 1.7, 0.7 Hz, 1H), 7.65-7.58 (m, 4H), 7.49-7.43 (m, 3H), 7.34-7.26 (m, 6H), 7.21-7.16 (m, 3H), 7.07-7.03 (m, 2H), 6.98 (dd, *J* = 8.4, 1.7 Hz, 1H), 3.67 (s, 3H), 3.63 (s, 3H), 3.35 (m, 1H), 2.41 (dd, *J* = 14.3, 2.6 Hz, 1H), 2.28 (m, 1H), 2.23 (m, 1H), 2.13 (m, 2H), 1.95 (d, *J* = 14.1 Hz, 1H), 1.56 (s, 1H), 0.79 (s, 3H);

^{19}F NMR (565 MHz, $CDCl_3$) δ -86.01 (d, *J* = 32.3 Hz), -88.66 (d, *J* = 32.3 Hz);

^{13}C NMR (151 MHz, $CDCl_3$) δ 172.6, 171.9, 154.5 (dd, *J* = 292.9, 290.8 Hz), 142.6, 141.0, 140.6, 140.0 (dd, *J* = 4.7, 2.6 Hz), 139.3, 137.6, 134.3, 132.0, 130.0, 129.5, 129.2, 128.9 (t, *J* = 3.0 Hz),127.9, 127.5, 127.2, 126.9, 126.3, 126.2, 123.2, 123.0, 120.2, 120.2, 118.4, 110.6, 109.9, 109.4, 89.2 (dd, *J* = 22.5, 13.4 Hz), 54.8, 52.9, 52.4, 42.2, 42.0, 42.0, 37.0 (t, *J* = 2.6 Hz), 35.0, 23.1;

HRMS: (ESI) calcd for $C_{46}H_{39}F_2N_2O_4^+$[M+H]$^+$ 721.2791; found 721.2797.

Dimethyl-3-(3,3-difluoro-2-(4-(methoxycarbonyl)phenyl)allyl)-5-(diphenylmethylene)-3-methylcyclohexane-1,1-dicarboxylate (60)

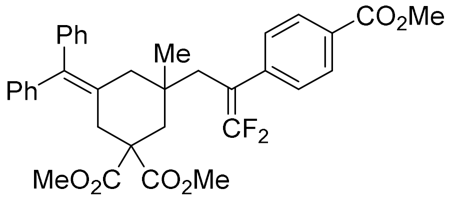

Chemical Formula: $C_{35}H_{34}F_2O_6$
Exact Mass: 588.2323

60 was prepared according to general procedure **2.2** using dimethyl 2-(2-methylallyl)-2-(3-phenylprop-2-yn-1-yl)malonate (0.2 mmol, 60.0 mg), bromobenzene (0.2 mmol, 31.4 mg) and methyl 4-(3,3,3-trifluoroprop-1-en-2-yl)benzoate (0.1 mmol, 23.0 mg), and was purified by silica gel column chromatography (PE/EtOAc = 5/1) to obtain **60** as colorless oil (43.5 mg, 74% yield).

^{1}H NMR (600 MHz, $CDCl_3$) δ 7.92-7.87 (m, 2H), 7.28 (dd, J = 8.3, 7.0 Hz, 2H), 7.22 (dd, J = 5.1, 1.9 Hz, 3H), 7.19 (dt, J = 8.3, 1.8 Hz, 3H), 7.15-7.11 (m, 2H), 6.96-6.91 (m, 2H), 3.93 (s, 3H), 3.62 (s, 3H), 3.60 (s, 3H), 3.24 (m, 1H), 2.42 (dd, J = 14.2, 2.6 Hz, 1H), 2.33-2.28 (m, 1H), 2.21 (m, 1H), 2.06 (dd, J = 14.3, 1.3 Hz, 1H), 2.00 (m, 1H), 1.93 (d, J = 14.1 Hz, 1H), 1.51 (d, J = 13.8 Hz, 1H), 0.69 (s, 3H);

^{19}F NMR (565 MHz, $CDCl_3$) δ -87.26 (d, J = 34.8 Hz), -89.53 (d, J = 34.8 Hz);

^{13}C NMR (151 MHz, $CDCl_3$) δ 172.5, 171.8, 166.7, 154.4 (dd, J = 289.8, 289.6 Hz), 142.4, 142.1, 140.1 (dd, J = 4.2, 2.3 Hz), 129.7, 129.7, 129.1, 128.7, 128.6, 128.3 (t, J = 2.8 Hz), 128.0, 127.9, 126.3, 126.2, 89.5 (dd, J = 21.8, 13.7 Hz), 54.7, 52.9, 52.3, 52.1, 42.3, 41.9, 41.8, 36.9 (t, J = 2.3 Hz), 34.8, 22.9;

HRMS: (ESI) calcd for $C_{35}H_{35}F_2O_6^+$[M+H]$^+$ 589.2344; found 589.2358.

Dimethyl-3-(2-(4-(dimethylcarbamoyl)phenyl)-3,3-difluoroallyl)-5-(diphenylmethylene)-3-methylcyclohexane-1,1-dicarboxylate (61)

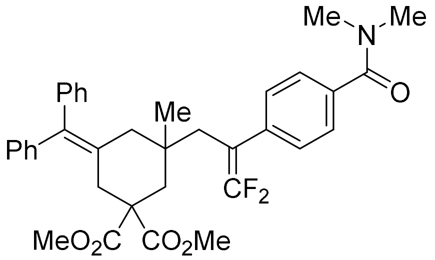

Chemical Formula: $C_{36}H_{37}F_2NO_5$
Exact Mass: 601.2640

61 was prepared according to general procedure **2.2** using dimethyl 2-(2-methylallyl)-2-(3-phenylprop-2-yn-1-yl)malonate (0.2 mmol, 60.0 mg), bromobenzene (0.2 mmol, 31.4 mg) and *N,N*-dimethyl-4-(3,3,3-trifluoroprop-1-en-2-yl)benzamide (0.1 mmol, 24.3 mg), and was purified by silica gel column chromatography (PE/EtOAc = 5/1) to obtain **61** as colorless oil (36.7 mg, 61% yield).

^{1}H NMR (600 MHz, $CDCl_3$) δ 7.31-7.26 (m, 4H), 7.24 (dd, *J* = 7.1, 1.2 Hz, 2H), 7.21-7.16 (m, 4H), 7.12-7.09 (m, 2H), 7.00-6.96 (m, 2H), 3.62 (s, 3H), 3.60 (s, 3H), 3.24 (d, *J* = 14.2 Hz, 1H), 3.11 (s, 3H), 2.96 (s, 3H), 2.42-2.37 (m, 1H), 2.31-2.26 (m, 1H), 2.20 (m, 1H), 2.09-2.04 (m, 2H), 1.91 (d, *J* = 14.1 Hz, 1H), 1.55 (d, *J* = 13.8 Hz, 1H), 0.71 (s, 3H);

^{19}F NMR (565 MHz, $CDCl_3$) δ -87.89 (d, *J* = 36.4 Hz), -90.32 (d, *J* = 37.1 Hz);

^{13}C NMR (151 MHz, $CDCl_3$) δ 172.5, 171.8, 171.2, 154.4 (dd, *J* = 291.2, 289.0 Hz), 142.5, 142.2, 139.9, 136.6 (dd, *J* = 4.7, 2.4 Hz), 134.8, 129.8, 129.1, 128.8, 128.3 (t, *J* = 2.7 Hz), 128.0, 127.9, 127.2, 126.3, 126.2, 89.4 (dd, *J* = 21.7, 14.1 Hz), 54.8, 52.8, 52.2, 42.5, 41.9, 41.8, 39.6, 37.0 (t, *J* = 2.5 Hz), 35.4, 34.9, 23.2;

HRMS: (ESI) calcd for $C_{36}H_{38}F_2NO_5^+$[M+H]$^+$ 602.2658; found 602.2684.

Dimethyl-3-(3,3-difluoro-2-(4-(methylsulfonyl)phenyl)allyl)-5-(diphenylmethylene)-3-methylcyclohexane-1,1-dicarboxylate (62)

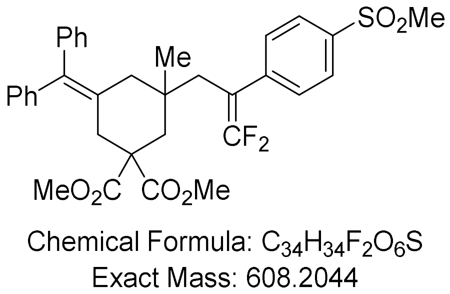

62 was prepared according to general procedure **2.2** using dimethyl 2-(2-methylallyl)-2-(3-phenylprop-2-yn-1-yl)malonate (0.2 mmol, 60.0 mg), bromobenzene (0.2 mmol, 31.4 mg) and 1-(methylsulfonyl)-4-(3,3,3-trifluoroprop-1-en-2-yl)benzene (0.1 mmol, 25.0 mg), and was purified by silica gel column chromatography (PE/EtOAc = 3/1) to obtain **62** as colorless oil (47.4 mg, 78% yield).

^{1}H NMR (600 MHz, $CDCl_3$) δ 7.78 (d, *J* = 8.5 Hz, 2H), 7.28 (dd, *J* = 8.2, 7.1 Hz, 3H), 7.26-7.25 (m, 2H), 7.24 (d, *J* = 1.4 Hz, 3H), 7.20-7.16 (m, 3H), 6.95 (dd, *J* = 7.8, 1.7 Hz, 2H), 3.63 (s, 3H), 3.61 (s, 3H), 3.24 (dt, *J* = 14.2, 1.8 Hz, 1H), 3.04 (s, 3H), 2.44 (dd, *J* = 14.3, 2.5 Hz, 1H), 2.33-2.28 (m, 1H), 2.21 (dt, *J* = 14.1, 2.1 Hz, 1H), 2.06 (dd, *J* = 14.2, 1.3 Hz, 1H), 1.98-1.90 (m, 2H), 1.47 (d, *J* = 13.7 Hz, 1H), 0.71 (s, 3H);

^{19}F NMR (565 MHz, CDCl3) δ -86.09 (d, *J* = 32.6 Hz), -88.80 (d, *J* = 32.6 Hz);

^{13}C NMR (151 MHz, $CDCl_3$) δ 172.4, 171.7, 154.6 (dd, *J* = 292.7, 290.5 Hz), 142.4, 141.9, 141.2 (dd, *J* = 4.9, 2.7 Hz), 140.1, 138.8, 129.5, 129.2 (t, *J* = 2.7 Hz), 129.0, 128.7, 128.1, 127.9, 127.5, 126.4, 126.3, 89.1 (dd, *J* = 22.5, 13.5 Hz), 54.7, 52.9, 52.3, 44.5, 42.3, 41.9, 41.9, 37.0 (t, *J* = 2.6 Hz), 34.7, 22.9;

HRMS: (ESI) calcd for $C_{34}H_{35}F_2O_6S^+[M+H]^+$ 609.2048; found 609.2057.

Dimethyl-3-(2-(4-acetylphenyl)-3,3-difluoroallyl)-5-(diphenylmethylene)-3-methylcyclohexane-1,1-dicarboxylate (63)

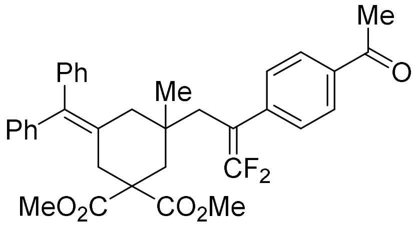

Chemical Formula: $C_{35}H_{34}F_2O_5$
Exact Mass: 572.2374

63 was prepared according to general procedure **2.2** using dimethyl 2-(2-methylallyl)-2-(3-phenylprop-2-yn-1-yl)malonate (0.2 mmol, 60.0 mg), bromobenzene (0.2 mmol, 31.4 mg) and 1-(4-(3,3,3-trifluoroprop-1-en-2-yl)phenyl)ethan-1-one (0.1 mmol, 21.4 mg), and was purified by silica gel column chromatography (PE/EtOAc = 5/1) to obtain **63** as colorless oil (45.2 mg, 79% yield).

^{1}H NMR (600 MHz, $CDCl_3$) δ 7.81 (d, *J* = 8.4 Hz, 2H), 7.28 (t, *J* = 7.7 Hz, 2H), 7.22 (dd, *J* = 5.0, 1.9 Hz, 3H), 7.20-7.13 (m, 5H), 6.94 (dd, *J* = 6.6, 3.0 Hz, 2H), 3.62 (s, 3H), 3.61 (s, 3H), 3.24 (m, 1H), 2.60 (s, 3H), 2.43 (dd, *J* = 14.3, 2.6 Hz, 1H), 2.33-2.28 (m, 1H), 2.22 (m, 1H), 2.06 (dd, *J* = 14.2, 1.3 Hz, 1H), 2.00 (m, 1H), 1.94 (d, *J* = 14.1 Hz, 1H), 1.51 (d, *J* = 13.8 Hz, 1H), 0.71 (s, 3H);

^{19}F NMR (565 MHz, CDCl$_3$) δ -87.02 (d, J = 34.4 Hz), -89.37 (d, J = 34.0 Hz);

^{13}C NMR (151 MHz, CDCl$_3$) δ 197.5, 172.5, 171.8, 154.4 (dd, J = 291.9, 289.9 Hz), 142.5, 142.1, 140.2 (dd, J = 4.6, 2.6 Hz), 140.0, 135.5, 129.7, 129.1, 128.8, 128.5 (t, J = 2.7 Hz), 128.4, 128.0, 127.9, 126.4, 126.2, 89.5 (dd, J = 21.8, 13.7 Hz), 54.7, 52.9, 52.3, 42.3, 41.9, 41.9, 37.0 (t, J = 2.3 Hz), 34.8, 26.6, 22.9;

HRMS: (ESI) calcd for $C_{35}H_{35}F_2O_5^+[M+H]^+$ 573.2358; found 573.2387.

Dimethyl-3-(3,3-difluoro-2-(4-(trifluoromethoxy)phenyl)allyl)-5-(diphenylmethylene)-3-methylcyclohexane-1,1-dicarboxylate (64)

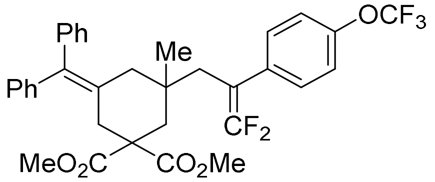

Chemical Formula: $C_{34}H_{31}F_5O_5$
Exact Mass: 614.2092

64 was prepared according to general procedure **2.2** using dimethyl 2-(2-methylallyl)-2-(3-phenylprop-2-yn-1-yl)malonate (0.2 mmol, 60.0 mg), bromobenzene (0.2 mmol, 31.4 mg) and 1-(trifluoromethoxy)-4-(3,3,3-trifluoroprop-1-en-2-yl)benzene (0.1 mmol, 25.6 mg), and was purified by silica gel column chromatography (PE/EtOAc = 5/1) to obtain **64** as colorless oil (47.3 mg, 77% yield).

^{1}H NMR (600 MHz, CDCl$_3$) δ 7.28 (dd, J = 8.1, 7.1 Hz, 2H), 7.26-7.21 (m, 3H), 7.19 (m, 3H), 7.06 (s, 4H), 6.96 (dd, J = 8.1, 1.6 Hz, 2H), 3.63 (s, 3H), 3.61 (s, 3H), 3.25 (dd, J = 14.2, 1.8 Hz, 1H), 2.39 (dd, J = 14.2, 2.6 Hz, 1H), 2.28-2.23 (m, 1H), 2.21 (m, 1H), 2.08 (dd, J = 14.3, 1.3 Hz, 1H), 2.00 (m, 1H), 1.91 (d, J = 14.1 Hz, 1H), 1.50 (d, J = 13.8 Hz, 1H), 0.72 (s, 3H);

^{19}F NMR (565 MHz, CDCl$_3$) δ -57.78, -88.26 (d, J = 37.6 Hz), -90.58 (dd, J = 37.5, 3.0 Hz);

^{13}C NMR (151 MHz, CDCl$_3$) δ 172.6, 171.8, 154.5 (dd, J = 290.8, 289.2 Hz),148.0, 142.6, 142.2, 140.0, 133.8 (dd, J = 5.0, 2.5 Hz), 129.83, 129.76 (t, J = 2.7 Hz), 129.1, 128.8, 128.1, 128.0, 126.4, 126.2, 120.8, 120.4 (q, J = 257.3 Hz), 89.0 (dd, J = 22.0, 14.2 Hz), 54.8, 52.9, 52.3, 42.7, 42.0, 41.9, 37.0 (t, J = 2.7 Hz), 34.8, 22.9;

HRMS: (ESI) calcd for $C_{34}H_{32}F_5O_5^+$[M+H]$^+$ 615.2103; found 615.2122.

Dimethyl3-(2-([1,1'-biphenyl]-4-yl)-3,3-difluoroallyl)-5-(diphenylmethylene)-3-methylcyclohexane-1,1-dicarboxylate (65)

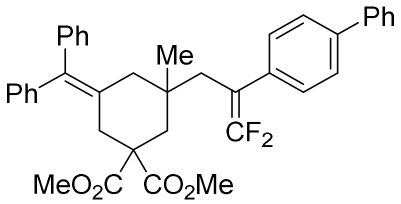

Chemical Formula: $C_{39}H_{36}F_2O_4$
Exact Mass: 606.2582

65 was prepared according to general procedure **2.2** using dimethyl 2-(2-methylallyl)-2-(3-phenylprop-2-yn-1-yl)malonate (0.2 mmol, 60.0 mg), bromobenzene (0.2 mmol, 31.4 mg) and 4-(3,3,3-trifluoroprop-1-en-2-yl)-1,1'-biphenyl (0.1 mmol, 24.8 mg), and was purified by silica gel column chromatography (PE/EtOAc = 10/1) to obtain **65** as colorless oil (41.2 mg, 68% yield).

^{1}H NMR (600 MHz, $CDCl_3$) δ 7.63-7.58 (m, 2H), 7.46 (m, 4H), 7.40-7.35 (m, 1H), 7.31-7.27 (m, 2H), 7.25-7.18 (m, 6H), 7.17-7.13 (m, 2H), 7.01-6.97 (m, 2H), 3.64 (s, 3H), 3.61 (s, 3H), 3.26 (m, 1H), 2.44 (dd, J = 14.2, 2.7 Hz, 1H), 2.33 (m, 1H), 2.27 (dd, J = 14.1, 2.2 Hz, 1H), 2.13-2.07 (m, 2H), 1.98 (d, J = 14.1 Hz, 1H), 1.58 (s, 1H), 0.76 (s, 3H);

^{19}F NMR (565 MHz, $CDCl_3$) δ -88.68 (d, J = 38.6 Hz), -90.94 (d, J = 38.5 Hz);

^{13}C NMR (151 MHz, $CDCl_3$) δ 172.6, 171.9, 154.4 (dd, J = 290.5, 288.6 Hz), 142.5, 142.2, 140.5, 139.8, 139.7, 134.0 (dd, J = 4.7, 2.4 Hz), 130.0, 129.1, 128.85, 128.76, 128.7 (t, J = 2.7 Hz), 128.0, 127.9, 127.3, 127.0, 126.9, 126.3, 126.1, 89.5 (dd, J = 21.2, 14.2 Hz), 54.8, 52.8, 52.2, 42.5, 41.9, 41.8, 37.0 (t, J = 2.3 Hz), 34.9, 23.0;

HRMS: (ESI) calcd for $C_{39}H_{37}F_2O_4^+$[M+H]$^+$ 607.2578; found 607.2587.

Dimethyl-3-(3,3-difluoro-2-(naphthalen-2-yl)allyl)-5-(diphenylmethylene)-3-methylcyclohexane-1,1-dicarboxylate (66)

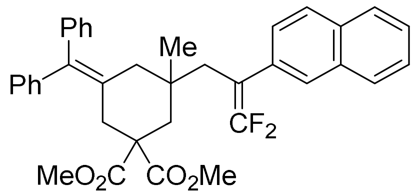

Chemical Formula: $C_{37}H_{34}F_2O_4$
Exact Mass: 580.2425

66 was prepared according to general procedure **2.2** using dimethyl 2-(2-methylallyl)-2-(3-phenylprop-2-yn-1-yl)malonate (0.2 mmol, 60.0 mg), bromobenzene (0.2 mmol, 31.4 mg) and 2-(3,3,3-trifluoroprop-1-en-2-yl)naphthalene (0.1 mmol, 22.2 mg), and was purified by silica gel column chromatography (PE/EtOAc = 5/1) to obtain **66** as colorless oil (42.3 mg, 73% yield).

^{1}H NMR (600 MHz, $CDCl_3$) δ 7.85-7.78 (m, 1H), 7.77-7.69 (m, 2H), 7.54 (s, 1H), 7.49 (m, 2H), 7.28 (d, *J* = 7.8 Hz, 2H), 7.22-7.12 (m, 7H), 6.91 (dd, *J* = 7.8, 1.7 Hz, 2H), 3.61 (s, 3H), 3.58 (s, 3H), 3.23 (dd, *J* = 14.3, 2.3 Hz, 1H), 2.51 (dd, *J* = 14.2, 2.7 Hz, 1H), 2.41 (m, 1H), 2.31-2.25 (m, 1H), 2.09 (dd, *J* = 17.8, 14.0 Hz, 2H), 1.99 (d, *J* = 14.1 Hz, 1H), 1.58 (s, 1H), 0.72 (s, 3H);

^{19}F NMR (565 MHz, $CDCl_3$) δ -88.44 (d, *J* = 37.8 Hz), -90.96 (d, *J* = 37.9 Hz);

^{13}C NMR (151 MHz, $CDCl_3$) δ 172.6, 171.8, 154.5 (dd, *J* = 290.5, 288.6 Hz), 142.4, 142.2, 139.8, 133.1, 132.5 (dd, *J* = 4.2, 2.4 Hz), 132.3, 129.9, 129.1, 128.8, 127.98, 127.96, 127.90, 127.87, 127.5, 127.3 (t, *J* = 2.8 Hz), 126.3, 126.2 (t, *J* = 6.4 Hz), 126.15, 126.07, 126.0, 89.8 (dd, *J* = 21.3, 14.0 Hz), 54.8, 52.8, 52.2, 42.6, 41.9, 41.7, 37.0 (t, *J* = 2.7 Hz), 34.9, 23.2;

HRMS: (ESI) calcd for $C_{37}H_{34}F_2O_4{}^+$[M+H]$^+$ 581.2458; found 581.2487.

dimethyl3-(2-(4-chlorophenyl)-3,3-difluoroallyl)-5-(diphenylmethylene)-3-methylcyclohexane-1,1-dicarboxylate (67)

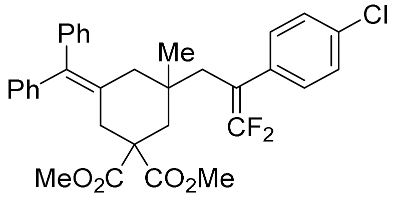

Chemical Formula: $C_{33}H_{31}ClF_2O_4$
Exact Mass: 564.1879

67 was prepared according to general procedure **2.2** using dimethyl 2-(2-methylallyl)-2-(3-phenylprop-2-yn-1-yl)malonate (0.2 mmol, 60.0 mg), bromobenzene (0.2 mmol, 31.4 mg) and 1-chloro-4-(3,3,3-trifluoroprop-1-en-2-yl)benzene (0.1 mmol, 20.6 mg), and was purified by silica gel column chromatography (PE/EtOAc = 5/1) to obtain **67** as colorless oil (36.7 mg, 65% yield).

^{1}H NMR (600 MHz, $CDCl_3$) δ 7.38-7.29 (m, 6H), 7.27 (s, 2H), 7.25-7.23 (m, 2H), 7.04 (m, 4H), 3.70 (d, *J* = 1.2 Hz, 3H), 3.68 (d, *J* = 1.3 Hz, 3H), 3.31 (m, 1H), 2.44 (dd, *J* = 14.1, 2.6 Hz, 1H), 2.29 (dd, *J* = 17.2, 14.2 Hz, 2H), 2.14 (d, *J* = 14.2 Hz, 1H), 2.09-2.05 (m, 1H), 1.99 (d, *J* = 14.0 Hz, 1H), 1.58 (d, *J* = 13.8 Hz, 1H), 0.77 (s, 3H);

^{19}F NMR (565 MHz, $CDCl_3$) δ -88.42 (d, *J* = 37.6 Hz), -90.64 (d, *J* = 37.4 Hz);

^{13}C NMR (151 MHz, $CDCl_3$) δ 172.6, 171.8, 154.4 (dd, *J* = 290.5, 288.6 Hz), 142.5, 142.1, 140.0, 133.5 (dd, *J* = 4.2, 2.3 Hz), 132.8, 129.8, 129.6 (t, *J* = 2.7 Hz), 129.1, 128.8, 128.6, 128.0, 127.9, 126.3, 126.2, 89.0 (dd, *J* = 21.8, 14.2 Hz), 54.7, 52.9, 52.3, 42.5, 41.9, 41.9, 34.8, 36.9 (d, *J* = 2.7 Hz), 22.9;

HRMS: (ESI) calcd for $C_{33}H_{32}ClF_2O_4^+$[M+H]$^+$ 565.1887; found 565.1882.

Dimethyl-3-(2-(benzo[*d*][1,3]dioxol-5-yl)-3,3-difluoroallyl)-5-(diphenylmethylene)-3-methylcyclohexane-1,1-dicarboxylate (68)

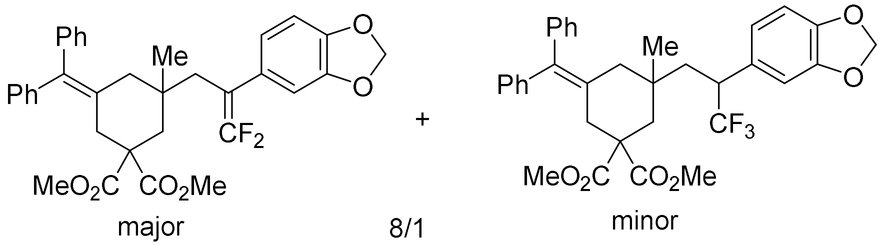

Chemical Formula: $C_{34}H_{32}F_2O_6$
Exact Mass: 574.2167

68 was prepared according to general procedure **2.2** using dimethyl 2-(2-methylallyl)-2-(3-phenylprop-2-yn-1-yl)malonate (0.2 mmol, 60.0 mg), bromobenzene (0.2 mmol, 31.4 mg) and 5-(3,3,3-trifluoroprop-1-en-2-yl)benzo[*d*][1,3]dioxole (0.1 mmol, 21.6 mg), and was purified by silica gel column chromatography (PE/EtOAc = 5/1) to obtain **68** as colorless oil (36.2 mg, 63% yield).

^{1}H NMR (600 MHz, $CDCl_3$) δ 7.29 (t, *J* = 7.6 Hz, 2H), 7.24 (d, *J* = 7.7 Hz, 2H), 7.22-

7.16 (m, 4H), 6.99 (m, 2H), 6.66 (dd, *J* = 8.0, 2.8 Hz, 1H), 6.56-6.48 (m, 2H), 5.97-5.93 (m, 2H), 3.63 (d, *J* = 2.7 Hz, 3H), 3.61 (d, *J* = 2.9 Hz, 3H), 3.25 (m, 1H), 2.33 (dd, *J* = 14.1, 2.7 Hz, 1H), 2.21 (m, 2H), 2.09 (m, 2H), 1.94 (dd, *J* = 14.1, 2.6 Hz, 1H), 1.57 (d, *J* = 13.9 Hz, 1H), 0.71 (d, *J* = 2.9 Hz, 3H);

^{19}F NMR (565 MHz, $CDCl_3$) δ -89.51 (d, *J* = 40.1 Hz), -91.35 (d, *J* = 39.6 Hz);

^{13}C NMR (151 MHz, $CDCl_3$) δ 207.4, 172.6, 171.9, 154.3 (dd, *J* = 290.6, 288.6 Hz), 147.4, 146.4, 142.4, 142.2, 139.8, 130.0, 129.1, 128.8, 128.6 (dd, *J* = 4.4, 2.1 Hz), 127.9, 127.9, 126.3, 126.2, 121.9 (t, *J* = 2.7 Hz),108.8 (t, *J* = 2.7 Hz), 108.2, 101.0, 89.5 (dd, *J* = 21.5, 14.4 Hz), 54.8, 52.8, 52.2, 42.8, 41.9, 41.7, 36.9 (t, *J* = 3.0 Hz), 34.9, 22.9;

HRMS: (ESI) calcd for $C_{34}H_{33}F_2O_6^+$[M+H]$^+$ 575.2154; found 575.2175.

Dimethyl-3-(2-(4-(benzyloxy)phenyl)-3,3-difluoroallyl)-5-(diphenylmethylene)-3-methylcyclohexane-1,1-dicarboxylate (69)

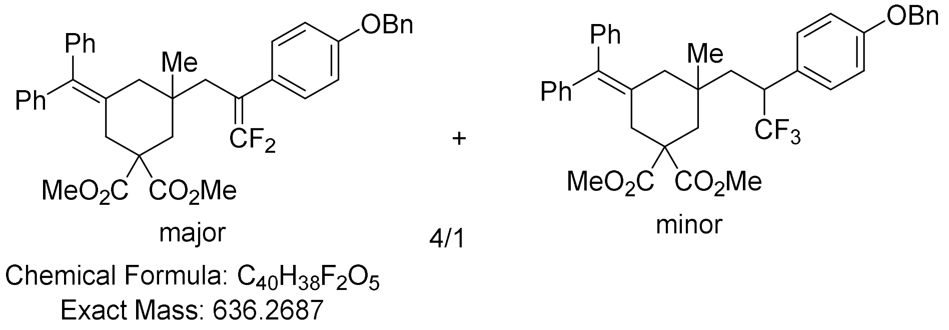

69 was prepared according to general procedure **2.2** using dimethyl 2-(2-methylallyl)-2-(3-phenylprop-2-yn-1-yl)malonate (0.2 mmol, 60.0 mg), bromobenzene (0.2 mmol, 31.4 mg) and 1-(benzyloxy)-4-(3,3,3-trifluoroprop-1-en-2-yl)benzene (0.1 mmol, 27.8 mg), and was purified by silica gel column chromatography (PE/EtOAc = 5/1) to obtain **69** as colorless oil (38.8 mg, 61% yield).

^{1}H NMR (600 MHz, $CDCl_3$) δ 7.45 (d, *J* = 7.3 Hz, 2H), 7.42 (d, *J* = 7.5 Hz, 2H), 7.36-7.33 (m, 1H), 7.29 (t, *J* = 7.6 Hz, 2H), 7.23-7.17 (m, 6H), 7.00-6.96 (m, 4H), 6.84 (d, *J* = 8.6 Hz, 2H), 5.04 (s, 2H), 3.63 (d, *J* = 0.9 Hz, 3H), 3.61 (d, *J* = 0.8 Hz, 3H), 3.25 (dd, *J* = 14.2, 2.0 Hz, 1H), 2.37 (dd, *J* = 14.1, 2.5 Hz, 1H), 2.23 (m, 2H), 2.08 (d, *J* = 14.1 Hz, 2H), 1.94 (d, *J* = 14.0 Hz, 1H), 1.55 (d, *J* = 13.8 Hz, 1H), 0.71 (s, 3H);

^{19}F NMR (565 MHz, $CDCl_3$) δ -89.89 (d, *J* = 41.2 Hz), -92.05 (d, *J* = 41.3 Hz);

^{13}C NMR (151 MHz, $CDCl_3$) δ 172.6, 171.9, 157.6, 154.5 (dd, *J* = 290.6, 288.6 Hz), 142.5, 139.8, 136.8, 130.2 (dd, *J* = 5.3, 2.1 Hz)130.0, 129.4 (t, *J* = 2.7 Hz), 129.2, 128.9, 128.6, 128.03, 127.98, 127.9, 127.5, 126.3, 126.1, 114.6, 89.2 (dd, *J* = 21.2, 14.7 Hz), 69.9, 60.4, 54.8, 52.8, 52.2, 42.7, 41.9, 41.8, 34.9, 36.9 (t, *J* = 2.4 Hz), 23.0, 21.1, 14.2; HRMS: (ESI) calcd for $C_{40}H_{39}F_2O_5^+$[M+H]$^+$ 637.2712; found 637.2701.

Dimethyl-3-(2-(dibenzo[*b*,*d*]thiophen-2-yl)-3,3-difluoroallyl)-5-(diphenylmethylene)-3-methylcyclohexane-1,1-dicarboxylate (70)

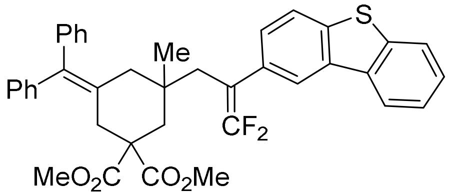

Chemical Formula: $C_{39}H_{34}F_2O_4S$
Exact Mass: 636.2146

70 was prepared according to general procedure **2.2** using dimethyl 2-(2-methylallyl)-2-(3-phenylprop-2-yn-1-yl)malonate (0.2 mmol, 60.0 mg), bromobenzene (0.2 mmol, 31.4 mg) and 2-(3,3,3-trifluoroprop-1-en-2-yl)dibenzo[b,d]thiophene (0.1 mmol, 27.8 mg), and was purified by silica gel column chromatography (PE/EtOAc = 5/1) to obtain **70** as colorless oil (45.2 mg, 71% yield).

^{1}H NMR (600 MHz, $CDCl_3$) δ 8.12-8.07 (m, 1H), 7.88-7.83 (m, 2H), 7.69 (d, *J* = 8.3 Hz, 1H), 7.50-7.46 (m, 2H), 7.28-7.25 (m, 1H), 7.24 (s, 1H), 7.18-7.13 (m, 4H), 7.07 (t, *J* = 7.6 Hz, 2H), 6.98-6.94 (m, 1H), 6.90-6.86 (m, 2H), 3.61 (s, 3H), 3.58 (s, 3H), 3.22 (m, 1H), 2.52 (dd, *J* = 14.2, 2.6 Hz, 1H), 2.44-2.38 (m, 1H), 2.28 (m, 1H), 2.06 (m, 2H), 2.01 (d, *J* = 14.2 Hz, 1H), 1.57 (d, *J* = 13.9 Hz, 1H), 0.74 (s, 3H);

^{19}F NMR (565 MHz, $CDCl_3$) δ -88.73 (d, *J* = 38.7 Hz), -91.21 (d, *J* = 38.8 Hz);

^{13}C NMR (151 MHz, $CDCl_3$) δ 172.6, 171.8, 155.4 (dd, *J* = 289.8, 288.8 Hz), 142.3, 142.2, 139.9, 139.7, 138.1, 135.6, 135.2, 131.4 (dd, *J* = 4.7, 2.4 Hz),129.8, 129.1, 128.7, 127.9, 127.8, 126.92 (t, *J* = 2.4 Hz), 126.89, 126.3, 126.1, 124.3, 122.8, 122.7, 121.8, 121.2 (t, *J* = 2.7 Hz), 89.7 (dd, *J* = 21.4, 14.1 Hz), 54.8, 52.8, 52.2, 42.8, 42.1, 41.9, 36.9 (t, *J* = 2.4 Hz), 34.9, 23.0;

HRMS: (ESI) calcd for $C_{39}H_{35}F_2NO_4S^+$[M+H]$^+$ 637.2184; found 637.2177.

Dimethyl-3-(3,3-difluoro-2-(9-phenyl-9*H*-carbazol-3-yl)allyl)-5-(diphenylmethylene)-3-methylcyclohexane-1,1-dicarboxylate (71)

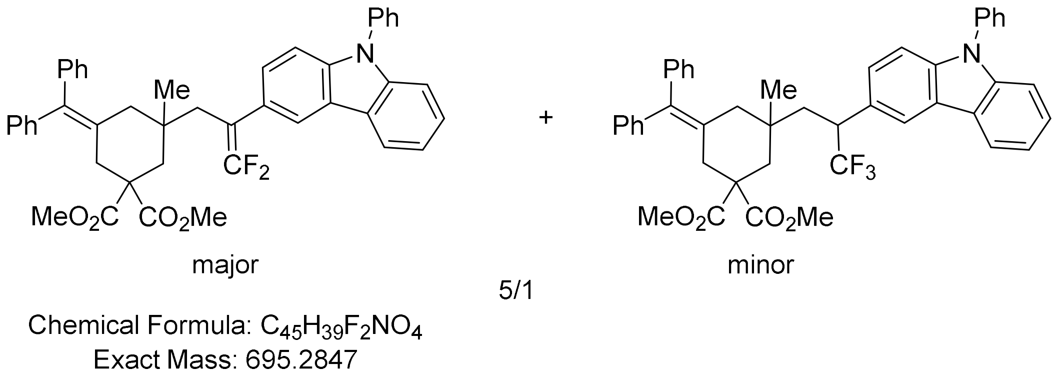

71 was prepared according to general procedure **2.2** using dimethyl 2-(2-methylallyl)-2-(3-phenylprop-2-yn-1-yl)malonate (0.2 mmol, 60.0 mg), bromobenzene (0.2 mmol, 31.4 mg) and 9-phenyl-3-(3,3,3-trifluoroprop-1-en-2-yl)-9H-carbazole (0.1 mmol, 33.7 mg), and was purified by silica gel column chromatography (PE/EtOAc = 5/1) to obtain **71** as colorless oil (53.5 mg, 77% yield).

^{1}H NMR (600 MHz, $CDCl_3$) δ 8.12 (m, 1H), 7.85 (s, 1H), 7.63 (m, 2H), 7.58-7.55 (m, 2H), 7.51-7.48 (m, 1H), 7.44-7.42 (m, 2H), 7.34-7.32 (m, 1H), 7.27 (d, *J* = 7.5 Hz, 2H), 7.23 (d, *J* = 8.5 Hz, 1H), 7.17 (m, 3H), 7.11-7.07 (m, 3H), 7.01-6.97 (m, 1H), 6.93-6.90 (m, 2H), 3.62 (s, 3H), 3.60 (s, 3H), 3.23 (m, 1H), 2.53 (dd, *J* = 14.1, 2.6 Hz, 1H), 2.45-2.40 (m, 1H), 2.31 (dt, *J* = 14.3, 2.1 Hz, 1H), 2.10 (td, *J* = 15.0, 14.2, 1.7 Hz, 2H), 2.03 (d, *J* = 14.1 Hz, 1H), 1.59 (d, *J* = 14.0 Hz, 1H), 0.76 (s, 3H);

^{19}F NMR (565 MHz, $CDCl_3$) δ -90.08 (d, *J* = 41.7 Hz), -92.45 (d, *J* = 41.6 Hz);

^{13}C NMR (151 MHz, $CDCl_3$) δ 172.7, 171.9, 154.3 (dd, *J* = 291.2, 289.0 Hz),142.3, 141.1, 139.7, 137.5, 130.0, 129.9, 129.2, 128.8, 127.9, 127.8, 127.5, 126.9, 126.6 (dd, *J* = 4.3, 2.1 Hz),126.4, 126.2, 126.1, 126.1, 123.3, 123.1, 120.4, 120.03 (t, *J* = 3.2 Hz), 119.97, 109.8, 109.6, 90.1 (dd, *J* = 20.9, 14.5 Hz), 54.8, 52.8, 52.2, 43.2, 42.1, 41.8, 36.9 (t, *J* = 2.7 Hz), 35.0, 23.1;

HRMS: (ESI) calcd for $C_{45}H_{40}F_2NO_4^+$[M+H]$^+$ 696.2844; found 696.2895.

Dimethyl-3-(2-(dibenzo[b,d]furan-2-yl)-3,3-difluoroallyl)-5-(diphenylmethylene)-

3-methylcyclohexane-1,1-dicarboxylate (72)

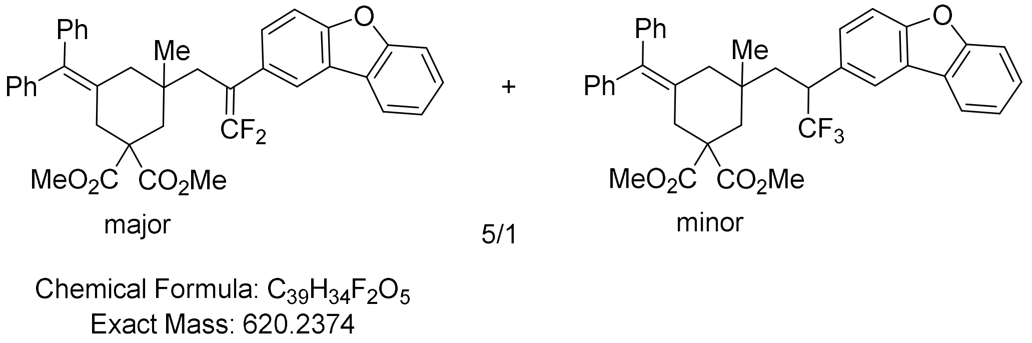

72 was prepared according to general procedure **2.2** using dimethyl 2-(2-methylallyl)-2-(3-phenylprop-2-yn-1-yl)malonate (0.2 mmol, 60.0 mg), bromobenzene (0.2 mmol, 31.4 mg) and 2-(3,3,3-trifluoroprop-1-en-2-yl)dibenzo[*b*,*d*]furan (0.1 mmol, 26.2 mg), and was purified by silica gel column chromatography (PE/EtOAc = 5/1) to obtain **72** as colorless oil (42.8 mg, 69% yield).

^{1}H NMR (600 MHz, $CDCl_3$) δ 7.93 (dd, *J* = 7.6, 1.2 Hz, 1H), 7.65 (t, *J* = 1.6 Hz, 1H), 7.58 (d, *J* = 8.2 Hz, 1H), 7.49 (m, 1H), 7.42-7.37 (m, 2H), 7.25 (s, 1H), 7.20-7.14 (m, 4H), 7.13-7.08 (m, 3H), 7.06-7.02 (m, 1H), 6.92-6.88 (m, 2H), 3.62 (s, 3H), 3.59 (s, 3H), 3.22 (m, 1H), 2.50 (dd, *J* = 14.3, 2.6 Hz, 1H), 2.41-2.37 (m, 1H), 2.27 (m, 1H), 2.09-2.04 (m, 2H), 2.00 (d, *J* = 14.2 Hz, 1H), 1.56 (d, *J* = 13.9 Hz, 1H), 0.74 (s, 3H);

^{13}C NMR (151 MHz, $CDCl_3$) δ 172.6, 171.9, 156.5, 155.0, 153.5 (dd, *J* = 291.2, 289.0 Hz), 142.3, 142.2, 139.9, 129.9, 129.7, 129.6 (dd, *J* = 4.1, 1.8 Hz), 129.1, 128.8, 127.9, 127.9, 127.5, 127.3, 126.3, 126.1, 124.3, 123.9, 122.7, 120.8, 120.5 (t, *J* = 2.3 Hz), 111.7, 111.5, 89.7 (dd, *J* = 21.3, 14.4 Hz), 54.8, 52.8, 52.2, 43.1, 42.1, 41.9, 36.9 (t, *J* = 2.7 Hz), 34.9, 23.0;

^{19}F NMR (565 MHz, $CDCl_3$) δ -89.35 (d, *J* = 40.0 Hz), -91.73 (d, *J* = 39.7 Hz);

HRMS: (ESI) calcd for $C_{39}H_{35}F_2O_5^+$ $[M+H]^+$ 621.2401; found 621.2389.

dimethyl 3-(3,3-difluoro-2-(quinolin-3-yl)allyl)-5-(diphenylmethylene)-3-methylcyclohexane-1,1-dicarboxylate (73)

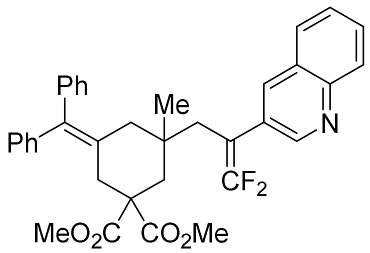

Chemical Formula: $C_{36}H_{33}F_2NO_4$
Exact Mass: 581.2378

73 was prepared according to general procedure **2.2** using dimethyl 2-(2-methylallyl)-2-(3-phenylprop-2-yn-1-yl)malonate (0.2 mmol, 60.1 mg), bromobenzene (0.2 mmol, 31.4 mg) and 3-(3,3,3-trifluoroprop-1-en-2-yl)quinoline (0.1 mmol, 22.3 mg), and was purified by silica gel column chromatography (PE/EtOAc = 10/1) to obtain **73** as colorless oil (34.3 mg, 59% yield).

^{1}H NMR (600 MHz, $CDCl_3$) δ 8.74-8.68 (m, 1H), 8.12-8.06 (m, 1H), 7.82-7.77 (m, 1H), 7.76-7.70 (m, 2H), 7.60-7.54 (m, 1H), 7.29-7.25 (m, 2H), 7.20-7.08 (m, 6H), 6.93-6.85 (m, 2H), 3.62 (s, 3H), 3.59 (s, 3H), 3.23 (d, *J* = 14.2 Hz, 1H), 2.53 (dd, *J* = 14.3, 2.6 Hz, 1H), 2.47-2.41 (m, 1H), 2.31-2.25 (m, 1H), 2.14-2.05 (m, 2H), 2.00 (d, *J* = 14.1 Hz, 1H), 1.63 (d, *J* = 13.8 Hz, 1H), 0.72 (s, 3H);

^{19}F NMR (565 MHz, $CDCl_3$) δ -86.11 (d, *J* = 34.1 Hz), -89.66 (d, *J* = 34.2 Hz);

^{13}C NMR (151 MHz, $CDCl_3$) δ 172.5, 171.7, 157.0-152.4 (m), 150.1 (t, *J* = 2.5 Hz), 146.9, 142.3, 142.1, 140.1, 134.9 (t, *J* = 2.4 Hz), 129.6, 129.6, 129.1, 129.1, 128.7, 128.3-128.2 (m), 127.9, 127.9, 127.5, 126.9, 126.4, 126.3, 87.4 (dd, *J* = 22.8, 14.0 Hz), 54.7, 52.8, 52.3, 42.2, 42.0, 41.9, 37.0 (t, *J* = 2.6 Hz), 34.8, 23.4;

HRMS: (ESI) calcd for $C_{36}H_{34}F_2NO_4^+$[M+H]$^+$ 582.2450; found 582.2436.

4-(3-((2*R*,3*S*)-5-(diphenylmethylene)-3-methyl-2-phenyl-1-tosylpiperidin-3-yl)-1,1-difluoroprop-1-en-2-yl)benzonitrile (75)

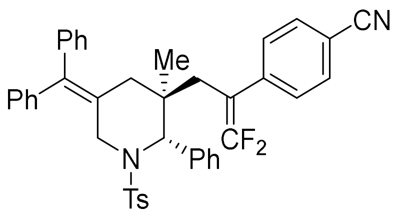

Chemical Formula: $C_{42}H_{36}F_2N_2O_2S$
Exact Mass: 670.2466

75 was prepared according to general procedure **2.3** using (*R*)-4-methyl-*N*-(2-methyl-1-phenylallyl)-*N*-(3-phenylprop-2-yn-1-yl)benzenesulfonamide (0.2 mmol, 83.0 mg), bromobenzene (0.2 mmol, 31.4 mg) and 4-(3,3,3-trifluoroprop-1-en-2-yl)benzonitrile (0.1 mmol, 19.7 mg), and was purified by silica gel column chromatography (PE/EtOAc = 5/1) to obtain **75** as white solid (46.2 mg, 69% yield).

^{1}H NMR (600 MHz, $CDCl_3$) δ 7.55 (d, *J* = 8.4 Hz, 2H), 7.38 (t, *J* = 7.6 Hz, 2H), 7.30 (m, 4H), 7.22-7.19 (m, 2H), 7.18-7.16 (m, 1H), 7.15-7.12 (m, 2H), 7.10 (d, *J* = 7.4 Hz, 2H), 7.09-7.06 (m, 2H), 7.05-7.01 (m, 4H), 6.90 (d, *J* = 8.0 Hz, 2H), 4.73-4.66 (m, 2H), 3.79 (d, *J* = 15.4 Hz, 1H), 2.89 (dt, *J* = 14.8, 2.7 Hz, 1H), 2.69 (dd, *J* = 14.7, 2.3 Hz, 1H), 2.33-2.24 (m, 4H), 2.14 (d, *J* = 15.1 Hz, 1H), 0.27 (s, 3H);

^{19}F NMR (565 MHz, $CDCl_3$) δ -84.48 (d, *J* = 29.6 Hz), -87.00 (d, *J* = 29.9 Hz);

^{13}C NMR (151 MHz, $CDCl_3$) δ 154.4, (dd, *J* = 292.2 Hz, 292.1 Hz), 142.4, 141.0, 141.0, 140.4, 139.6 (dd, *J* = 4.4, 2.3 Hz)., 138.1, 135.6, 132.2, 129.6, 129.4, 129.3 (t, *J* = 2.3 Hz), 128.9, 128.8, 128.5, 128.2, 128.2, 127.6, 127.3, 127.2, 127.0, 118.6, 111.1, 89.4 (dd, *J* = 21.6, 14.1 Hz), 64.5, 45.2, 40.0 (t, *J* = 2.2 Hz)., 36.8, 36.4, 25.7, 21.4;

HRMS: (ESI) calcd for $C_{42}H_{37}F_2N_2O_2S^+$[M+H]$^+$ 671.2471; found 671.2479.

methyl 4-(3-((2*R*,3*S*)-5-(diphenylmethylene)-3-methyl-2-phenyl-1-tosylpiperidin-3-yl)-1,1-difluoroprop-1-en-2-yl)benzoate (76)

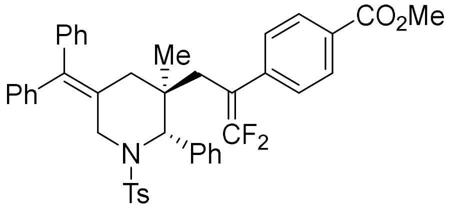

Chemical Formula: $C_{43}H_{39}F_2NO_4S$
Exact Mass: 703.2568

76 was prepared according to general procedure **2.3** using (*R*)-4-methyl-*N*-(2-methyl-1-phenylallyl)-*N*-(3-phenylprop-2-yn-1-yl)benzenesulfonamide (0.2 mmol, 83.0 mg), bromobenzene (0.2 mmol, 31.4 mg) and methyl 4-(3,3,3-trifluoroprop-1-en-2-yl)benzoate (0.1 mmol, 23.0 mg), and was purified by silica gel column chromatography (PE/EtOAc = 5/1) to obtain **76** as white solid (47.1 mg, 67% yield).

^{1}H NMR (600 MHz, $CDCl_3$) δ 7.93-7.90 (m, 2H), 7.37 (t, *J* = 7.6 Hz,2H), 7.31-7.27 (m, 3H), 7.22-7.17 (m, 2H), 7.16 (m, 1H), 7.13-7.10 (m, 5H), 7.09 (d, *J* = 1.6 Hz, 1H), 7.07-7.05 (m, 2H), 7.02-6.98 (m, 2H), 6.93 (d, *J* = 8.0 Hz, 2H), 4.76 (s, 1H), 4.69 (d, *J* = 15.5 Hz, 1H), 3.91 (s, 3H), 3.83 (d, *J* = 15.5 Hz, 1H), 2.85 (m, 1H), 2.75-2.67 (m, 1H), 2.30 (s, 3H), 2.23 (d, *J* = 15.2 Hz, 1H), 2.11 (d, *J* = 15.2 Hz, 1H), 0.24 (s, 3H);

^{19}F NMR (565 MHz, $CDCl_3$) δ -85.54 (d, *J* = 32.0 Hz), -87.70 (d, *J* = 32.1 Hz);

^{13}C NMR (151 MHz, $CDCl_3$) δ 166.7, 154.3 (dd, *J* = 291.1 Hz, 291.1 Hz), 142.4, 141.1, 141.1, 140.2, 139.5 (dd, *J* = 4.3, 2.4 Hz)., 138.4, 135.9, 129.7, 129.6, 129.4, 128.9, 128.9, 128.8, 128.6 (t, *J* = 2.7 Hz), 128.5, 128.1, 128.1, 127.8, 127.4, 127.2, 127.2, 126.9, 89.7 (dd, *J* = 20.9, 14.5 Hz), 64.7, 52.1, 45.3, 40.0 (t, *J* = 2.4 Hz), 36.7, 36.7, 25.8, 21.4;

HRMS: (ESI) calcd for $C_{43}H_{40}F_2NO_4S^+[M+H]^+$ 704.2578; found 704.2575.

(2*R*,3*S*)-3-(2-(4-chlorophenyl)-3,3-difluoroallyl)-5-(diphenylmethylene)-3-methyl-2-phenyl-1-tosylpiperidine (77)

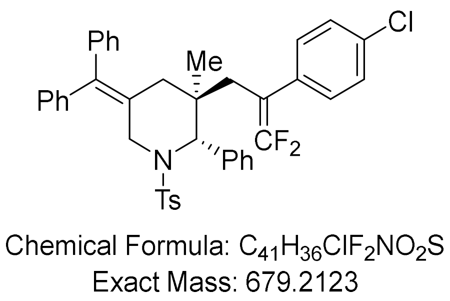

77 was prepared according to general procedure **2.3** using (*R*)-4-methyl-*N*-(2-methyl-1-phenylallyl)-*N*-(3-phenylprop-2-yn-1-yl)benzenesulfonamide (0.2 mmol, 83.0 mg), bromobenzene (0.2 mmol, 31.4 mg) and 1-chloro-4-(3,3,3-trifluoroprop-1-en-2-yl)benzene (0.1 mmol, 20.6 mg), and was purified by silica gel column chromatography

(PE/EtOAc = 5/1) to obtain **77** as colorless oil (38.7 mg, 57% yield).

^{1}H NMR (600 MHz, CDCl$_3$) δ 7.36 (t, *J* = 7.6 Hz, 2H), 7.30-7.26 (m, 4H), 7.21 (d, *J* = 8.4 Hz, 2H), 7.19-7.16 (m, 3H), 7.14-7.09 (m, 6H), 7.00-6.97 (m, 2H), 6.95-6.90 (m, 4H), 4.75 (s, 1H), 4.67 (d, *J* = 15.6 Hz, 1H), 3.84 (d, *J* = 15.6 Hz, 1H), 2.78 (m, 1H), 2.67 (d, *J* = 14.1 Hz, 1H), 2.31 (s, 3H), 2.22 (d, *J* = 15.3 Hz, 1H), 0.28 (s, 3H);

^{19}F NMR (565 MHz, CDCl$_3$) δ -86.60 (d, *J* = 34.5 Hz), -88.80 (d, *J* = 34.4 Hz);

^{13}C NMR (151 MHz, CDCl$_3$) δ 155.6 (dd, *J* = 290.2 Hz, *J* = 290.5 Hz), 142.4, 141.1, 141.1, 140.1, 138.5, 135.9, 133.1, 133.0 (dd, *J* = 4.5, 2.4 Hz), 129.9 (t, *J* = 2.7 Hz), 129.9, 129.6, 129.4, 128.9, 128.8, 128.7, 128.5, 128.1, 127.9, 127.4, 127.2, 127.2, 126.90, 89.1 (dd, *J* = 20.9, 15.0 Hz), 64.7, 45.3, 39.9 (t, *J* = 2.5 Hz), 37.0, 36.7, 25.7, 21.4;

HRMS: (ESI) calcd for $C_{41}H_{37}ClF_2NO_2S^+[M+H]^+$ 680.2128; found 680.2132.

(2*R*,3*S*)-3-(3,3-difluoro-2-(4-(methylsulfonyl)phenyl)allyl)-5-(diphenylmethylene)-3-methyl-2-phenyl-1-tosylpiperidine (78)

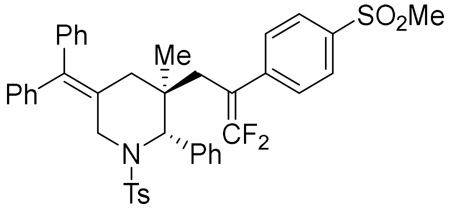

Chemical Formula: $C_{42}H_{39}F_2NO_4S_2$
Exact Mass: 723.2289

78 was prepared according to general procedure **2.3** using (*R*)-4-methyl-*N*-(2-methyl-1-phenylallyl)-*N*-(3-phenylprop-2-yn-1-yl)benzenesulfonamide (0.2 mmol, 83.0 mg), bromobenzene (0.2 mmol, 31.4 mg) and 1-(methylsulfonyl)-4-(3,3,3-trifluoroprop-1-en-2-yl)benzene (0.1 mmol, 25.0 mg), and was purified by silica gel column chromatography (PE/EtOAc = 5/1) to obtain **78** as colorless oil (45.5 mg, 63% yield).

^{1}H NMR (600 MHz, CDCl$_3$) δ 7.88 (d, *J* = 8.5 Hz, 2H), 7.39 (t, *J* = 7.6 Hz, 2H), 7.34-7.26 (m, 6H), 7.24-7.21 (m, 2H), 7.19-7.16 (m, 1H), 7.12-7.08 (m, 3H), 7.07-7.04 (m, 3H), 6.95-6.92 (m, 2H), 6.86 (d, *J* = 8.1 Hz, 2H), 4.68 (d, *J* = 15.0 Hz, 1H), 4.64 (s, 1H), 3.72 (d, *J* = 15.1 Hz, 1H), 3.02 (s, 3H), 2.97 (m, 1H), 2.64 (dd, *J* = 14.7, 2.2 Hz,

1H), 2.34 (d, J = 15.0 Hz, 1H), 2.27 (s, 3H), 2.19 (d, J = 15.0 Hz, 1H), 0.29 (s, 3H);

^{19}F NMR (565 MHz, $CDCl_3$) δ -84.6 (d, J = 30.5 Hz), -87.2 (d, J = 30.2 Hz);

^{13}C NMR (151 MHz, $CDCl_3$) δ 154.5 (dd, J = 291.8 Hz, J = 291.8 Hz), 142.4, 141.2, 141.0, 140.6, 140.5 (dd, J = 4.7, 2.4 Hz), 139.2, 138.1, 135.4, 129.6, 129.5 (t, J = 2.2 Hz), 129.4, 129.0, 128.7, 128.5, 128.3, 128.2, 127.8, 127.6, 127.5, 127.3, 127.2, 127.1, 89.4 (dd, J = 21.6, 14.3 Hz), 64.1, 45.3, 44.6, 40.2 (t, J = 2.2 Hz), 36.9, 36.5, 25.9, 21.3;

HRMS: (ESI) calcd for $C_{42}H_{40}F_2NO_4S_2^+$[M+H]$^+$ 724.2286; found 724.2283.

(2*R*,3*S*)-3-(3,3-difluoro-2-(4-(trifluoromethoxy)phenyl)allyl)-5-(diphenylmethylene)-3-methyl-2-phenyl-1-tosylpiperidine (79)

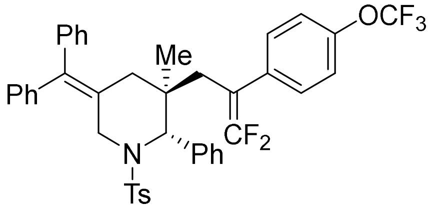

Chemical Formula: $C_{42}H_{36}F_5NO_3S$
Exact Mass: 729.2336

79 was prepared according to general procedure **2.3** using (*R*)-4-methyl-*N*-(2-methyl-1-phenylallyl)-*N*-(3-phenylprop-2-yn-1-yl)benzenesulfonamide (0.2 mmol, 83.0 mg), bromobenzene (0.2 mmol, 31.4 mg) and 1-(trifluoromethoxy)-4-(3,3,3-trifluoroprop-1-en-2-yl)benzene (0.1 mmol, 25.6 mg), and was purified by silica gel column chromatography (PE/EtOAc = 5/1) to obtain **79** as colorless oil (44.5 mg, 61% yield).

^{1}H NMR (600 MHz, $CDCl_3$) δ 7.36 (d, J = 7.5 Hz, 2H), 7.31-7.26 (m, 4H), 7.22-7.17 (m, 2H), 7.17-7.15 (m, 1H), 7.14-7.05 (m, 8H), 7.05-7.01 (m, 2H), 7.00 (d, J = 8.3 Hz, 2H), 6.92 (d, J = 8.0 Hz, 2H), 4.74 (s, 1H), 4.69 (d, J = 15.4 Hz, 1H), 3.82 (d, J = 15.4 Hz, 1H), 2.83-2.77 (m, 1H), 2.70 (dd, J = 14.7, 2.3 Hz, 1H), 2.29 (s, 3H), 2.27 (d, J = 15.5 Hz, 1H), 2.14 (d, J = 15.2 Hz, 1H), 0.26 (s, 3H);

^{19}F NMR (565 MHz, $CDCl_3$) δ -57.80, -86.52 (d, J = 34.6 Hz), -88.72 (d, J = 34.2 Hz).

^{13}C NMR (151 MHz, $CDCl_3$) δ 154.6 (dd, J = 290.6 Hz, 290.2 Hz), 148.5, 142.8, 141.5, 141.5, 140.6, 138.7, 136.1, 133.5 (dd, J = 4.4, 2.0 Hz), 130.4 (t, J = 2.5 Hz), 129.9, 129.8, 129.2, 129.2, 128.8, 128.5, 128.4, 128.1, 127.8, 127.6, 127.5, 127.3, 121.6 (q, J

= 257.2 Hz), 121.2, 89.4 (dd, J = 21.1, 15.0 Hz), 65.0, 45.6, 40.3 (t, J = 2.2 Hz), 37.2, 37.0, 26.1, 21.7;

HRMS: (ESI) calcd for $C_{42}H_{37}F_5NO_3S^+$[M+H]$^+$ 730.2334; found 730.2340.

methyl 3-(3-((2*R*,3*S*)-5-(diphenylmethylene)-3-methyl-2-phenyl-1-tosylpiperidin-3-yl)-1,1-difluoroprop-1-en-2-yl)benzoate (80)

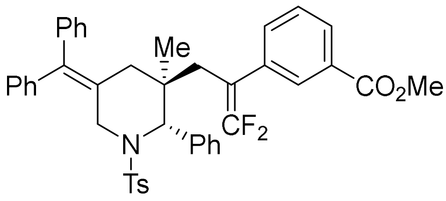

Chemical Formula: $C_{43}H_{39}F_2NO_4S$
Exact Mass: 703.2568

80 was prepared according to general procedure **2.3** using (*R*)-4-methyl-*N*-(2-methyl-1-phenylallyl)-*N*-(3-phenylprop-2-yn-1-yl)benzenesulfonamide (0.2 mmol, 83.0 mg), bromobenzene (0.2 mmol, 31.4 mg) and methyl 3-(3,3,3-trifluoroprop-1-en-2-yl)benzoate (0.1 mmol, 23.0 mg), and was purified by silica gel column chromatography (PE/EtOAc = 5/1) to obtain **80** as colorless oil (38.7 mg, 55% yield).

^{1}H NMR (600 MHz, $CDCl_3$) δ 7.90 (dt, J = 7.8, 1.4 Hz, 1H), 7.79 (d, J = 2.0 Hz, 1H), 7.39-7.32 (m, 3H), 7.31-7.27 (m, 2H), 7.26-7.24 (m, 2H), 7.18 (ddt, J = 8.4, 5.3, 2.0 Hz, 4H), 7.13-7.10 (m, 4H), 7.09-7.07 (m, 2H), 7.01-6.97 (m, 2H), 6.93-6.90 (m, 2H), 4.76 (s, 1H), 4.67 (d, J = 15.4 Hz, 1H), 3.93 (s, 3H), 3.83 (d, J = 15.5 Hz, 1H), 2.88 (dt, J = 14.7, 2.5 Hz, 1H), 2.69 (dt, J = 15.0, 1.8 Hz, 1H), 2.29 (s, 3H), 2.25 (d, J = 15.3 Hz, 1H), 2.11 (d, J = 15.2 Hz, 1H), 0.27 (s, 3H);

^{19}F NMR (565 MHz, $CDCl_3$) δ -86.3 (d, J = 34.2 Hz), -88.8 (d, J = 34.1 Hz);

^{13}C NMR (151 MHz, $CDCl_3$) δ 167.0, 154.8 (dd, J = 290.4 Hz, 290.5), 142.7, 141.5, 141.4, 140.6, 138.9, 136.1, 135.3 (dd, J = 4.4, 2.0 Hz), 133.5 (t, J = 2.0 Hz), 130.8, 129.9, 129.8, 129.2, 129.2, 129.0, 128.8, 128.4, 128.4, 128.2, 127.7, 127.5, 127.2, 89.7 (dd, J = 20.7, 14.8 Hz), 65.0, 52.5, 45.7, 40.3 (t, J = 2.2 Hz), 37.2, 37.1, 26.0, 21.7;

HRMS: (ESI) calcd for $C_{43}H_{40}F_2NO_4S^+$[M+H]$^+$ 704.2569; found 704.2573.

1-(4-(3-((2*R*,3*S*)-5-(diphenylmethylene)-3-methyl-2-phenyl-1-tosylpiperidin-3-yl)-

1,1-difluoroprop-1-en-2-yl)phenyl)ethan-1-one (81)

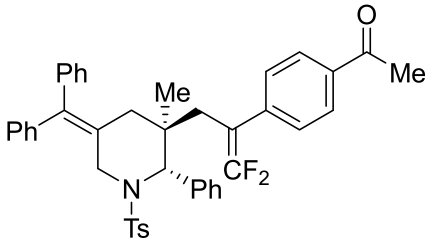

Chemical Formula: $C_{43}H_{39}F_2NO_3S$
Exact Mass: 687.2619

81 was prepared according to general procedure **2.3** using (*R*)-4-methyl-*N*-(2-methyl-1-phenylallyl)-*N*-(3-phenylprop-2-yn-1-yl)benzenesulfonamide (0.2 mmol, 83.0 mg), bromobenzene (0.2 mmol, 31.4 mg) and 1-(4-(3,3,3-trifluoroprop-1-en-2-yl)phenyl)ethan-1-one (0.1 mmol, 21.4 mg), and was purified by silica gel column chromatography (PE/EtOAc = 5/1) to obtain **81** as colorless oil (43.2 mg, 63% yield).

^{1}H NMR (600 MHz, $CDCl_3$) δ 7.86 – 7.82 (m, 2H), 7.37 (t, *J* = 7.6 Hz, 2H), 7.31 – 7.26 (m, 4H), 7.21 – 7.18 (m, 2H), 7.16 (dt, *J* = 5.7, 2.8 Hz, 1H), 7.13 – 7.08 (m, 6H), 7.07 (d, *J* = 8.1 Hz, 2H), 7.04 – 7.00 (m, 2H), 6.92 (d, *J* = 8.1 Hz, 2H), 4.76 (s, 1H), 4.70 (d, *J* = 15.3 Hz, 1H), 3.81 (d, *J* = 15.4 Hz, 1H), 2.87 (dt, *J* = 14.7, 2.6 Hz, 1H), 2.76 – 2.70 (m, 1H), 2.58 (s, 3H), 2.29 (s, 3H), 2.26 (d, *J* = 15.2 Hz, 1H), 2.15 (d, *J* = 15.2 Hz, 1H), 0.25 (s, 3H);

^{19}F NMR (565 MHz, $CDCl_3$) δ -85.3 (d, *J* = 31.5 Hz), -87.6 (d, *J* = 31.9 Hz);

^{13}C NMR (151 MHz, $CDCl_3$) δ 197.6, 155.3 (dd, *J* = 291.1 Hz, *J* = 291.4 Hz). 142.4, 141.1, 141.1, 140.3, 139.7 (dd, *J* = 4.3, 2.2 Hz), 138.4, 135.8, 135.8, 129.6, 129.4, 128.9, 128.8, 128.8 (t, *J* = 2.6 Hz), 128.5, 128.5, 128.1, 128.1, 127.8, 127.4, 127.2, 127.2, 126.9, 89.7 (dd, *J* = 21.0, 14.4 Hz), 64.6, 45.3, 40.0 (t, *J* = 2.2 Hz), 36.7, 36.6, 26.6, 25.9, 21.4;

HRMS: (ESI) calcd for $C_{43}H_{40}F_2NO_3S^+$[M+H]$^+$ 688.2613; found 688.2617.

4-(3-((2*R*,3*S*)-5-(diphenylmethylene)-3-methyl-2-phenyl-1-tosylpiperidin-3-yl)-1,1-difluoroprop-1-en-2-yl)-*N*,*N*-dimethylbenzamide (82)

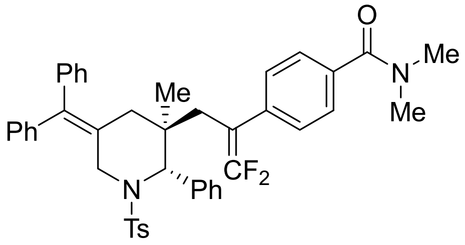

82 was prepared according to general procedure **2.3** using (*R*)-4-methyl-*N*-(2-methyl-1-phenylallyl)-*N*-(3-phenylprop-2-yn-1-yl)benzenesulfonamide (0.2 mmol, 83.0 mg), bromobenzene (0.2 mmol, 31.4 mg) and *N*,*N*-dimethyl-4-(3,3,3-trifluoroprop-1-en-2-yl)benzamide (0.1 mmol, 24.3 mg), and was purified by silica gel column chromatography (PE/EtOAc = 5/1) to obtain **82** as colorless oil (48.0 mg, 67% yield).

^{1}H NMR (600 MHz, $CDCl_3$) δ 7.39-7.33 (m, 4H), 7.31 (dd, *J* = 8.0, 1.7 Hz, 2H), 7.30-7.27 (m, 2H), 7.23-7.20 (m, 2H), 7.18-7.14 (m, 1H), 7.11-7.04 (m, 8H), 7.00 (d, *J* = 8.2 Hz, 2H), 6.88 (d, *J* = 8.0 Hz, 2H), 4.74 (s, 1H), 4.69 (d, *J* = 15.0 Hz, 1H), 3.76 (d, *J* = 15.1 Hz, 1H), 3.09 (s, 3H), 2.96 (s, 3H), 2.90 (dt, *J* = 14.7, 2.9 Hz, 1H), 2.65 (dd, *J* = 14.7, 2.2 Hz, 1H), 2.32 (d, *J* = 15.4 Hz, 1H), 2.28 (s, 3H), 2.21 (d, *J* = 15.0 Hz, 1H), 0.26 (s, 3H);

^{19}F NMR (565 MHz, $CDCl_3$) δ -86.32 (d, *J* = 33.9 Hz), -88.56 (d, *J* = 34.0 Hz);

^{13}C NMR (151 MHz, $CDCl_3$) δ 171.2, 154.4 (dd, *J* = 290.8 Hz, 290.5 Hz), 142.3, 141.2, 141.1, 140.3, 138.3, 135.9 (dd, *J* = 4.2, 2.6 Hz)., 135.6, 135.1, 129.6, 129.4, 128.9, 128.7, 128.6 (t, *J* = 2.8 Hz), 128.5, 128.2, 128.1, 127.7, 127.4, 127.4, 127.2, 127.0, 89.6 (dd, *J* = 20.8, 14.8 Hz), 64.3, 45.3, 40.0 (t, *J* = 2.3 Hz), 39.6, 36.8, 36.5, 35.4, 26.1, 21.3;

HRMS: (ESI) calcd for $C_{44}H_{43}F_2N_2O_3S^+$[M+H]$^+$ 717.2880; found 717.2883.

4-(1,1-difluoro-3-((2*R*,3*S*)-3-methyl-2,5,6-triphenyl-1-tosyl-2,3,4,7-tetrahydro-1*H*-azepin-3-yl)prop-1-en-2-yl)benzonitrile (83)

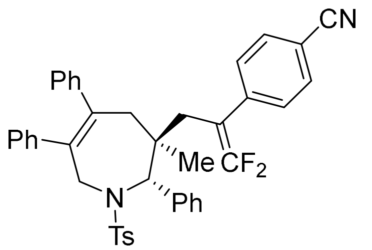

Chemical Formula: $C_{42}H_{36}F_2N_2O_2S$
Exact Mass: 670.2466

83 was prepared according to general procedure **2.4** using (*R*)-4-methyl-*N*-(2-methyl-1-phenylallyl)-*N*-(3-phenylprop-2-yn-1-yl)benzenesulfonamide (0.2 mmol, 83.0 mg), bromobenzene (0.2 mmol, 31.4 mg) and 4-(3,3,3-trifluoroprop-1-en-2-yl)benzonitrile (0.1 mmol, 19.7 mg), and was purified by silica gel column chromatography (PE/EtOAc = 5/1) to obtain **83** as white solid (36.9 mg, 55% yield).

^{1}H NMR (600 MHz, $CDCl_3$) δ 7.57 (d, *J* = 8.3 Hz, 2H), 7.33-7.29 (m, 4H), 7.25 (d, *J* = 7.5 Hz, 1H), 7.20 (t, *J* = 7.4 Hz, 2H), 7.16 (d, *J* = 7.2 Hz, 2H), 7.10 (dd, *J* = 8.0, 6.6 Hz, 2H), 7.07-7.05 (m, 1H), 7.04-7.00 (m, 5H), 6.99 (d, *J* = 8.0 Hz, 2H), 6.72-6.68 (m, 2H), 5.01 (s, 1H), 4.71 (d, *J* = 18.9 Hz, 1H), 4.40 (dd, *J* = 19.0, 3.0 Hz, 1H), 2.87-2.78 (m, 2H), 2.61 (dt, *J* = 14.6, 2.7 Hz, 1H), 2.45 (d, *J* = 17.0 Hz, 1H), 2.29 (s, 3H), 0.99 (s, 3H);

^{19}F NMR (565 MHz, $CDCl_3$) δ -84.6 (d, *J* = 30.1 Hz), -87.2 (d, *J* = 29.9 Hz);

^{13}C NMR (151 MHz, $CDCl_3$) δ 154.9 (dd, *J* = 292.3 Hz, 292.9 Hz), 144.6, 142.9, 141.1, 139.9 (dd, *J* = 4.5, 2.7 Hz), 137.8, 137.1, 136.5, 136.2, 132.3, 129.6, 129.4 (t, *J* = 2.9 Hz), 129.2, 129.1, 128.9, 128.2, 128.0, 127.8, 127.6, 127.1, 126.6, 126.0, 118.5, 111.2, 89.4 (dd, *J* = 21.8, 13.9 Hz), 67.6, 51.3, 45.3, 44.5 (t, *J* = 2.5 Hz), 44.5, 39.7, 21.9, 21.4;

HRMS: (ESI) calcd for $C_{42}H_{37}F_2N_2O_2S^+$ $[M+H]^+$ 671.2461; found 671.2465.

methyl 4-(1,1-difluoro-3-((2R,3S)-3-methyl-2,5,6-triphenyl-1-tosyl-2,3,4,7-tetrahydro-1H-azepin-3-yl)prop-1-en-2-yl)benzoate (84)

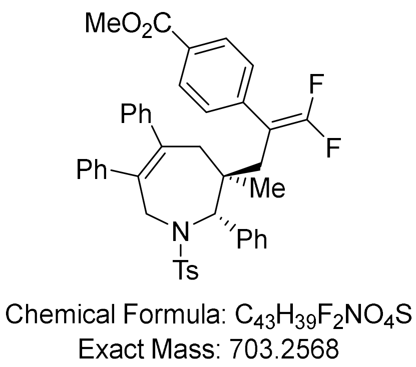

84 was prepared according to general procedure **2.3** using (*R*)-4-methyl-*N*-(2-methyl-1-phenylallyl)-*N*-(2-methyl-3-phenyl-2l5-prop-2-yn-1-yl)benzenesulfonamide (0.2 mmol, 83.0 mg), bromobenzene (0.2 mmol, 31.4 mg), and methyl 4-(3,3,3-trifluoroprop-1-en-2-yl)benzoate (0.1 mmol, 23.0 mg), and was purified by silica gel column chromatography (PE/EtOAc = 5/1) to obtain **84** as colorless oil (30.2 mg, 43% yield).

^{1}H NMR (600 MHz, $CDCl_3$) δ 7.95 (d, *J* = 8.3 Hz, 2H), 7.34 (d, *J* = 8.2 Hz, 2H), 7.30-7.27 (m, 2H), 7.25-7.22 (m, 1H), 7.21-7.14 (m, 4H), 7.11-7.07 (m, 2H), 7.07-7.04 (m, 1H), 7.03-7.01 (m, 2H), 7.01-6.95 (m, 5H), 6.70 (dd, *J* = 7.4, 2.1 Hz, 2H), 5.00 (s, 1H), 4.76 (d, *J* = 18.9 Hz, 1H), 4.41 (dd, *J* = 19.1, 3.1 Hz, 1H), 3.91 (s, 3H), 2.86-2.76 (m, 2H), 2.63-2.57 (m, 1H), 2.47 (d, *J* = 17.0 Hz, 1H), 2.28 (s, 3H), 0.99 (s, 3H);

^{13}C NMR (151 MHz, $CDCl_3$) δ 166.7, 154.3 (dd, *J* = 291.1 Hz, 291.1 Hz), 142.4, 141.1, 141.1, 140.2, 139.5 (dd, *J* = 4.3, 2.4 Hz), 138.4, 135.8, 129.7, 129.6, 129.4, 128.91, 128.83, 128.82, 128.6 (t, *J* = 2.7 Hz), 128.5, 128.1, 128.1, 127.8, 127.4, 127.2, 127.2, 126.9, 89.7 (dd, *J* = 20.9, 14.5 Hz), 64.7, 52.1, 45.3, 40.0 (t, *J* = 2.4 Hz), 36.7, 25.8, 21.4;

^{19}F NMR (565 MHz, $CDCl_3$) δ -85.8 (d, *J* = 32.1 Hz), -88.0 (d, *J* = 32.1 Hz);

HRMS: (ESI) calcd for $C_{43}H_{40}F_2NO_4S^+$[M+H]$^+$ 704.2584; found 704.2588.

(2*R*,3*S*)-3-(3,3-difluoro-2-(4-(methylsulfonyl)phenyl)allyl)-3-methyl-2,5,6-triphenyl-1-tosyl-2,3,4,7-tetrahydro-1*H*-azepine (85)

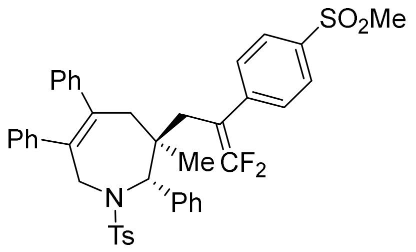

Chemical Formula: $C_{42}H_{39}F_2NO_4S_2$
Exact Mass: 723.2289

85 was prepared according to general procedure **2.4** using (*R*)-4-methyl-*N*-(2-methyl-1-phenylallyl)-*N*-(3-phenylprop-2-yn-1-yl)benzenesulfonamide (0.2 mmol, 83.0 mg), bromobenzene (0.2 mmol, 31.4 mg) and 1-(methylsulfonyl)-4-(3,3,3-trifluoroprop-1-en-2-yl)benzene (0.1 mmol, 25.0 mg), and was purified by silica gel column chromatography (PE/EtOAc = 5/1) to obtain **85** as colorless oil (36.9 mg, 51% yield).

^{1}H NMR (600 MHz, $CDCl_3$) δ 7.86 (d, *J* = 8.4 Hz, 2H), 7.46 – 7.41 (m, 2H), 7.28 (d, *J* = 8.3 Hz, 2H), 7.25 – 7.23 (m, 1H), 7.19 (dd, *J* = 8.4, 6.8 Hz, 2H), 7.16 – 7.13 (m, 2H), 7.11 – 7.08 (m, 2H), 7.07 – 7.00 (m, 6H), 6.98 (d, *J* = 7.9 Hz, 2H), 6.76 – 6.71 (m, 2H), 4.99 (d, *J* = 1.2 Hz, 1H), 4.71 (d, *J* = 18.9 Hz, 1H), 4.41 (dd, *J* = 18.9, 2.9 Hz, 1H), 2.95 (s, 3H), 2.84 (dt, *J* = 16.4, 3.2 Hz, 2H), 2.61 (dt, *J* = 14.6, 2.6 Hz, 1H), 2.44 (d, *J* = 16.6 Hz, 1H), 2.28 (s, 3H), 1.01 (s, 3H);

^{19}F NMR (565 MHz, $CDCl_3$) δ -84.6 (d, *J* = 30.1 Hz), -87.2 (d, *J* = 30.2 Hz);

^{13}C NMR (151 MHz, $CDCl_3$) δ 155.9 (dd, *J* = 291.4 Hz, 293.0 Hz), 144.7, 142.8, 141.0, 140.9 (dd, *J* = 4.6, 2.7 Hz), 139.3, 137.8, 137.0, 136.5, 136.3, 129.6 (t, *J* = 3.7 Hz), 129.1, 129.1, 128.9, 128.2, 128.0, 127.8, 127.7, 127.6, 127.0, 126.6, 126.0, 89.2 (dd, *J* = 21.6, 14.0 Hz), 67.5, 51.3, 45.3, 44.5 (t, *J* = 2.4 Hz), 44.5, 39.9, 22.0, 21.4;

HRMS: (ESI) calcd for $C_{42}H_{40}F_2NO_4S_2^+$[M+H]$^+$ 724.2294; found 724.2294.

1-(4-(1,1-difluoro-3-((2*R*,3*S*)-3-methyl-2,5,6-triphenyl-1-tosyl-2,3,4,7-tetrahydro-1*H*-azepin-3-yl)prop-1-en-2-yl)phenyl)ethan-1-one (86)

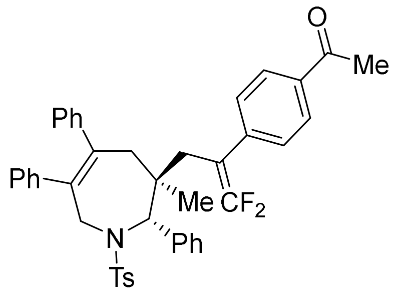

Chemical Formula: $C_{43}H_{39}F_2NO_3S$
Exact Mass: 687.2619

86 was prepared according to general procedure **2.4** using (*R*)-4-methyl-*N*-(2-methyl-1-phenylallyl)-*N*-(3-phenylprop-2-yn-1-yl)benzenesulfonamide (0.2 mmol, 83.0 mg), bromobenzene (0.2 mmol, 31.4 mg) and 1-(4-(3,3,3-trifluoroprop-1-en-2-yl)phenyl)ethan-1-one (0.1 mmol, 21.4 mg), and was purified by silica gel column chromatography (PE/EtOAc = 5/1) to obtain **86** as colorless oil (39.2 mg, 57% yield).

^{1}H NMR (600 MHz, $CDCl_3$) δ 7.87 (d, *J* = 8.4 Hz, 2H), 7.32 (t, *J* = 7.9 Hz, 4H), 7.25-7.23 (m, 1H), 7.21-7.17 (m, 4H), 7.08 (dd, *J* = 7.2, 1.2 Hz, 2H), 7.06-7.04 (m, 1H), 7.03-7.01 (m, 2H), 7.00-6.96 (m, 5H), 6.70-6.67 (m, 2H), 5.01 (s, 1H), 4.77-4.72 (m, 1H), 4.42 (dd, *J* = 19.0, 3.1 Hz, 1H), 2.85-2.78 (m, 2H), 2.62 (dt, *J* = 14.6, 2.5 Hz, 1H), 2.56 (s, 3H), 2.46 (d, *J* = 17.0 Hz, 1H), 2.28 (s, 3H), 1.01 (s, 3H);

^{19}F NMR (565 MHz, $CDCl_3$) δ -85.6 (d, *J* = 31.9 Hz), -87.9 (d, *J* = 32.2 Hz);

^{13}C NMR (151 MHz, $CDCl_3$) δ 197.4, 155.8 (dd, *J* = 292.4 Hz, 291.1 Hz), 144.6, 142.8, 141.1, 139.9 (dd, *J* = 4.4, 2.7 Hz), 138.0, 137.2, 136.6, 136.1, 135.9, 129.6, 129.1, 129.1, 129.0, 128.8 (t, *J* = 2.7 Hz), 128.5, 128.1, 128.0, 127.7, 127.5, 127.1, 126.5, 125.8, 89.6 (dd, *J* = 20.9, 14.1 Hz), 67.8, 51.4, 45.1, 44.5 (t, *J* = 2.6 Hz), 39.9, 26.6, 21.4, 21.4;

HRMS: (ESI) calcd for $C_{43}H_{40}F_2NO_3S^+$[M+H]$^+$ 688.2624; found 688.2623.

3-benzyl-3-methyl-4,5-diphenyl-1-tosyl-1,2,3,6-tetrahydropyridine (87)

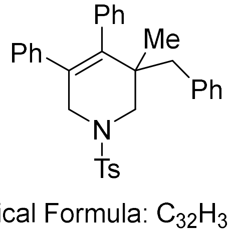

Chemical Formula: $C_{32}H_{31}NO_2S$
Exact Mass: 493.2075

87 was prepared according to general procedure **2.5** using 4-methyl-*N*-(2-methylallyl)-*N*-(3-phenylprop-2-yn-1-yl)benzenesulfonamide (0.1 mmol, 33.9 mg) and bromobenzene (0.4 mmol, 62.8 mg), and was purified by silica gel column chromatography (PE/EtOAc = 10/1) to obtain **87** as white solid (25.6 mg, 52% yield).

^{1}H NMR (400 MHz, $CDCl_3$) δ 7.70- 7.63 (m, 2H), 7.36-7.27 (m, 5H), 7.24 (d, *J* = 7.0 Hz, 0H), 7.14-7.02 (m, 5H), 6.94 (dt, *J* = 7.7, 1.5 Hz, 4H), 4.16 (d, *J* = 16.1 Hz, 1H), 3.57 (d, *J* = 11.4 Hz, 1H), 3.50 (d, *J* = 16.0 Hz, 1H), 3.18 (d, *J* = 13.2 Hz, 1H), 2.74 (d, *J* = 13.2 Hz, 1H), 2.45 (s, 3H), 2.41 (d, *J* = 11.4 Hz, 1H), 0.89 (s, 3H).

^{13}C NMR (151 MHz, $CDCl_3$) δ 143.7, 142.8, 139.3, 137.9, 137.1, 132.0, 132.0, 131.3, 131.1, 129.7, 129.1, 128.1, 127.9, 127.7, 127.2, 126.7, 126.4, 126.3, 52.0, 50.4, 42.2, 40.2, 22.6, 21.6.

HRMS: (ESI) calcd for $C_{32}H_{32}NO_2S^+$[M+H]$^+$ 494.2148; found 494.2150.

3-(4-methoxybenzyl)-5-(4-methoxyphenyl)-3-methyl-4-phenyl-1-tosyl-1,2,3,6-tetrahydropyridine (88)

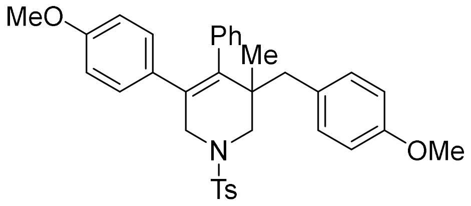

Chemical Formula: $C_{34}H_{35}NO_4S$
Exact Mass: 553.2287

88 was prepared according to general procedure **2.5** using 4-methyl-*N*-(2-methylallyl)-*N*-(3-phenylprop-2-yn-1-yl)benzenesulfonamide (0.1 mmol, 33.9 mg), 1-bromo-4-methoxybenzene (0.4 mmol, 74.8 mg), and was purified by silica gel column chromatography (PE/EtOAc = 20/1) to obtain **88** as white solid (35.4 mg, 64% yield).

^{1}H NMR (600 MHz, $CDCl_3$) δ 7.69-7.64 (m, 2H), 7.36-7.31 (m, 2H), 7.21-7.16 (m, 2H), 7.15-7.06 (m, 3H), 6.95-6.90 (m, 2H), 6.88-6.79 (m, 4H), 6.61-6.56 (m, 2H), 4.13 (d, *J* = 16.1 Hz, 1H), 3.80 (s, 3H), 3.67 (s, 3H), 3.54 (d, *J* = 11.5 Hz, 1H), 3.44 (d, *J* = 16.1 Hz, 1H), 3.11 (d, *J* = 13.4 Hz, 1H), 2.66 (d, *J* = 13.4 Hz, 1H), 2.44 (s, 3H), 2.36 (d, *J* = 11.4 Hz, 1H), 0.85 (s, 3H);

^{13}C NMR (151 MHz, $CDCl_3$) δ 158.1, 158.0, 143.7, 142.6, 138.2, 132.3, 132.0, 131.6, 131.3, 131.1, 130.2, 129.7, 129.1, 128.1, 127.2, 126.3, 113.3, 113.1, 55.2, 55.0, 51.8, 50.5, 41.2, 40.2, 22.6, 21.5.

HRMS: (ESI) calcd for $C_{34}H_{36}NO_4S^+[M+H]^+$ 554.2360; found 554.2348.

5-([1,1'-biphenyl]-4-yl)-3-([1,1'-biphenyl]-4-ylmethyl)-3-methyl-4-phenyl-1-tosyl-1,2,3,6-tetrahydropyridine (89)

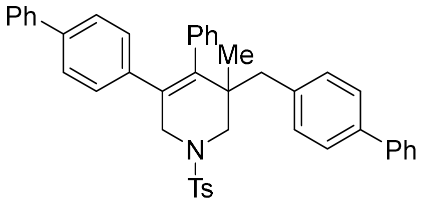

Chemical Formula: $C_{44}H_{39}NO_2S$
Exact Mass: 645.2702

89 was prepared according to general procedure **2.5** using 4-methyl-*N*-(2-methylallyl)-*N*-(3-phenylprop-2-yn-1-yl)benzenesulfonamide (0.1 mmol, 33.9 mg), 4-bromo-1,1'-biphenyl (0.4 mmol, 93.2 mg), and was purified by silica gel column chromatography (PE/EtOAc = 20/1) to obtain **89** as white solid (41.9 mg, 65% yield).

^{1}H NMR (600 MHz, $CDCl_3$) δ 7.76-7.69 (m, 2H), 7.66-7.61 (m, 2H), 7.59-7.54 (m, 2H), 7.51-7.42 (m, 4H), 7.42-7.28 (m, 10H), 7.20-7.14 (m, 2H), 7.14-7.09 (m, 1H), 7.07-6.98 (m, 4H), 4.26 (d, *J* = 16.1 Hz, 1H), 3.67 (d, *J* = 11.5 Hz, 1H), 3.55 (d, *J* = 16.1 Hz, 1H), 3.27 (d, *J* = 13.1 Hz, 1H), 2.81 (d, *J* = 13.1 Hz, 1H), 2.48 (d, *J* = 11.5 Hz, 1H), 2.46 (s, 3H), 0.96 (s, 3H);

13C NMR (151 MHz, $CDCl_3$) δ 143.8, 142.9, 141.0, 140.4, 139.3, 139.1, 138.3, 137.9, 136.2, 132.0, 131.8, 131.7, 131.1, 129.7, 129.5, 128.7, 128.6, 128.1, 127.3, 127.2, 127.0, 127.0, 126.8, 126.6, 126.5, 126.3, 51.9, 50.4, 41.8, 40.4, 22.7, 21.5.

HRMS: (ESI) calcd for $C_{44}H_{40}NO_2S^+[M+H]^+$ 646.2774; found 646.2756.

3-(4-fluorobenzyl)-5-(4-fluorophenyl)-3-methyl-4-phenyl-1-tosyl-1,2,3,6-tetrahydropyridine (90)

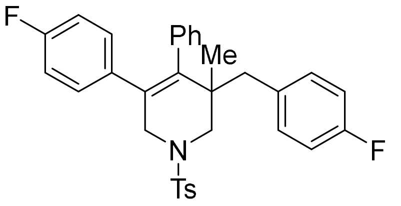

Chemical Formula: $C_{32}H_{29}F_2NO_2S$
Exact Mass: 529.1887

90 was prepared according to general procedure **2.5** using 4-methyl-*N*-(2-methylallyl)-*N*-(3-phenylprop-2-yn-1-yl)benzenesulfonamide (0.1 mmol, 33.9 mg), 1-bromo-4-fluorobenzene (0.4 mmol, 70.0 mg), and was purified by silica gel column chromatography (PE/EtOAc = 20/1) to obtain **90** as white solid (31.2 mg, 59% yield).
^{1}H NMR (600 MHz, $CDCl_3$) δ 7.70-7.65 (m, 2H), 7.37-7.32 (m, 2H), 7.26-7.22 (m, 2H), 7.16-7.08 (m, 3H), 7.02-6.95 (m, 2H), 6.93-6.85 (m, 4H), 6.78-6.72 (m, 2H), 4.17 (d, *J* = 16.1 Hz, 1H), 3.58 (d, *J* = 11.5 Hz, 1H), 3.40 (d, *J* = 16.1 Hz, 1H), 3.18 (d, *J* = 13.4 Hz, 1H), 2.70 (d, *J* = 13.3 Hz, 1H), 2.45 (s, 3H), 2.32 (d, *J* = 11.4 Hz, 1H), 0.83 (s, 3H);
^{19}F NMR (565 MHz, $CDCl_3$) δ -114.80--115.22 (m), -116.54--117.28 (m);
^{13}C NMR (151 MHz, $CDCl_3$) δ 162.3 (d, *J* = 56.5 Hz), 160.7 (d, *J* = 58.5 Hz), 143.9, 143.1, 137.6, 135.0 (d, *J* = 3.3 Hz), 132.8 (d, *J* = 7.7 Hz), 132.5 (d, *J* = 3.0 Hz), 131.7, 131.2, 130.9, 130.7 (d, *J* = 8.1 Hz), 129.7, 128.0, 127.4, 126.6, 114.8, 114.6, 51.5, 50.4, 41.0, 40.1, 22.4, 21.6.
HRMS: (ESI) calcd for $C_{32}H_{30}F_2NO_2S^+$[M+H]$^+$ 530.1960; found 530.1940.

3-methyl-4-phenyl-1-tosyl-3-(4-(trifluoromethyl)benzyl)-5-(4-(trifluoromethyl)phenyl)-1,2,3,6-tetrahydropyridine (91)

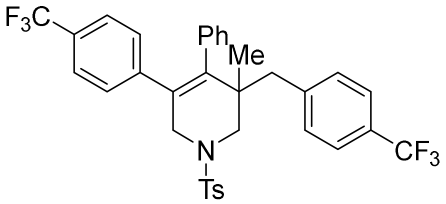

Chemical Formula: $C_{34}H_{29}F_6NO_2S$
Exact Mass: 629.1823

91 was prepared according to general procedure **2.5** using 4-methyl-*N*-(2-methylallyl)-*N*-(3-phenylprop-2-yn-1-yl)benzenesulfonamide (0.1 mmol, 33.9 mg), 1-bromo-4-

(trifluoromethyl)benzene (0.4 mmol, 90.0 mg), and was purified by silica gel column chromatography (PE/EtOAc = 20/1) to obtain **91** as white solid (37.1 mg, 59% yield).

^{1}H NMR (600 MHz, CDCl$_3$) δ 7.71-7.64 (m, 2H), 7.56 (d, J = 8.0 Hz, 2H), 7.43 (d, J = 8.0 Hz, 2H), 7.38-7.31 (m, 4H), 7.18-7.10 (m, 3H), 7.09-7.05 (m, 2H), 6.96-6.90 (m, 2H), 4.23 (d, J = 16.1 Hz, 1H), 3.62 (d, J = 11.6 Hz, 1H), 3.41 (d, J = 16.1 Hz, 1H), 3.31 (d, J = 13.0 Hz, 1H), 2.81 (d, J = 13.1 Hz, 1H), 2.45 (s, 3H), 2.34 (d, J = 11.6 Hz, 1H), 0.85 (s, 3H);

^{19}F NMR (565 MHz, CDCl$_3$) δ -62.30, -62.64;

^{13}C NMR (151 MHz, CDCl$_3$) δ 144.1, 143.6, 142.9, 141.0, 137.0, 131.68, 131.67, 131.4, 130.8, 129.8, 129.4, 128.9 (q, J = 32.5 Hz), 128.7 (q, J = 32.2 Hz), 128.1, 127.6, 127.0, 124.9 (q, J = 3.6 Hz)， 124.8 (q, J = 3.8 Hz), 124.4 (q, J = 271.7 Hz), 123.9 (q, J = 272.2 Hz), 51.5, 50.2, 41.6, 40.4, 22.4, 21.6.

HRMS: (ESI) calcd for $C_{34}H_{30}F_6NO_2S^+[M+H]^+$ 630.1896; found 630.1888.

Ethyl 4-(5-(4-(ethoxycarbonyl)benzyl)-5-methyl-4-phenyl-1-tosyl-1,2,5,6-tetrahydropyridin-3-yl)benzoate (92)

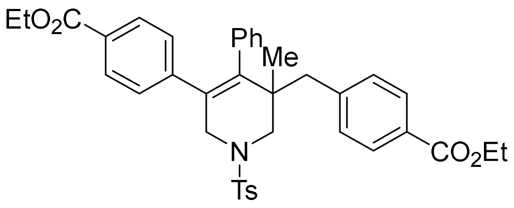

Chemical Formula: $C_{38}H_{39}NO_6S$
Exact Mass: 637.2498

92 was prepared according to general procedure **2.5** using 4-methyl-*N*-(2-methylallyl)-*N*-(3-phenylprop-2-yn-1-yl)benzenesulfonamide (0.1 mmol, 33.9 mg), ethyl 4-bromobenzoate (0.4 mmol, 91.6 mg), and was purified by silica gel column chromatography (PE/EtOAc = 20/1) to obtain **92** as white solid (29.9 mg, 47% yield).

^{1}H NMR (600 MHz, CDCl$_3$) δ 7.98 (d, J = 8.3 Hz, 2H), 7.74 (d, J = 8.4 Hz, 2H), 7.66 (d, J = 8.3 Hz, 2H), 7.35 (dd, J = 8.0, 6.0 Hz, 4H), 7.15-7.07 (m, 3H), 7.04-6.99 (m, 2H), 6.95-6.90 (m, 2H), 4.37 (q, J = 7.1 Hz, 2H), 4.29 (q, J = 7.1 Hz, 2H), 4.18 (d, J = 16.1 Hz, 1H), 3.56 (d, J = 11.5 Hz, 1H), 3.46 (d, J = 16.2 Hz, 1H), 3.26 (d, J = 13.0 Hz,

1H), 2.79 (d, J = 13.0 Hz, 1H), 2.45 (s, 3H), 2.37 (d, J = 11.5 Hz, 1H), 1.40 (t, J = 7.1 Hz, 3H), 1.33 (t, J = 7.1 Hz, 3H), 0.86 (s, 3H);

^{13}C NMR (151 MHz, $CDCl_3$) δ 166.8, 166.2, 144.00, 143.97, 143.4, 142.4, 137.2, 131.8, 131.7, 131.4, 130.9, 129.8, 129.2, 129.1, 128.8, 128.7, 128.1, 127.6, 126.9, 60.95, 60.87, 51.8, 50.1, 42.0, 40.4, 22.6, 21.6, 14.4, 14.3.

HRMS: (ESI) calcd for $C_{38}H_{40}NO_6S^+[M+H]^+$ 638.2571; found 638.2554.

3-(3-fluorobenzyl)-5-(3-fluorophenyl)-3-methyl-4-phenyl-1-tosyl-1,2,3,6-tetrahydropyridine (93)

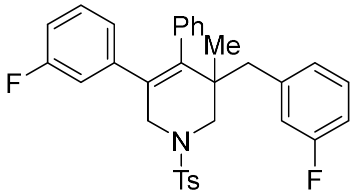

Chemical Formula: $C_{32}H_{29}F_2NO_2S$
Exact Mass: 529.1887

93 was prepared according to general procedure **2.5** using 4-methyl-*N*-(2-methylallyl)-*N*-(3-phenylprop-2-yn-1-yl)benzenesulfonamide (0.1 mmol, 33.9 mg), 1-bromo-3-fluorobenzene (0.4 mmol, 70.0 mg), and was purified by silica gel column chromatography (PE/EtOAc = 20/1) to obtain **93** as white solid (27.0 mg, 51% yield).

^{1}H NMR (600 MHz, $CDCl_3$) δ 7.71-7.63 (m, 2H), 7.41-7.33 (m, 2H), 7.29-7.26 (m, 1H), 7.18-7.07 (m, 4H), 7.06-7.00 (m, 1H), 6.99-6.90 (m, 4H), 6.77-6.69 (m, 2H), 6.67-6.61 (m, 1H), 4.13 (d, J = 16.1 Hz, 1H), 3.55 (d, J = 11.5 Hz, 1H), 3.47 (d, J = 16.2 Hz, 1H), 3.18 (d, J = 13.2 Hz, 1H), 2.72 (d, J = 13.2 Hz, 1H), 2.45 (s, 3H), 2.41 (d, J = 11.5 Hz, 1H), 0.88 (s, 3H);

^{19}F NMR (565 MHz, $CDCl_3$) δ -113.41--113.72 (m), -113.84--114.13 (m);

^{13}C NMR (151 MHz, Chloroform-*d*) δ 163.2 (d, J = 63.1 Hz), 161.5 (d, J = 63.8 Hz), 144.0, 143.3, 141.4 (d, J = 7.6 Hz), 139.5 (d, J = 7.1 Hz), 137.3, 131.9, 131.3 (d, J = 1.9 Hz), 130.9, 129.8, 129.4 (d, J = 8.6 Hz), 129.3 (d, J = 8.7 Hz), 128.1, 127.5, 127.1 (d, J = 2.7 Hz), 126.8, 124.9 (d, J = 2.8 Hz), 118.0 (d, J = 20.8 Hz), 116.1 (d, J = 21.7 Hz), 113.8 (d, J = 21.0 Hz), 113.4 (d, J = 20.9 Hz), 51.9, 50.2, 41.8, 40.2, 22.6, 21.6.

HRMS: (ESI) calcd for $C_{32}H_{30}F_2NO_2S^+[M+H]^+$ 530.1960; found 530.1945.

5-(benzo[d][1,3]dioxol-5-yl)-3-(benzo[*d*][1,3]dioxol-5-ylmethyl)-3-methyl-4-phenyl-1-tosyl-1,2,3,6-tetrahydropyridine (94)

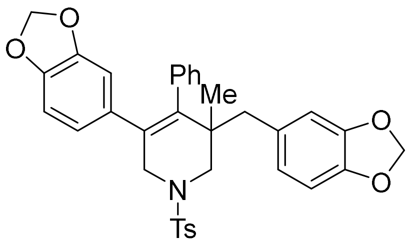

Chemical Formula: $C_{34}H_{31}NO_6S$
Exact Mass: 581.1872

94 was prepared according to general procedure **2.5** using 4-methyl-*N*-(2-methylallyl)-*N*-(3-phenylprop-2-yn-1-yl)benzenesulfonamide (0.1 mmol, 33.9 mg), 5-bromobenzo[*d*][1,3]dioxole (0.4 mmol, 80.4 mg), and was purified by silica gel column chromatography (PE/EtOAc = 20/1) to obtain **94** as white solid (27.9 mg, 48% yield).

^{1}H NMR (600 MHz, $CDCl_3$) δ 7.67 (d, *J* = 8.2 Hz, 2H), 7.34 (d, *J* = 8.0 Hz, 2H), 7.17-7.08 (m, 3H), 6.94-6.90 (m, 2H), 6.77-6.71 (m, 3H), 6.50 (d, *J* = 8.0 Hz, 1H), 6.42 (d, *J* = 1.6 Hz, 1H), 6.39 (dd, *J* = 8.0, 1.7 Hz, 1H), 5.93 (dd, *J* = 9.1, 1.5 Hz, 2H), 5.81 (s, 2H), 4.07 (d, *J* = 16.1 Hz, 1H), 3.53 (d, *J* = 11.4 Hz, 1H), 3.43 (d, *J* = 16.1 Hz, 1H), 3.07 (d, *J* = 13.4 Hz, 1H), 2.62 (d, *J* = 13.4 Hz, 1H), 2.45 (s, 3H), 2.39 (d, *J* = 11.4 Hz, 1H), 0.86 (s, 3H);

^{13}C NMR (151 MHz, $CDCl_3$) δ 147.2, 146.9, 146.1, 143.7, 142.8, 137.9, 133.1, 132.0, 131.5, 130.9, 130.6, 129.7, 128.1, 127.3, 126.4, 124.4, 122.6, 111.6, 109.5, 107.8, 107.7, 100.8, 100.7, 51.9, 50.5, 41.8, 40.2, 22.6, 21.6.

HRMS: (ESI) calcd for $C_{34}H_{32}NO_6S^+$[M+H]$^+$ 582.1945; found 582.1940.

2-((3-methyl-4-phenyl-5-(9-phenyl-9*H*-carbazol-3-yl)-1-tosyl-1,2,3,6-tetrahydropyridin-3-yl)methyl)-9-phenyl-9*H*-carbazole (95)

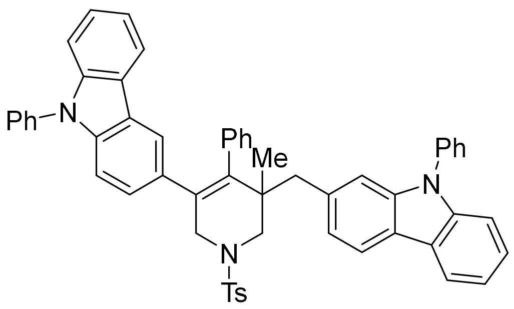

Chemical Formula: $C_{56}H_{45}N_3O_2S$
Exact Mass: 823.3232

95 was prepared according to general procedure **2.5** using 4-methyl-*N*-(2-methylallyl)-*N*-(3-phenylprop-2-yn-1-yl)benzenesulfonamide (0.1 mmol, 33.9 mg), 3-bromo-9-phenyl-9*H*-carbazole (0.4 mmol, 128.8 mg), and was purified by silica gel column chromatography (PE/EtOAc = 20/1) to obtain **95** as white solid (32.9 mg, 66% yield).

^{1}H NMR (600 MHz, $CDCl_3$) δ 8.28-8.21 (m, 2H), 8.06-8.01 (m, 1H), 7.81 (dd, *J* = 1.7, 0.6 Hz, 1H), 7.73-7.68 (m, 2H), 7.64-7.58 (m, 4H), 7.58-7.54 (m, 2H), 7.49-7.39 (m, 6H), 7.39-7.33 (m, 3H), 7.33-7.28 (m, 4H), 7.27-7.26 (m, 1H), 7.17-7.04 (m, 6H), 7.02 (dd, *J* = 8.4, 1.7 Hz, 1H), 4.41 (d, *J* = 16.0 Hz, 1H), 3.82 (d, *J* = 11.4 Hz, 1H), 3.61 (d, *J* = 16.0 Hz, 1H), 3.50 (d, *J* = 13.4 Hz, 1H), 2.97 (d, *J* = 13.4 Hz, 1H), 2.45 (d, *J* = 11.5 Hz, 1H), 2.43 (s, 3H), 0.98 (s, 3H);

^{13}C NMR (151 MHz, $CDCl_3$) δ 143.6, 142.9, 140.97, 140.95, 139.7, 139.4, 138.4, 137.9, 137.5, 132.3, 131.9, 131.3, 131.1, 129.79, 129.77, 129.6, 129.4, 128.5, 128.2, 127.4, 127.4, 127.3, 127.2, 127.0, 126.9, 126.3, 125.9, 125.7, 123.5, 123.3, 123.2, 123.1, 122.8, 120.8, 120.7, 120.1, 119.93, 119.88, 109.8, 109.6, 108.96, 108.95, 51.9, 51.2, 42.2, 40.6, 22.7, 21.5.

HRMS: (ESI) calcd for $C_{56}H_{46}N_3O_2S^+$[M+H]$^+$ 824.3305; found 824.3295.

(2*S*,3*R*)-5-([1,1'-biphenyl]-4-yl)-3-([1,1'-biphenyl]-4-ylmethyl)-3-methyl-2,4-diphenyl-1-tosyl-1,2,3,6-tetrahydropyridine (96)

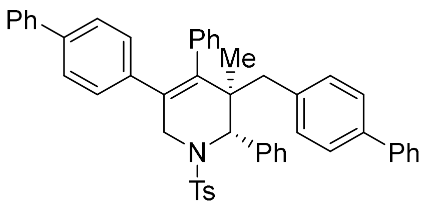

Chemical Formula: $C_{50}H_{43}NO_2S$
Exact Mass: 721.3015

96 was prepared according to general procedure **2.5** using (*R*)-4-methyl-*N*-(2-methyl-1-phenylallyl)-*N*-(3-phenylprop-2-yn-1-yl)benzenesulfonamide (0.1 mmol, 41.6 mg), 4-bromo-1,1'-biphenyl (0.3 mmol, 69.6 mg), and was purified by silica gel column chromatography (PE/EtOAc = 10/1) to obtain **96** as white solid (28.1 mg, 39% yield).

^{1}H NMR (600 MHz, $CDCl_3$) δ 7.69-7.64 (m, 2H), 7.64-7.59 (m, 2H), 7.56-7.47 (m, 4H), 7.48-7.42 (m, 2H), 7.42-7.36 (m, 4H), 7.36-7.29 (m, 2H), 7.22-7.16 (m, 3H), 7.17-7.09 (m, 6H), 7.11-7.01 (m, 3H), 6.90 (d, *J* = 8.1 Hz, 3H), 6.78 (s, 1H), 5.21 (s, 1H), 4.75 (d, *J* = 17.2 Hz, 1H), 3.85 (d, *J* = 17.2 Hz, 1H), 3.53 (d, *J* = 13.1 Hz, 1H), 3.09 (d, *J* = 13.1 Hz, 1H), 2.26 (s, 3H), 0.48 (s, 3H);

^{13}C NMR (151 MHz, $CDCl_3$) δ 142.6, 141.4, 141.0, 140.3, 139.5, 139.2, 138.7, 138.3, 138.2, 136.2, 134.2, 132.1, 131.3, 130.7, 129.2, 129.1, 128.8, 128.7, 128.2, 127.7, 127.5, 127.4, 127.1, 127.0, 126.9, 126.7, 126.6, 126.4, 61.5, 47.0, 44.7, 44.6, 23.0, 21.4.

HRMS: (APCI) calcd for $C_{50}H_{44}NO_2S^+$[M+H]$^+$ 722.3087; found 722.3103.

2.6 NMR Spectra

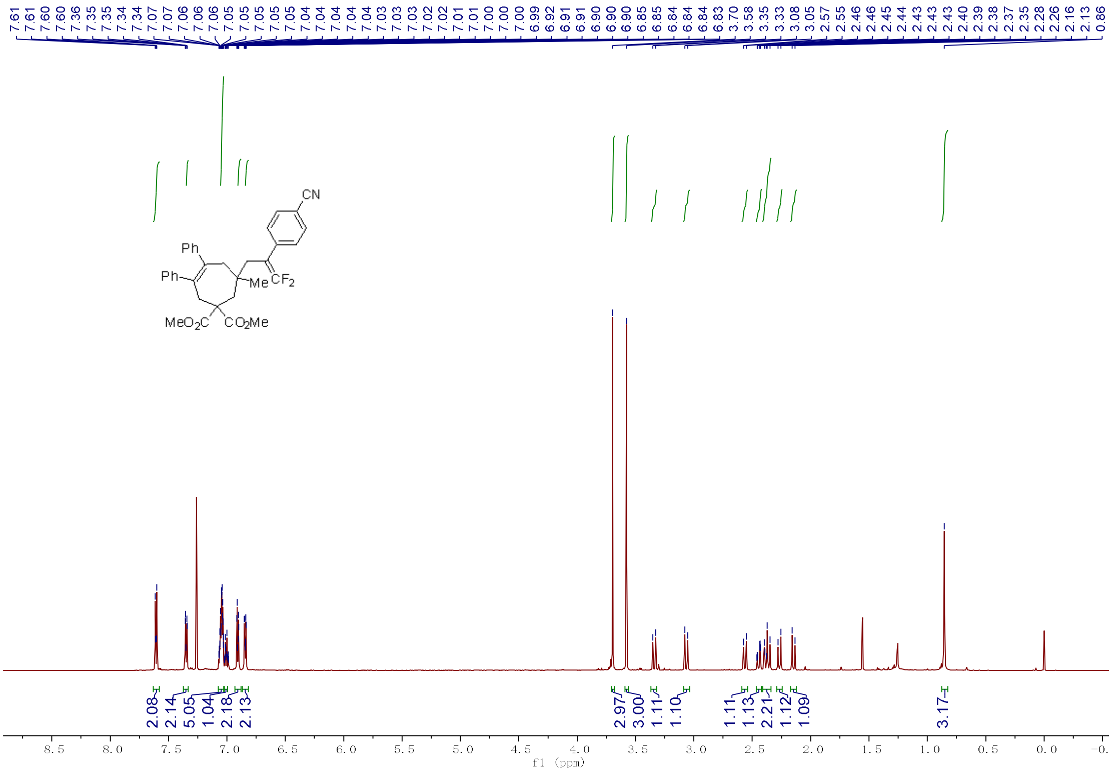

Supplementary Figure 18. ^{1}H NMR of compound **4**

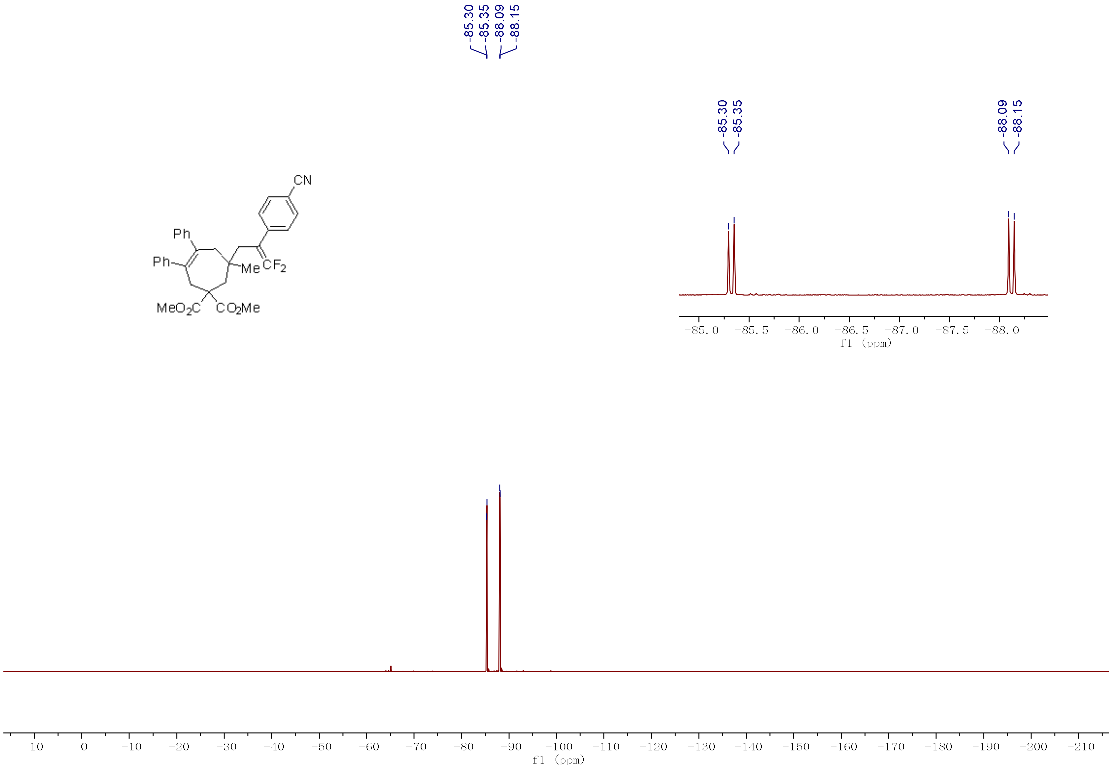

Supplementary Figure 19. ^{19}F NMR of compound **4**

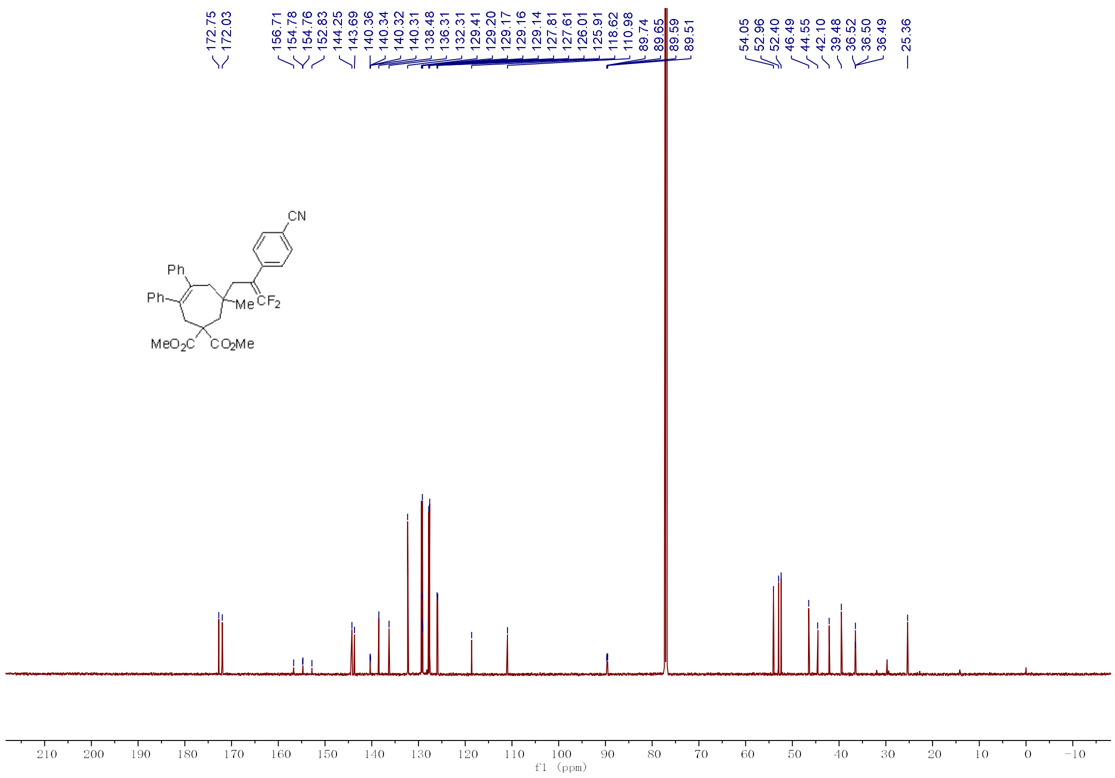

Supplementary Figure 20. ^{13}C NMR of compound **4**

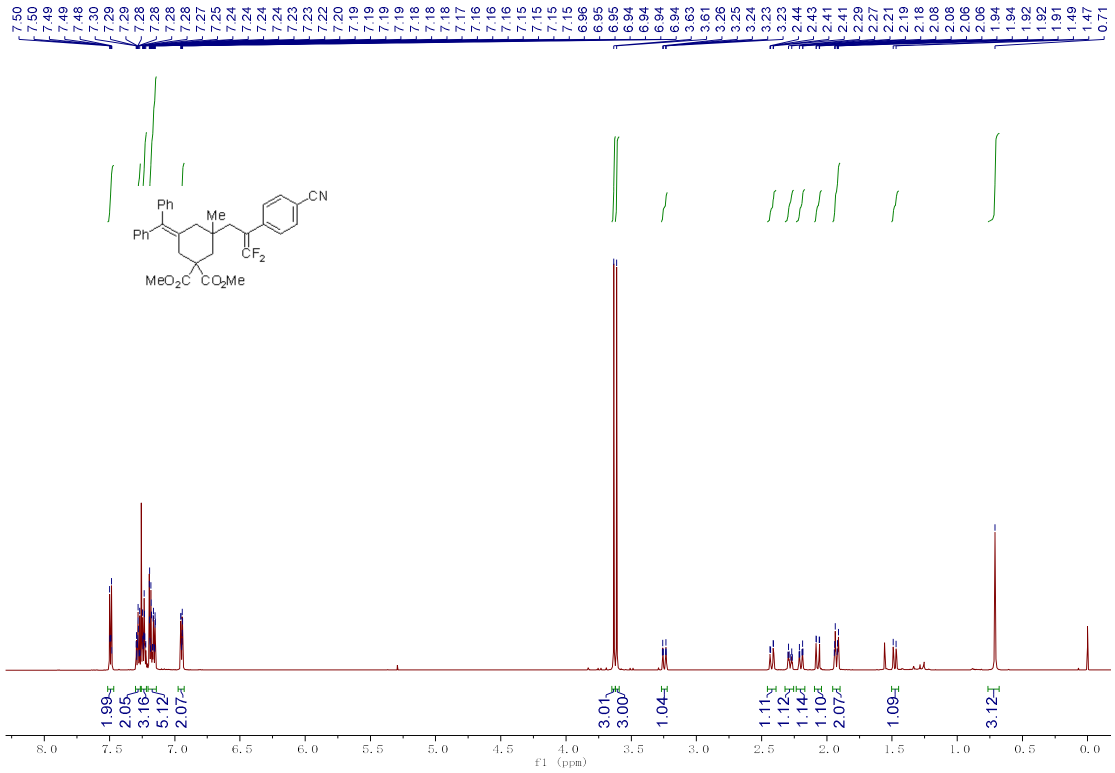

Supplementary Figure 21. ^{1}H NMR of compound **5**

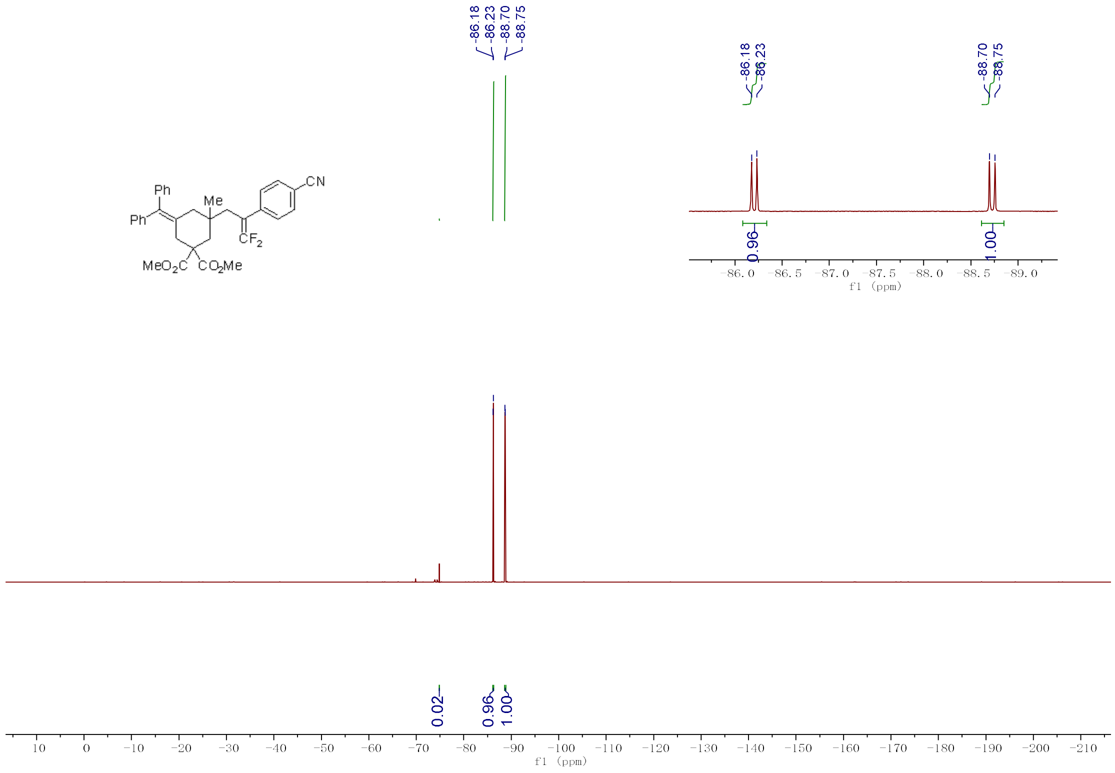

Supplementary Figure 22. ^{19}F NMR of compound **5**

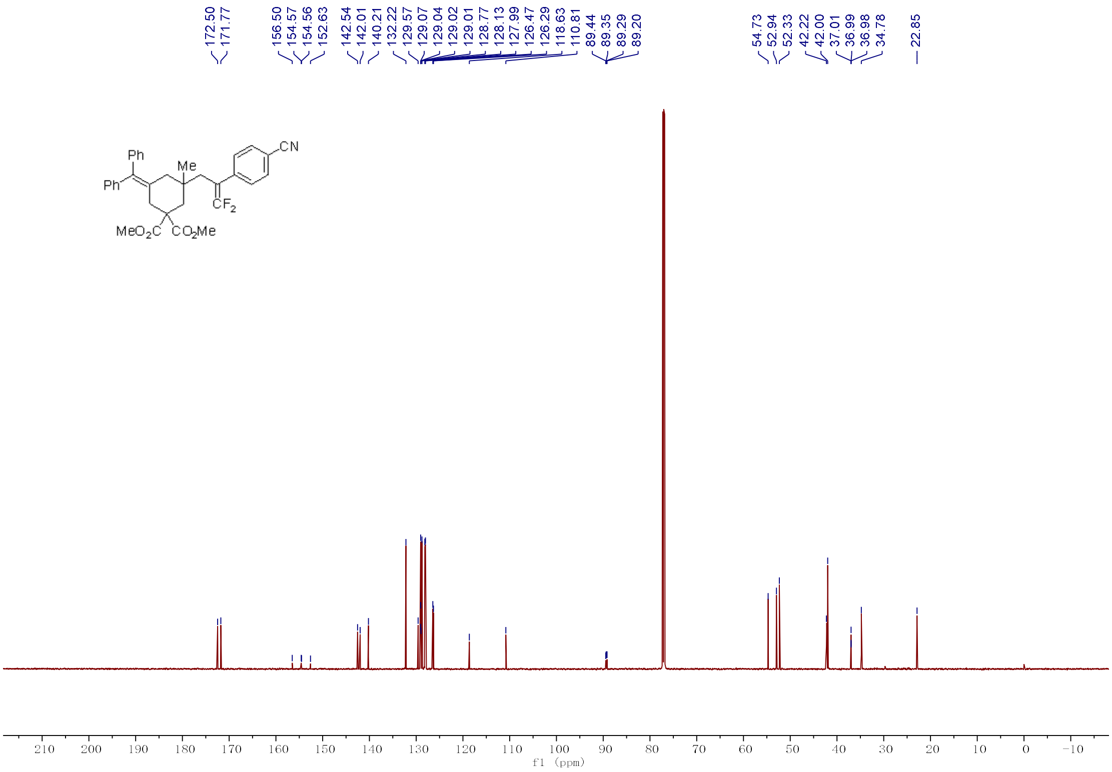

Supplementary Figure 23. ^{13}C NMR of compound **5**

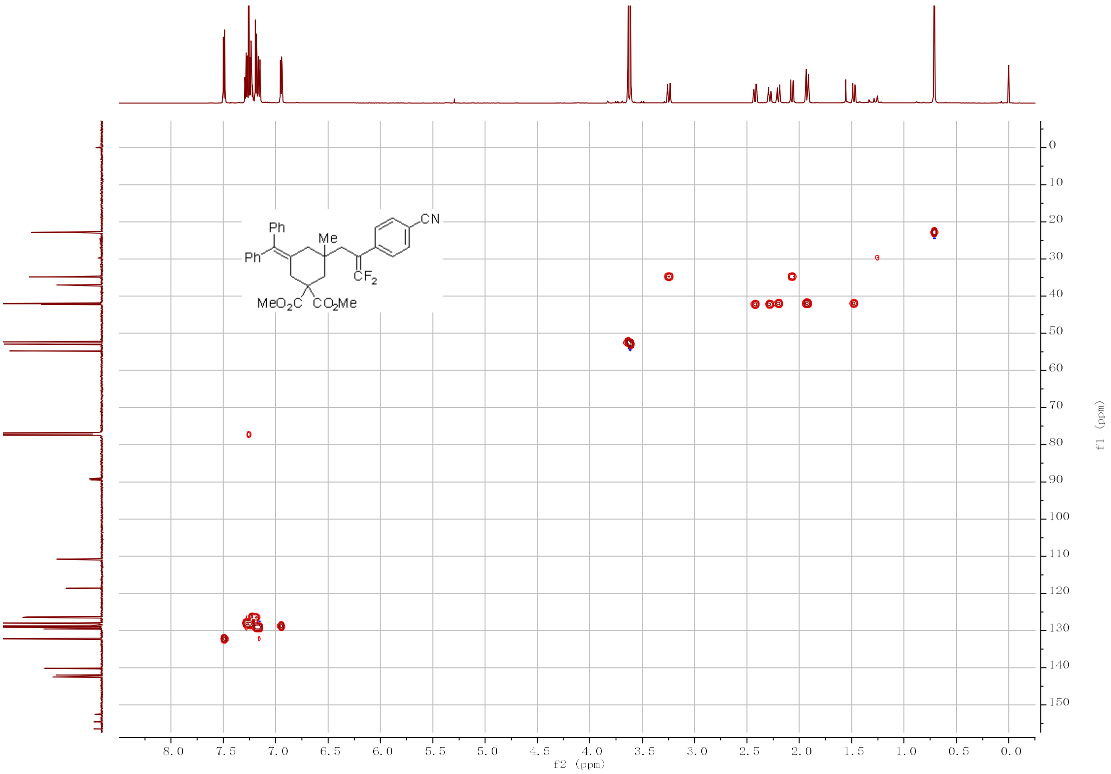

Supplementary Figure 24. HSQC of compound **5**

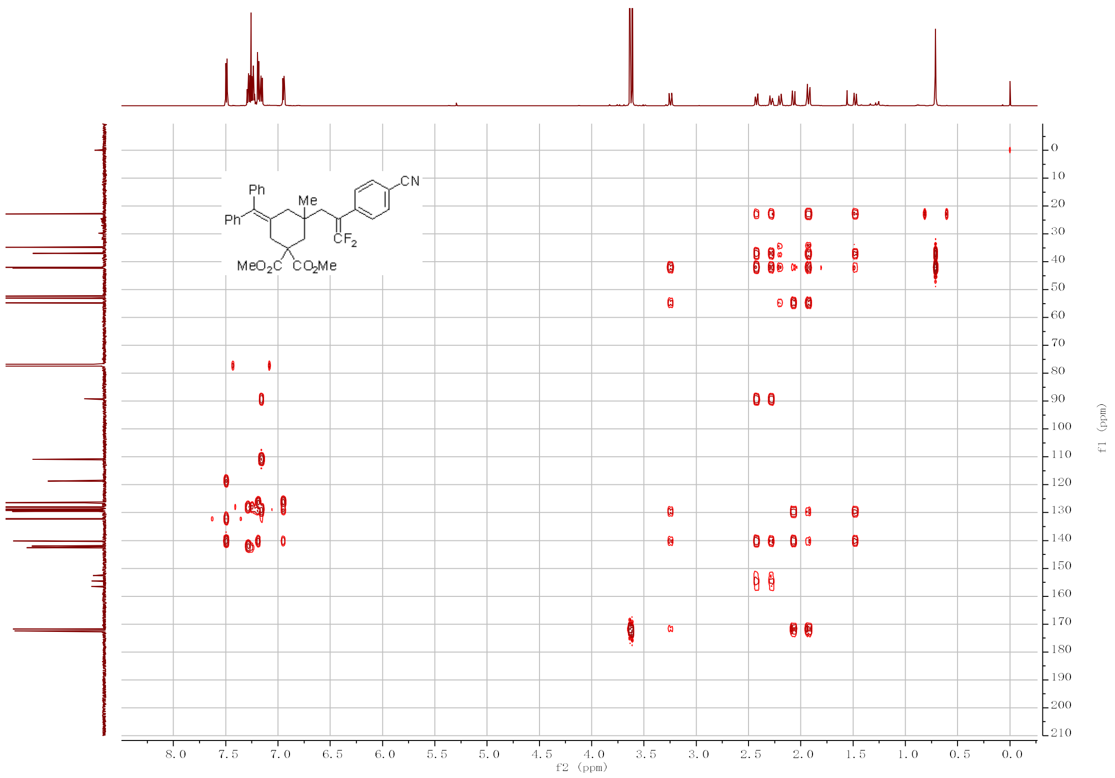

Supplementary figure 25. HMBC of compound **5**

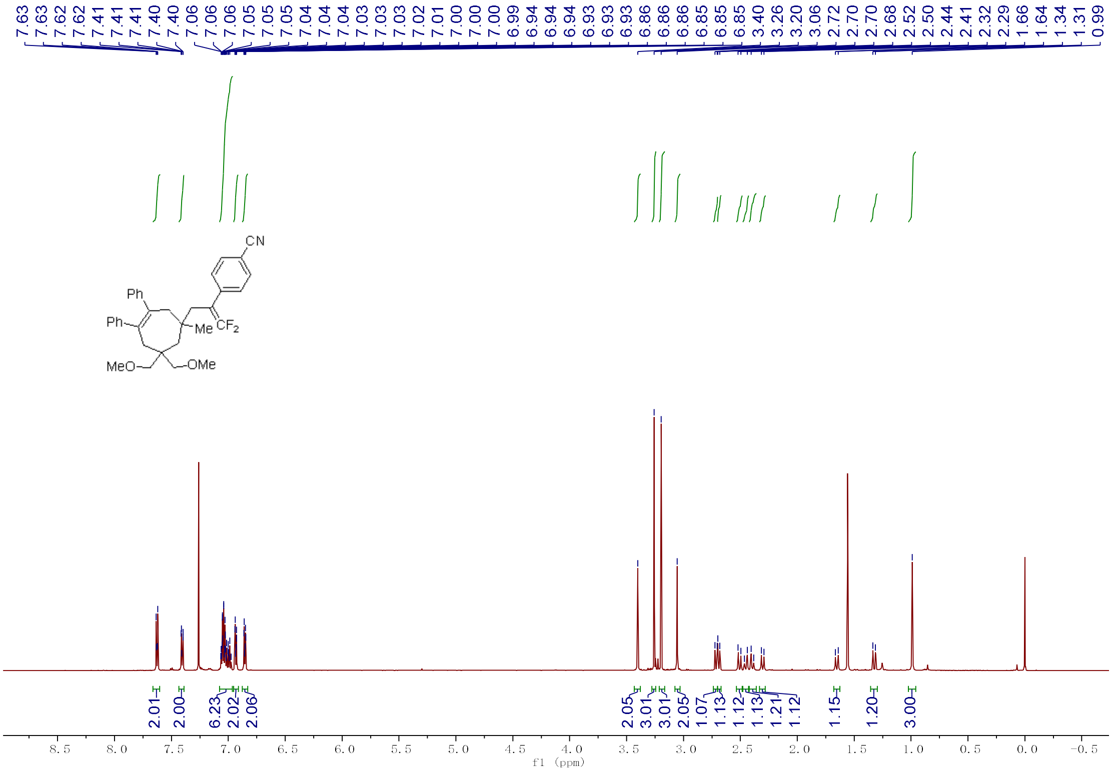

Supplementary Figure 26. ^{1}H NMR of compound **6**

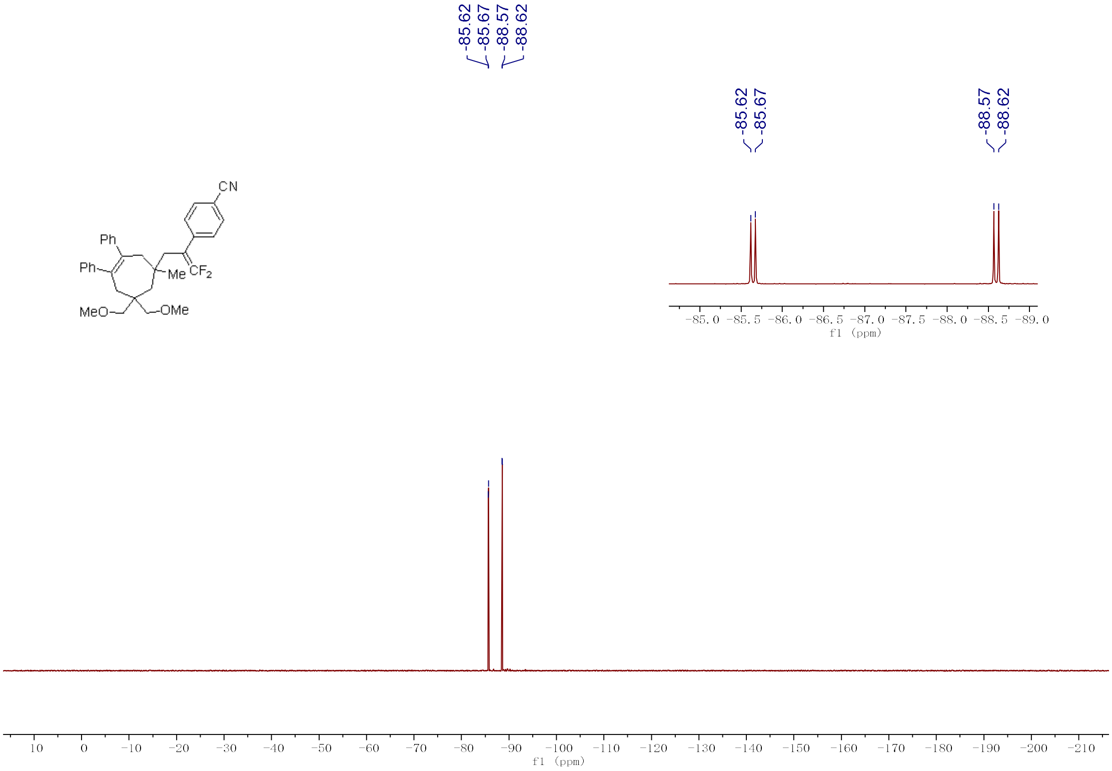

Supplementary Figure 27. ^{19}F NMR of compound **6**

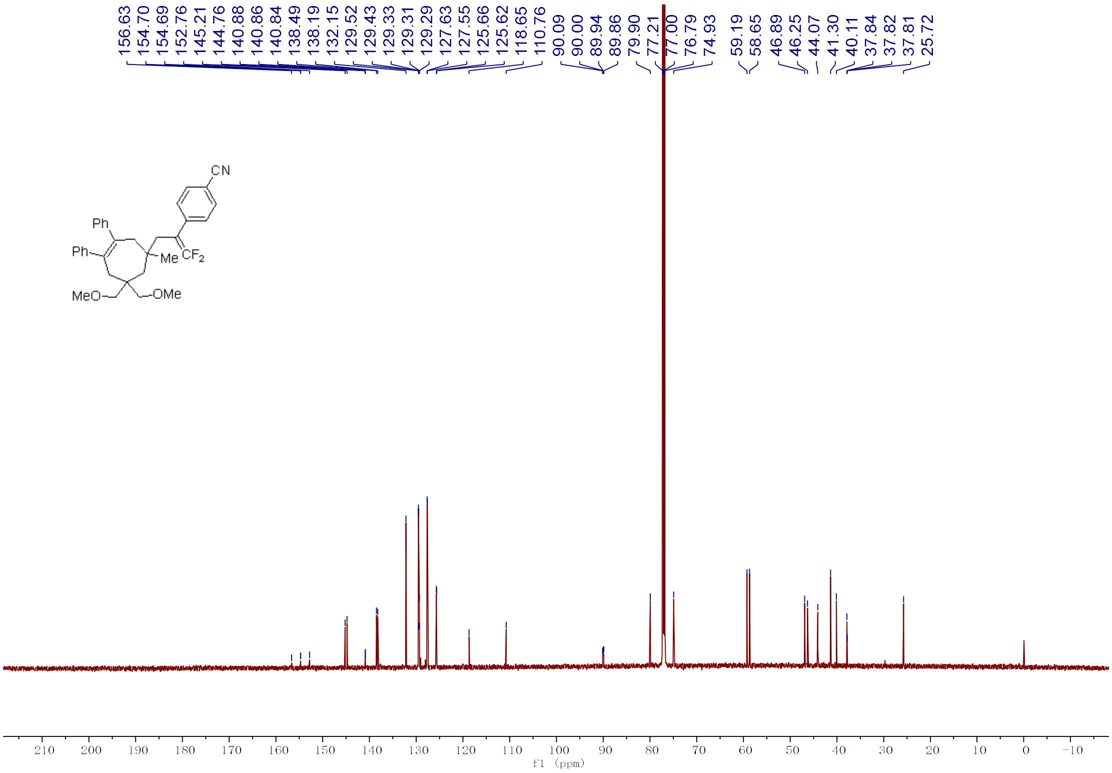

Supplementary figure 28. ^{13}C NMR of compound **6**

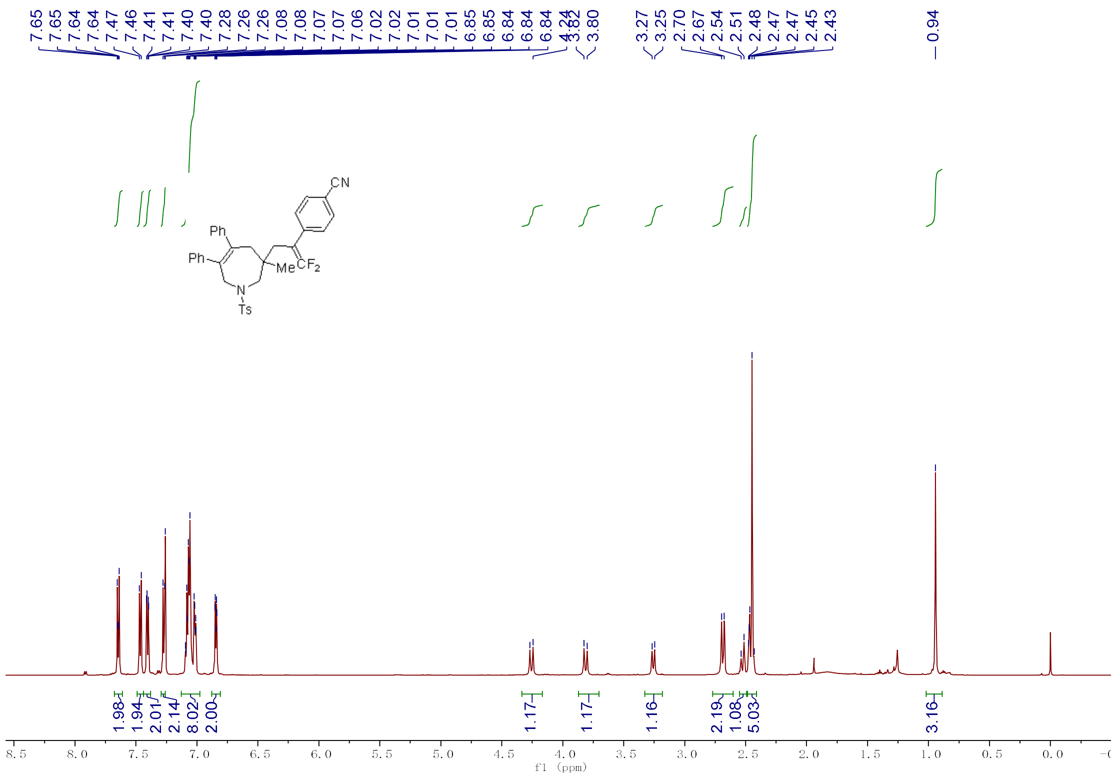

Supplementary Figure 29. ^{1}H NMR of compound **7**

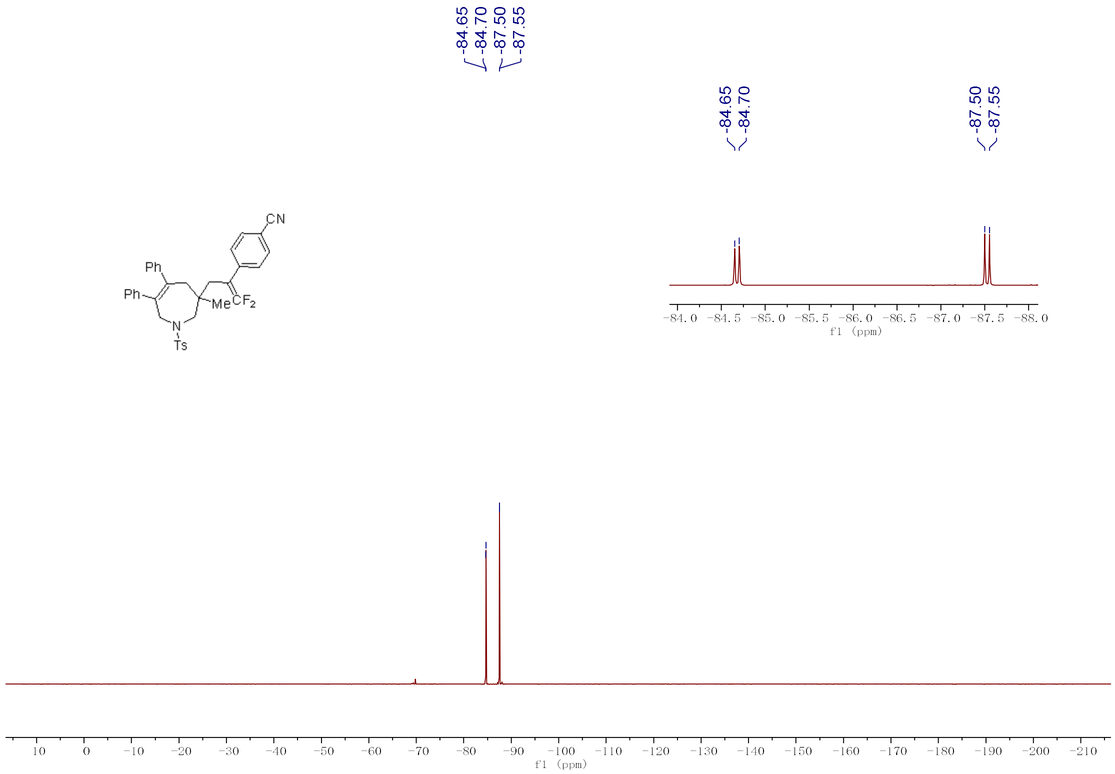

Supplementary Figure 30. ^{19}F NMR of compound **7**

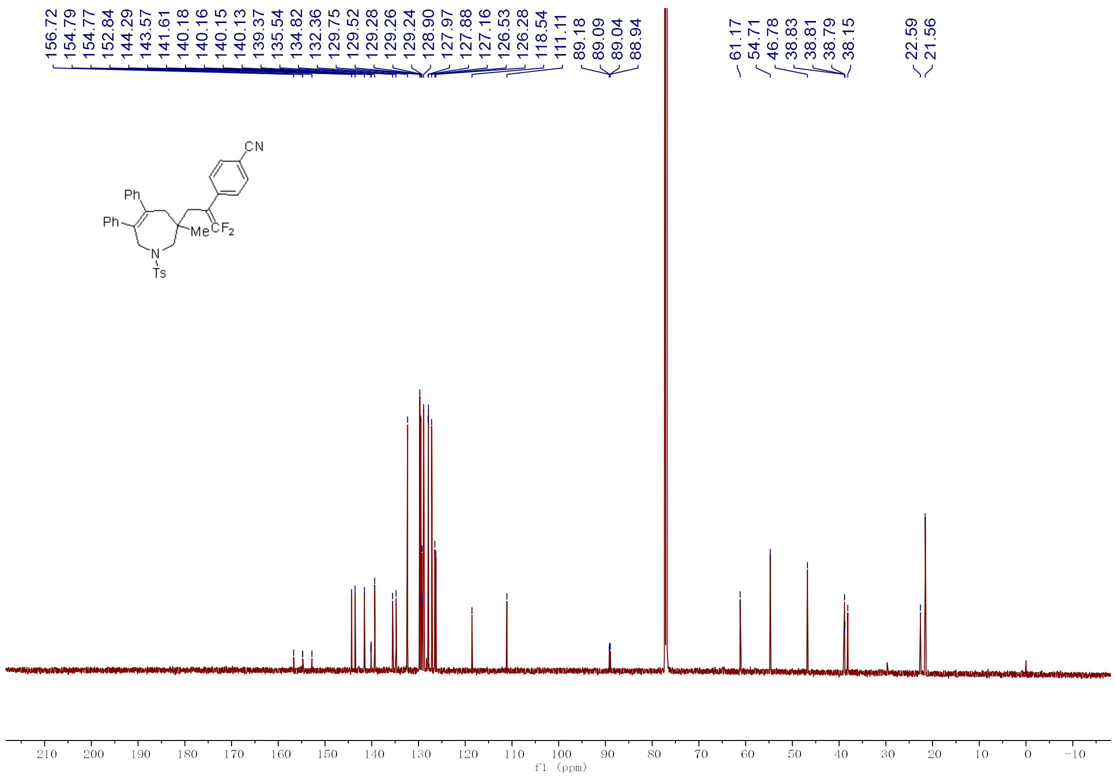

Supplementary figure 31. ^{13}C NMR of compound **7**

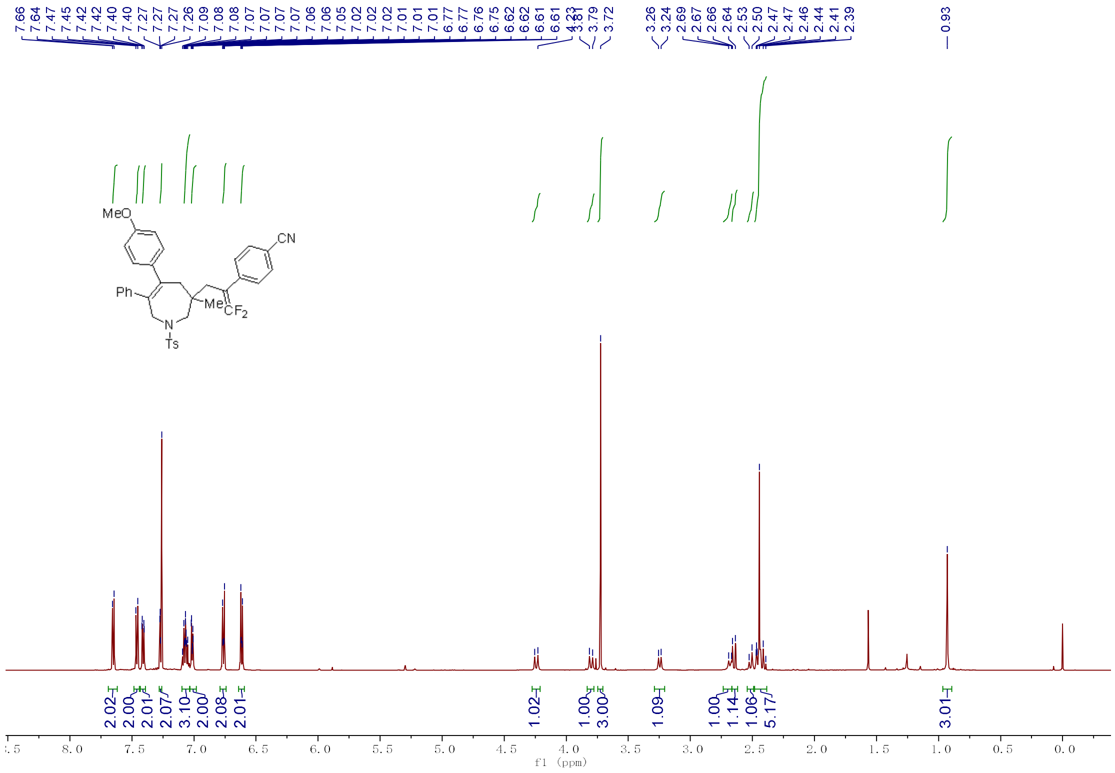

Supplementary figure 32. ^{1}H NMR of compound **8**

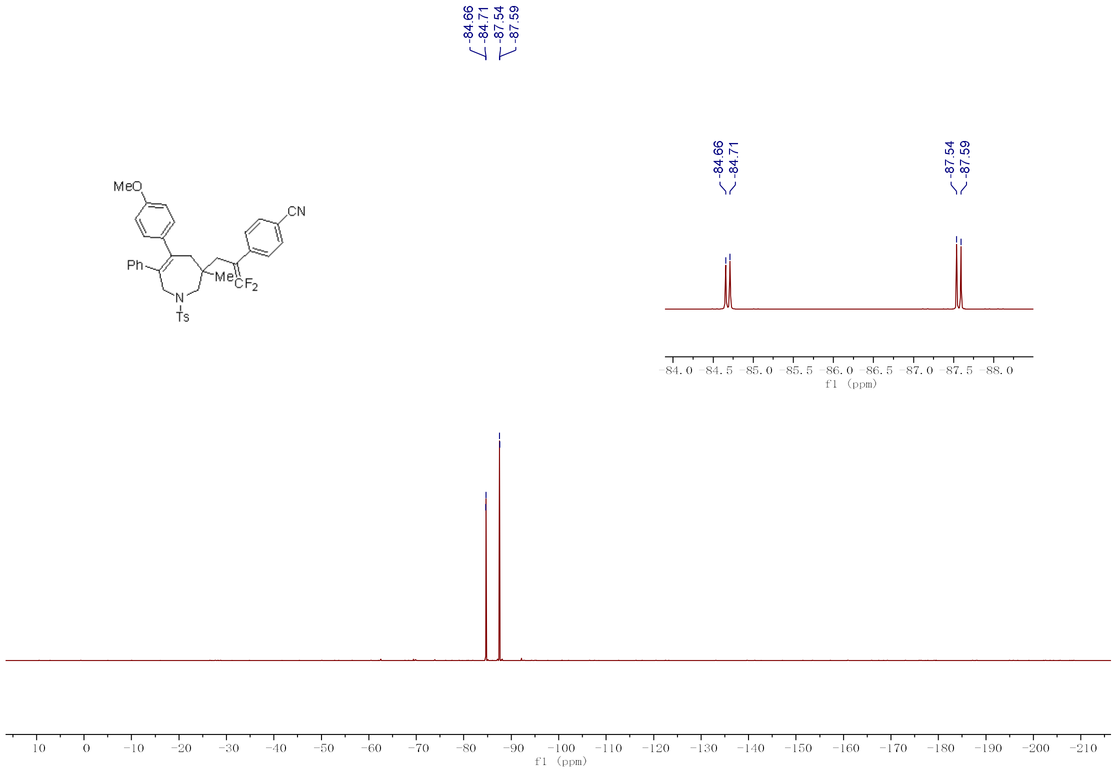

Supplementary figure 33. ^{19}F NMR of compound **8**

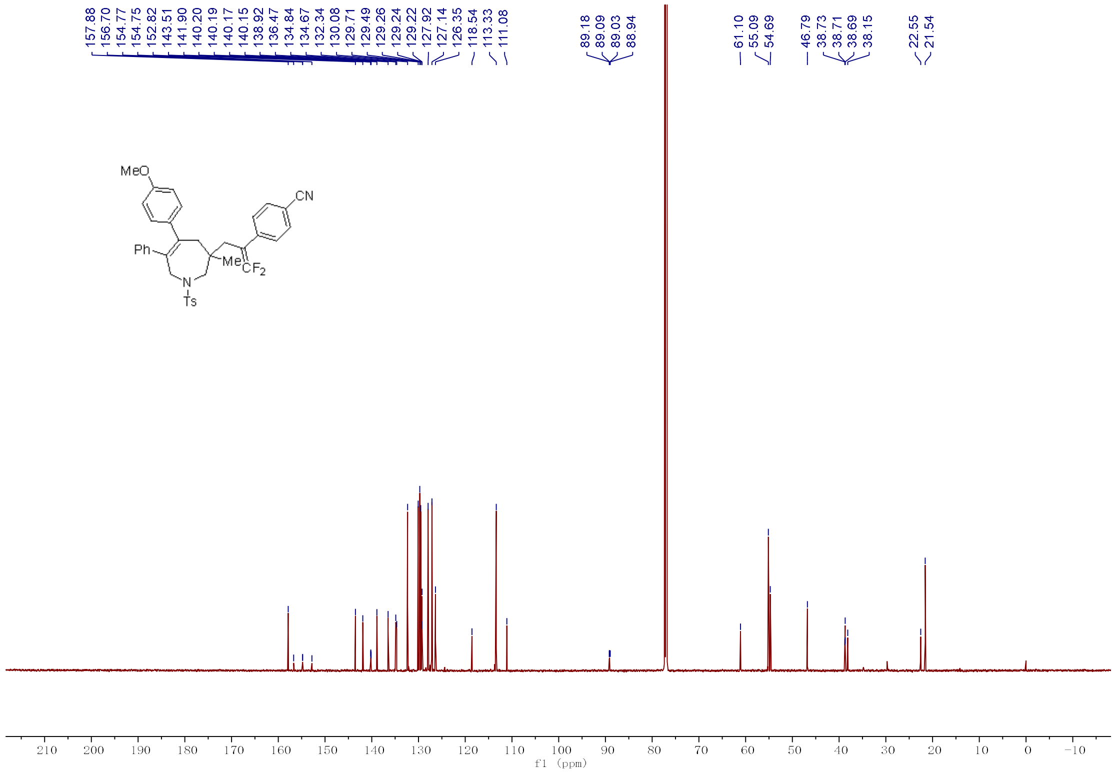

Supplementary figure 34. ^{13}C NMR of compound **8**

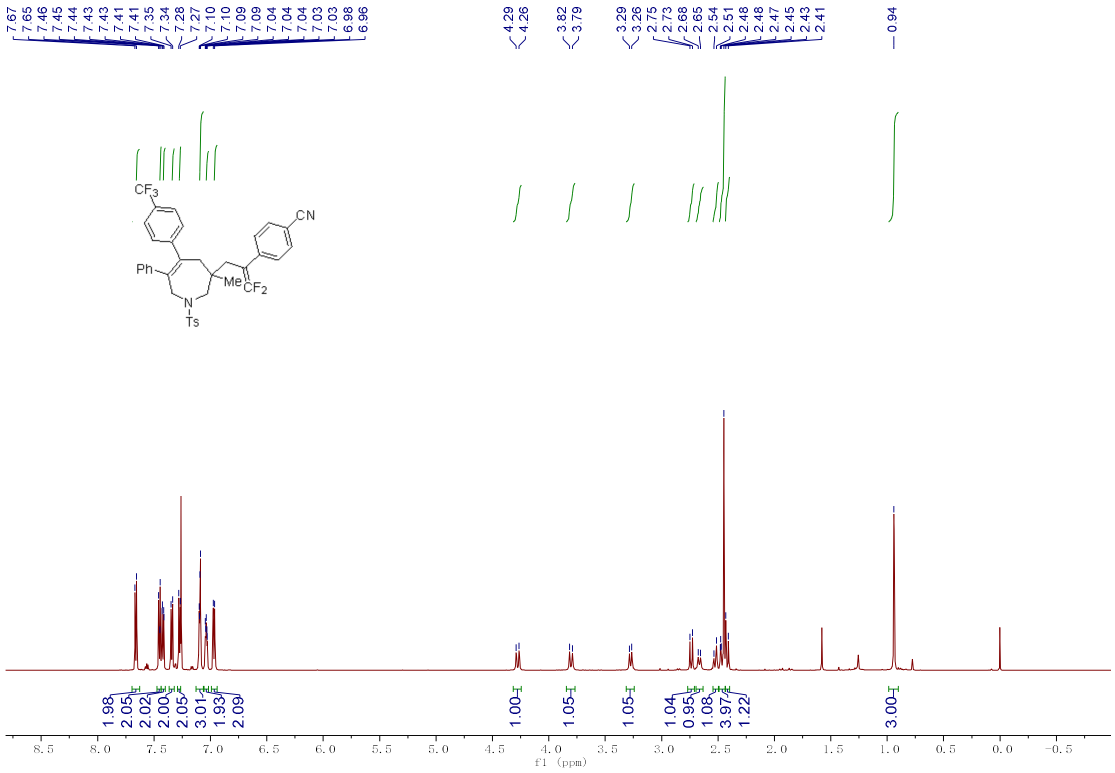

Supplementary figure 35. ^{1}H NMR of compound **9**

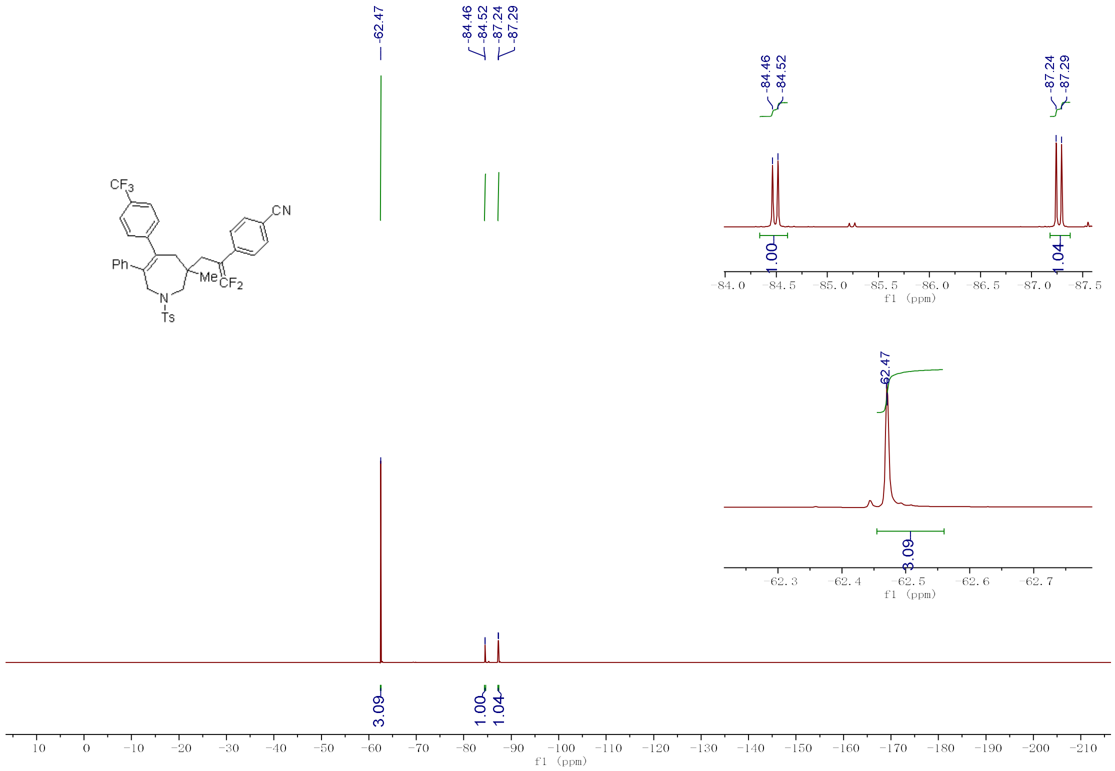

Supplementary figure 36. ^{19}F NMR of compound **9**

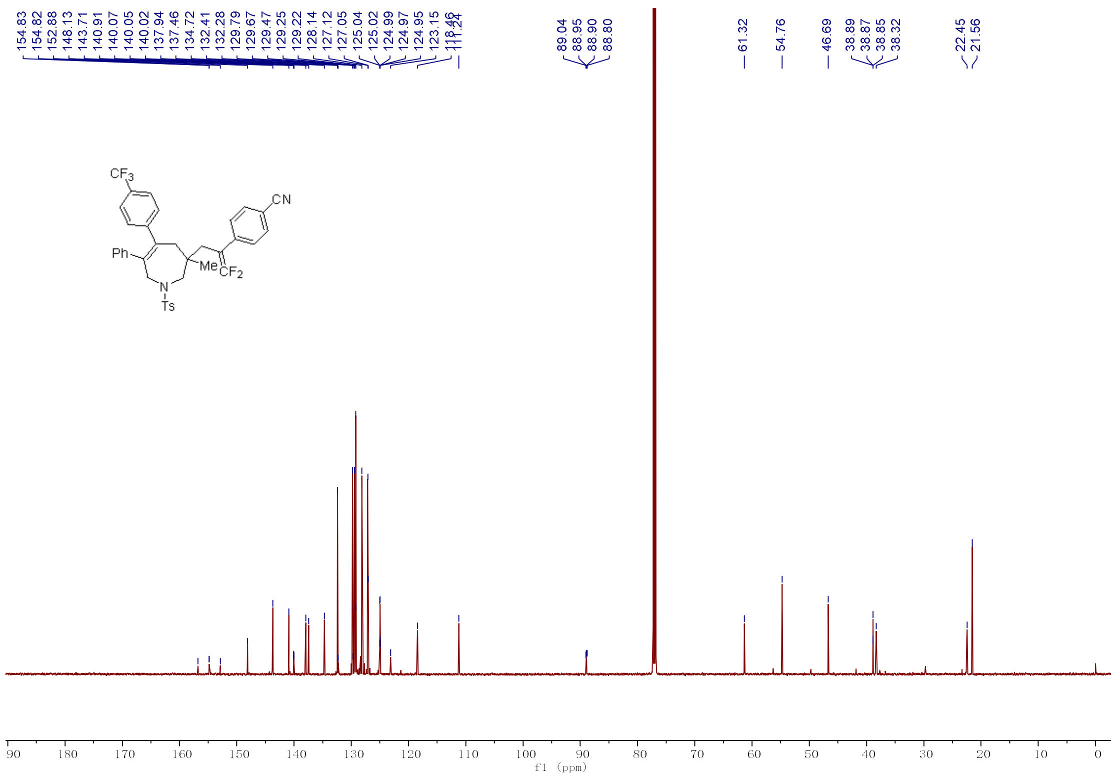

Supplementary figure 37. ^{13}C NMR of compound **9**

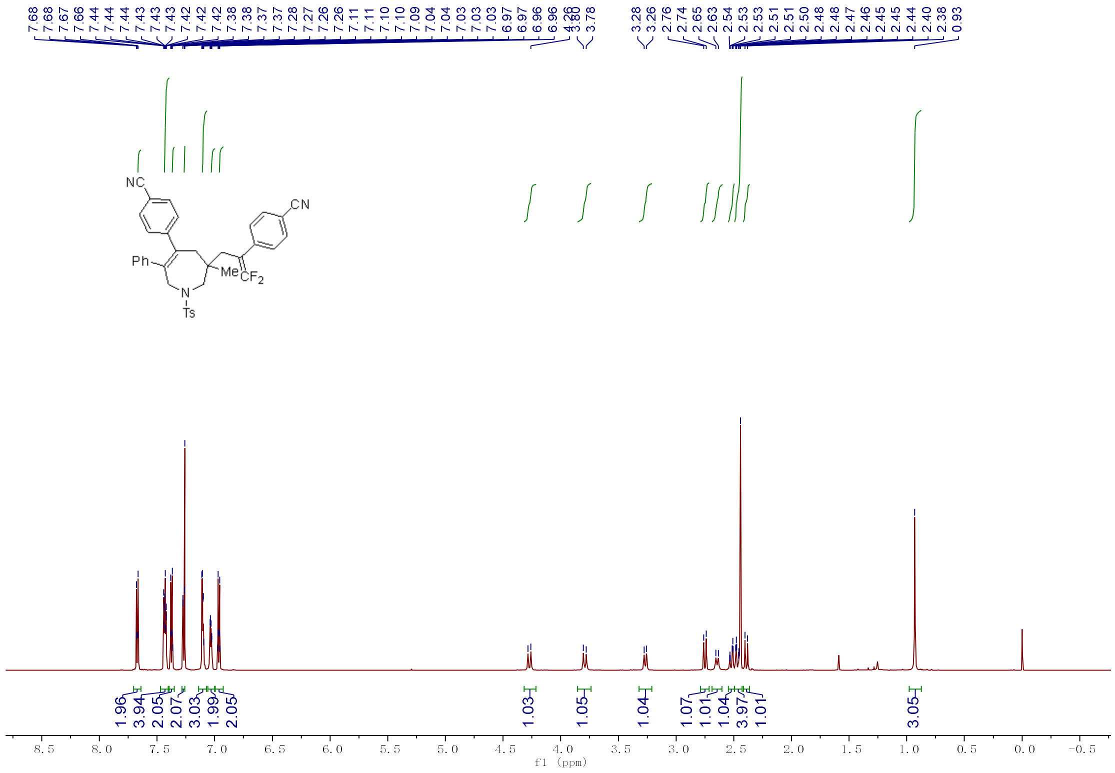

Supplementary figure 38. ^{1}H NMR of compound **10**

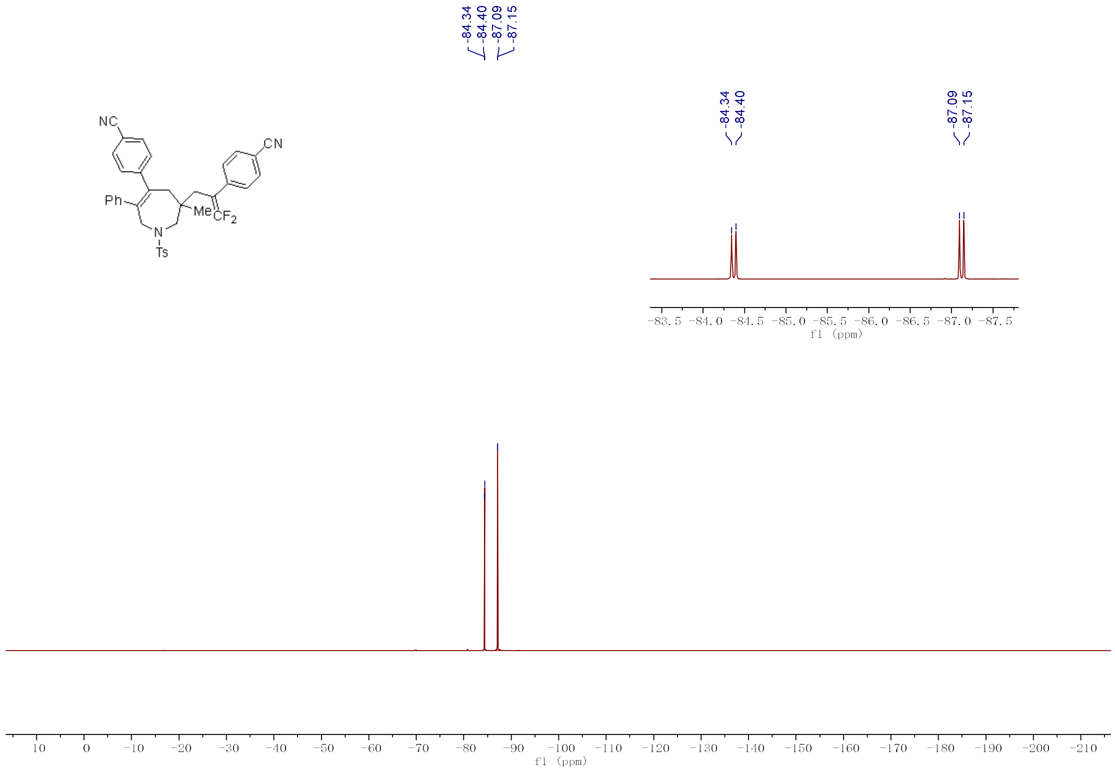

Supplementary figure 39. ^{19}F NMR of compound **9**

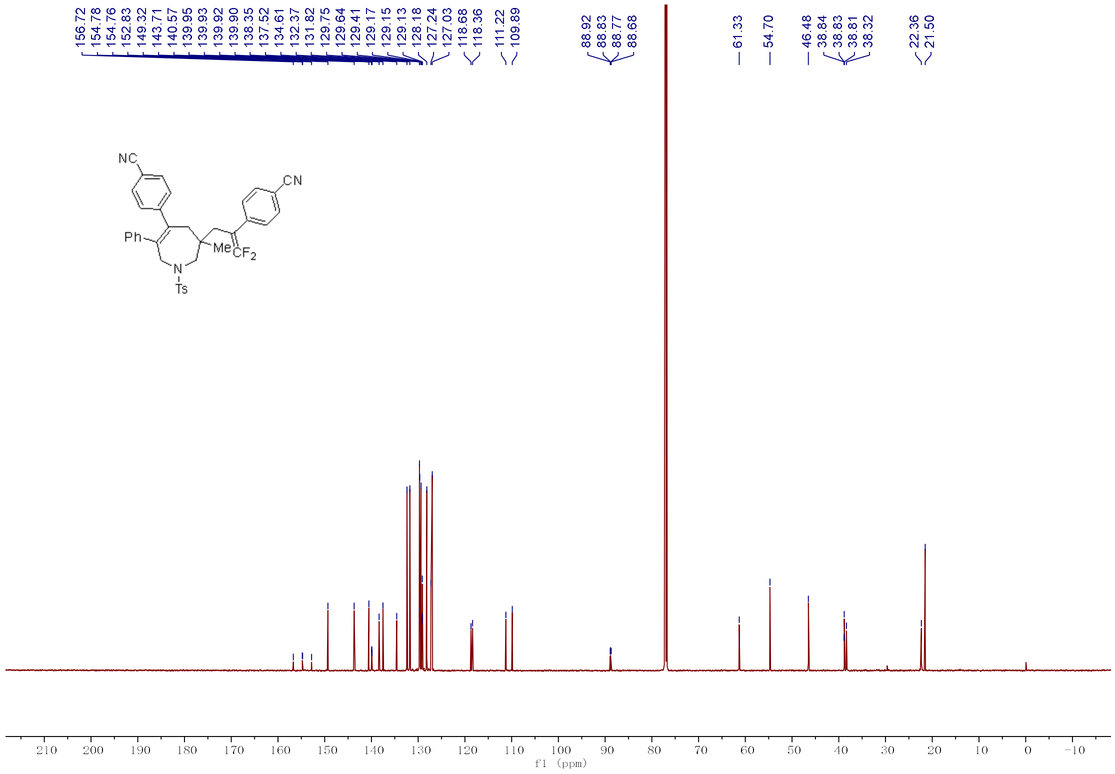

Supplementary figure 40. ^{13}C NMR of compound **10**

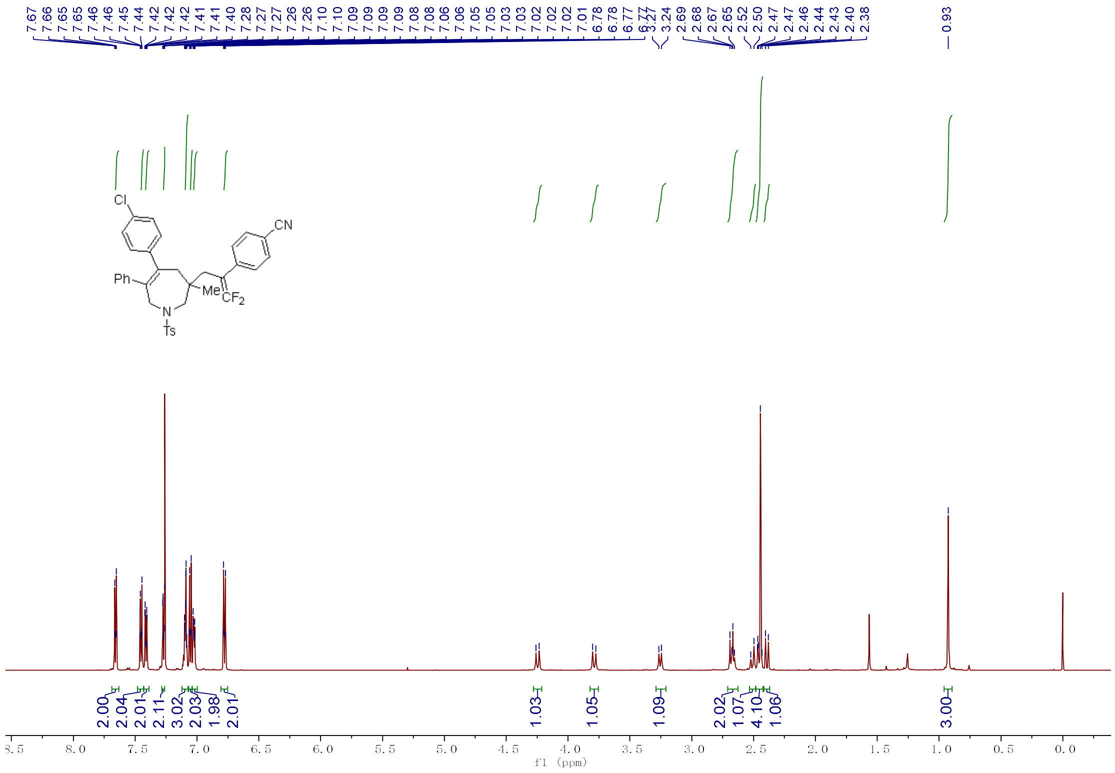

Supplementary figure 41. ^{1}H NMR of compound **11**

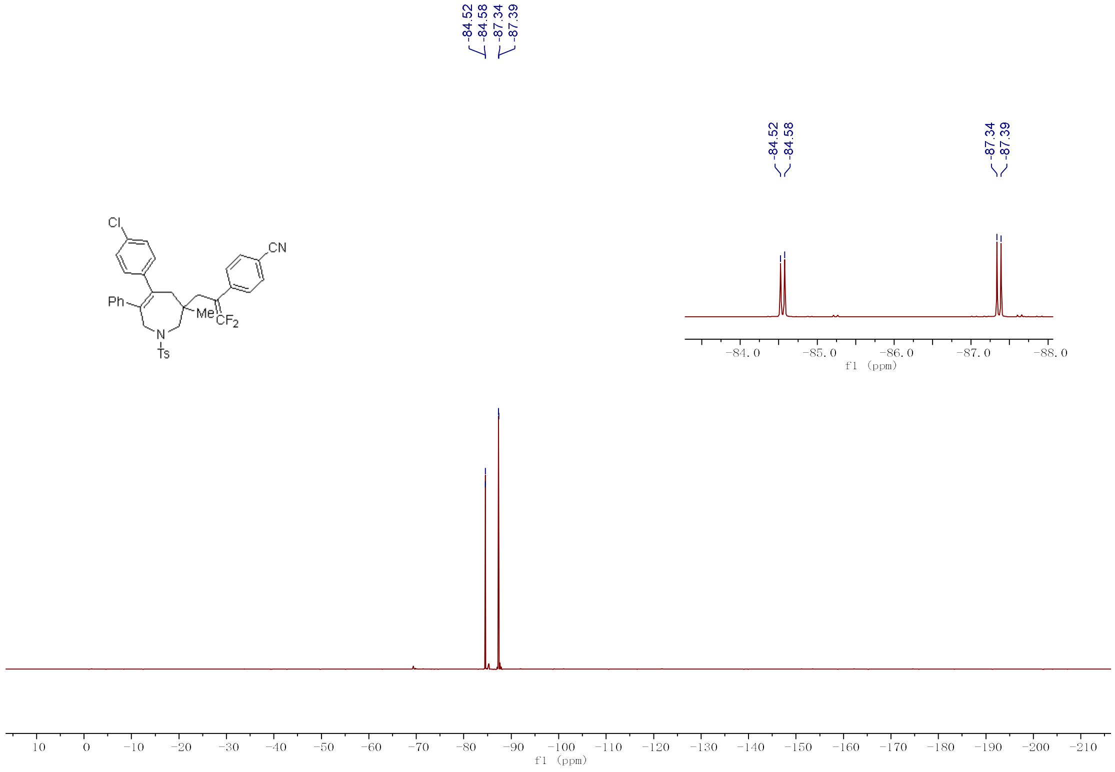

Supplementary figure 42. ^{19}F NMR of compound **11**

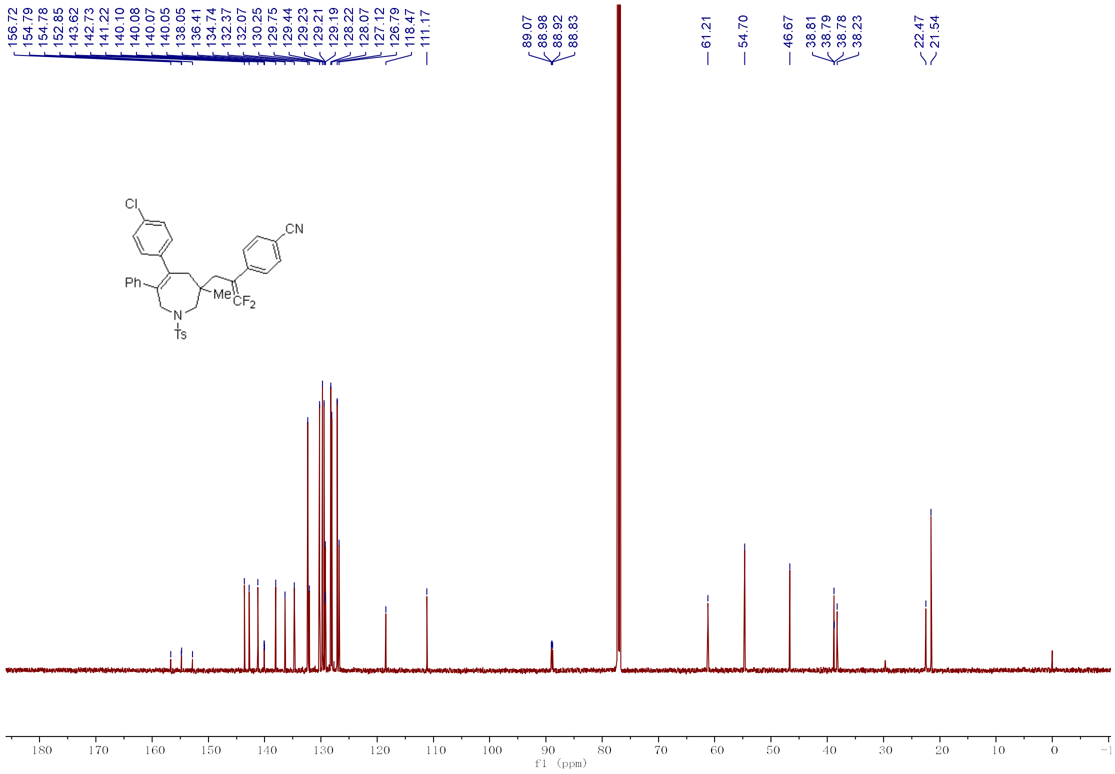

Supplementary figure 43. ^{13}C NMR of compound **11**

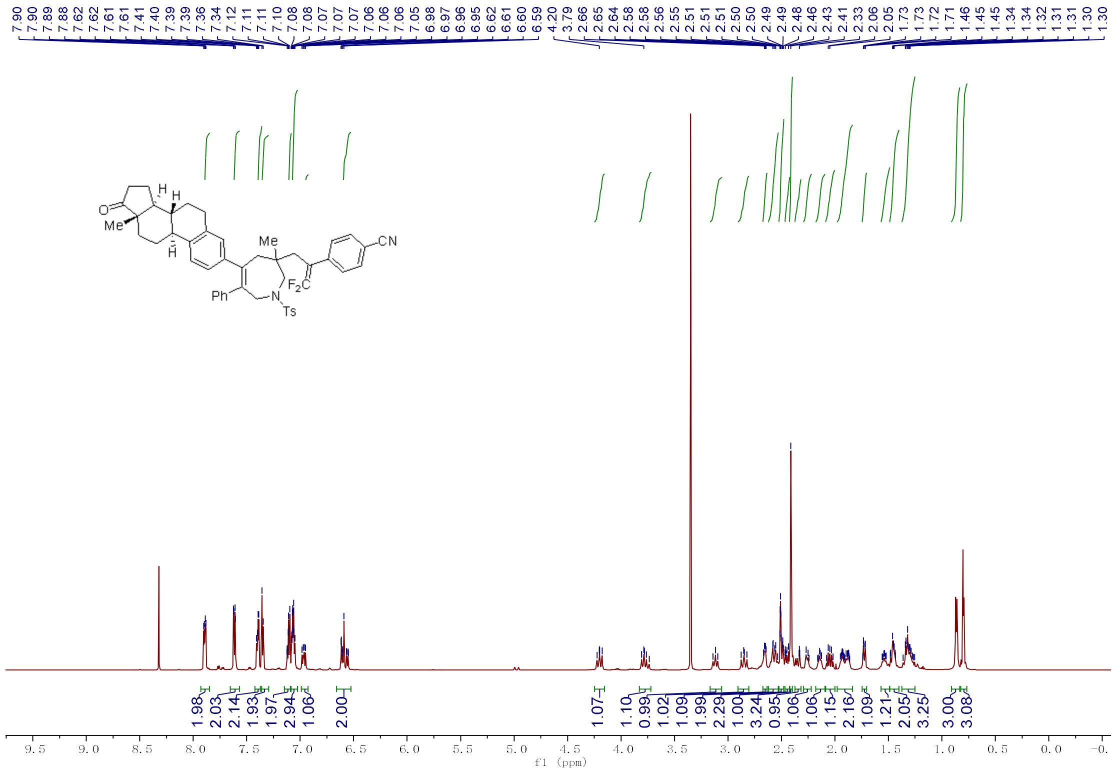

Supplementary figure 44. ^{1}H NMR of compound **12**

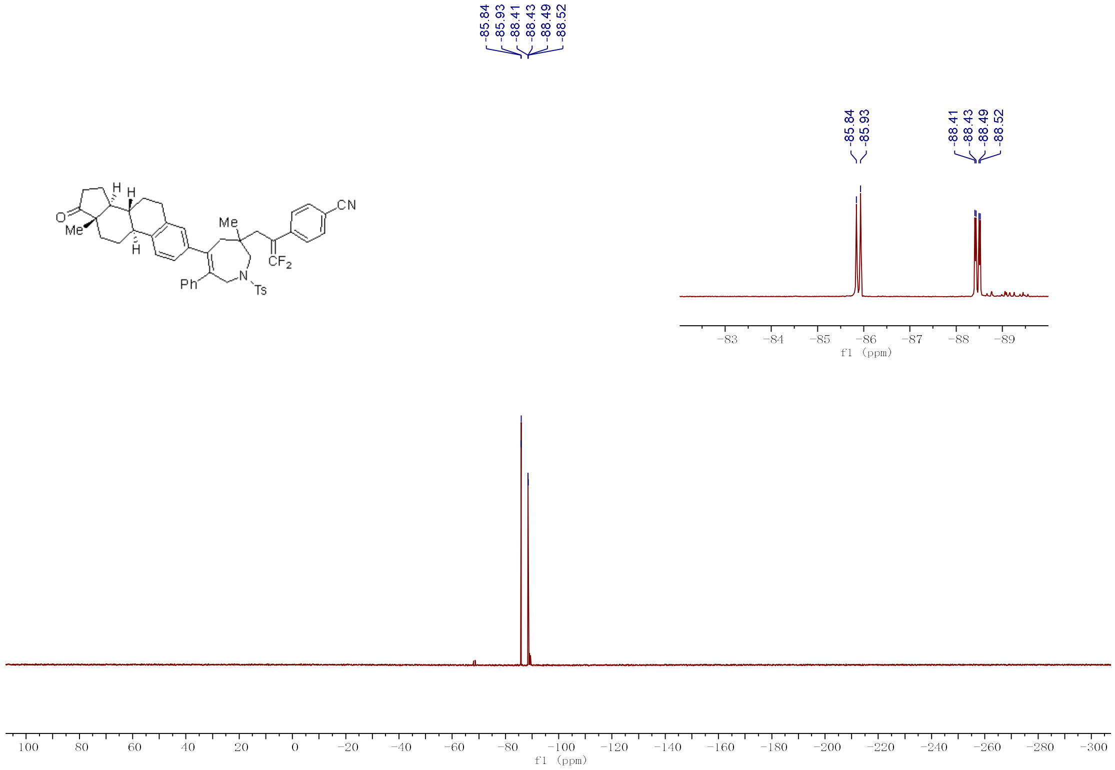

Supplementary figure 45. ^{19}F NMR of compound **12**

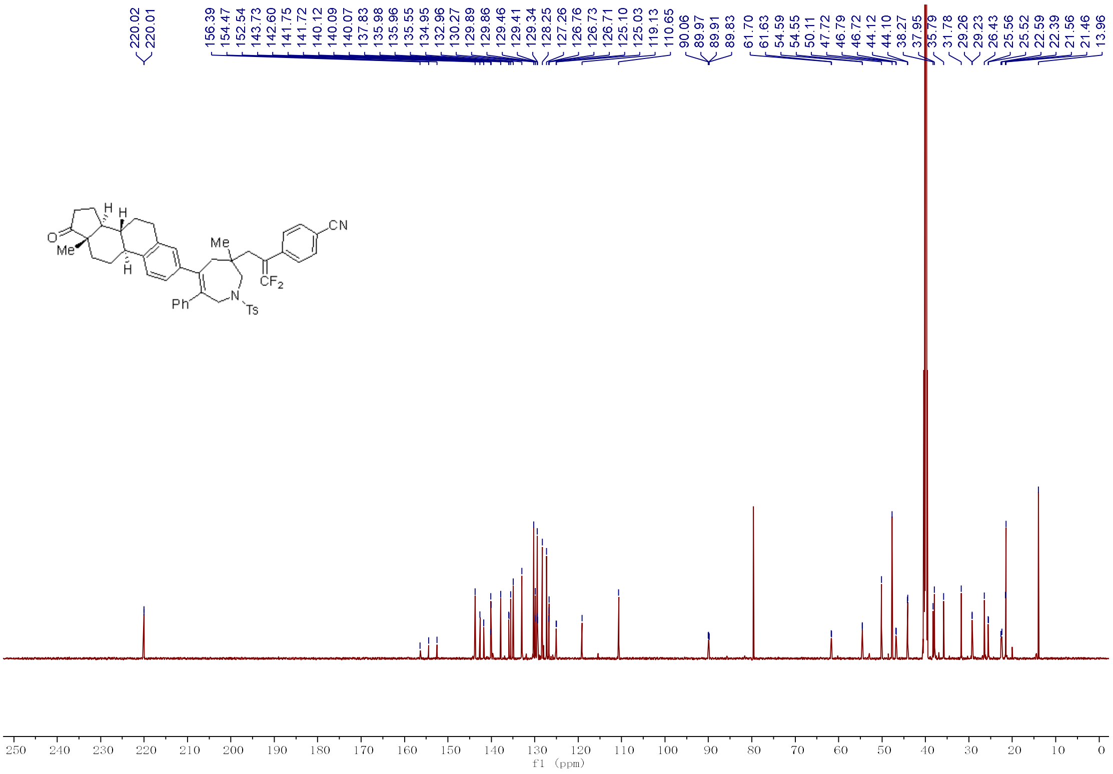

Supplementary figure 46. ^{13}C NMR of compound **12**

13

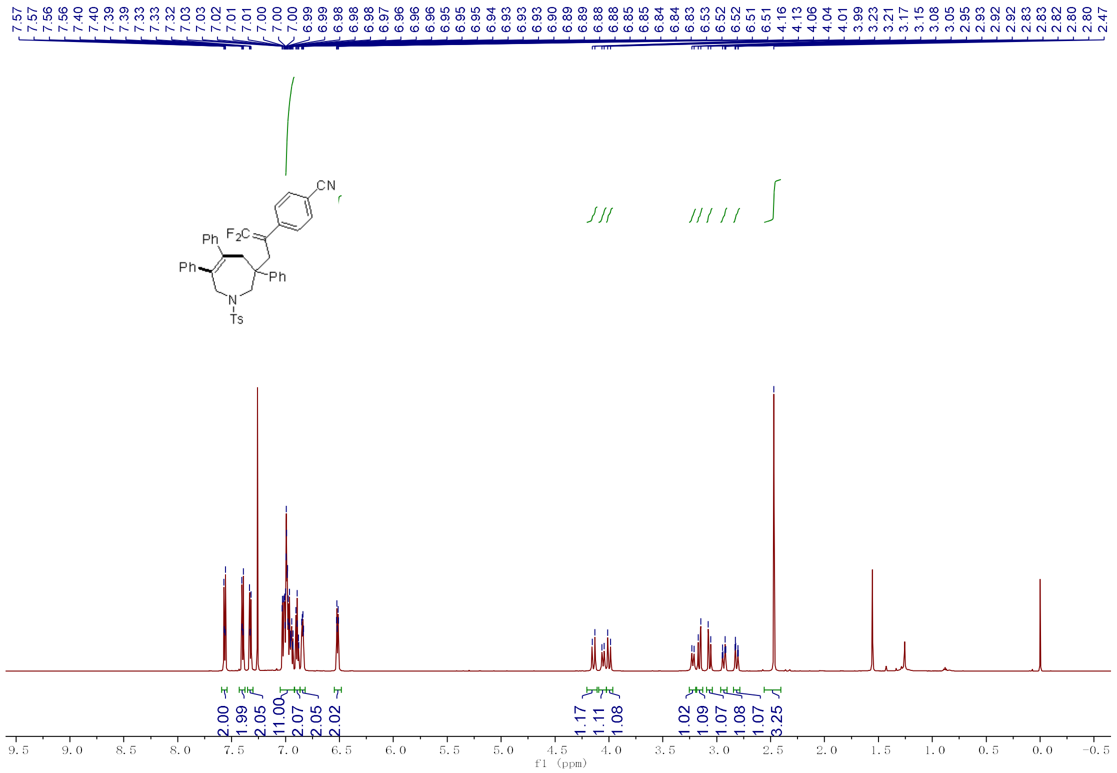

Supplementary figure 47. ^{1}H NMR of compound **13**

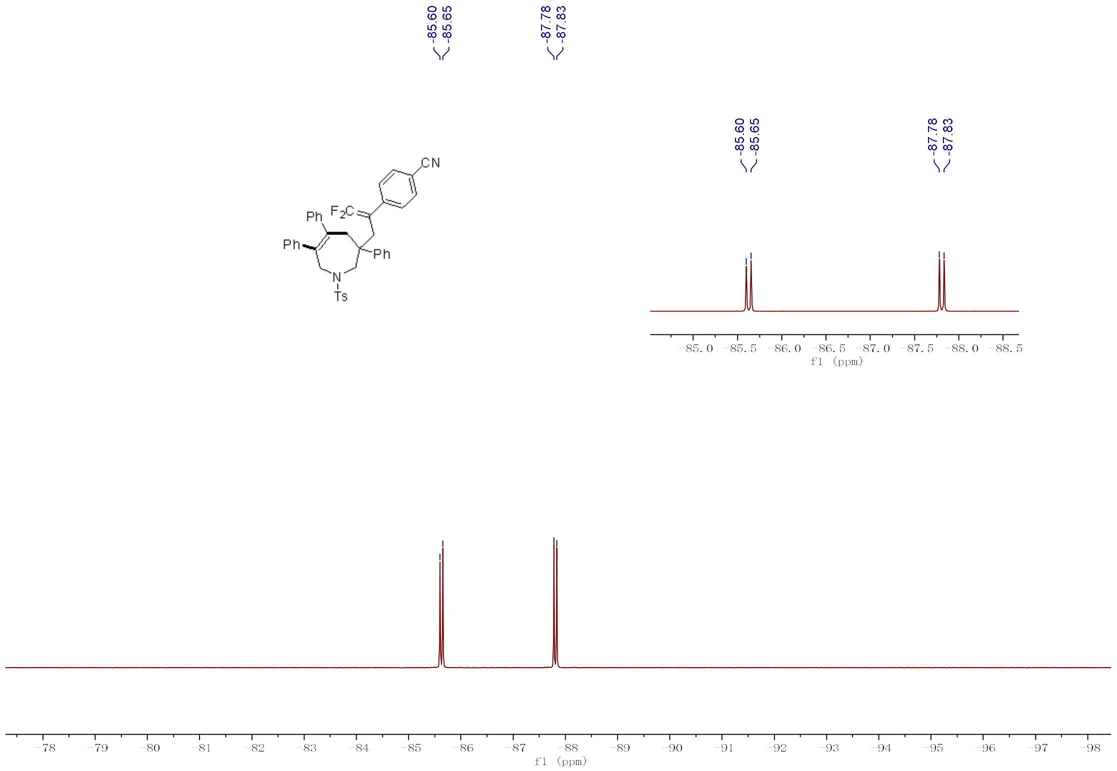

Supplementary figure 48. ^{19}F NMR of compound **13**

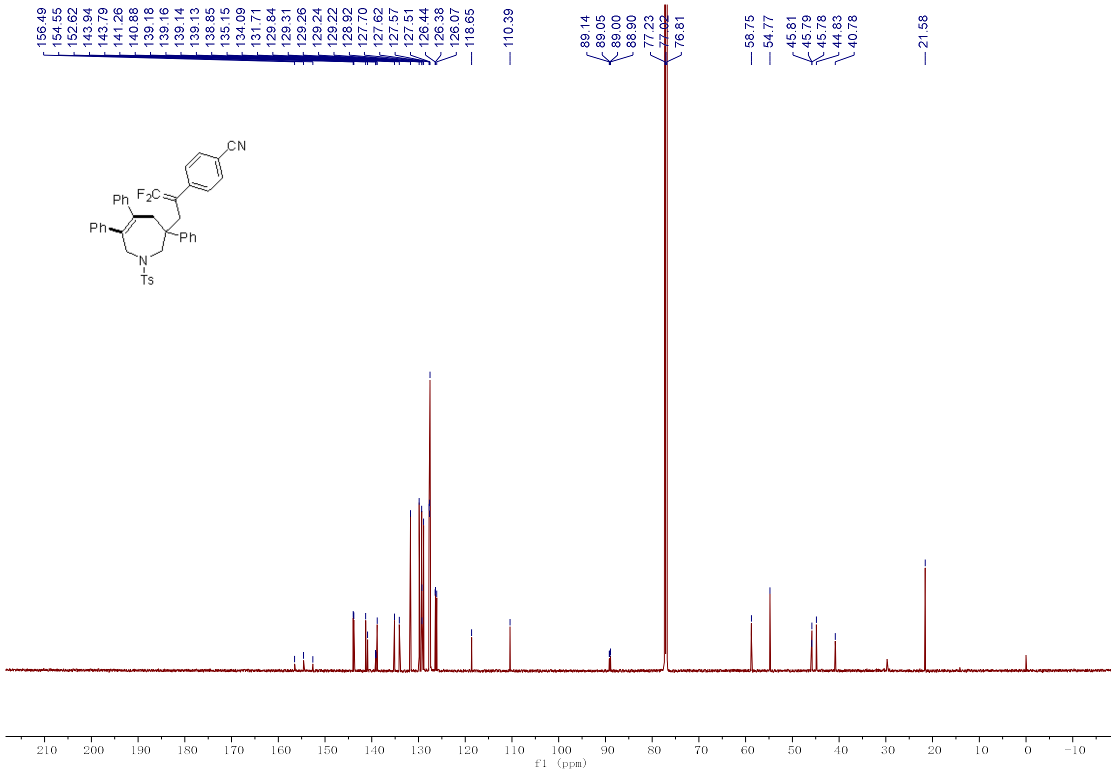

Supplementary figure 49. ^{13}C NMR of compound **13**

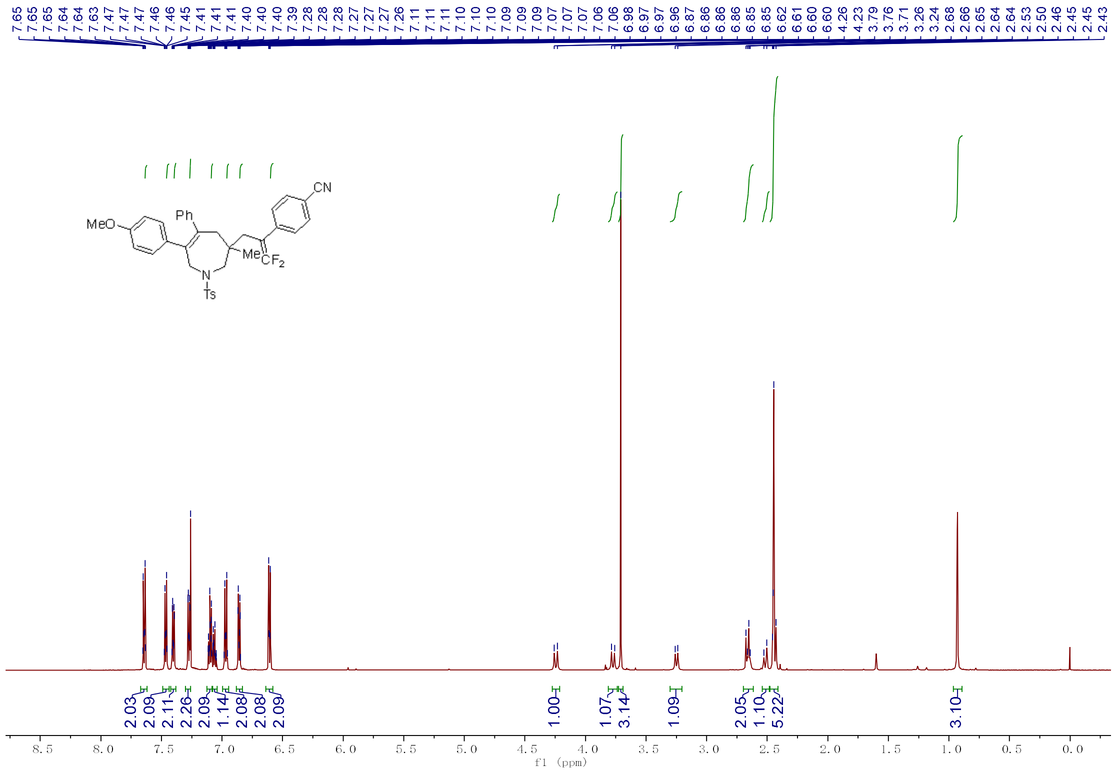

Supplementary figure 50. ^{1}H NMR of compound **14**

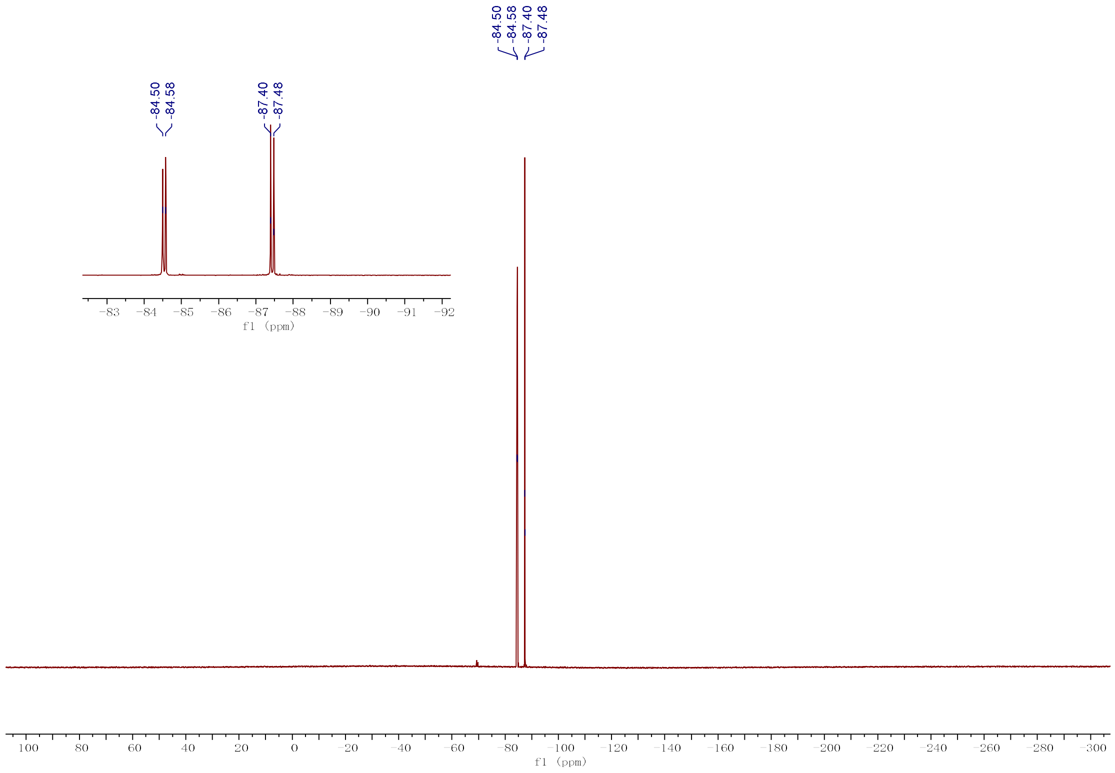

Supplementary figure 51. ^{19}F NMR of compound **14**

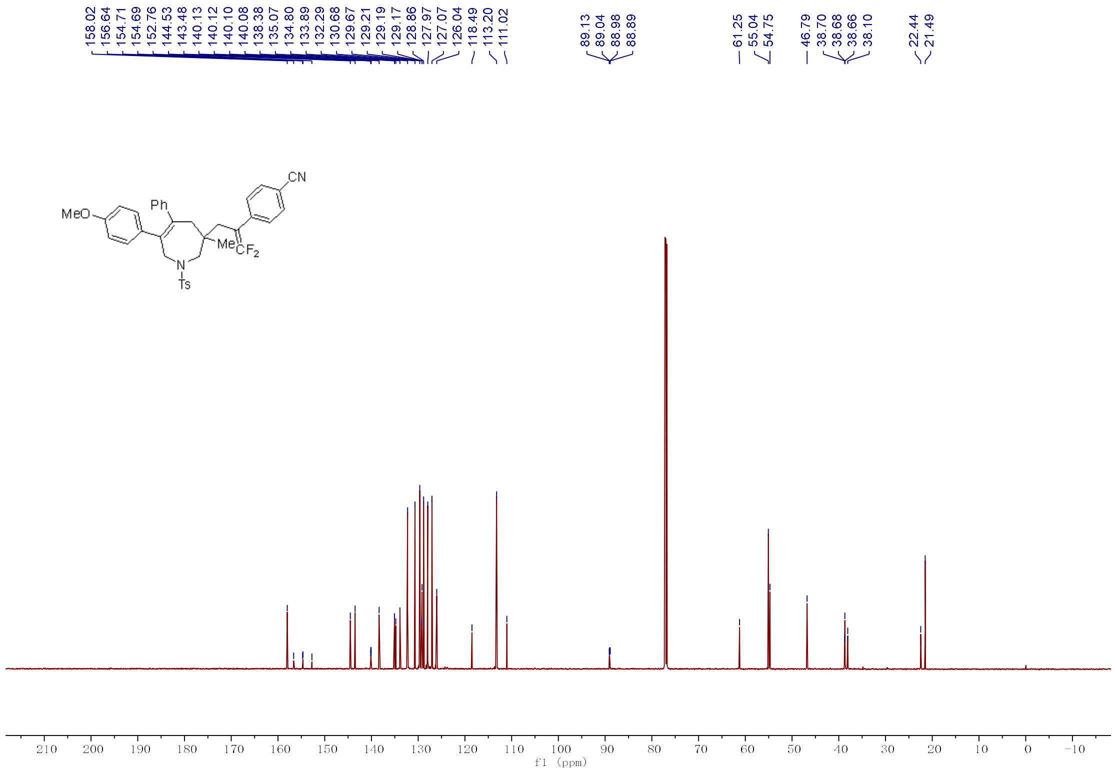

Supplementary figure 52. ^{13}C NMR of compound **14**

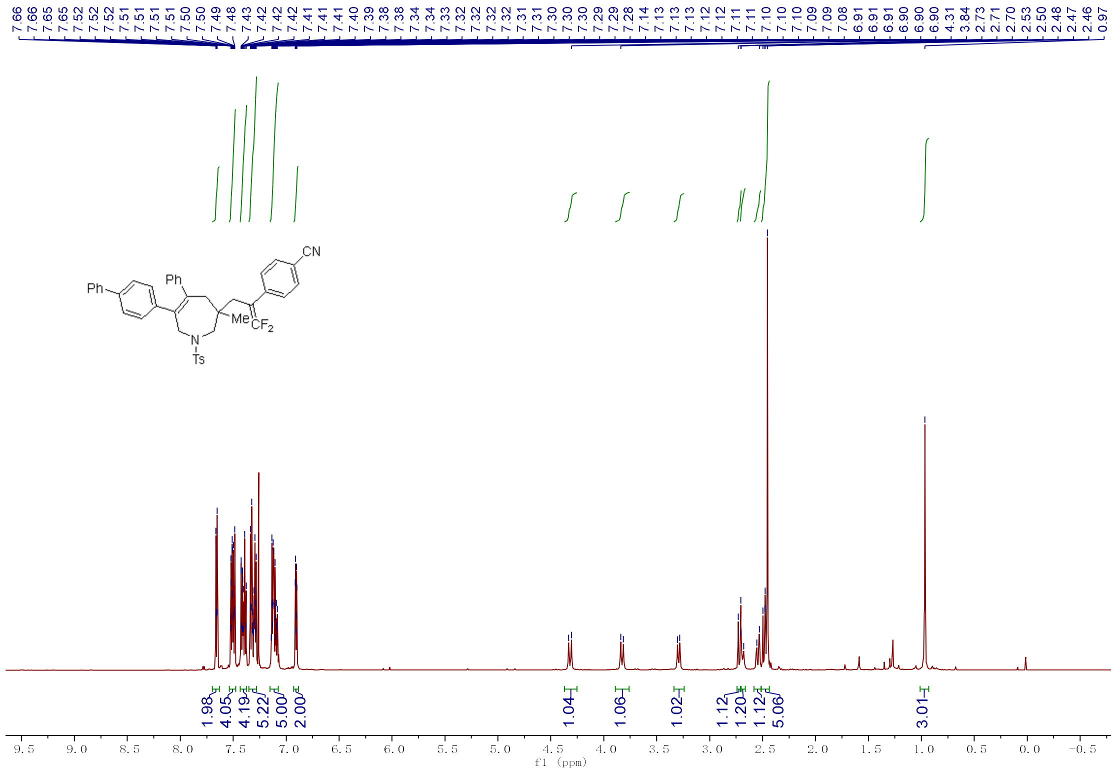

Supplementary figure 53. ^{1}H NMR of compound **15**

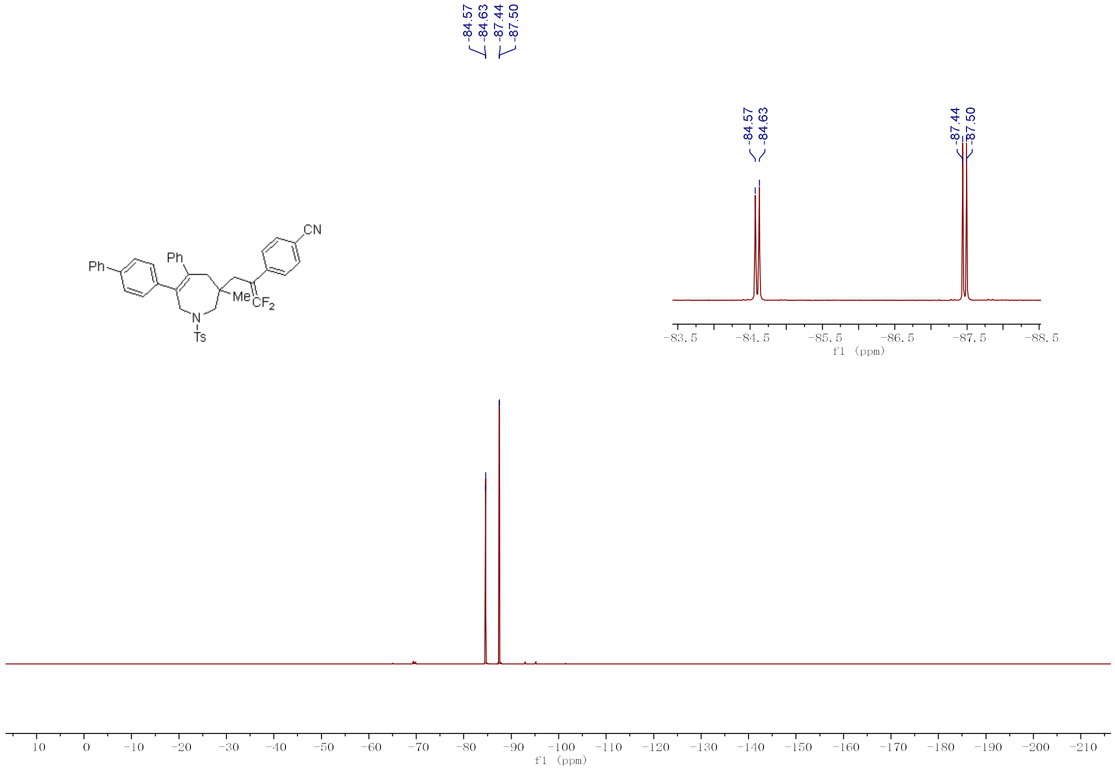

Supplementary figure 54. ^{19}F NMR of compound **15**

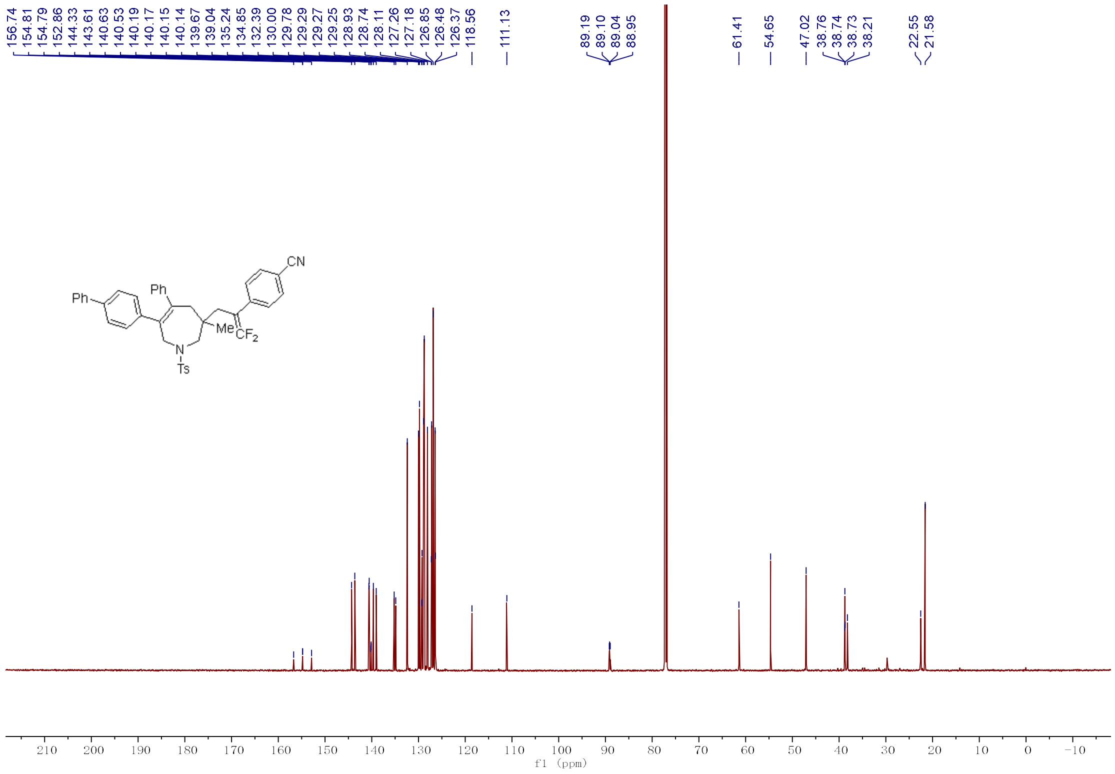

Supplementary figure 55. ^{13}C NMR of compound **15**

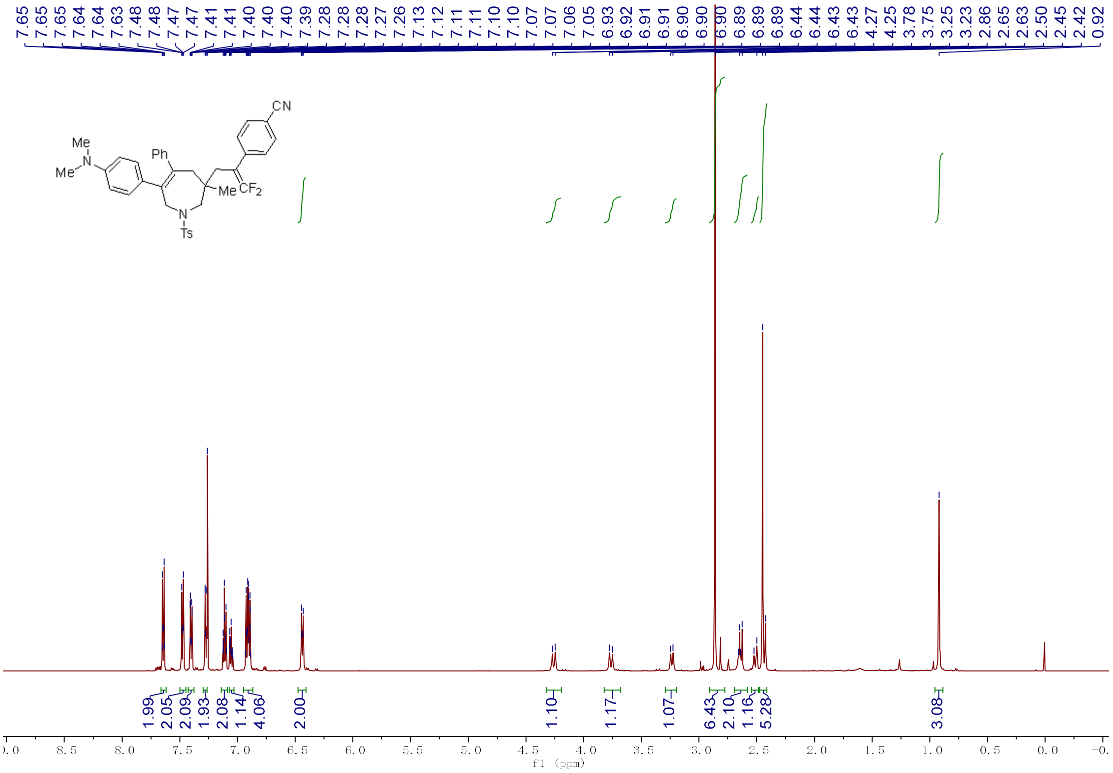

Supplementary figure 56. ^{1}H NMR of compound **16**

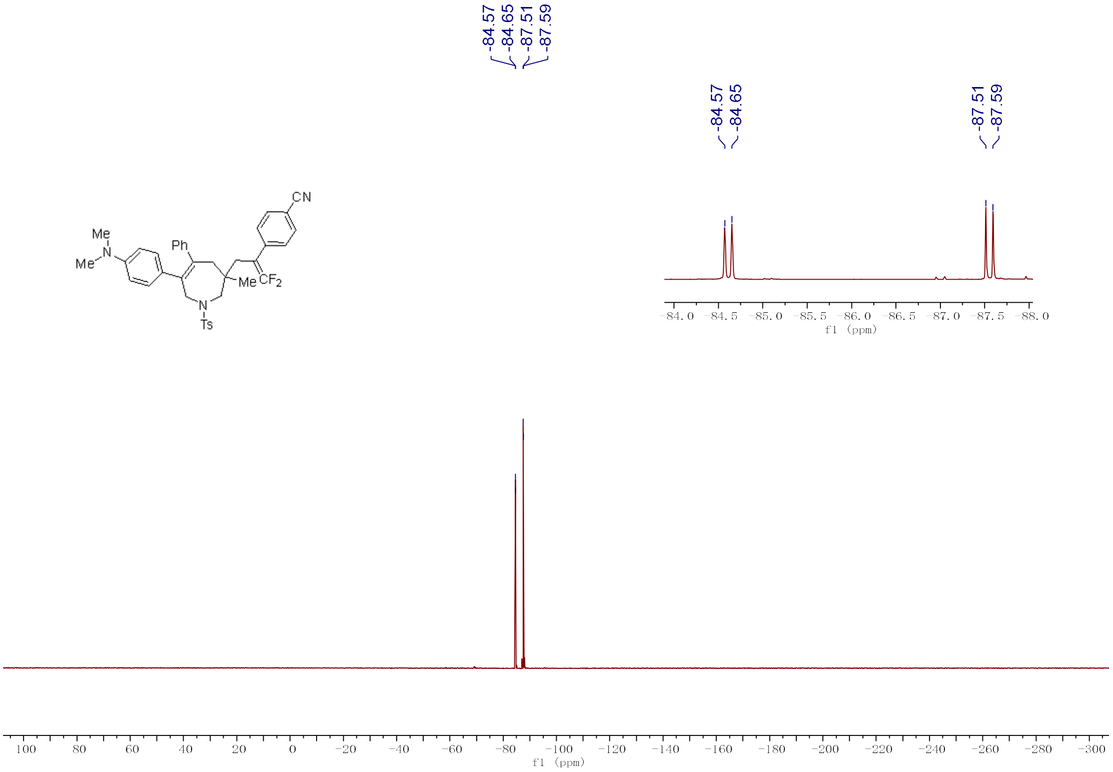

Supplementary figure 57. ^{19}F NMR of compound **16**

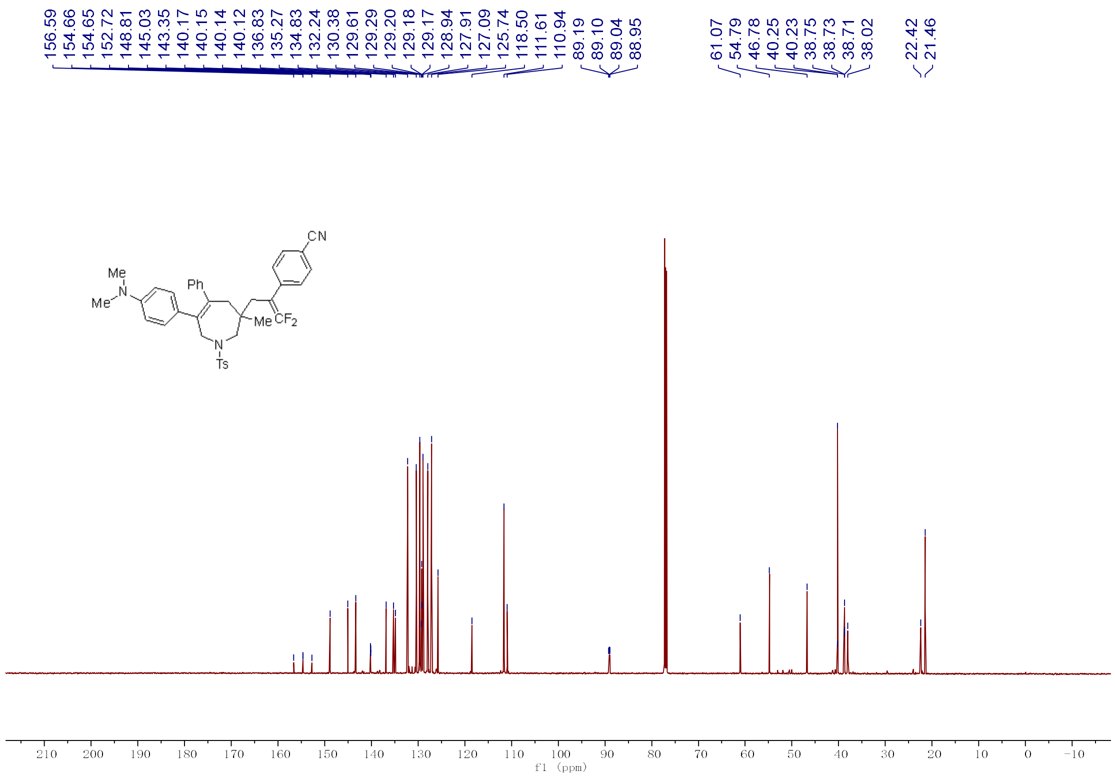

Supplementary figure 58. ^{13}C NMR of compound **16**

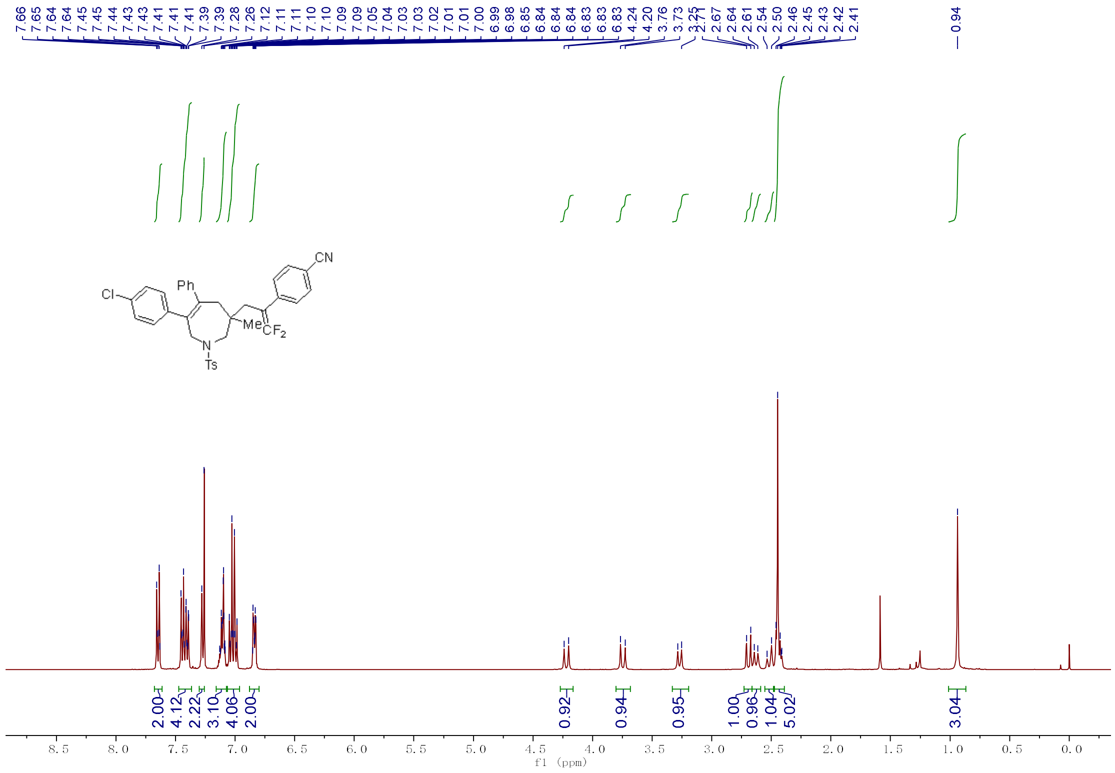

Supplementary figure 59. ^{1}H NMR of compound **17**

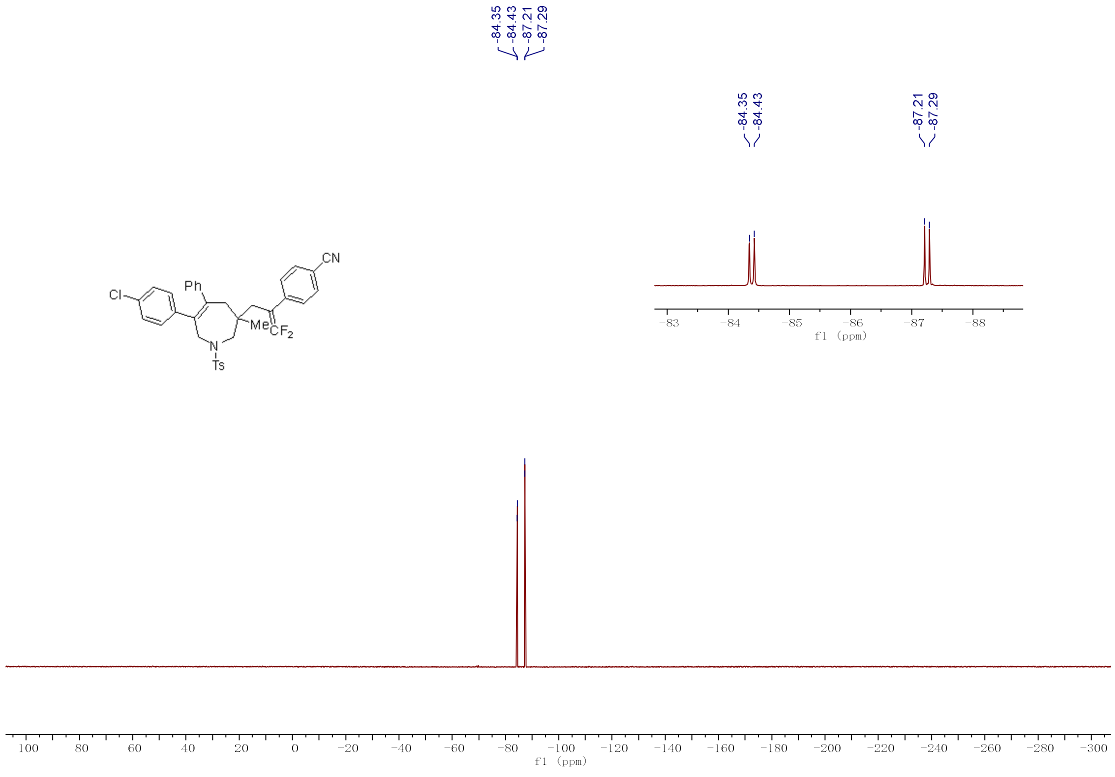

Supplementary figure 60. ^{19}F NMR of compound **17**

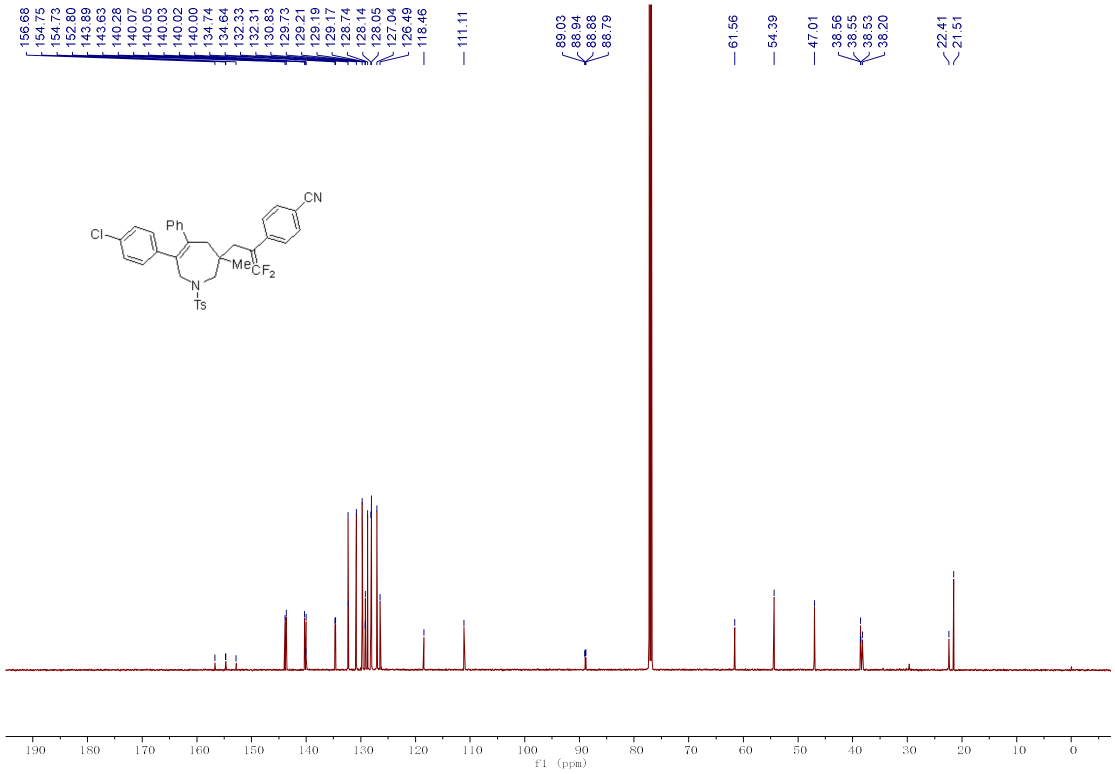

Supplementary figure 61. ^{13}C NMR of compound **17**

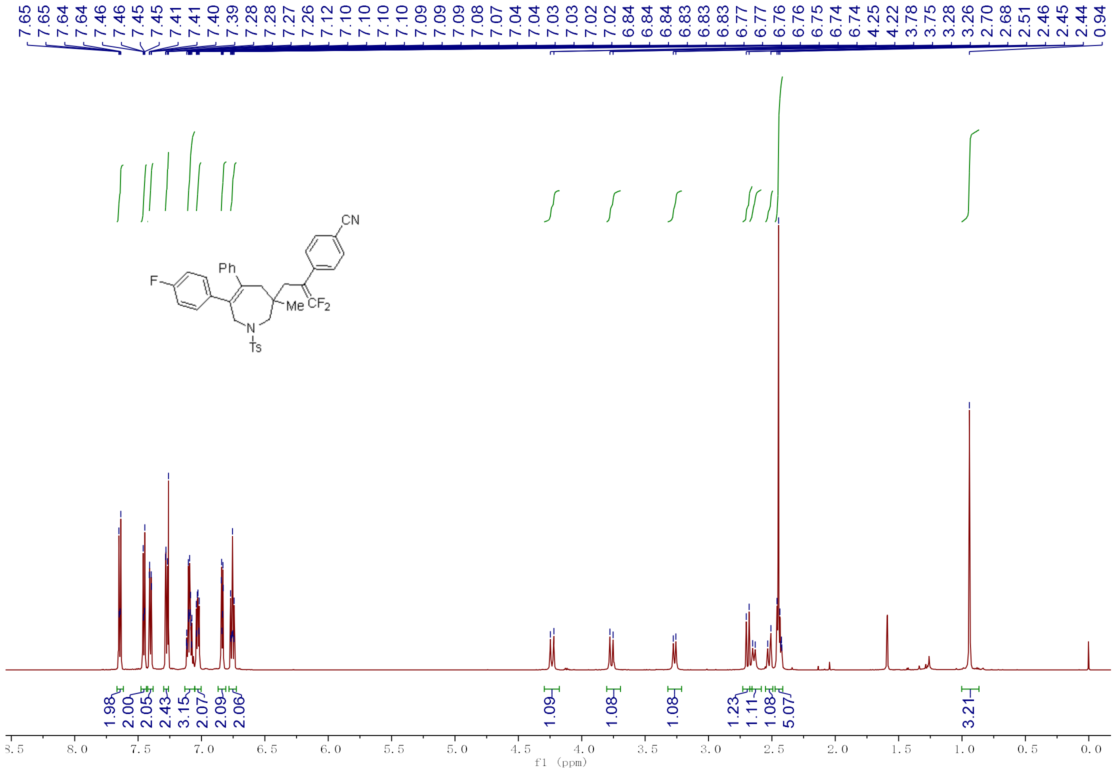

Supplementary figure 62. ^{1}H NMR of compound **18**

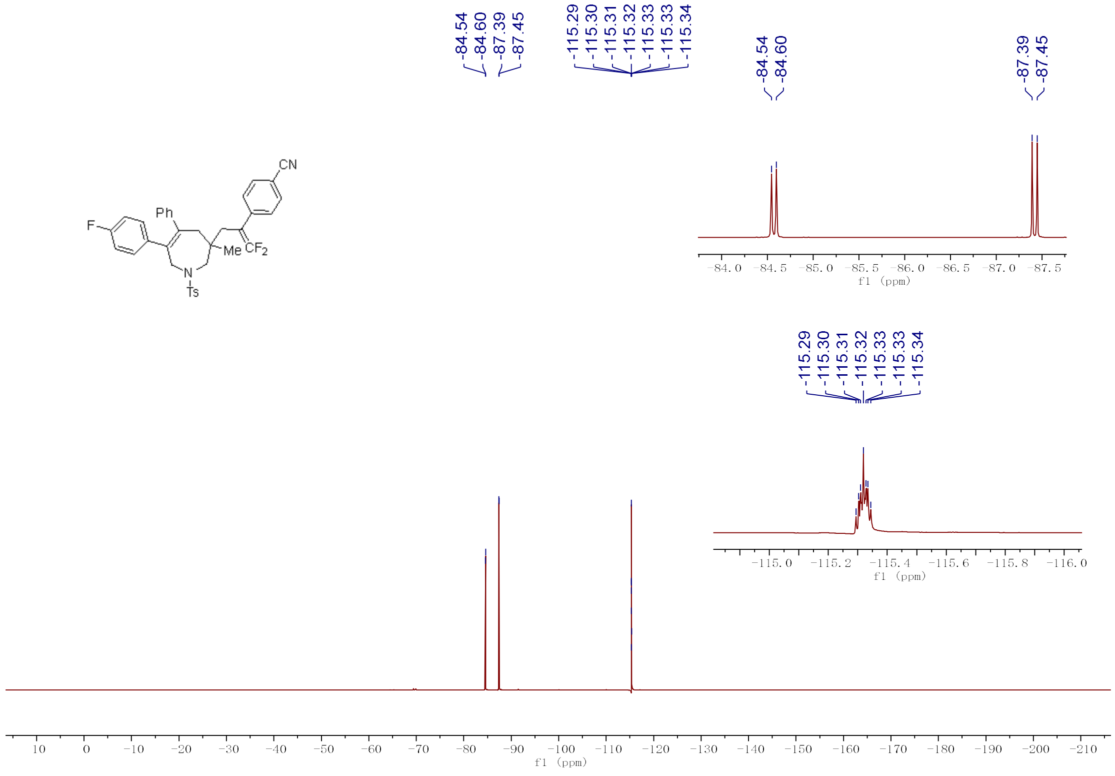

Supplementary figure 63. ^{19}F NMR of compound **18**

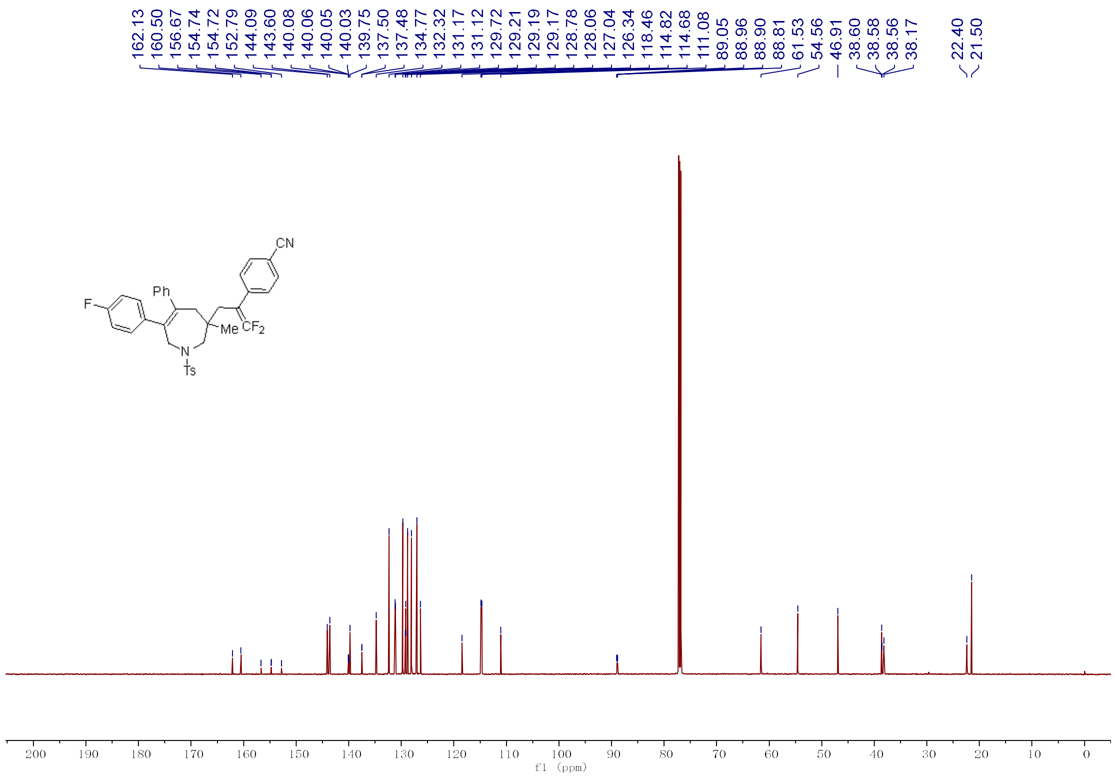

Supplementary figure 64. ^{13}C NMR of compound **18**

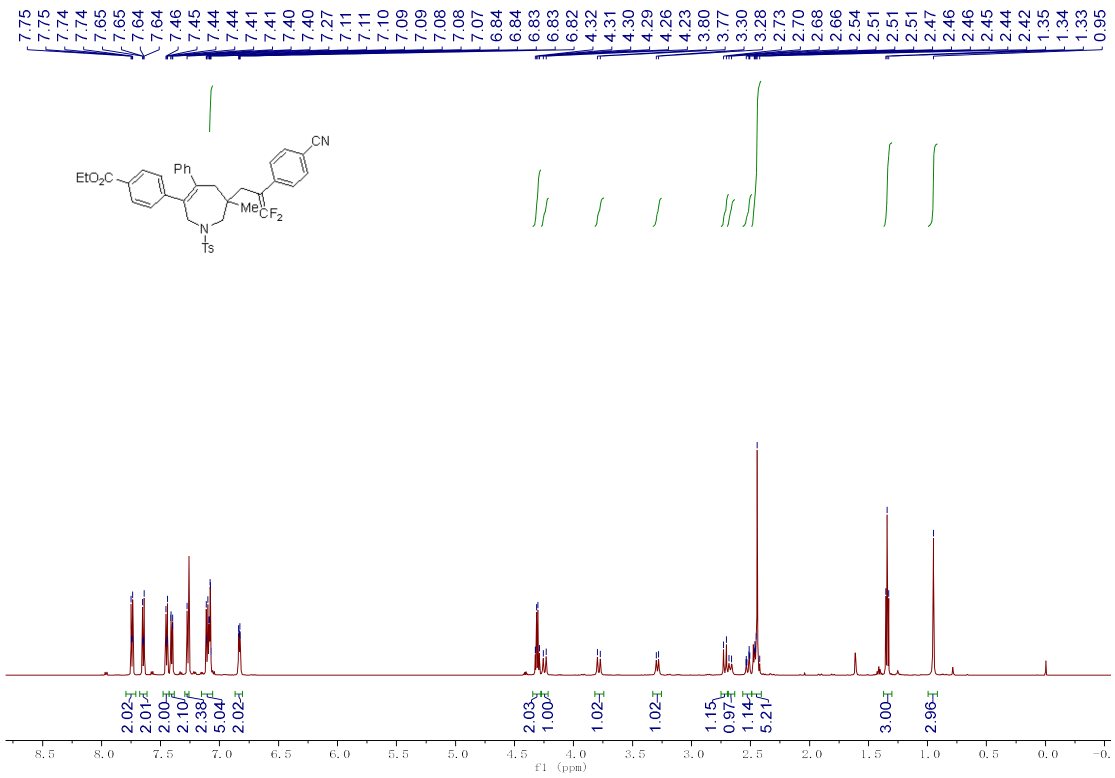

Supplementary figure 65. ^{1}H NMR of compound **19**

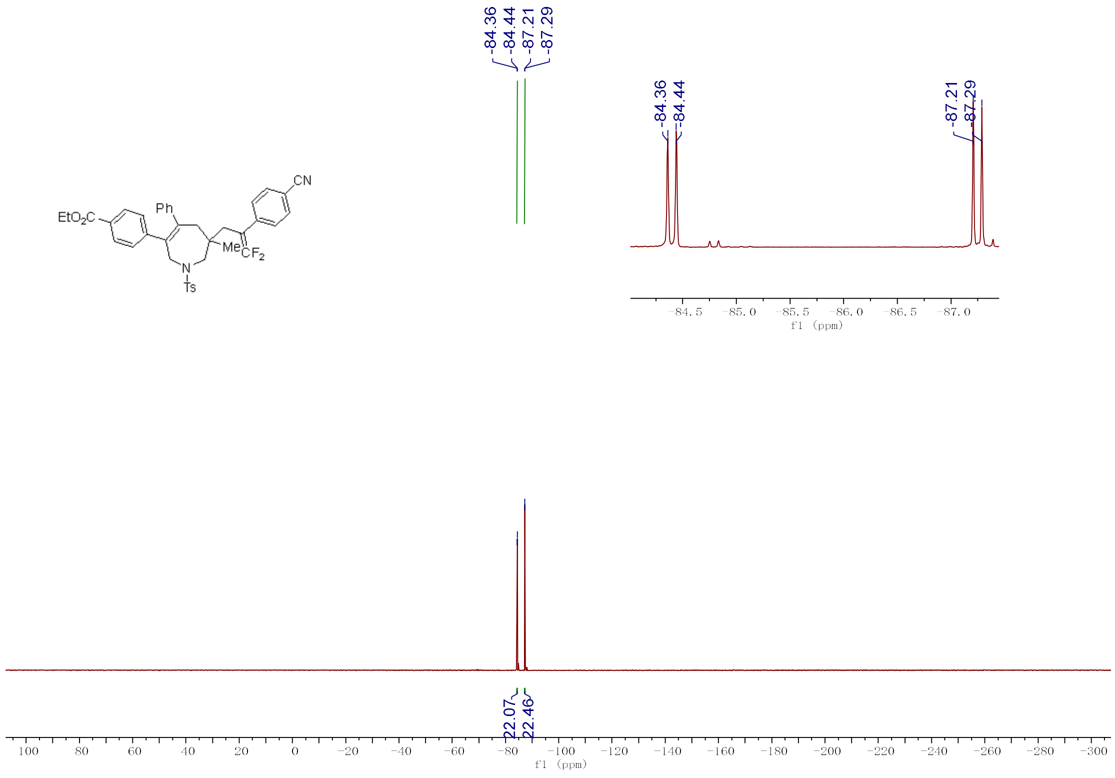

Supplementary figure 66. ^{19}F NMR of compound **19**

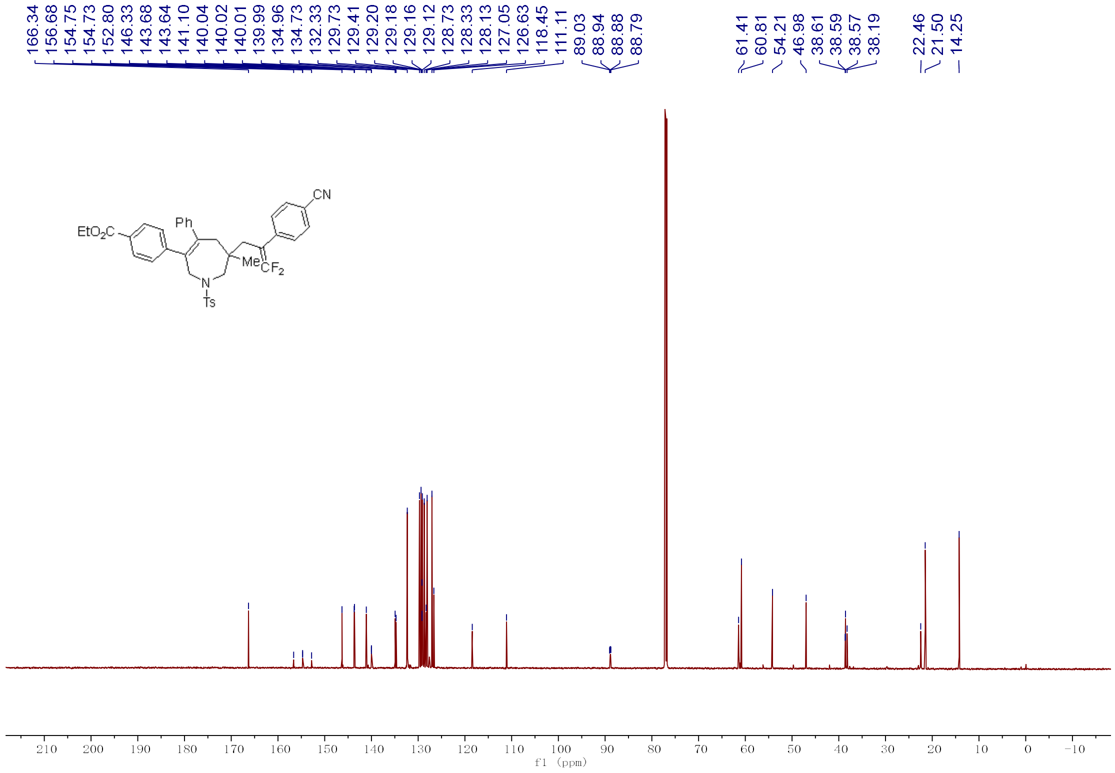

Supplementary figure 67. ^{13}C NMR of compound **19**

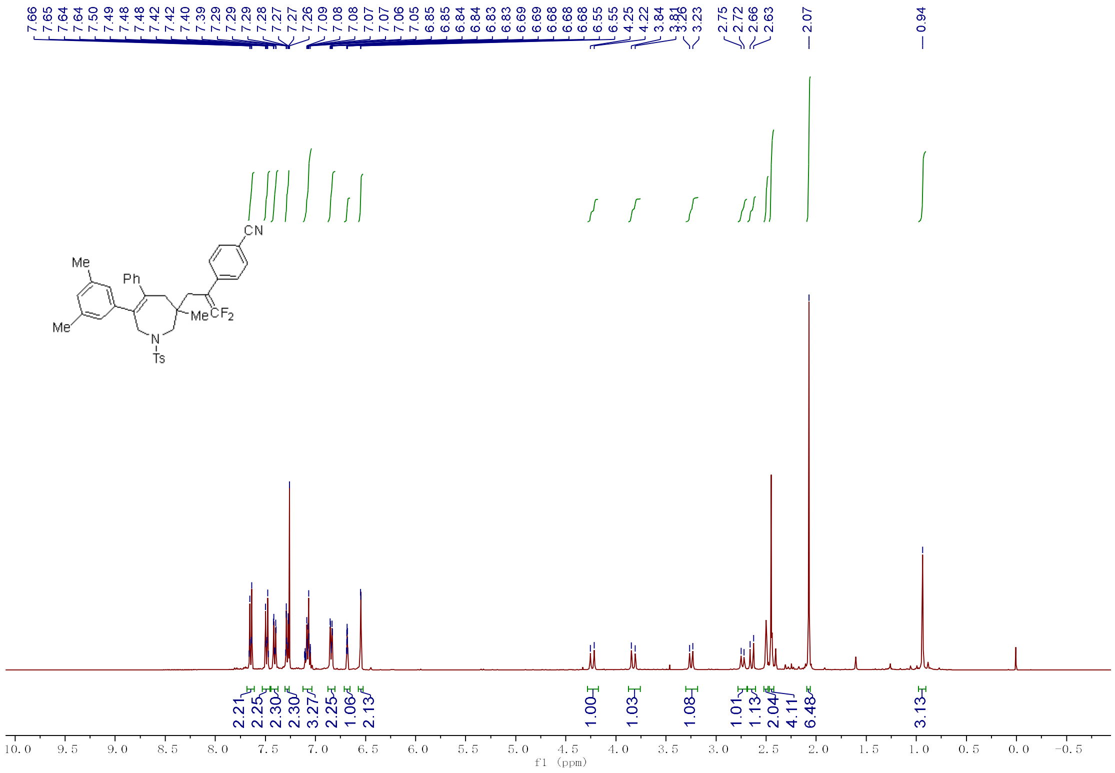

Supplementary figure 68. ^{1}H NMR of compound **20**

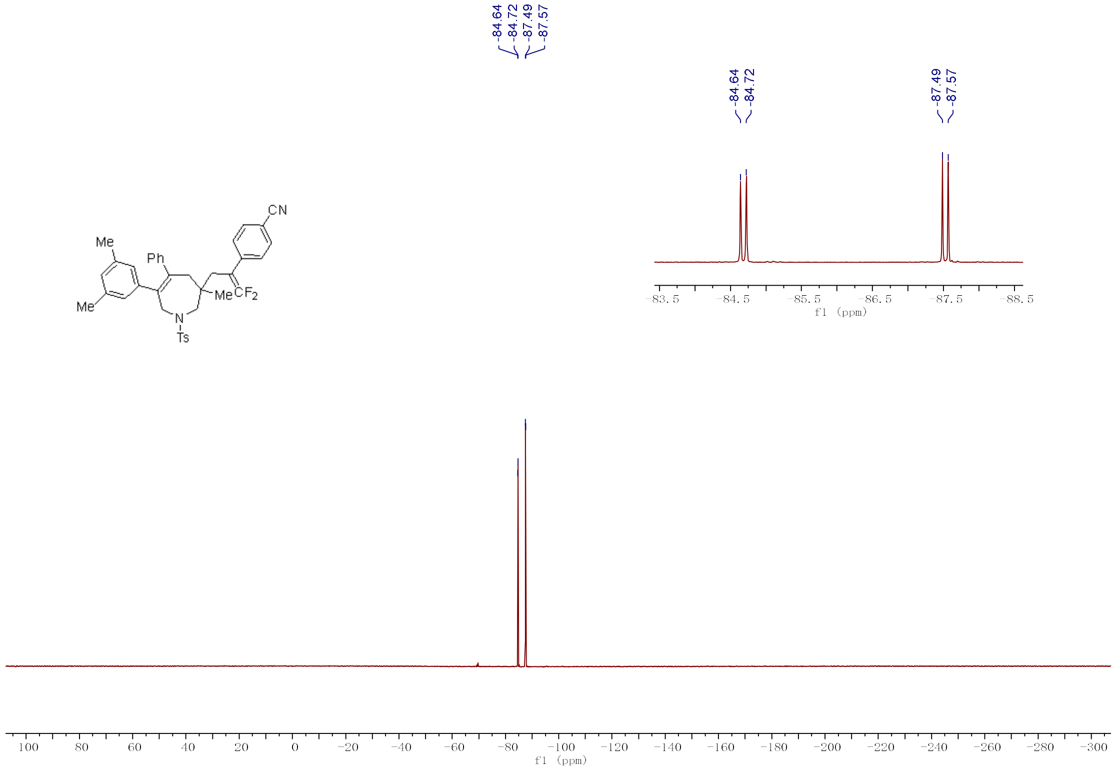

Supplementary figure 69. ^{19}F NMR of compound **20**

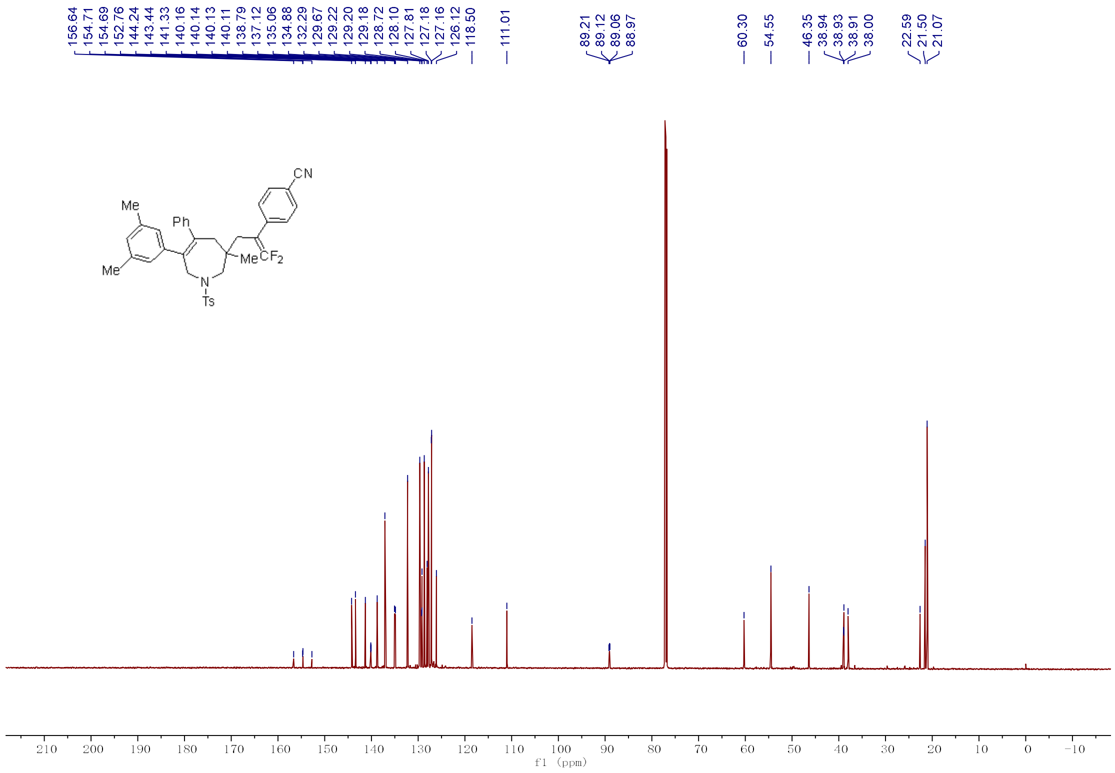

Supplementary figure 70. ^{13}C NMR of compound **20**

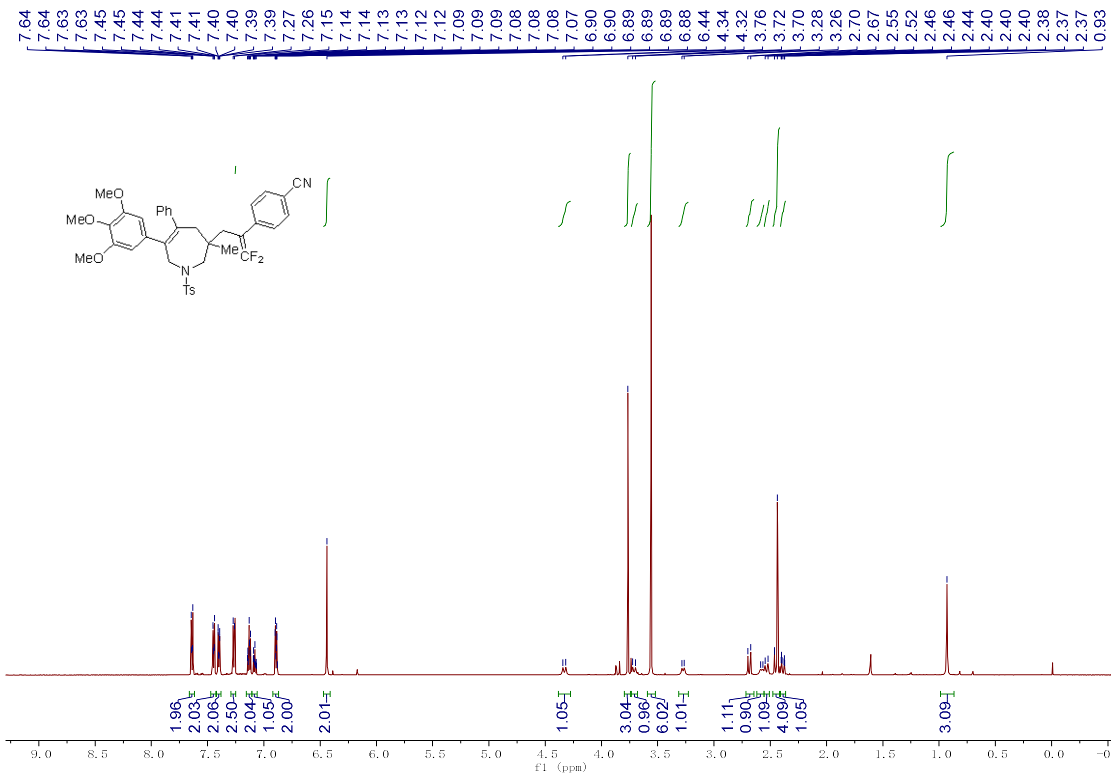

Supplementary figure 71. ^{1}H NMR of compound **21**

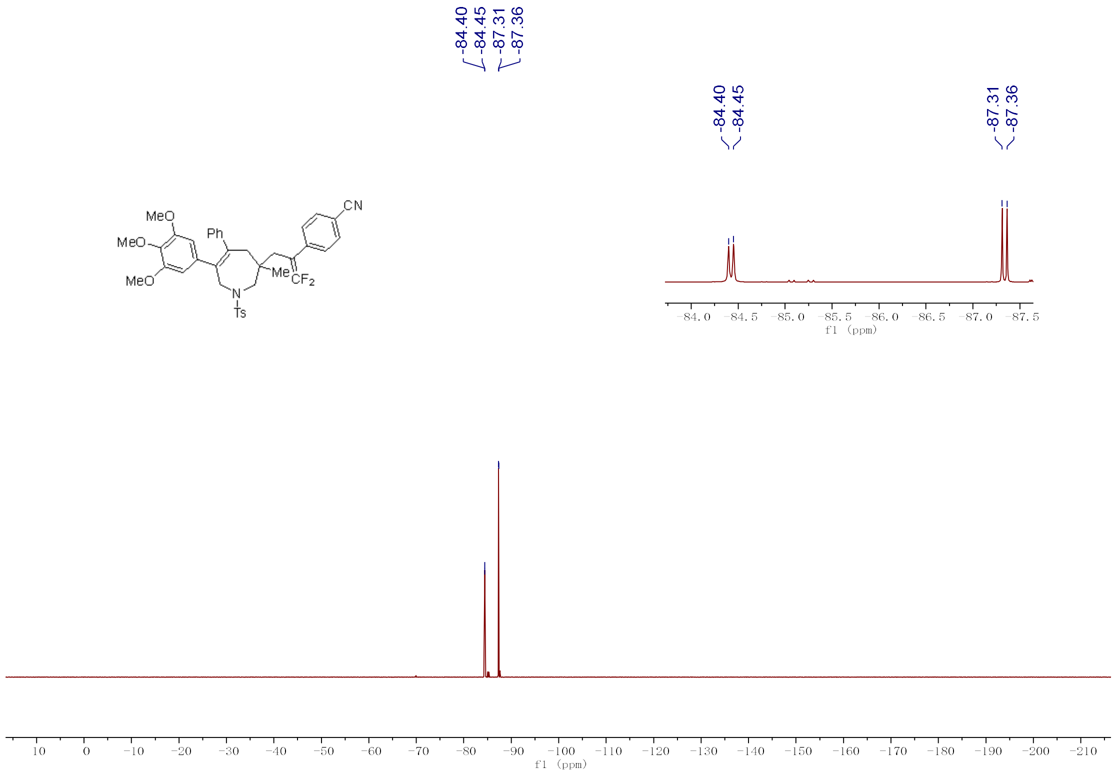

Supplementary figure 72. ^{19}F NMR of compound **21**

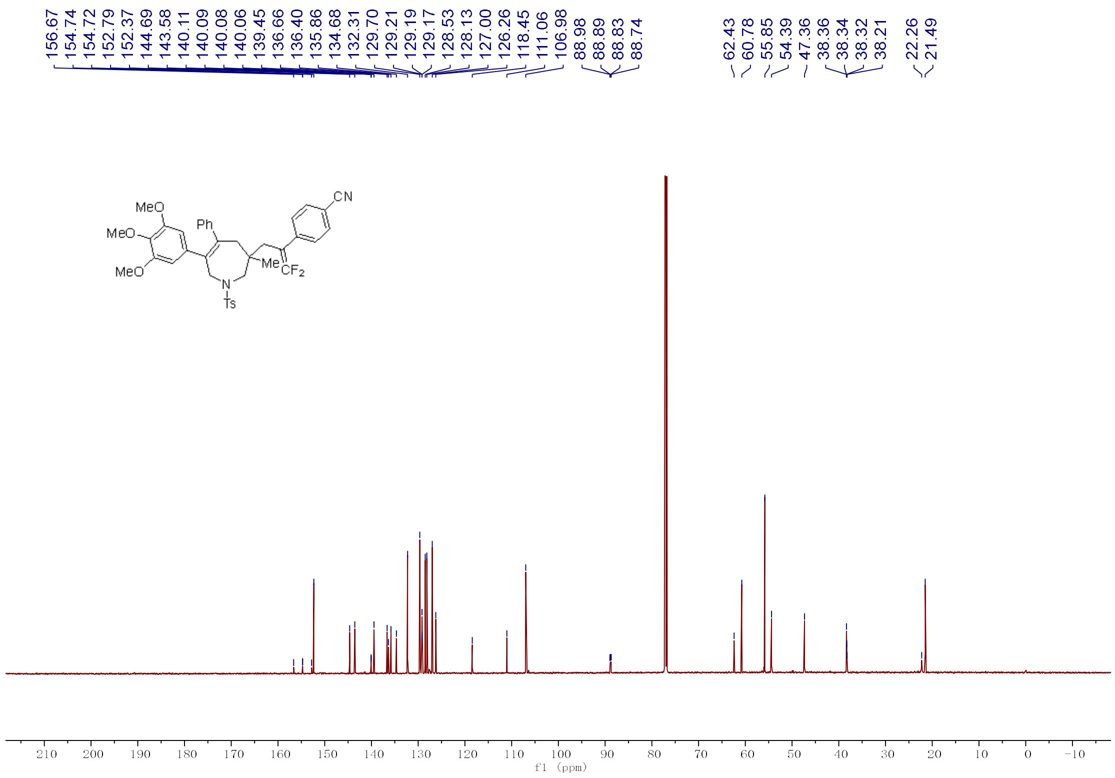

Supplementary figure 73. ^{13}C NMR of compound **21**

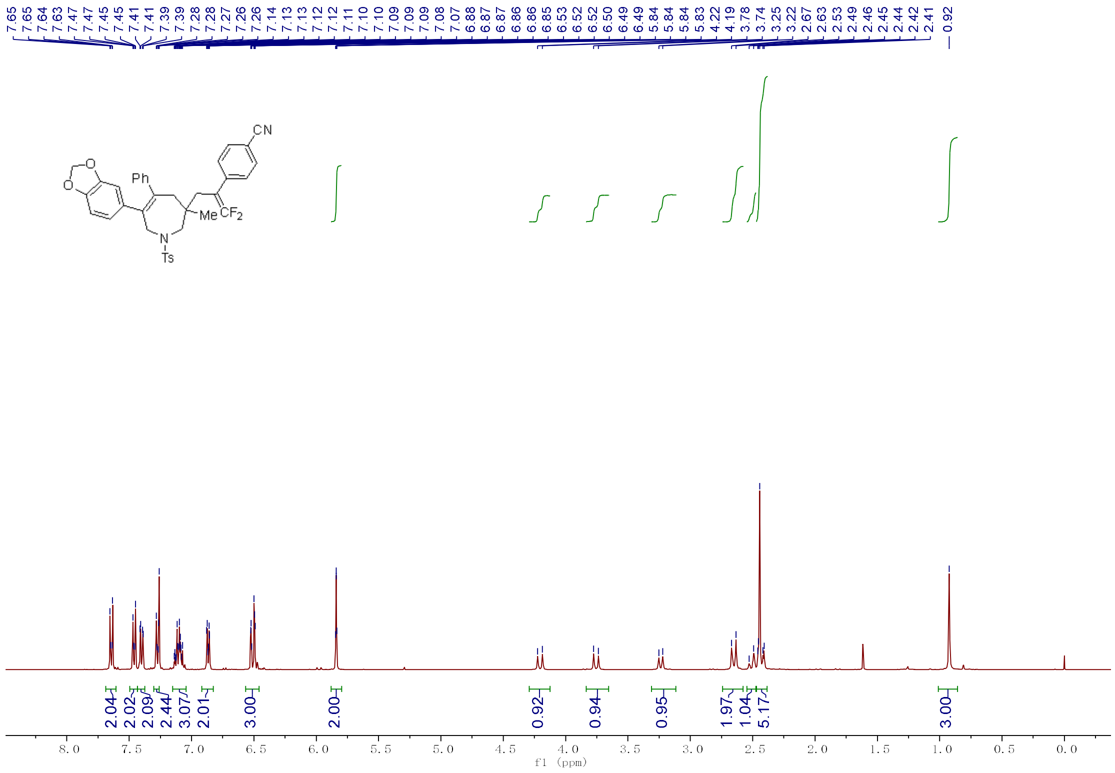

Supplementary figure 74. ^{1}H NMR of compound **22**

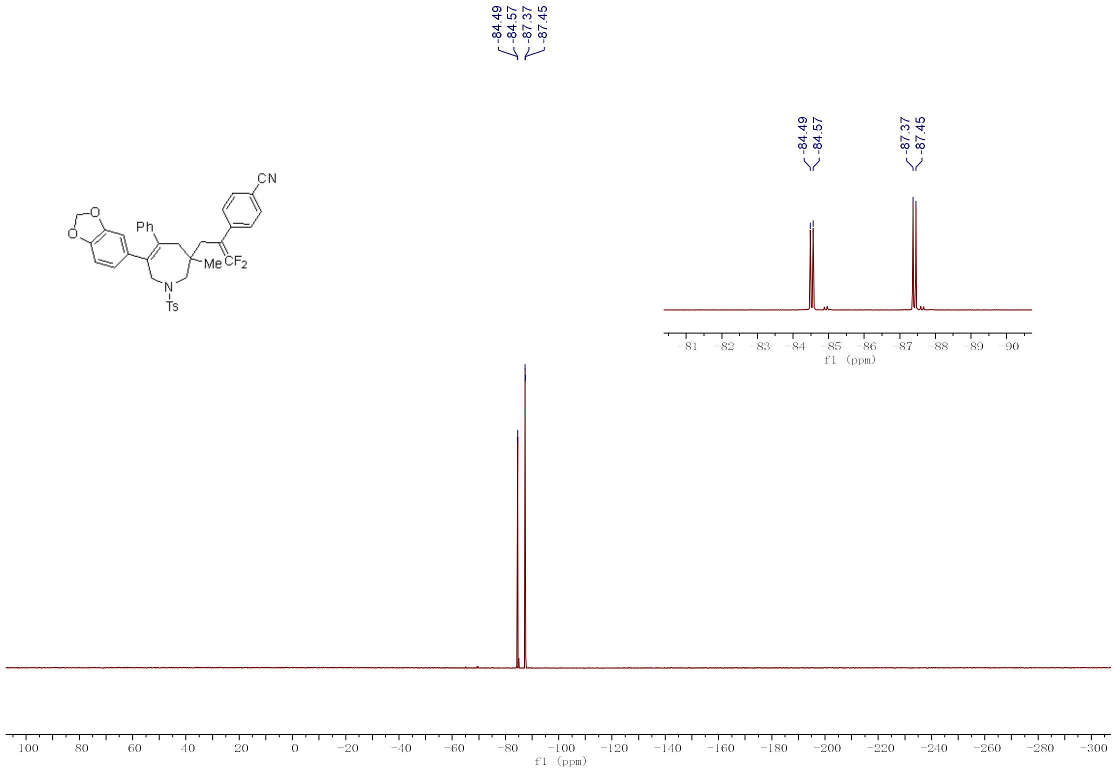

Supplementary figure 75. ^{19}F NMR of compound **22**

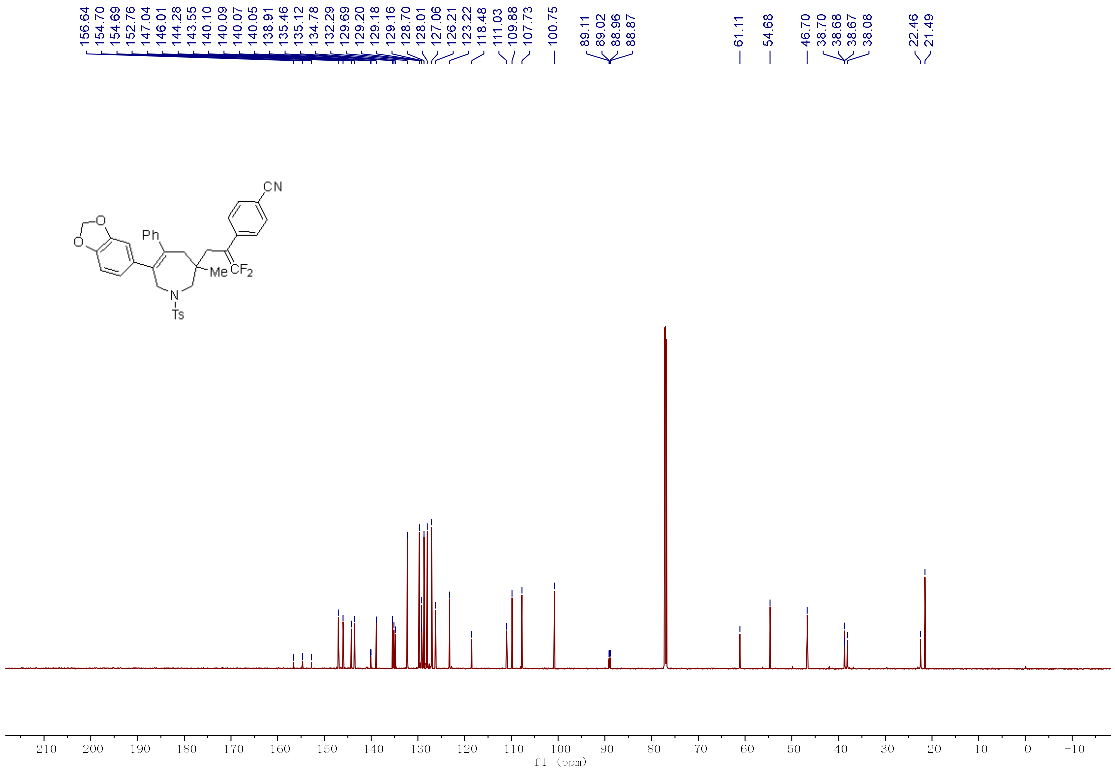

Supplementary figure 76. ^{13}C NMR of compound **22**

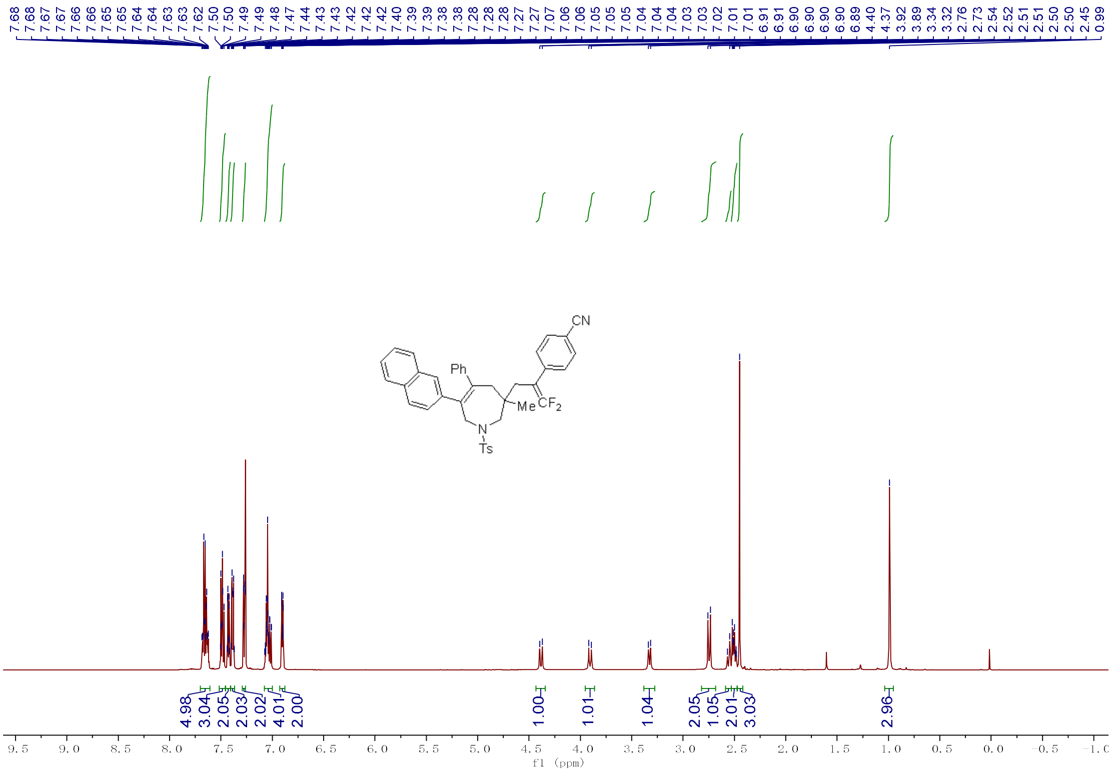

Supplementary figure 77. ^{1}H NMR of compound **23**

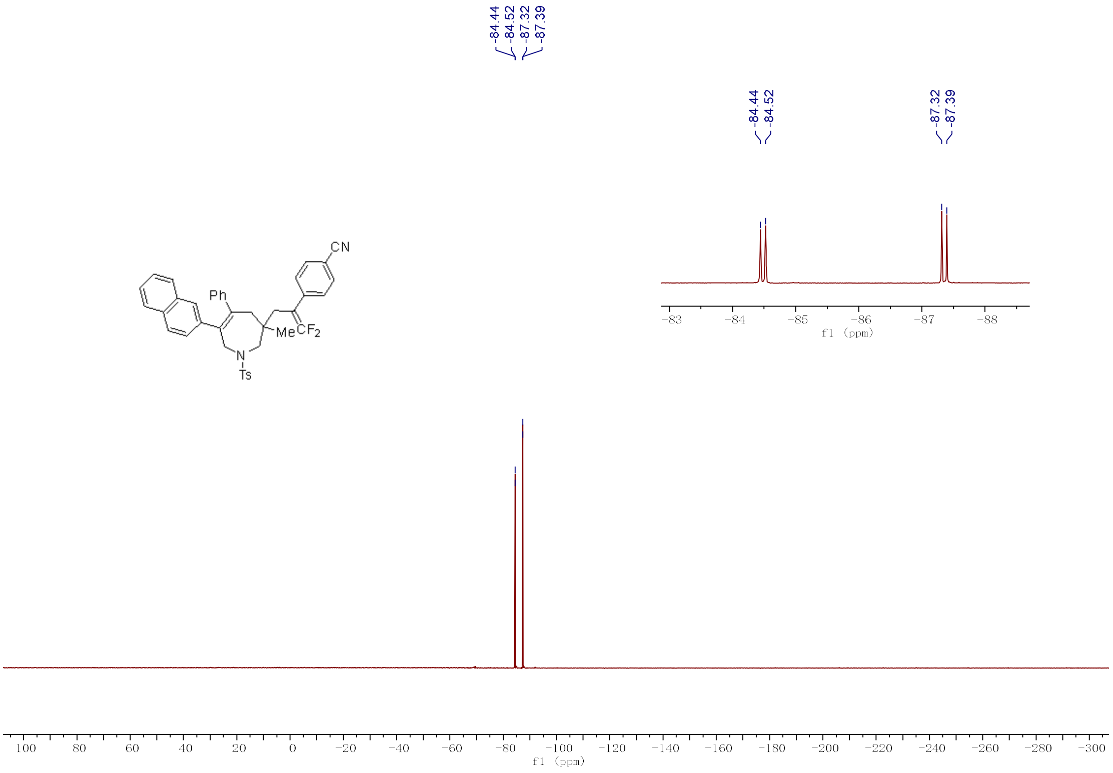

Supplementary figure 78. ^{19}F NMR of compound **23**

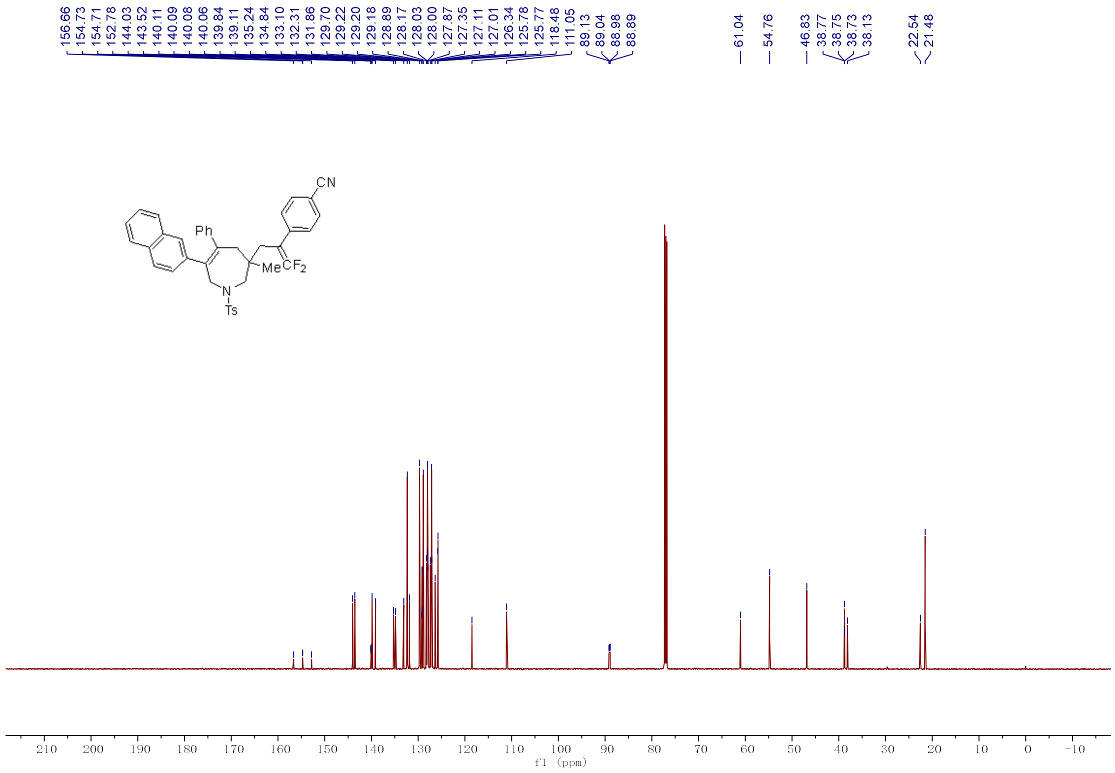

Supplementary figure 79. ^{13}C NMR of compound **23**

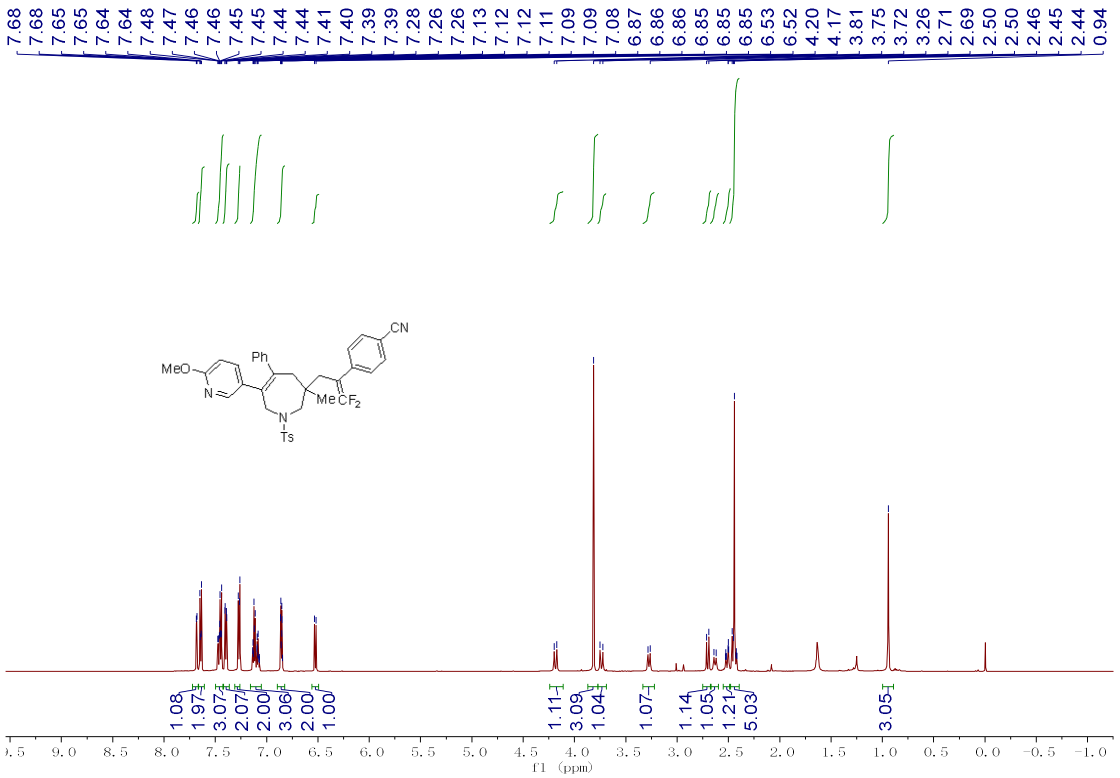

Supplementary figure 80. ^{1}H NMR of compound **24**

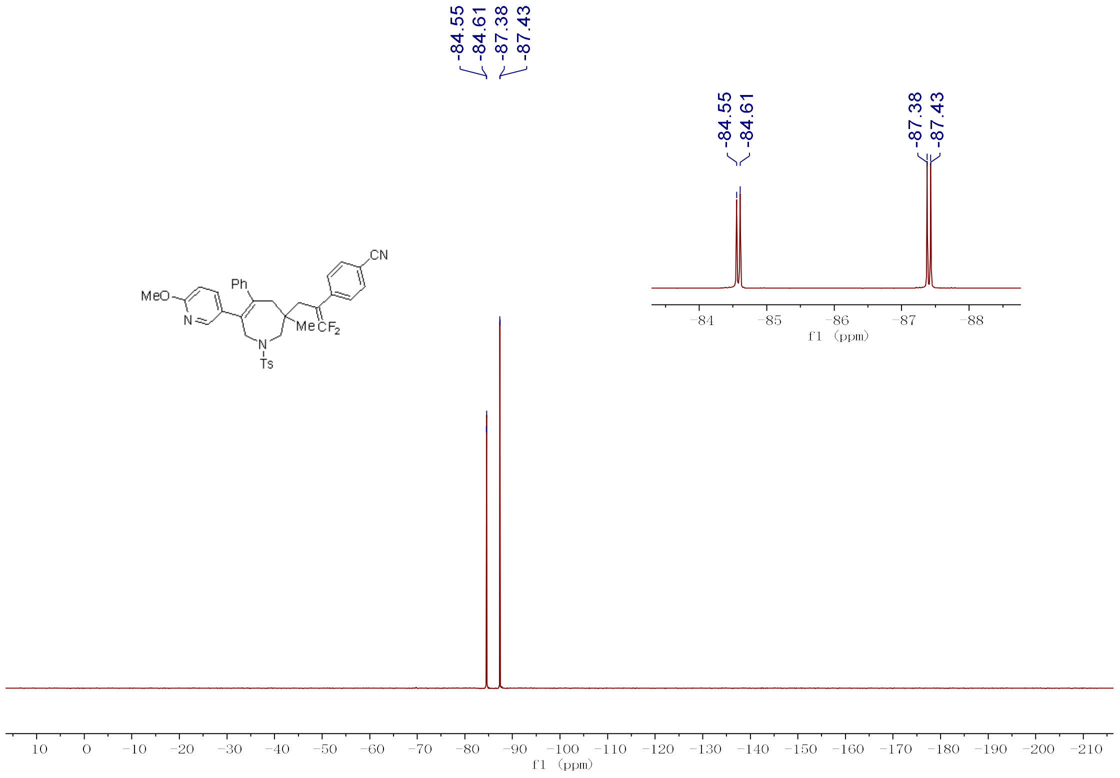

Supplementary figure 81. ^{19}F NMR of compound **24**

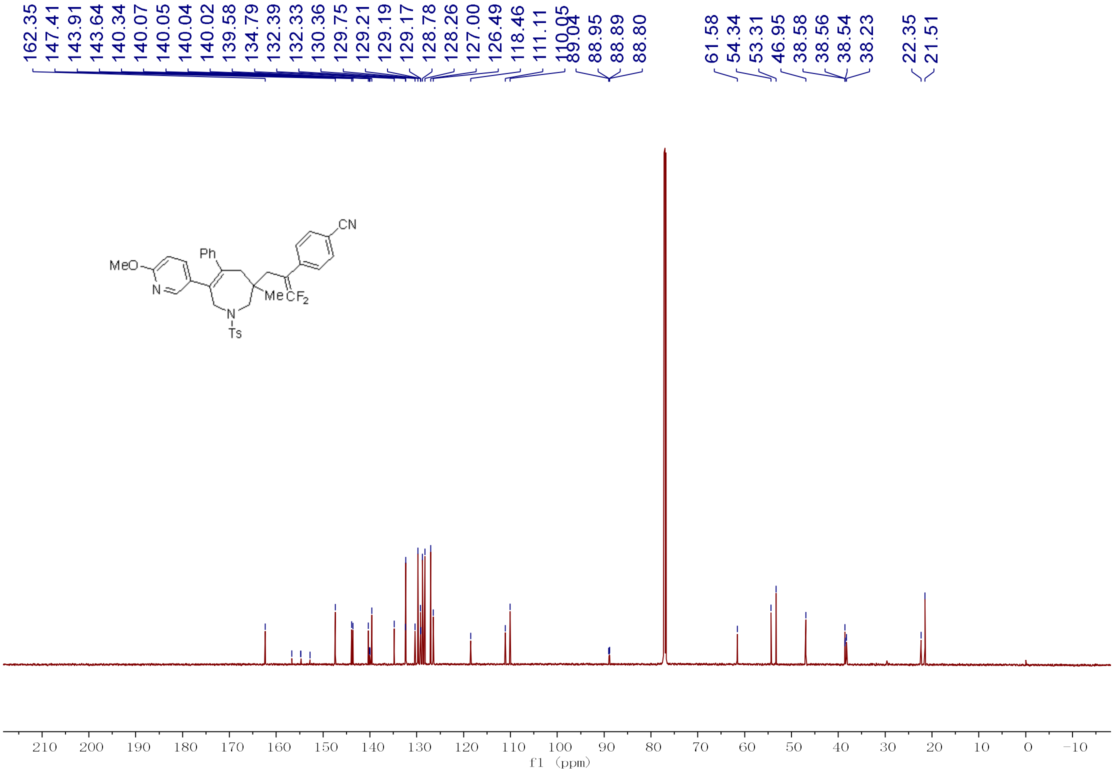

Supplementary figure 82. ^{13}C NMR of compound **24**

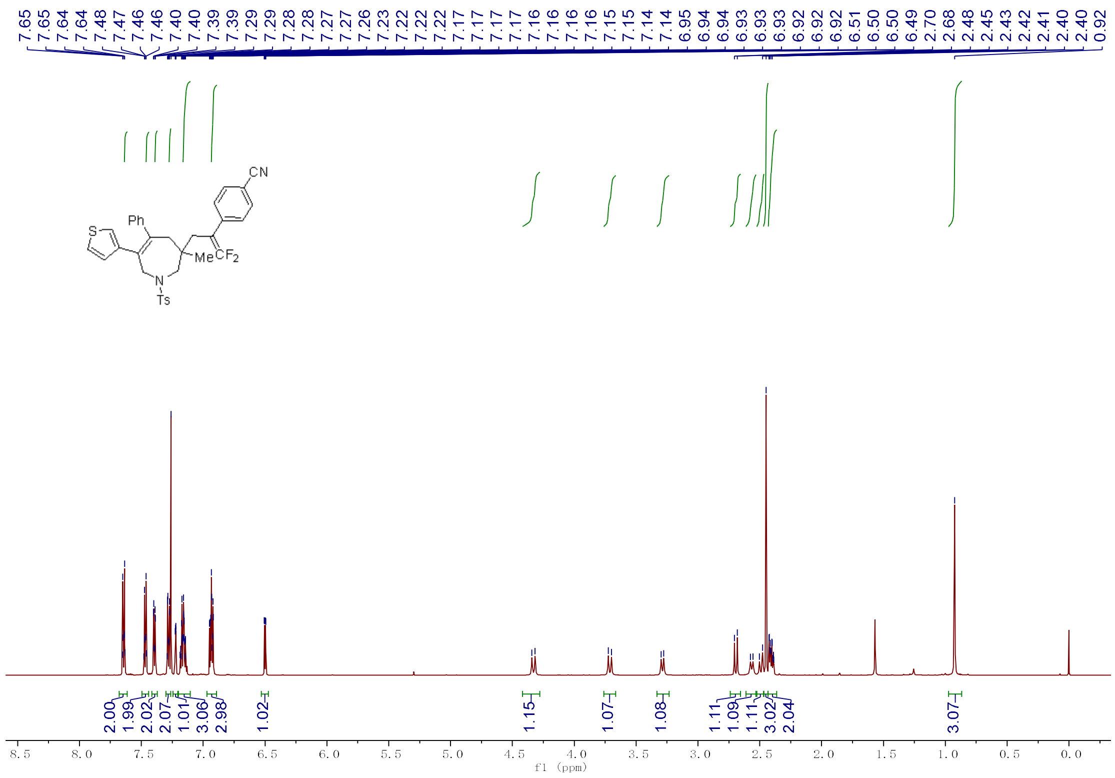

Supplementary figure 83. ^{1}H NMR of compound **25**

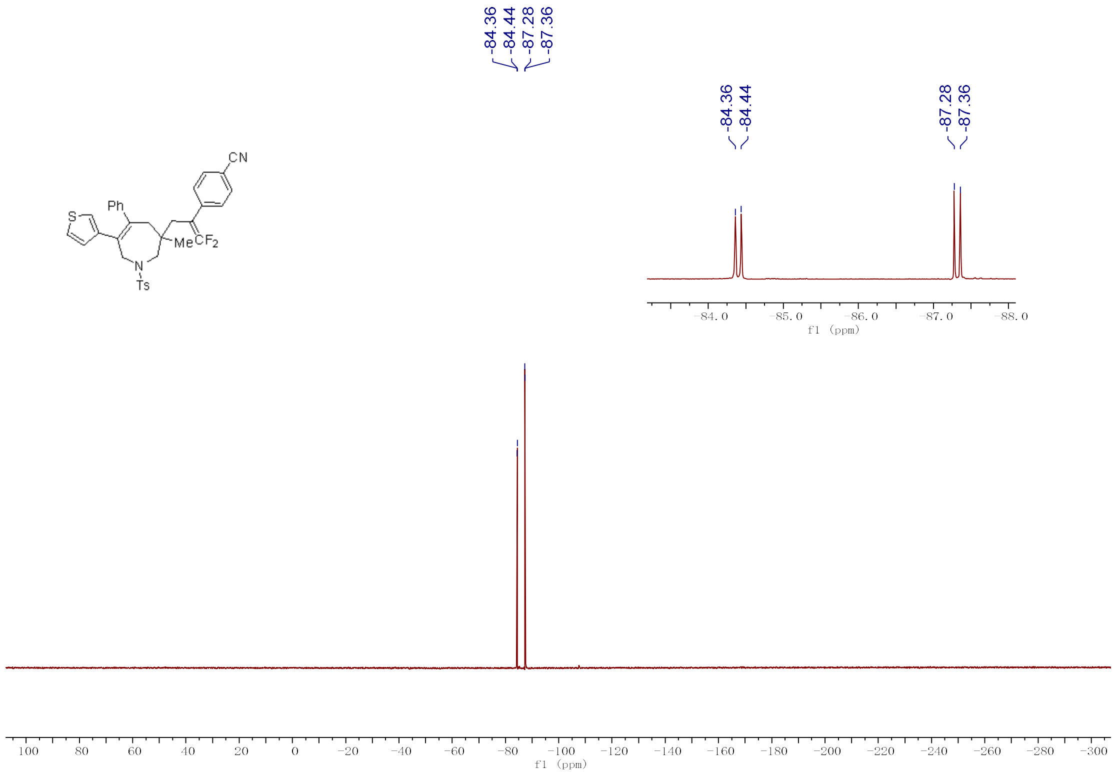

Supplementary figure 84. ^{19}F NMR of compound **25**

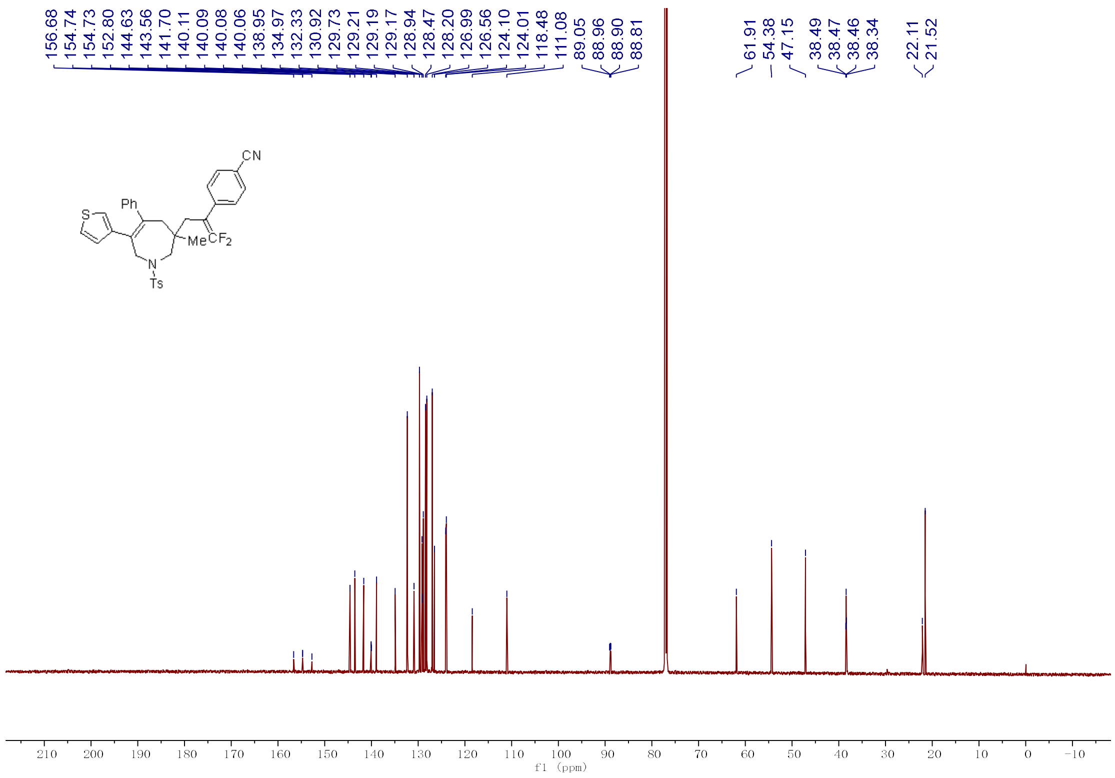

Supplementary figure 85. ^{13}C NMR of compound **25**

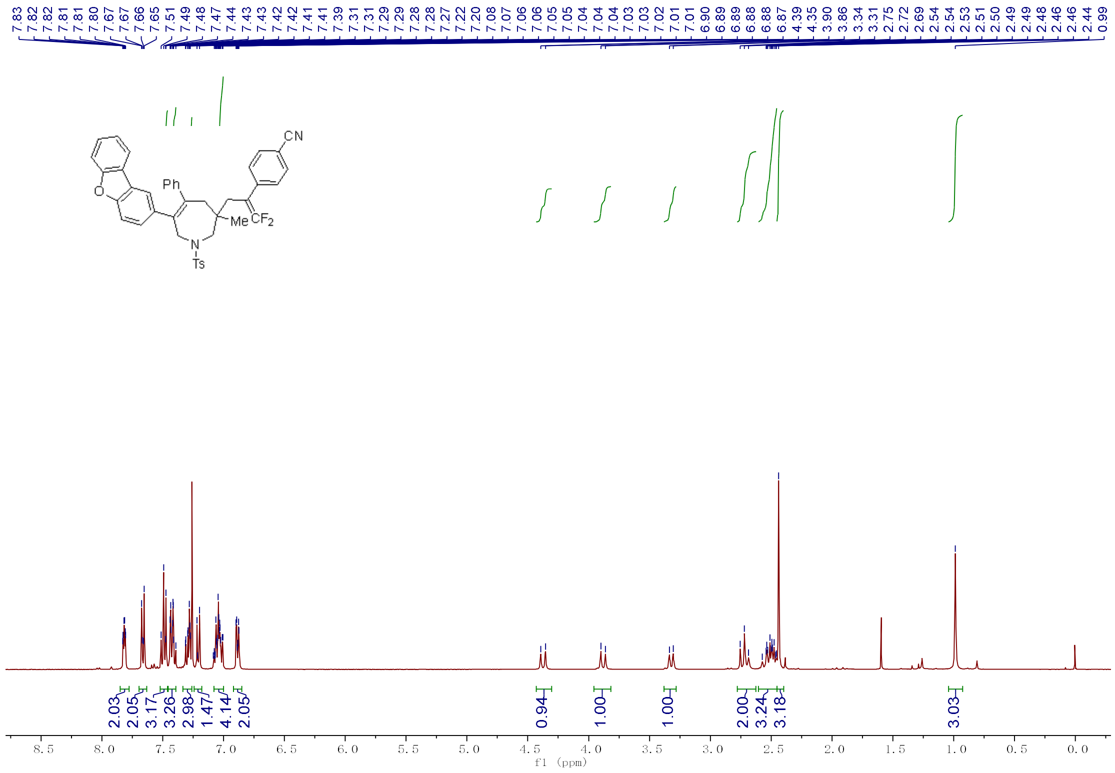

Supplementary figure 86. ^{1}H NMR of compound **26**

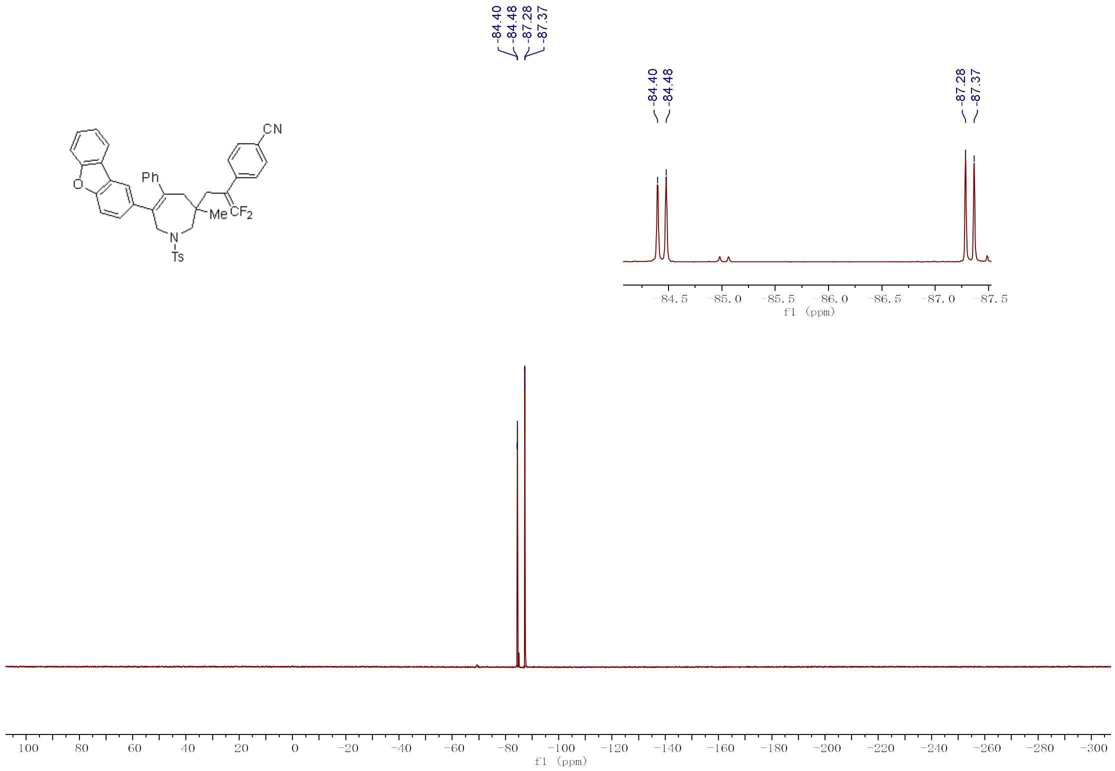

Supplementary figure 87. ^{19}F NMR of compound **26**

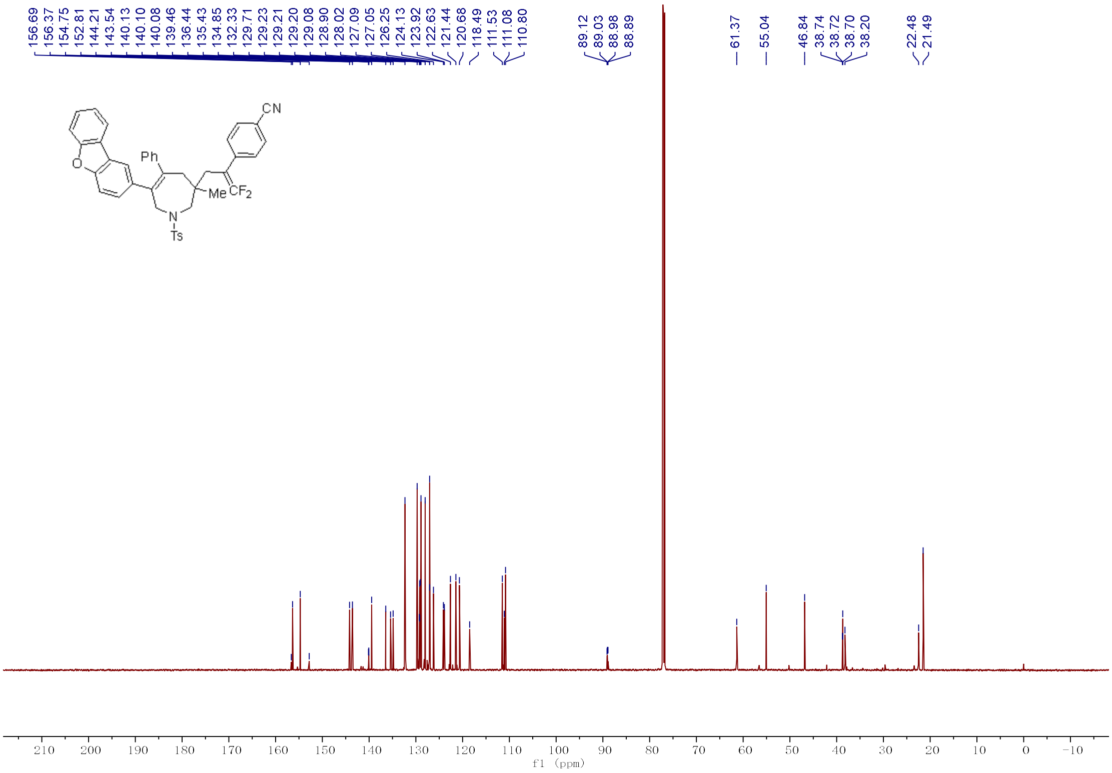

Supplementary figure 88. ^{13}C NMR of compound **26**

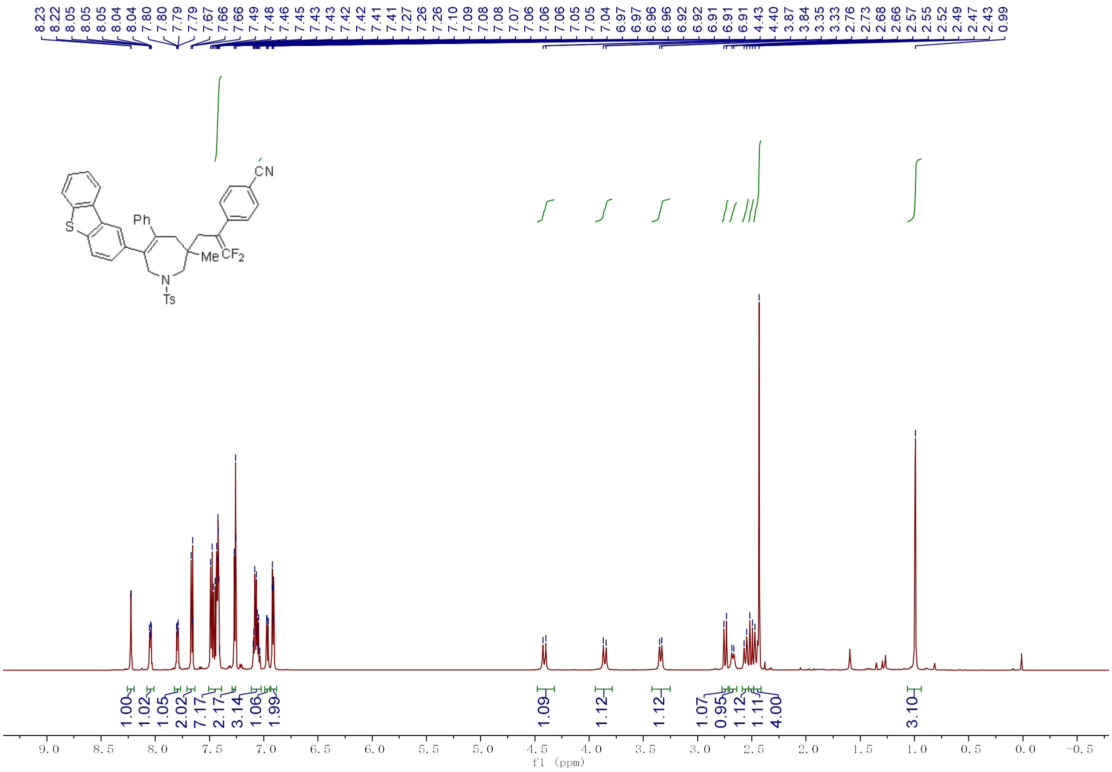

Supplementary figure 89. ^{1}H NMR of compound **27**

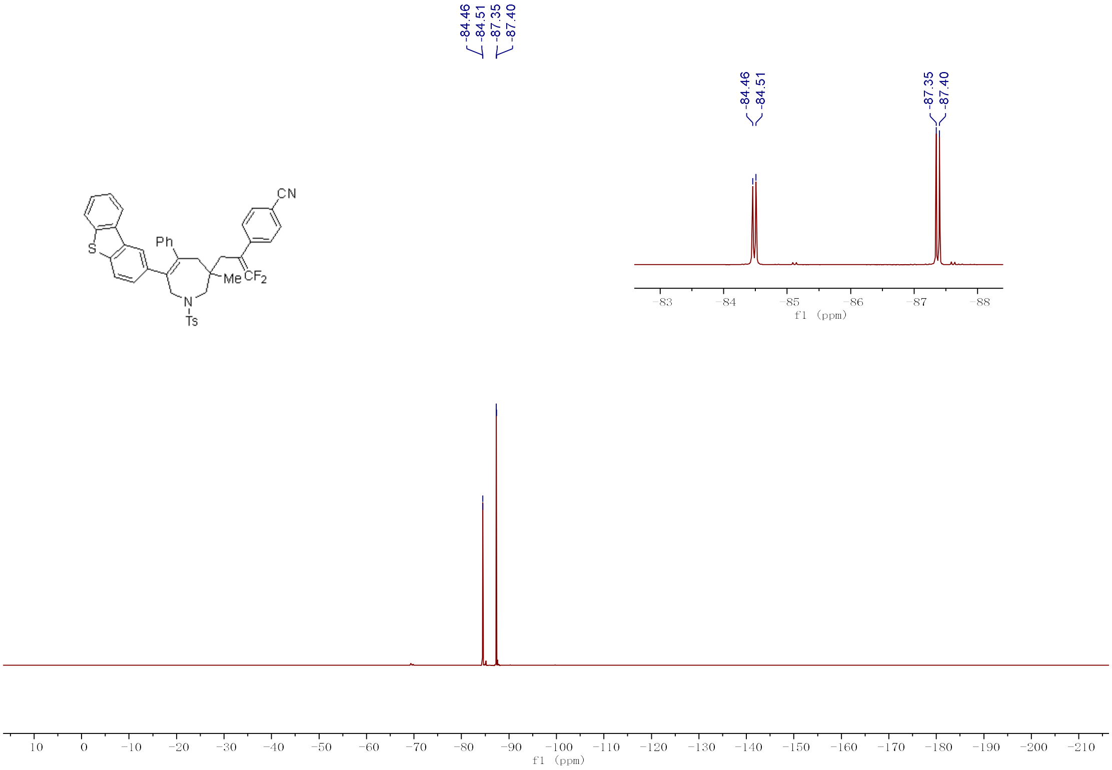

Supplementary figure 90. ^{19}F NMR of compound **27**

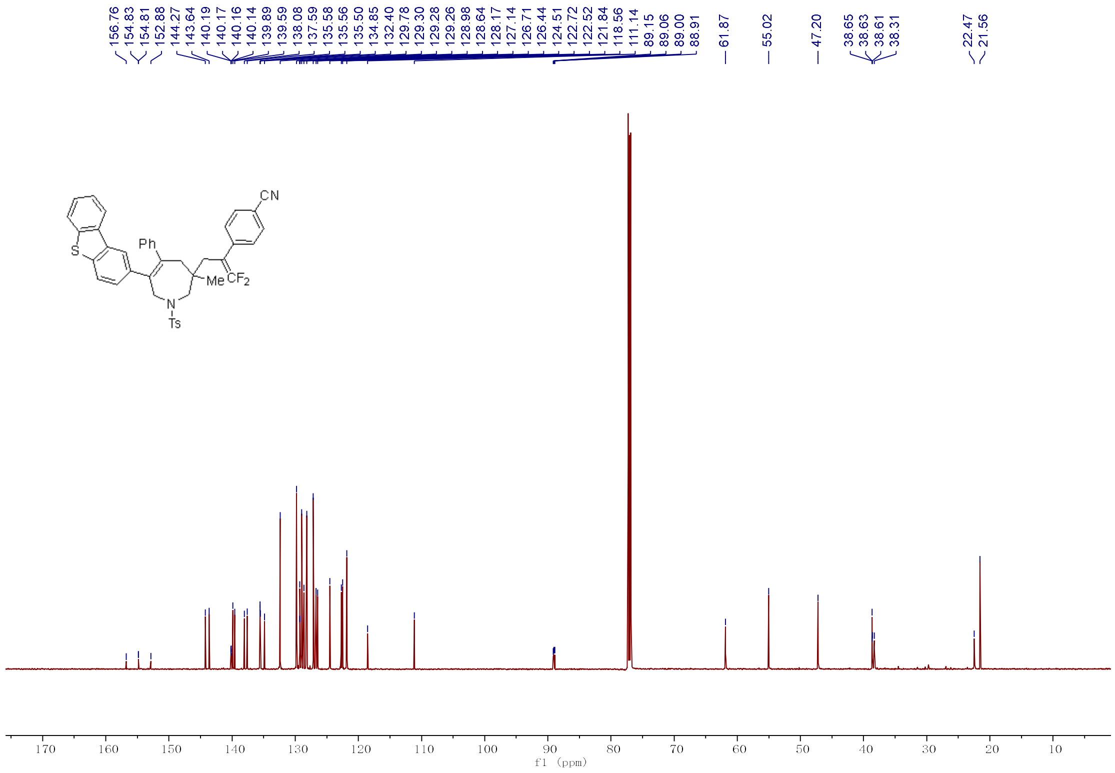

Supplementary figure 91. ^{13}C NMR of compound **27**

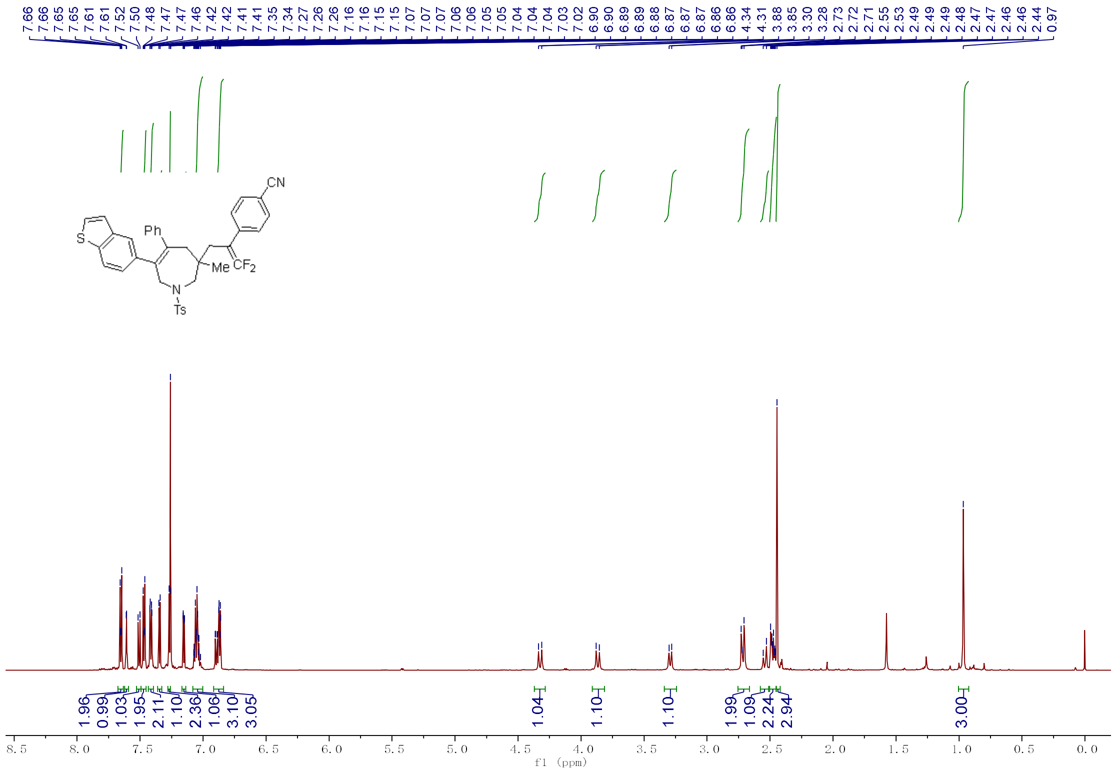

Supplementary figure 92. ^{1}H NMR of compound **28**

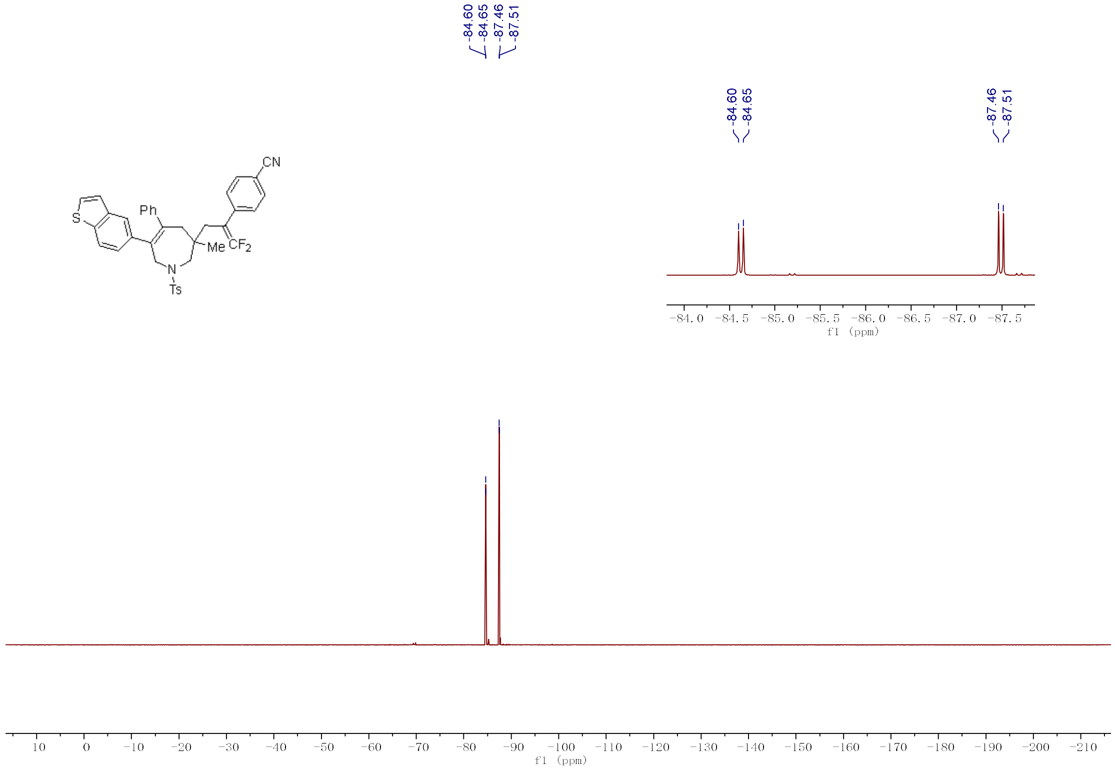

Supplementary figure 93. ^{19}F NMR of compound **28**

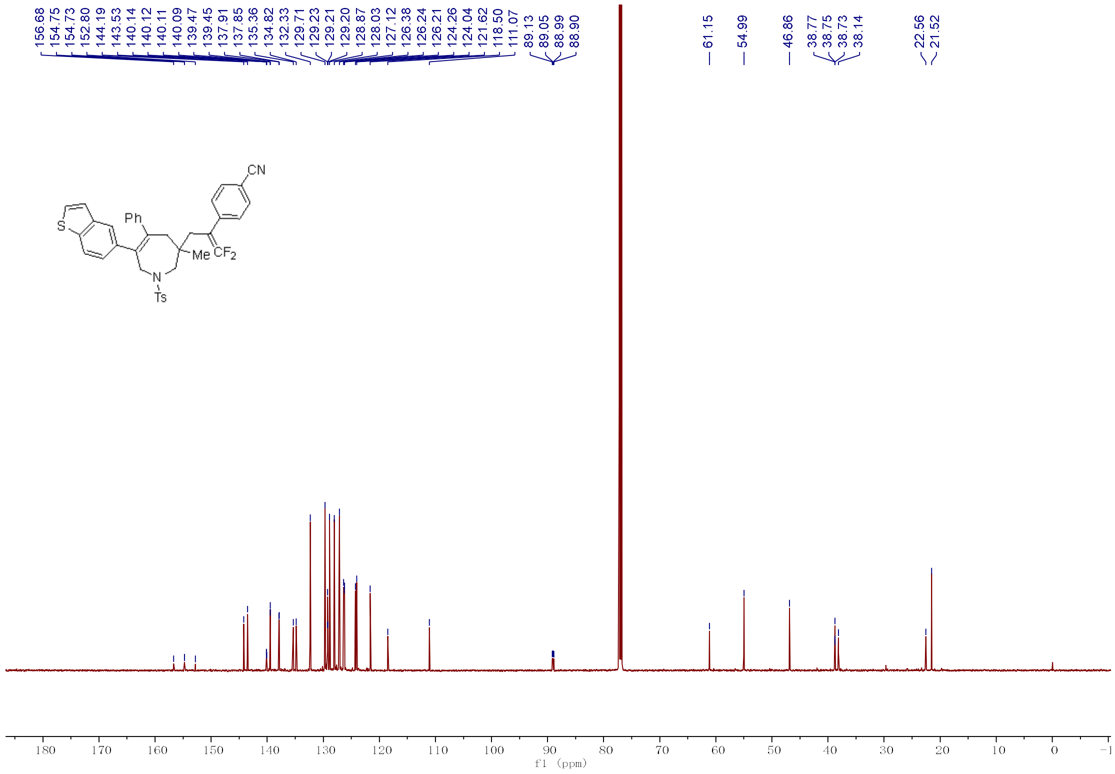

Supplementary figure 94. ^{13}C NMR of compound **28**

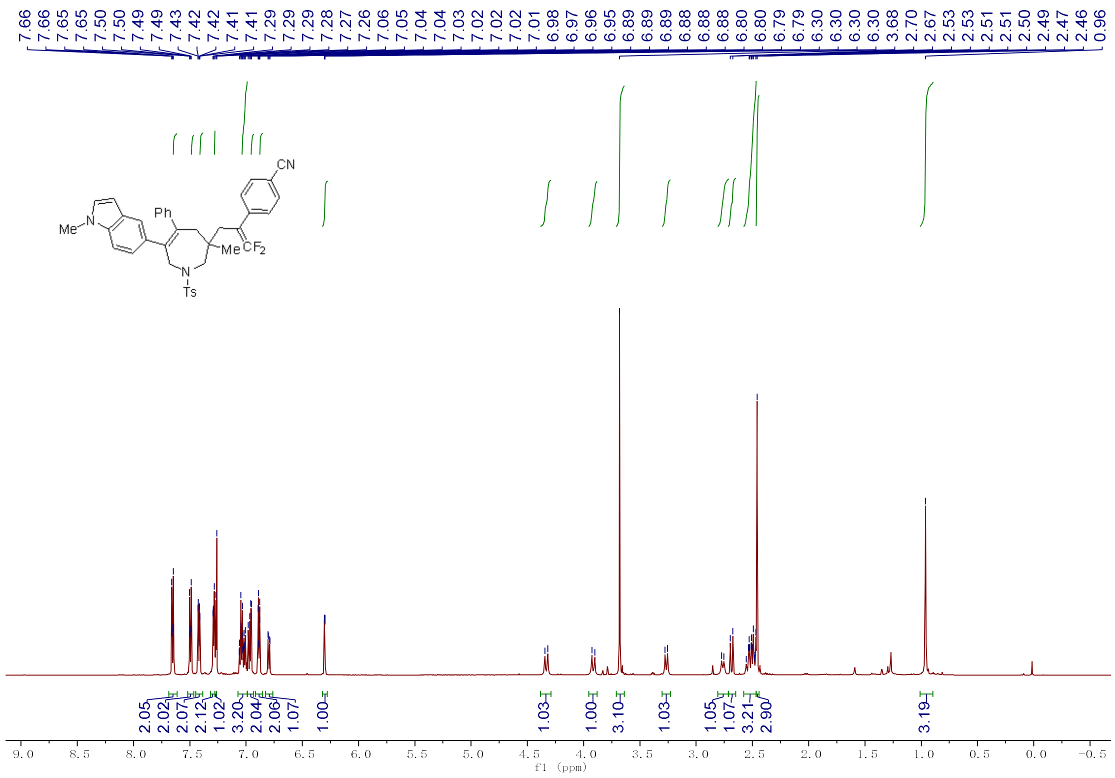

Supplementary figure 95. ^{1}H NMR of compound **29**

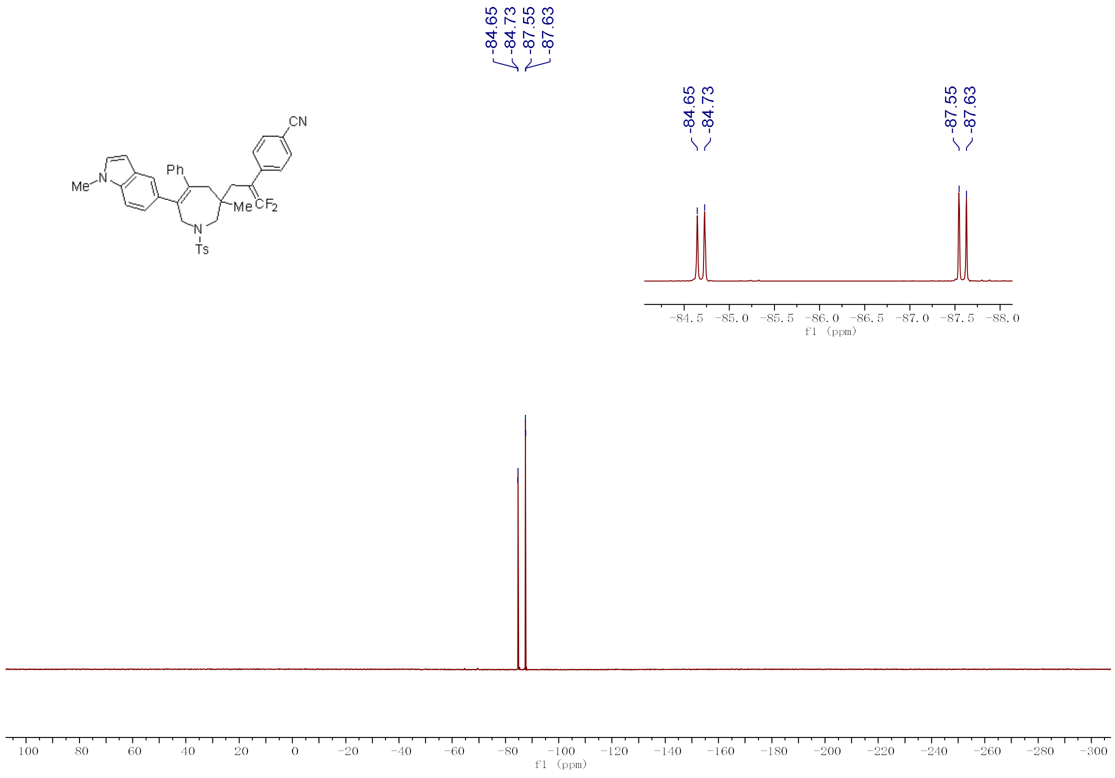

Supplementary figure 96. ^{19}F NMR of compound **29**

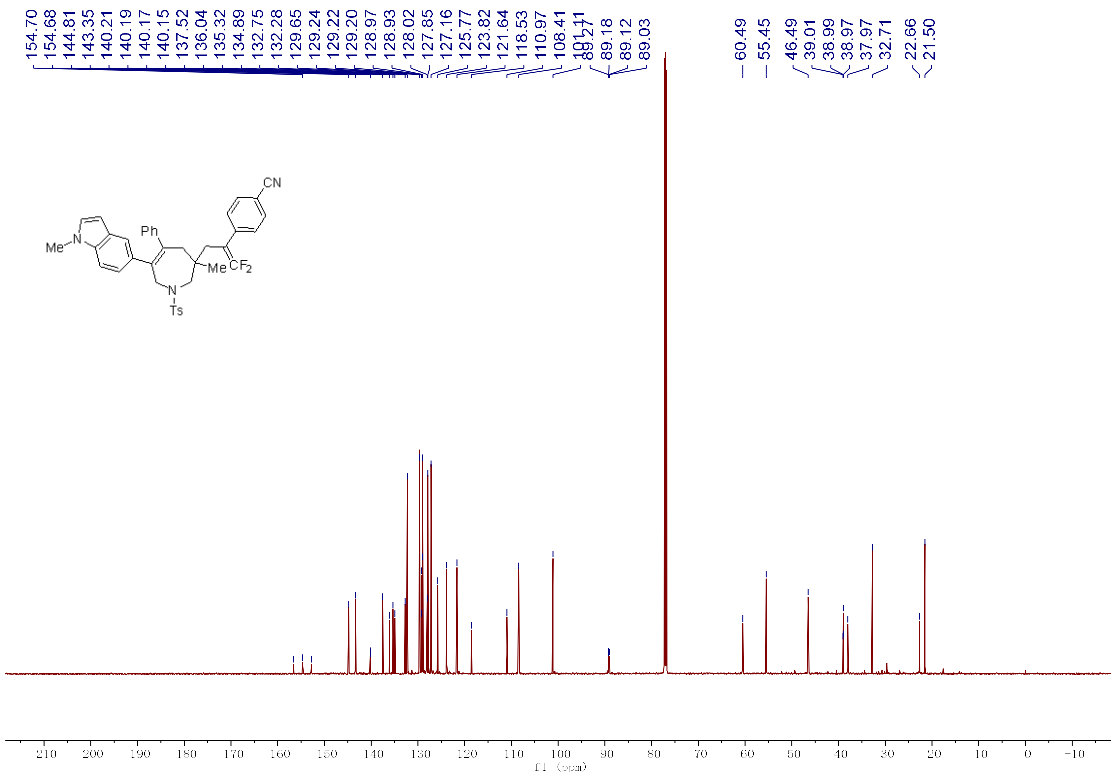

Supplementary figure 97. ^{13}C NMR of compound **29**

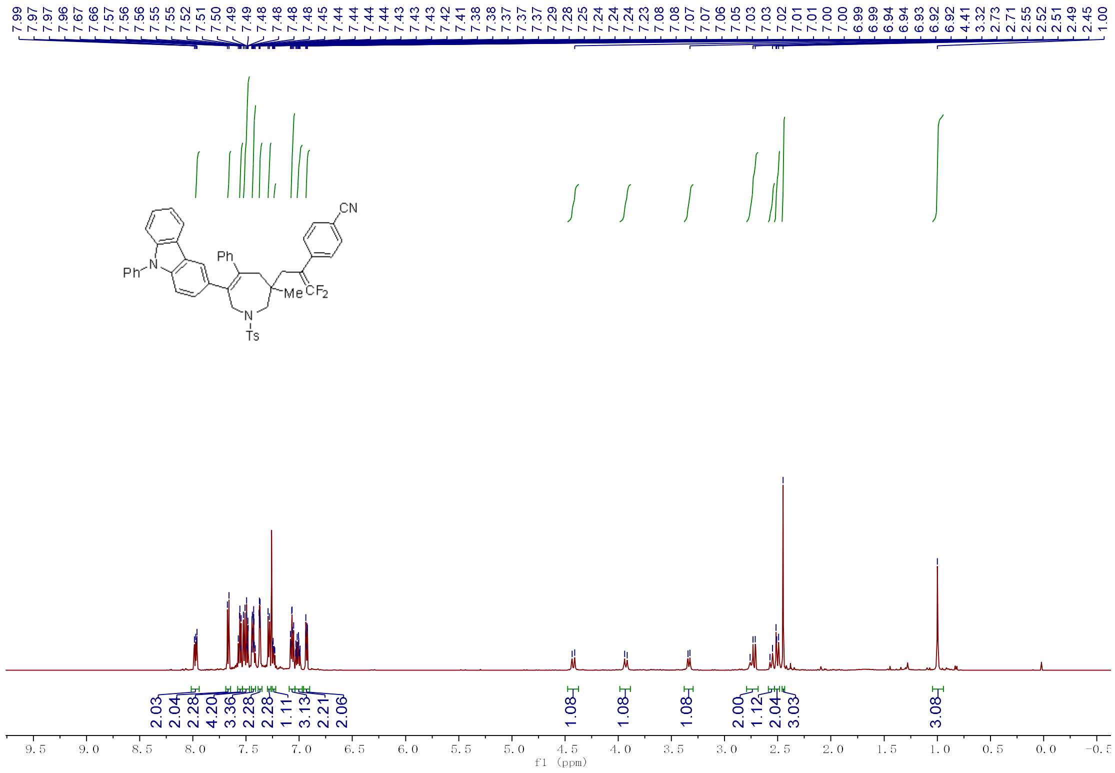

Supplementary figure 98. ^{1}H NMR of compound **30**

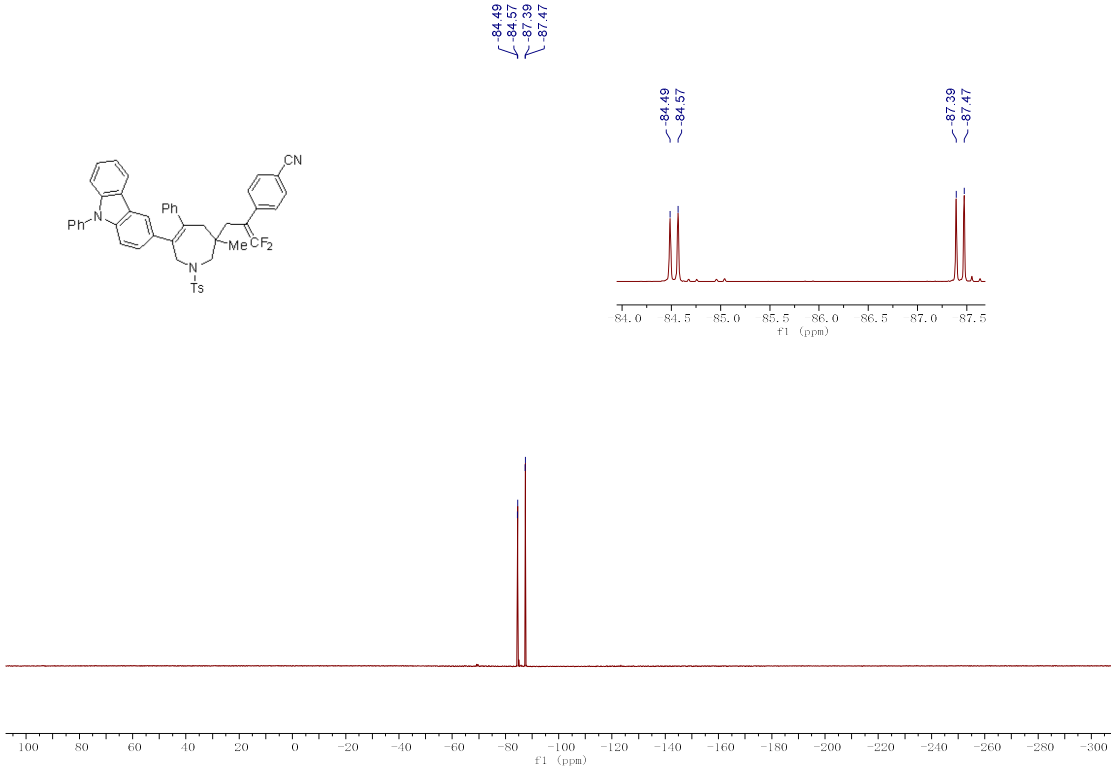

Supplementary figure 99. ^{19}F NMR of compound **30**

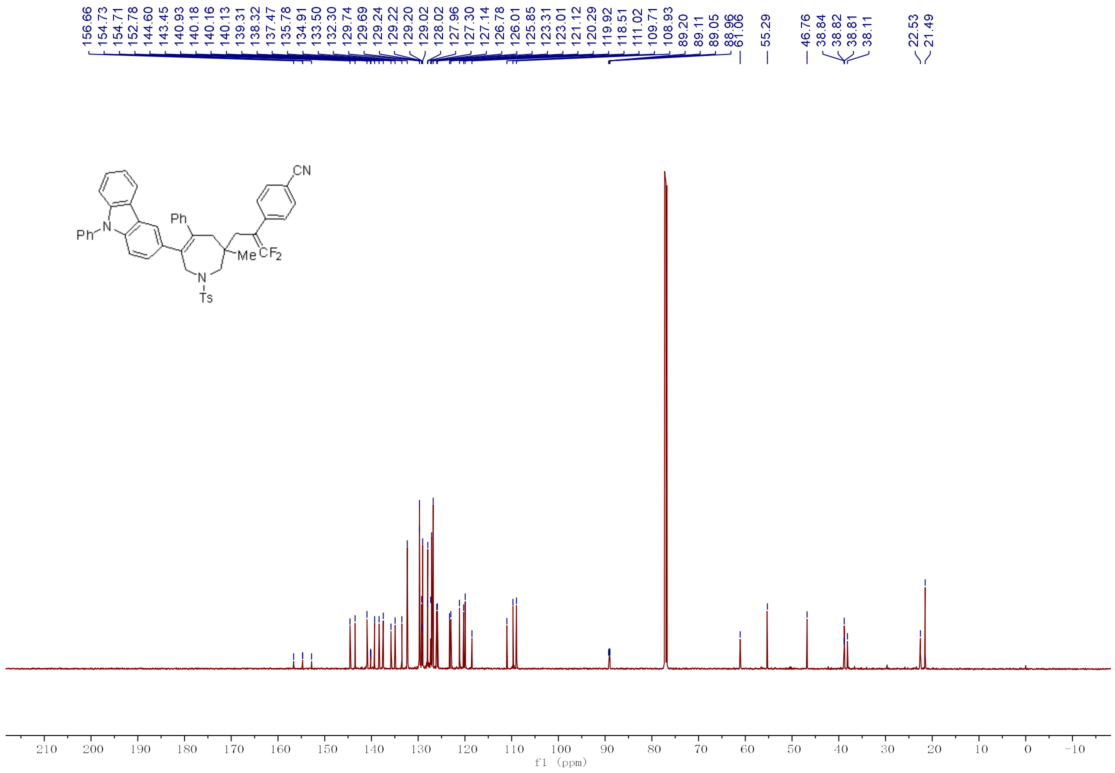

Supplementary figure 100. ^{13}C NMR of compound **30**

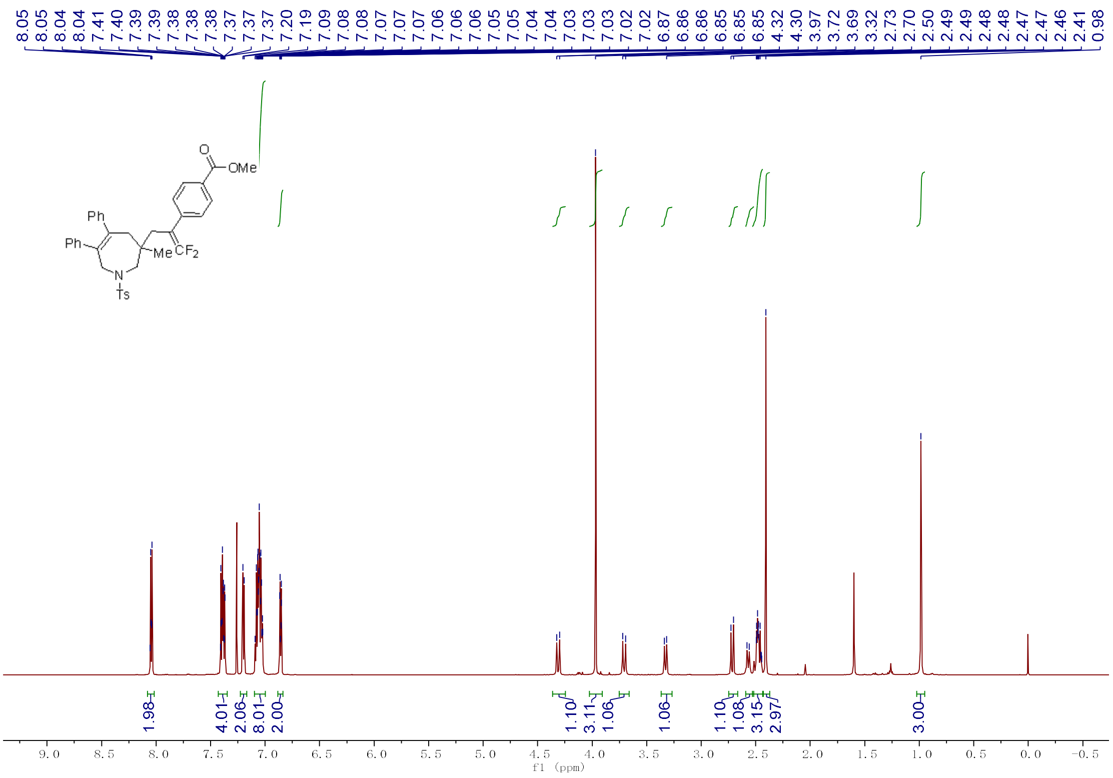

Supplementary figure 101. ^{1}H NMR of compound **31**

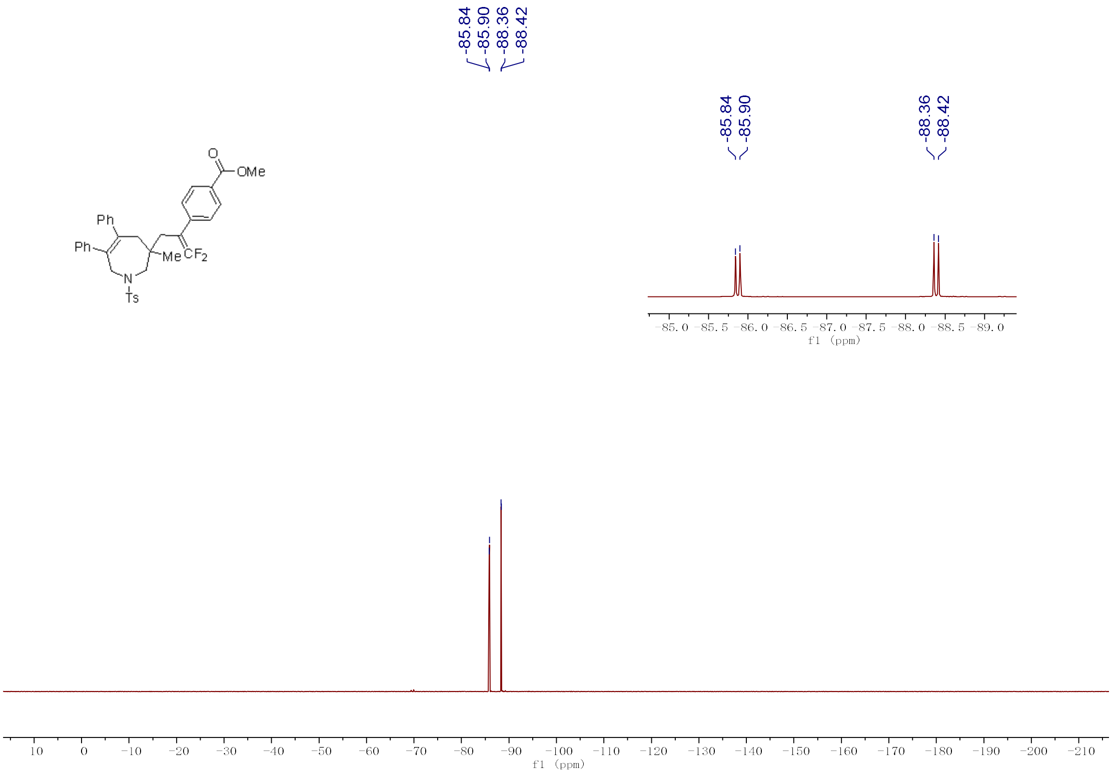

Supplementary figure 102. ^{19}F NMR of compound **31**

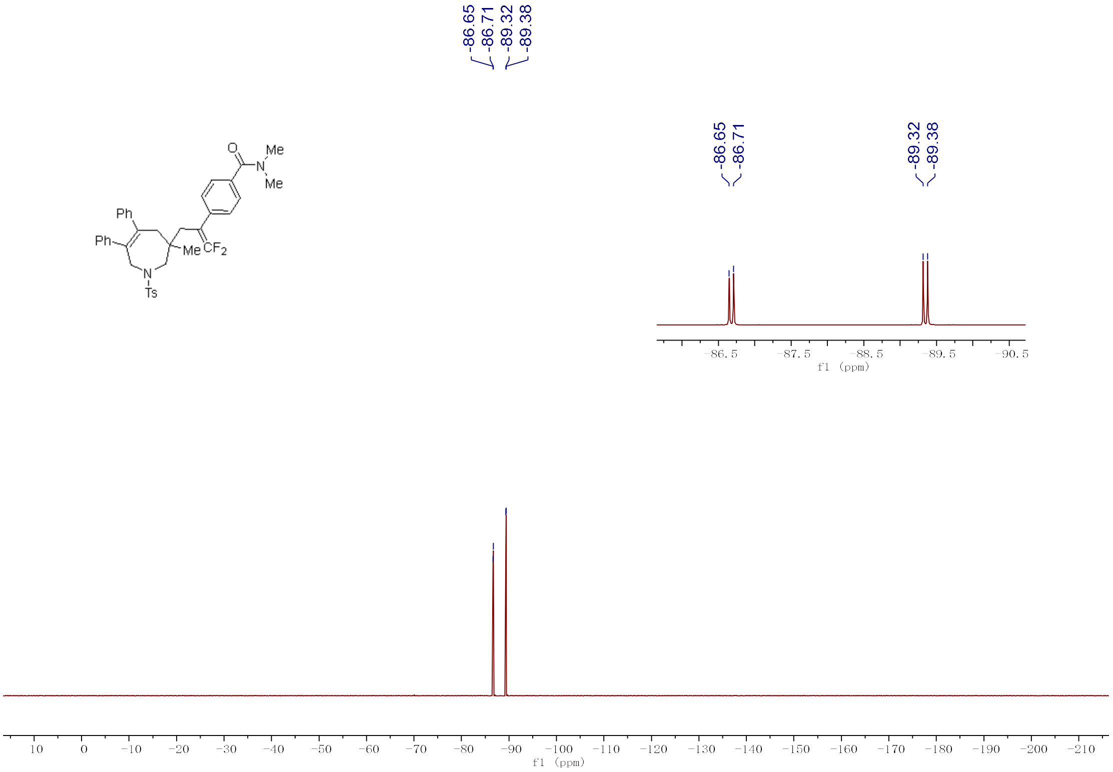

Supplementary figure 103. ^{13}C NMR of compound **31**

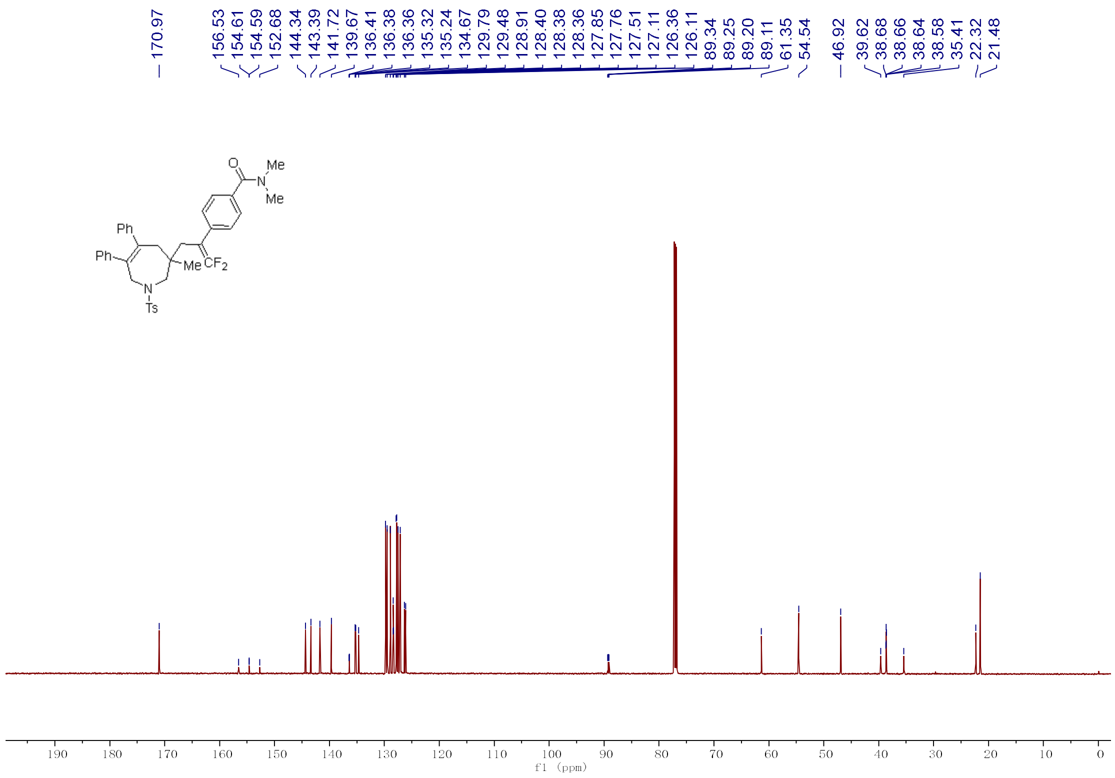

Supplementary figure 104. ^{1}H NMR of compound **32**

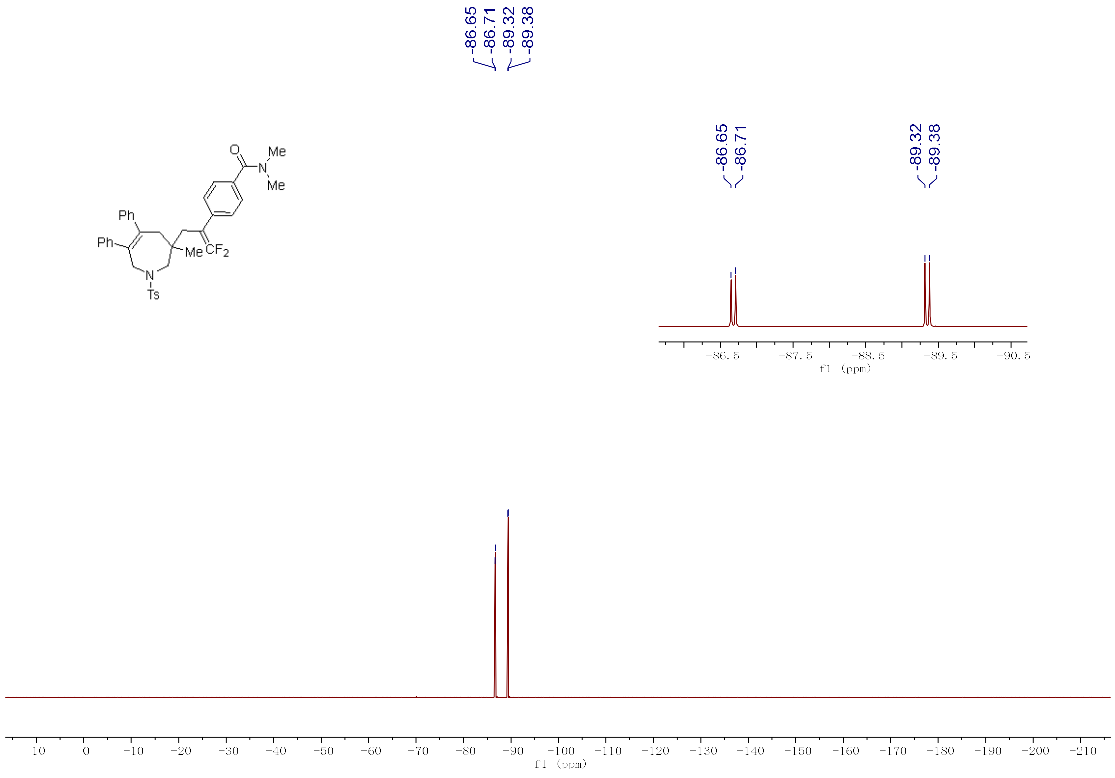

Supplementary figure 105. ^{19}F NMR of compound **32**

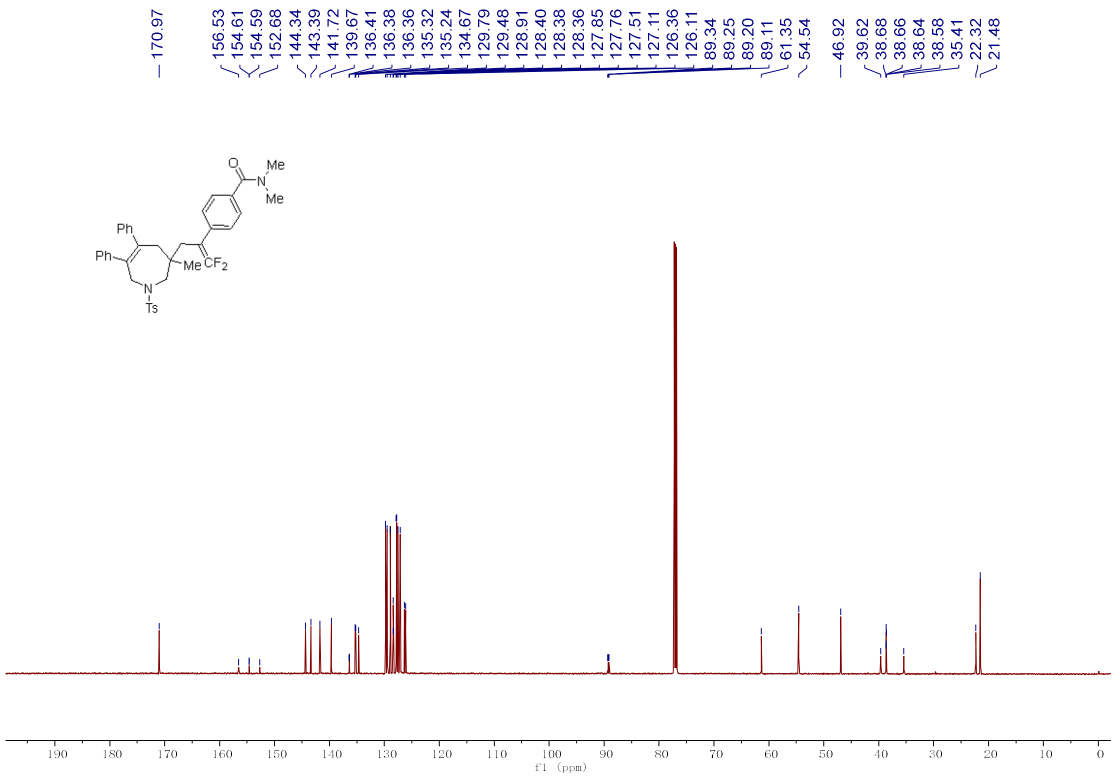

Supplementary figure 106. ^{13}C NMR of compound **32**

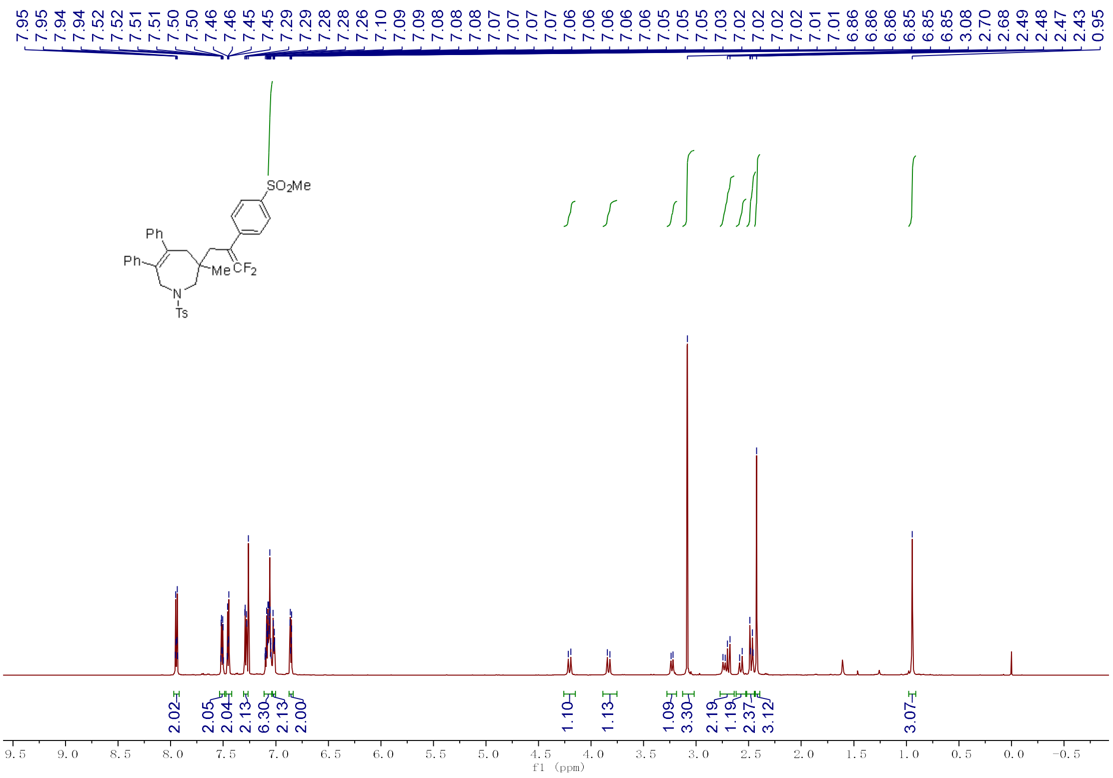

Supplementary figure 107. ^{1}H NMR of compound **33**

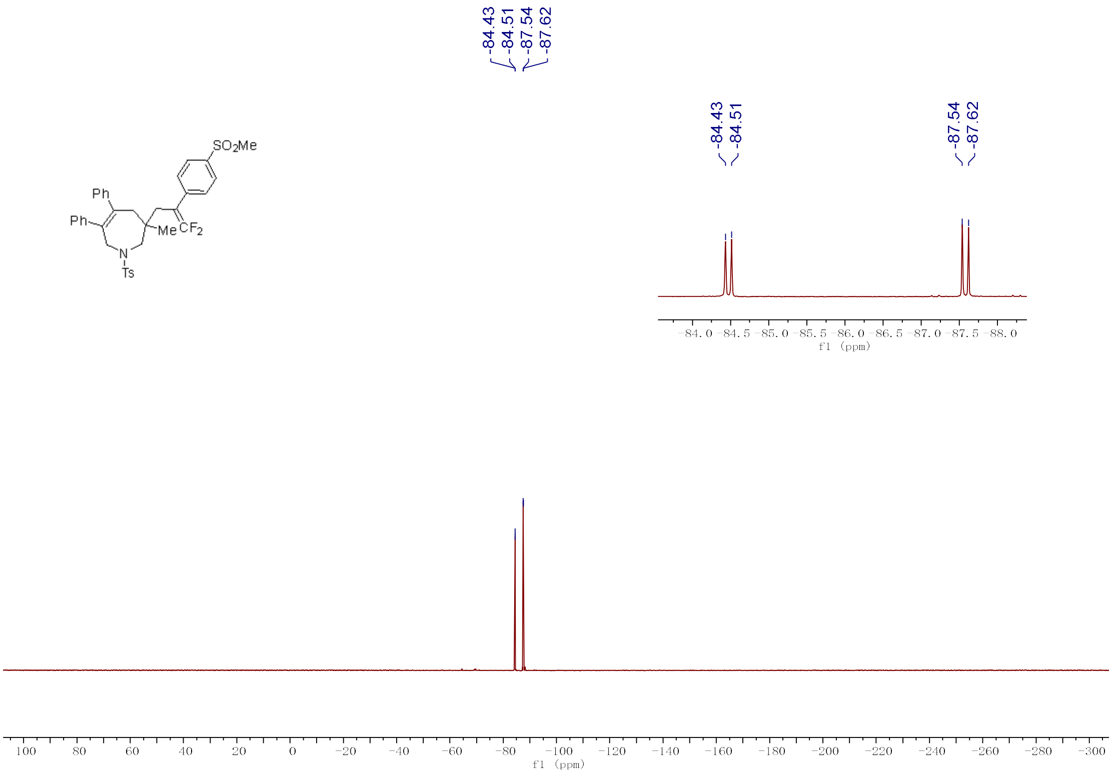

Supplementary figure 108. ^{19}F NMR of compound **33**

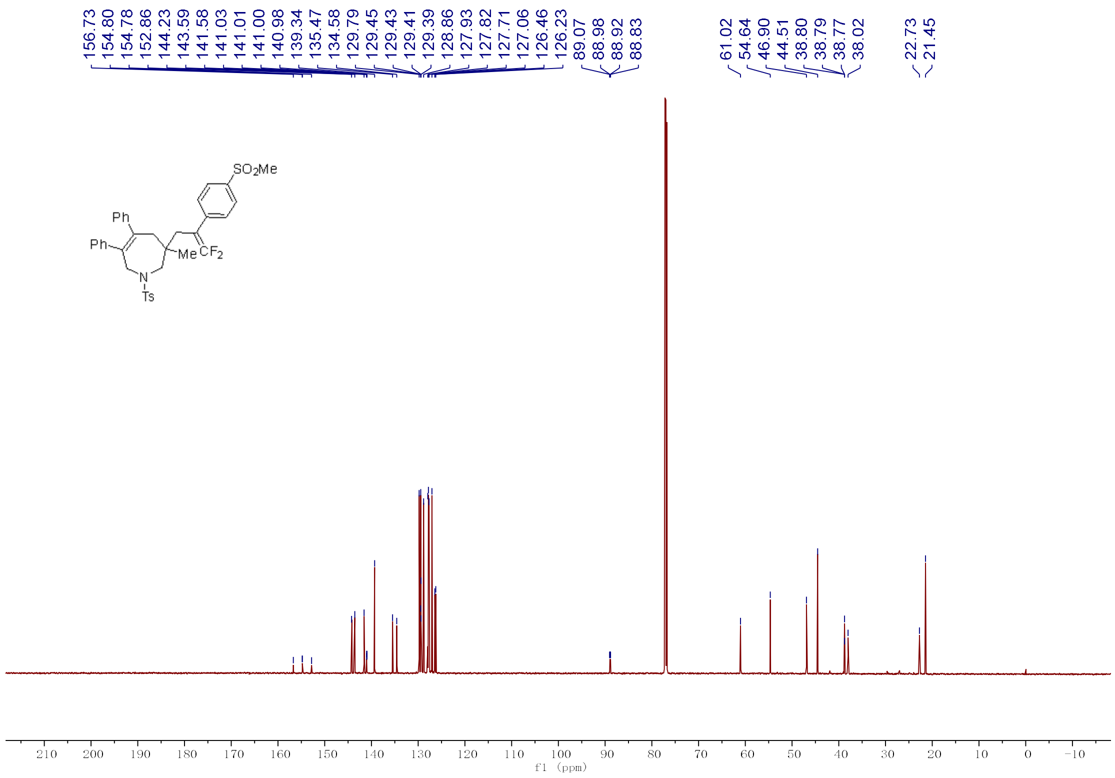

Supplementary figure 109. ^{13}C NMR of compound **33**

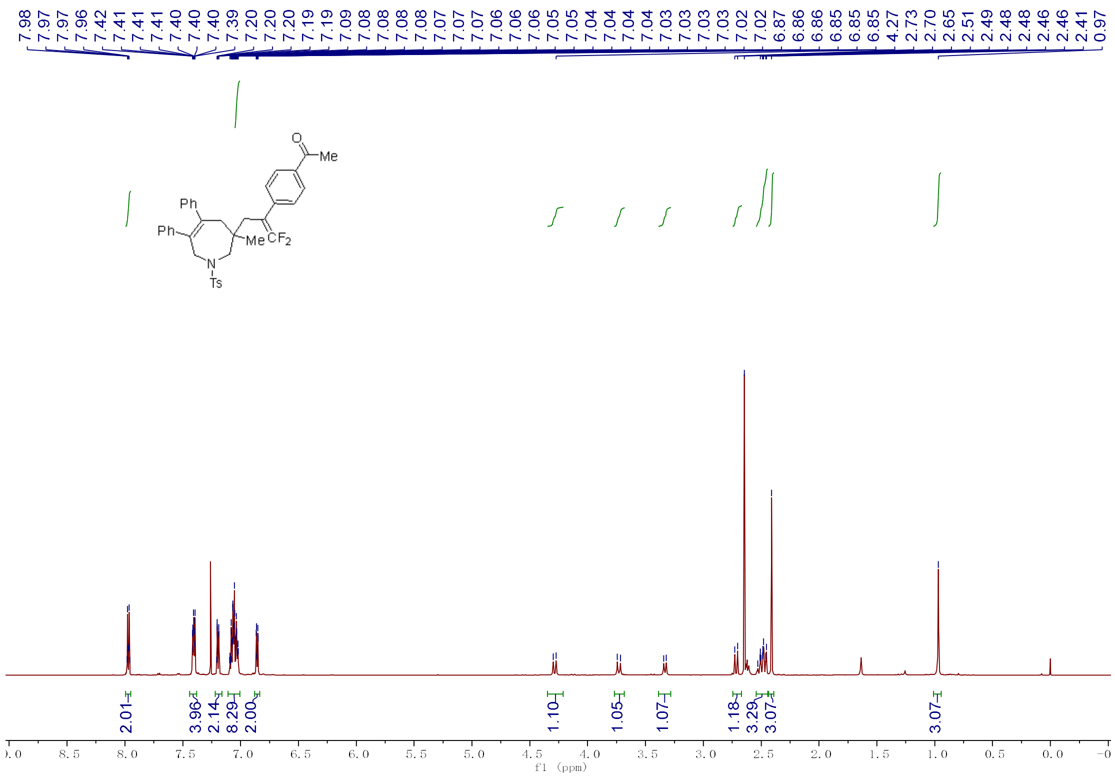

Supplementary figure 110. ^{1}H NMR of compound **34**

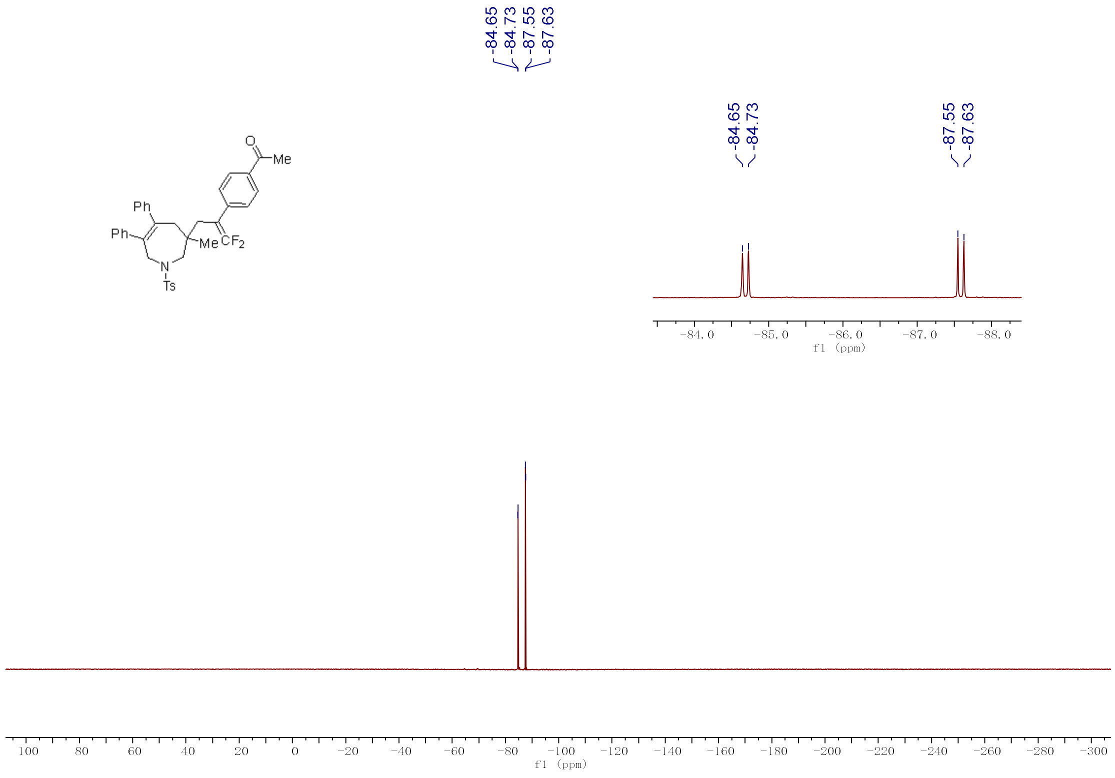

Supplementary figure 111. ^{19}F NMR of compound **34**

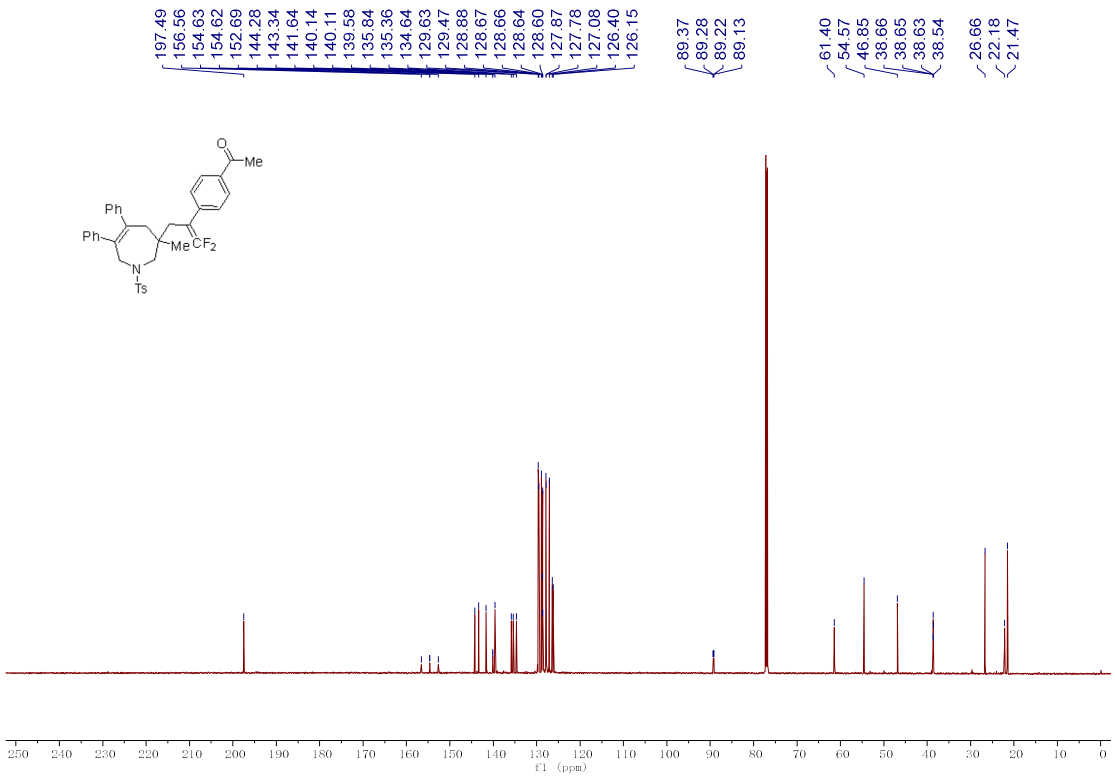

Supplementary figure 112. ^{13}C NMR of compound **34**

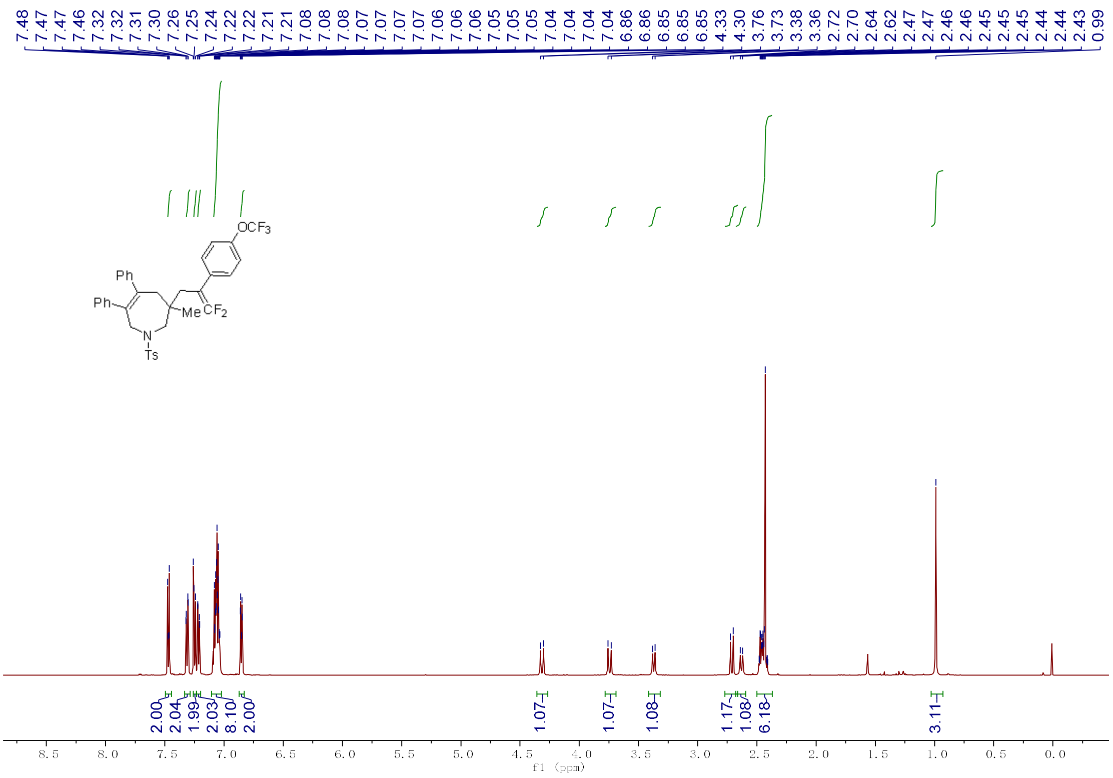

Supplementary figure 113. ^{1}H NMR of compound **35**

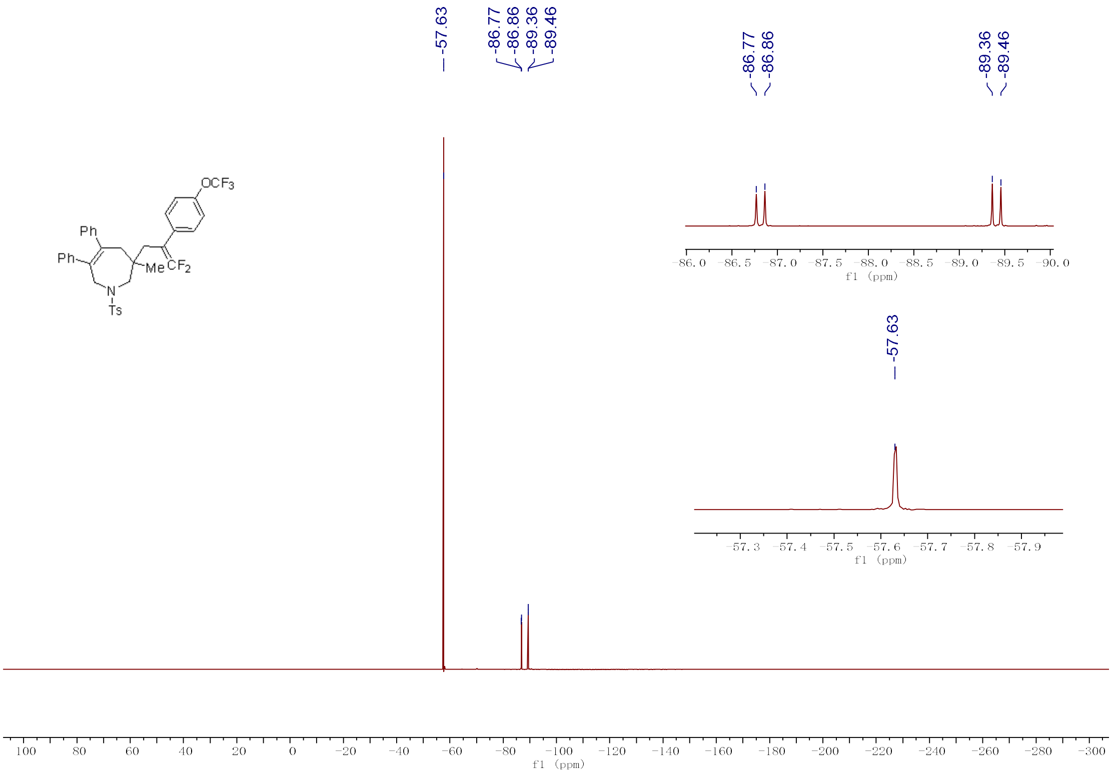

Supplementary figure 114. ^{19}F NMR of compound **35**

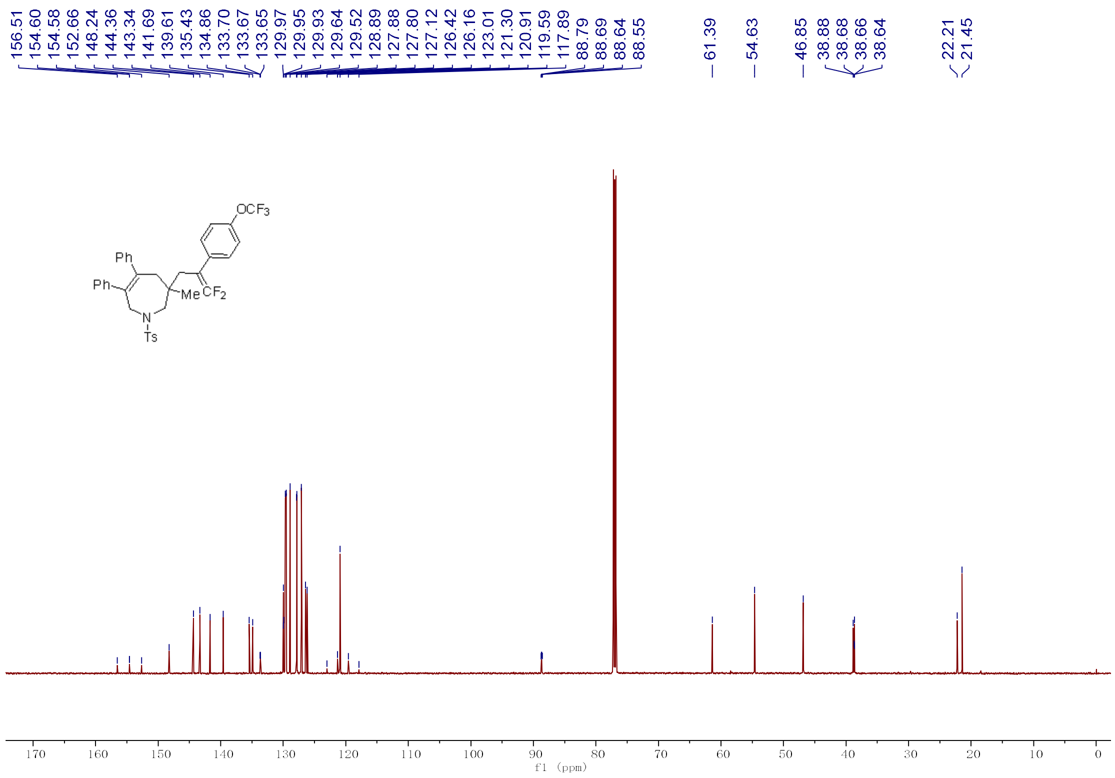

Supplementary figure 115. ^{13}C NMR of compound **35**

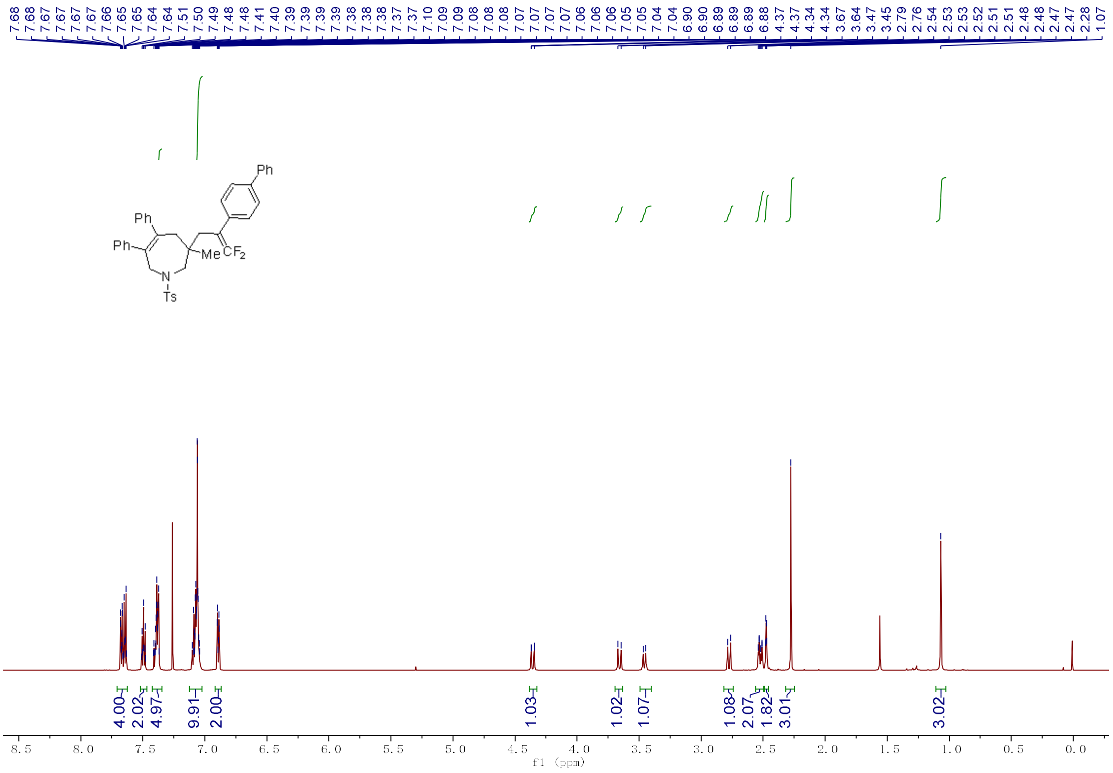

Supplementary figure 116. ^{1}H NMR of compound **36**

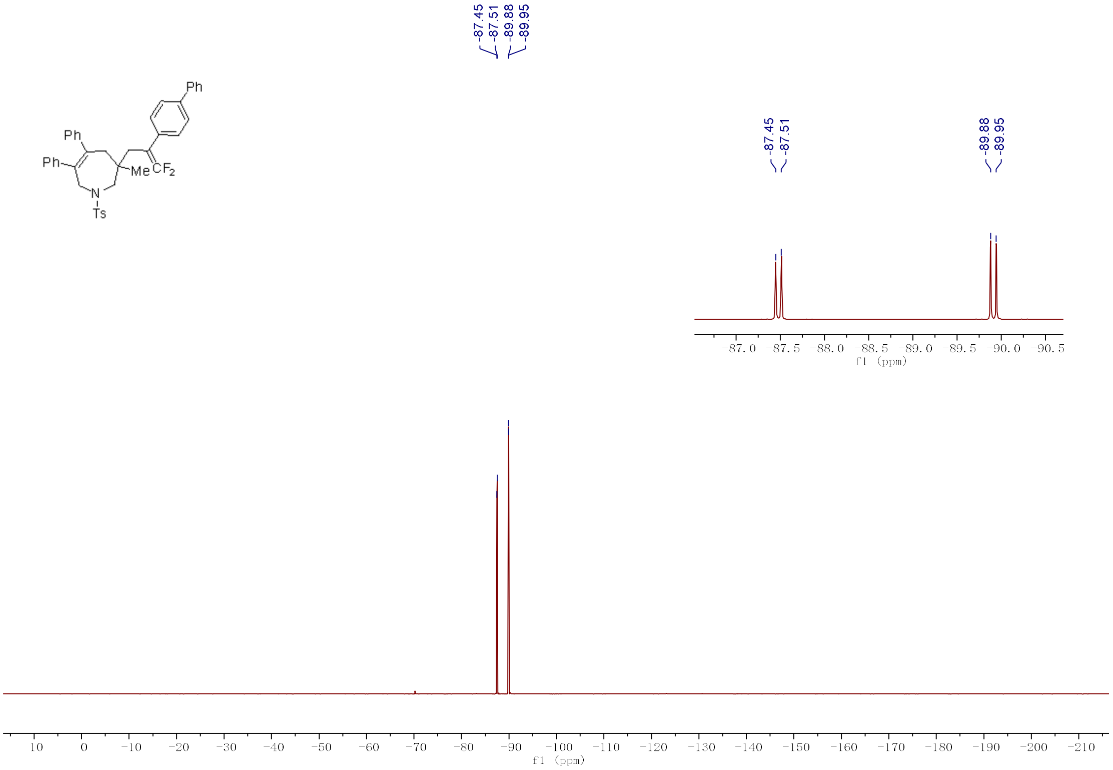

Supplementary figure 117. ^{19}F NMR of compound **36**

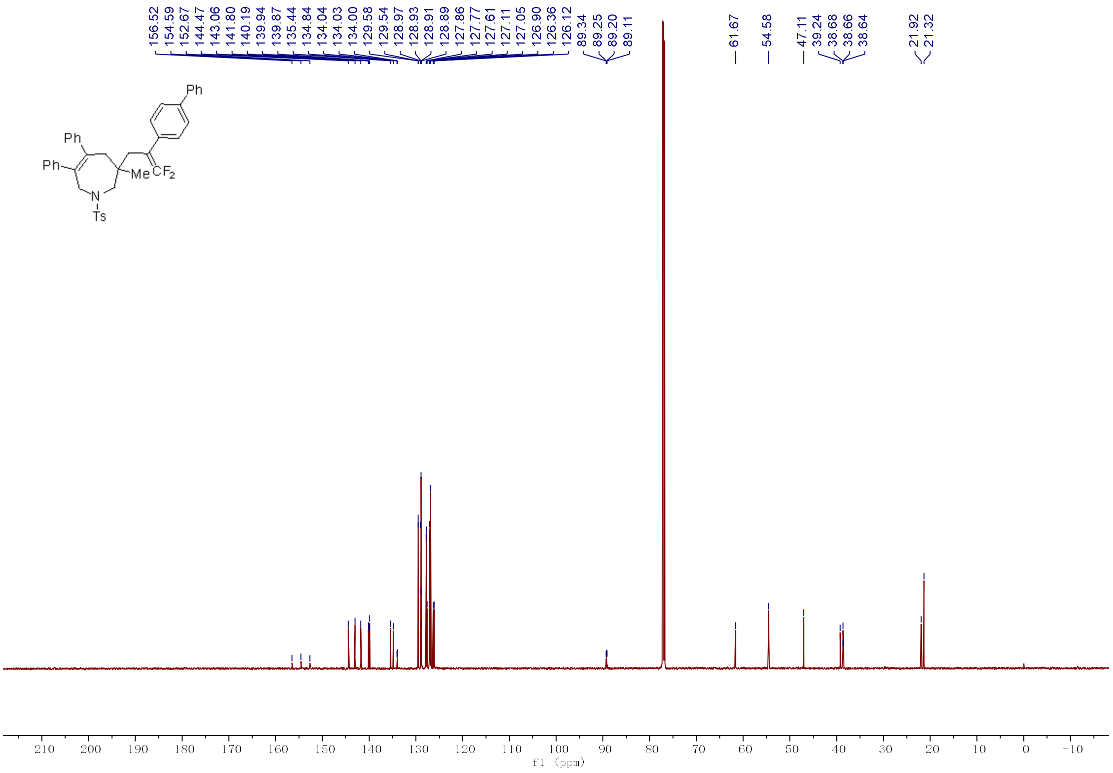

Supplementary figure 118. ^{13}C NMR of compound **36**

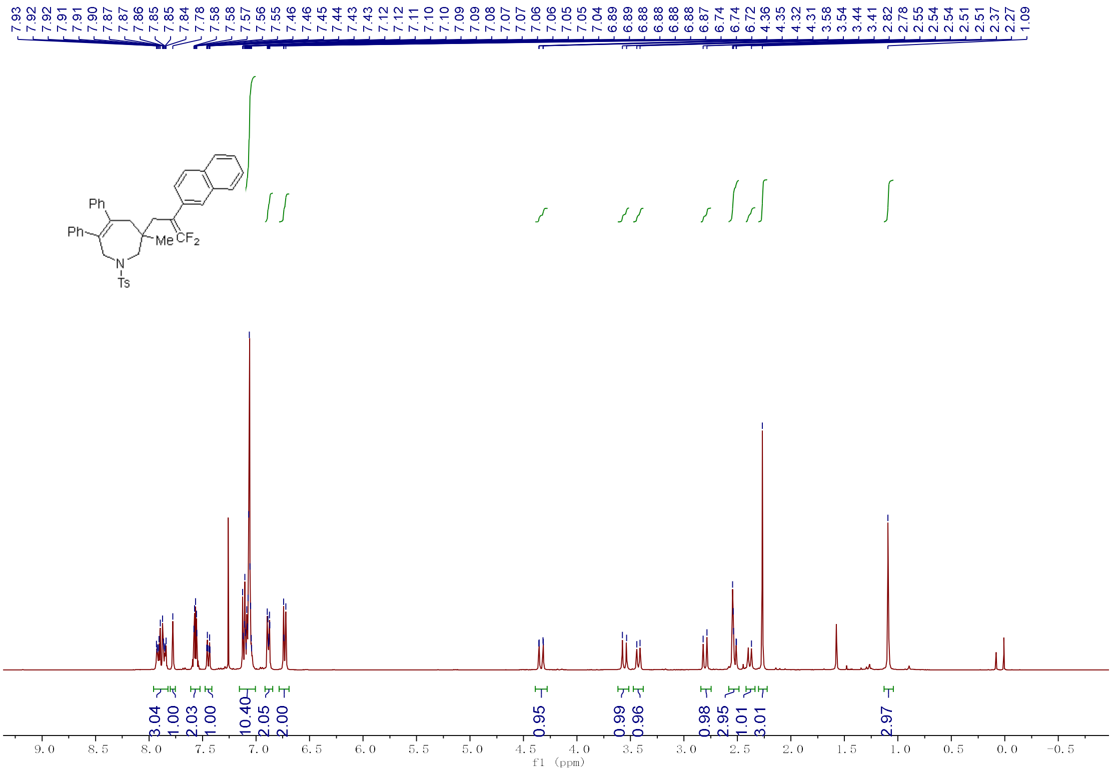

Supplementary figure 119. ^{1}H NMR of compound **37**

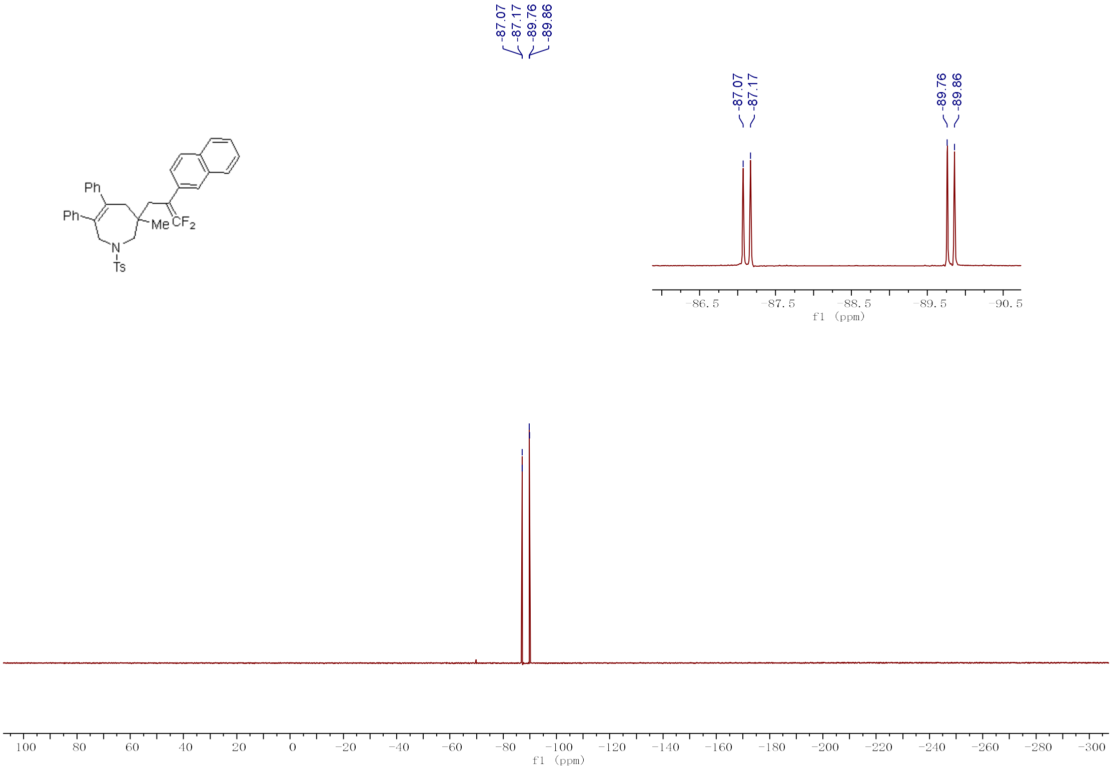

Supplementary figure 120. ^{19}F NMR of compound **37**

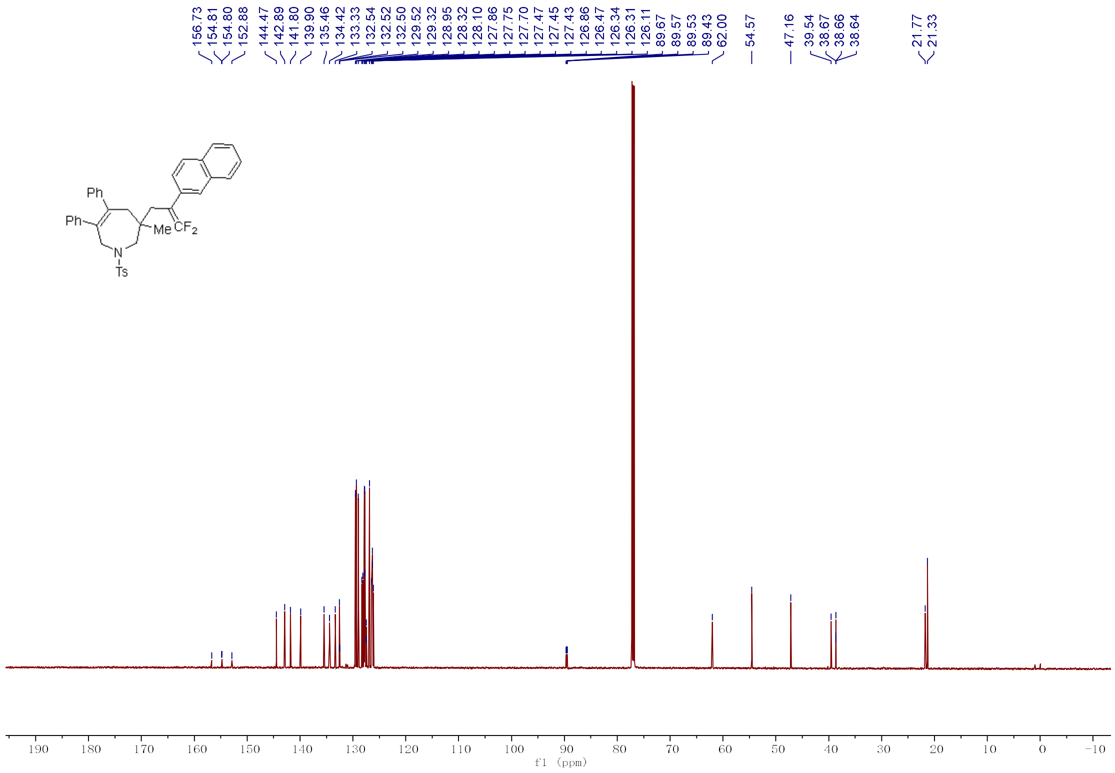

Supplementary figure 121. ^{13}C NMR of compound **37**

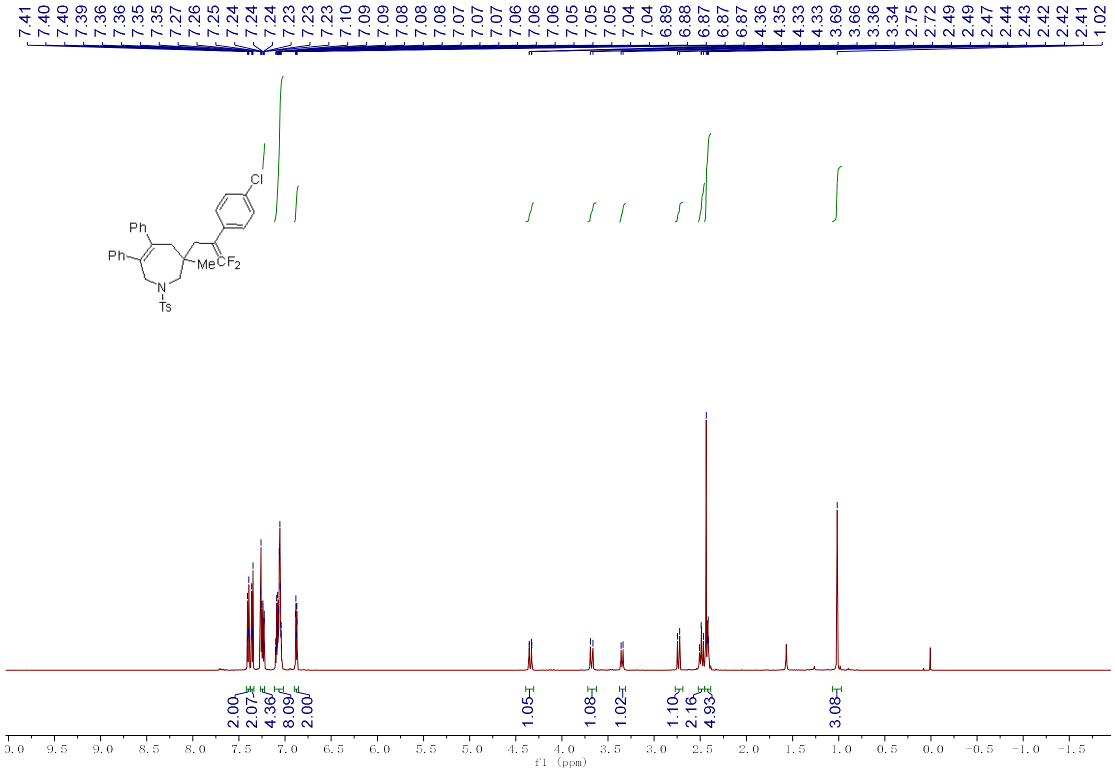

Supplementary figure 122. ^{1}H NMR of compound **38**

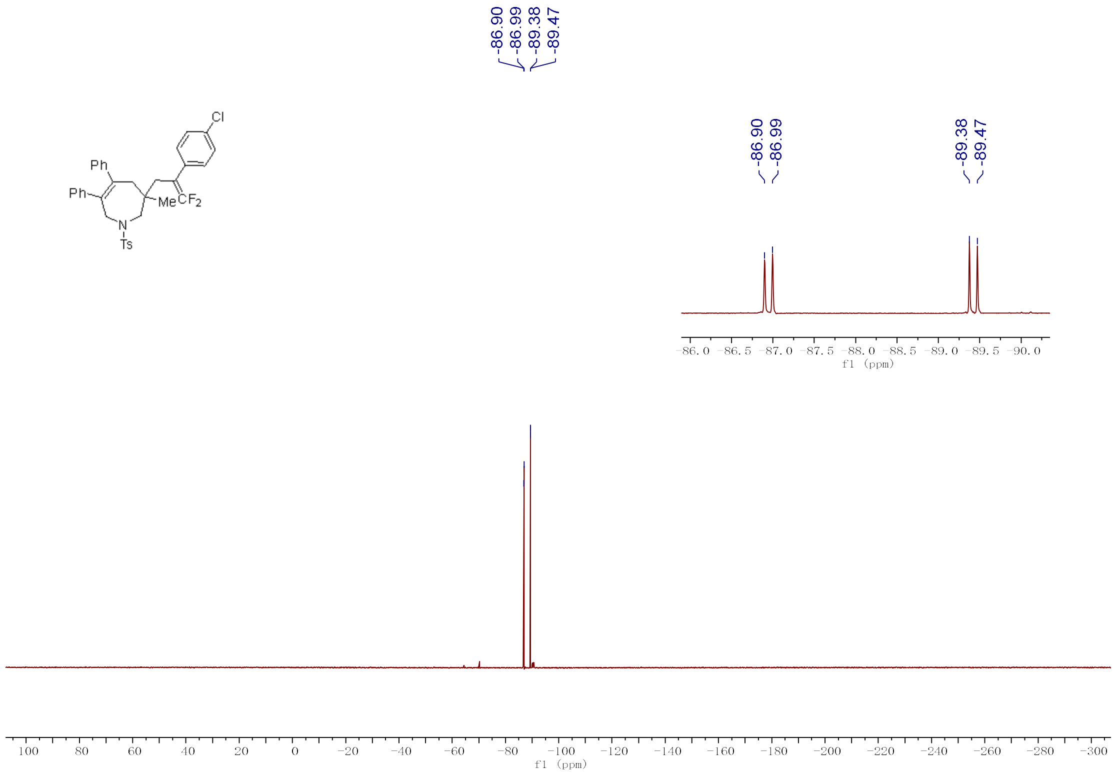

Supplementary figure 123. ^{19}F NMR of compound **38**

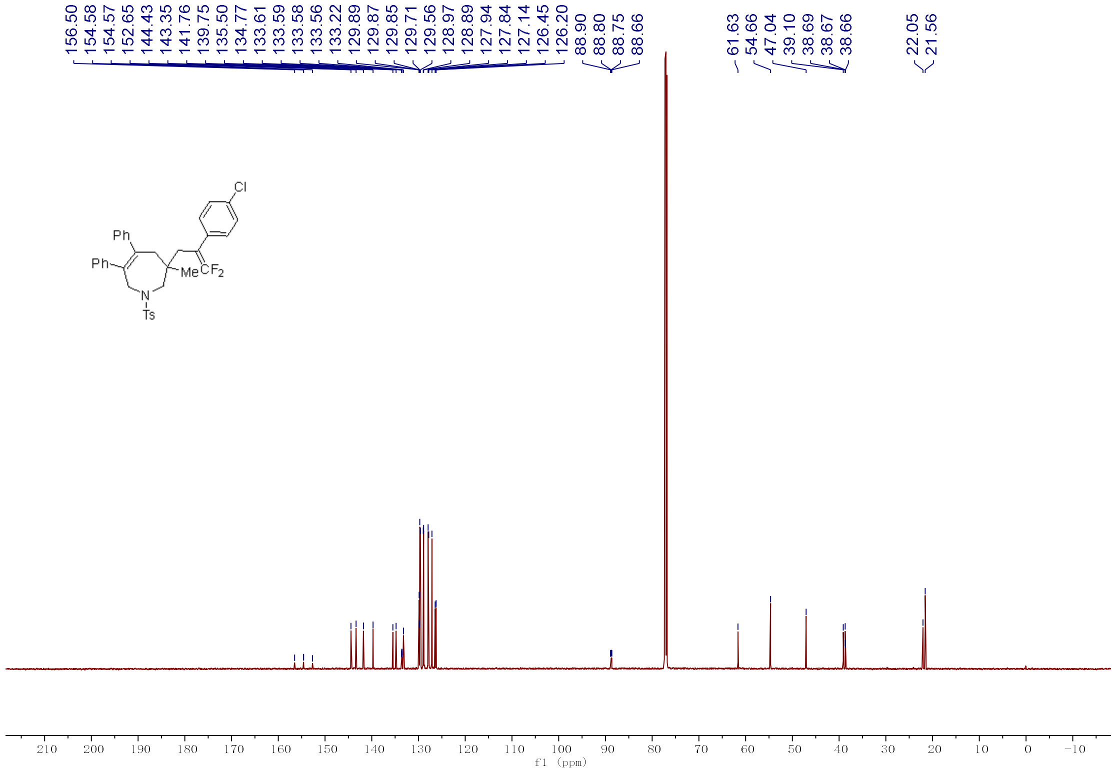

Supplementary figure 124. ^{13}C NMR of compound **38**

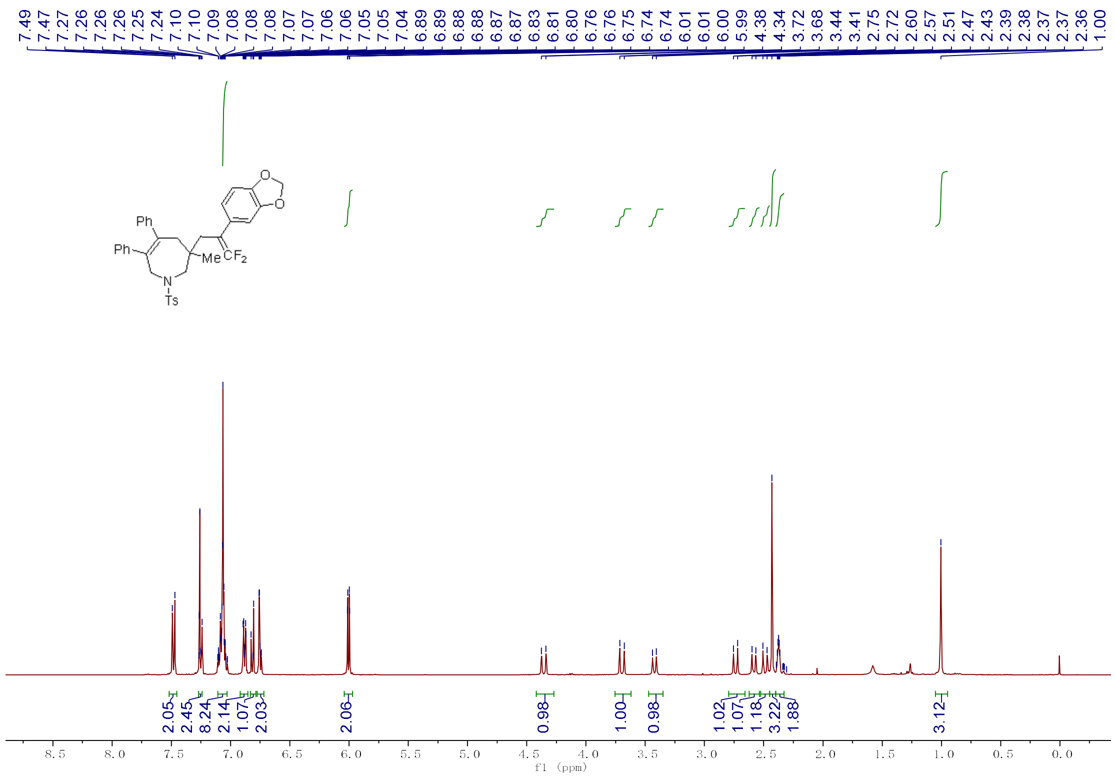

Supplementary figure 125. ^{1}H NMR of compound **39**

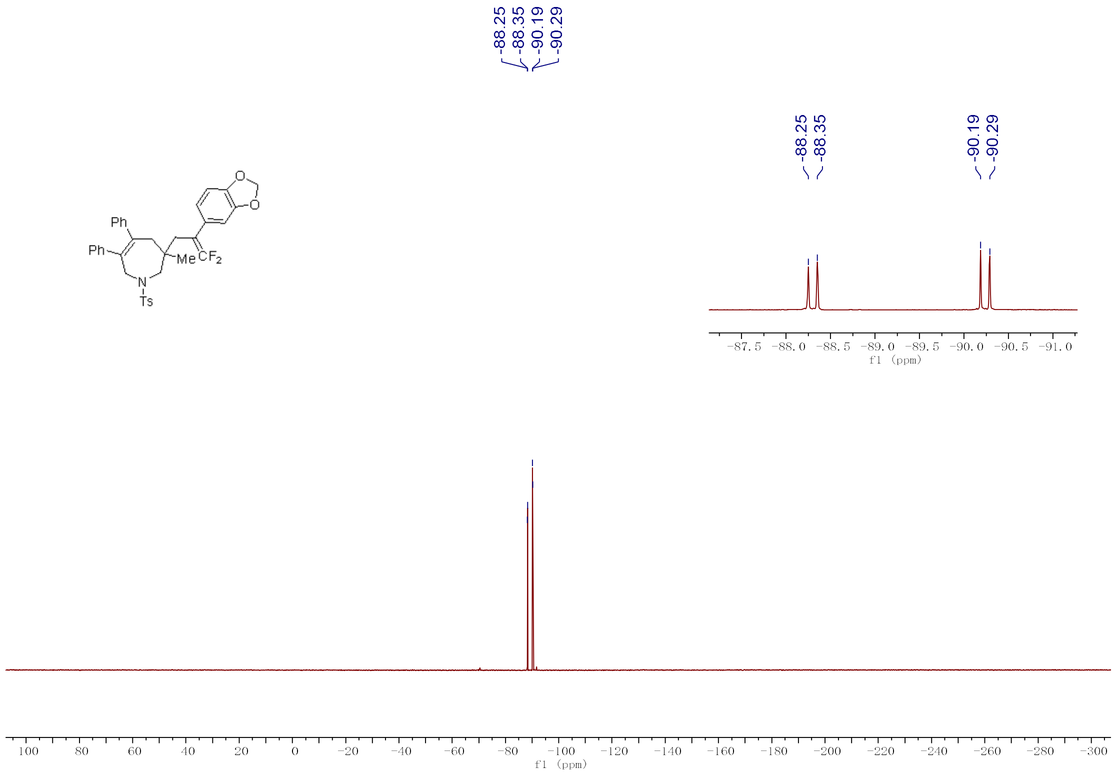

Supplementary figure 126. ^{19}F NMR of compound **39**

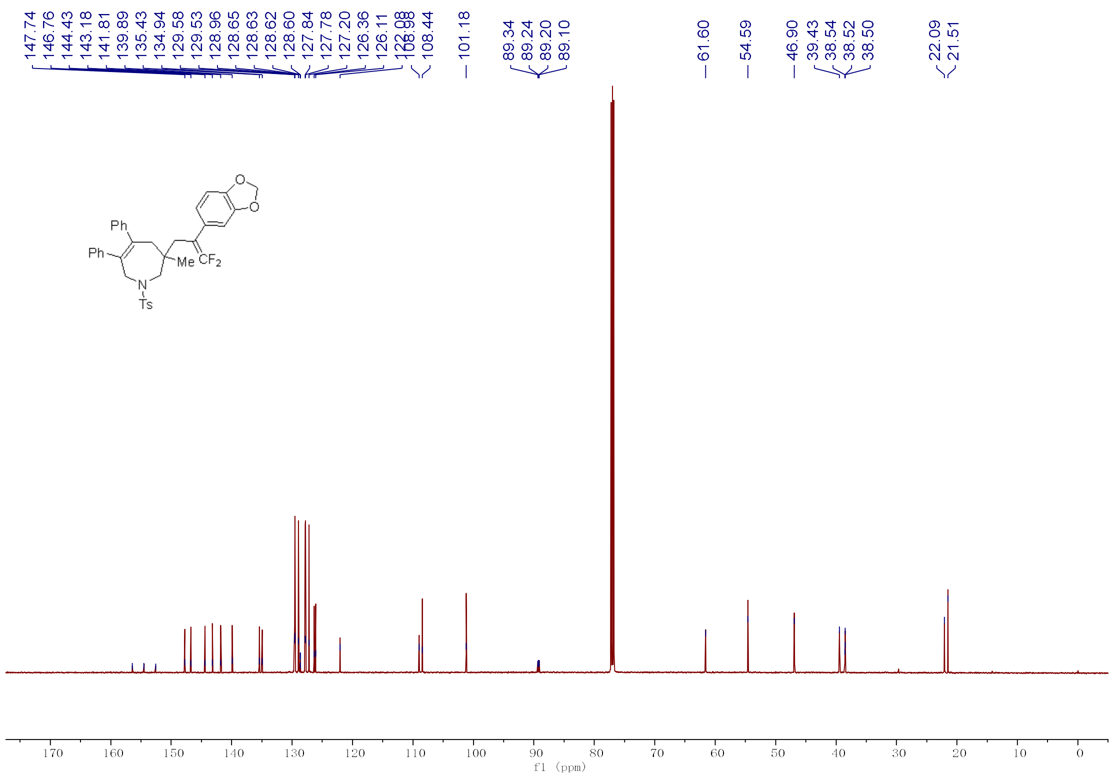

Supplementary figure 127. ^{13}C NMR of compound **39**

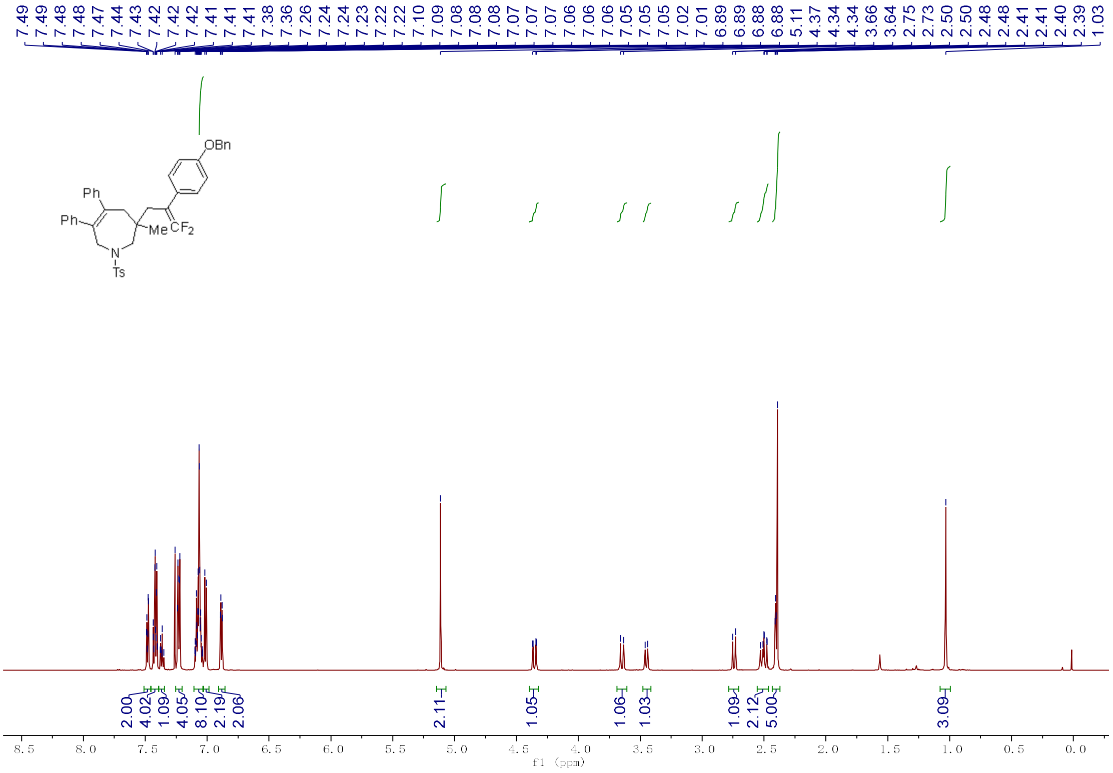

Supplementary figure 128. ^{1}H NMR of compound **40**

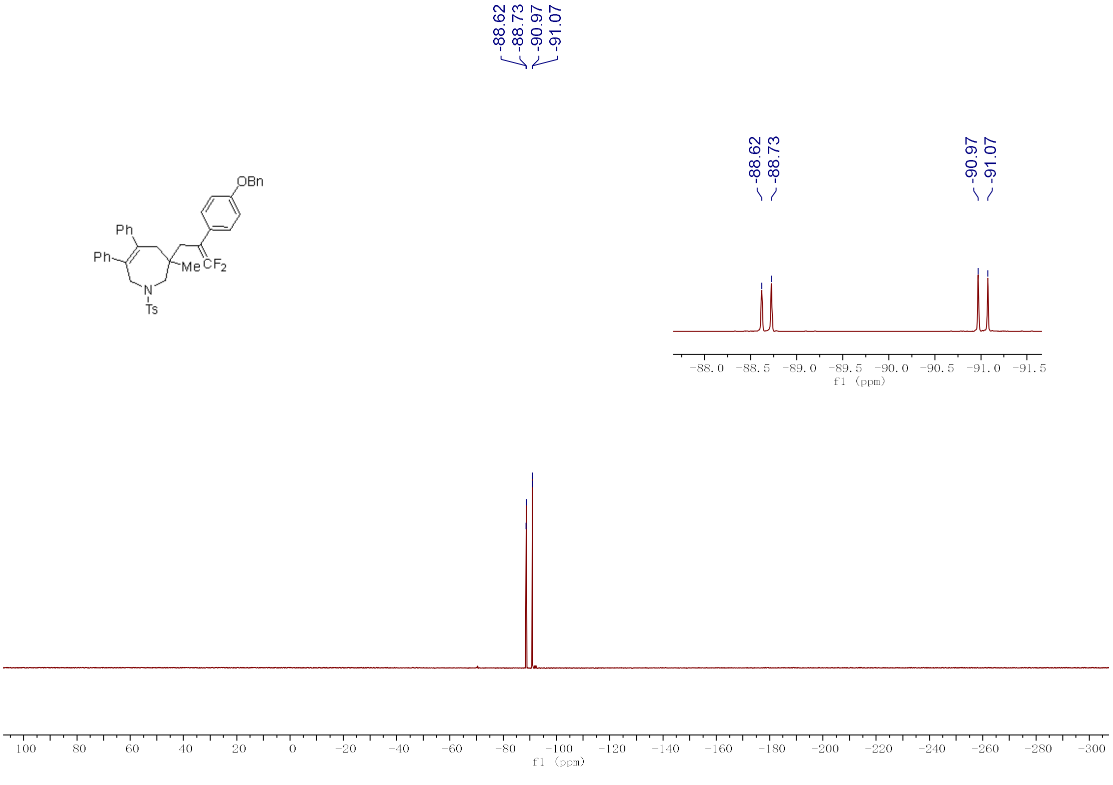

Supplementary figure 129. ^{19}F NMR of compound **40**

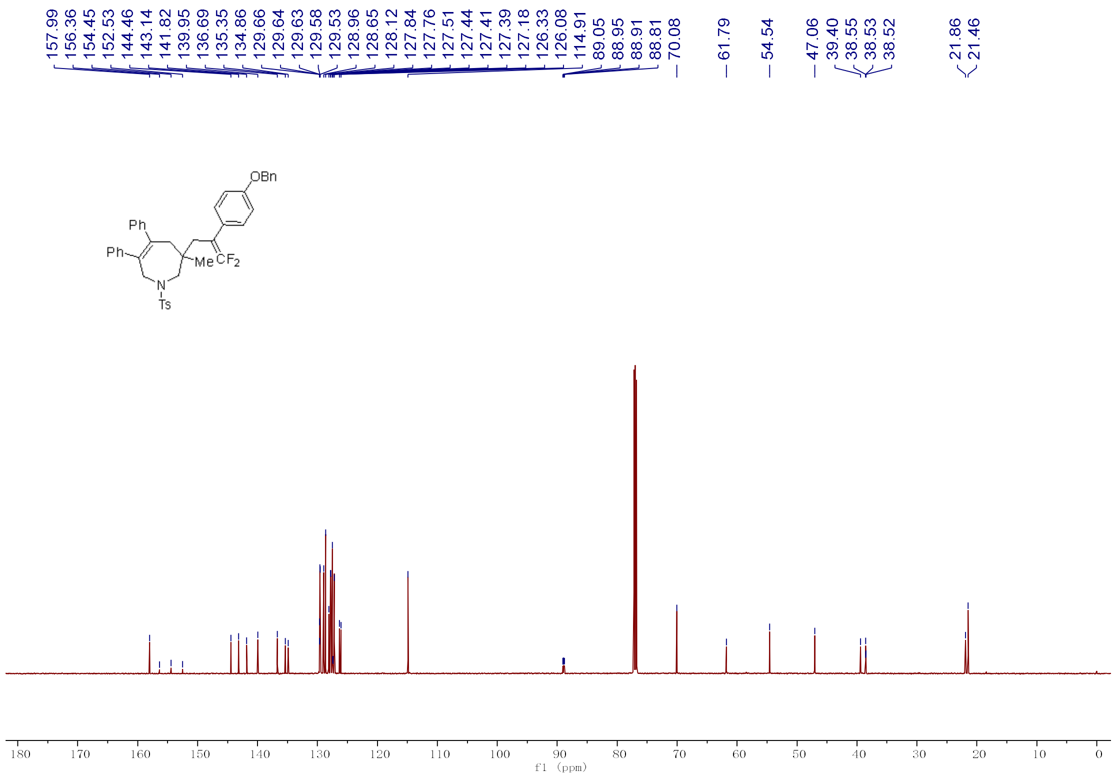

Supplementary figure 130. ^{13}C NMR of compound **40**

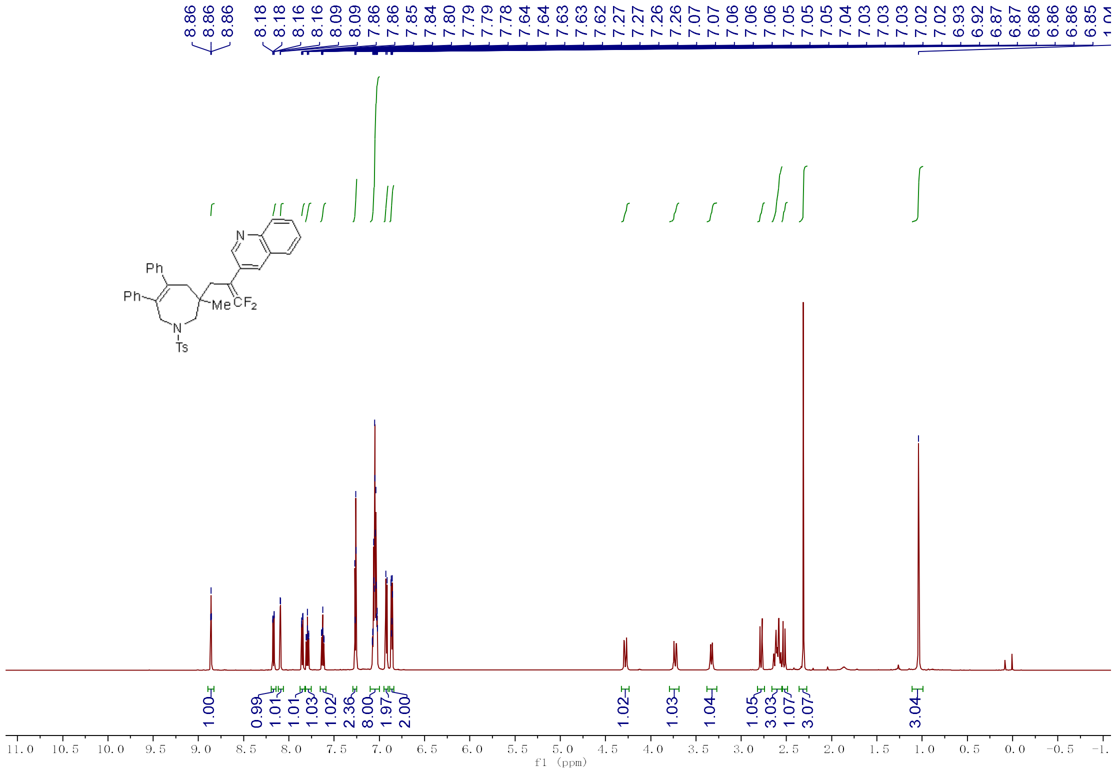

Supplementary figure 131. ^{1}H NMR of compound **41**

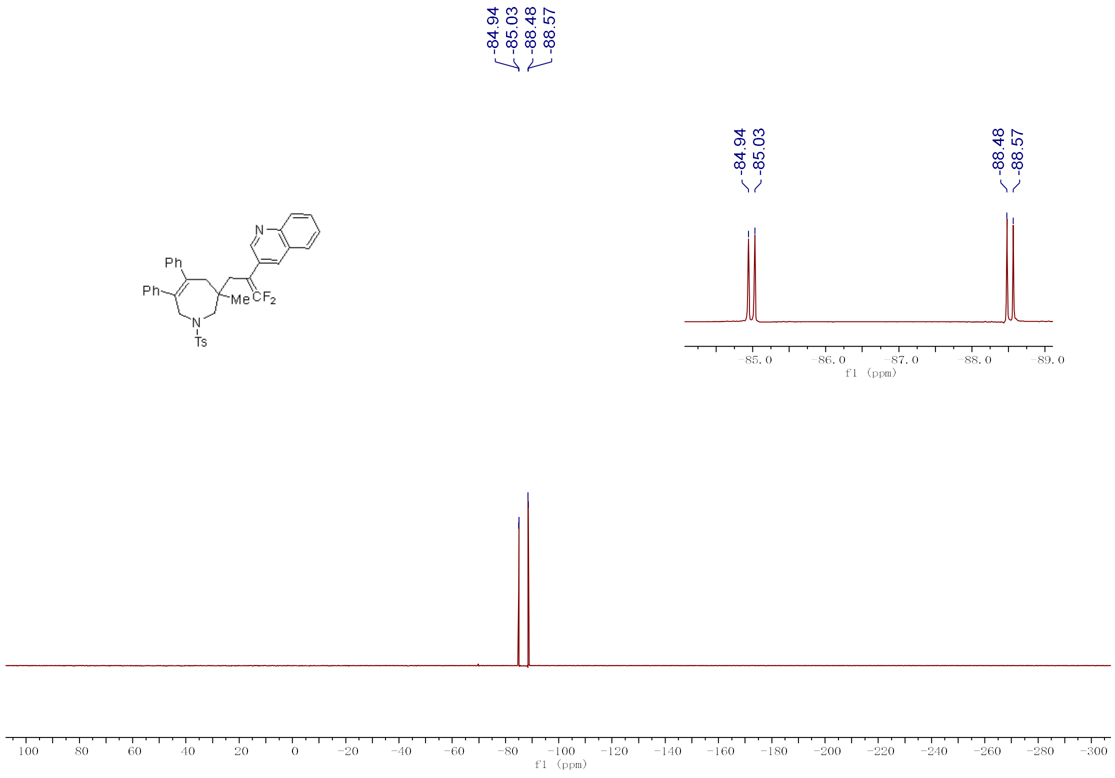

Supplementary figure 132. ^{19}F NMR of compound **41**

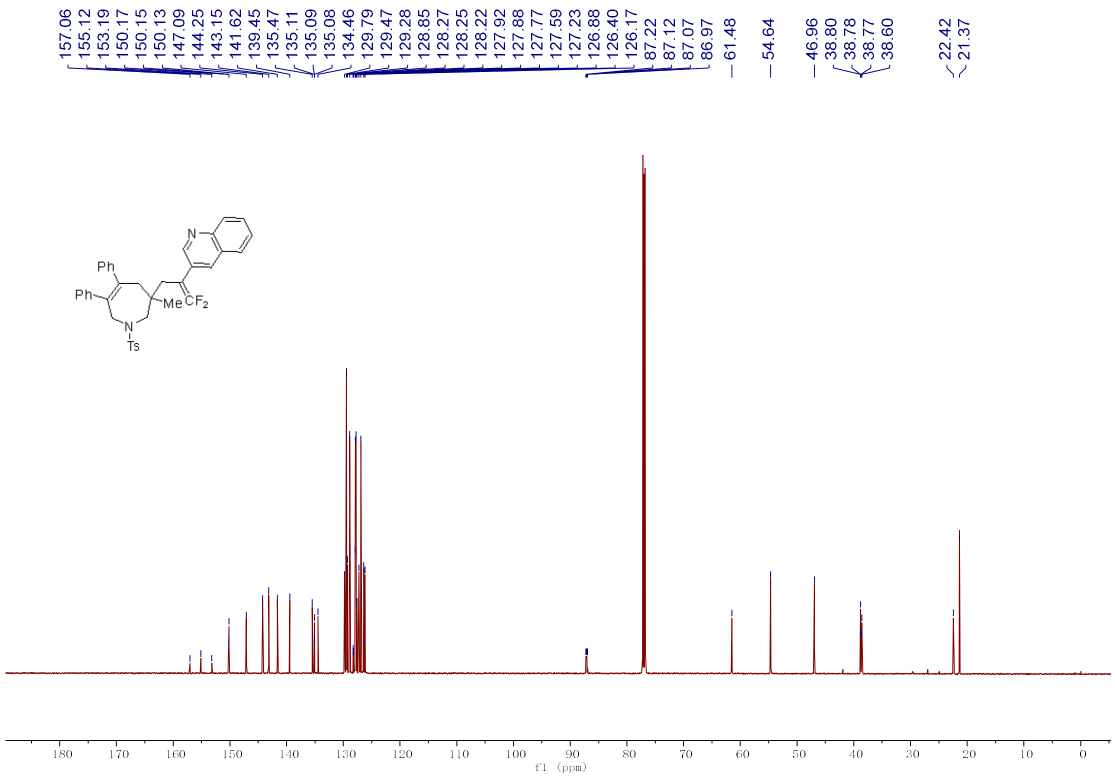

Supplementary figure 133. ^{13}C NMR of compound **41**

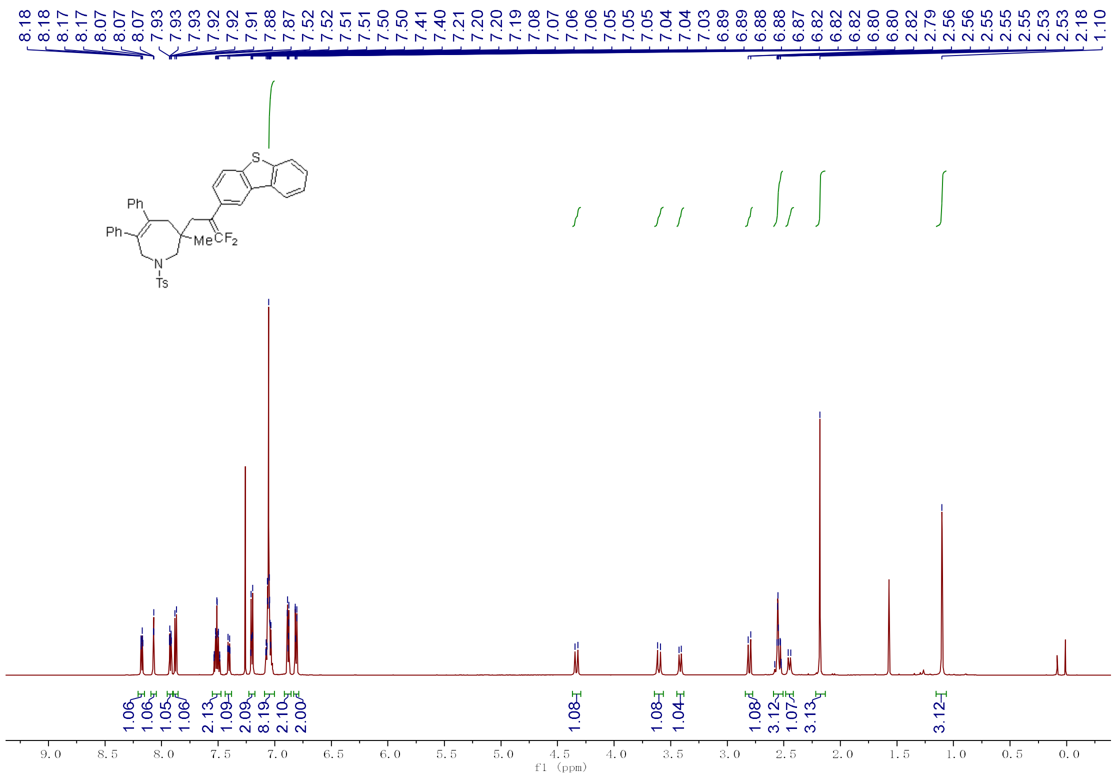

Supplementary figure 134. ^{1}H NMR of compound **42**

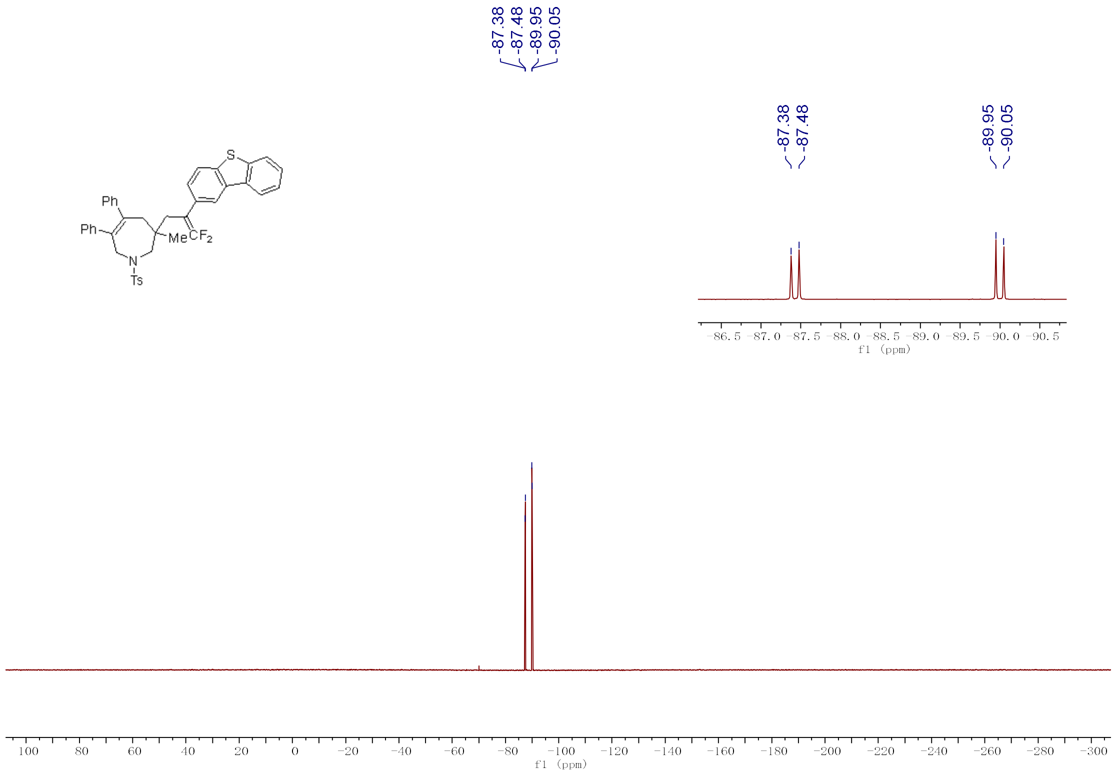

Supplementary figure 135. ^{19}F NMR of compound **42**

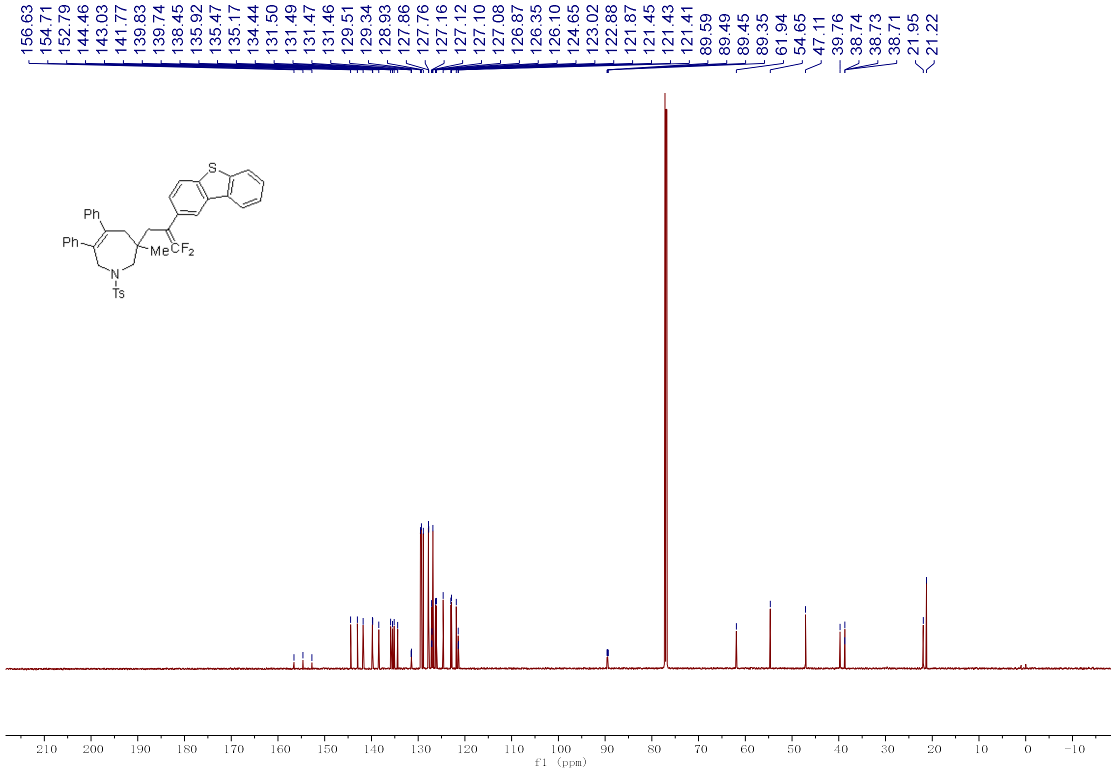

Supplementary figure 136. ^{13}C NMR of compound **42**

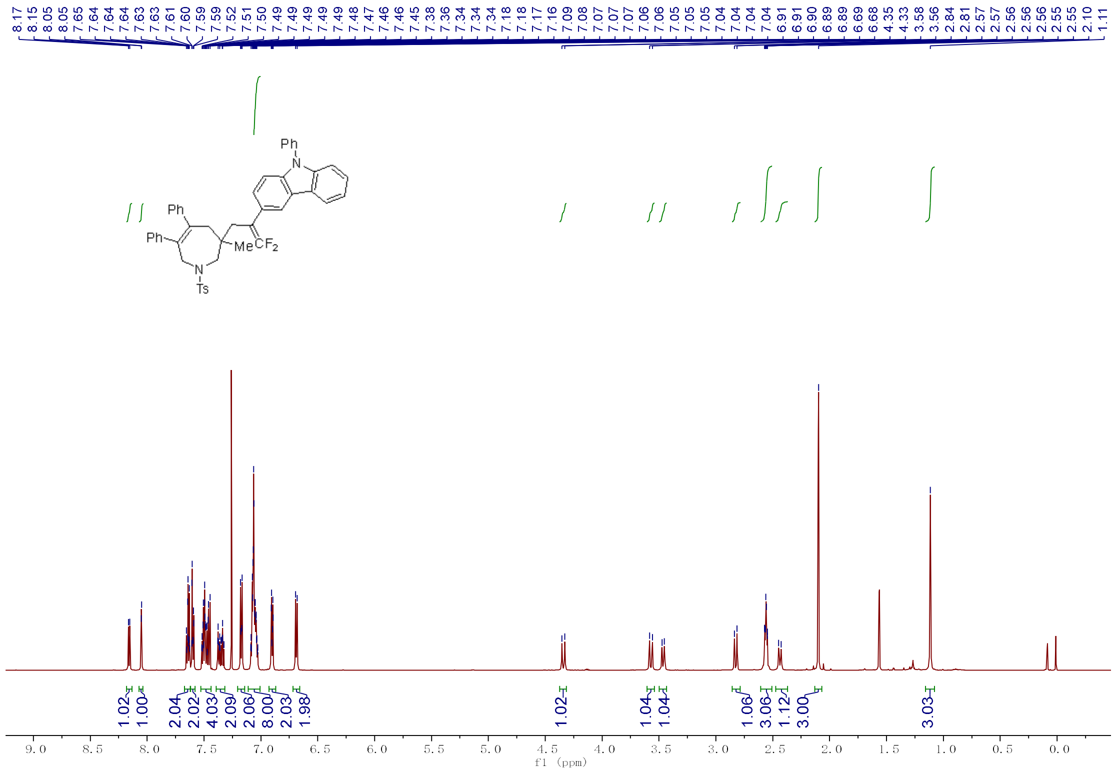

Supplementary figure 137. ^{1}H NMR of compound **43**

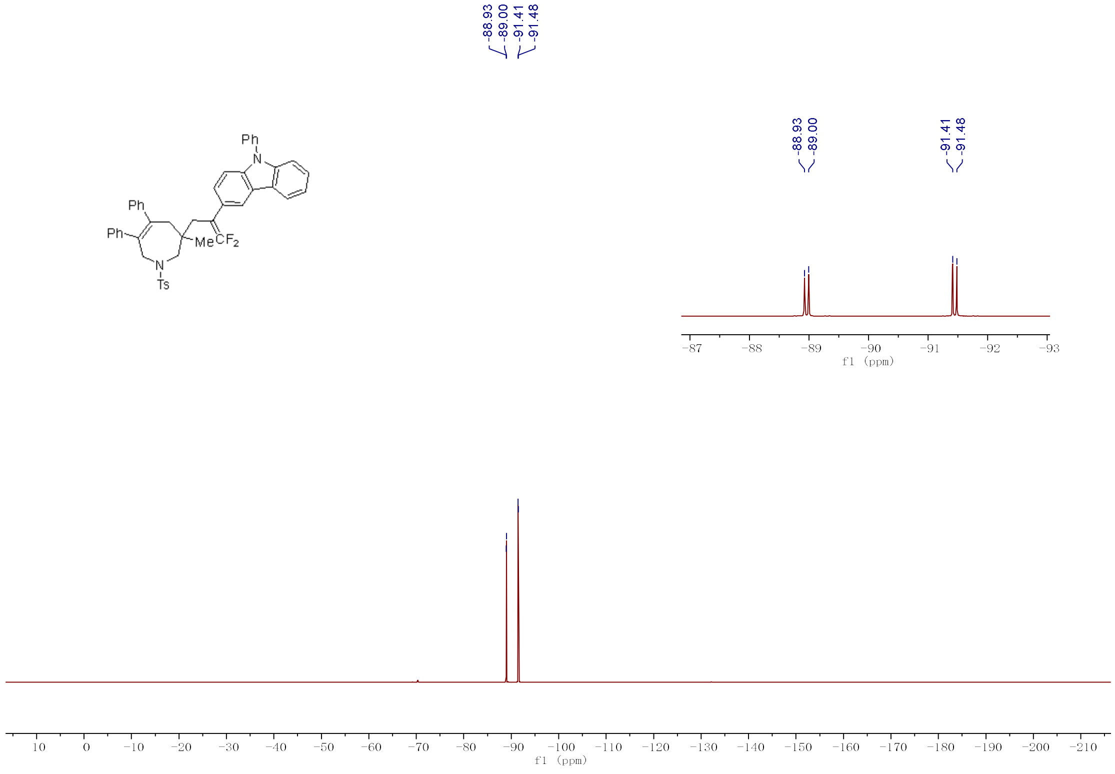

Supplementary figure 138. ^{19}F NMR of compound **43**

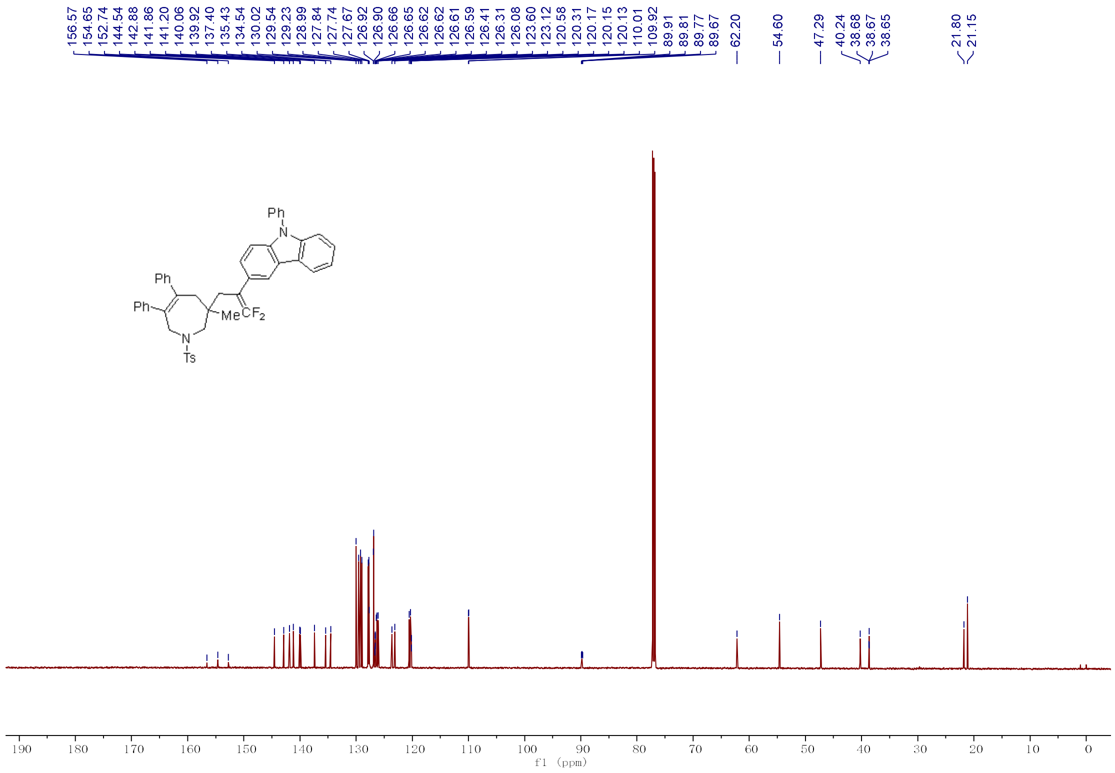

Supplementary figure 139. ^{13}C NMR of compound **43**

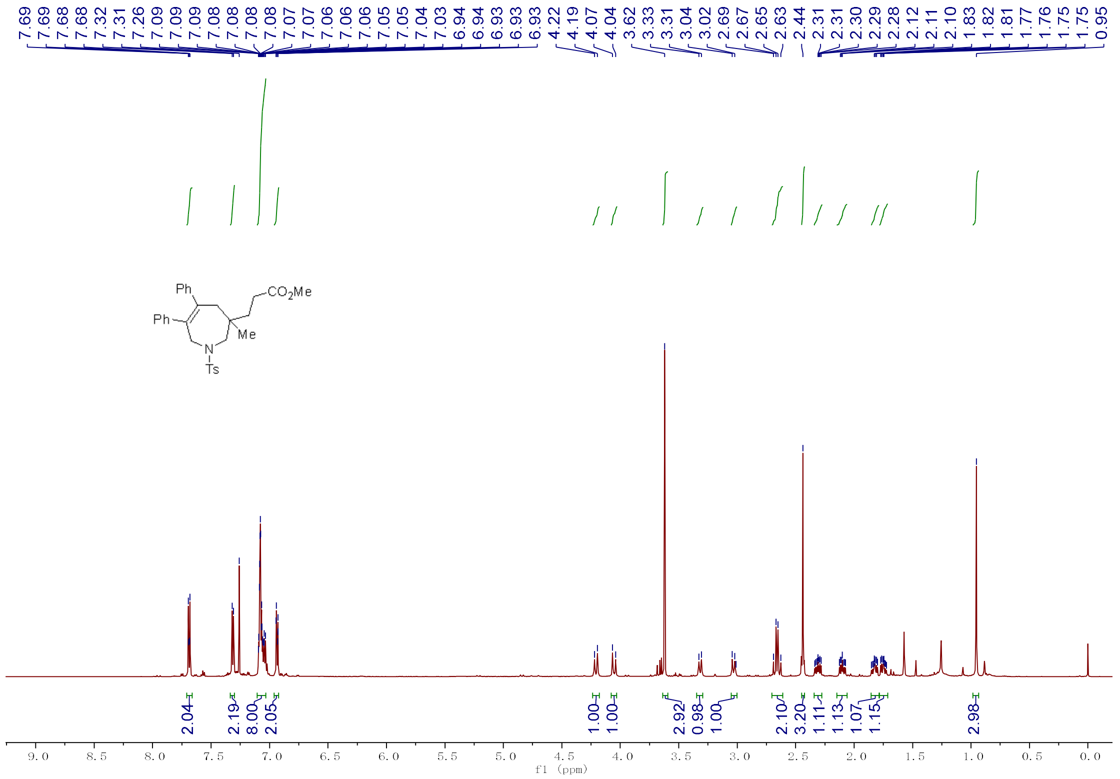

Supplementary figure 140. ^{1}H NMR of compound **44**

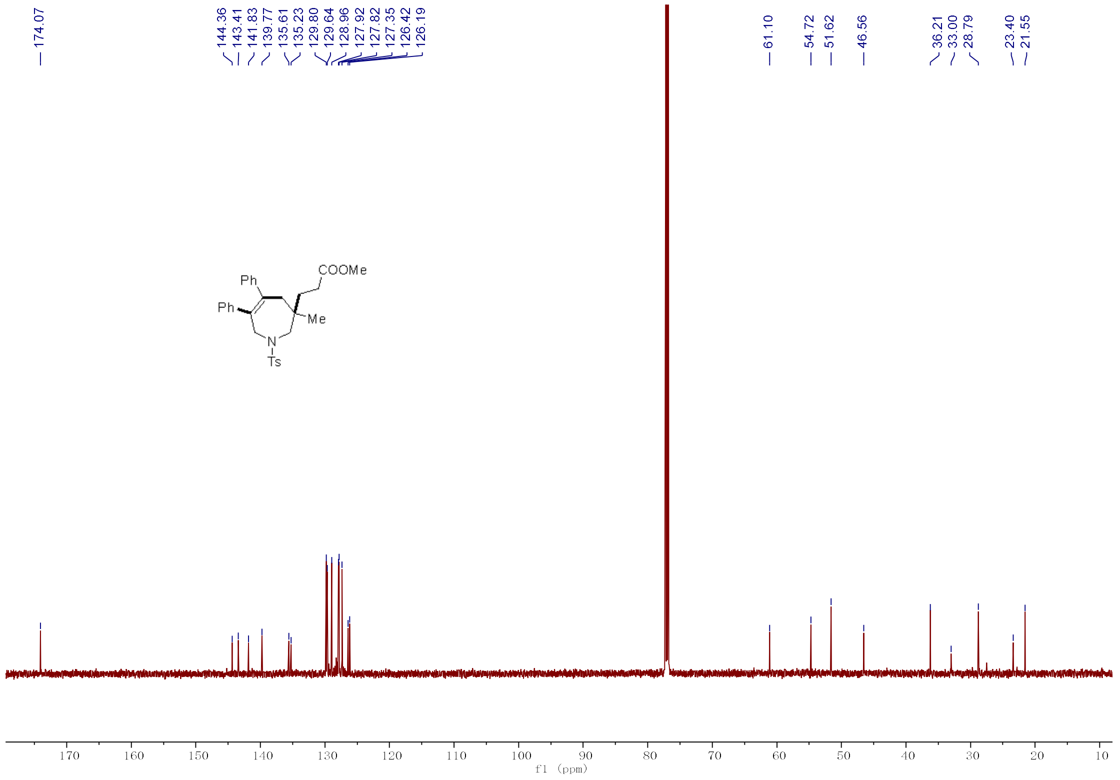

Supplementary figure 141. ^{13}C NMR of compound **44**

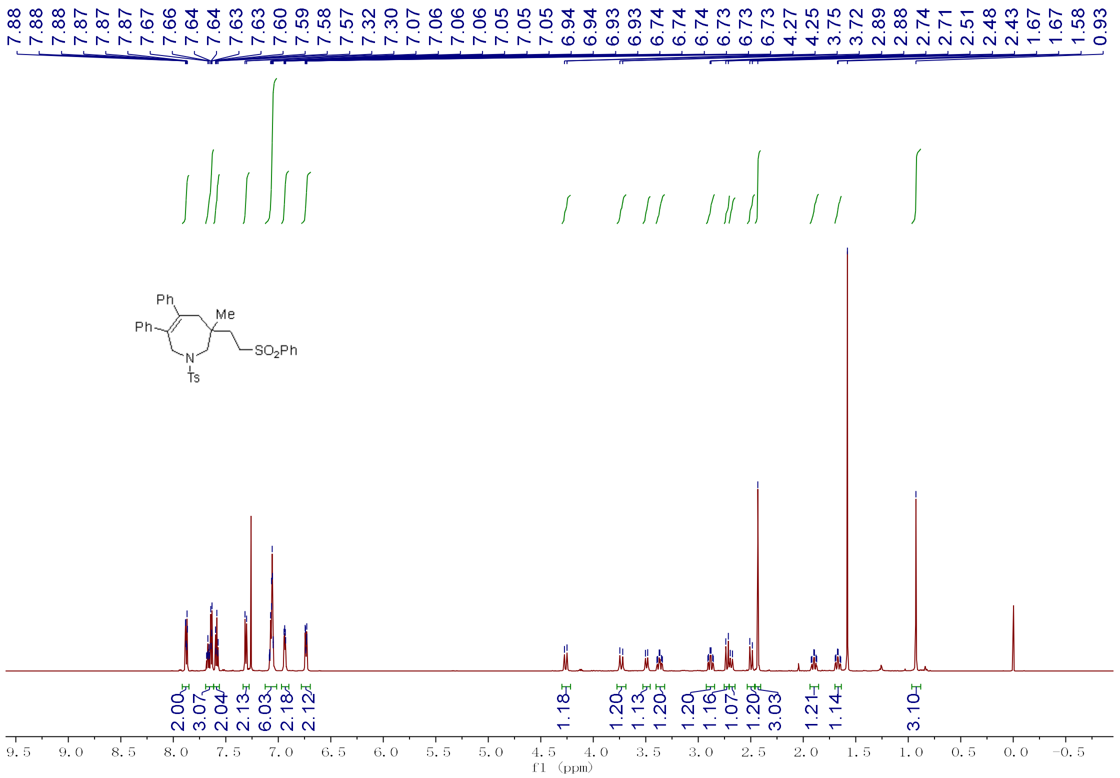

Supplementary figure 142. ^{1}H NMR of compound **45**

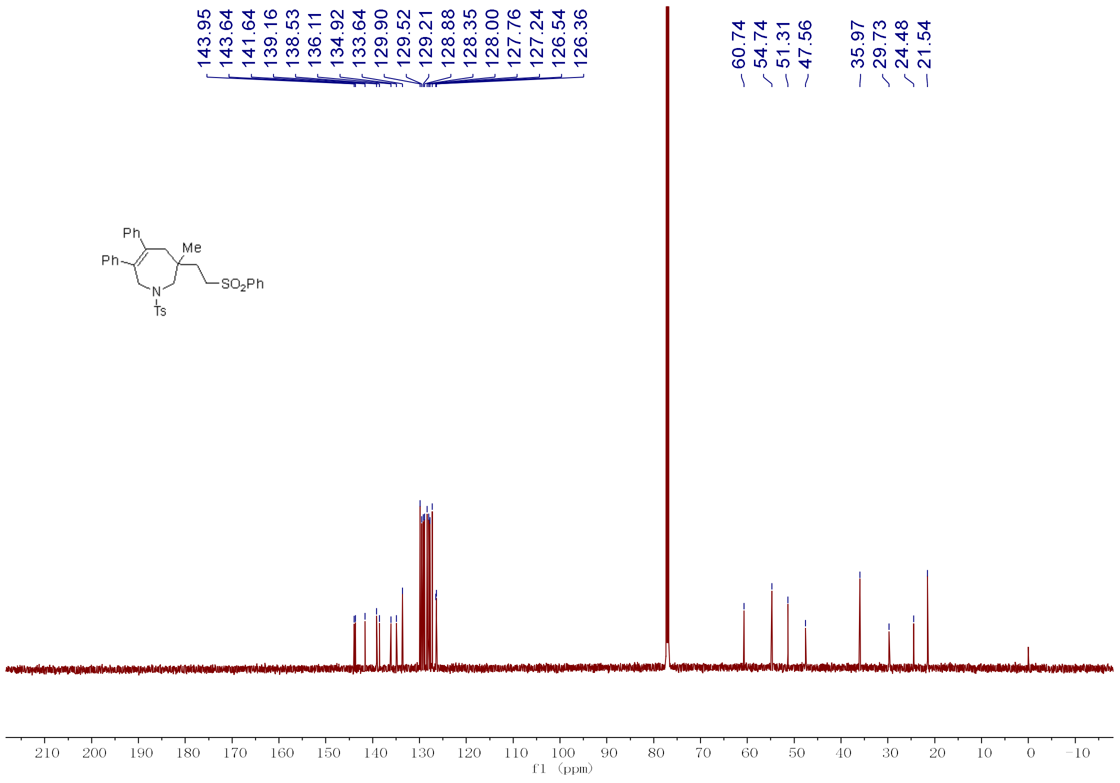

Supplementary figure 143. ^{13}C NMR of compound **45**

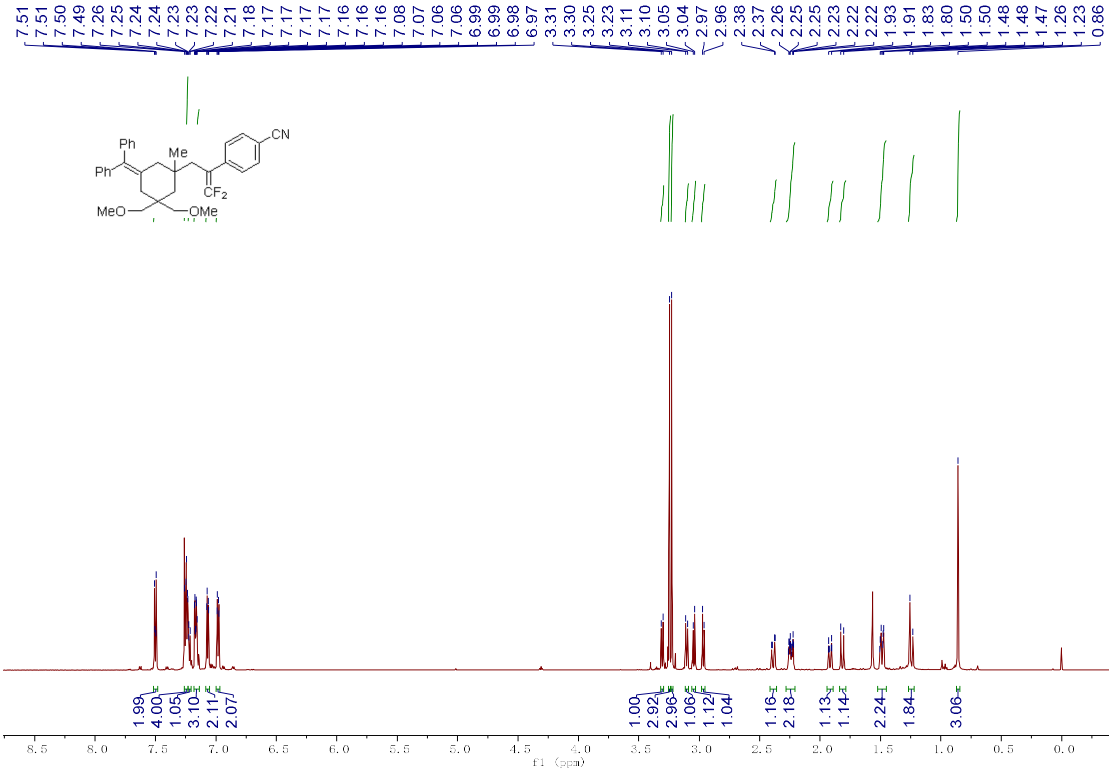

Supplementary figure 144. ^{1}H NMR of compound **46**

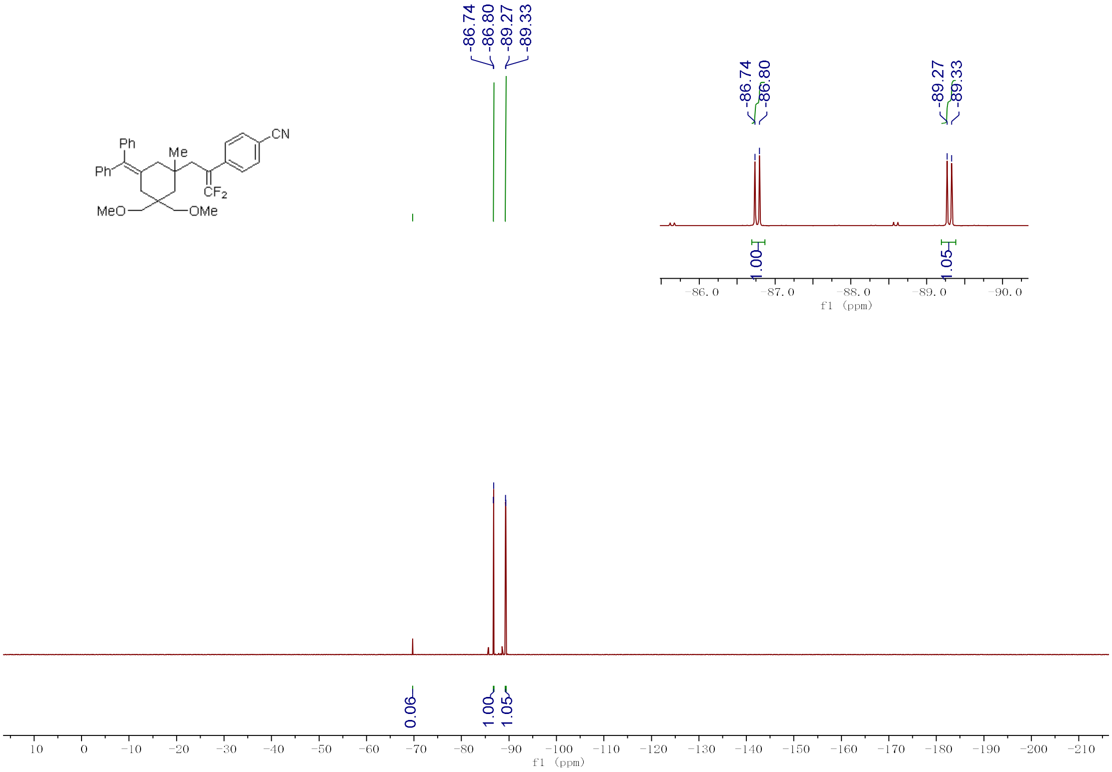

Supplementary figure 145. ^{19}F NMR of compound **46**

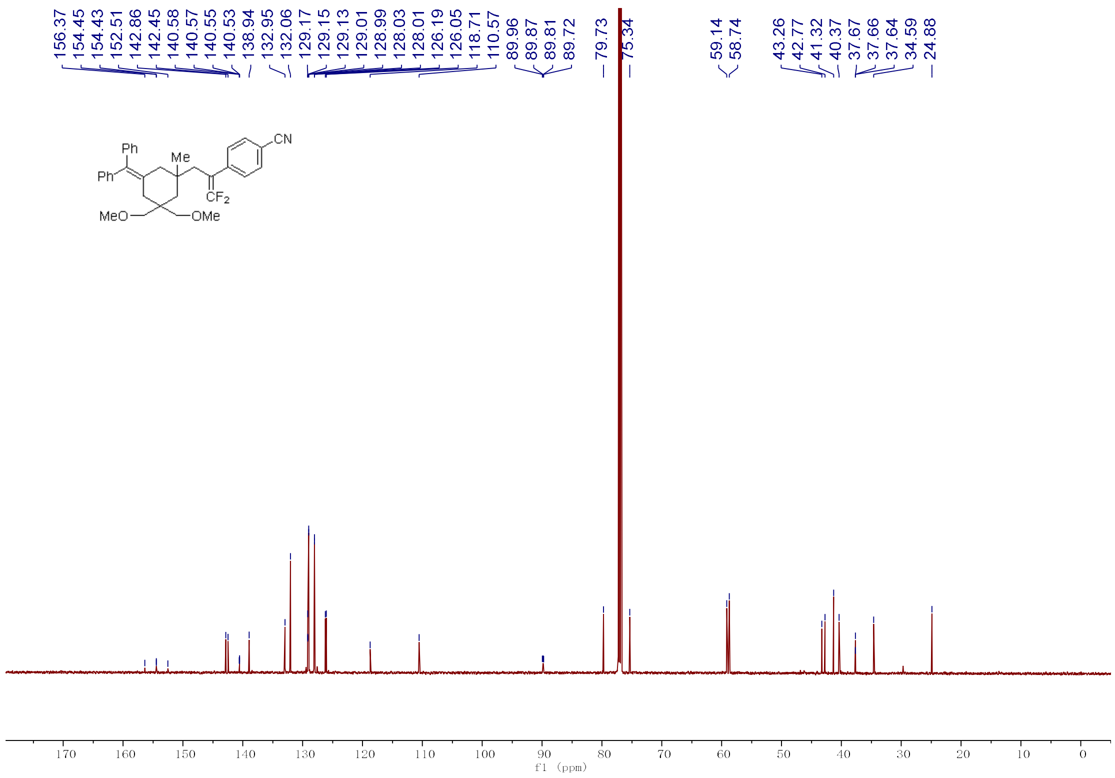

Supplementary figure 146. ^{13}C NMR of compound **46**

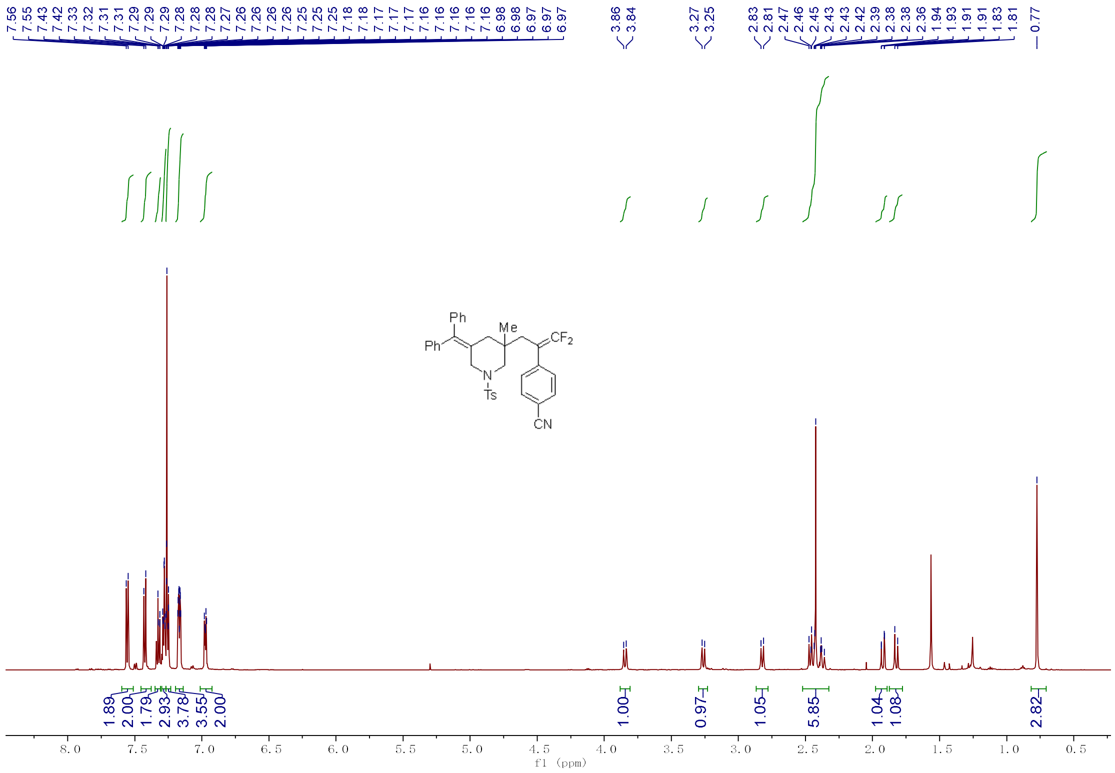

Supplementary figure 147. ^{1}H NMR of compound **47**

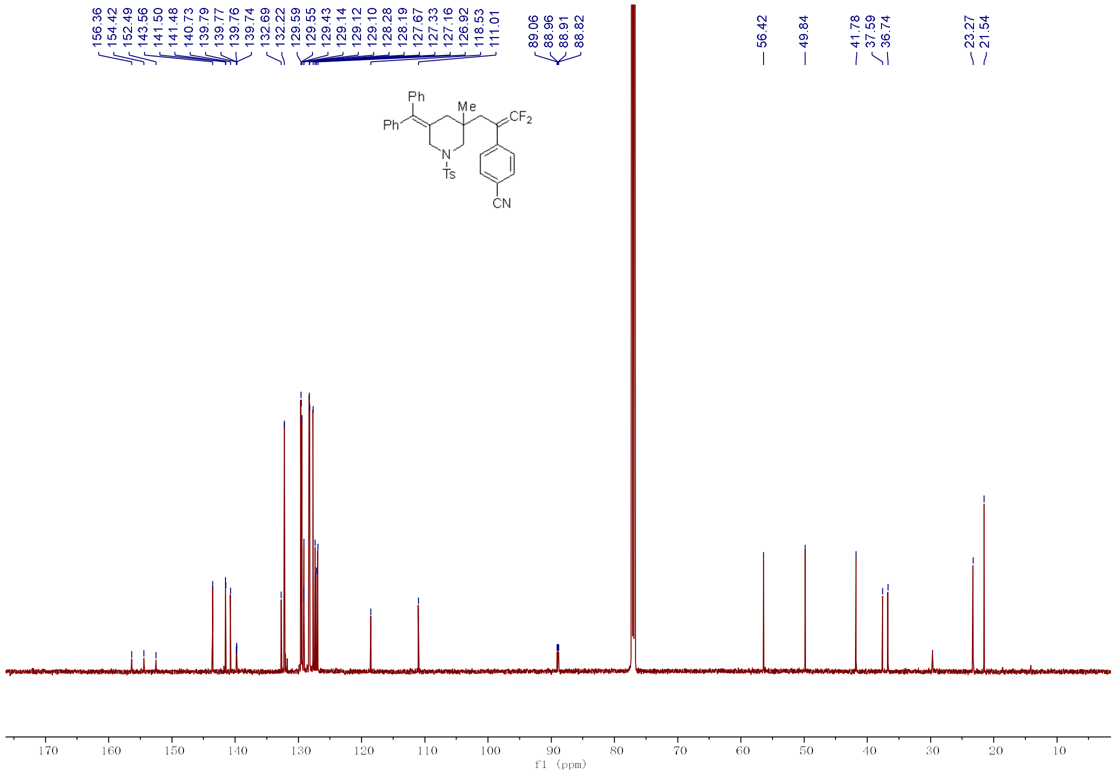

Supplementary figure 148. ^{13}C NMR of compound **47**

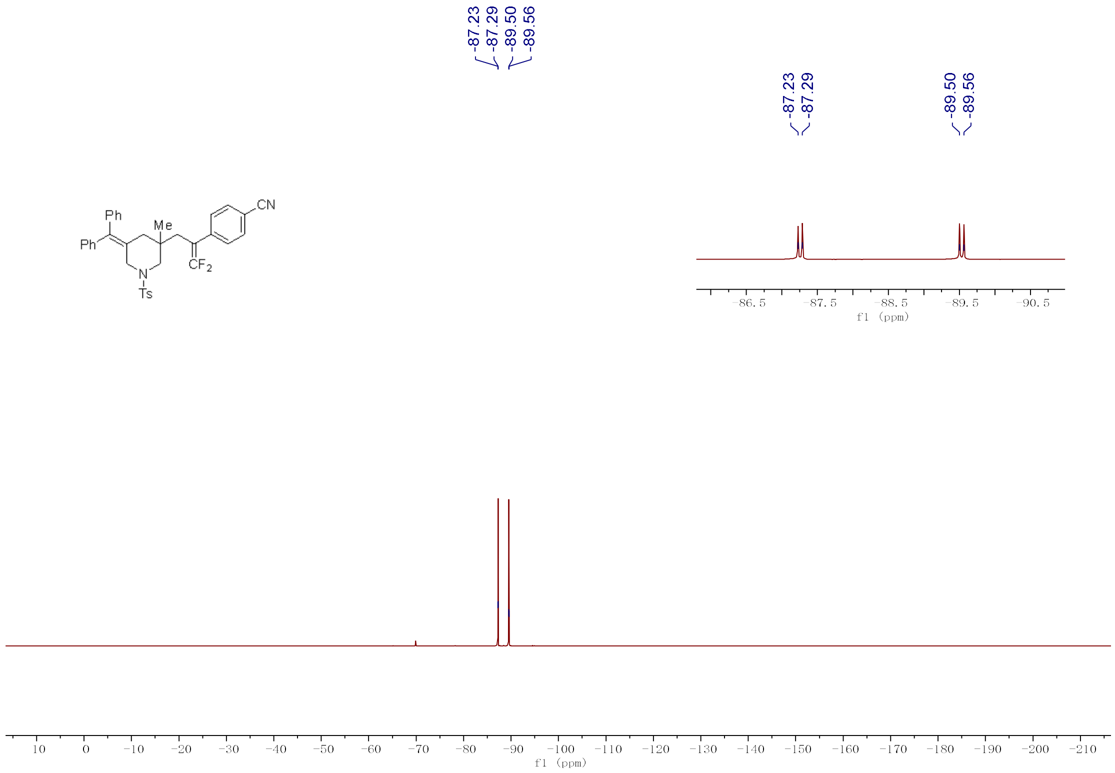

Supplementary figure 149. ^{19}F NMR of compound **47**

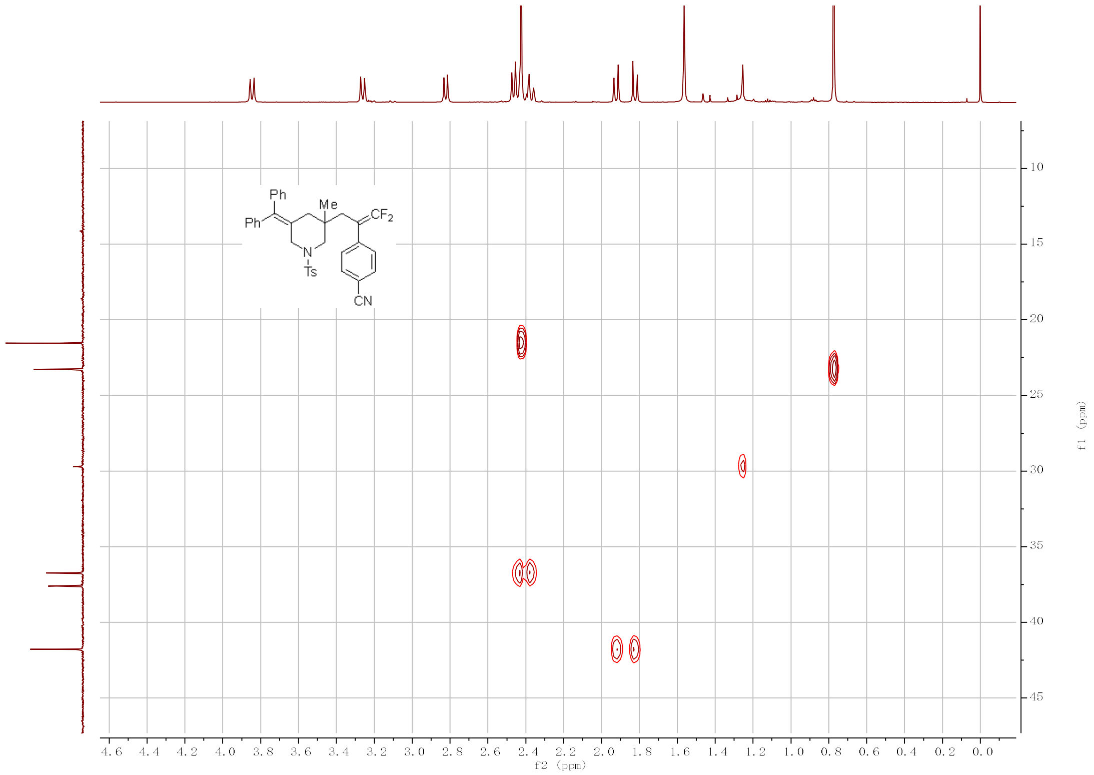

Supplementary figure 150. HSQC NMR of compound **47**

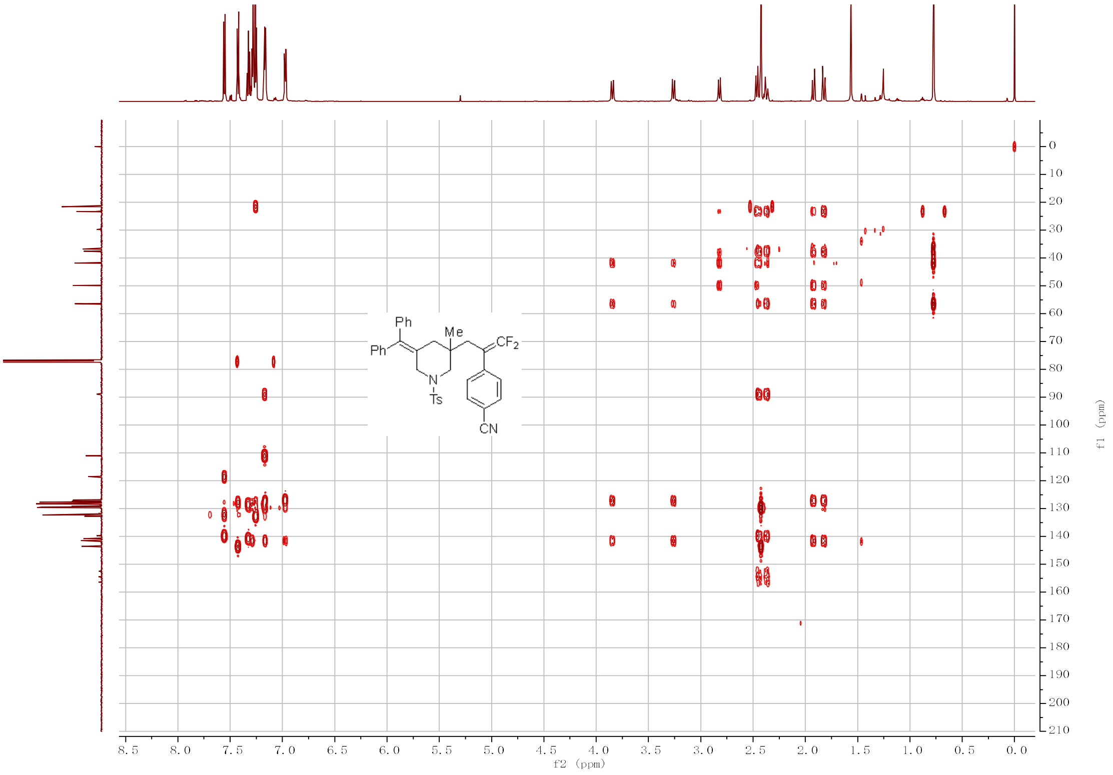

Supplementary figure 151. HMBC NMR of compound **47**

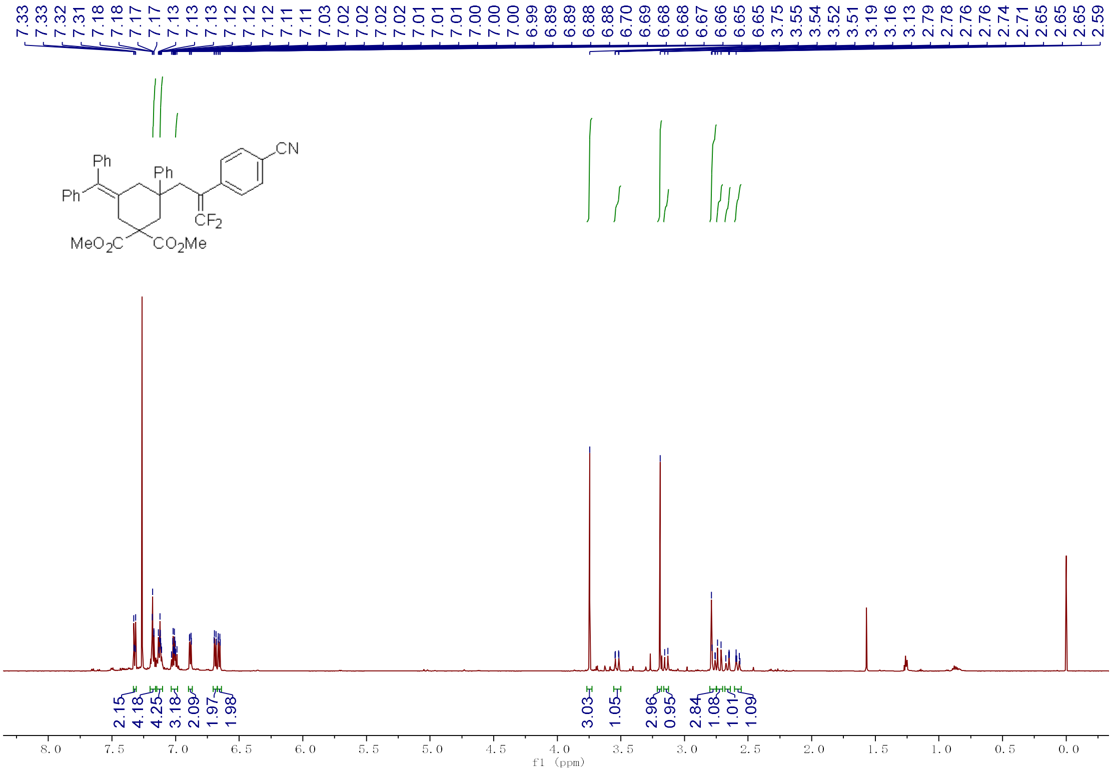

Supplementary figure 152. ^{1}H NMR of compound **48**

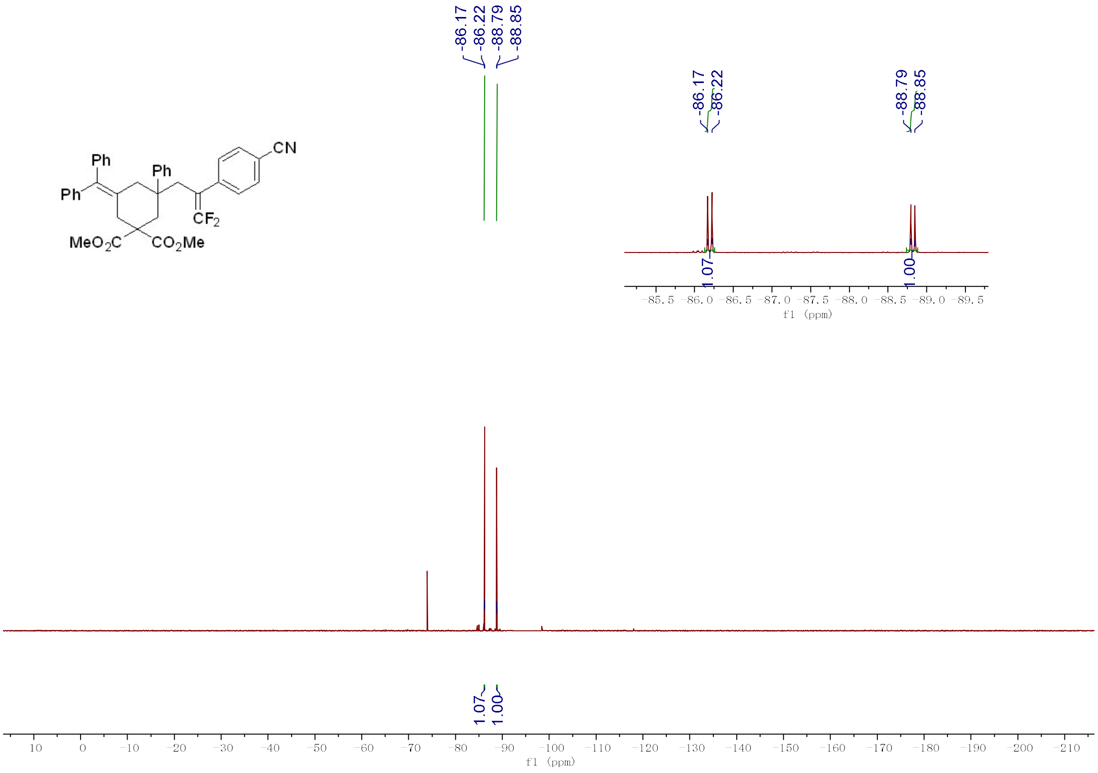

Supplementary figure 153. ^{19}F NMR of compound **48**

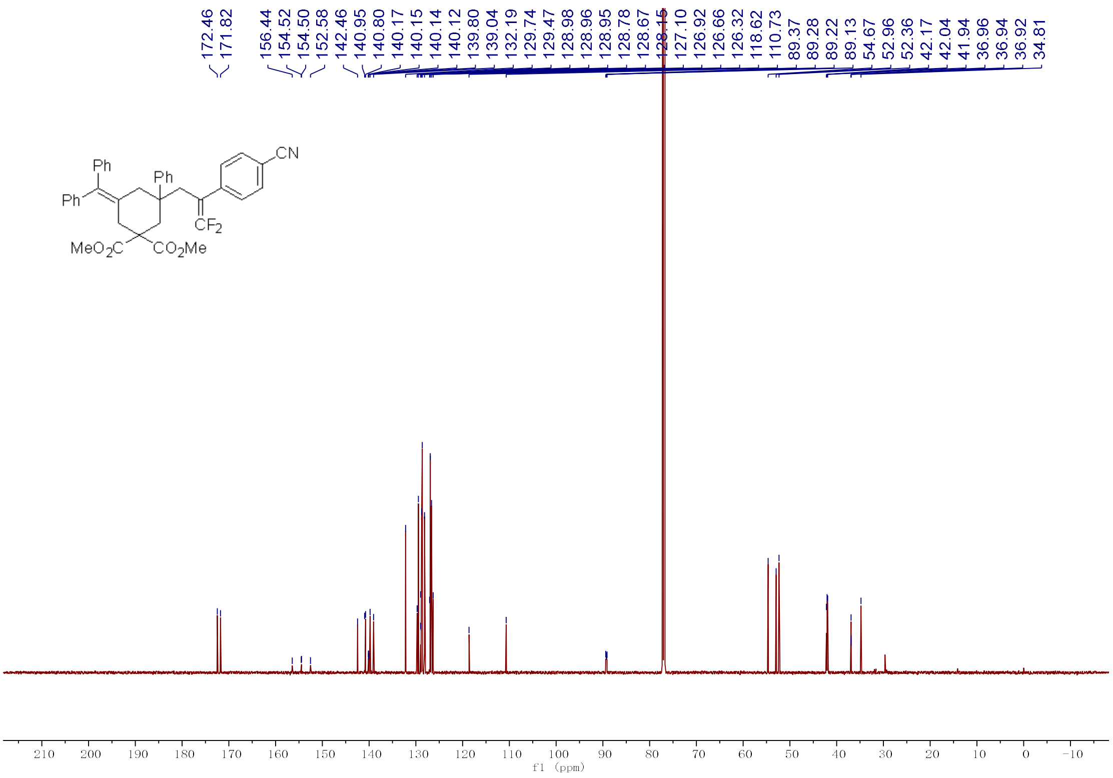

Supplementary figure 154. ^{13}C NMR of compound **48**

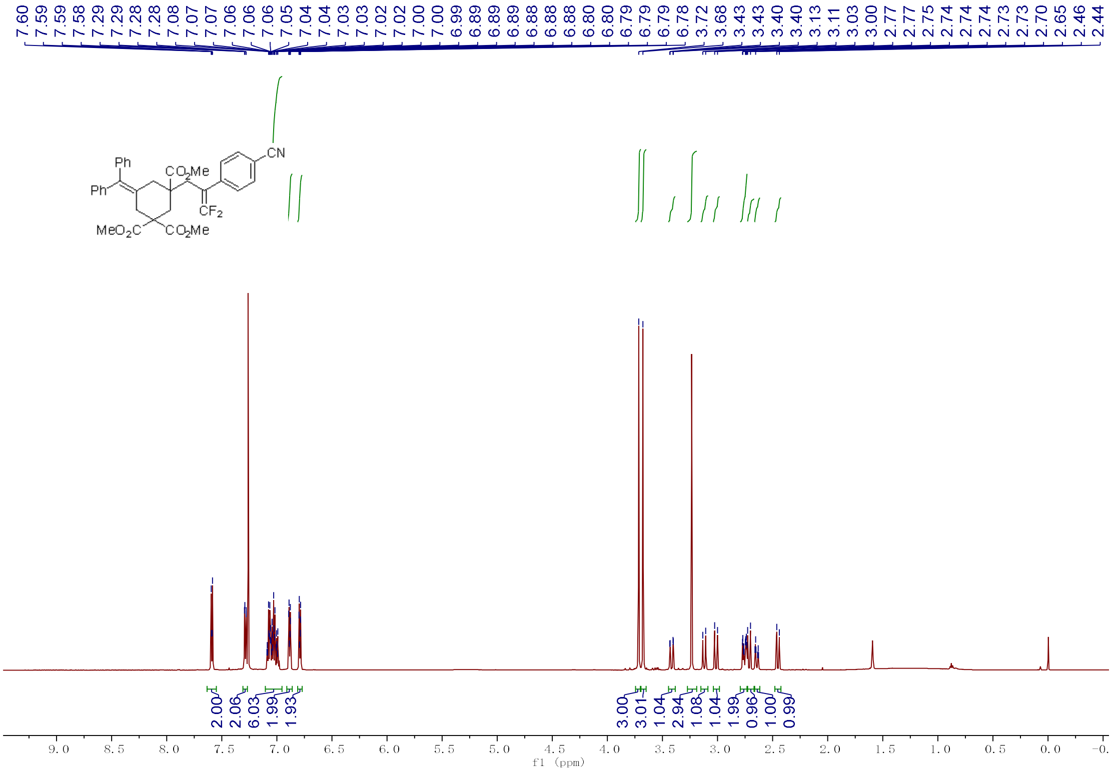

Supplementary figure 155. ^{1}H NMR of compound **49**

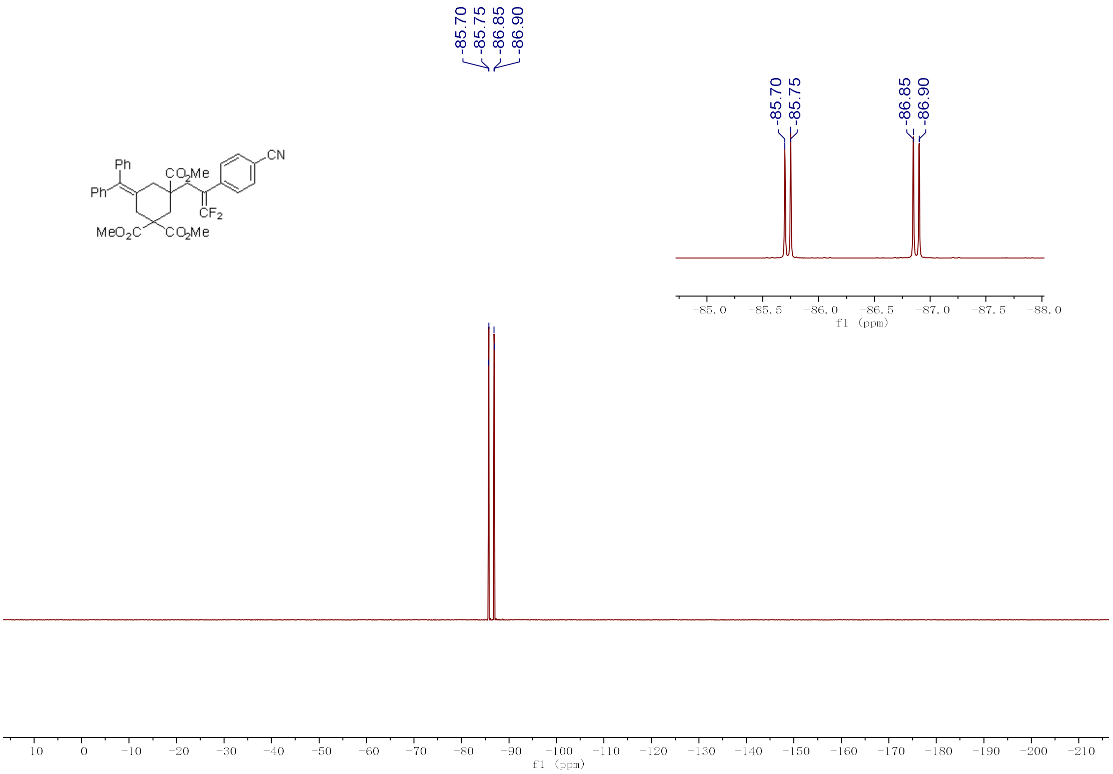

Supplementary figure 156. ^{19}F NMR of compound **49**

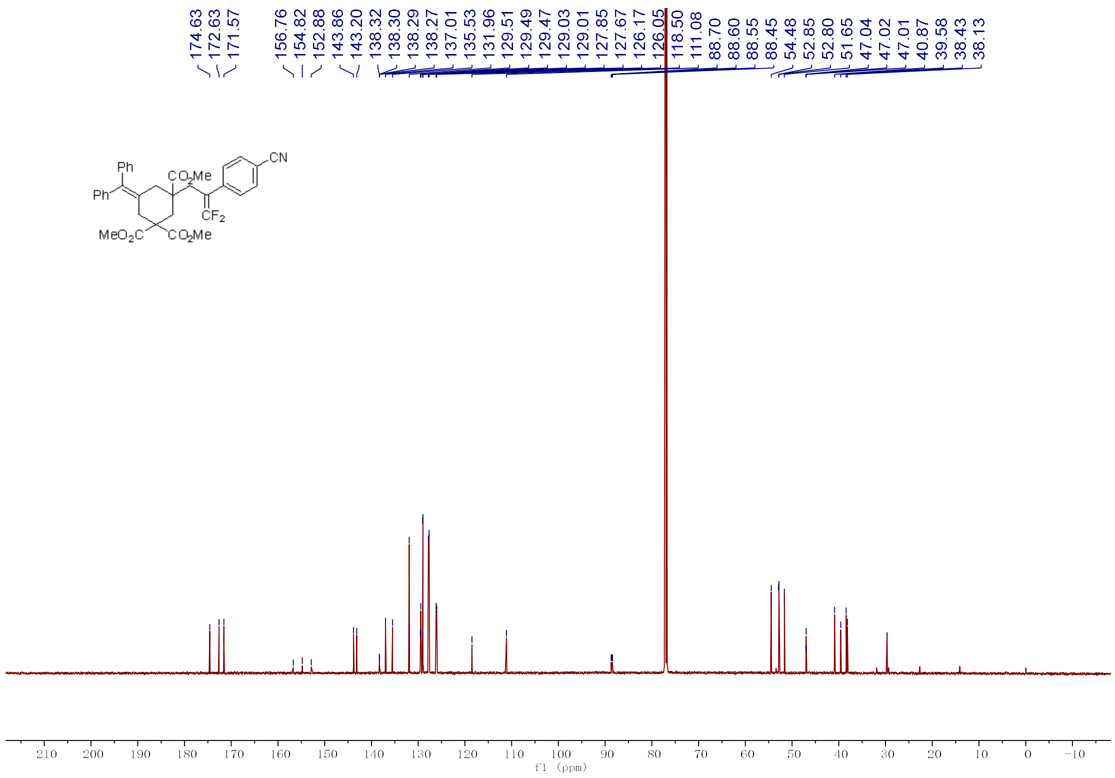

Supplementary figure 157. ^{13}C NMR of compound **49**

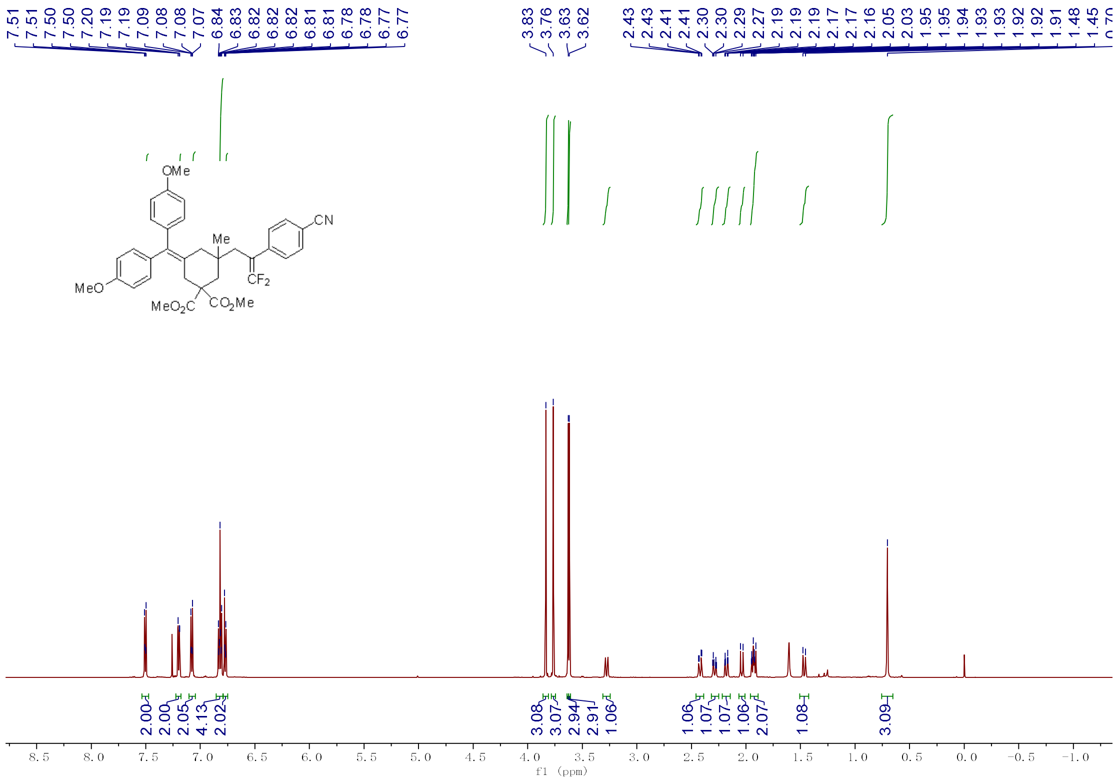

Supplementary figure 158. ^{1}H NMR of compound **50**

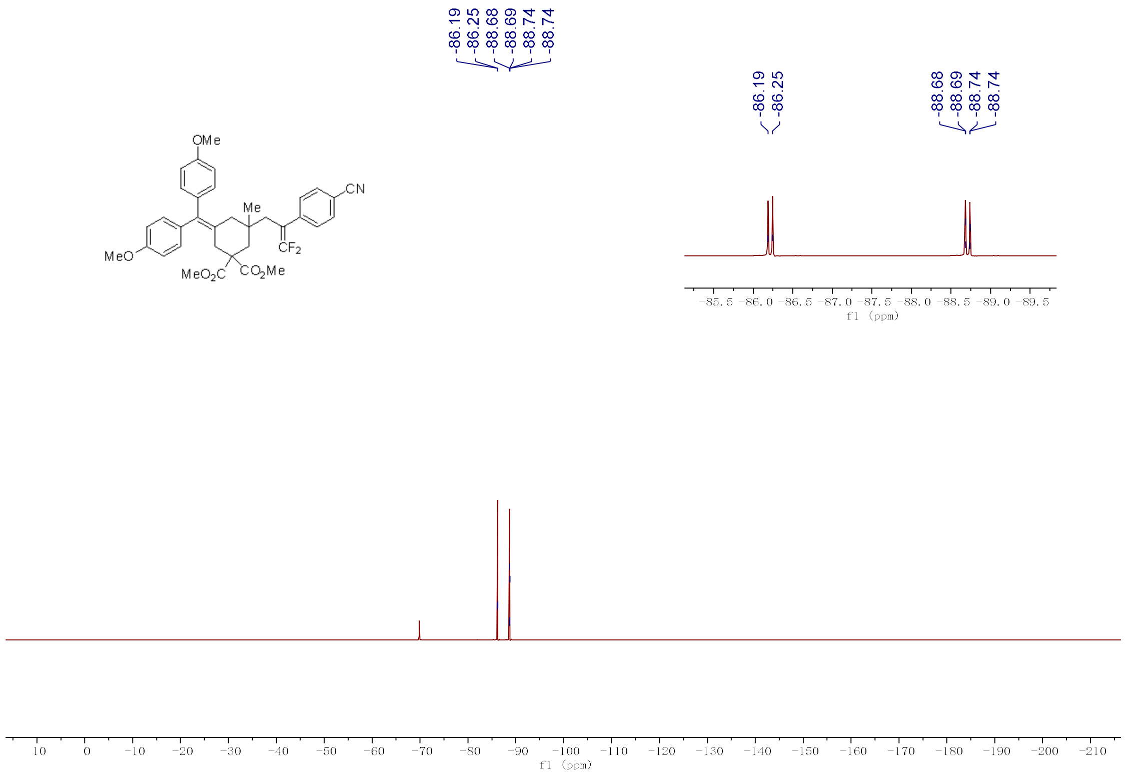

Supplementary figure 159. ^{19}F NMR of compound **50**

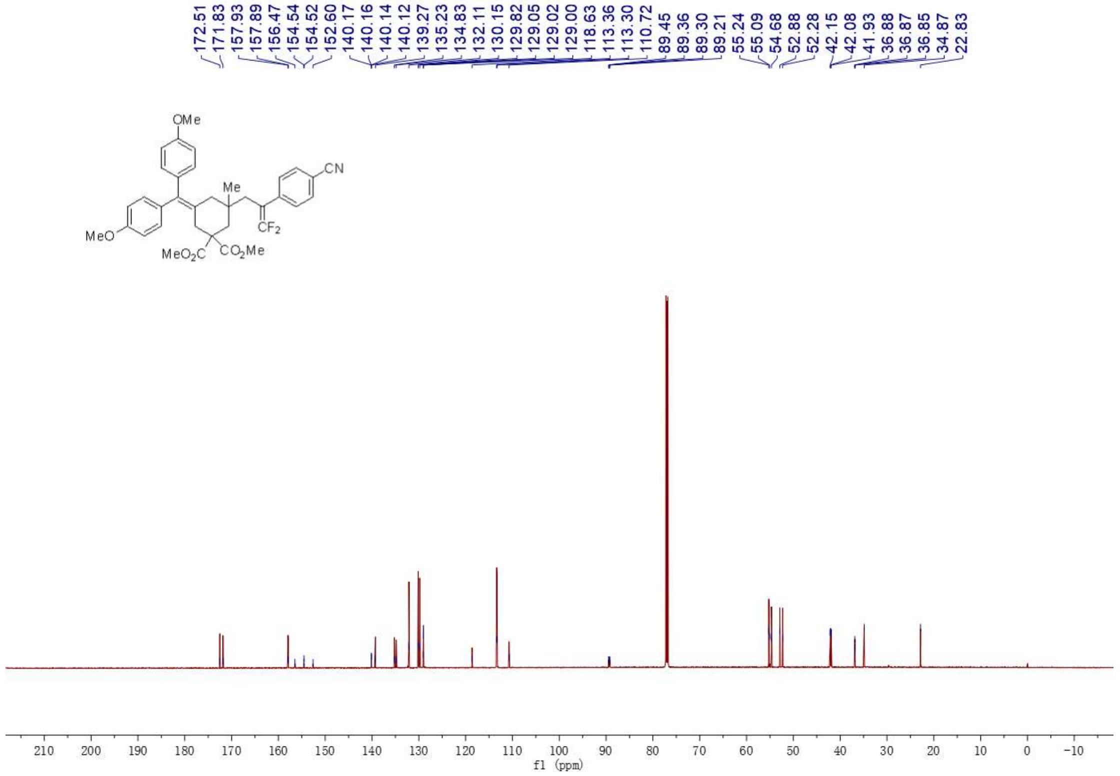

Supplementary figure 160. ^{13}C NMR of compound **50**

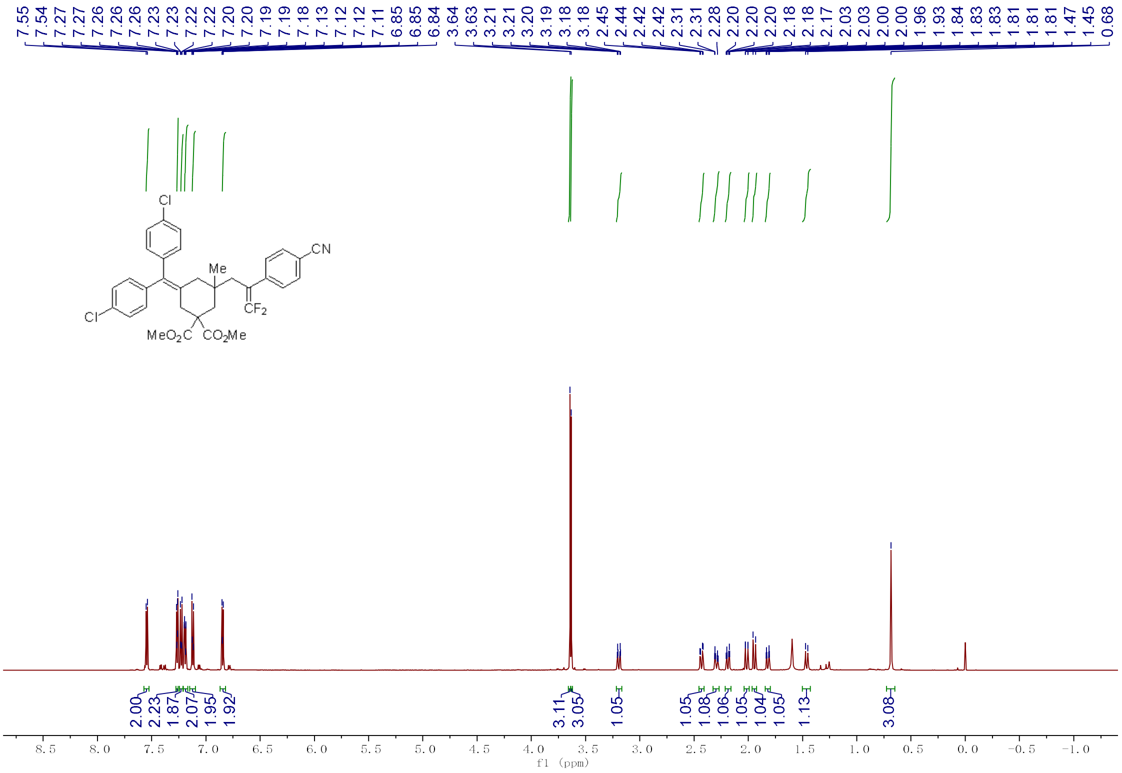

Supplementary figure 161. ^{1}H NMR of compound **51**

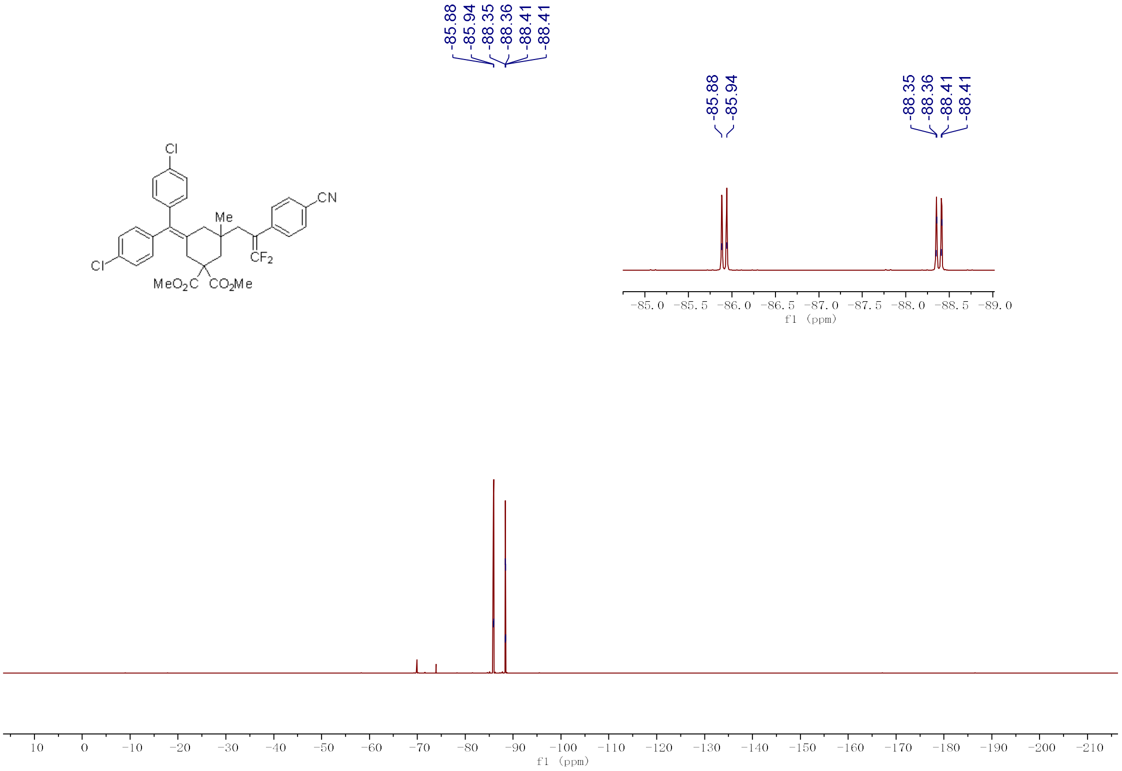

Supplementary figure 162. ^{19}F NMR of compound **51**

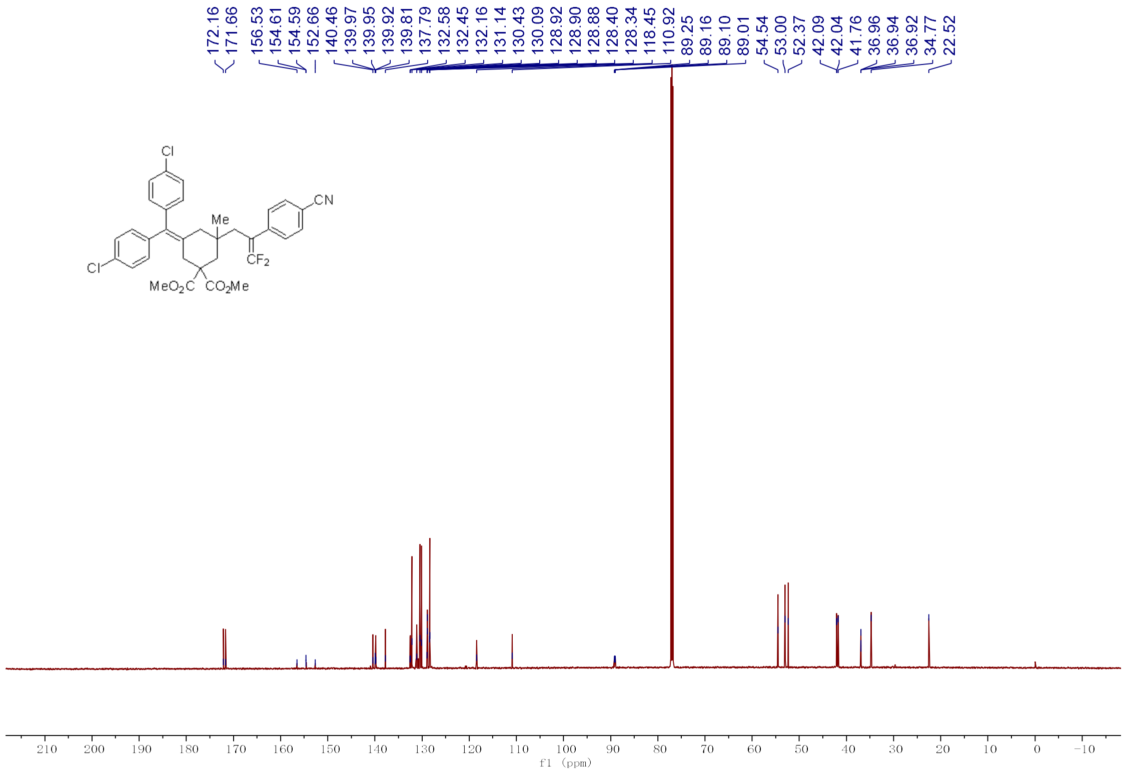

Supplementary figure 163. ^{13}C NMR of compound **51**

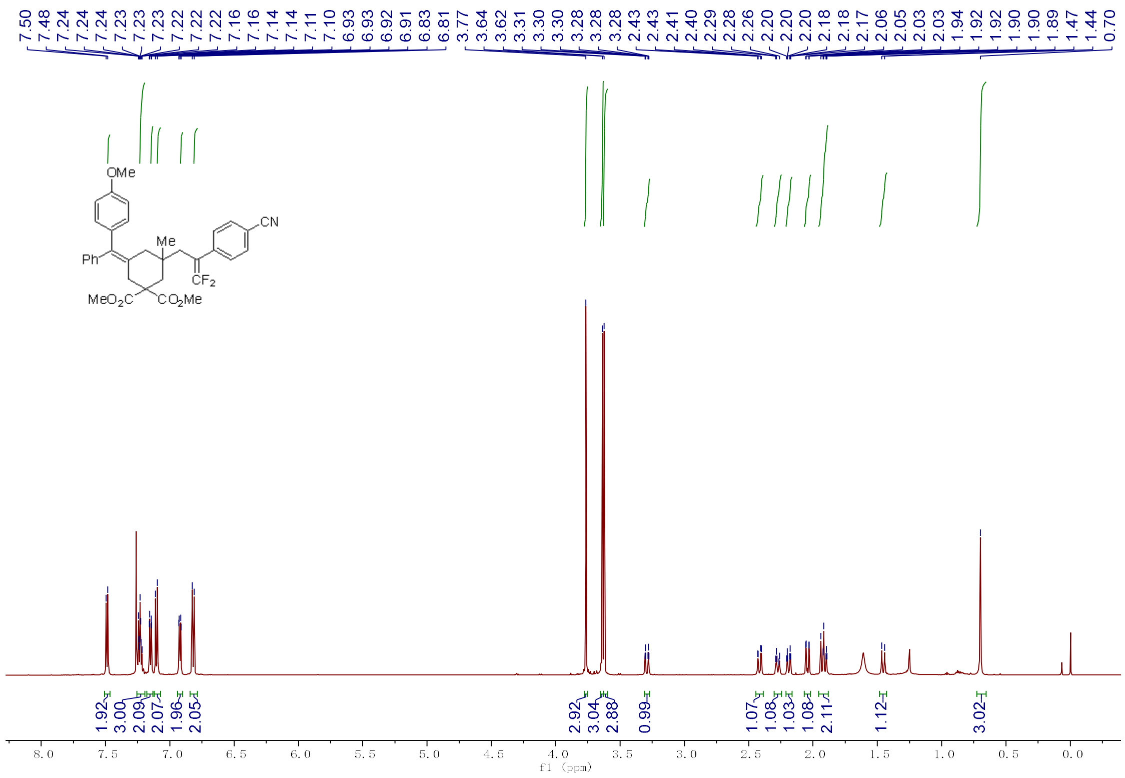

Supplementary figure 164. ^{1}H NMR of compound **52**

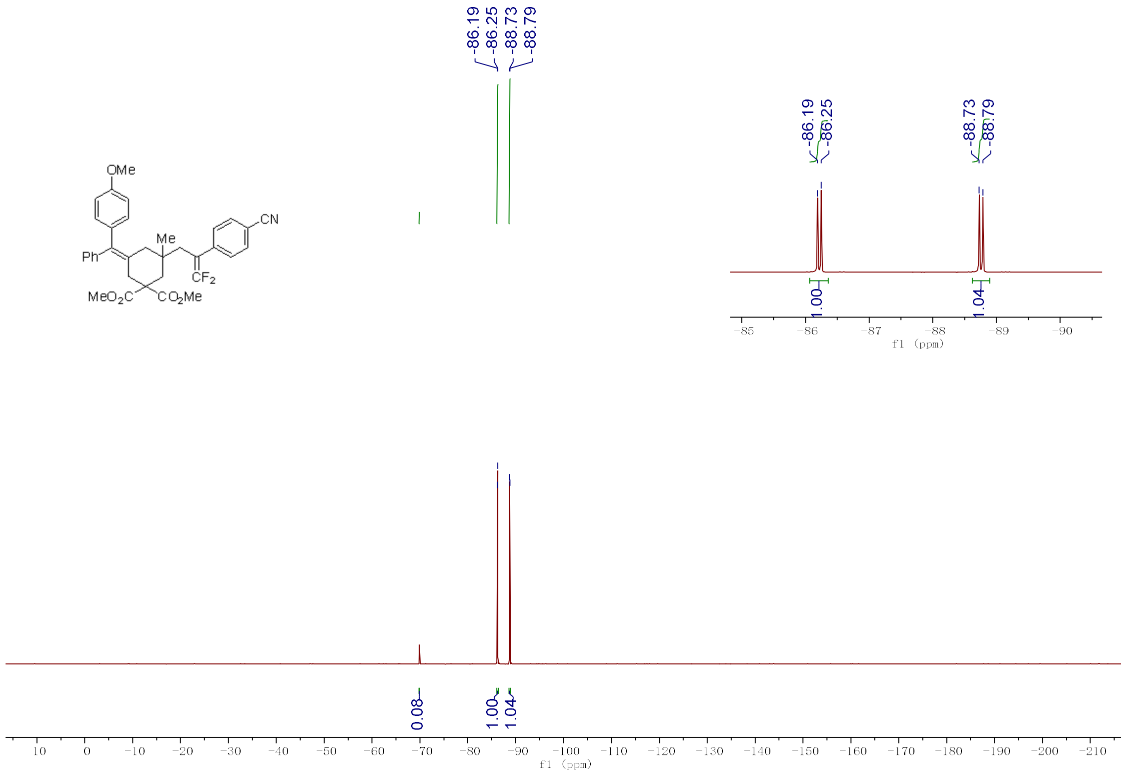

Supplementary figure 165. ^{19}F NMR of compound **52**

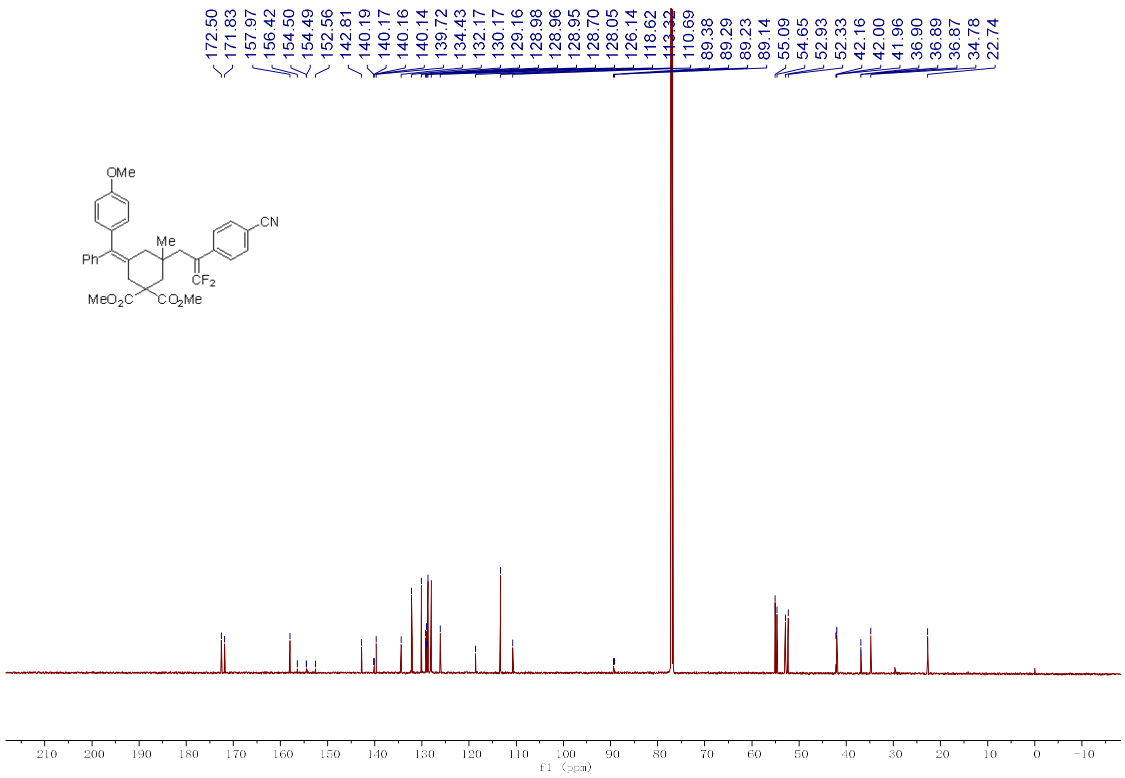

Supplementary figure 166. ^{13}C NMR of compound **52**

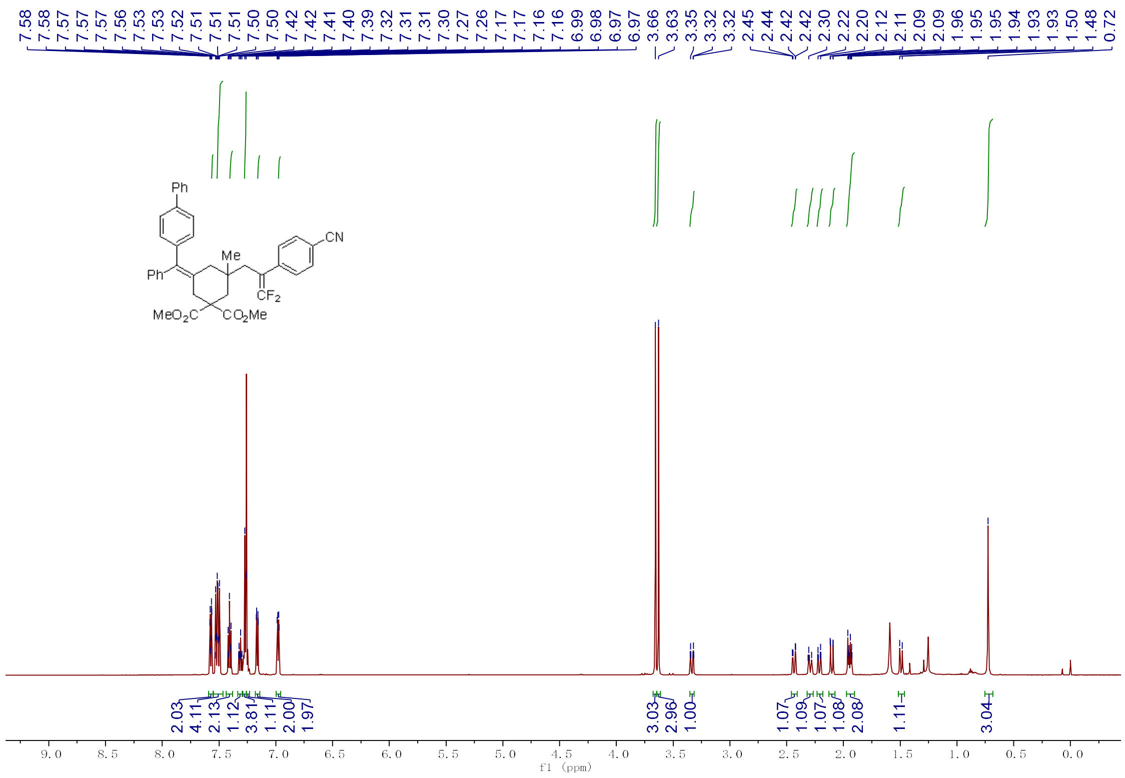

Supplementary figure 167. ^{1}H NMR of compound **53**

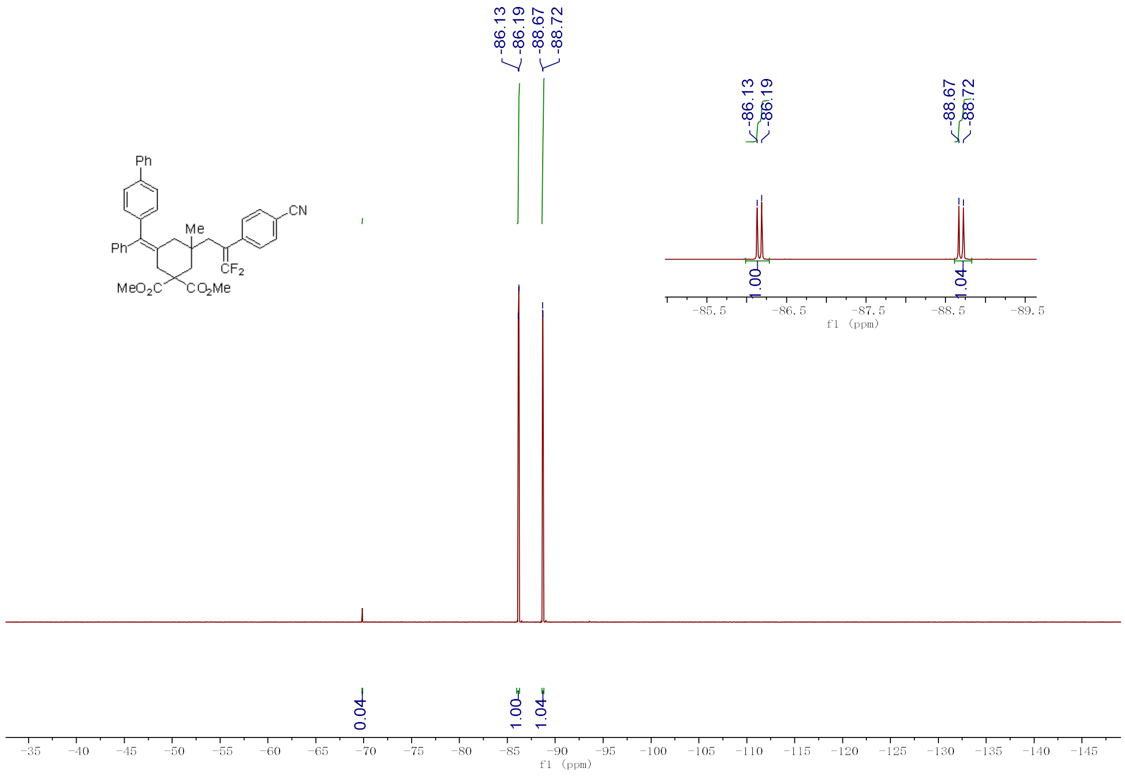

Supplementary figure 168. ^{19}F NMR of compound **53**

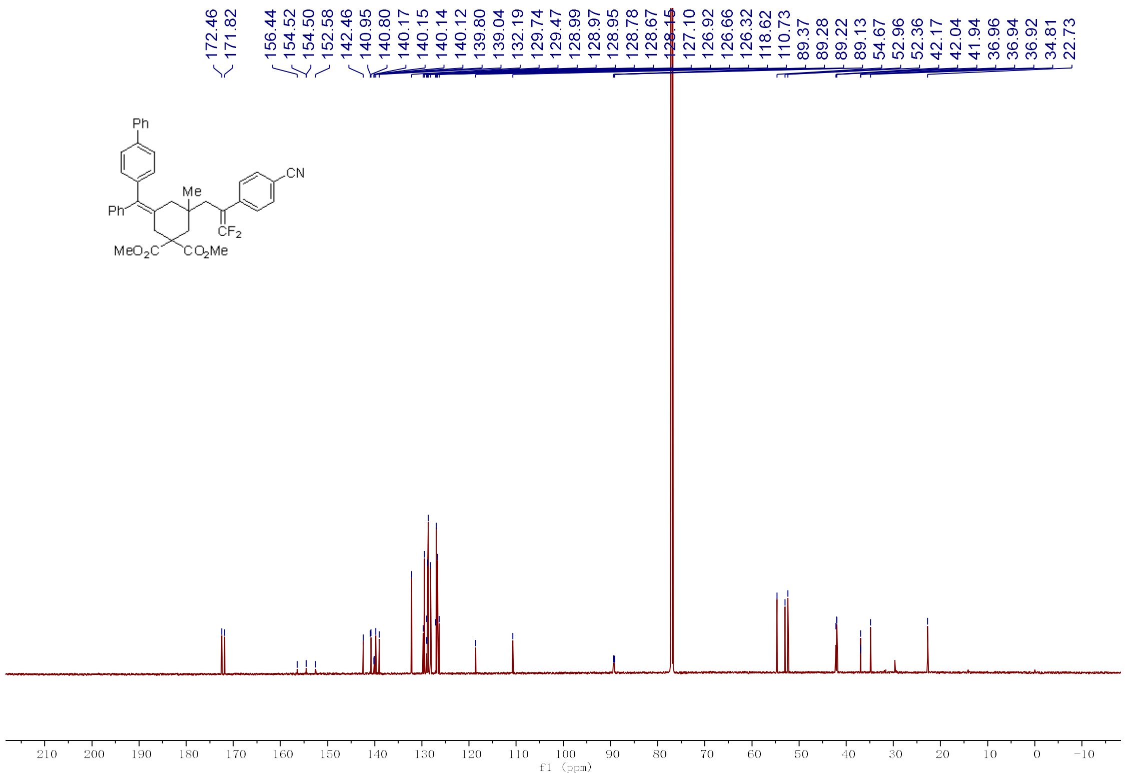

Supplementary figure 169. ^{13}C NMR of compound **53**

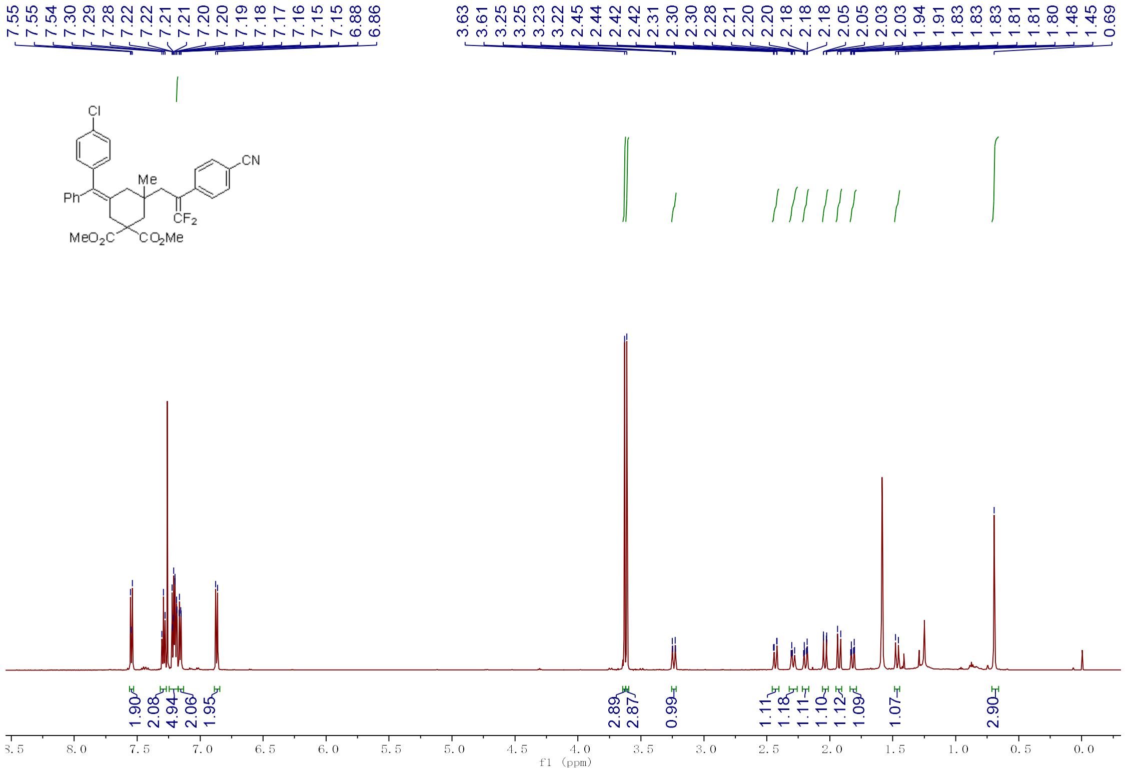

Supplementary figure 170. ^{1}H NMR of compound **54**

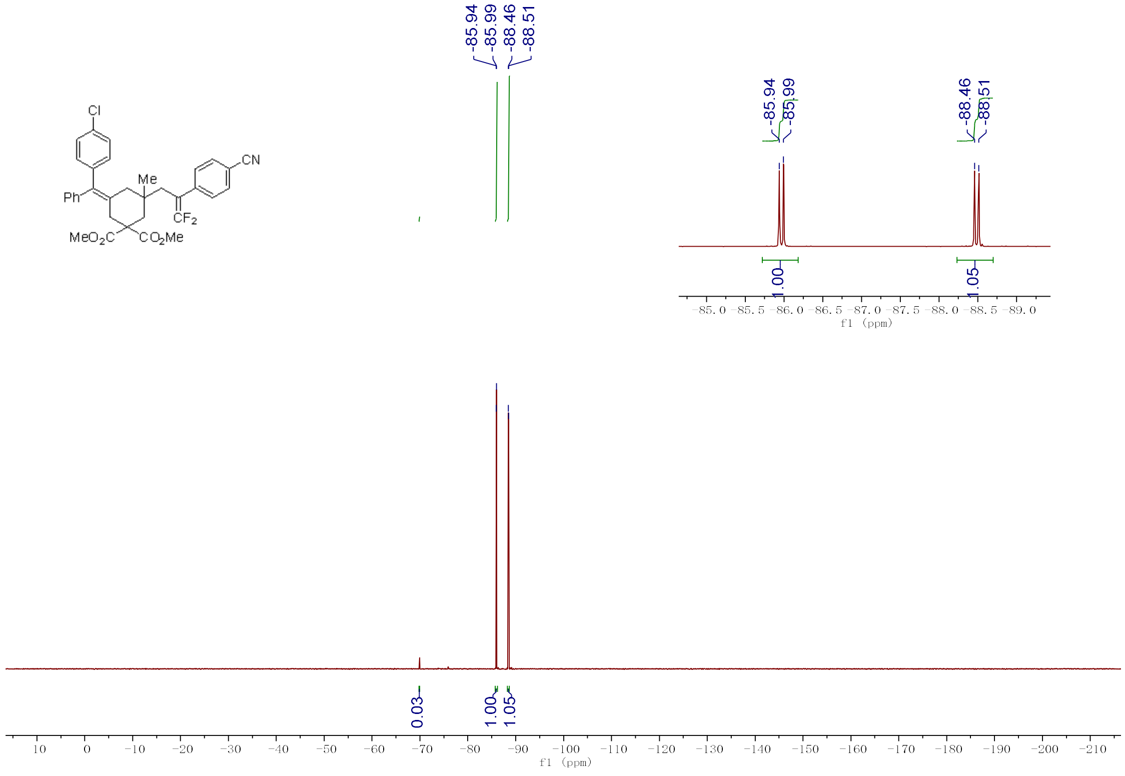

Supplementary figure 171. ^{19}F NMR of compound **54**

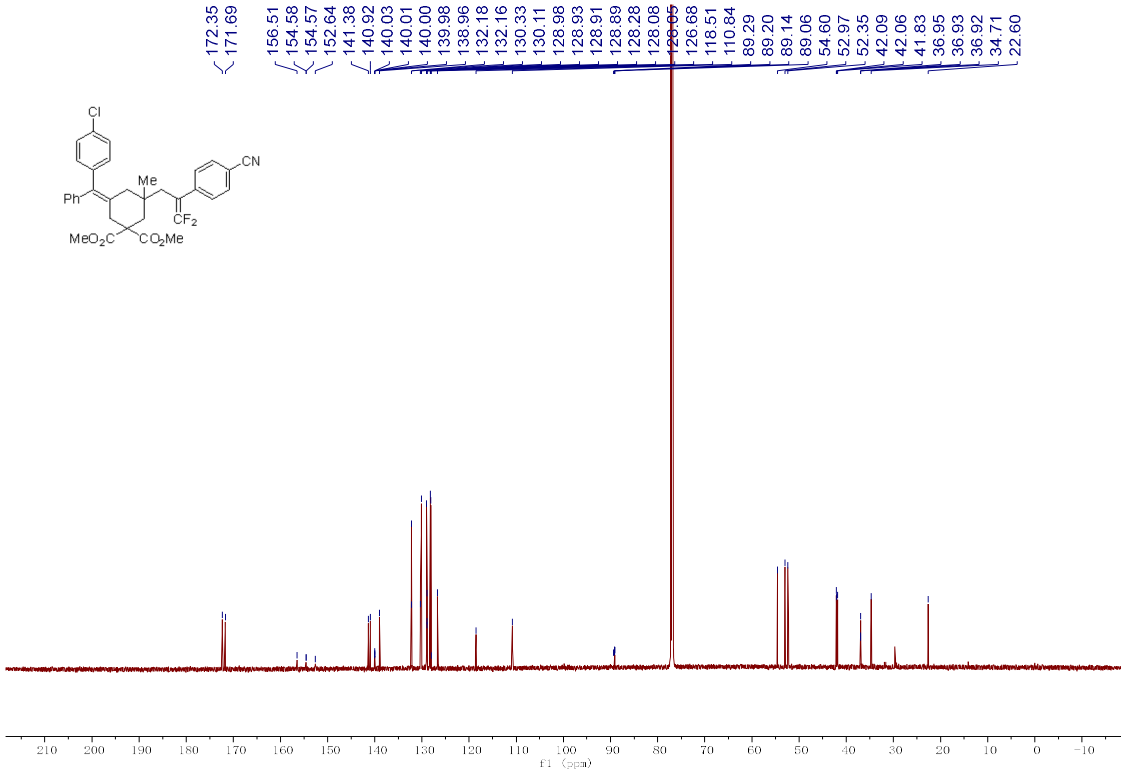

Supplementary figure 172. ^{13}C NMR of compound **54**

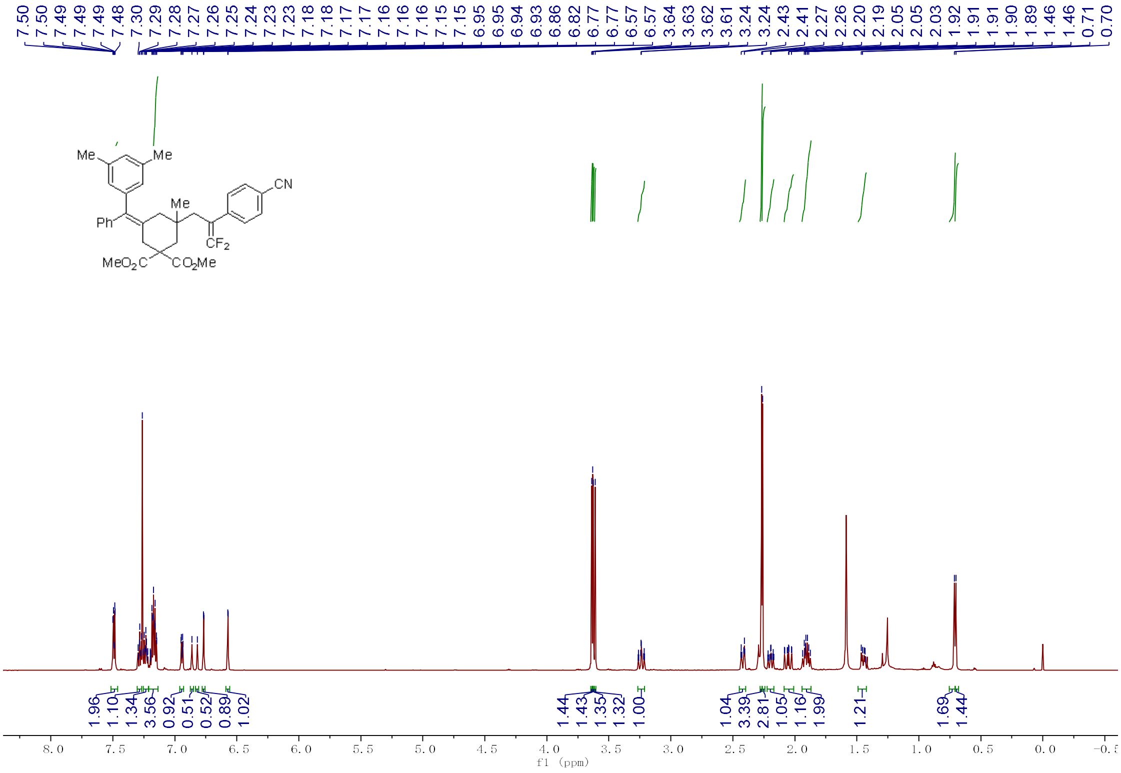

Supplementary figure 173. ^{1}H NMR of compound **55**

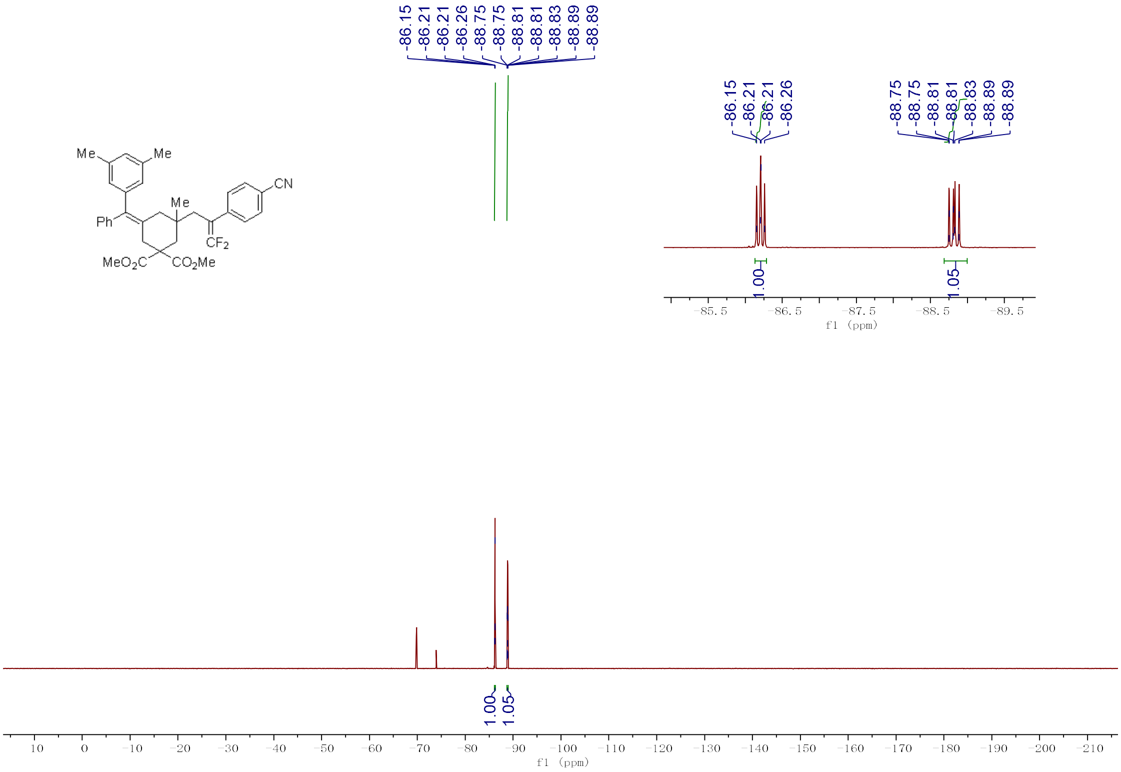

Supplementary figure 174. ^{19}F NMR of compound **55**

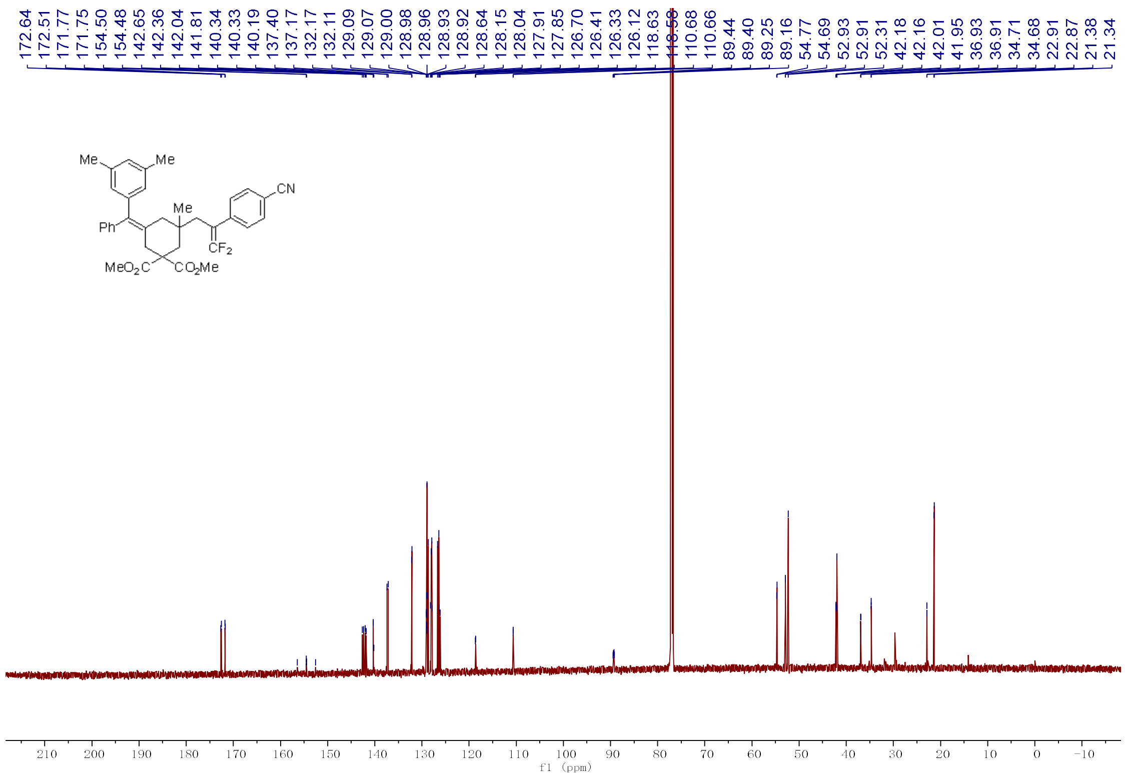

Supplementary figure 175. ^{13}C NMR of compound **55**

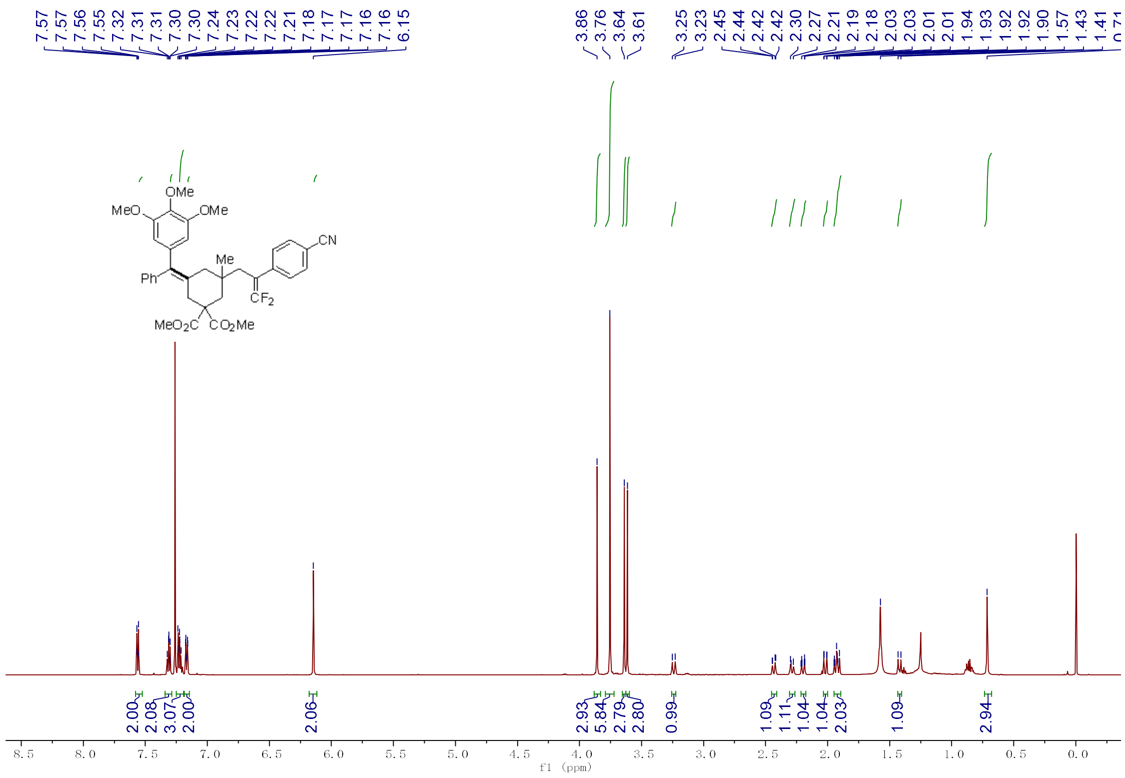

Supplementary figure 176. ^{1}H NMR of compound **56**

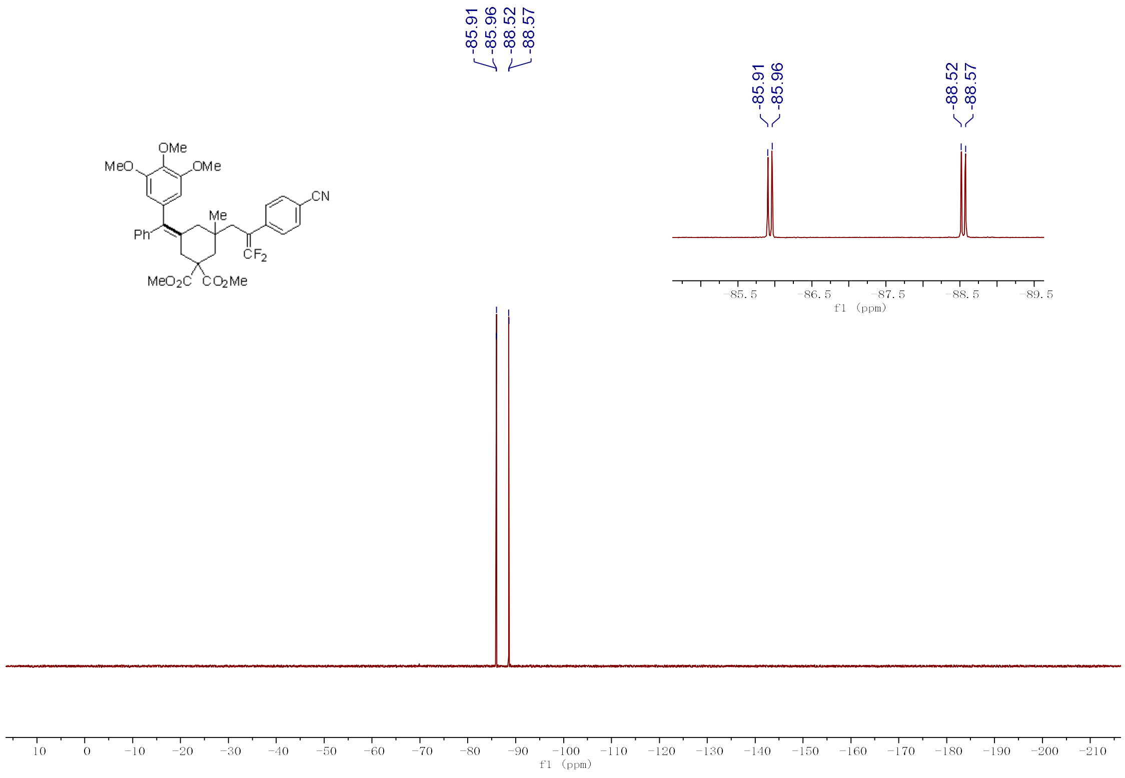

Supplementary figure 177. ^{19}F NMR of compound **56**

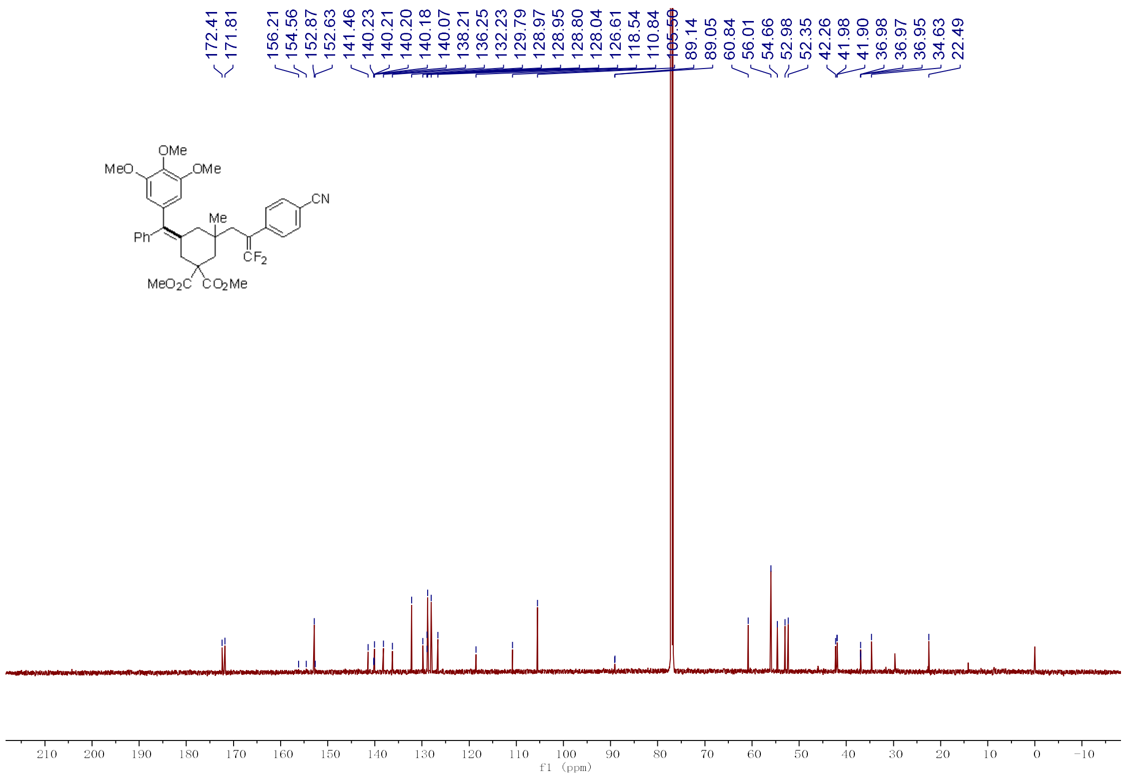

Supplementary figure 178. ^{13}C NMR of compound **56**

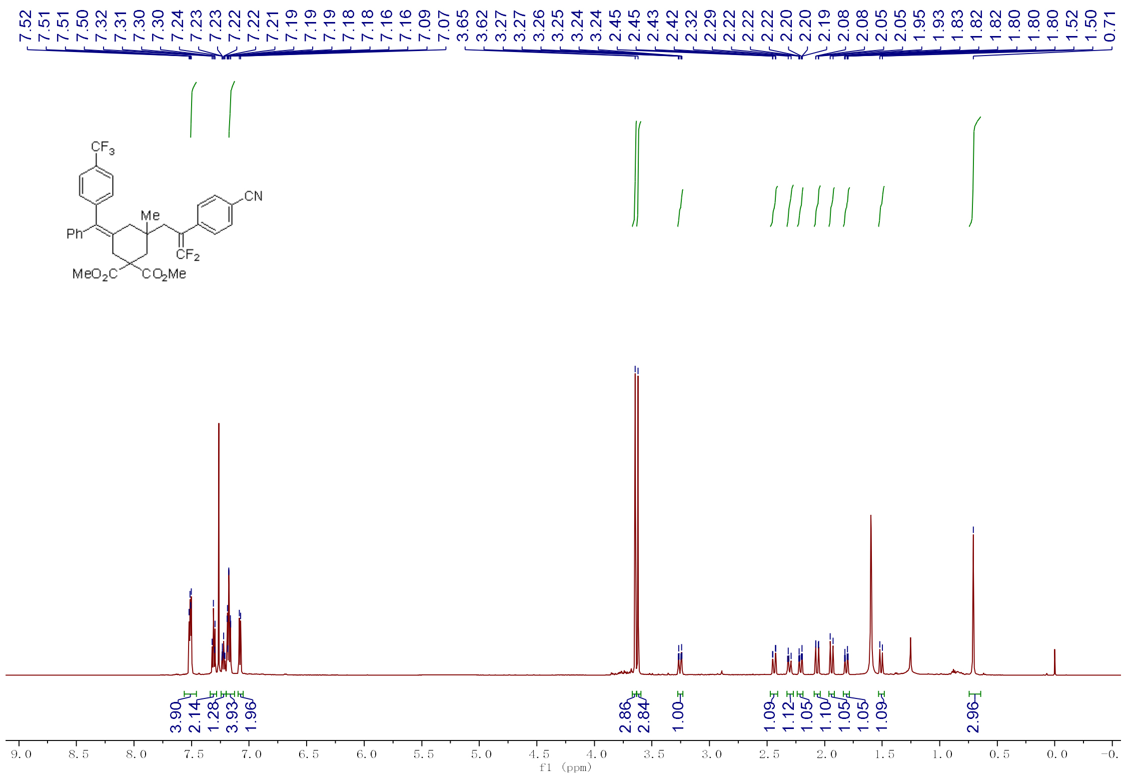

Supplementary figure 179. ^{1}H NMR of compound **58**

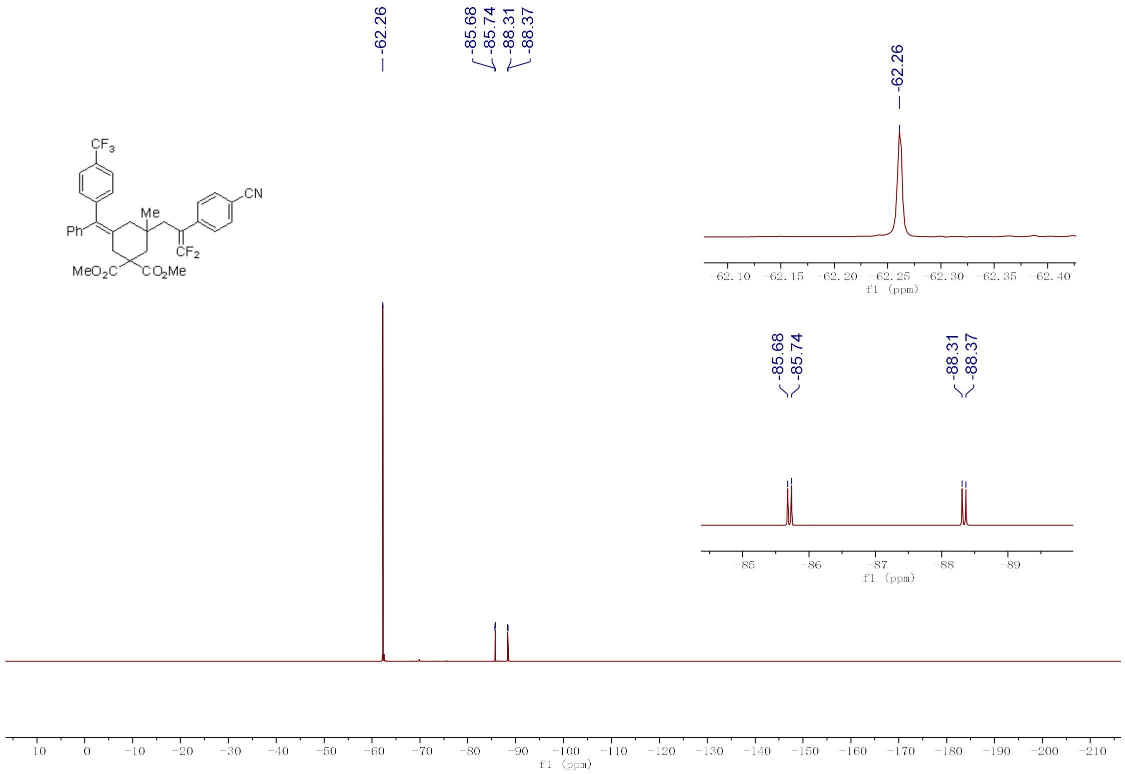

Supplementary figure 180. ^{19}F NMR of compound **58**

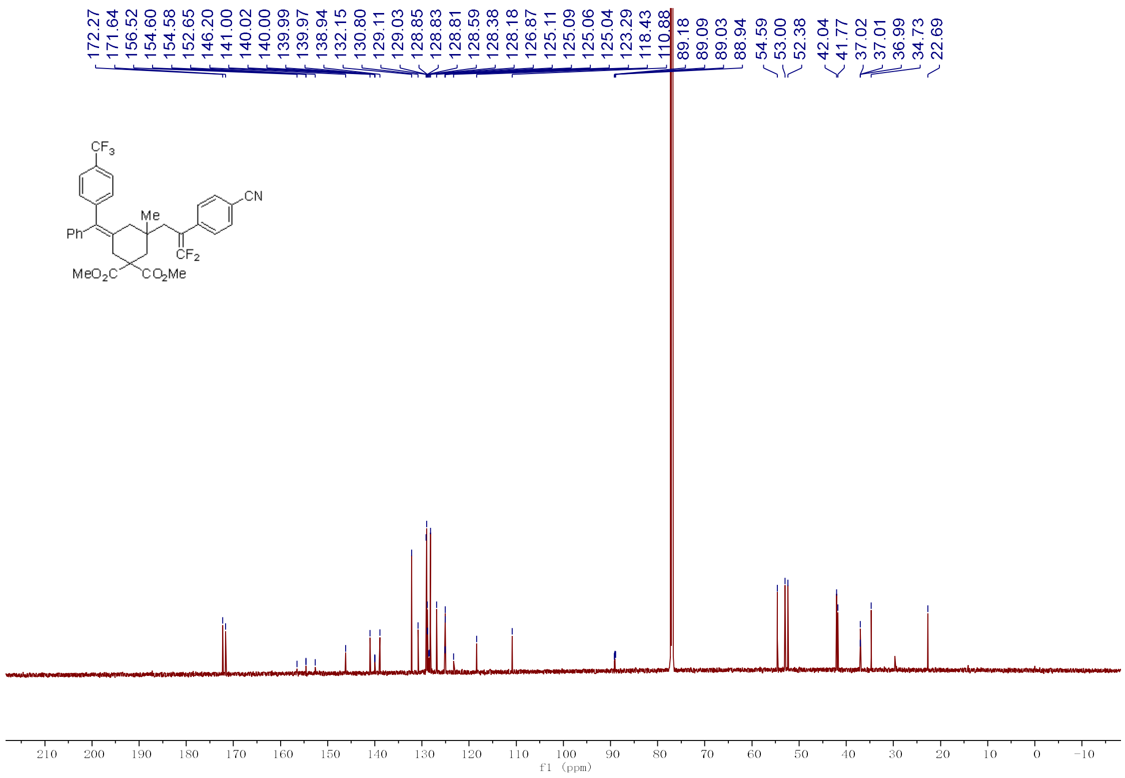

Supplementary figure 181. ^{13}C NMR of compound **58**

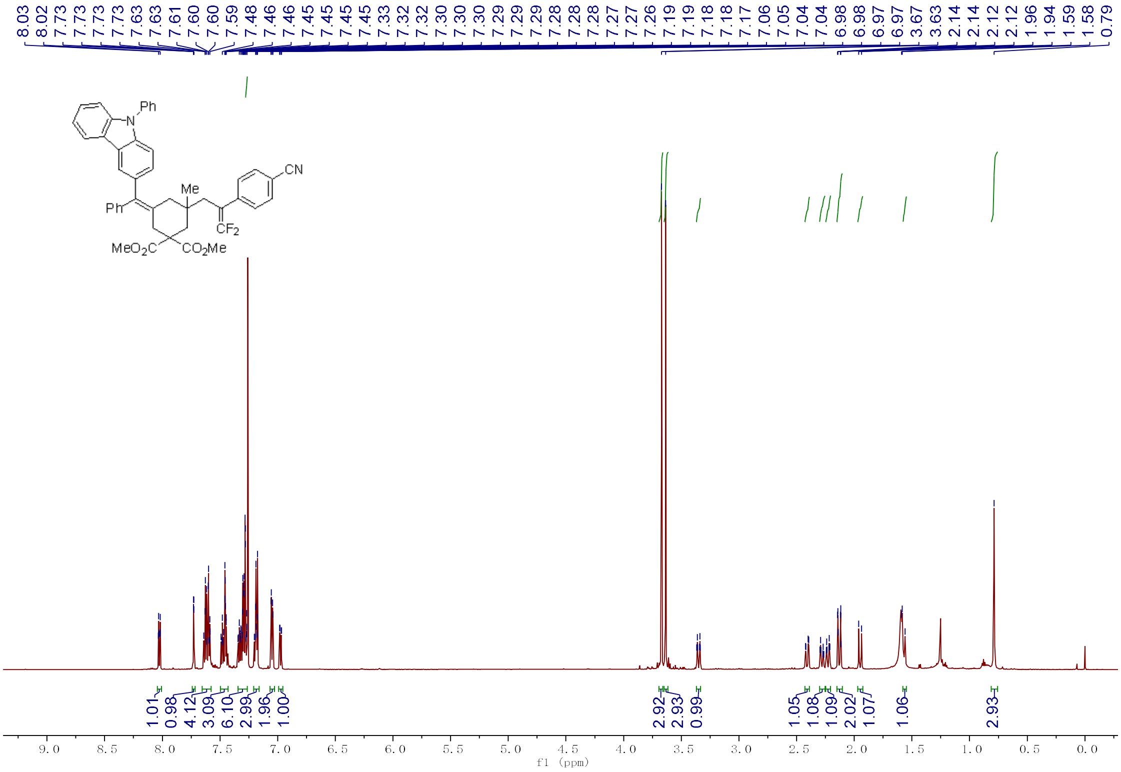

Supplementary figure 182. ^{1}H NMR of compound **59**

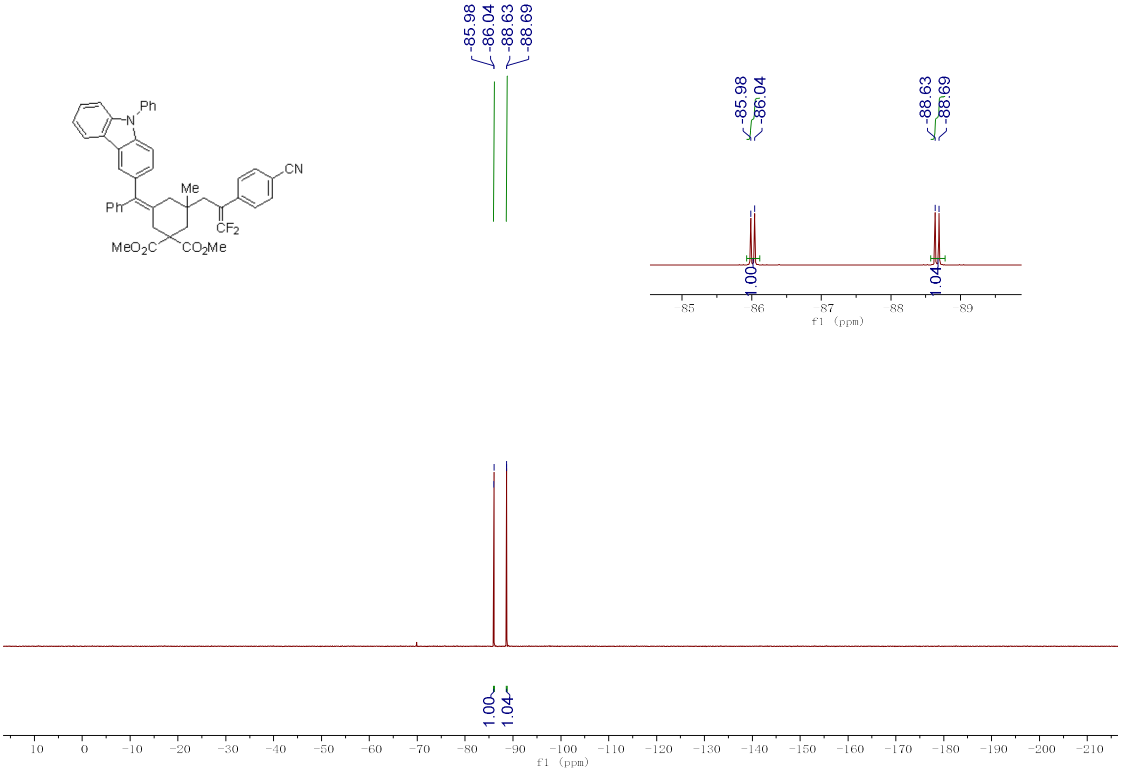

Supplementary figure 183. ^{19}F NMR of compound **59**

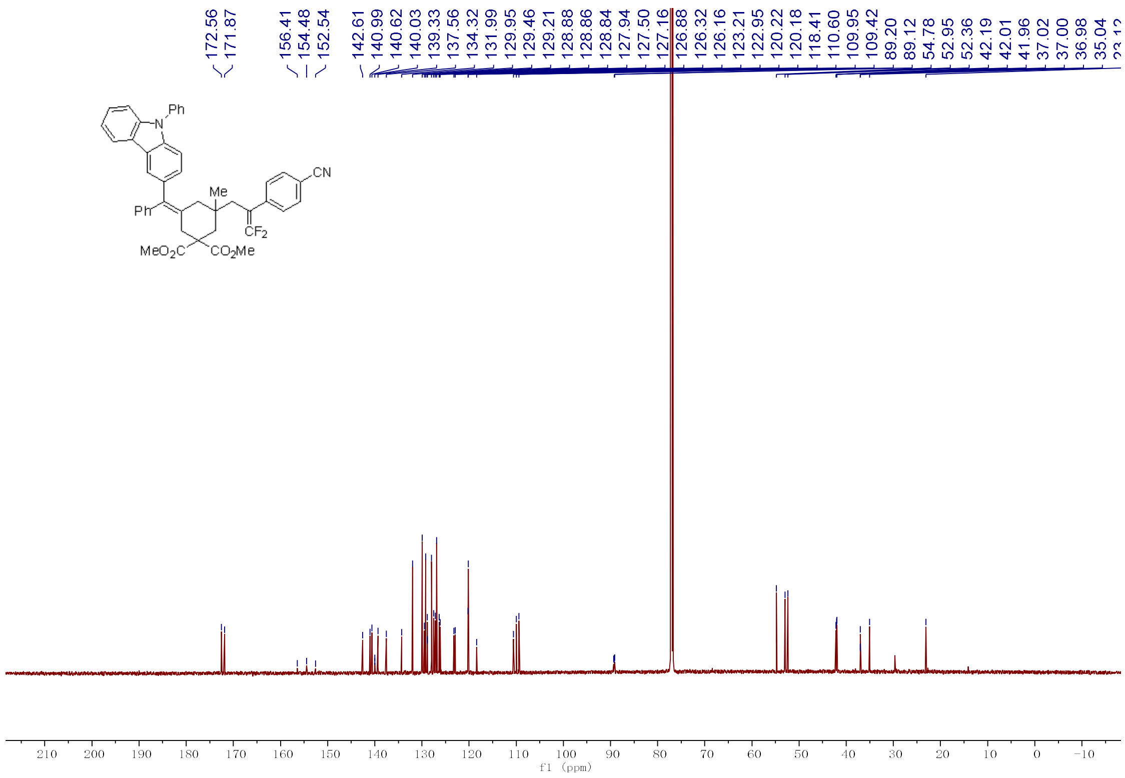

Supplementary figure 184. ^{13}C NMR of compound **59**

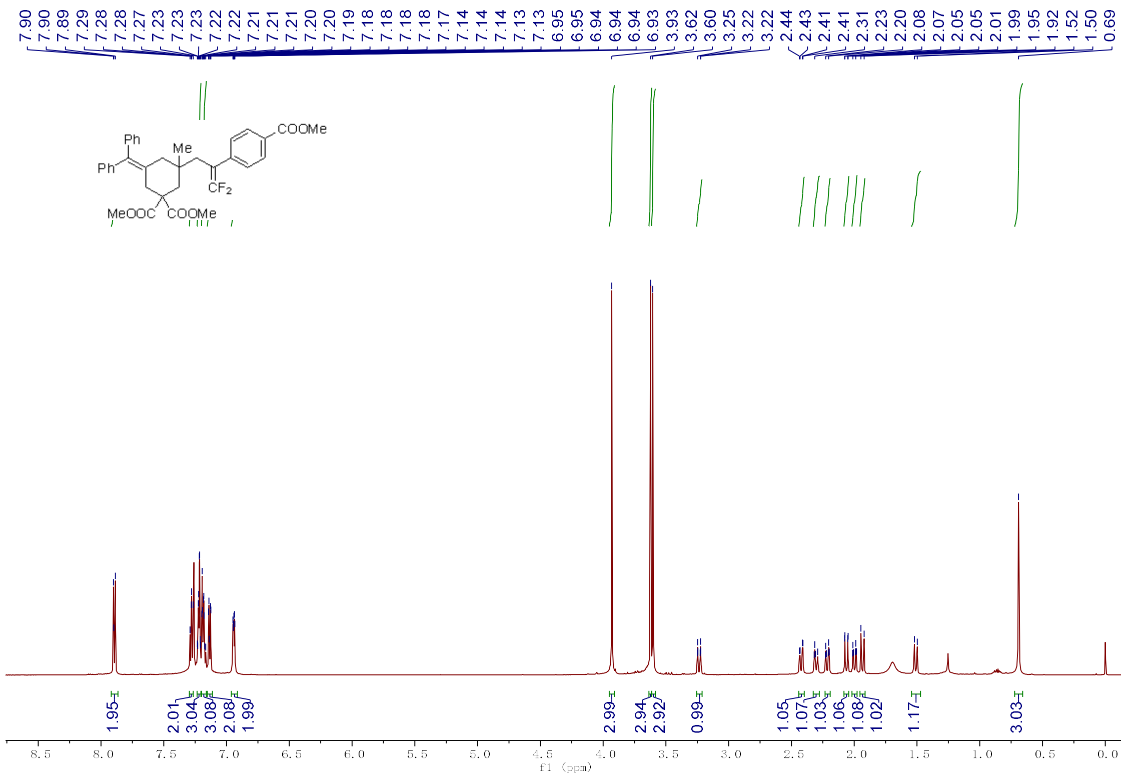

Supplementary figure 185. ^{1}H NMR of compound **60**

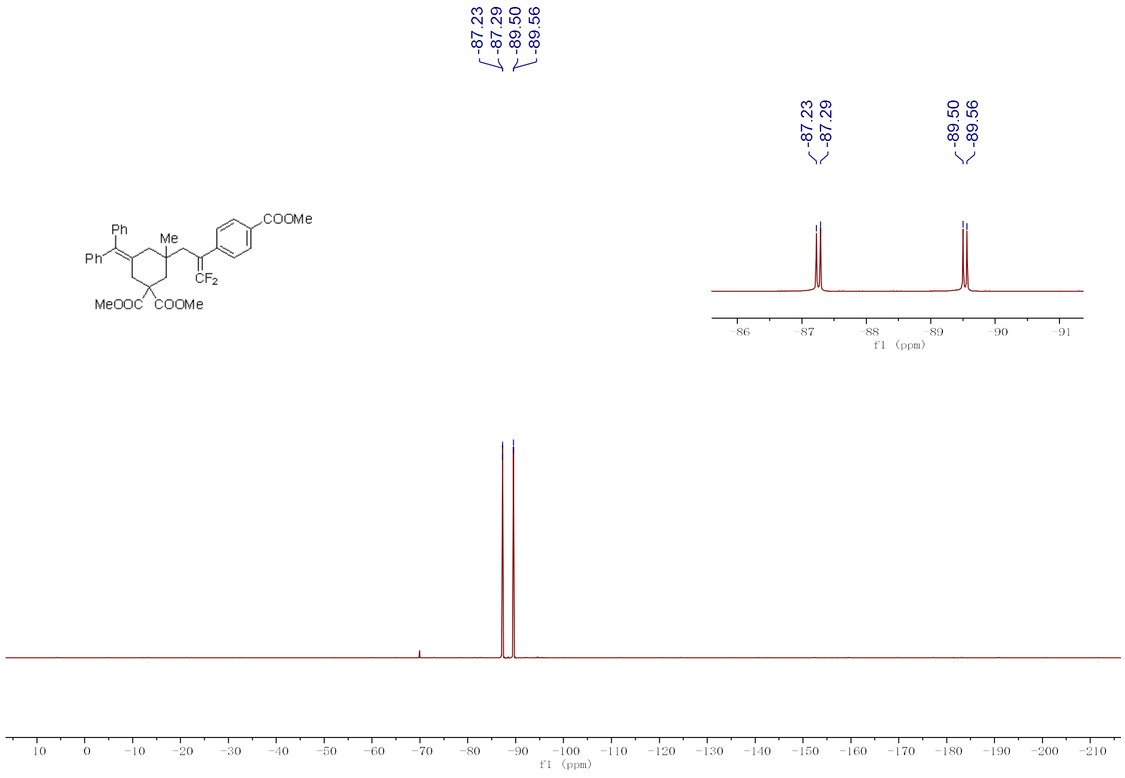

Supplementary figure 186. ^{19}F NMR of compound **60**

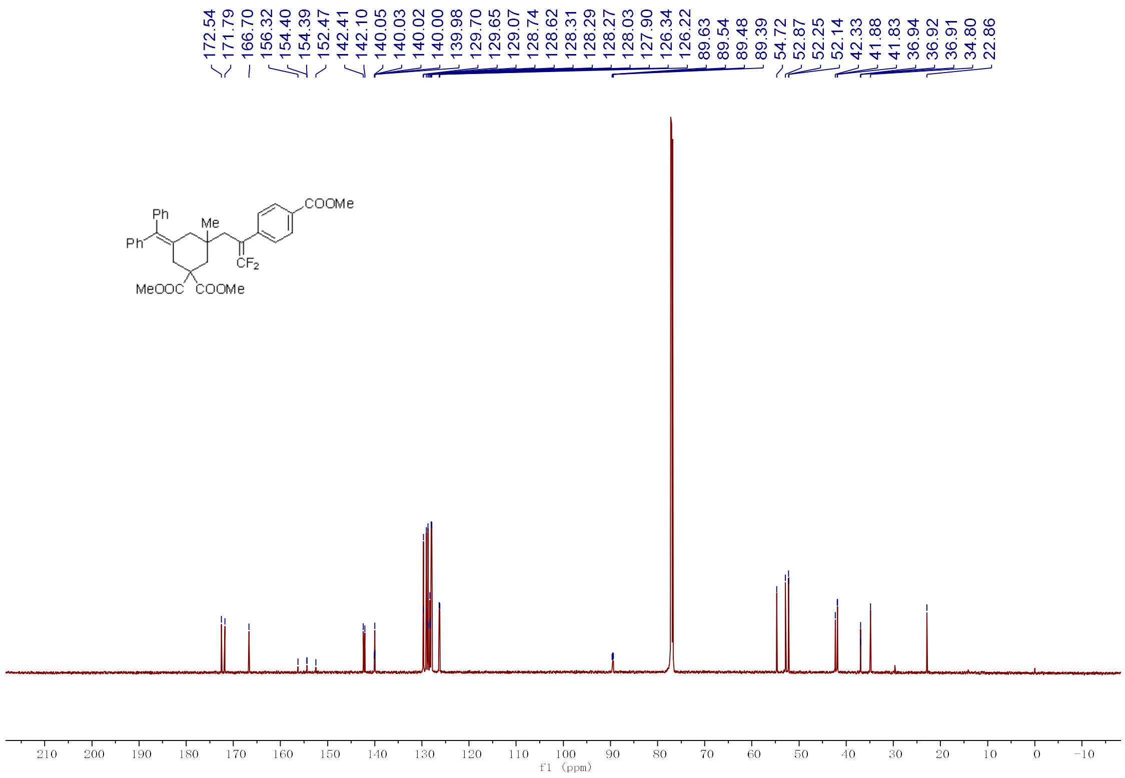

Supplementary figure 187. ^{13}C NMR of compound **60**

61

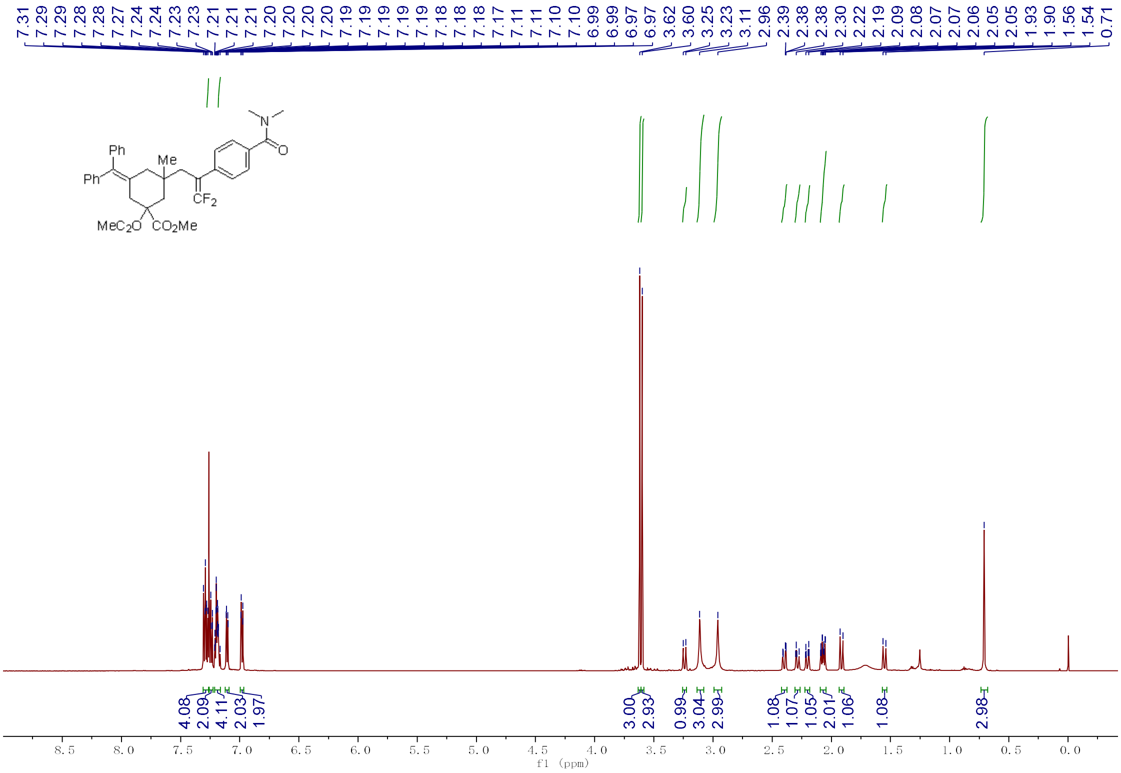

Supplementary figure 188. ^{1}H NMR of compound **61**

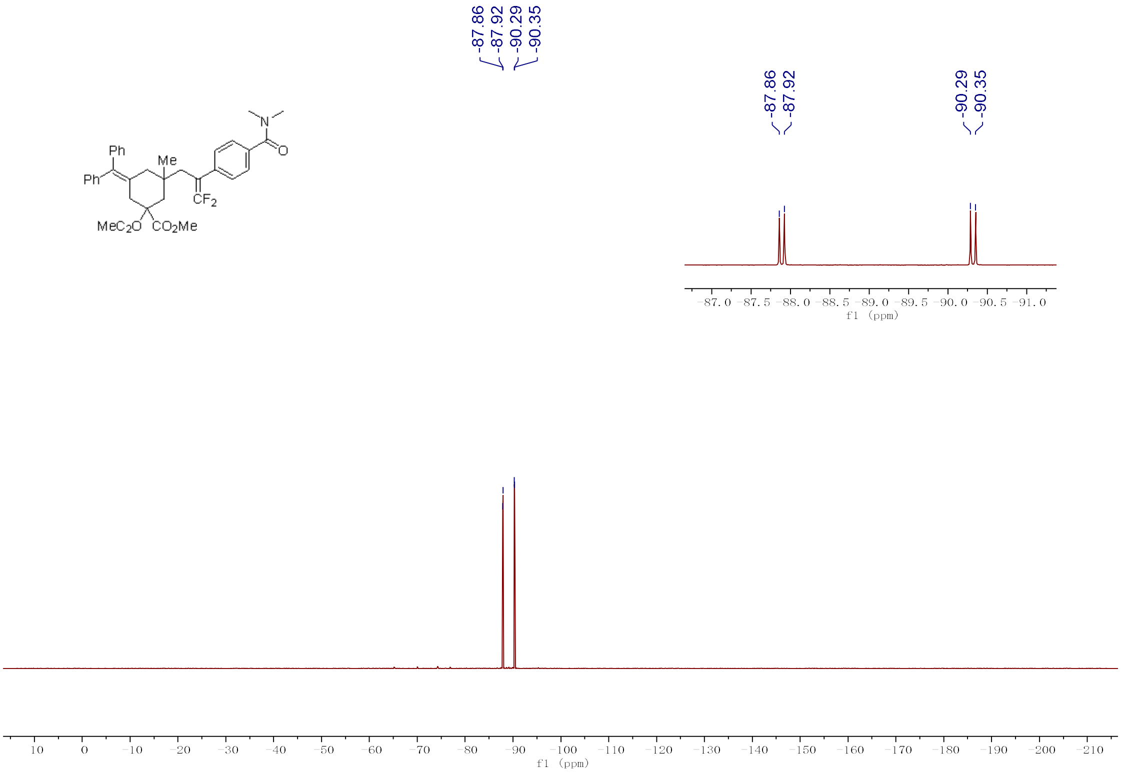

Supplementary figure 189. ^{19}F NMR of compound **61**

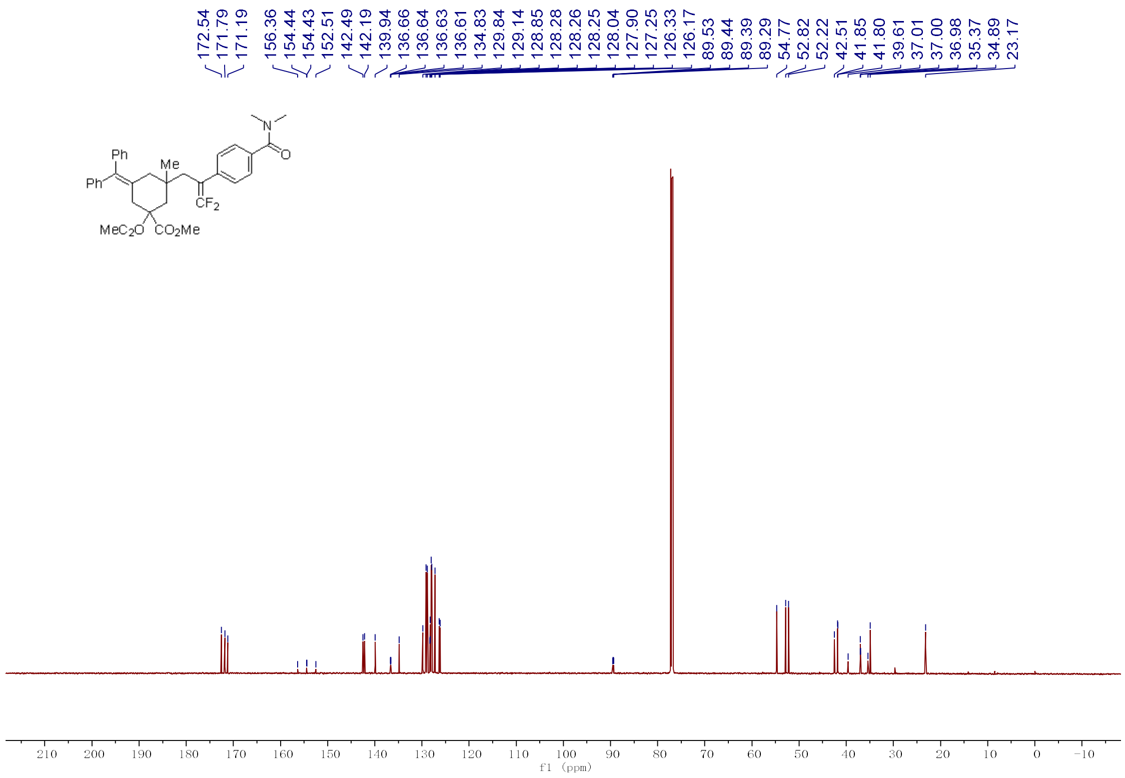

Supplementary figure 190. ^{13}C NMR of compound **61**

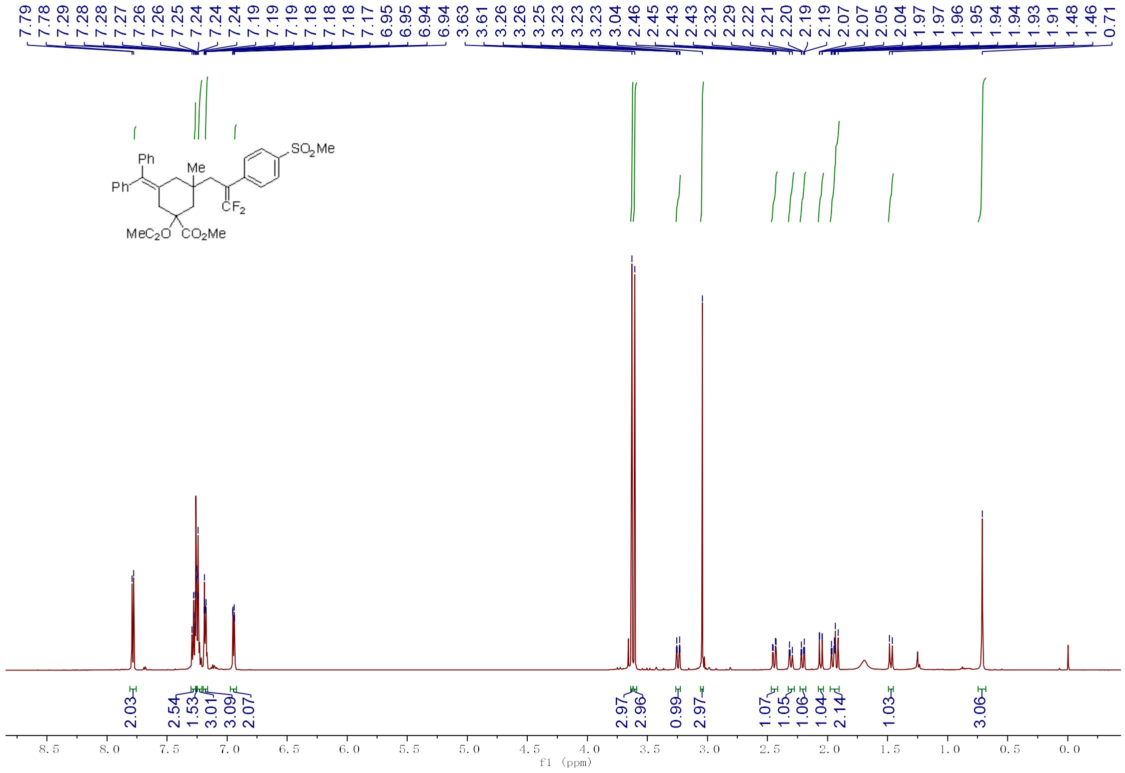

Supplementary figure 191. ^{1}H NMR of compound **62**

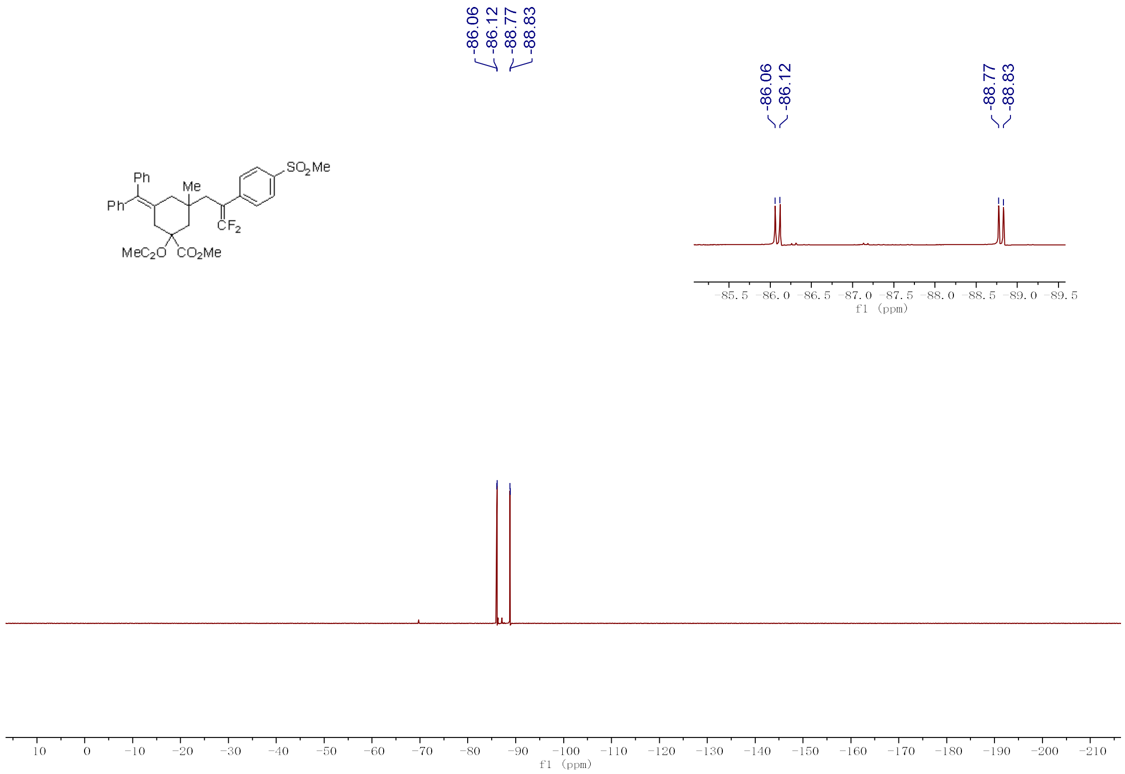

Supplementary figure 192. ^{19}F NMR of compound **62**

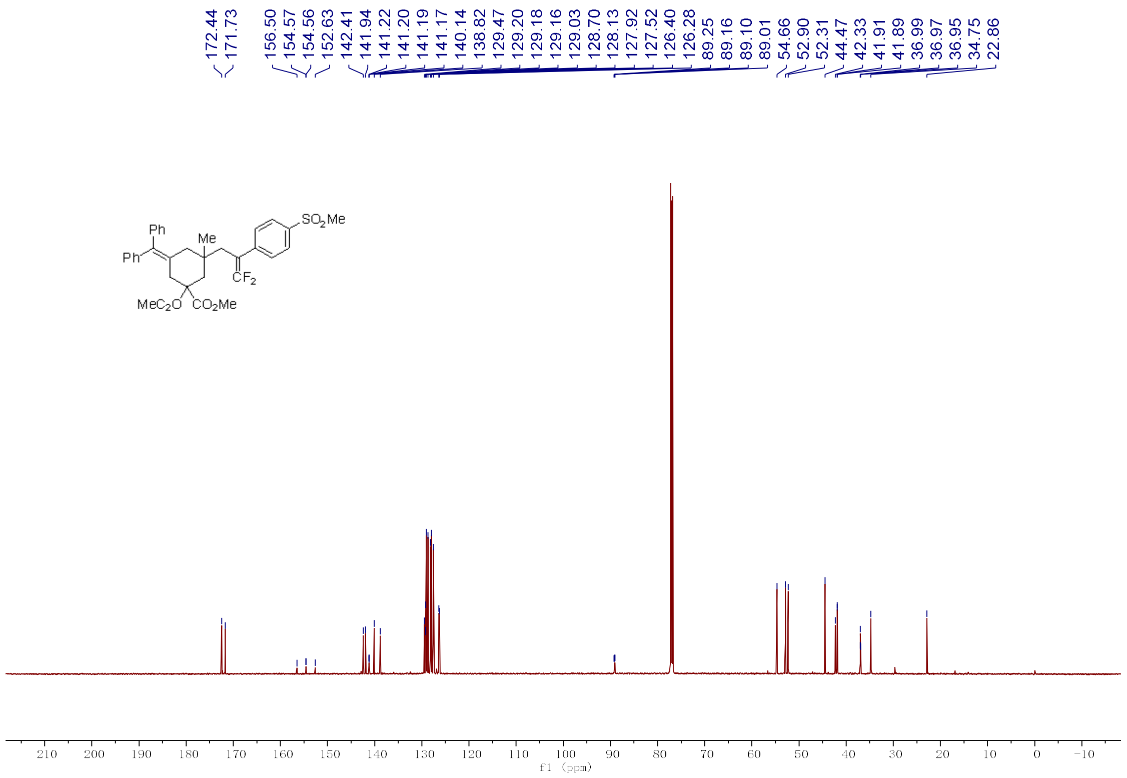

Supplementary figure 193. ^{13}C NMR of compound **62**

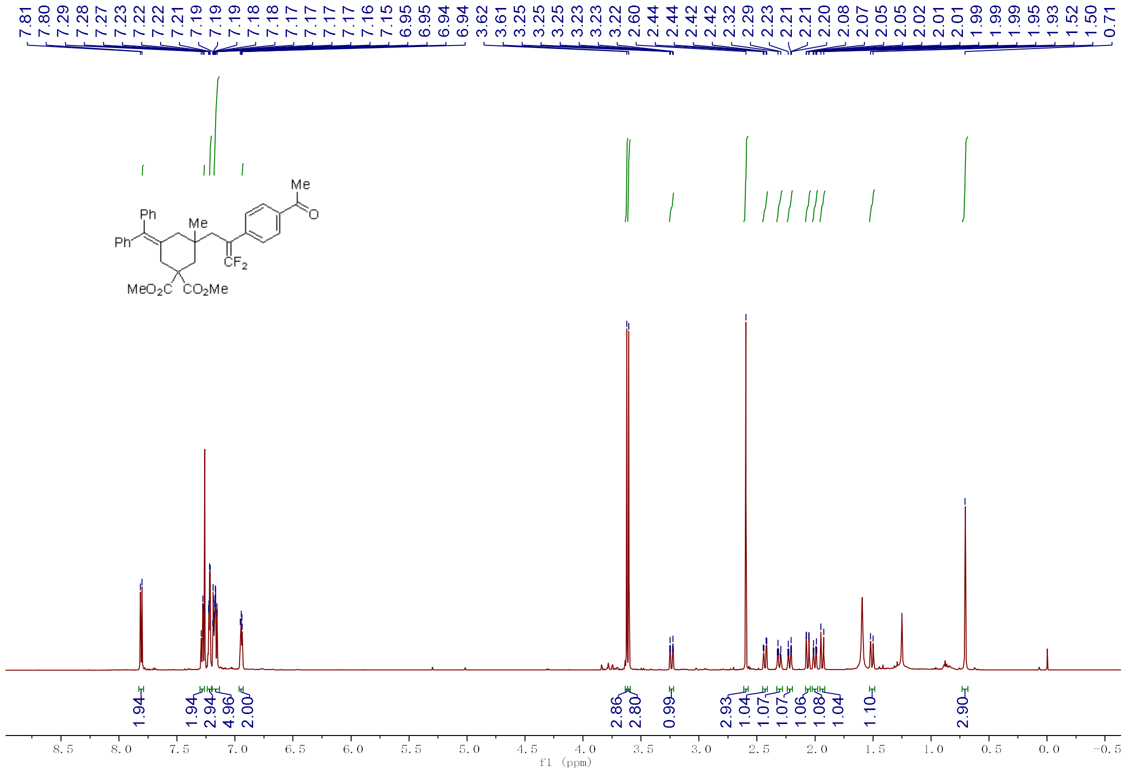

Supplementary figure 194. ^{1}H NMR of compound **63**

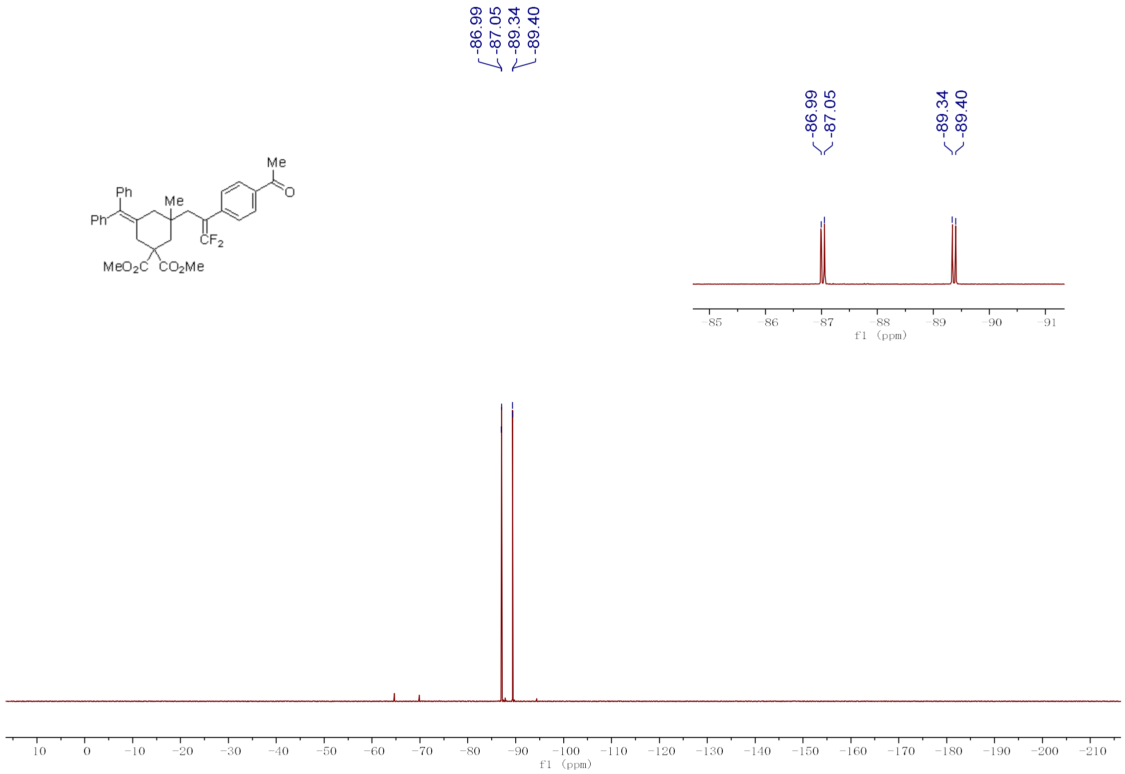

Supplementary figure 195. ^{19}F NMR of compound **63**

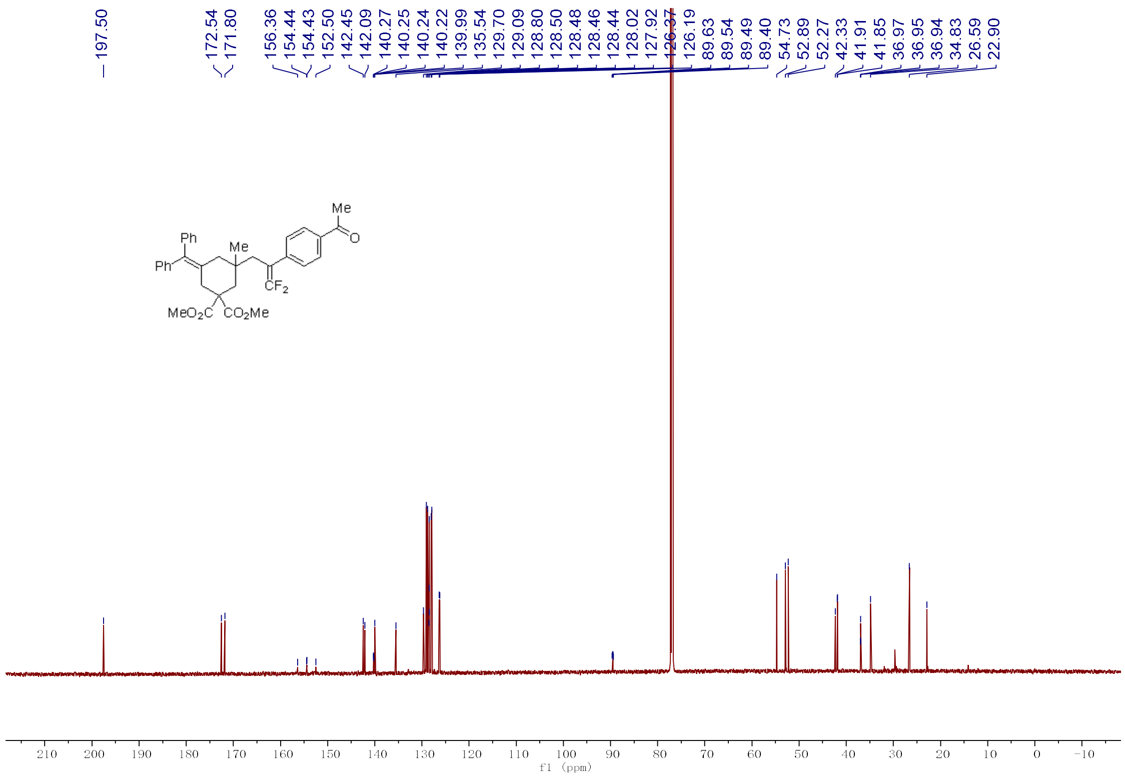

Supplementary figure 196. ^{13}C NMR of compound **63**

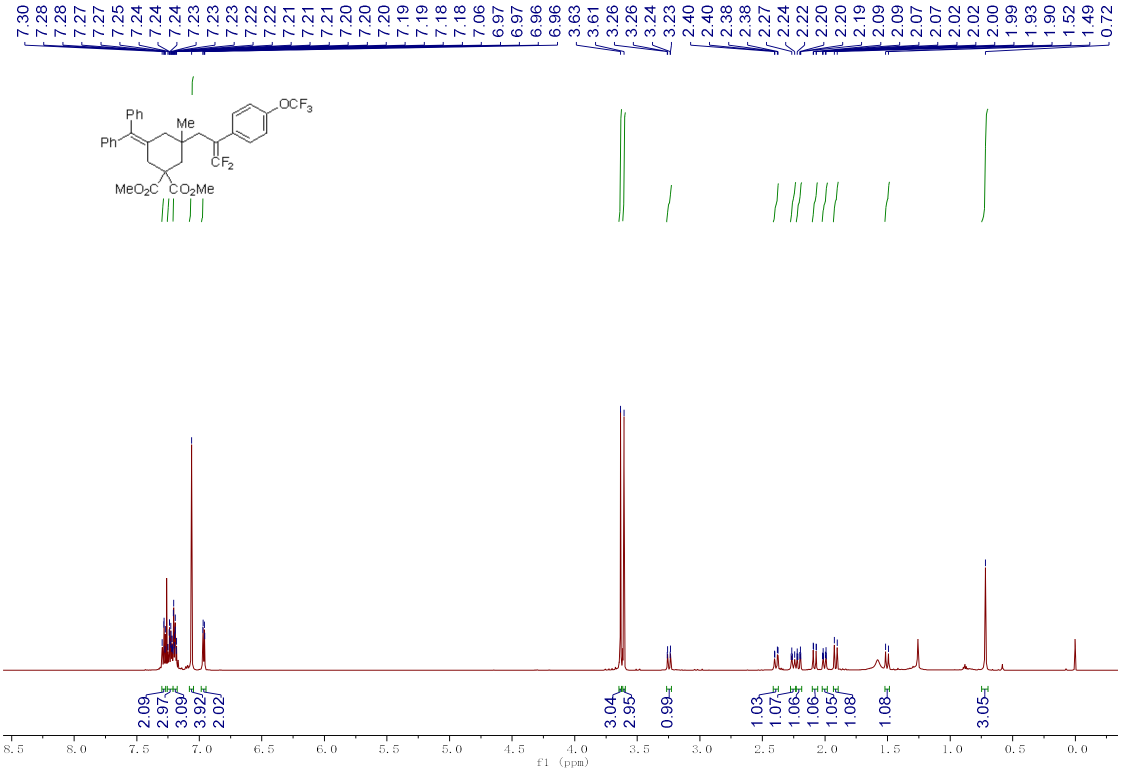

Supplementary figure 197. ^{1}H NMR of compound **64**

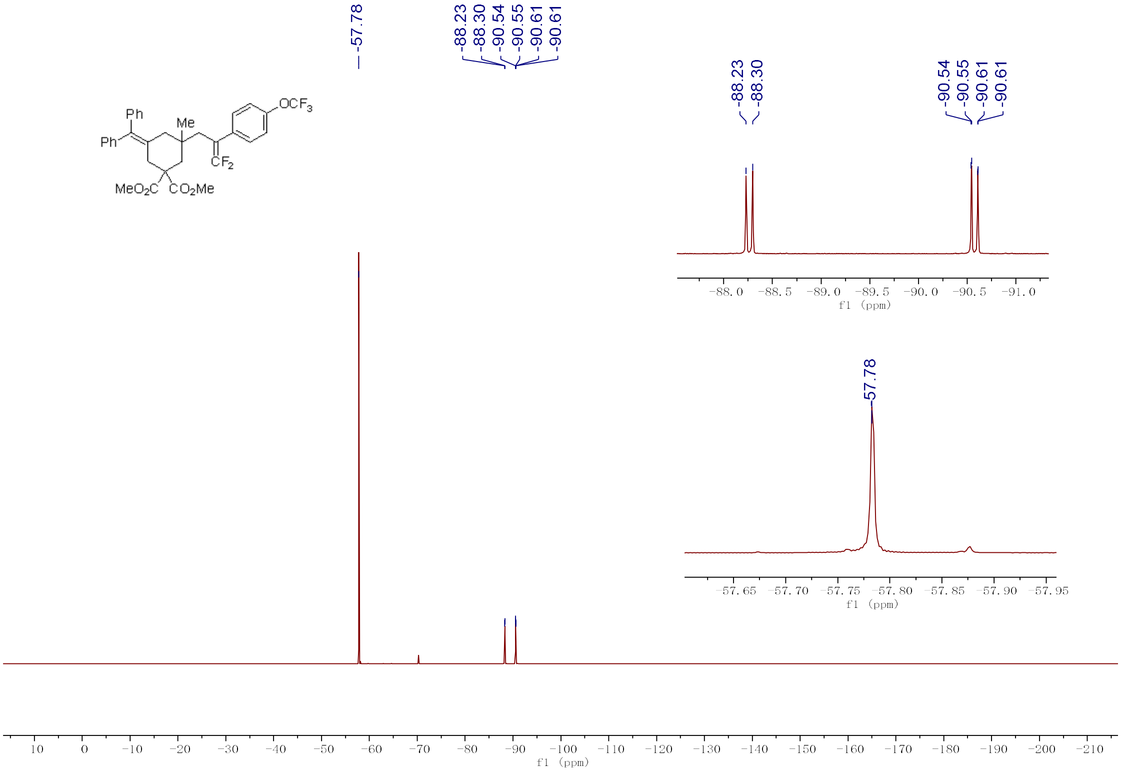

Supplementary figure 198. ^{19}F NMR of compound **64**

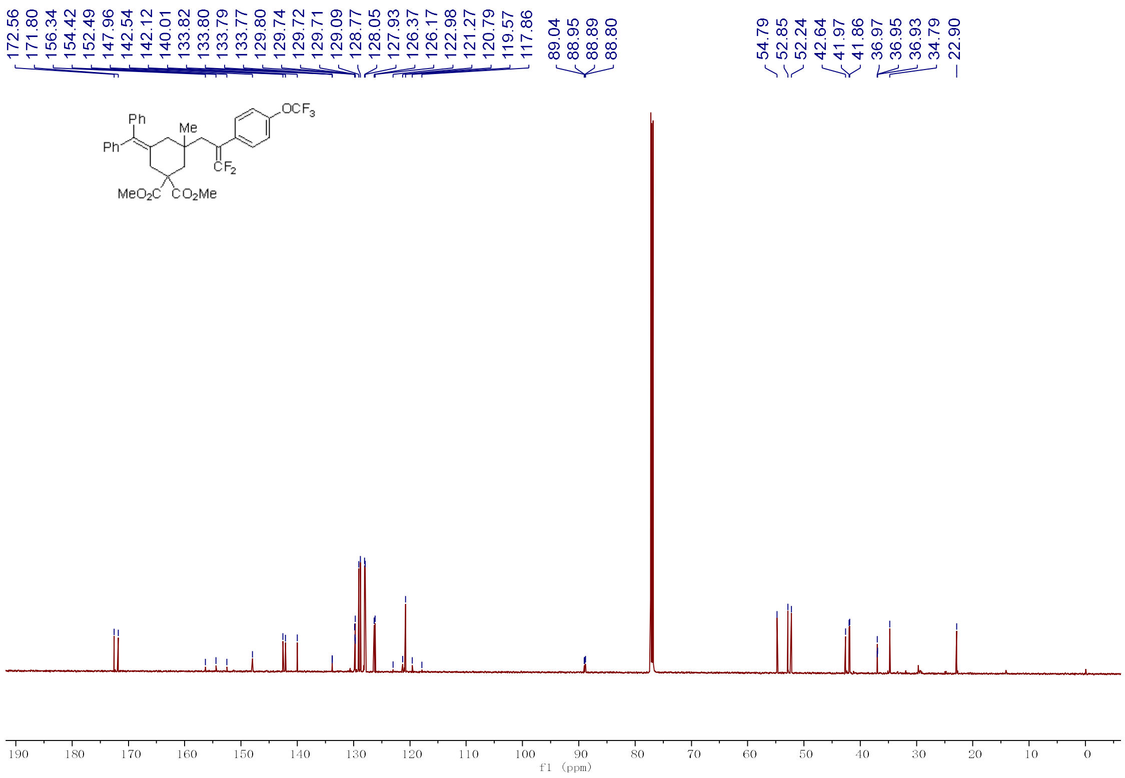

Supplementary figure 199. ^{13}C NMR of compound **64**

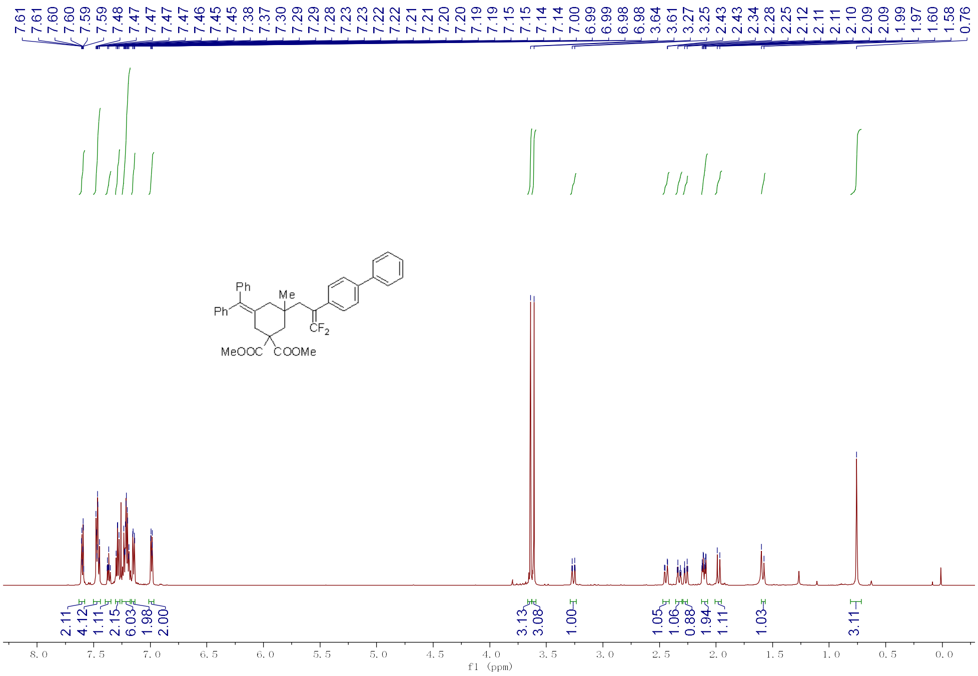

Supplementary figure 200. ^{1}H NMR of compound **65**

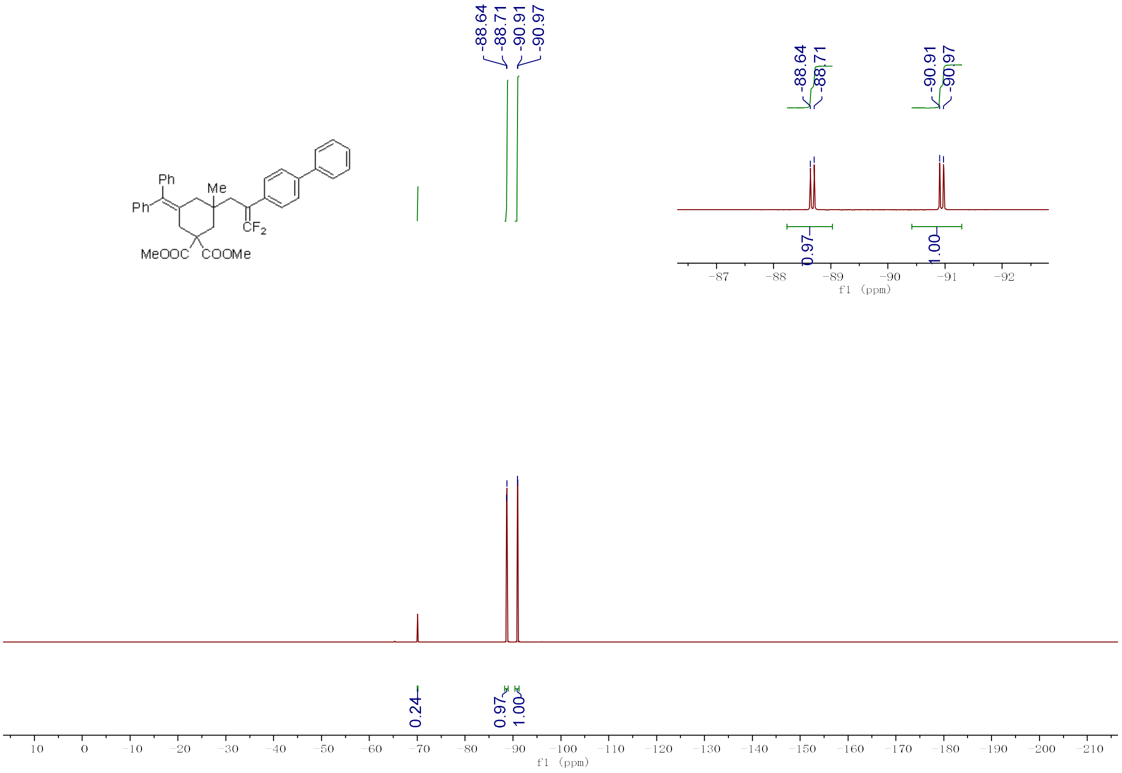

Supplementary figure 201. ^{19}F NMR of compound **65**

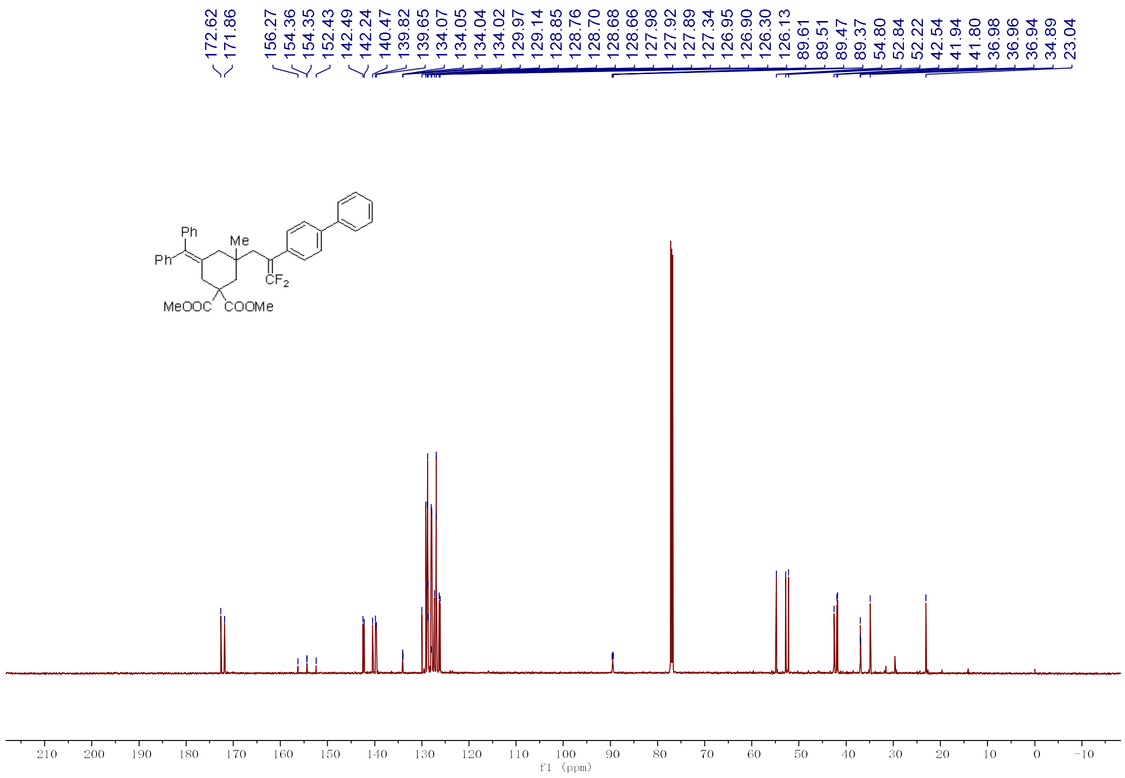

Supplementary figure 202. ^{13}C NMR of compound **65**

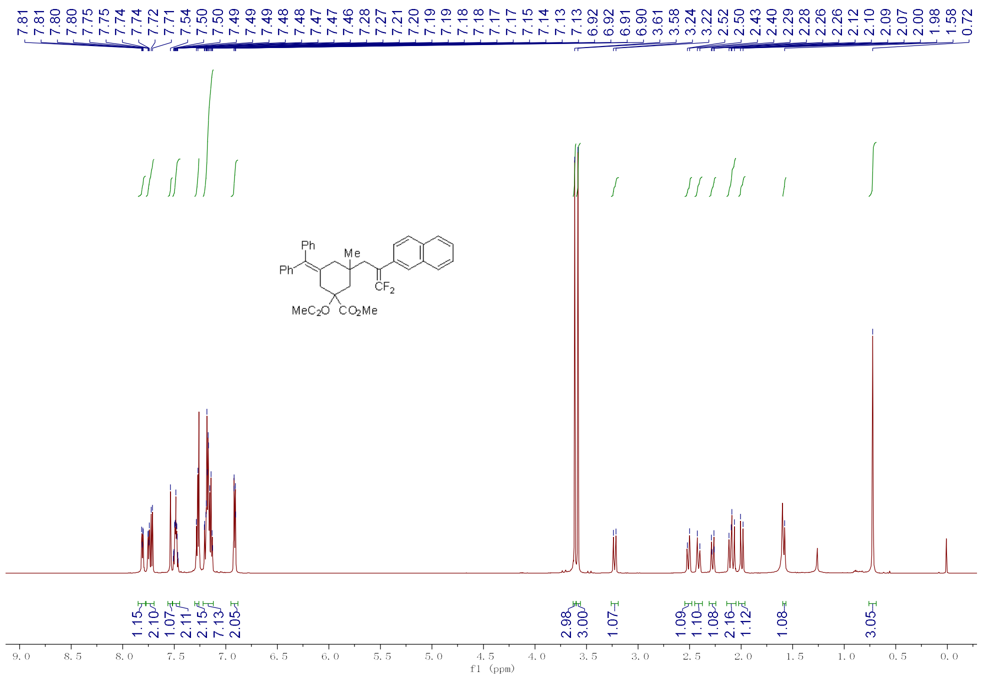

Supplementary figure 203. ^{1}H NMR of compound **66**

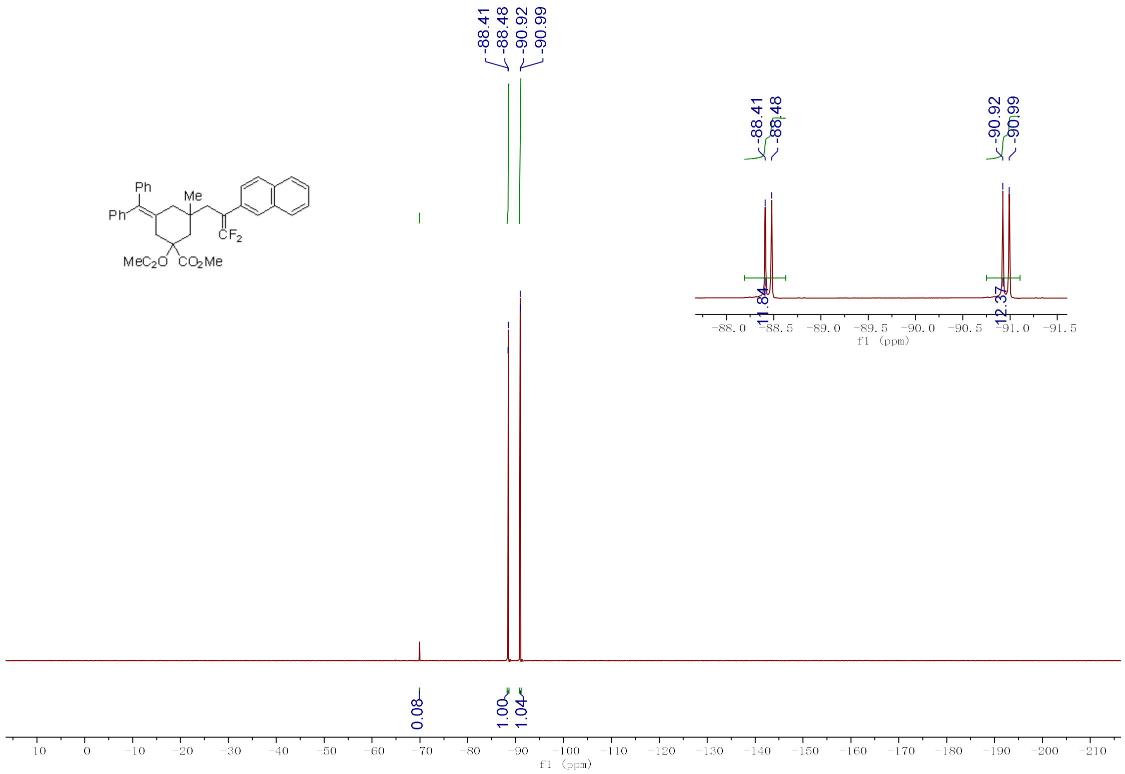

Supplementary figure 204. ^{19}F NMR of compound **66**

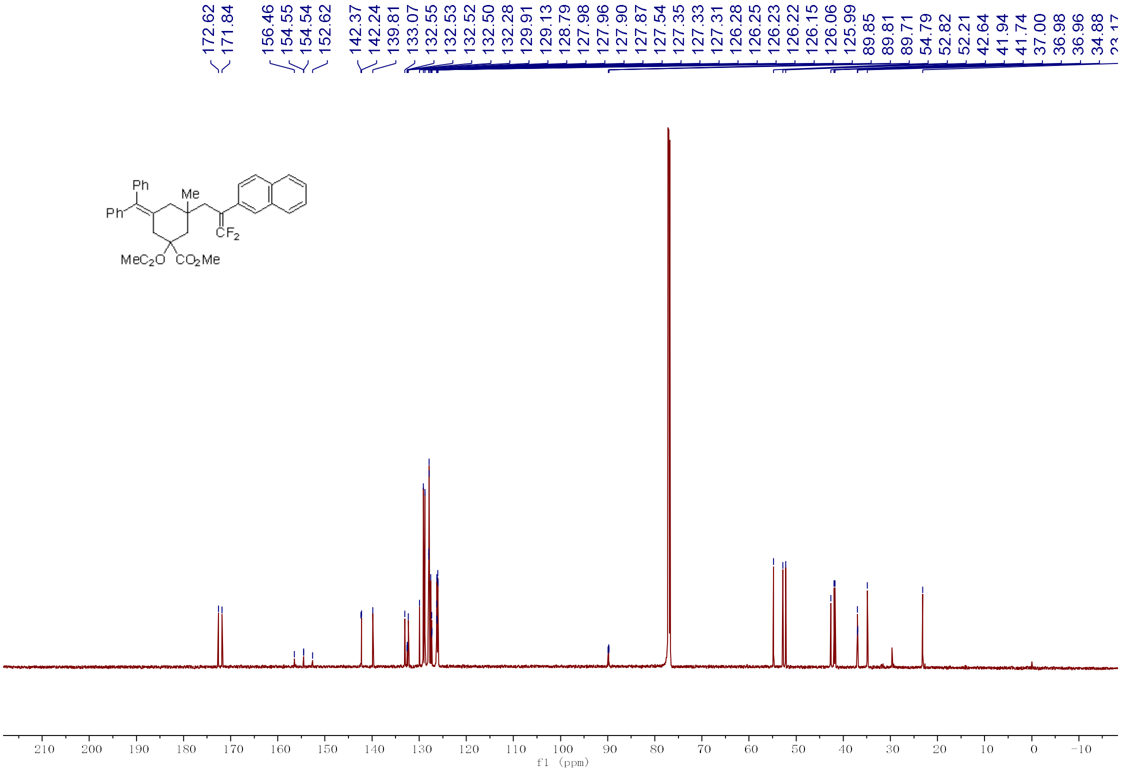

Supplementary figure 205. ^{13}C NMR of compound **66**

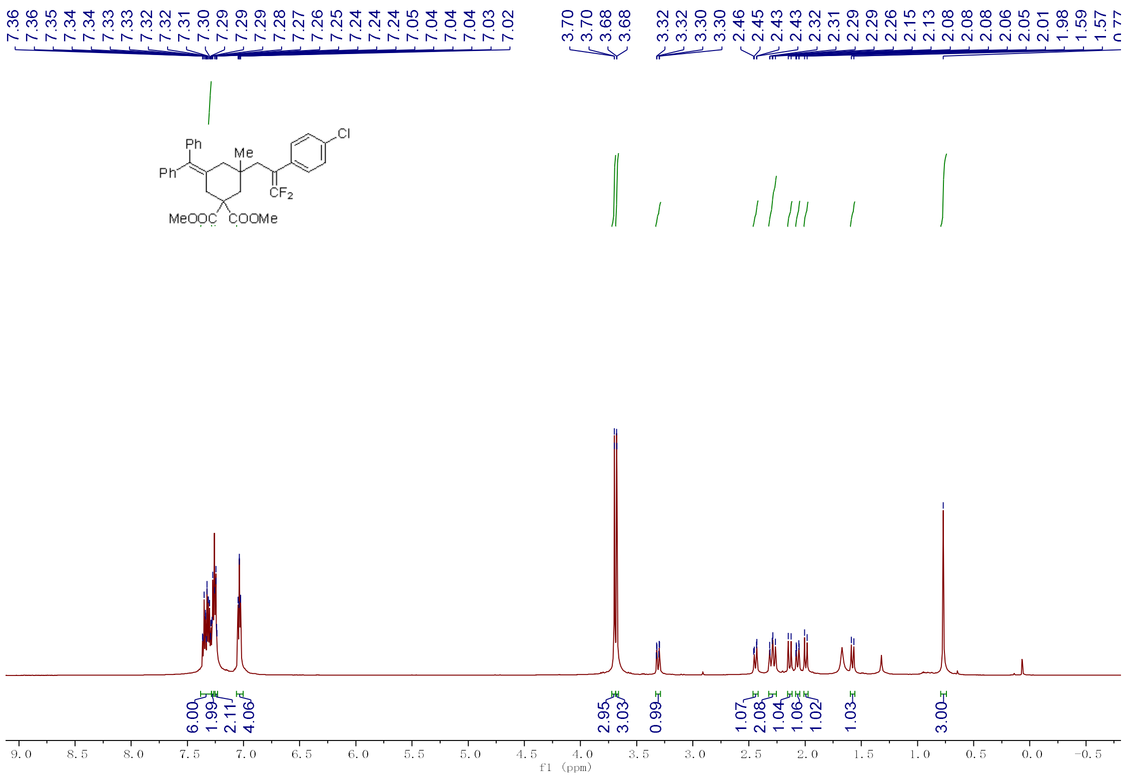

Supplementary figure 206. ^{1}H NMR of compound **67**

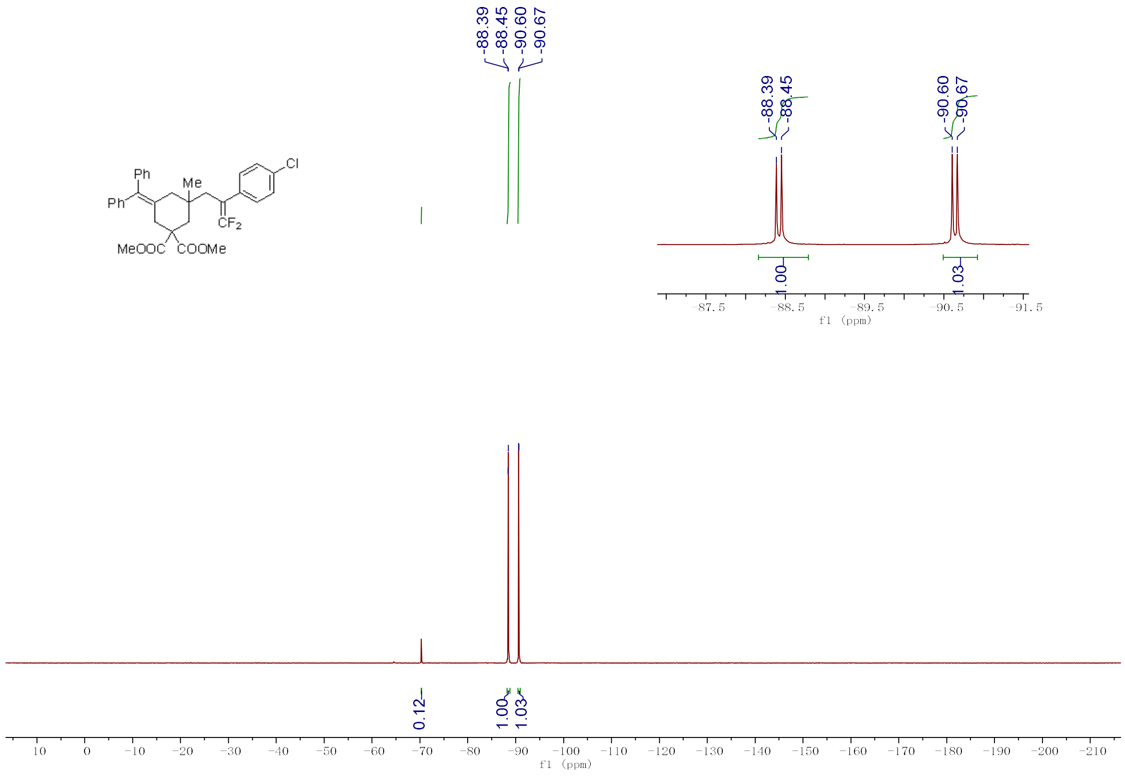

Supplementary figure 207. ^{19}F NMR of compound **67**

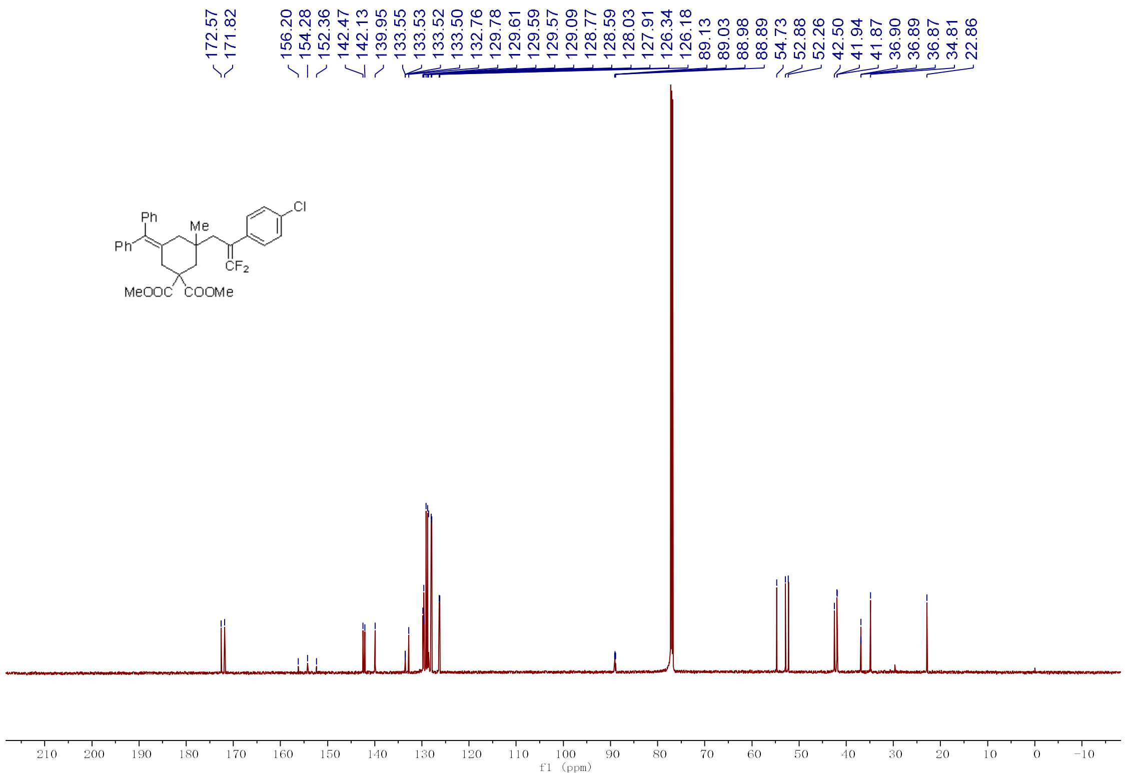

Supplementary figure 208. ^{13}C NMR of compound **67**

68

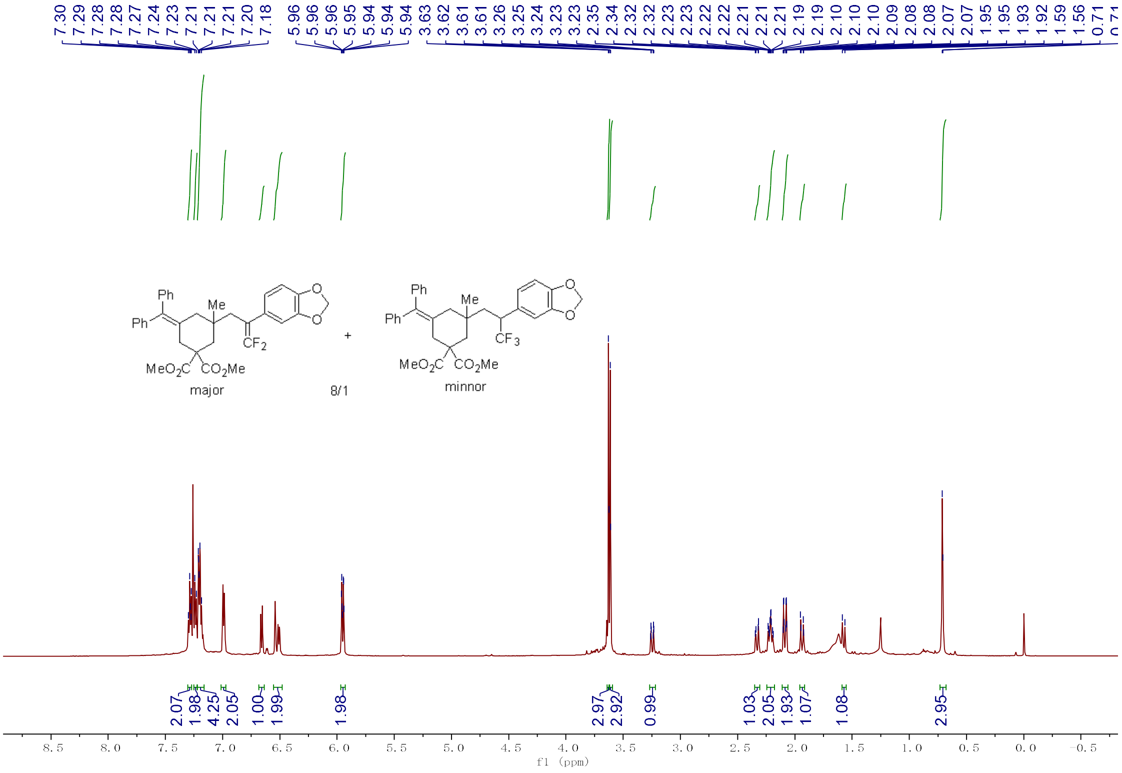

Supplementary figure 209. ^{1}H NMR of compound **68**

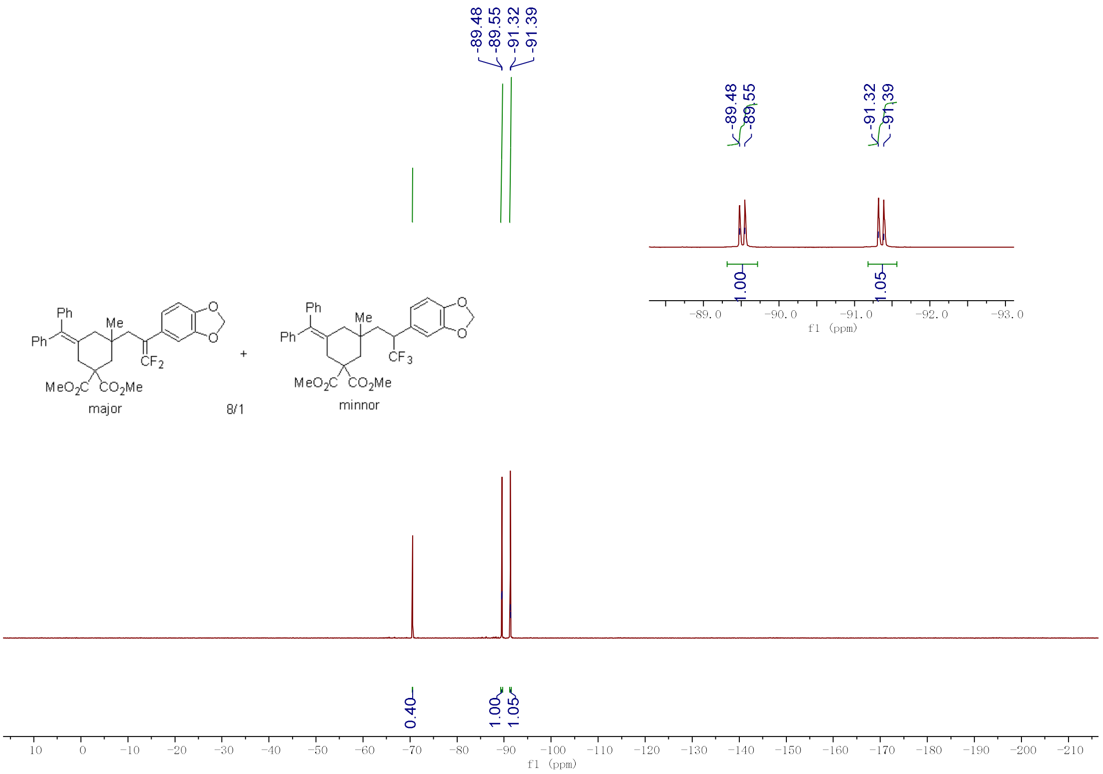

Supplementary figure 210. ^{19}F NMR of compound **68**

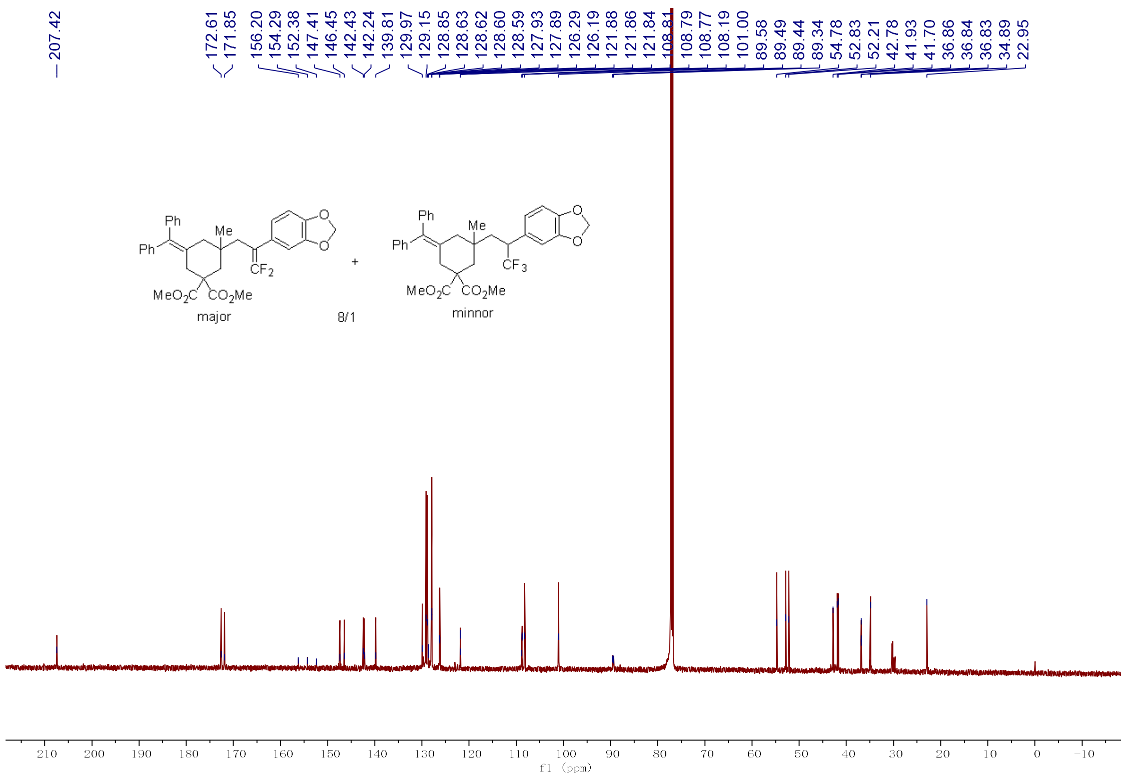

Supplementary figure 211. ^{13}C NMR of compound **68**

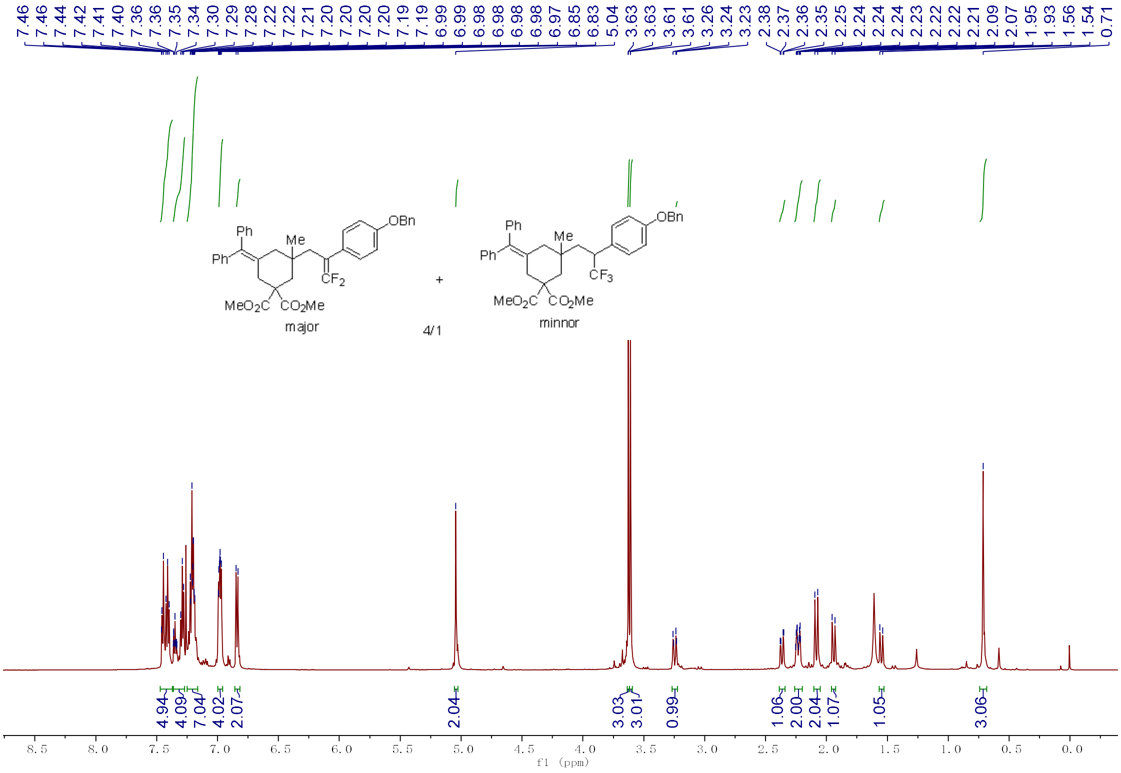

Supplementary figure 212. ^{1}H NMR of compound **69**

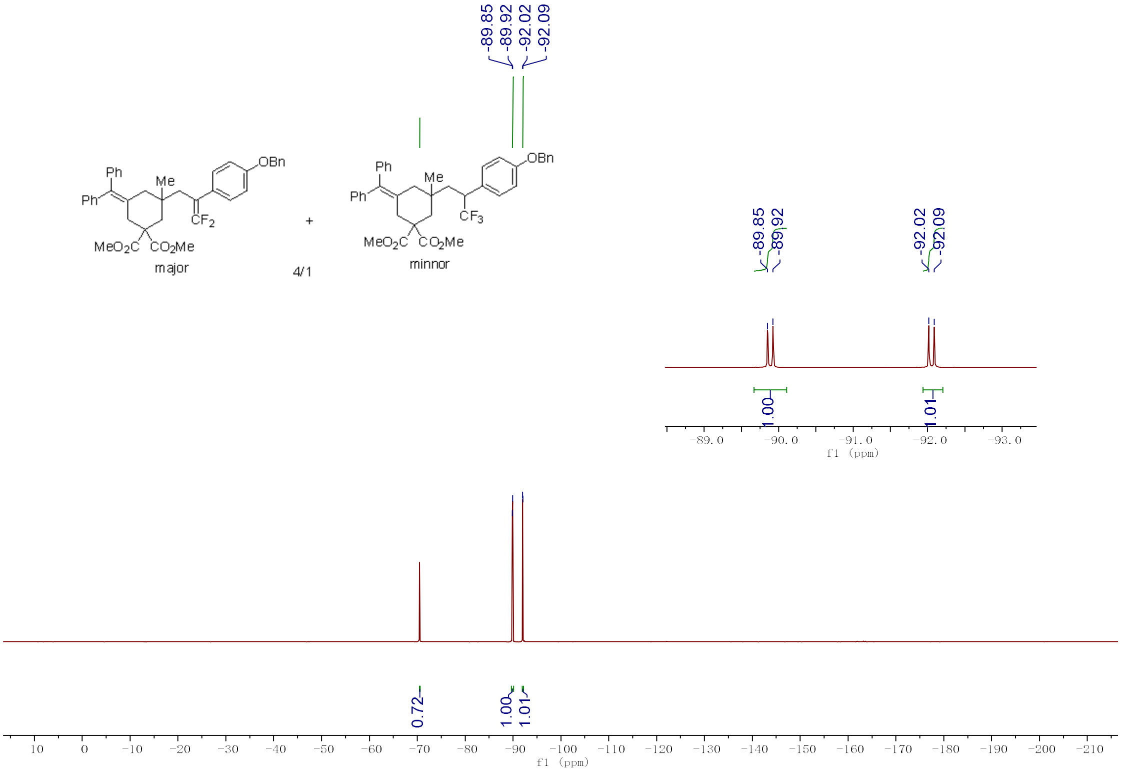

Supplementary figure 213. ^{19}F NMR of compound **69**

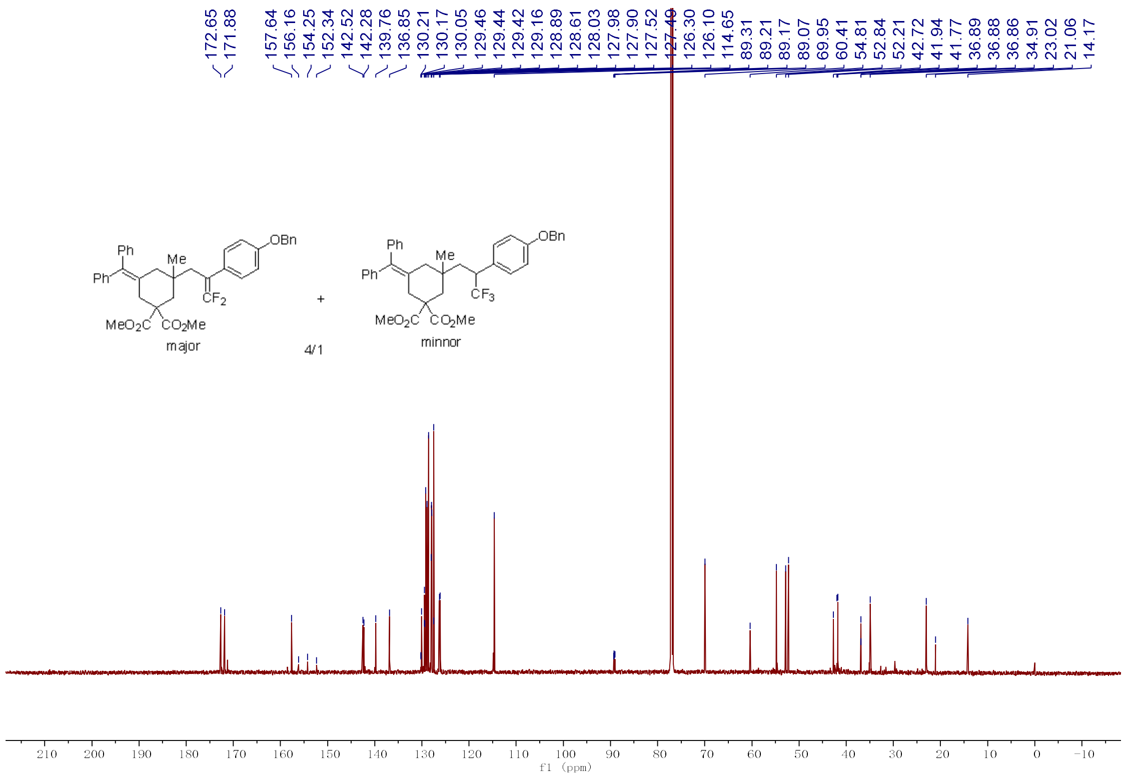

Supplementary figure 214. ^{13}C NMR of compound **69**

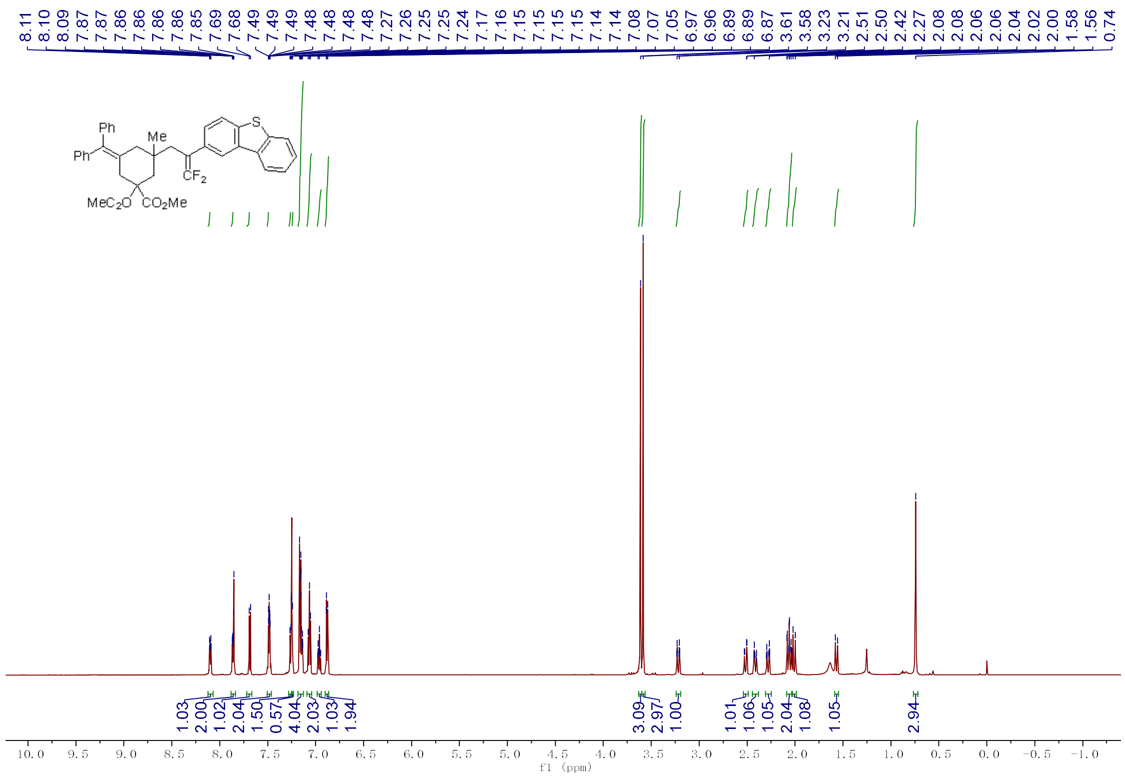

Supplementary figure 215. ^{1}H NMR of compound **70**

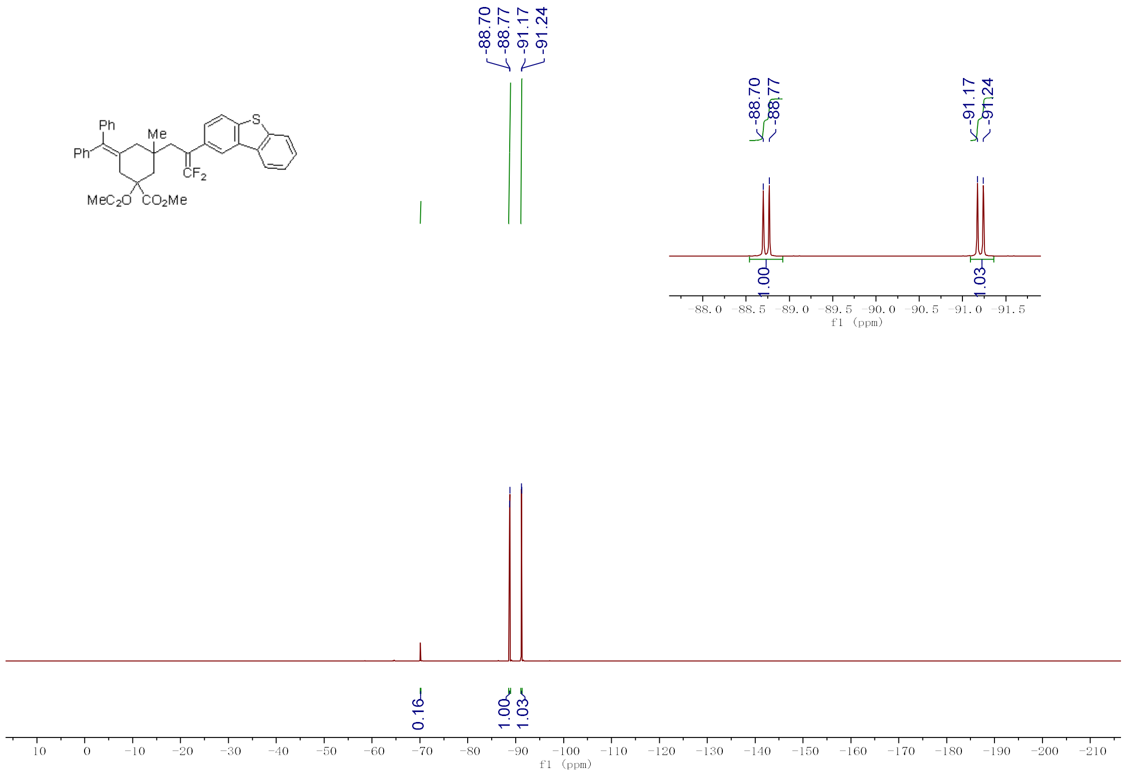

Supplementary figure 216. ^{19}F NMR of compound **70**

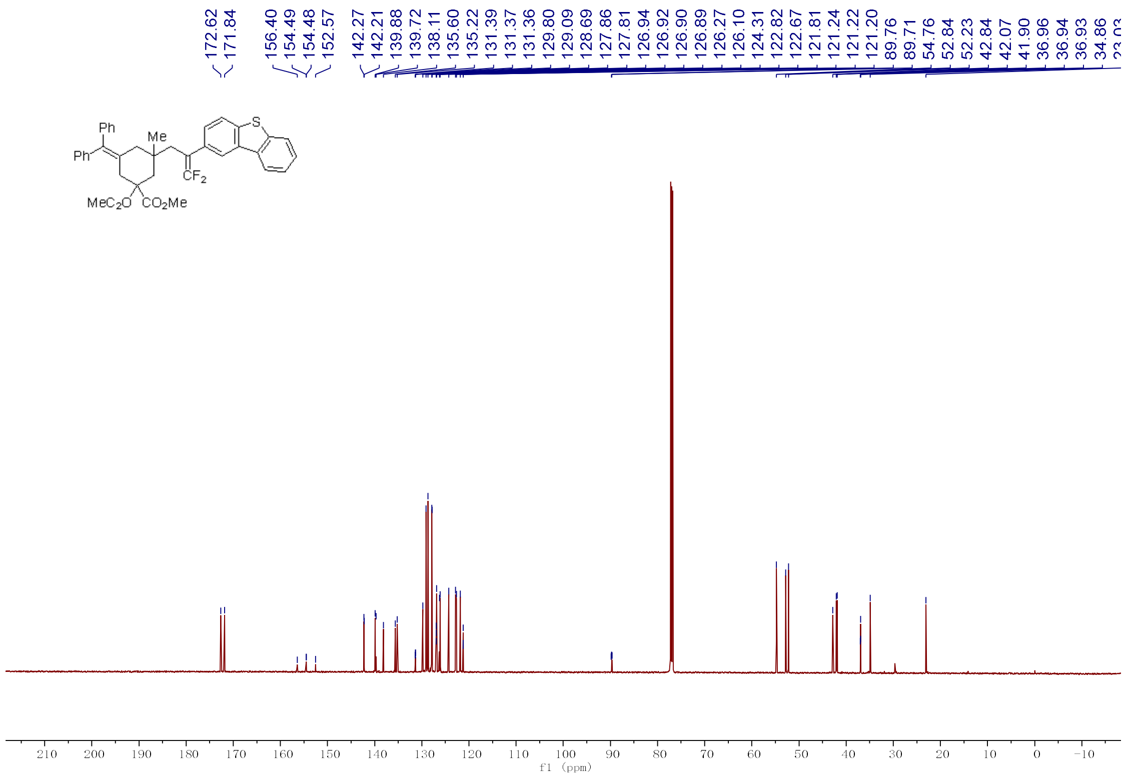

Supplementary figure 217. ^{13}C NMR of compound **70**

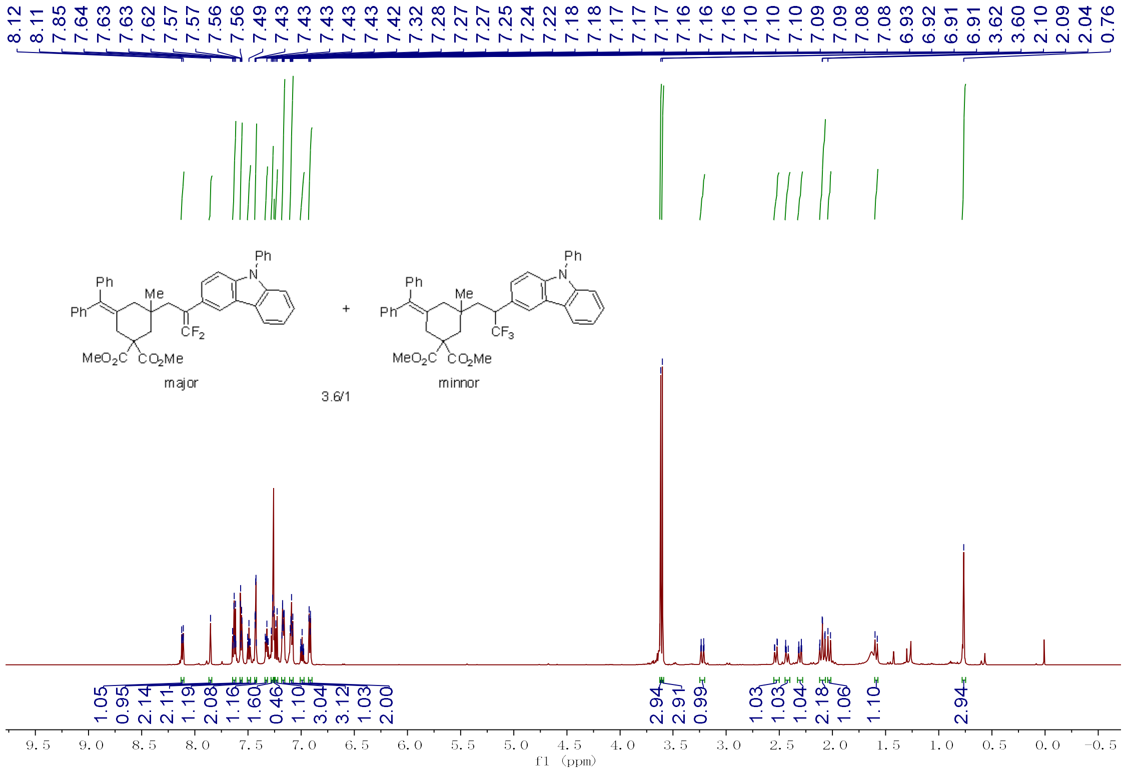

Supplementary figure 218. ^{1}H NMR of compound **71**

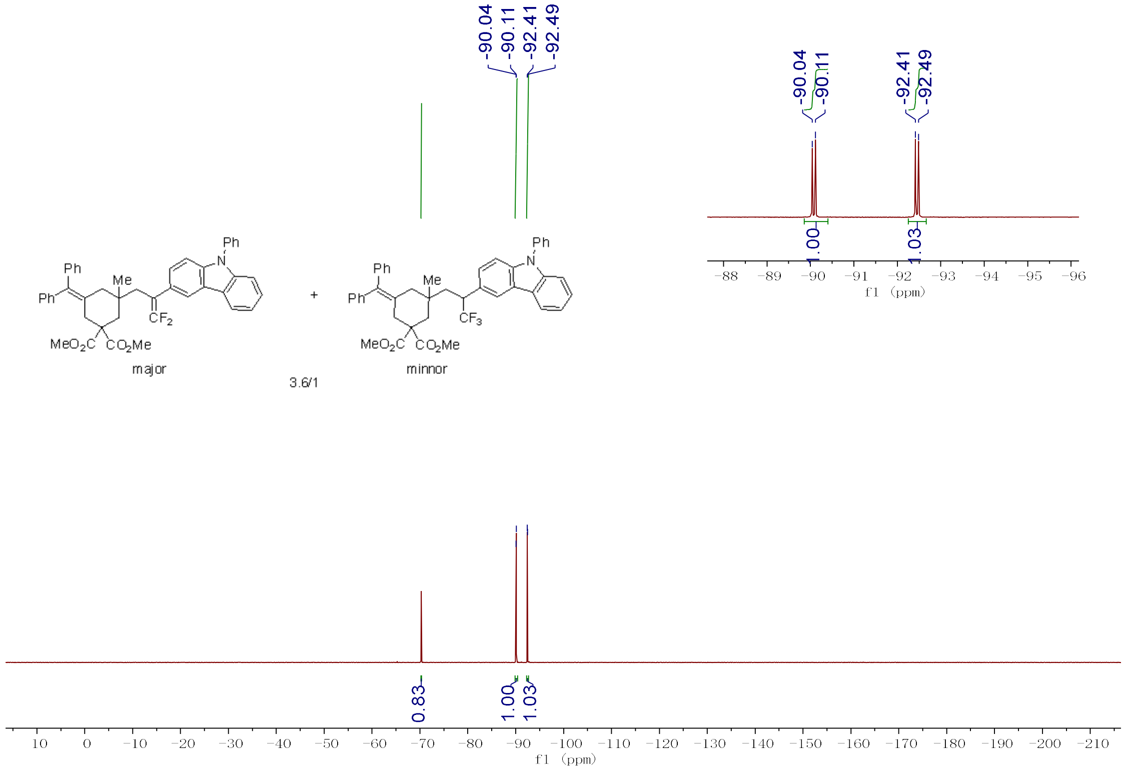

Supplementary figure 219. ^{19}F NMR of compound **71**

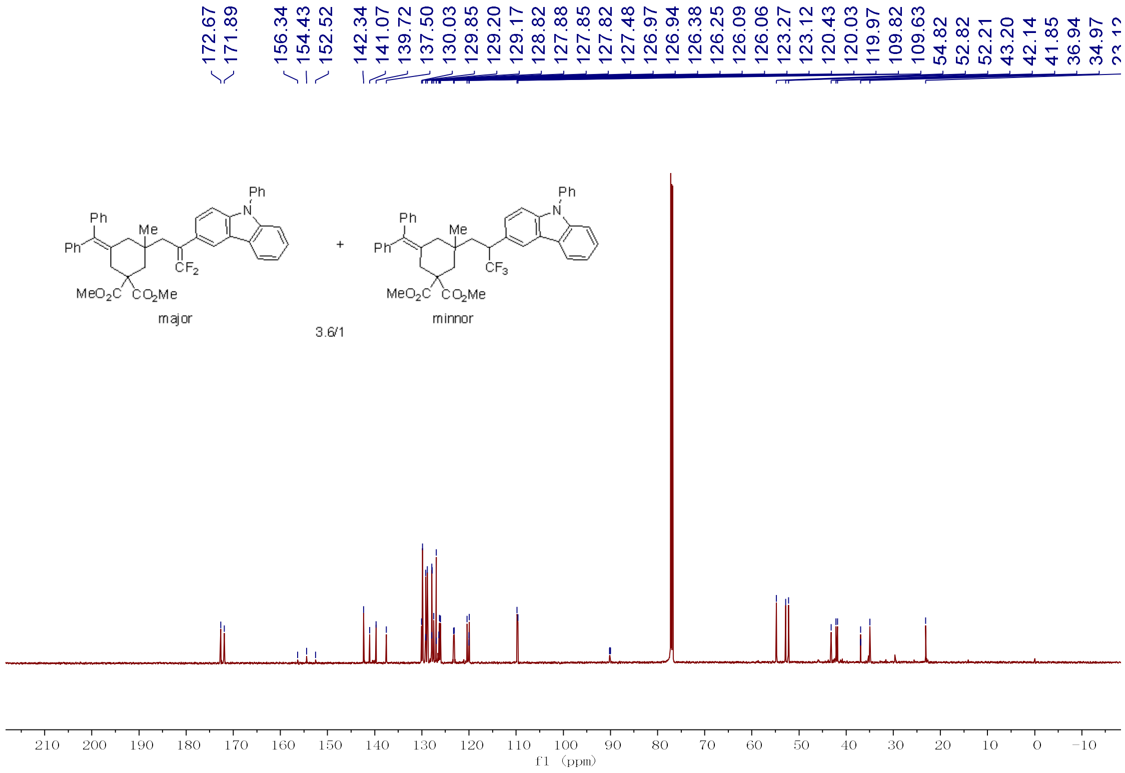

Supplementary figure 220. ^{13}C NMR of compound **71**

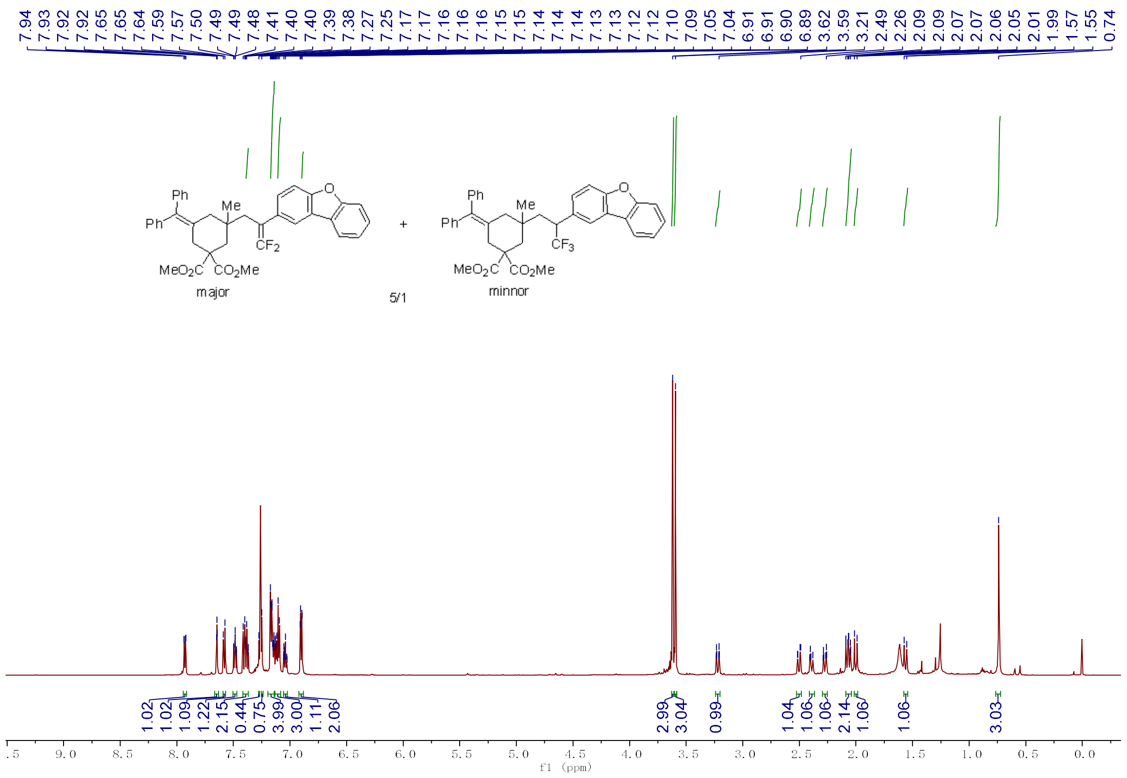

Supplementary figure 221. ^{1}H NMR of compound **72**

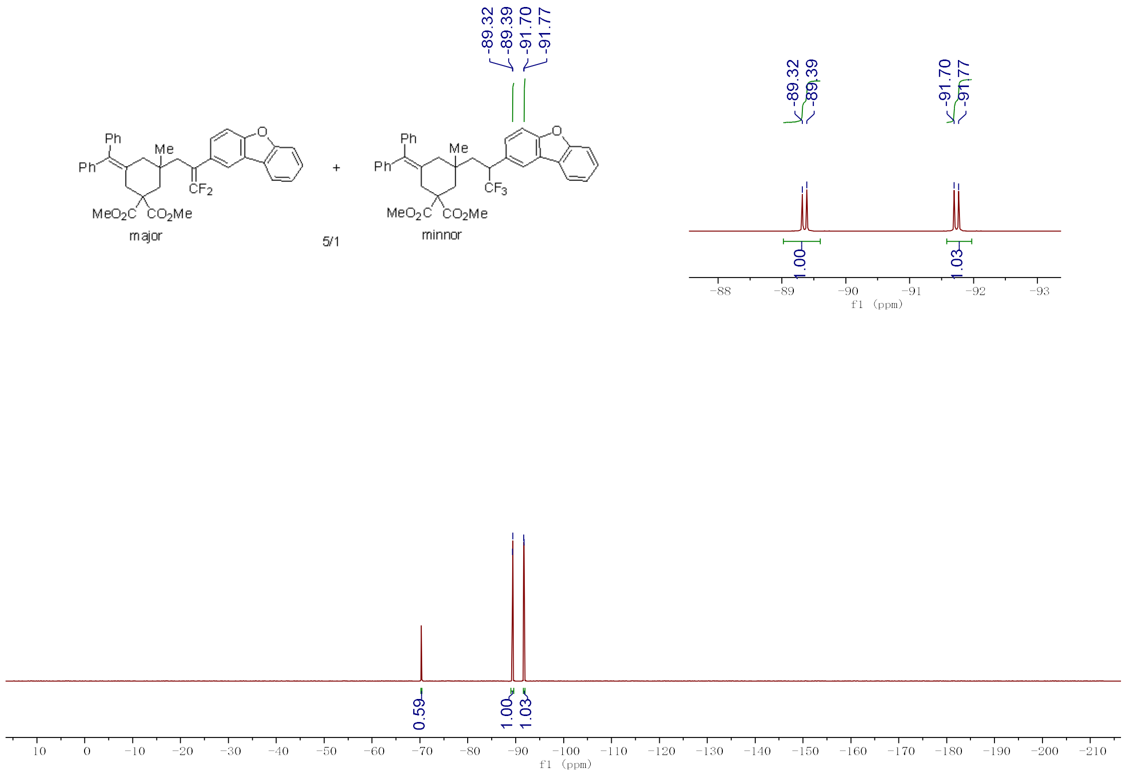

Supplementary figure 222. ^{19}F NMR of compound **72**

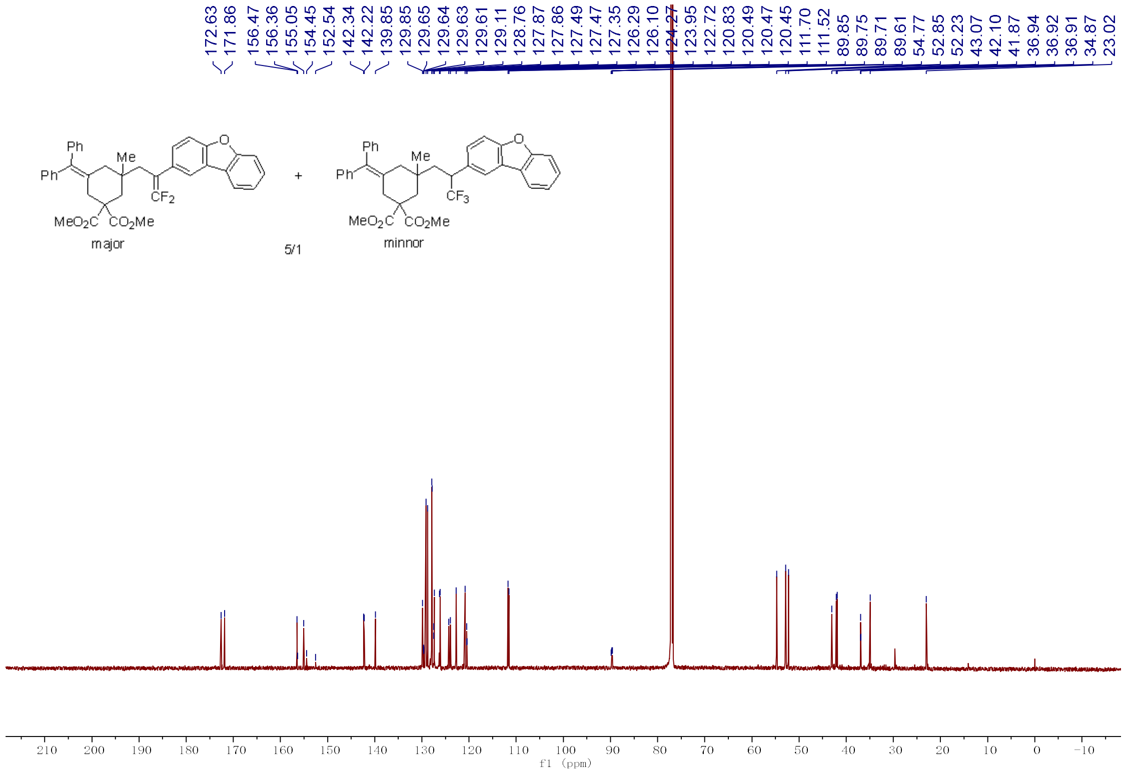

Supplementary figure 223. ^{13}C NMR of compound **72**

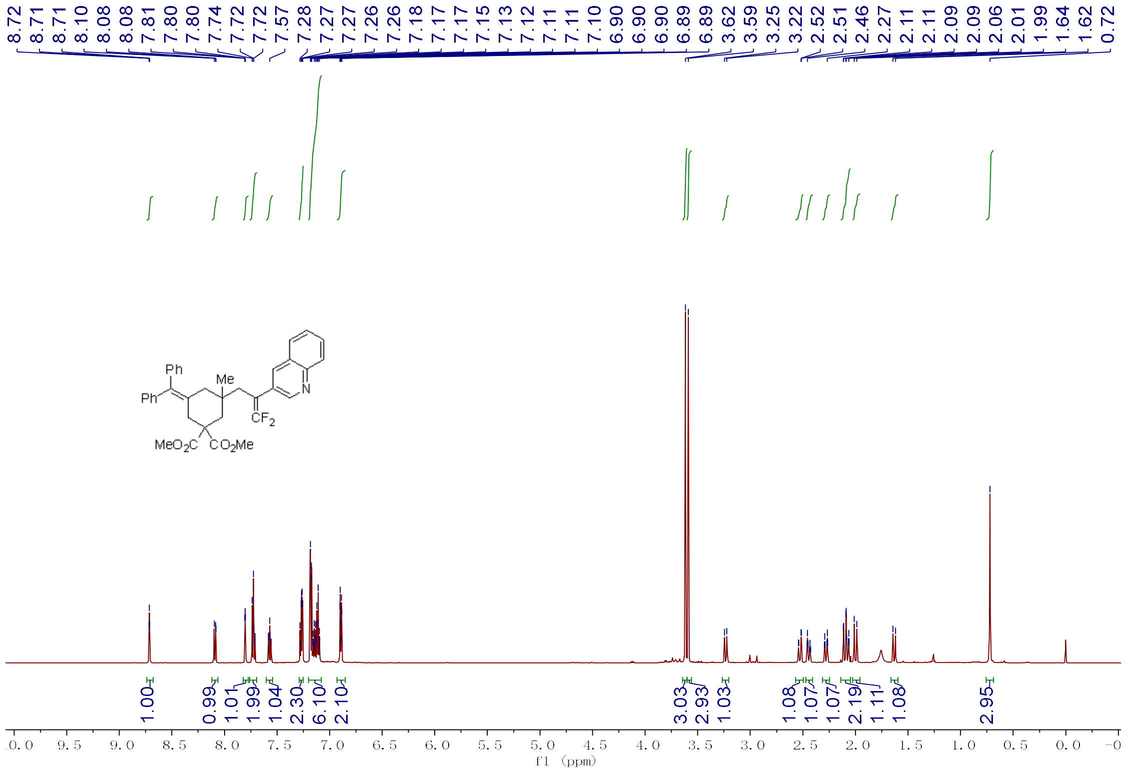

Supplementary figure 224. ^{1}H NMR of compound **73**

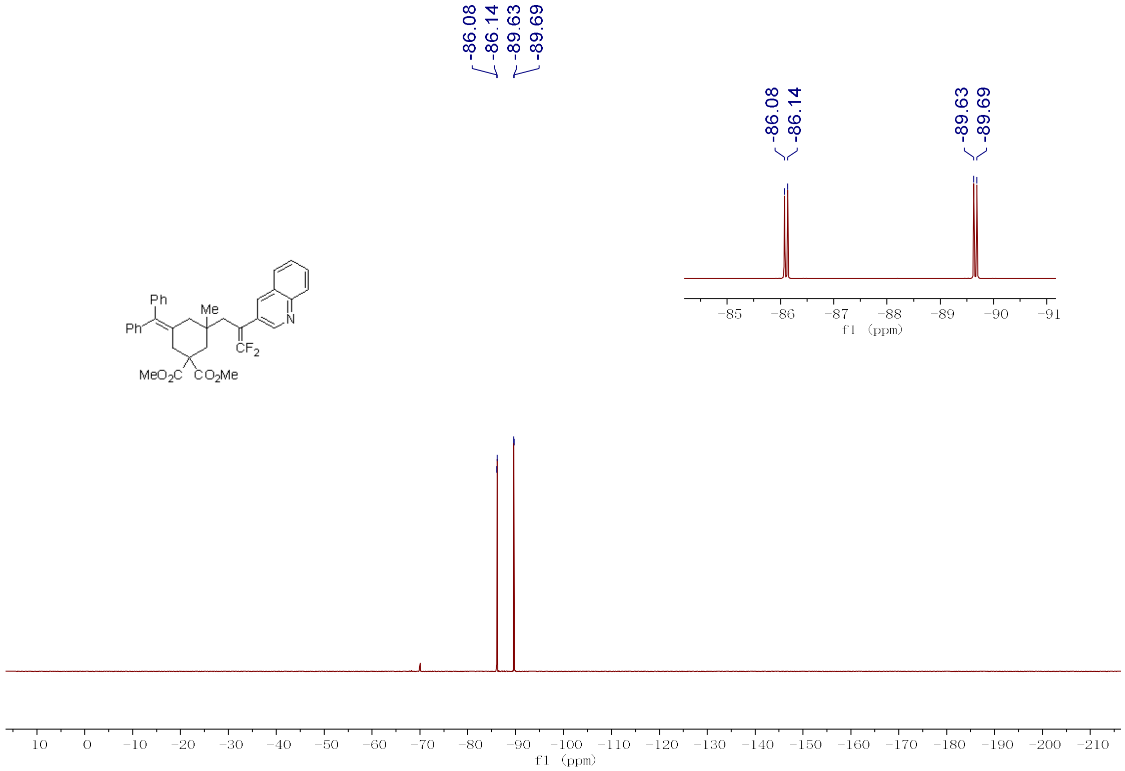

Supplementary figure 225. ^{19}F NMR of compound **73**

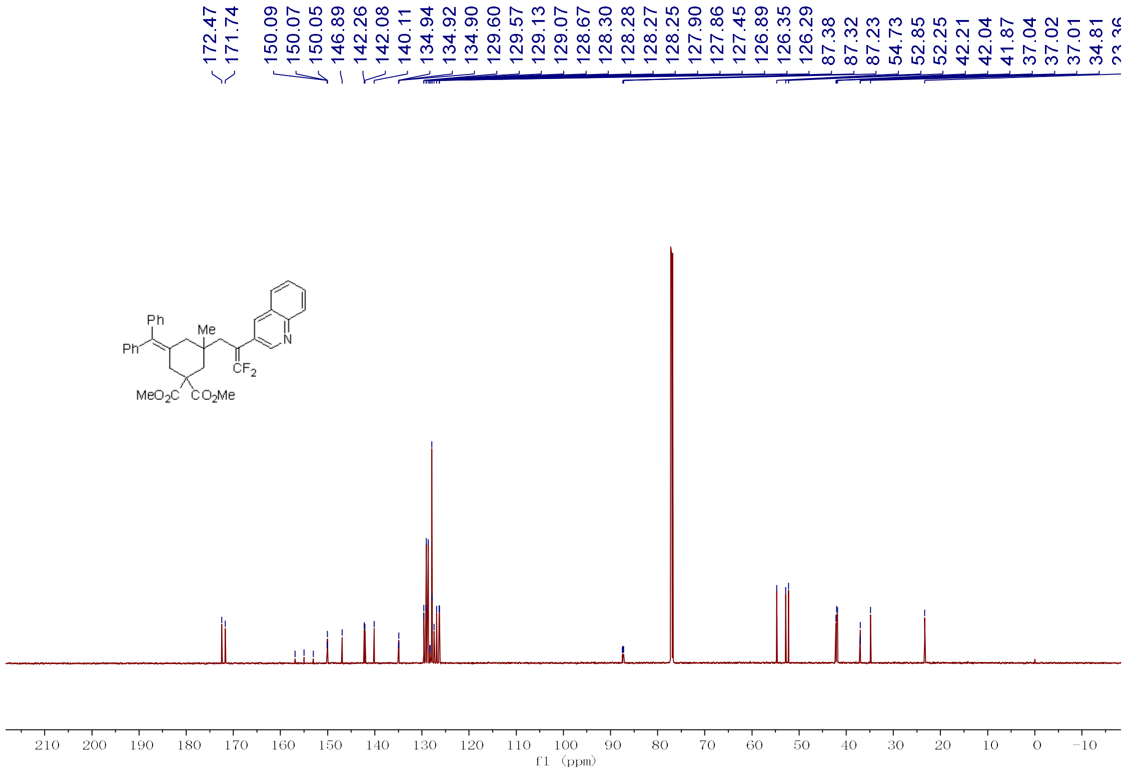

Supplementary figure 226. ^{13}C NMR of compound **73**

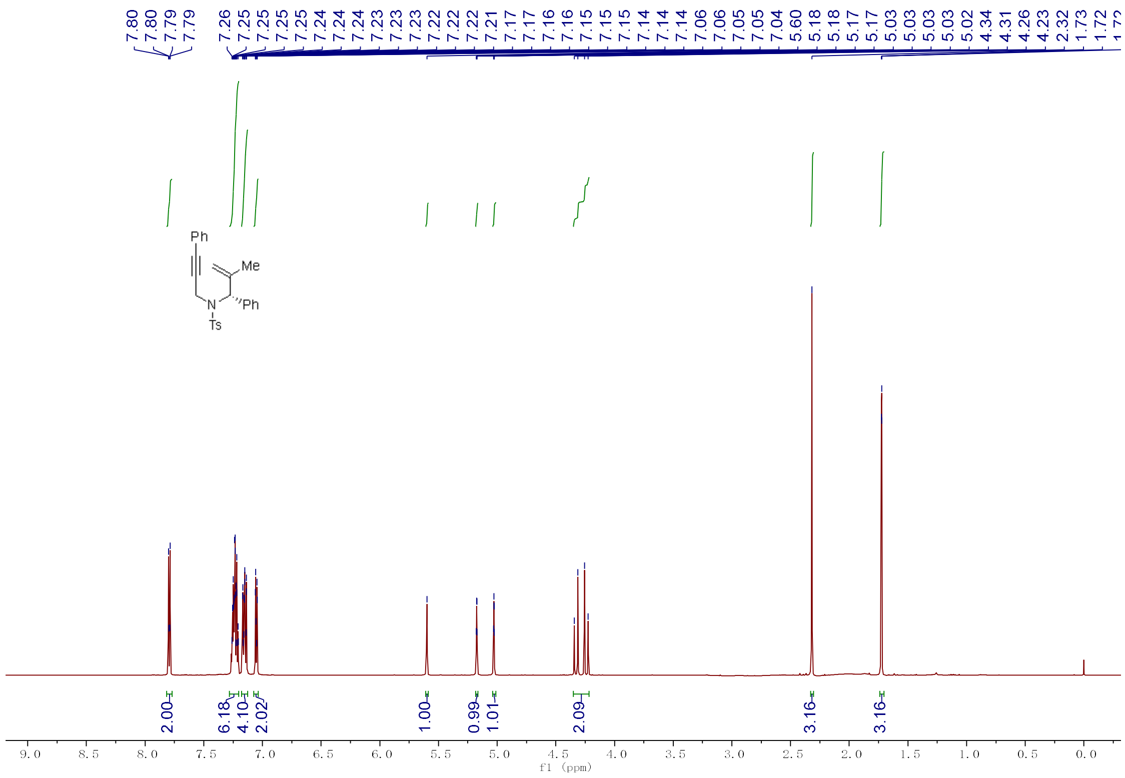

Supplementary figure 227. ^{1}H NMR of compound **74**

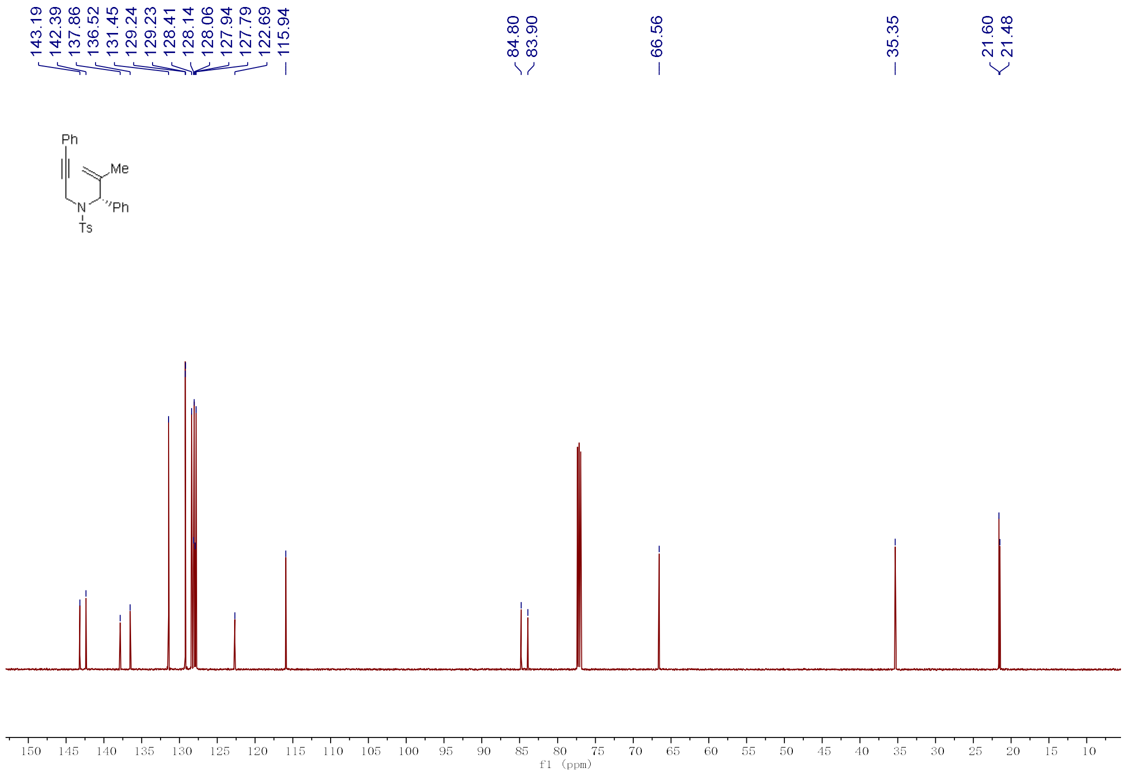

Supplementary figure 228. ^{13}C NMR of compound **74**

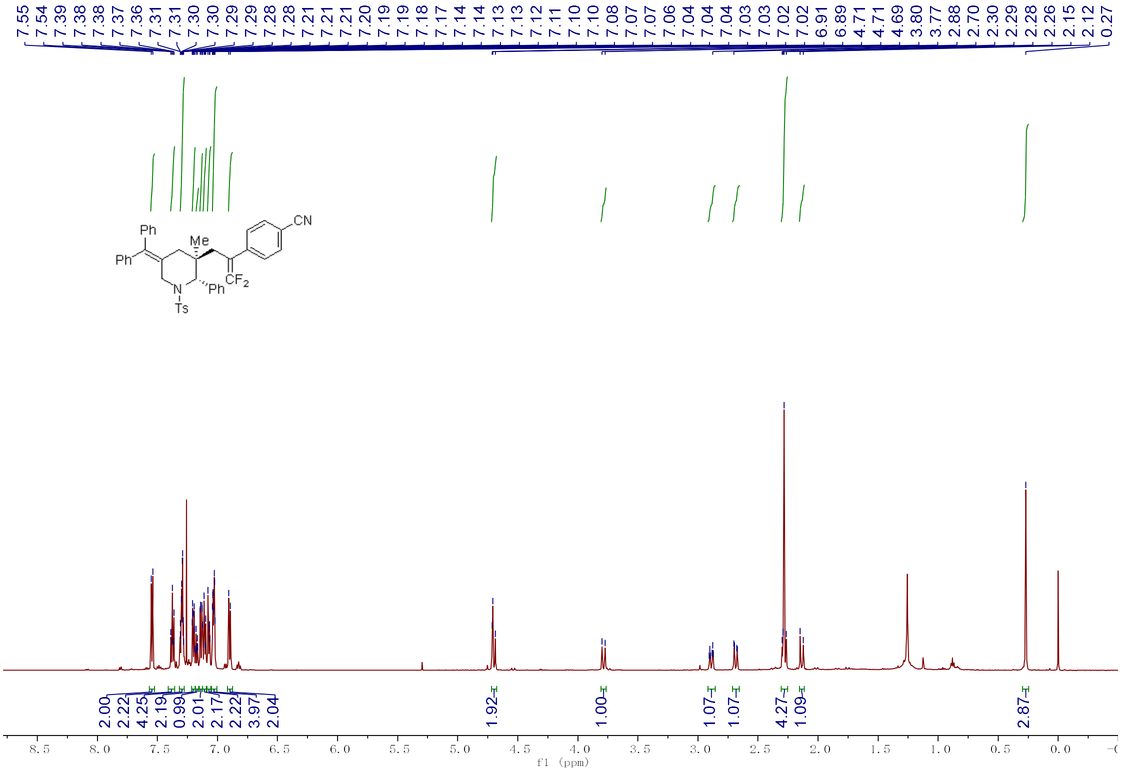

Supplementary figure 229. ^{1}H NMR of compound **75**

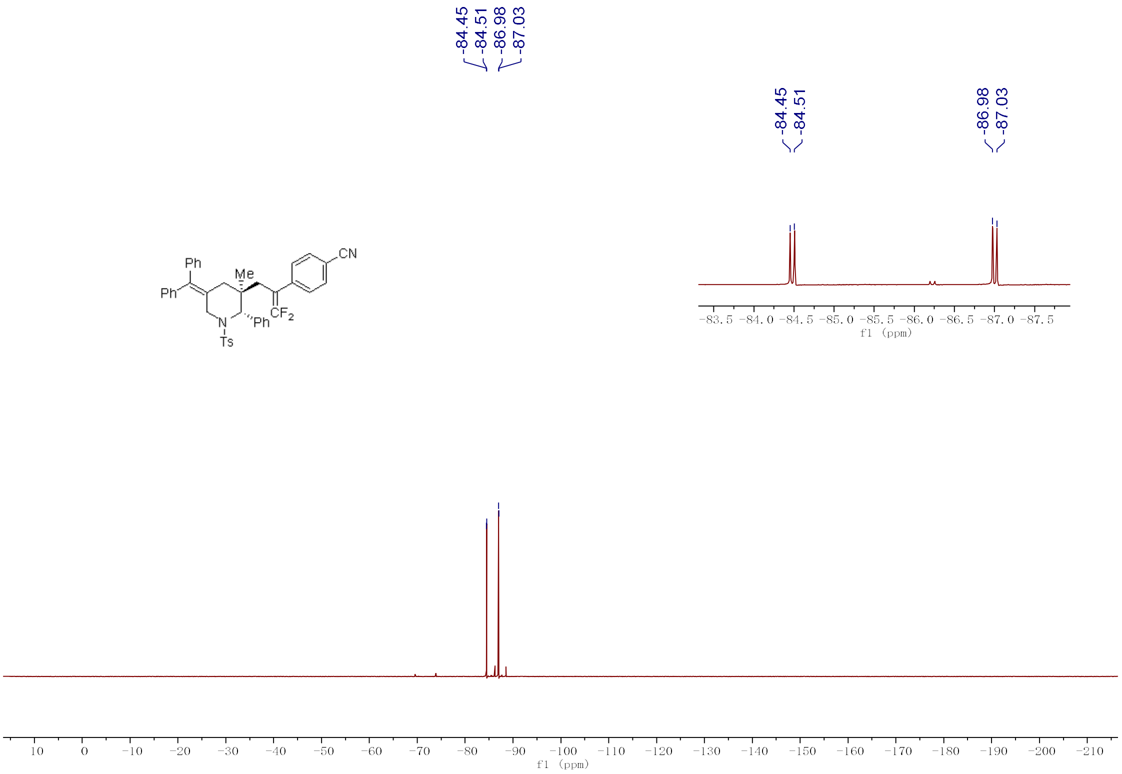

Supplementary figure 230. ^{19}F NMR of compound **75**

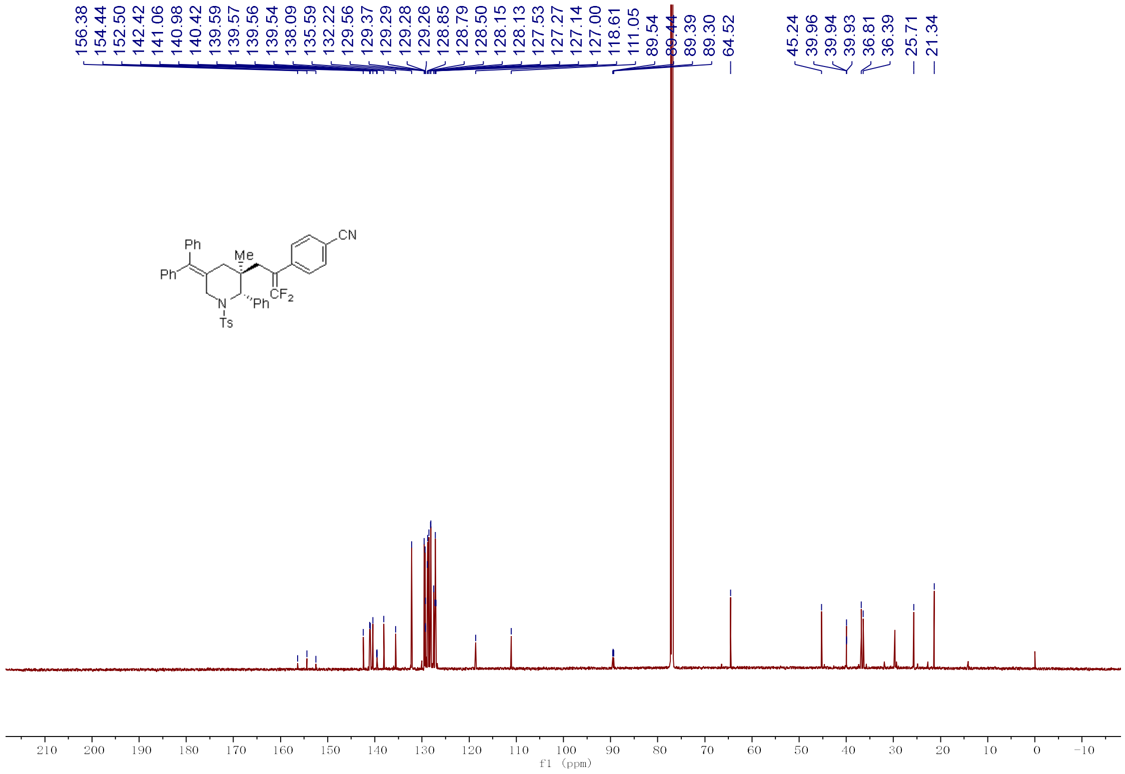

Supplementary figure 231. ^{13}C NMR of compound **75**

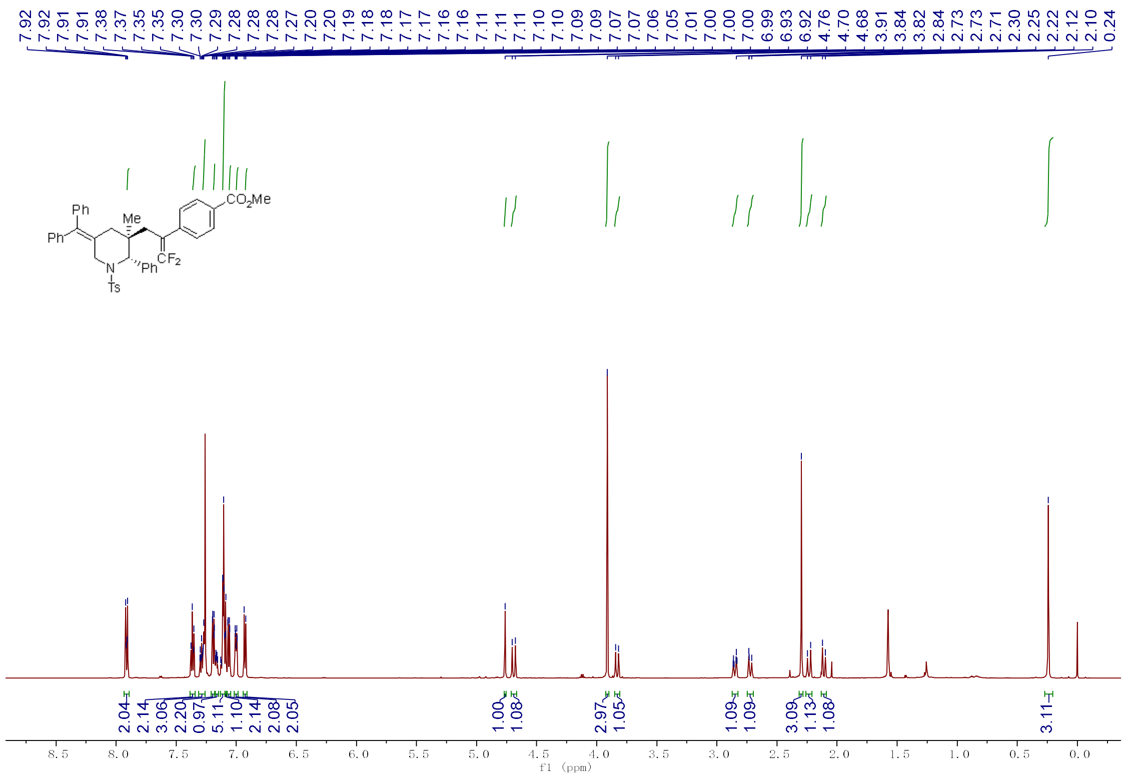

Supplementary figure 232. ^{1}H NMR of compound **76**

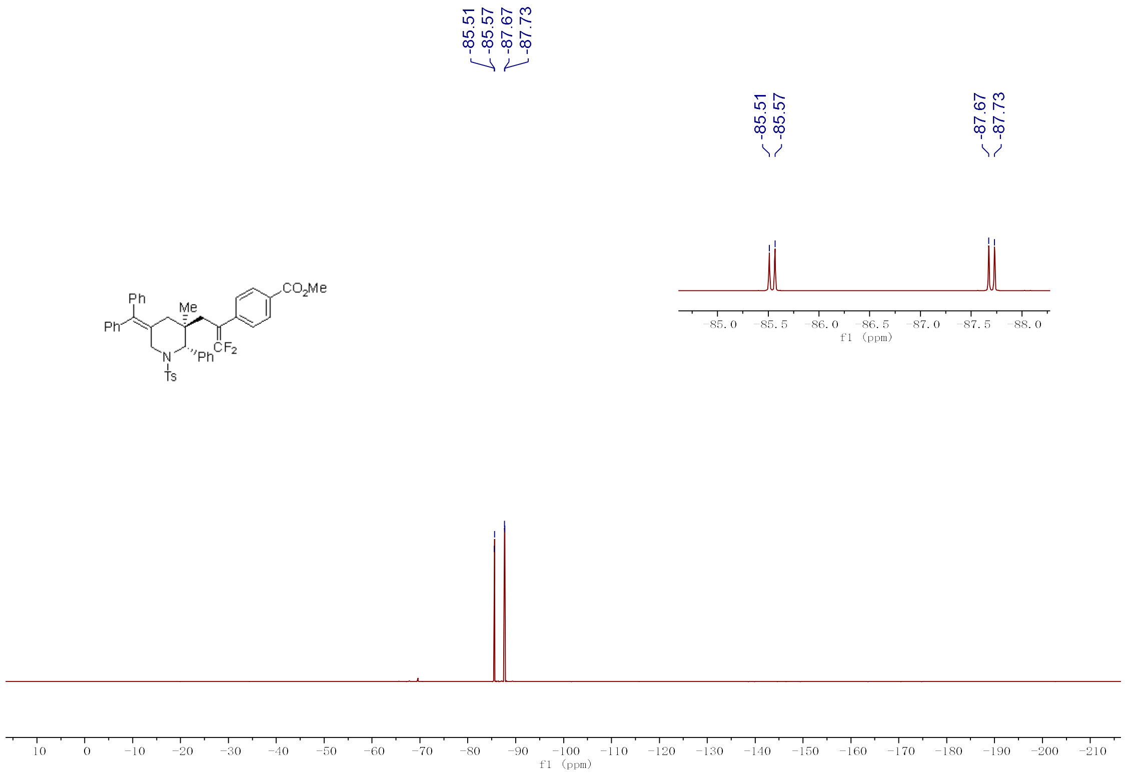

Supplementary figure 233. ^{19}F NMR of compound **76**

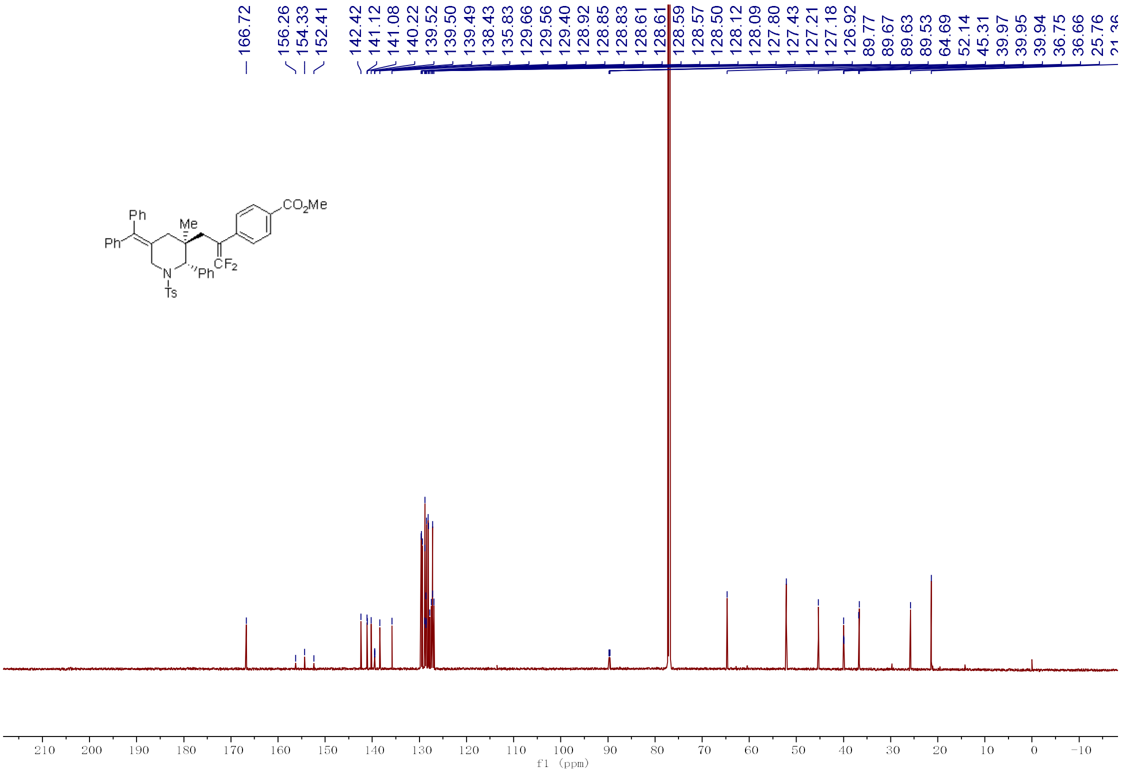

Supplementary figure 234. ^{13}C NMR of compound **76**

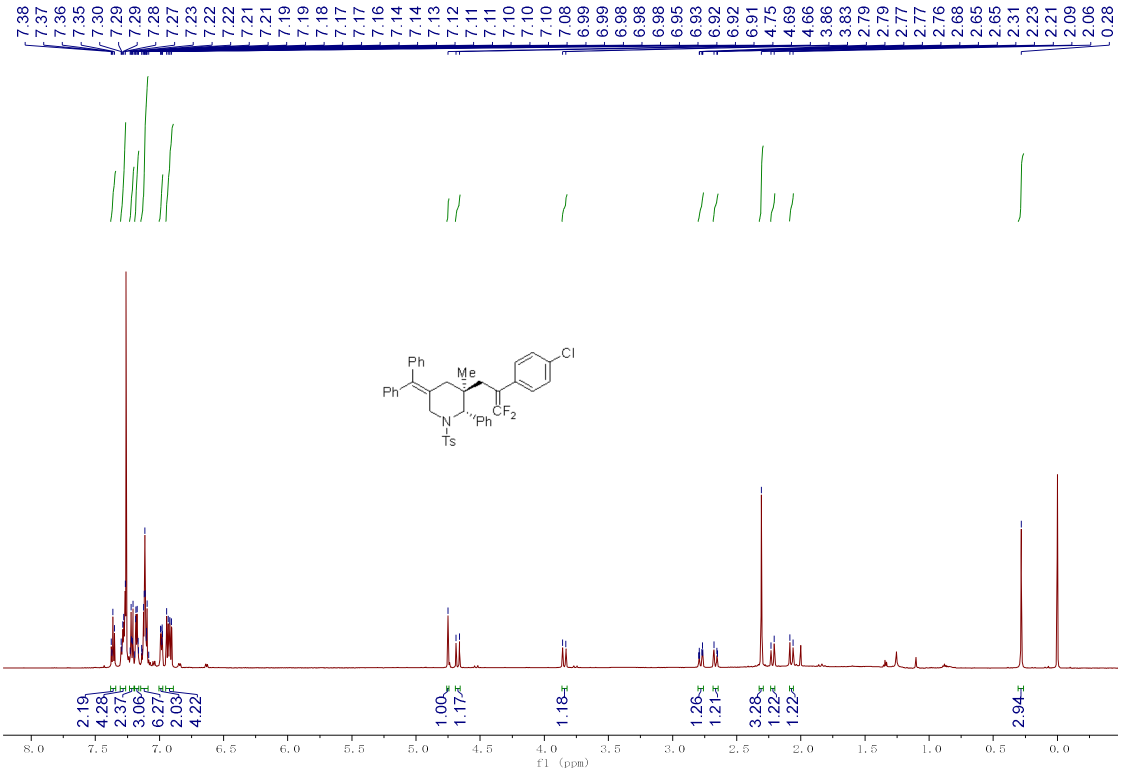

Supplementary figure 235. ^{1}H NMR of compound **77**

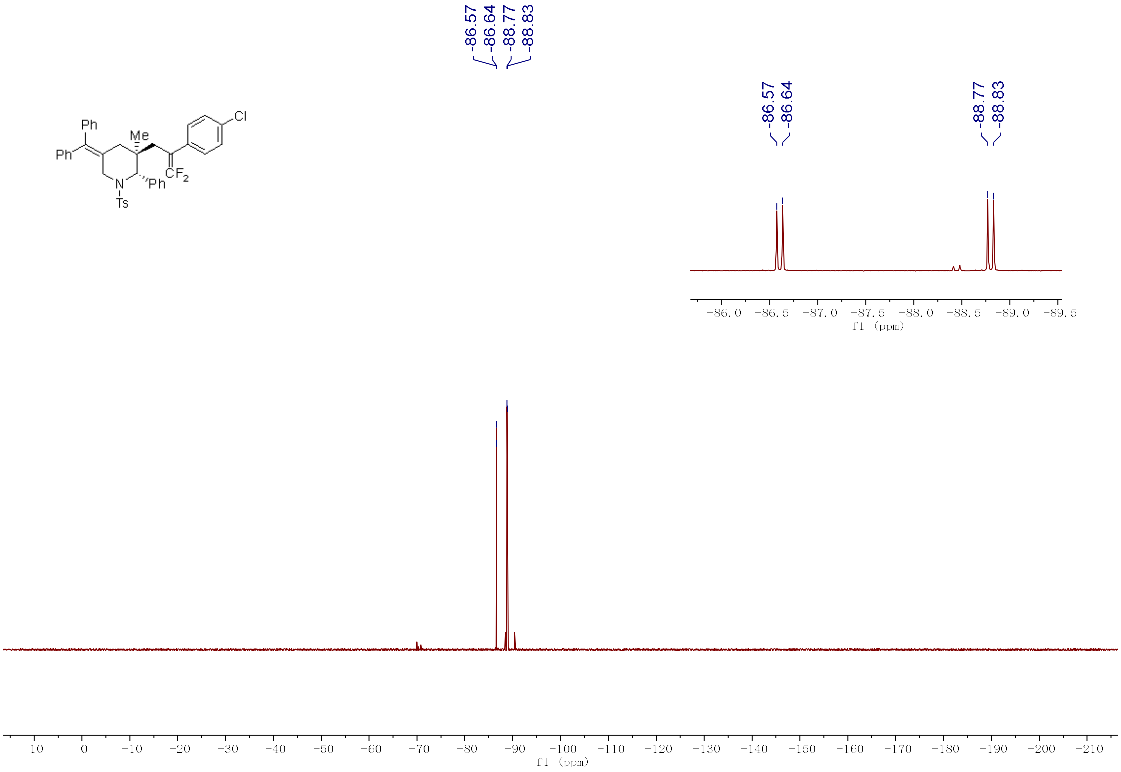

Supplementary figure 236. ^{19}F NMR of compound **77**

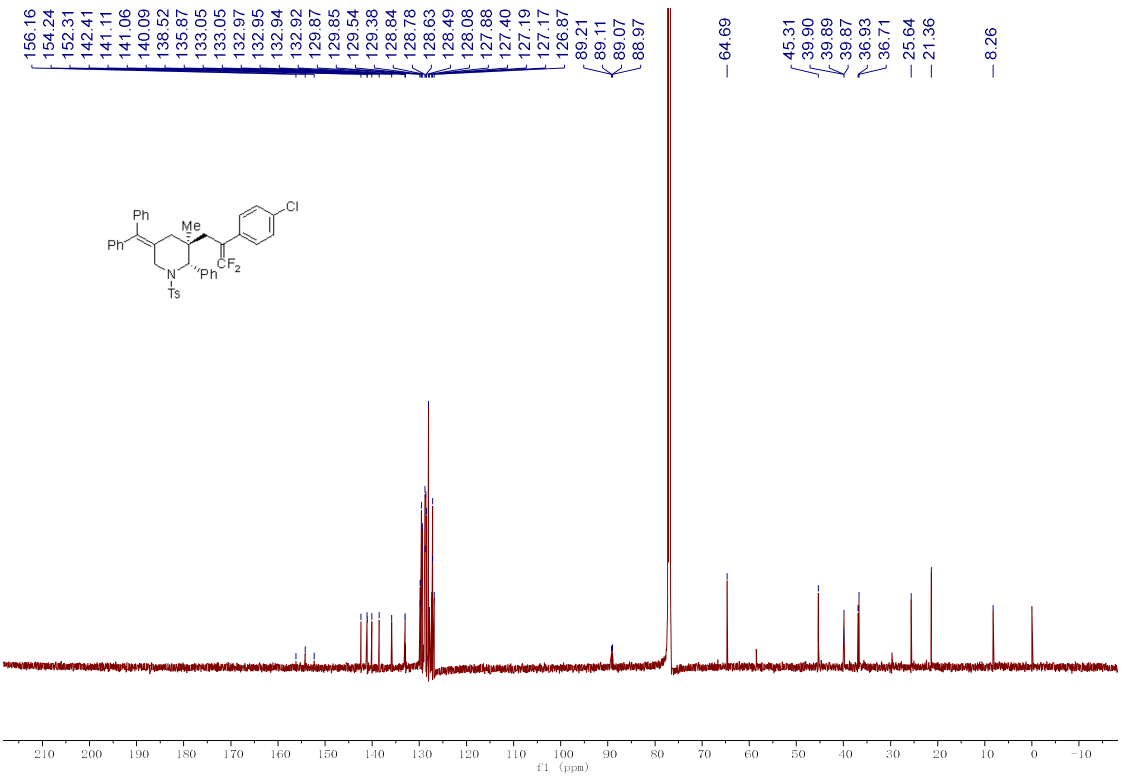

Supplementary figure 237. ^{13}C NMR of compound **77**

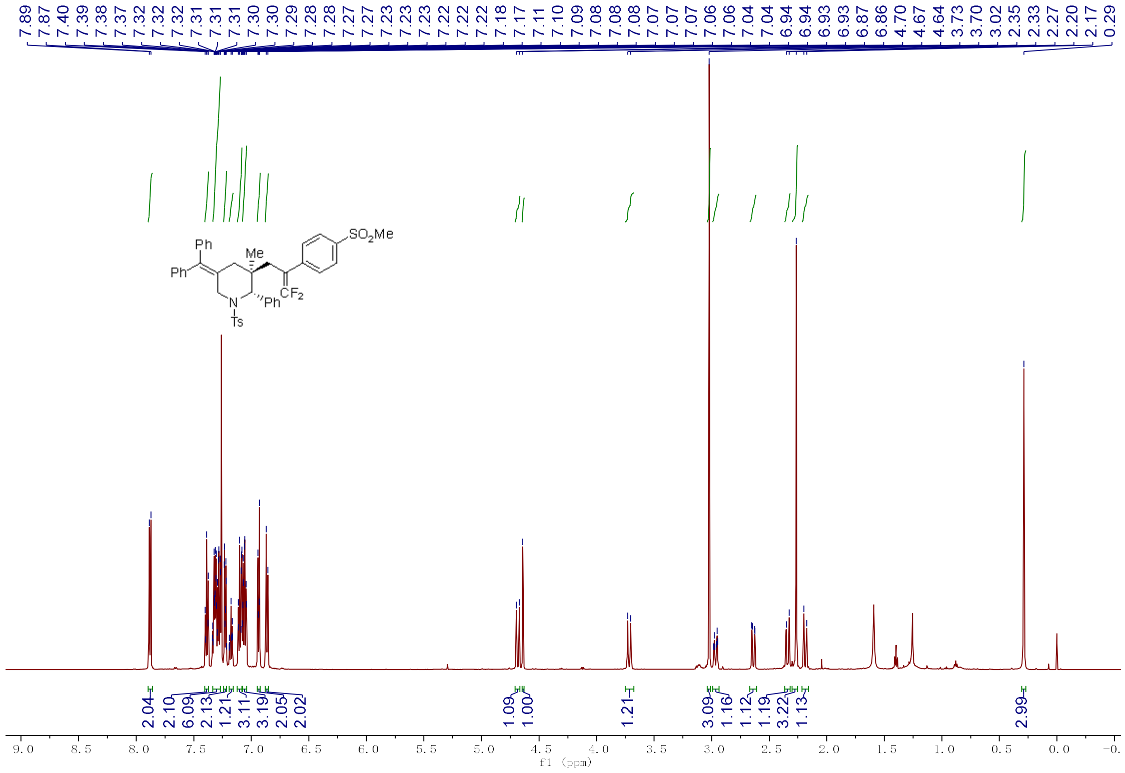

Supplementary figure 238. ^{1}H NMR of compound **78**

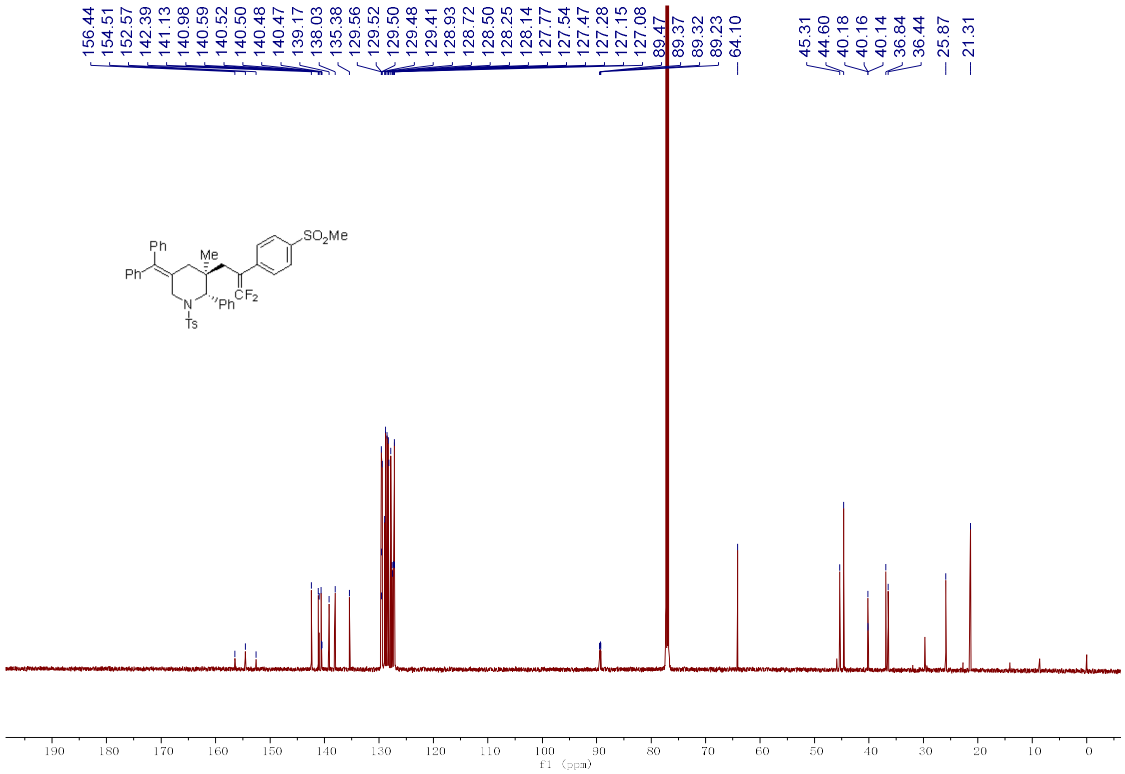

Supplementary figure 239. ^{13}C NMR of compound **78**

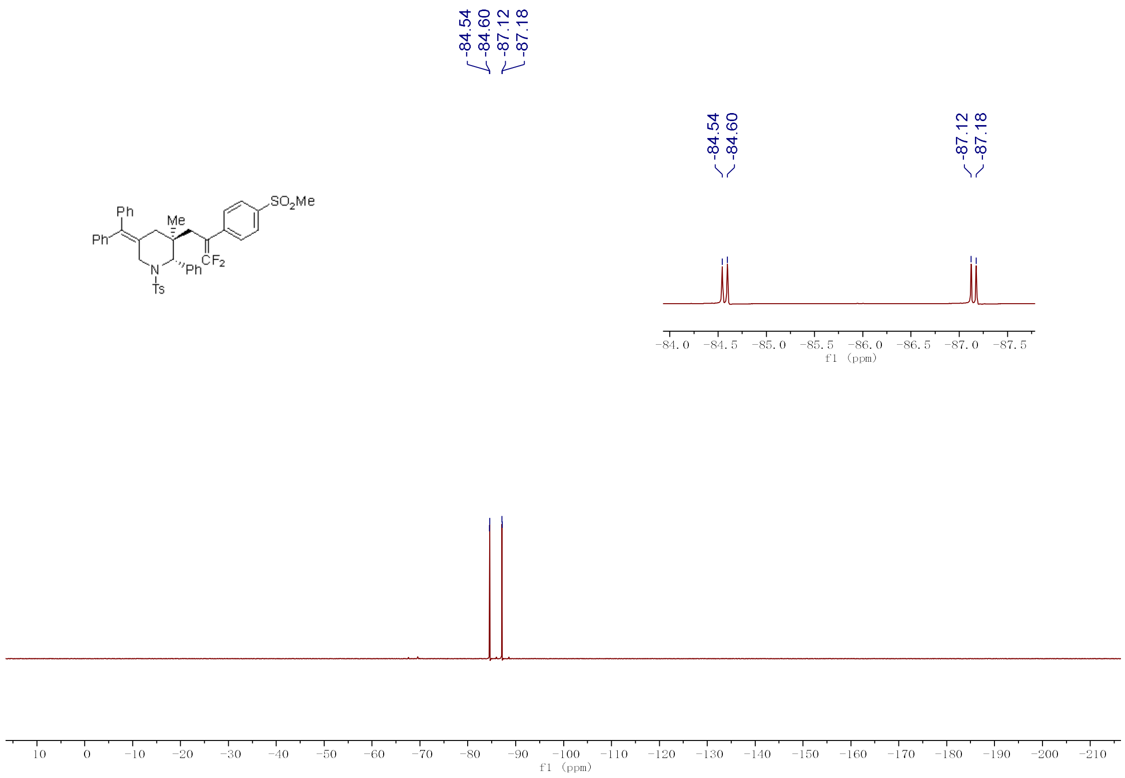

Supplementary figure 240. ^{19}F NMR of compound **78**

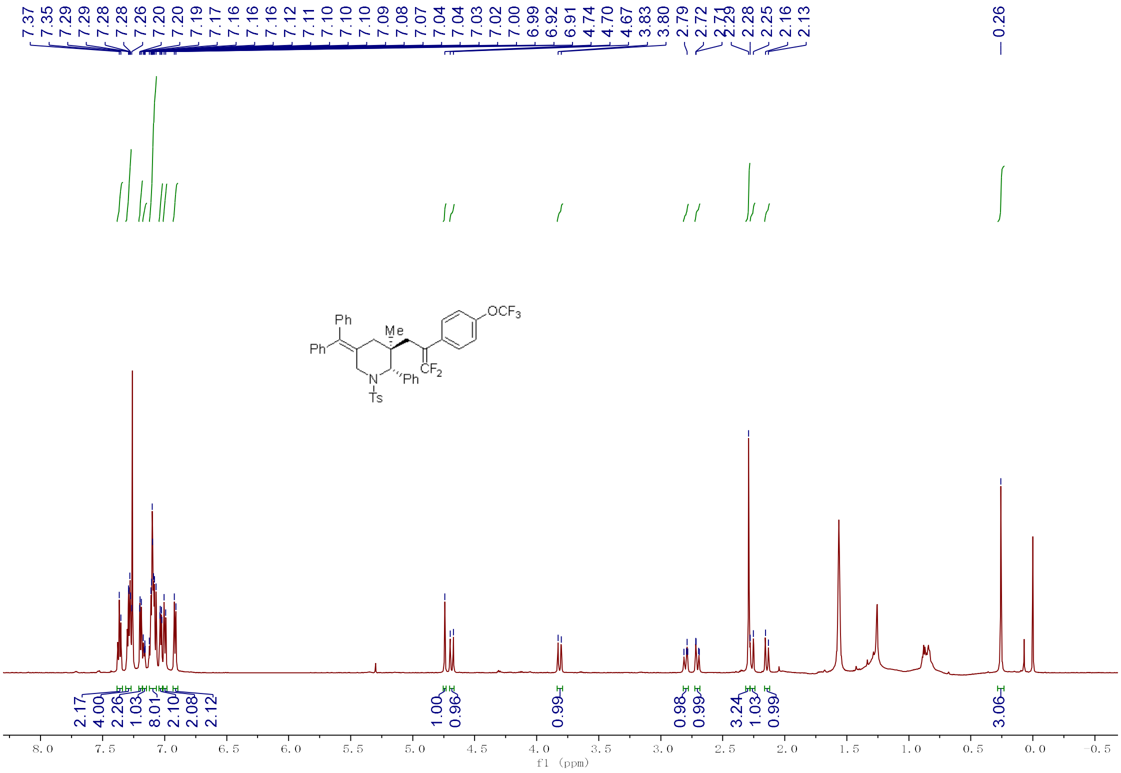

Supplementary figure 241. ^{1}H NMR of compound **79**

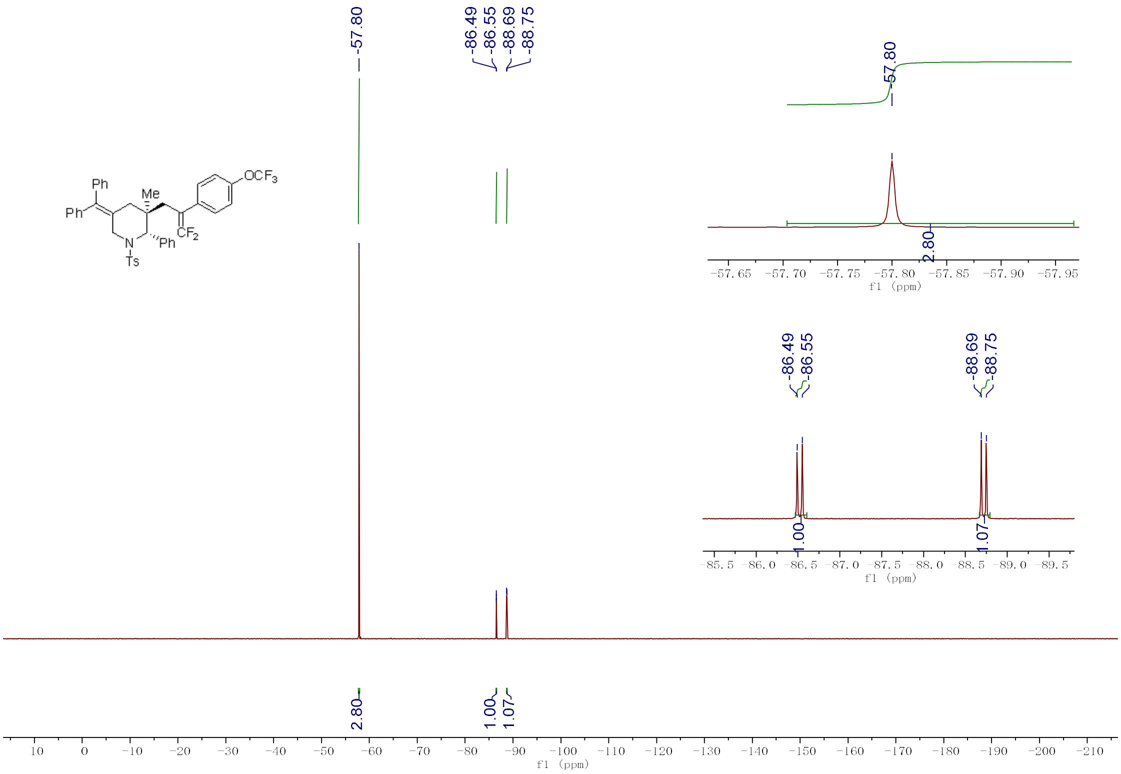

Supplementary figure 242. ^{19}F NMR of compound **79**

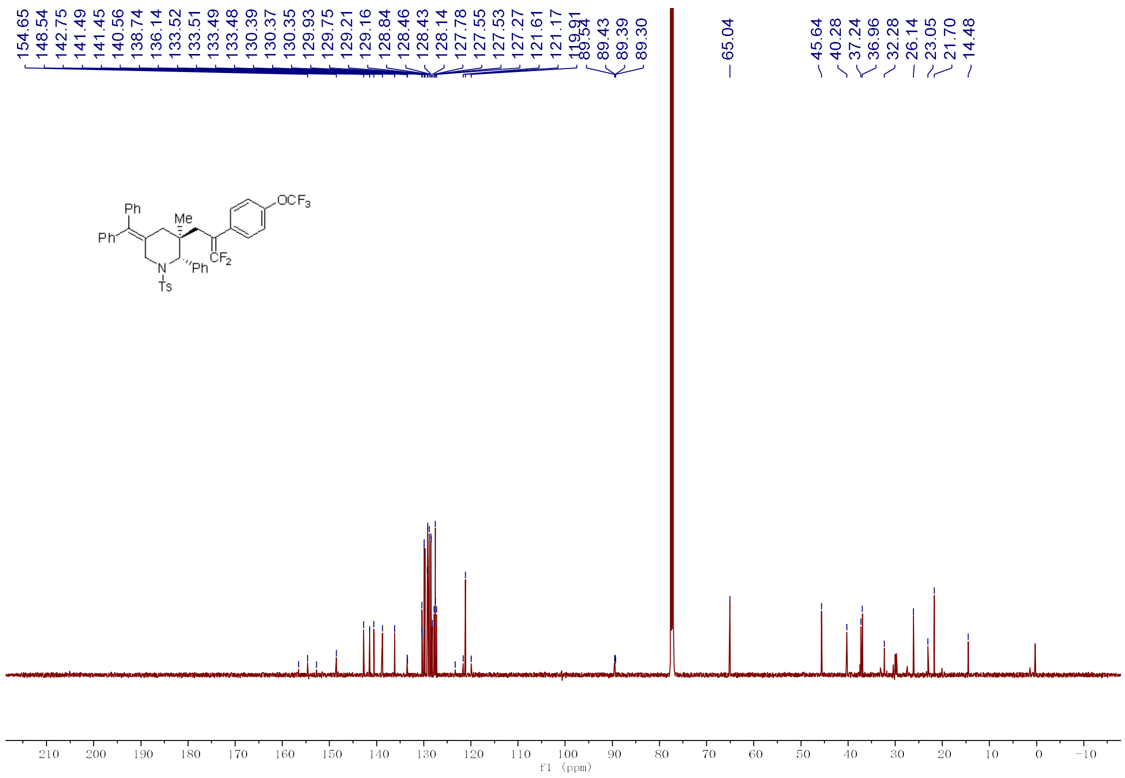

Supplementary figure 243. ^{13}C NMR of compound **79**

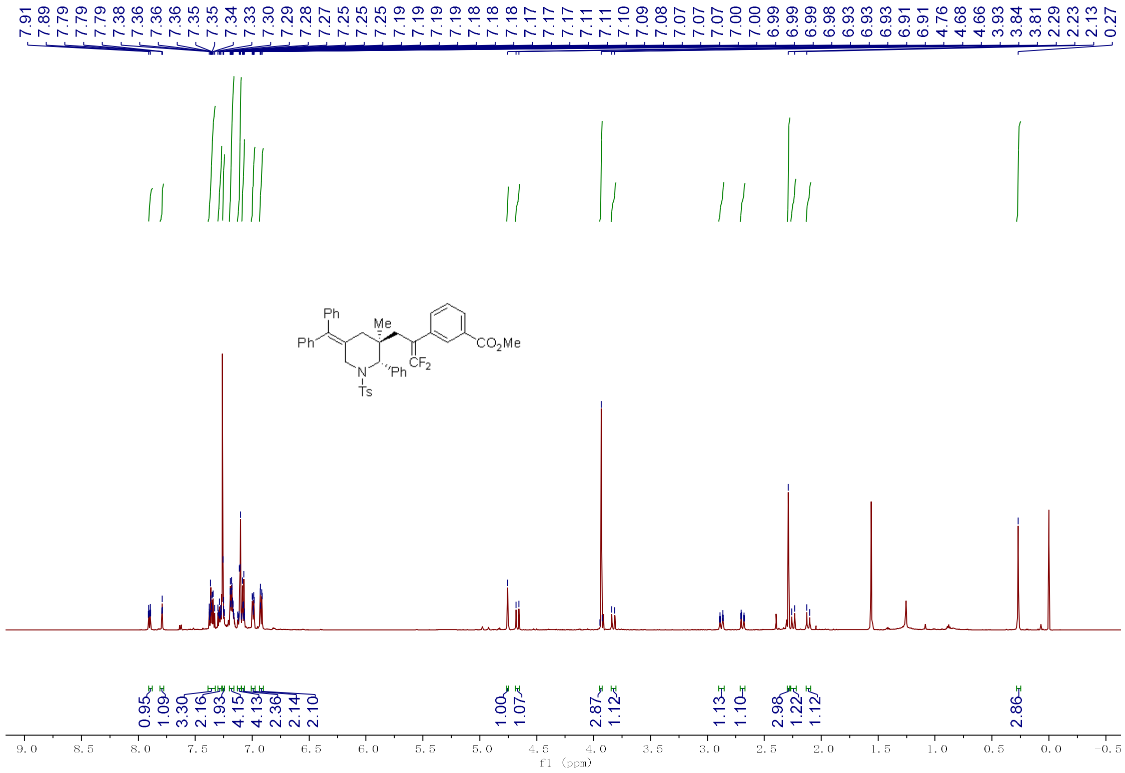

Supplementary figure 244. ^{1}H NMR of compound **80**

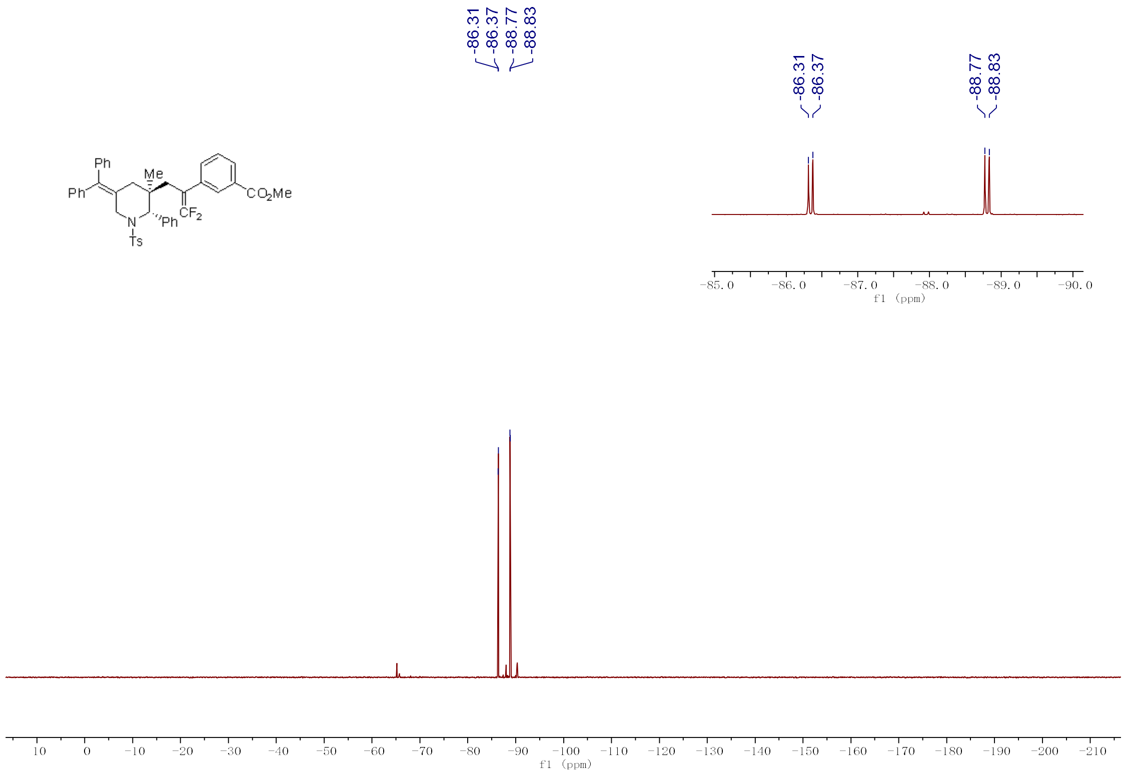

Supplementary figure 245. ^{19}F NMR of compound **80**

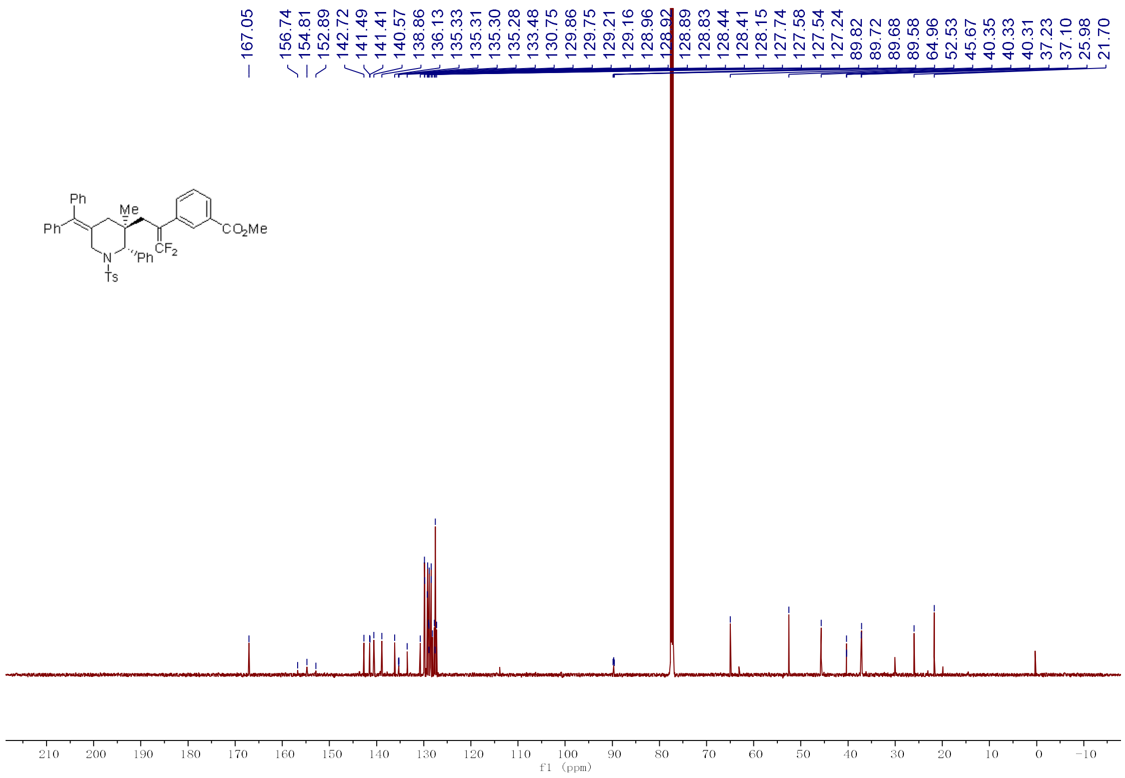

Supplementary figure 246. ^{13}C NMR of compound **80**

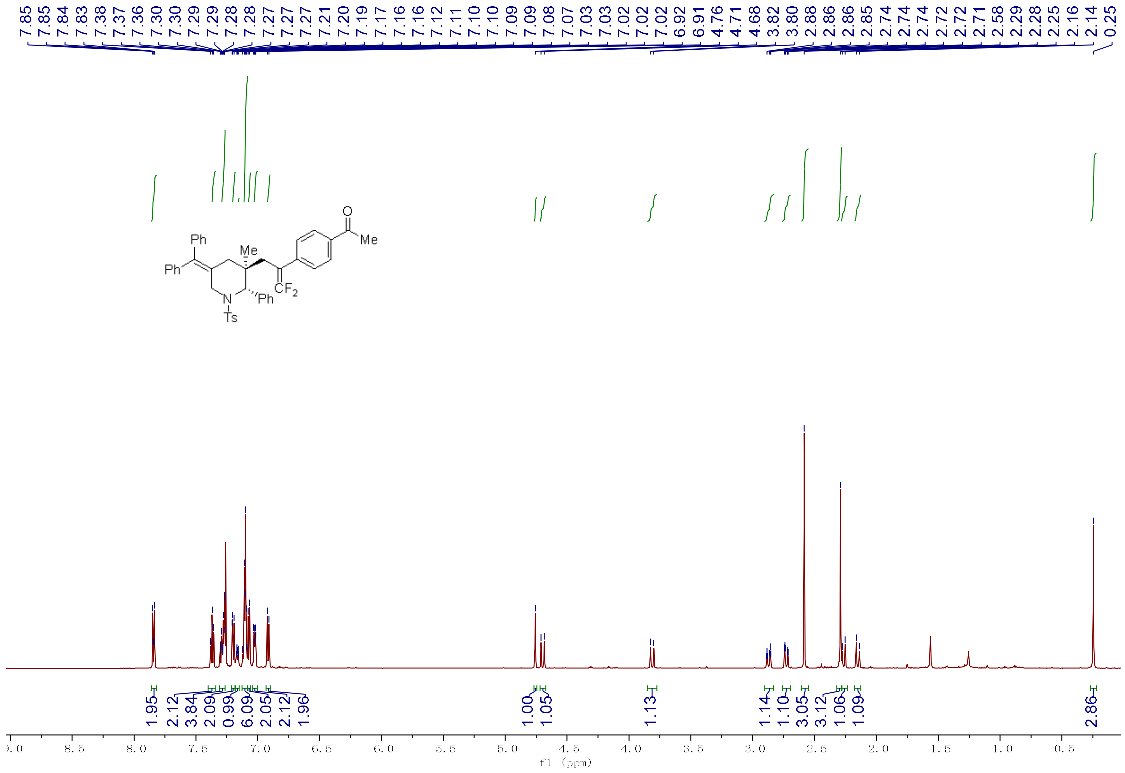

Supplementary figure 247. ^{1}H NMR of compound **81**

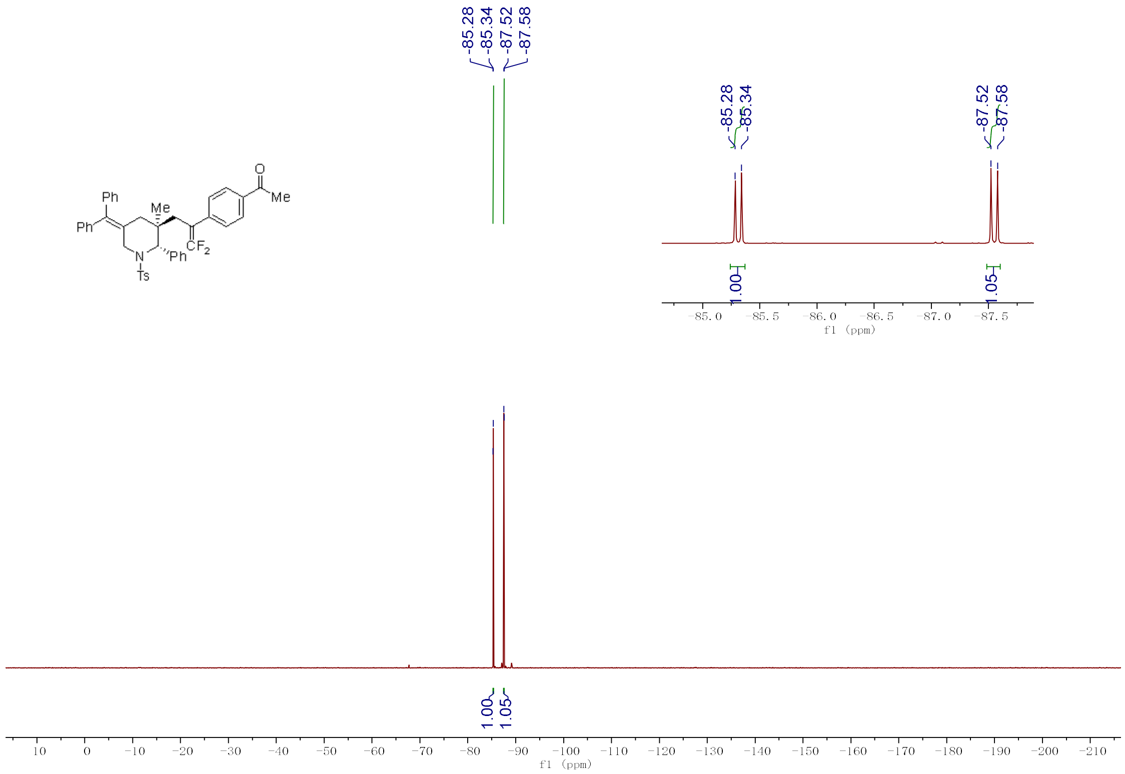

Supplementary figure 248. ^{19}F NMR of compound **81**

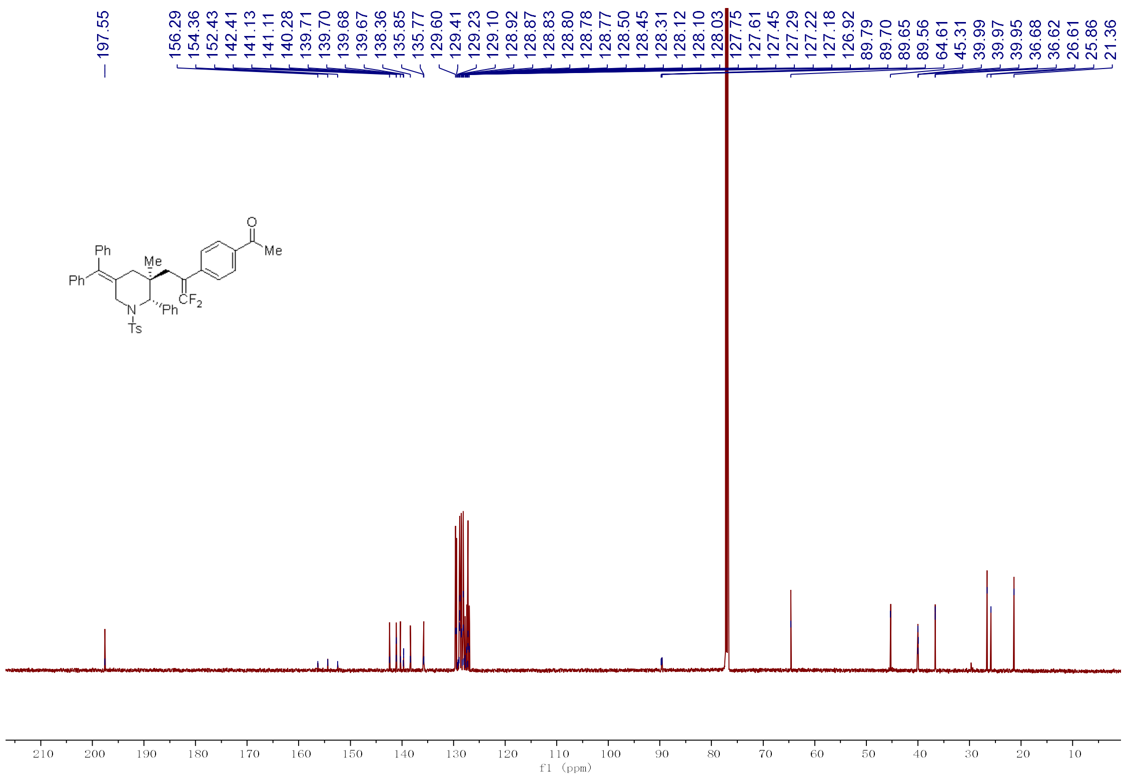

Supplementary figure 249. ^{13}C NMR of compound **81**

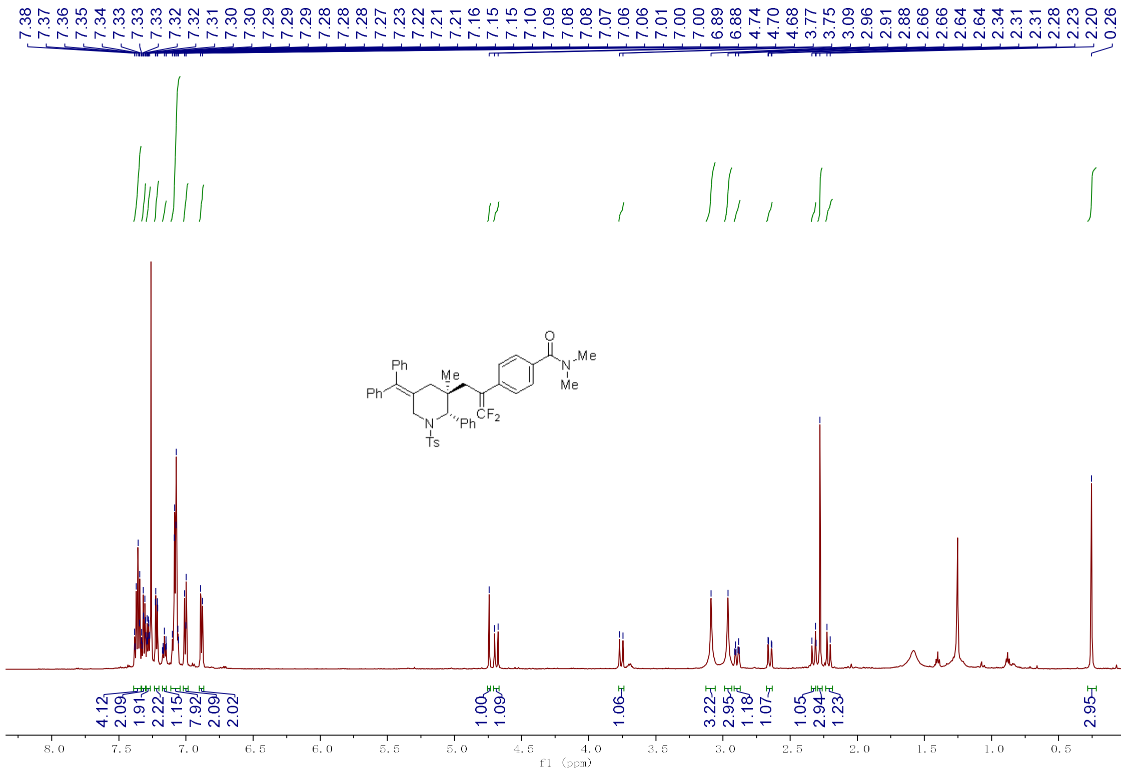

Supplementary figure 250. ^{1}H NMR of compound **82**

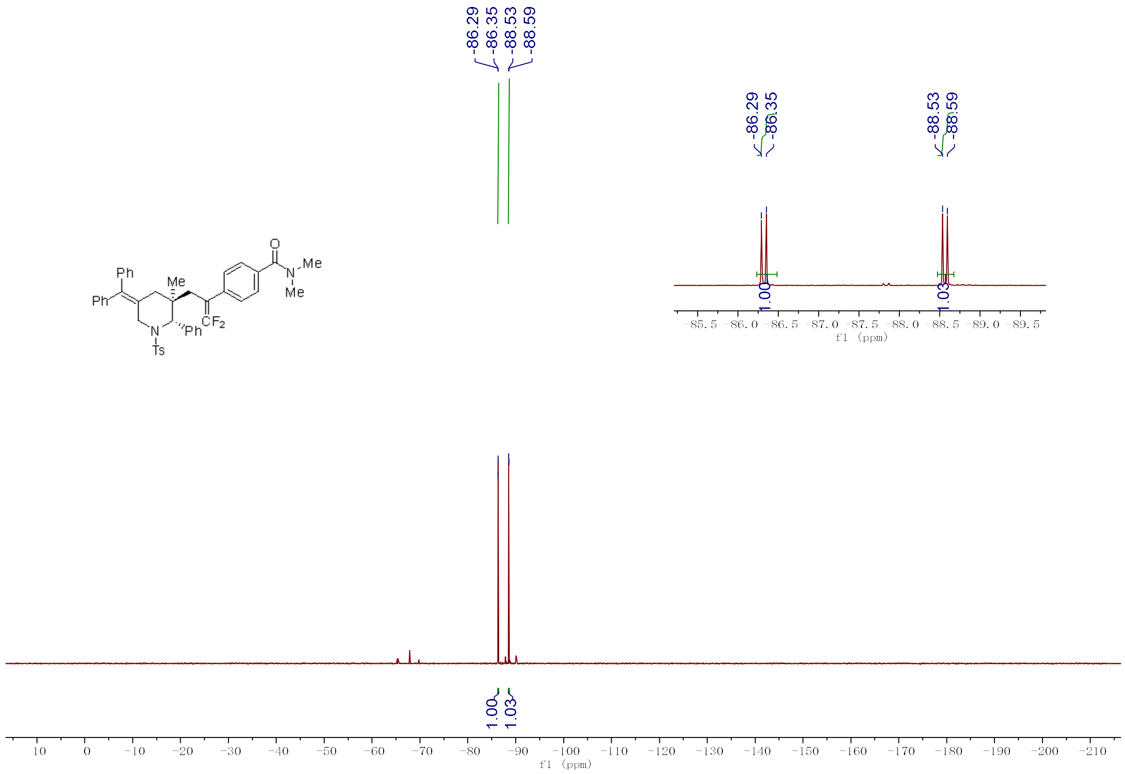

Supplementary figure 251. ^{19}F NMR of compound **82**

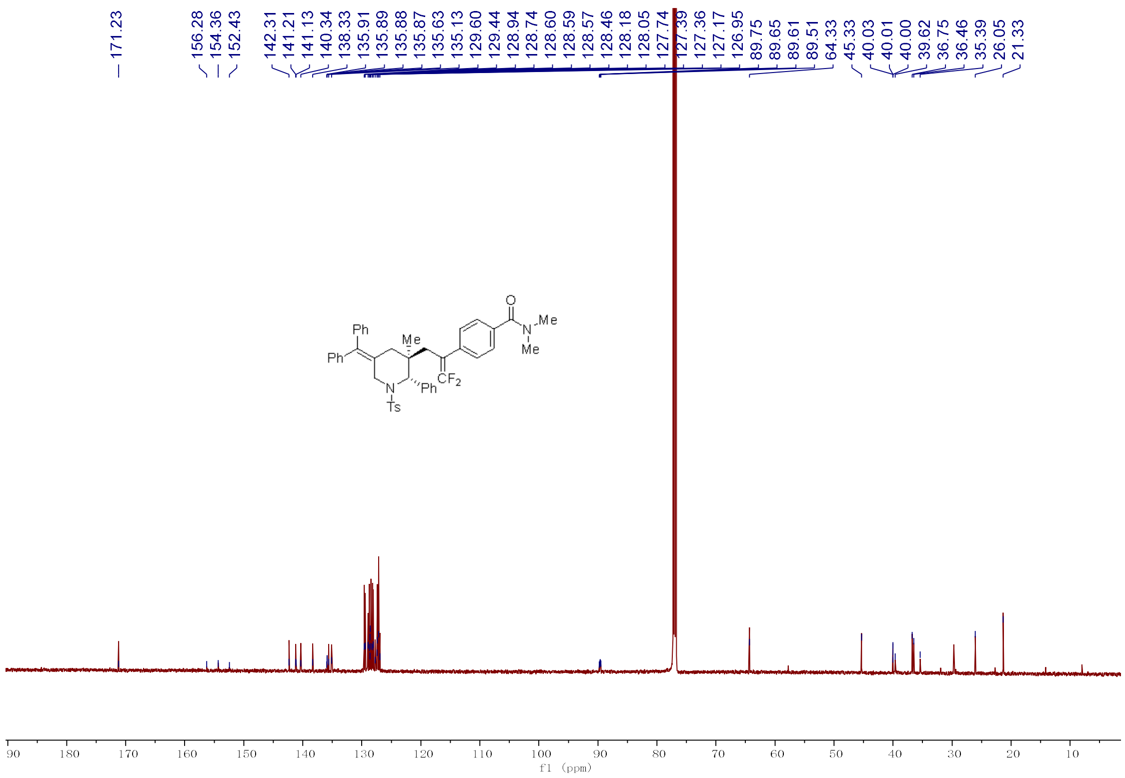

Supplementary figure 252. ^{13}C NMR of compound **82**

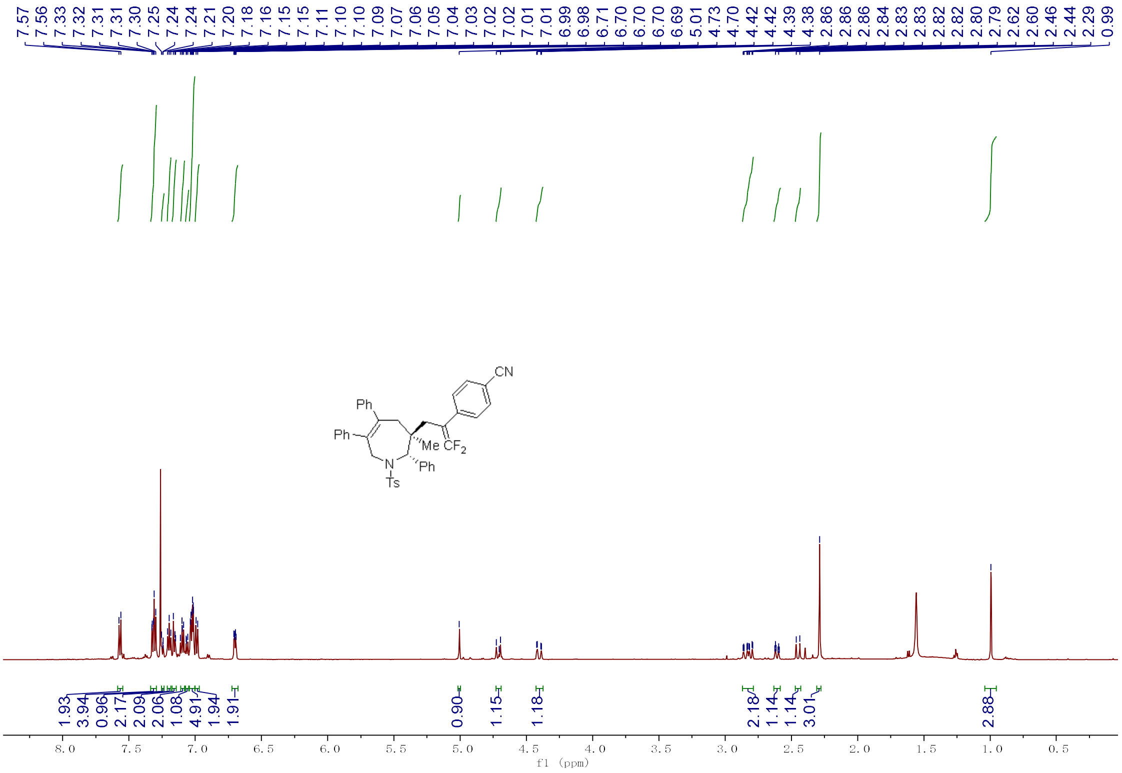

Supplementary figure 253. ^{1}H NMR of compound **83**

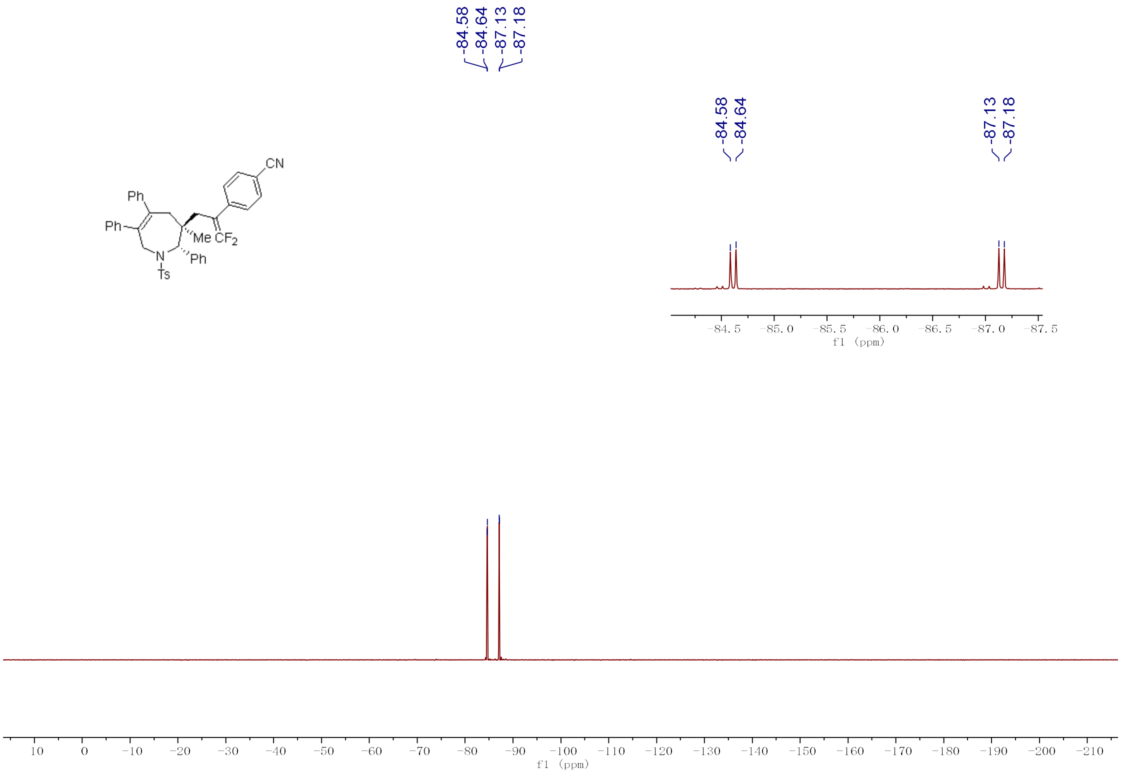

Supplementary figure 254. ^{19}F NMR of compound **83**

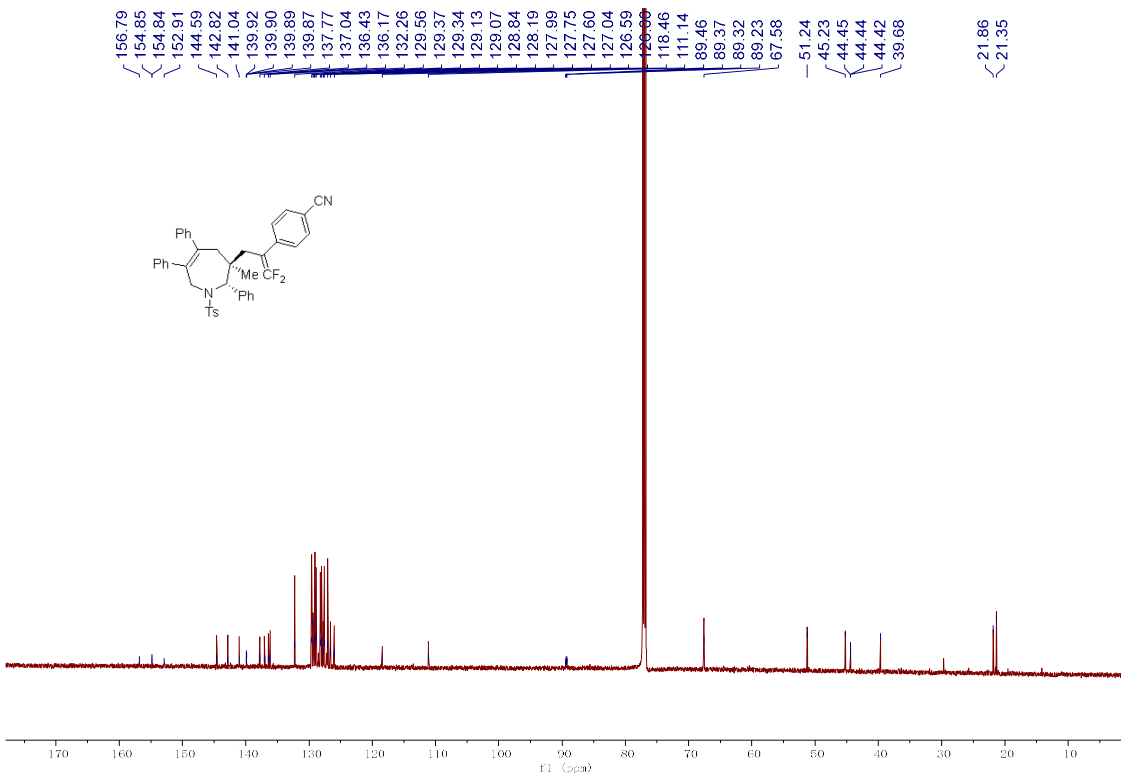

Supplementary figure 255. ^{13}C NMR of compound **83**

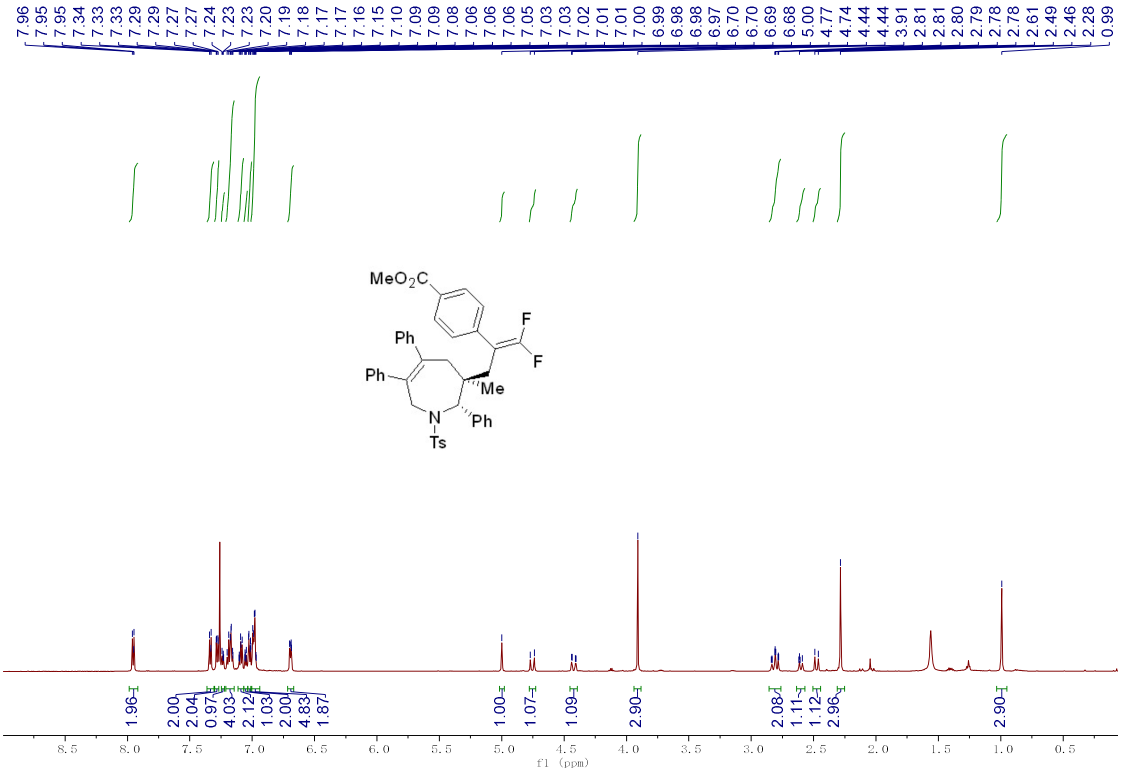

Supplementary figure 256. ^{1}H NMR of compound **84**

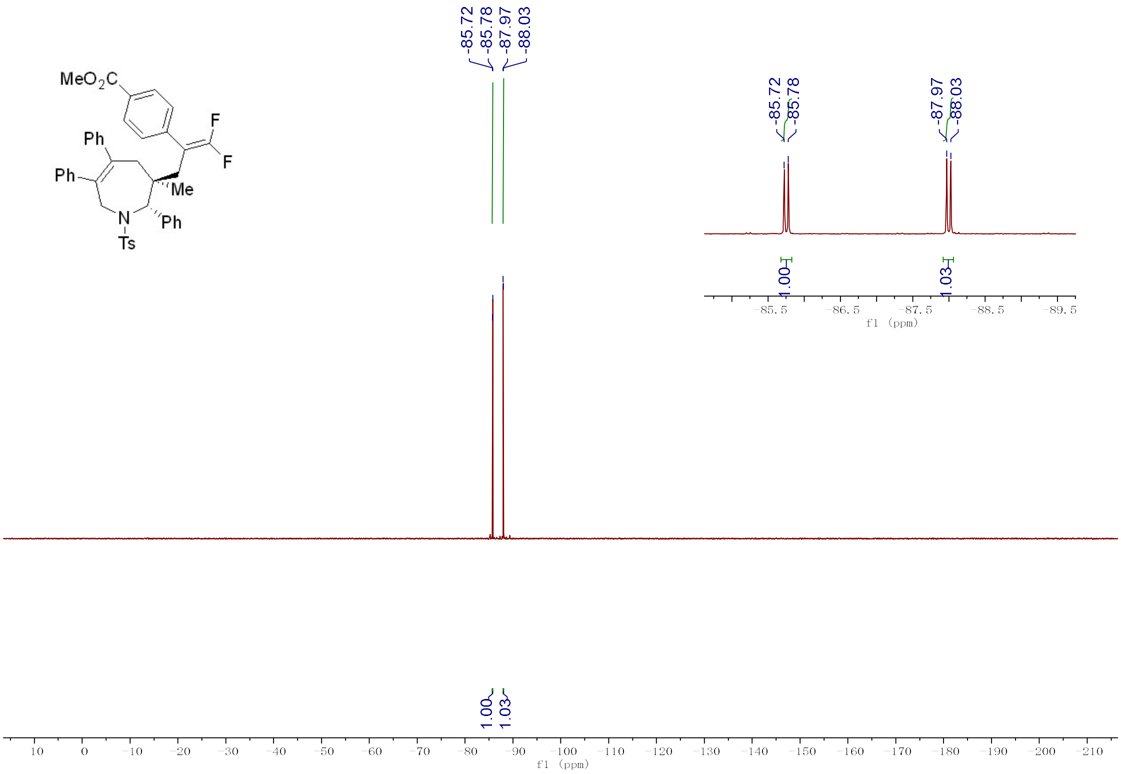

Supplementary figure 257. ^{19}F NMR of compound **84**

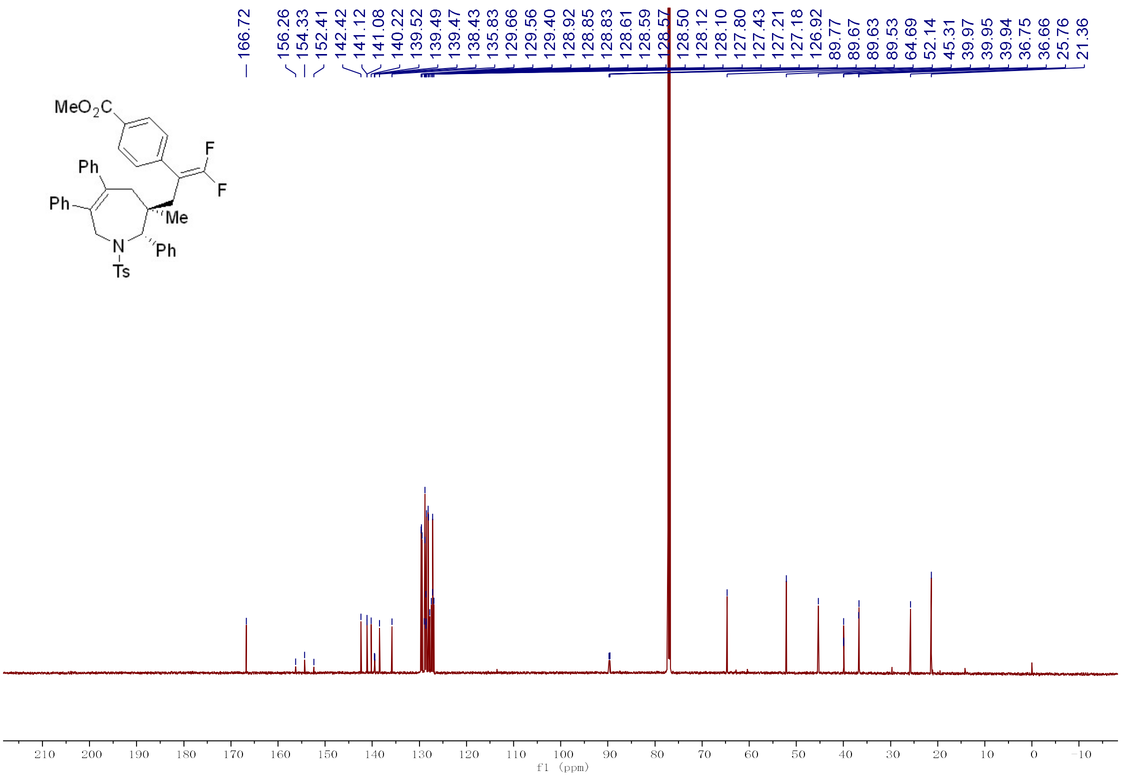

Supplementary figure 258. ^{13}C NMR of compound **84**

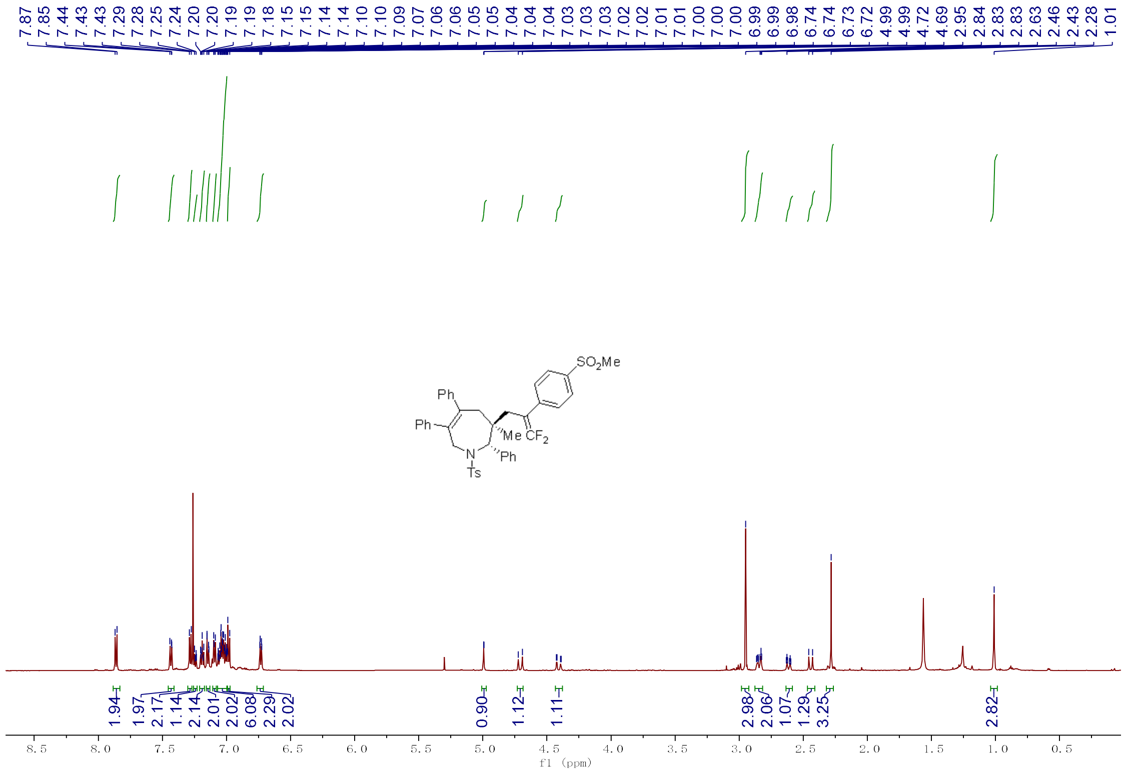

Supplementary figure 259. ^{1}H NMR of compound **85**

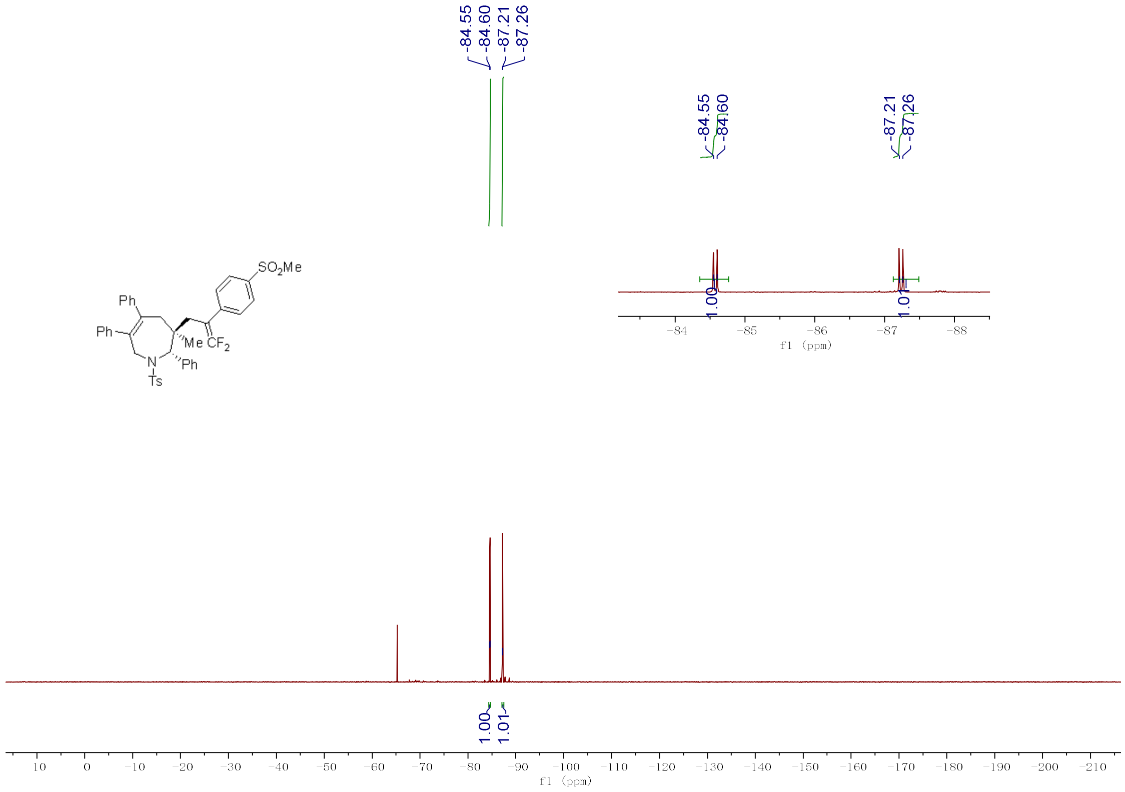

Supplementary figure 260. ^{19}F NMR of compound **85**

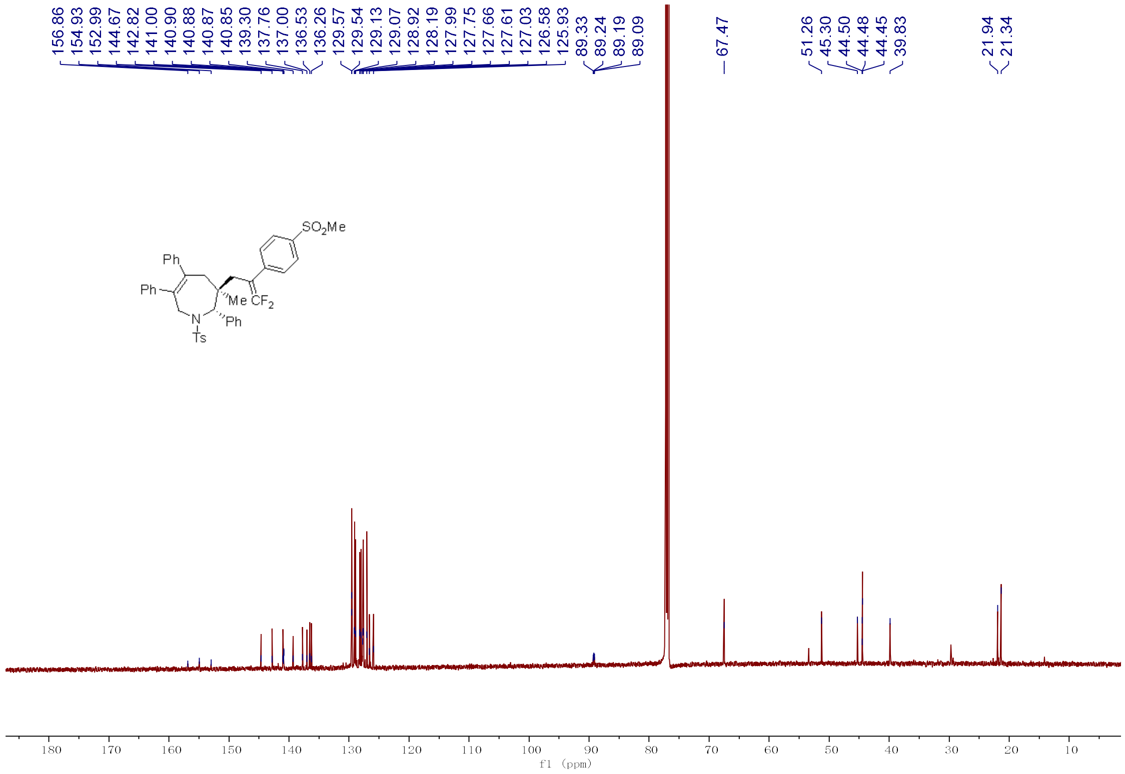

Supplementary figure 261. ^{13}C NMR of compound **85**

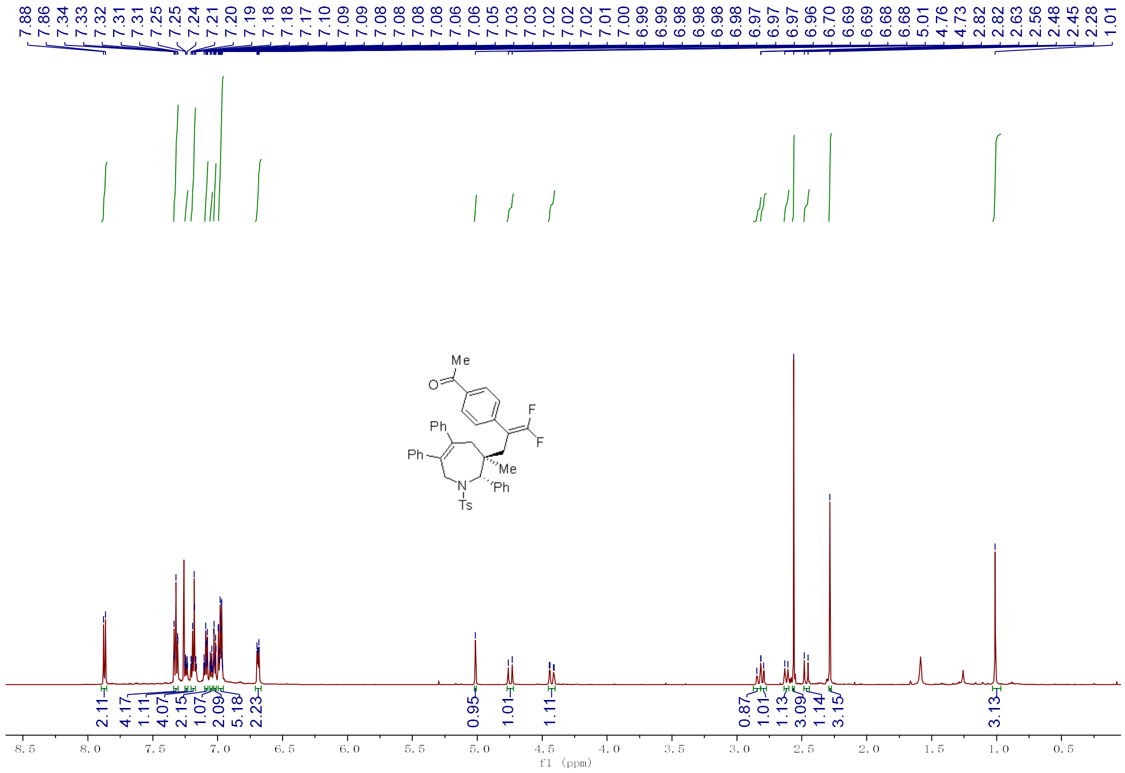

Supplementary figure 262. ^{1}H NMR of compound **86**

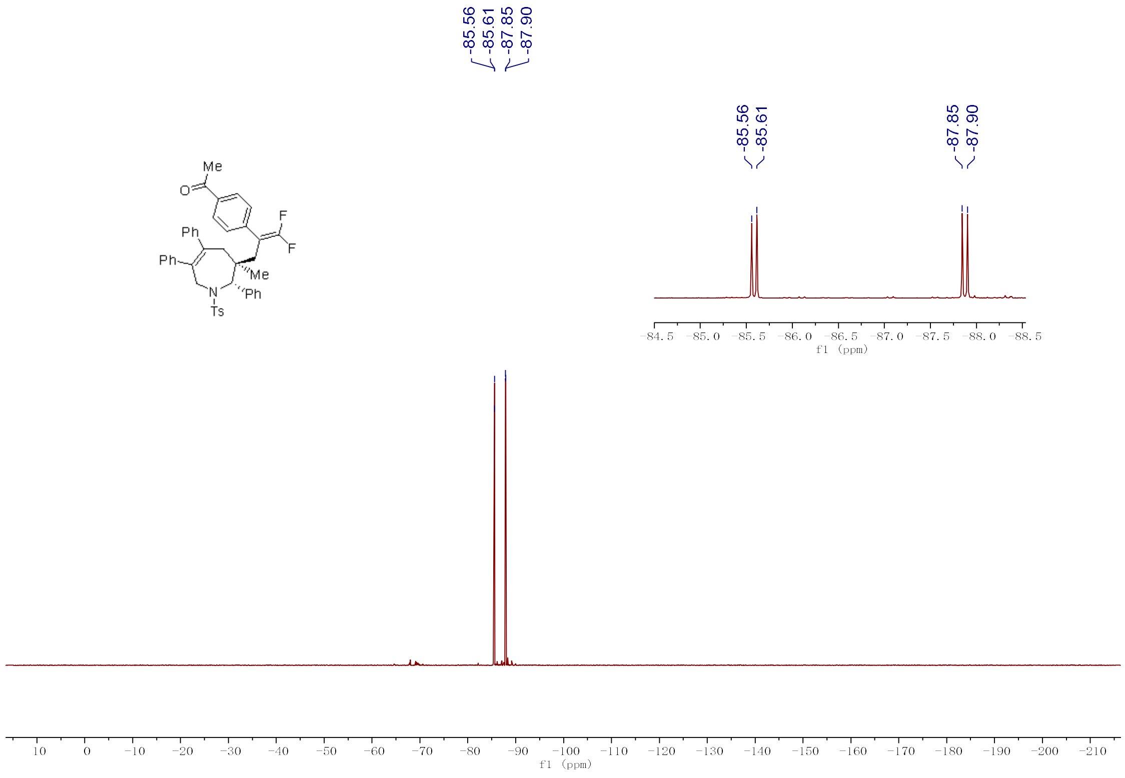

Supplementary figure 263. ^{19}F NMR of compound **86**

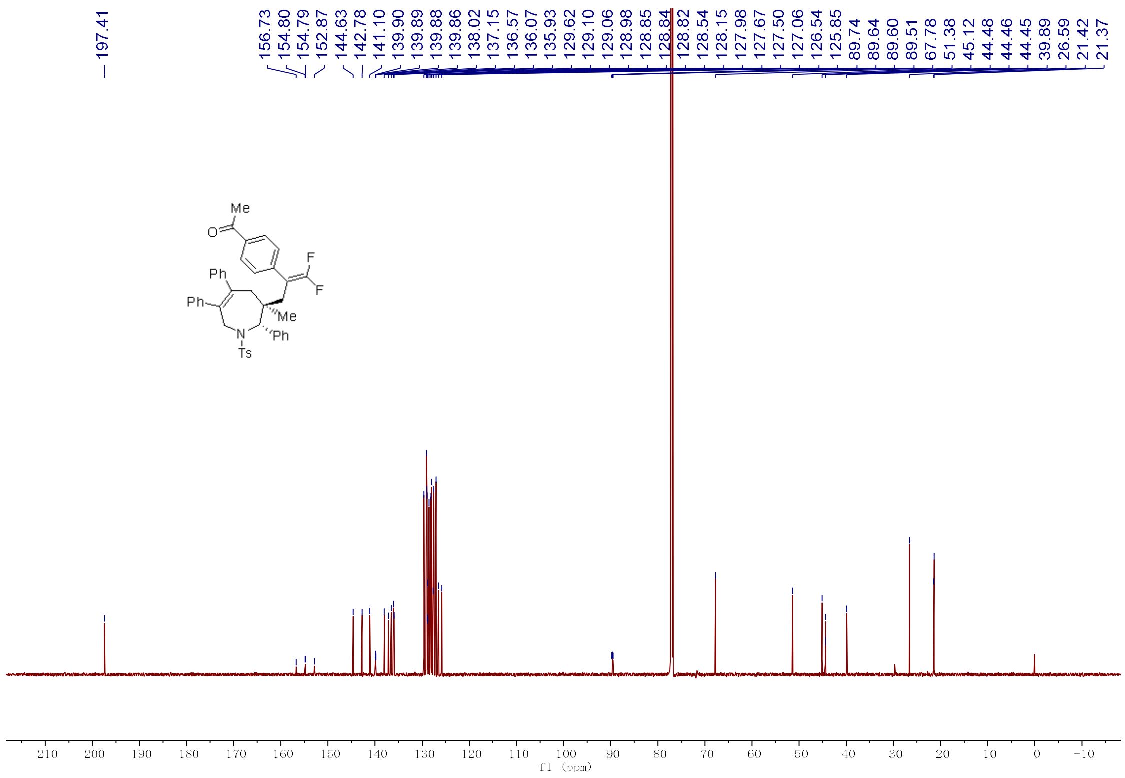

Supplementary figure 264. ^{13}C NMR of compound **86**

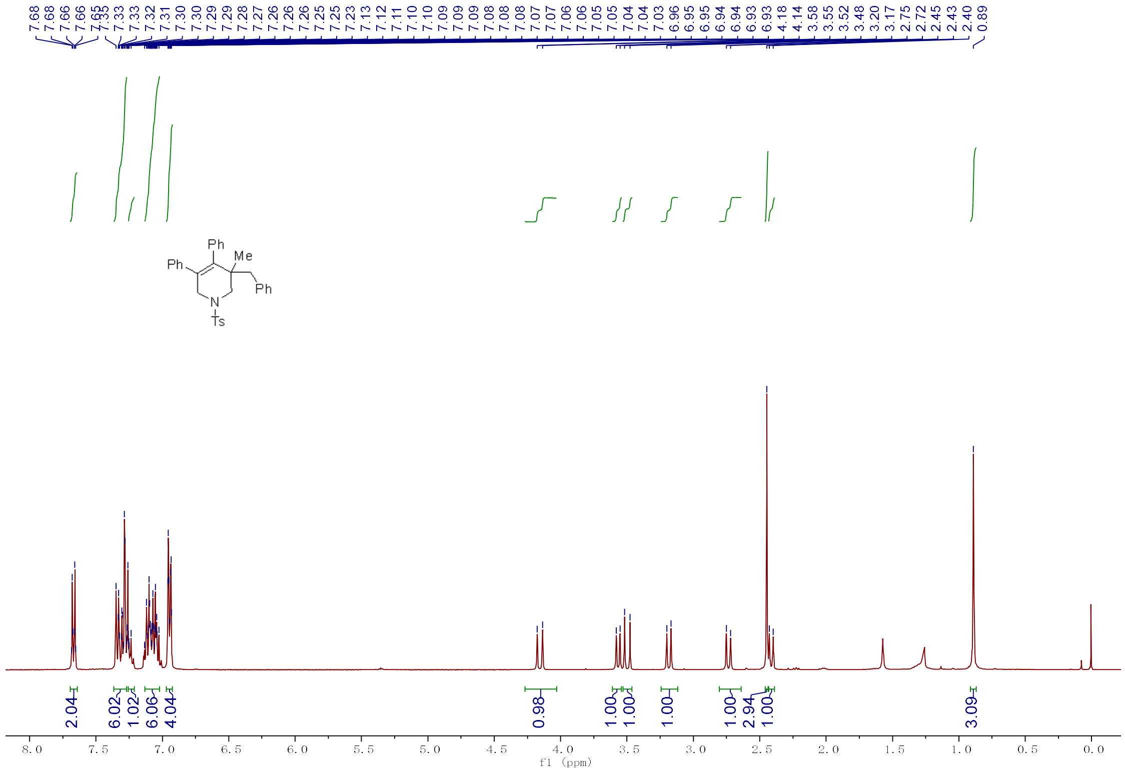

Supplementary figure 265. ^{1}H NMR of compound **87**

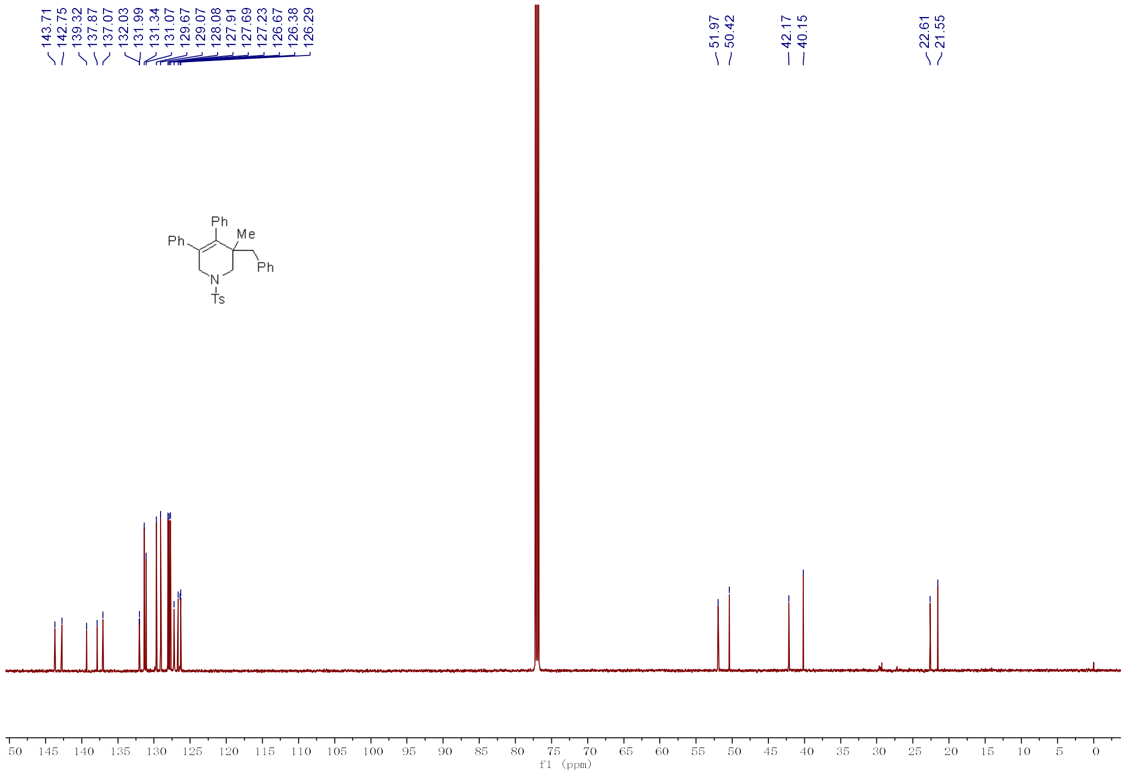

Supplementary figure 266. ^{13}C NMR of compound **87**

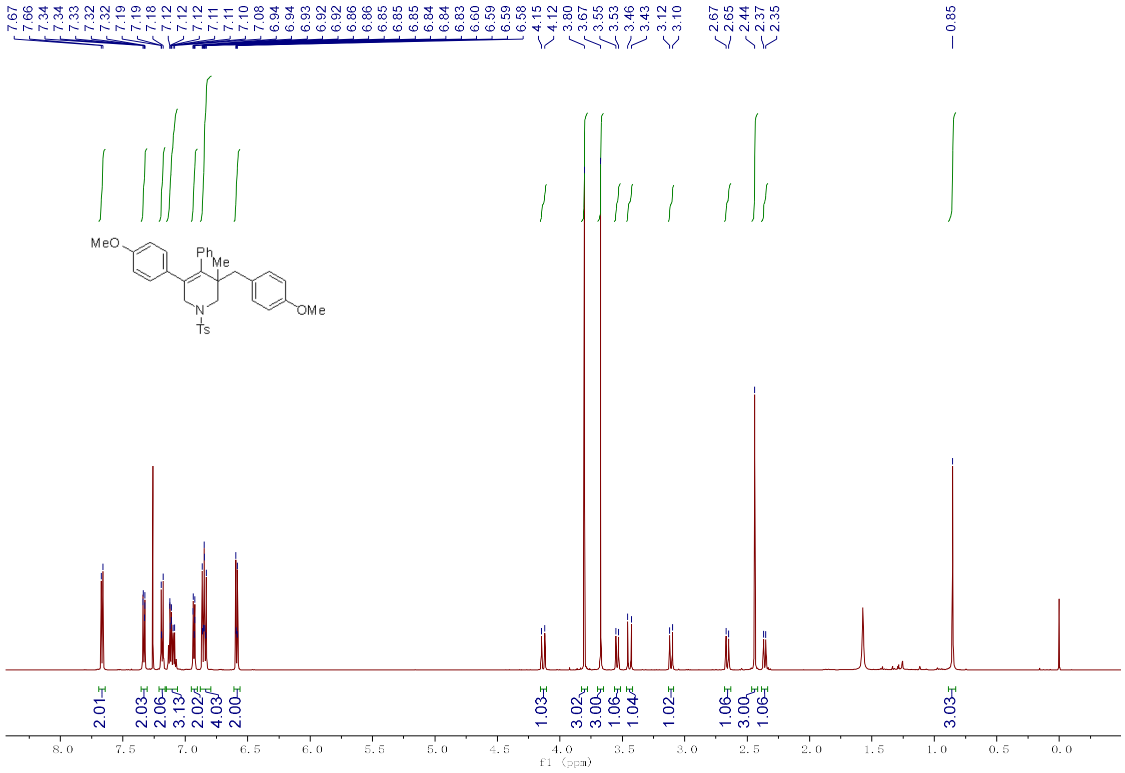

Supplementary figure 267. ^{1}H NMR of compound **88**

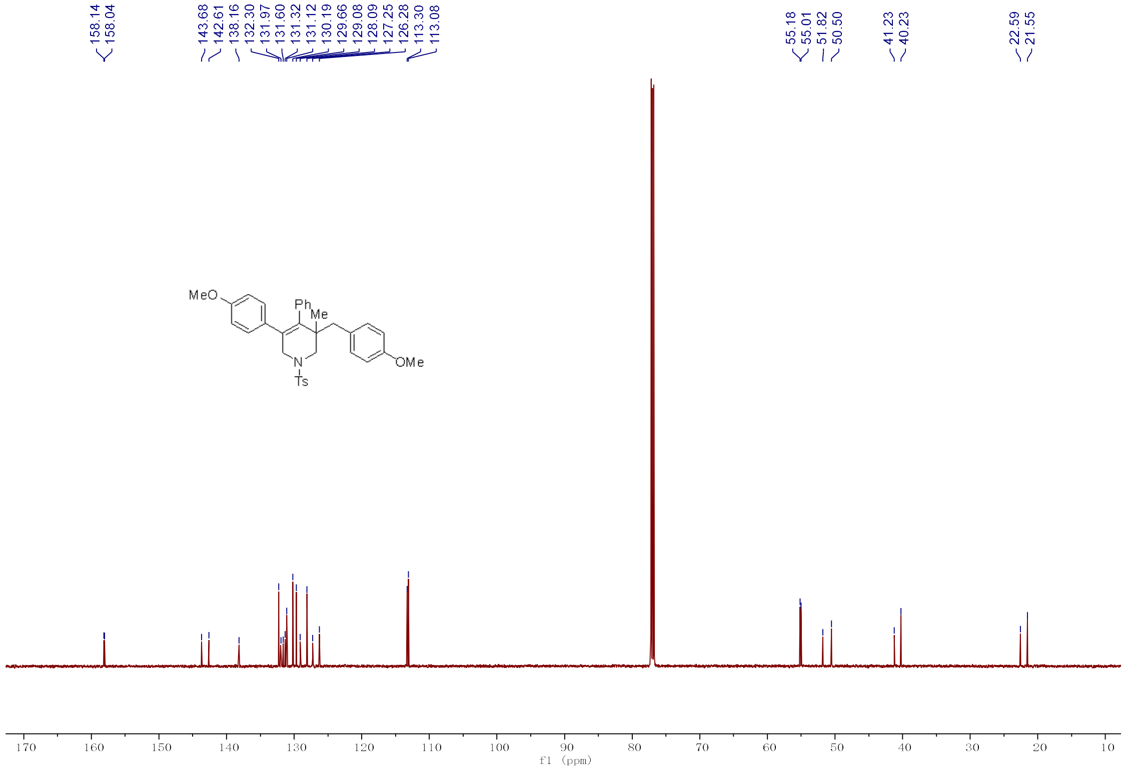

Supplementary figure 268. ^{13}C NMR of compound **88**

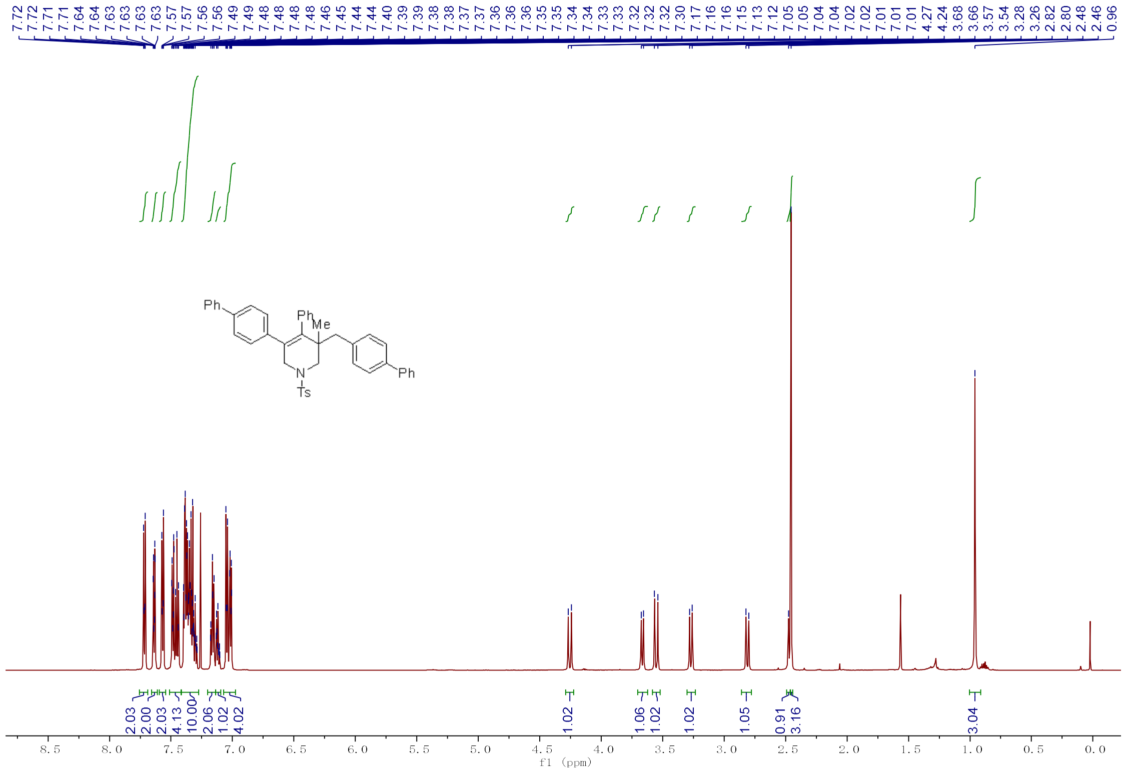

Supplementary figure 269. ^{1}H NMR of compound **89**

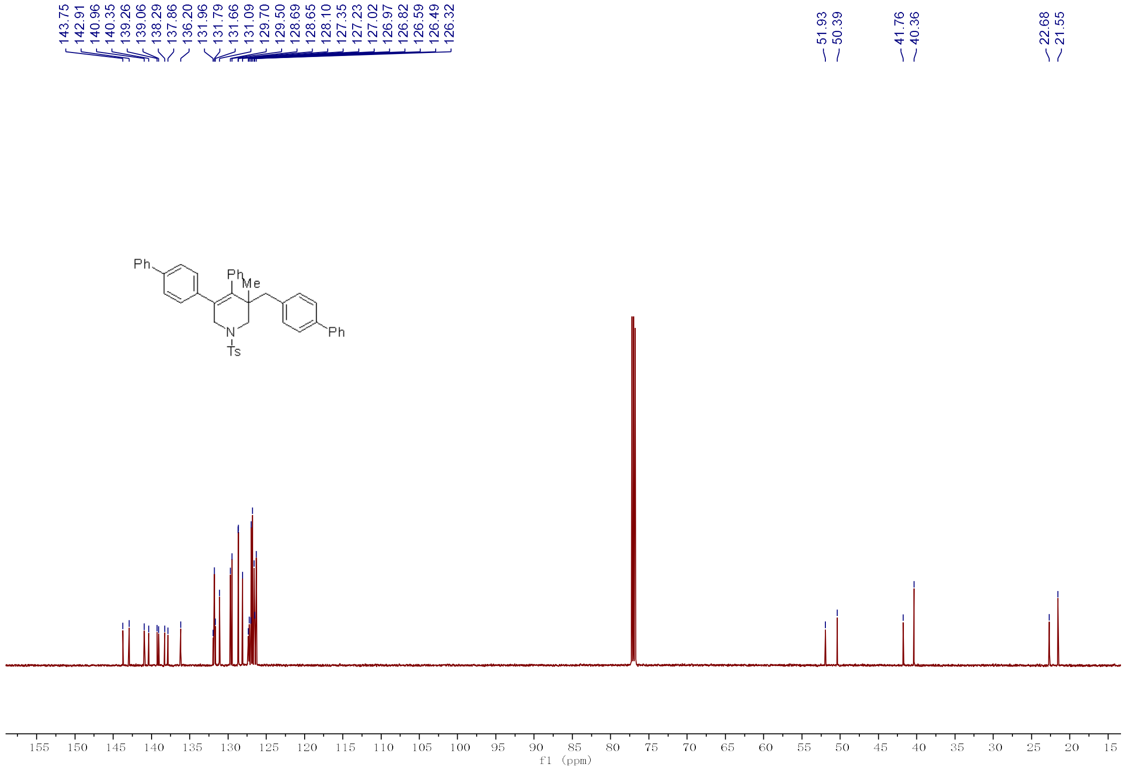

Supplementary figure 270. ^{13}C NMR of compound **89**

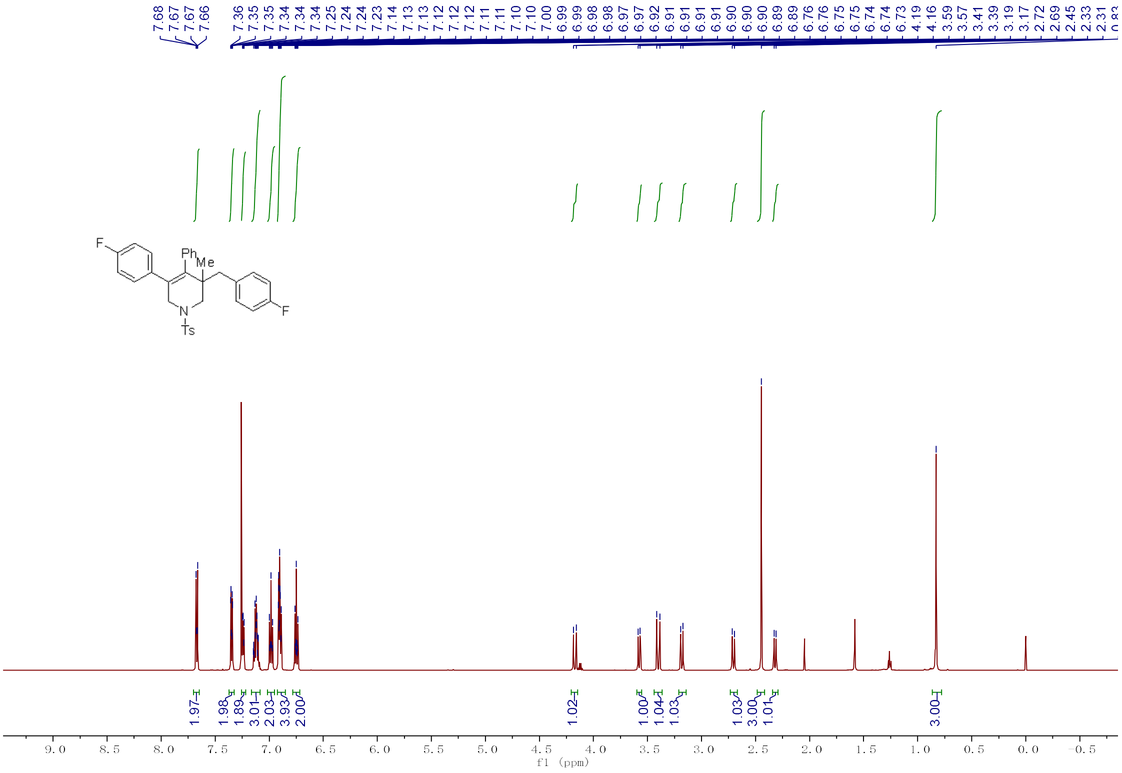

Supplementary figure 271. ^{1}H NMR of compound **90**

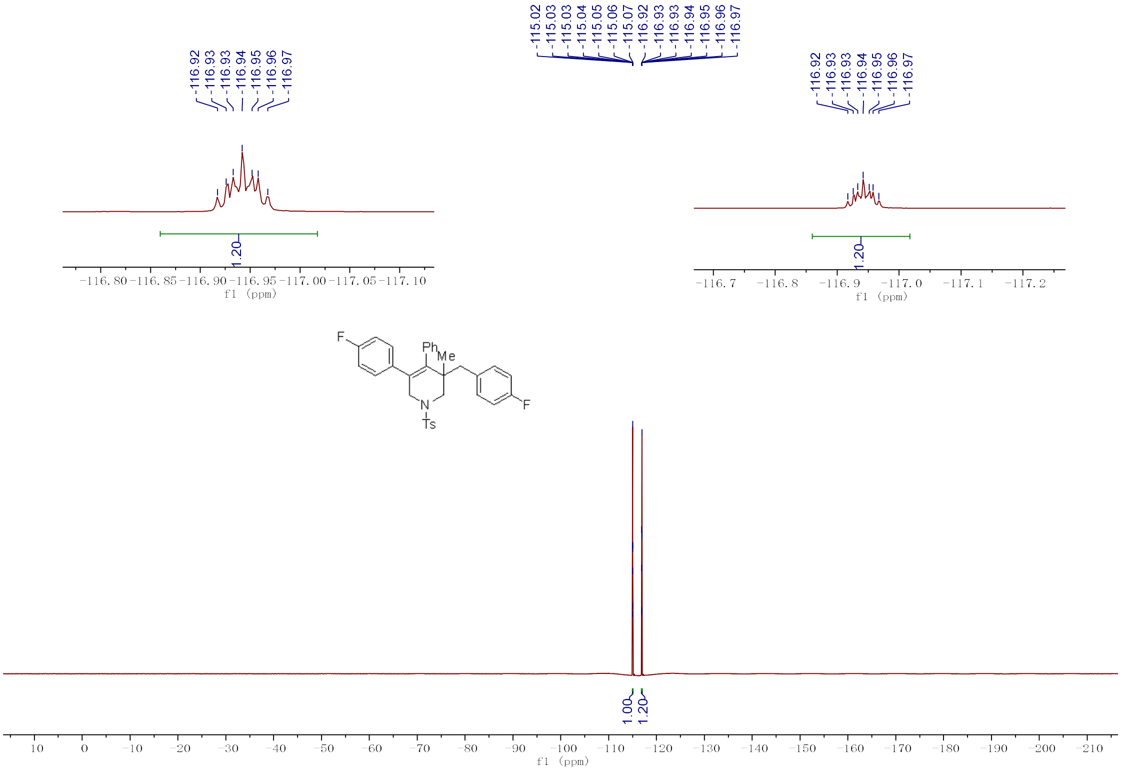

Supplementary figure 272. ^{19}F NMR of compound **90**

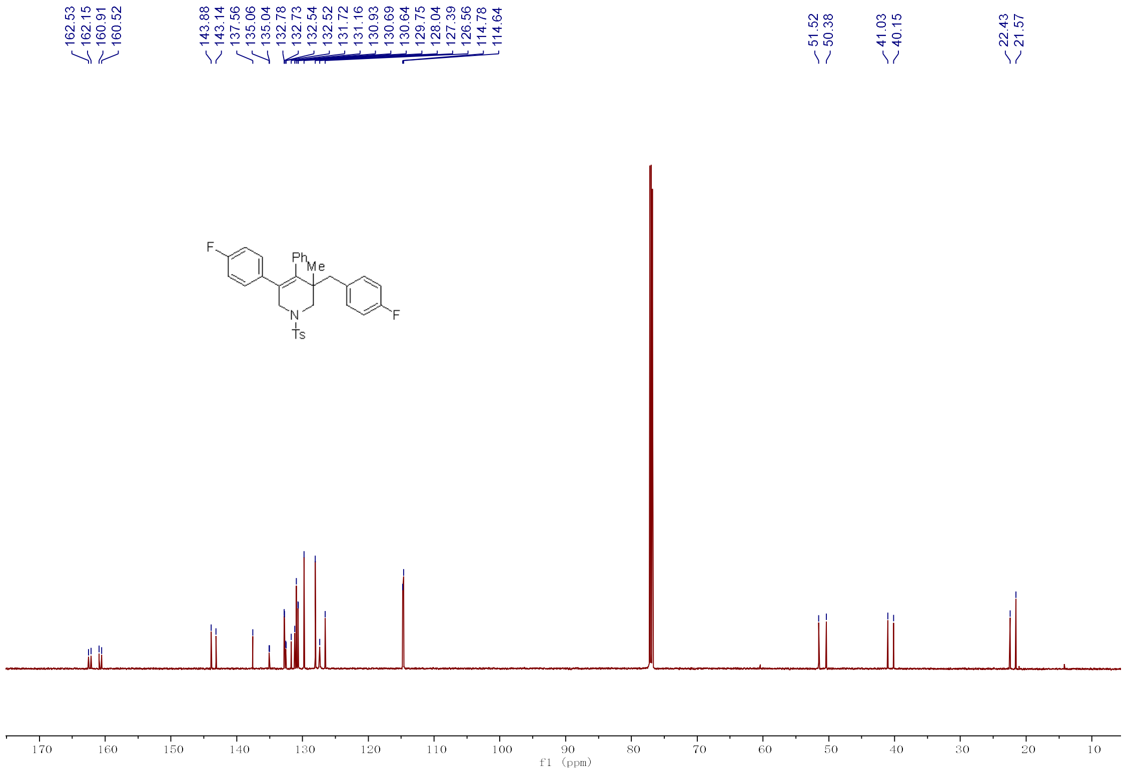

Supplementary figure 273. ^{13}C NMR of compound **90**

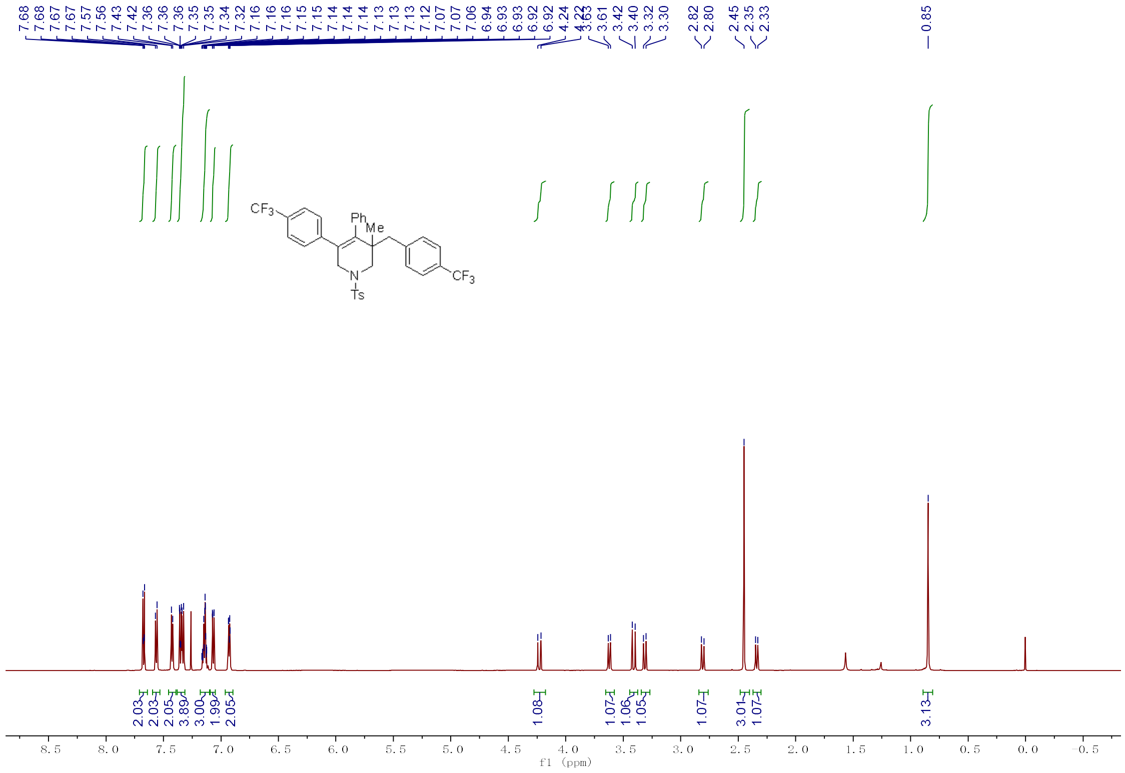

Supplementary figure 274. ^{1}H NMR of compound **91**

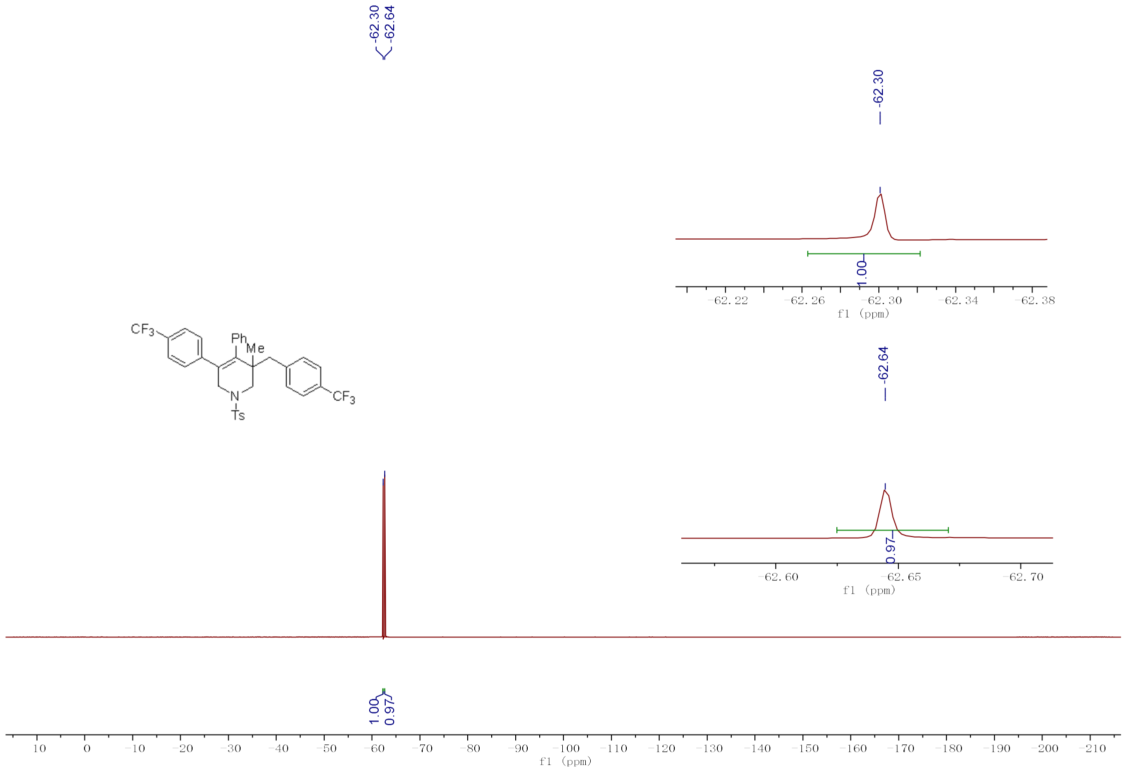

Supplementary figure 275. ^{19}F NMR of compound **91**

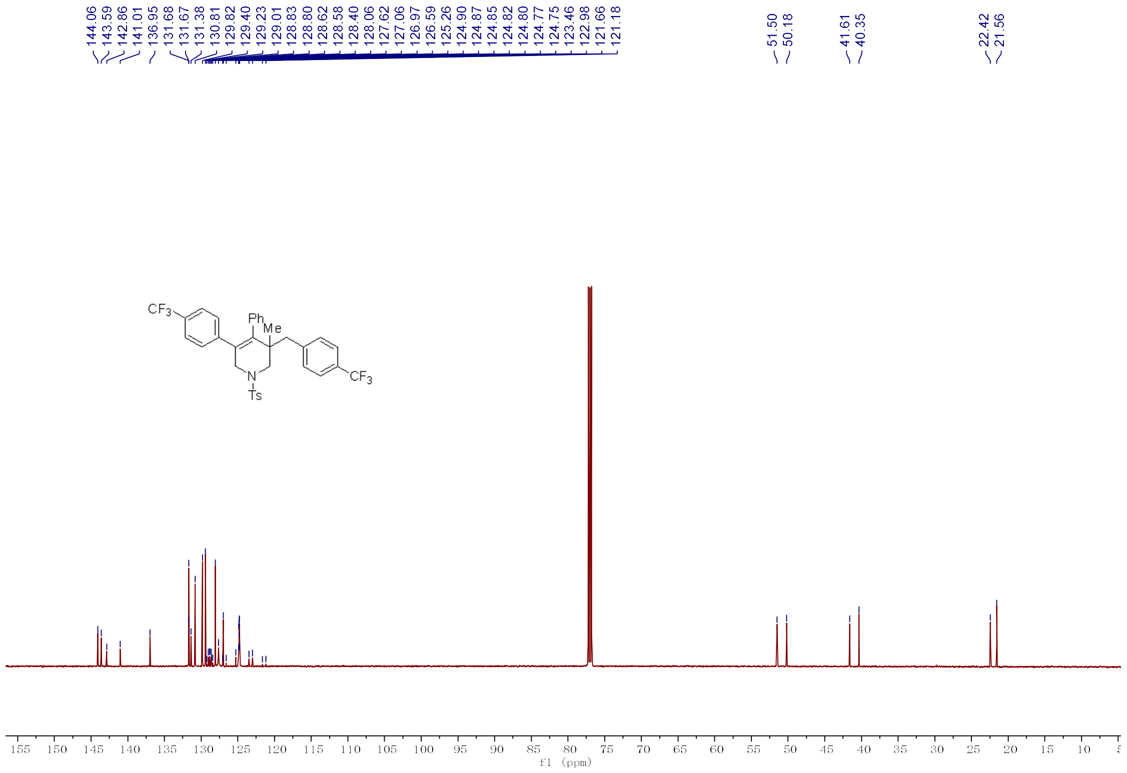

Supplementary figure 276. ^{13}C NMR of compound **91**

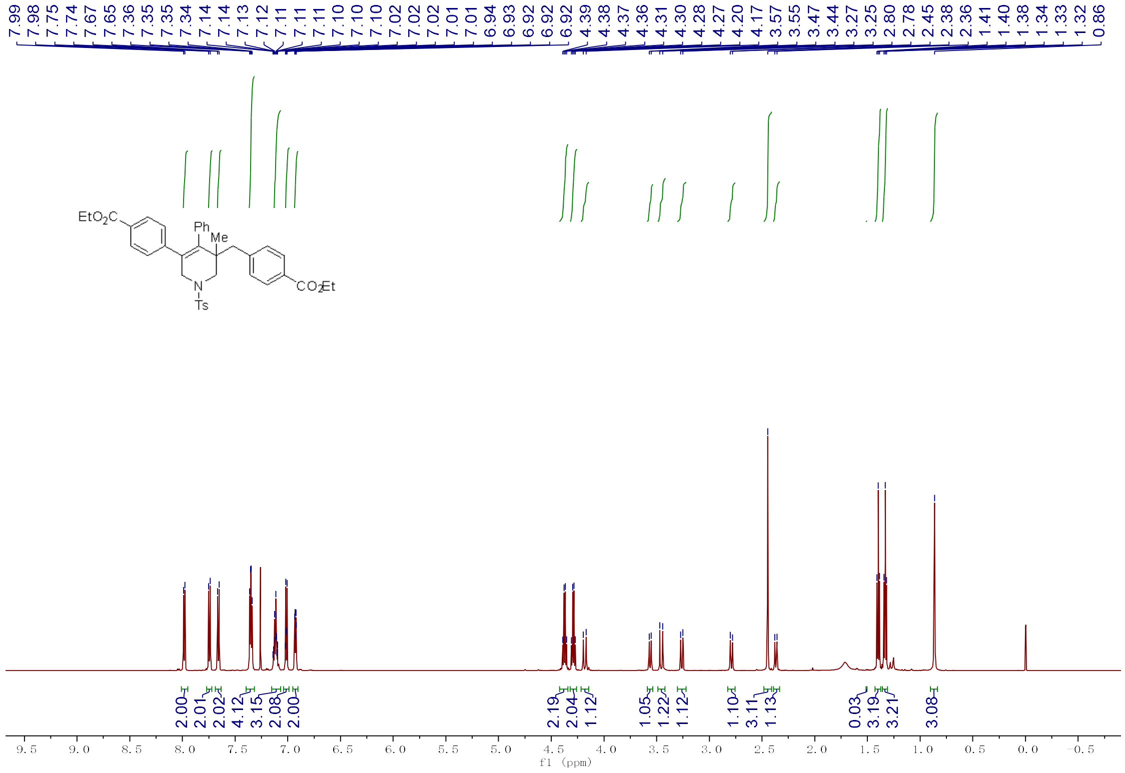

Supplementary figure 277. ^{1}H NMR of compound **92**

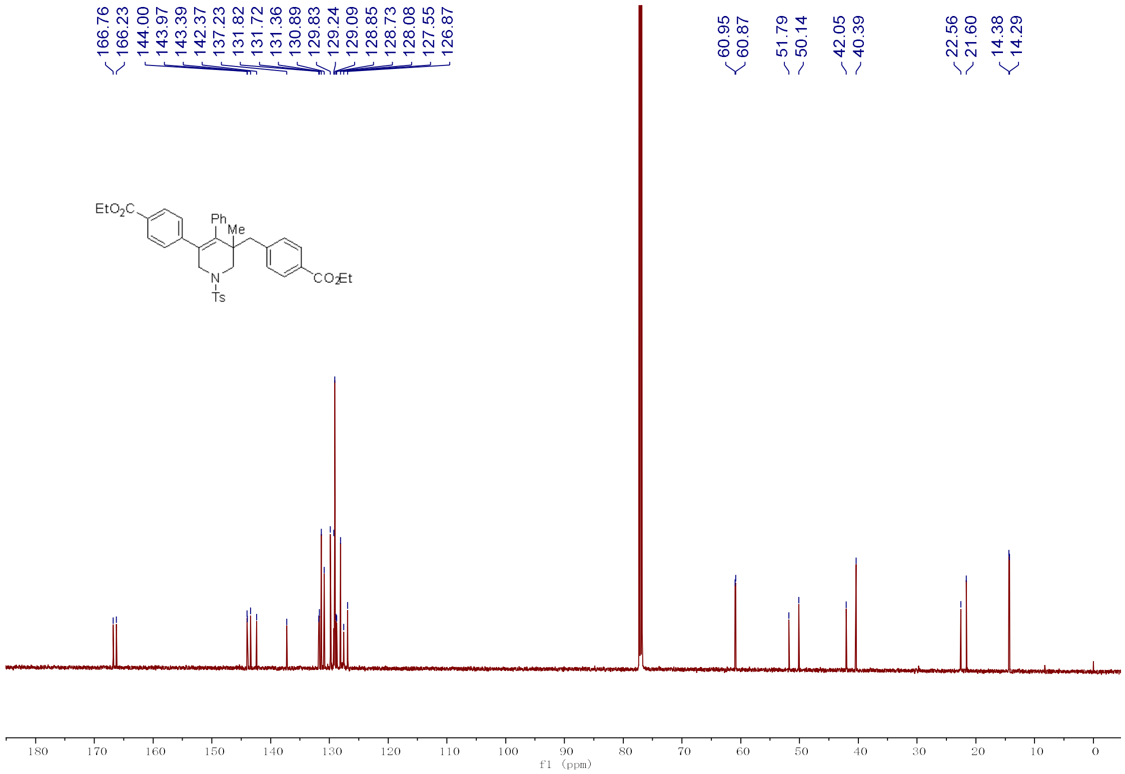

Supplementary figure 278. ^{13}C NMR of compound **92**

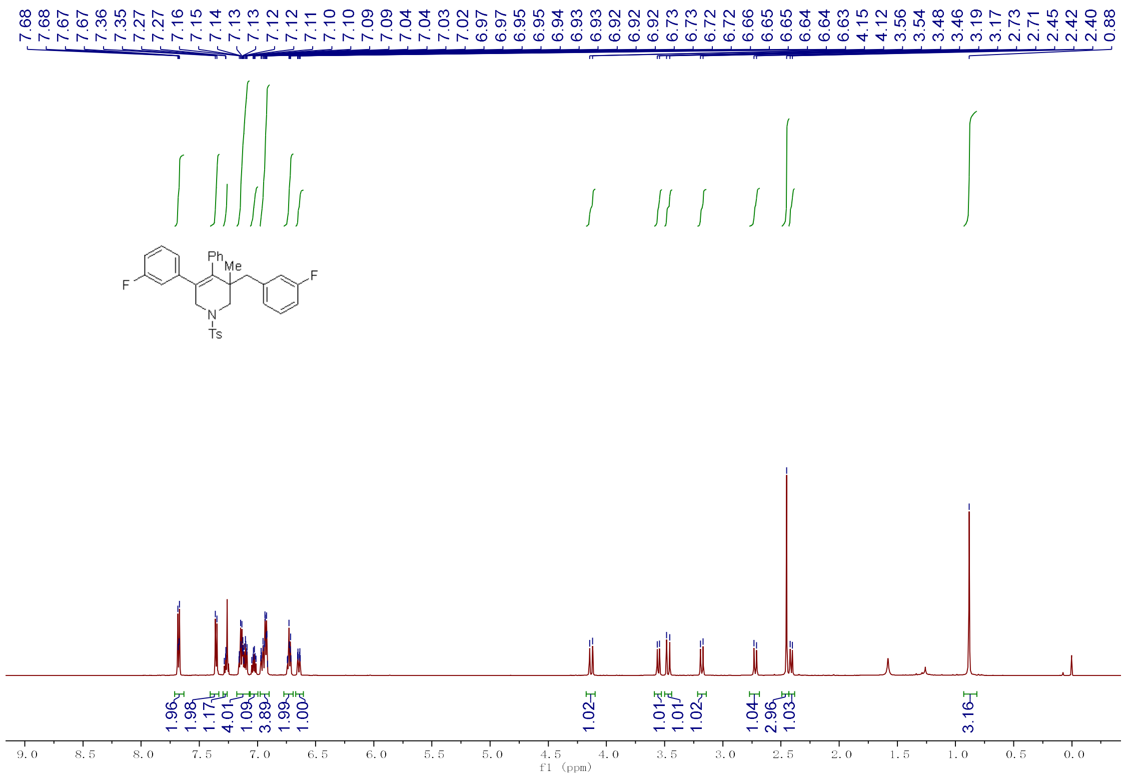

Supplementary figure 279. ^{1}H NMR of compound **93**

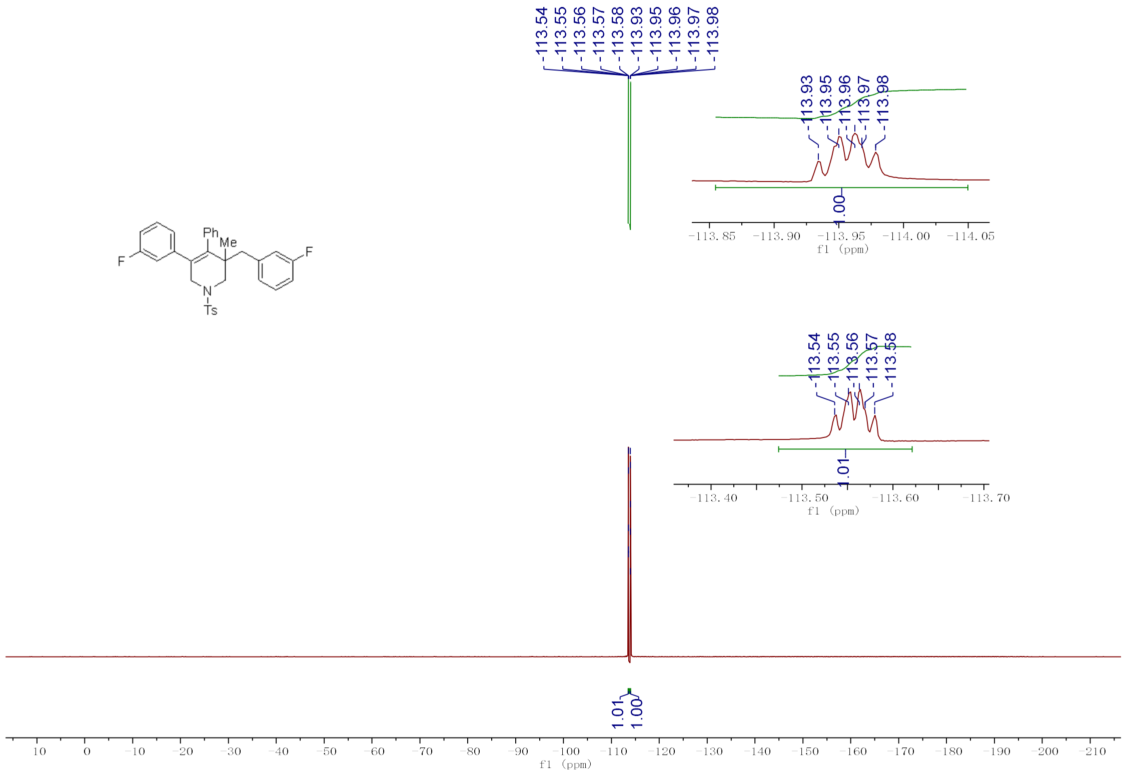

Supplementary figure 280. ^{19}F NMR of compound **93**

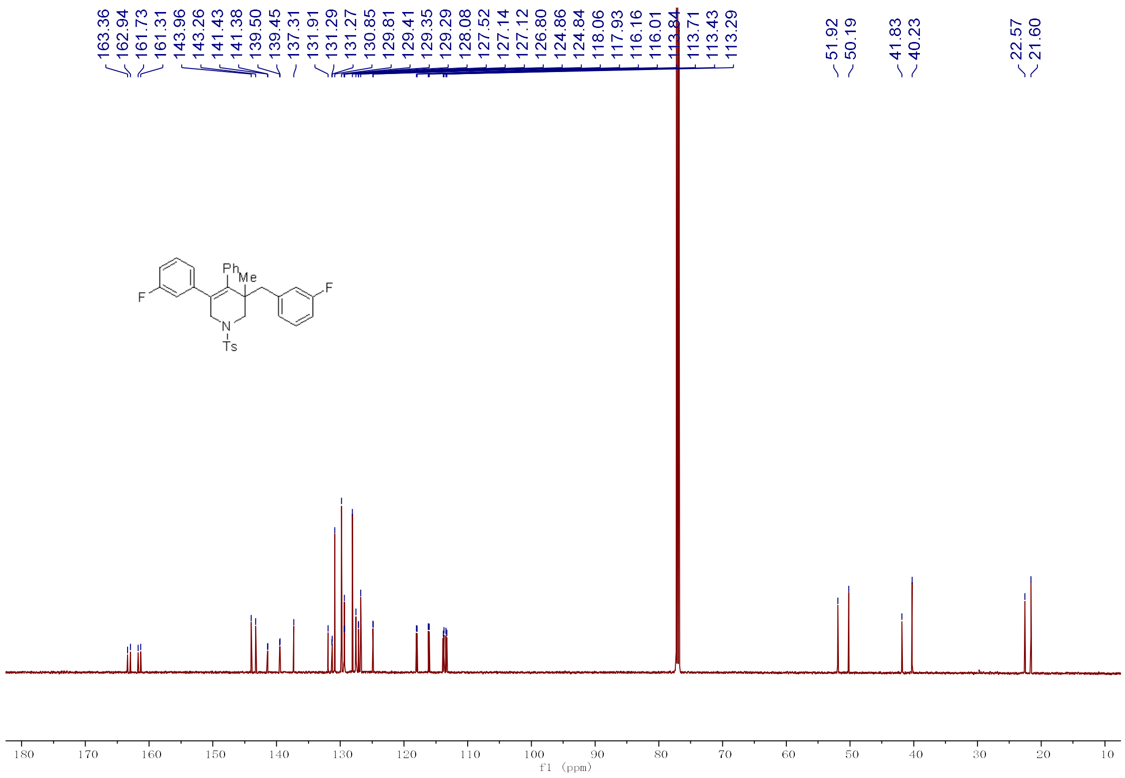

Supplementary figure 281. ^{13}C NMR of compound **93**

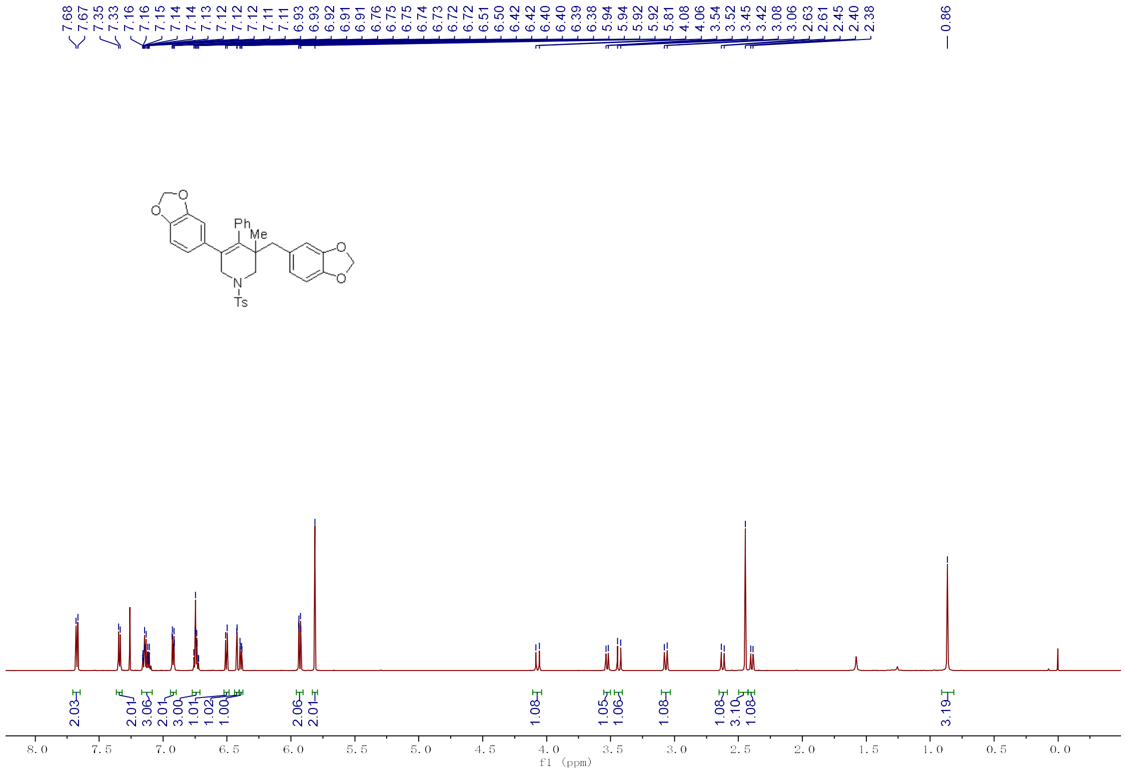

Supplementary figure 282. ^{1}H NMR of compound **94**

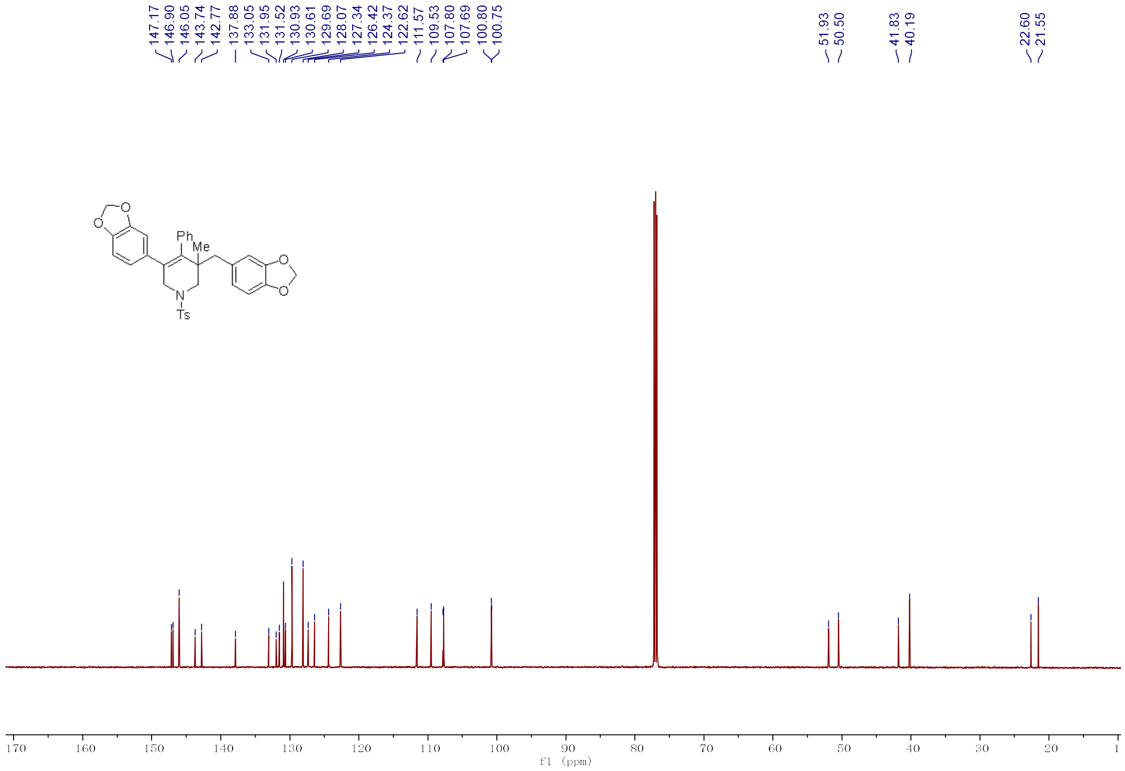

Supplementary figure 283. ^{13}C NMR of compound **94**

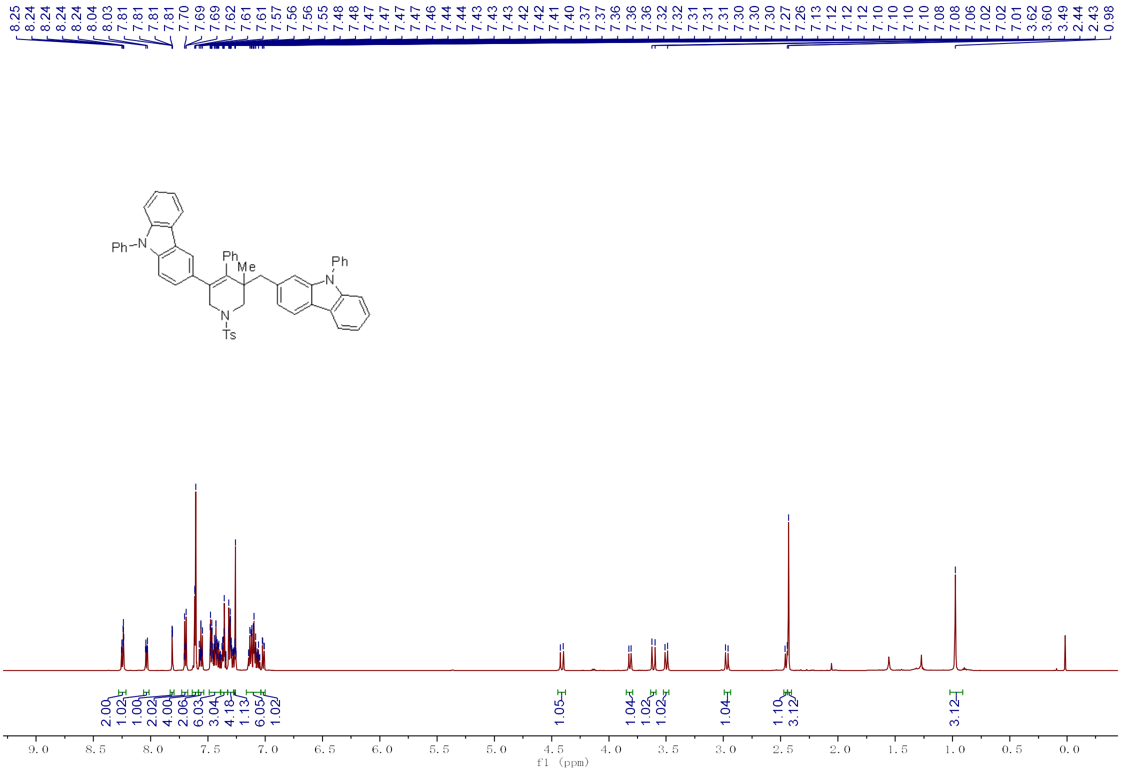

Supplementary figure 284. ^{1}H NMR of compound **95**

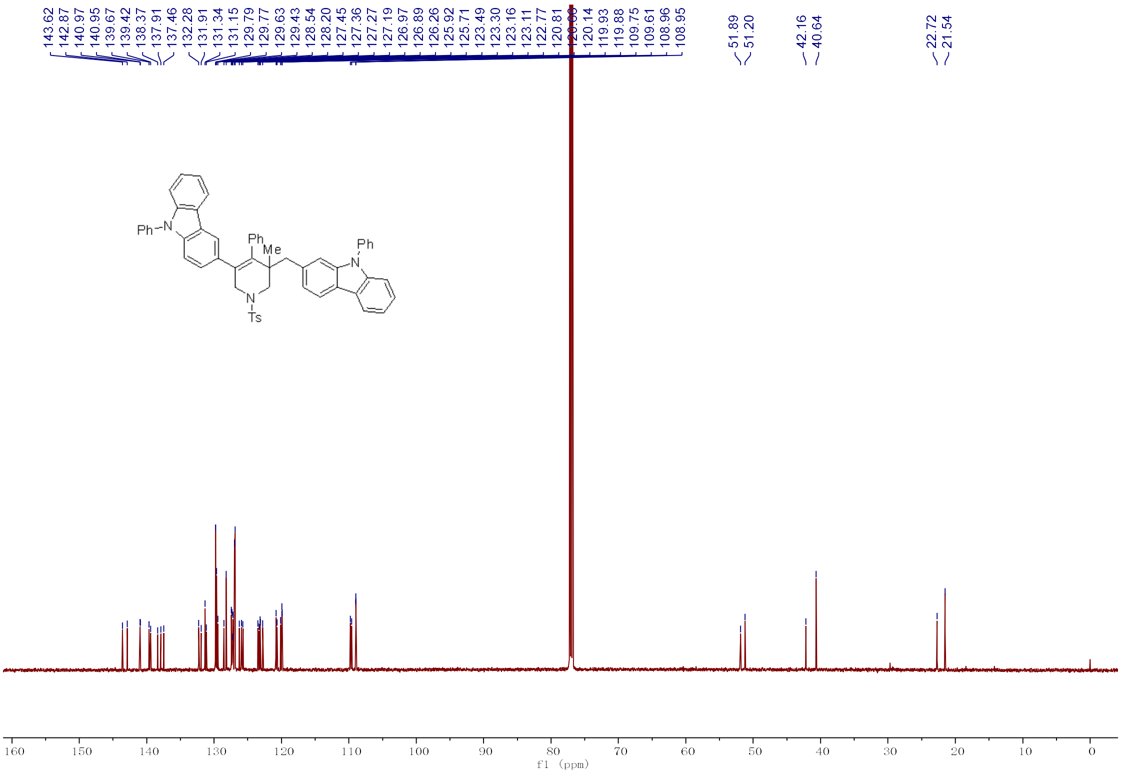

Supplementary figure 285. ^{13}C NMR of compound **95**

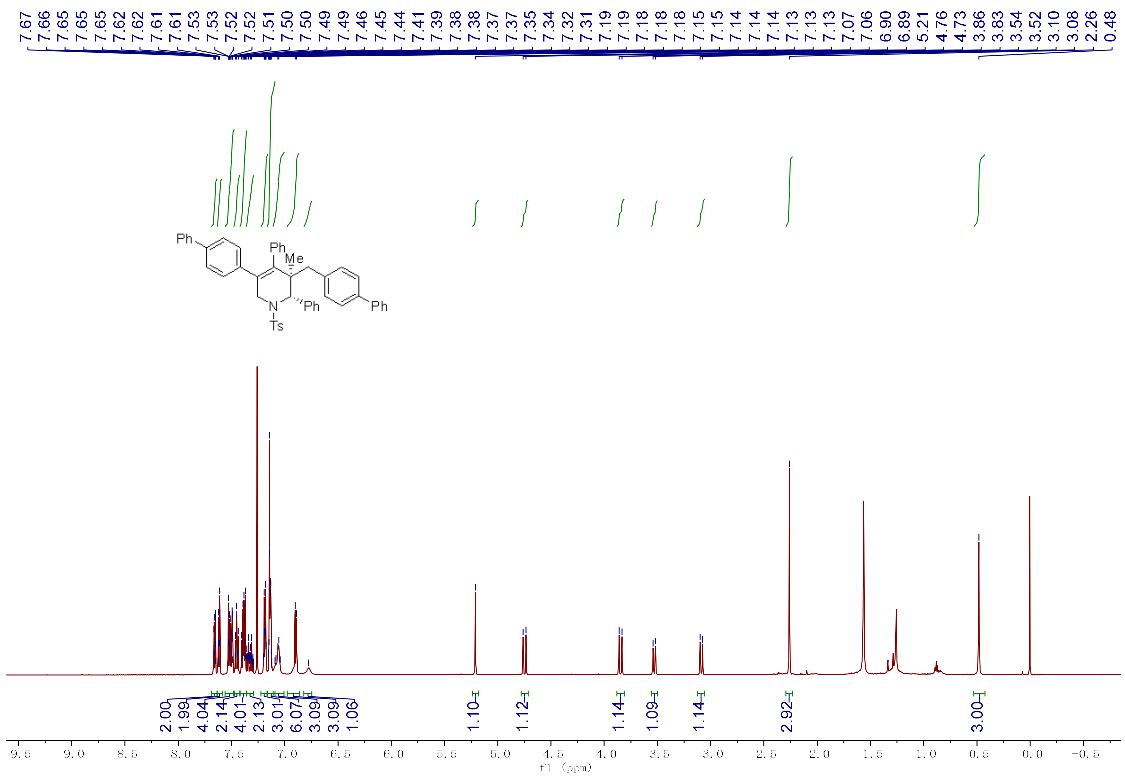

Supplementary figure 286. ^{1}H NMR of compound **96**

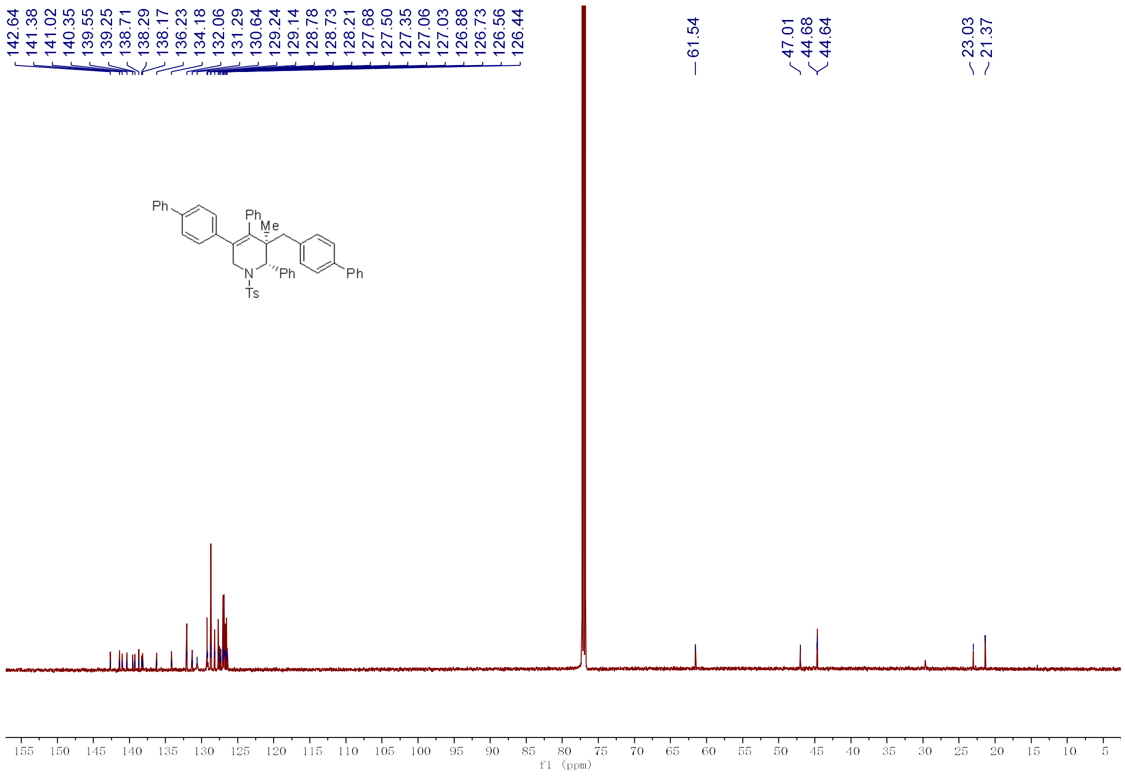

Supplementary figure 287. ^{13}C NMR of compound **96**

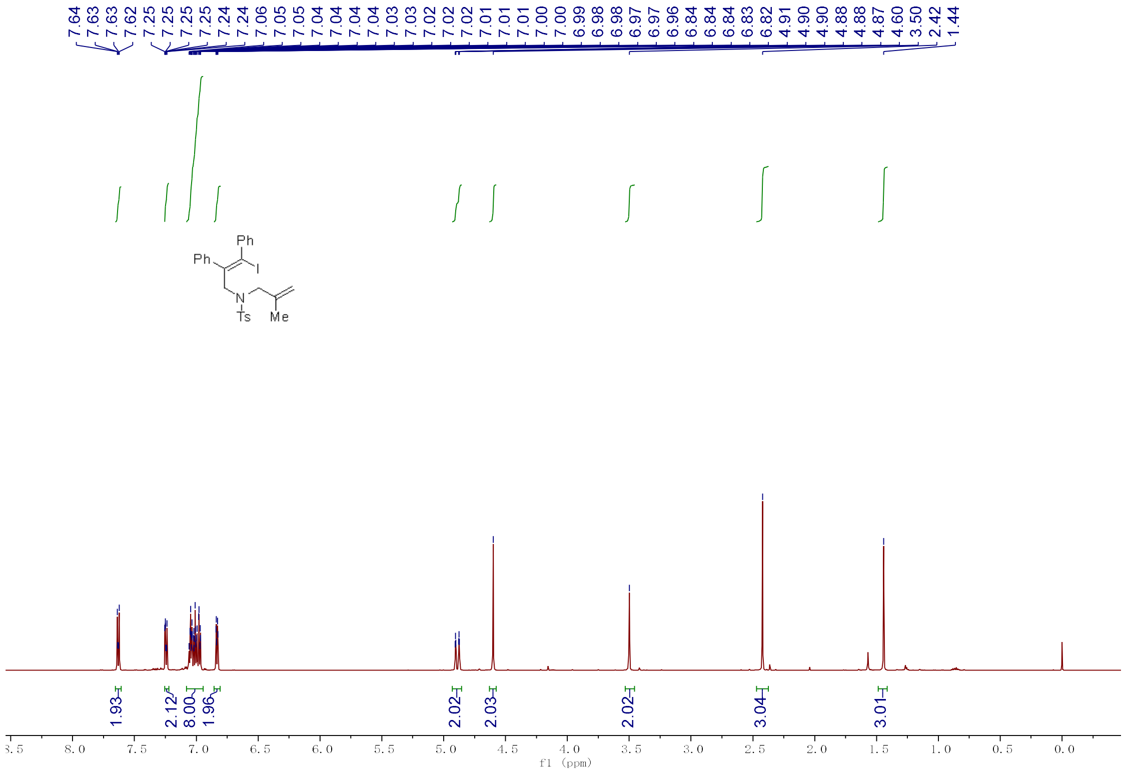

Supplementary figure 288. ^{1}H NMR of compound **100**

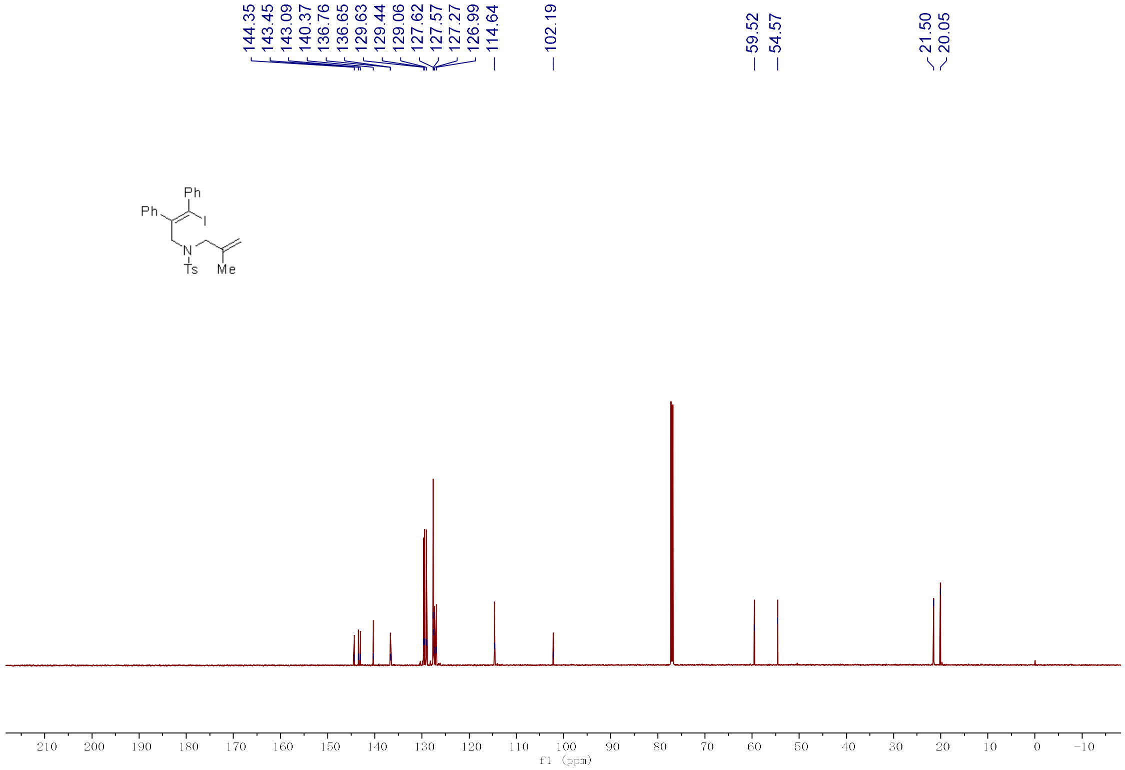

Supplementary figure 289. ^{13}C NMR of compound **100**

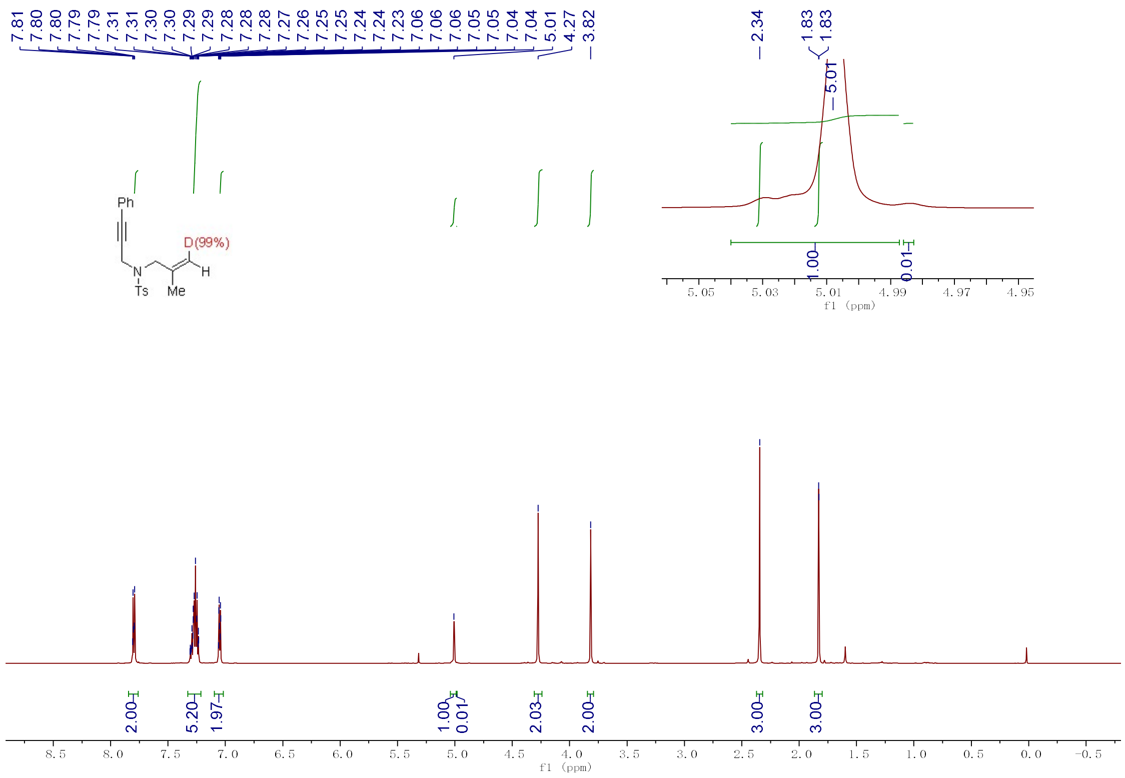

Supplementary figure 290. ^{1}H NMR of compound **(Z)-107-D**

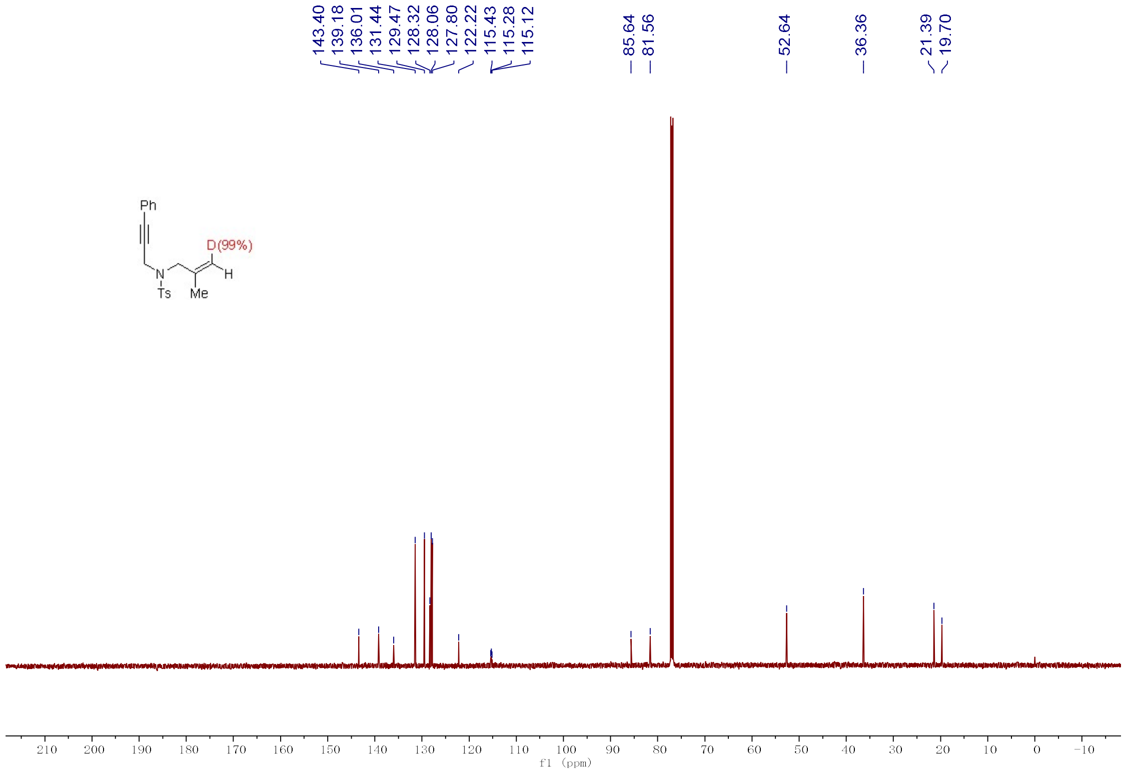

Supplementary figure 291. ^{13}C NMR of compound **(Z)-107-D**

3 Supplementary References

1. Petrone, D. A. Yoon, H. Weinstabl, H. & Lautens, M. Additive Effects in the Palladium-Catalyzed Carboiodination of Chiral N-Allyl Carboxamides. *Angew. Chem. Int. Ed.* **53**, 7908-7912 (2014).

2. Chi, X. Meng, L. Pang, Q. Guo, L. Liu, Q. Zhao, P. Zhang, D. Sun, F. Li, X. & Liu, H. Palladium-Catalyzed Domino Process to Construct 2,3,9,9a-Tetrahydro-1H-Fluorene Derivatives: Transient σ-Alkyl palladium(II) Complex Mediated C(sp^2)-H Bond Activation. *Asian J. Org. Chem.* **8**, 2201-2204 (2019).

3. Chen, C. Hou, L. Cheng, M. Su, J. & Tong, X. Palladium(0)-Catalyzed Iminohalogenation of Alkenes: Synthesis of 2-Halomethyl Dihydropyrroles and Mechanistic Insights into the Alkyl Halide Bond Formation. *Angew. Chem. Int. Ed.* **54**, 3092-3096 (2015).

4. Lin, Q. & Diao, T. Mechanism of Ni-Catalyzed Reductive 1,2-Dicarbofunctionalization of Alkenes. *J. Am. Chem. Soc.* **141**, 17937-17948 (2019).

6. Gaussian 09, Revision E.01, Frisch, M. J. Trucks, G. W. Schlegel, H. B. Scuseria, G. E. Robb, M. A. Cheeseman, J. R. Scalmani, G. Barone, V. Mennucci, B. Petersson, G. A. Nakatsuji, H. Caricato, M. Li, X. Hratchian, H. P. Izmaylov, A. F. Bloino, J. Zheng, G. Sonnenberg, J. L. Hada, M. Ehara, M. Toyota, K. Fukuda, R. Hasegawa, J. Ishida, M. Nakajima, T. Honda, Y. Kitao, O. Nakai, H. Vreven, T. Montgomery, J. A. Peralta, Jr., J. E. Ogliaro, F. Bearpark, M. Heyd, J. J. Brothers, E. Kudin, K. N. Staroverov, V. N. Keith, T. Kobayashi, R. Normand, J. Raghavachari, K. Rendell, A. Burant, J. C. Iyengar, S. S. Tomasi, J. Cossi, M. Rega, N. Millam, J. M. Klene, M. Knox, J. E. Cross, J. B. Bakken, V. Adamo, C. Jaramillo, J. Gomperts, R. Stratmann, R. E. Yazyev, O. Austin, A. J. Cammi, R. Pomelli, C. Ochterski, J. W. Martin, R. L. Morokuma, K. Zakrzewski, V. G. Voth, G. A. Salvador, P. Dannenberg, J. J. Dapprich, S. Daniels, A. D. Farkas, O. Foresman, J. B. Ortiz, J. V. Cioslowski, J. & Fox, D. J. Gaussian, Inc., Wallingford CT, **2013**.

7. Becke, A. D. Density-functional thermochemistry. III. The role of exact exchange. *J. Chem. Phys.* **98**, 5648-5652 (1993).

8. Lee, C. Yang, W. & Parr, R. G. Development of the Colic-Salvetti correlation-energy formula into a functional of the electron density. *Phys. Rev. B*, **37**, 785-789 (1988).

9. Grimme, S. Antony, J. Ehrlich, S. & Krieg, H. A consistent and accurate ab initio parametrization of density functional dispersion correction (DFT-D) for the 94 elements H-Pu. *J. Chem. Phys*. **132**, 154104-154119 (2010).

10. Roy, L. E. Hay, P. J. & Martin, R. L. Revised Basis Sets for the LANL Effective Core Potentials. *J. Chem. Theory Comput*. **4**, 1029-1031 (2008).

11. Hay, P. J. & Wadt, W. R. Ab initio effective core potentials for molecular calculations. Potentials for K to Au including the outermost core orbitals. *J. Chem. Phys*. **82**, 299-310 (1985).

12. Pritchard, B. P. Altarawy, D. Didier, B. Gibsom, T. D. & Windus, T. L. New Basis Set Exchange: An Open, Up-to-Date Resource for the Molecular Sciences Community. *J. Chem. Inf. Model.* **59**, 4814-4820 (2019).

13. Feller, D. The Role of Databases in Support of Computational Chemistry Calculations. *J. Comput. Chem.* **17**, 1571-1586 (1996).

14. Schuchardt, K. L. Didier, B. T. Elsethagen, T. Sun, L. Gurumoorthi, V. Chase, J. Li, J. & Windus, T. L. *J. Chem. Inf. Model.* **47**, 1045 2007 (2007).

15. Ditchfield, R. Hehre, W. J. & Pople, J. A. Self-Consistent Molecular-Orbital Methods. IX. An Extended Gaussian-Type Basis for Molecular-Orbital Studies of Organic Molecules. *J. Chem. Phys.* **54**, 724-728 (1971).

16. Hehre, W. J. Ditchfield, R. & Pople, J. A. Self-Consistent Molecular Orbital Methods. XII. Further Extensions of Gaussian-Type Basis Sets for Use in Molecular Orbital Studies of Organic Molecules. *J. Chem. Phys.* **56**, 2257-2261 (1972).

17. Hariharan, P. C. & Pople, J. A. The influence of polarization functions on molecular orbital hydrogenation energies. *Theor. Chem. Acc.* **28**, 213-222 (1973).

18. Zhao, Y. & Truhlar, D. G. The M06 suite of density functionals for main group thermochemistry, thermochemical kinetics, noncovalent interactions, excited states, and transition elements: two new functionals and systematic testing of four M06-class functionals and 12 other functionals. *Theor. Chem. Acc.* **120**, 215-241 (2008).

19. Dolg, M. Wedig, U. Stoll, H. & Preuss, H. Energy-adjusted ab initio pseudopotentials for the first row transition elements. *J. Chem. Phys.* **86**, 866-872 (1987).

20. Nicklass, A. Dolg, M. Stoll, H. & Preuss, H. Ab initio energy-adjusted pseudopotentials for the noble gases Ne through Xe: Calculation of atomic dipole and quadrupole polarizabilities. *J. Chem.*

Phys. **102**, 8942-8952 (1995).

21. Clark, T. Chandrasekhar, J. Spitznagel, G. W. & Schleyer, P. Von R. Efficient diffuse function-augmented basis sets for anion calculations. III.[‡] The 3-21+G basis set for first-row elements, Li–F. *J. Comput. Chem*. **4**, 294-301 (1983).

22. Krishnan, R. Binkley, J. S. Seeger, R. & Pople, J. A. Self-consistent molecular orbital methods. XX. A basis set for correlated wave functions. *J. Chem. Phys.* **72**, 650-654 (1980).

23. Marenich, A. V. Cramer, C. J. & Truhlar, D. G. Universal Solvation Model Based on Solute Electron Density and on a Continuum Model of the Solvent Defined by the Bulk Dielectric Constant and Atomic Surface Tensions. *J. Phys. Chem. B*. **113**, 6378-6396 (2009).

24. Johnson, E. R. Keinan, S. Mori-Sánchez, P. Contreras-Garcia, J. Cohen, A. J. & Yang, W. Revealing Noncovalent Interactions. *J. Am. Chem. Soc.* **132**, 6498-6506 (2010).

25. Lu, T. & Chen, F. Multiwfn: A Multifunctional Wavefunction Analyzer. *J. Comput. Chem.* **33**, 580-592 (2012).

26. Humphrey, W. Dalke, A. & Schulten, VMD: Visual molecular dynamics. *J. Mol. Graph.* **14**, 33-38 (1996).

27. Legault, C. Y. CYL View, version 1.0 b; Universite de Sherbrooke, Sherbrooke, Quebec, Canada, **2009**; http://www.cylview.org.

28. Wang, X., Liu, Y., Martin, R. Ni-Catalyzed Divergent Cyclization/Carboxylation of Unactivated Primary and Secondary Alkyl Halides with CO_2. *J. Am. Chem. Soc.* **137**, 6476-6479 (2015).

29. Börjesson, M., Moragas, T., Martin, R. Ni-Catalyzed Carboxylation of Unactivated Alkyl Chlorides with CO_2. *J. Am. Chem. Soc.* **138**, 7504-7507 (2016).

30. Ryan Barber, E., Hynds, H. M., Stephens, C. P., Lemons, H. E., Fredrickson, E. T., Wilger, D. J. Nickel-Catalyzed Hydroarylation of Alkynes under ReductiveConditions with Aryl Bromides and Water. *J. Org. Chem.* **84**, 11612−1162 (2019).

31. Choi, H., Lyu, X., Kim, D., Seo, S., Chang, S. Endo-Selective Intramolecular Alkyne Hydroamidation Enabled by NiH Catalysis Incorporating Alkenylnickel Isomerization. *J. Am. Chem. Soc.* **144**, 10064-10074 (2022).

32. Li, J., Tang, D., Zhang, Y., Chen, W., Su, X., Yu, P., Qu, S. DFT study of Ni-catalyzed intramolecular asymmetric anti-hydrometalative cyclization of alkynone: mechanism and origins of selectivity. Org. Chem. Front. 10, 4263-4274 (2023).

33. Shan, C., He, M., Luo, X., Lia, R. & Zhang, T. Mechanistic insight into anti-carbometalation of an alkyne via η2 -vinyl-nickel type Z/E isomerization. *Org. Chem. Front.* **10**, 4243-4249 (2023).
