# Supplementary material for: Nickel-catalyzed switchable arylative/endo-cyclization of 1,6-enynes
Source: Nat Commun. 2024 Apr 4;15:2914. doi: 10.1038/s41467-024-47200-z (PMC10995176; doi:10.1038/s41467-024-47200-z)
Supplement: Supplementary file 3 — Description of Additional Supplementary Files [file 41467_2024_47200_MOESM3_ESM.docx]

**Description of Additional Supplementary Files**

**File Name: Supplementary Data 1**

**Description:** Supplementary Data for X-RAY.
