# Supplementary material for: Nickel-catalyzed switchable arylative/endo-cyclization of 1,6-enynes
Source: Nat Commun. 2024 Apr 4;15:2914. doi: 10.1038/s41467-024-47200-z (PMC10995176; doi:10.1038/s41467-024-47200-z)
Supplement: Supplementary file 4 — Supplementary Data 1 [file 41467_2024_47200_MOESM4_ESM.docx]

**X-Ray Crystallographic Data**

**X-Ray Crystallographic Analysis of Complex 7**

**(CCDC number: 2220556)**


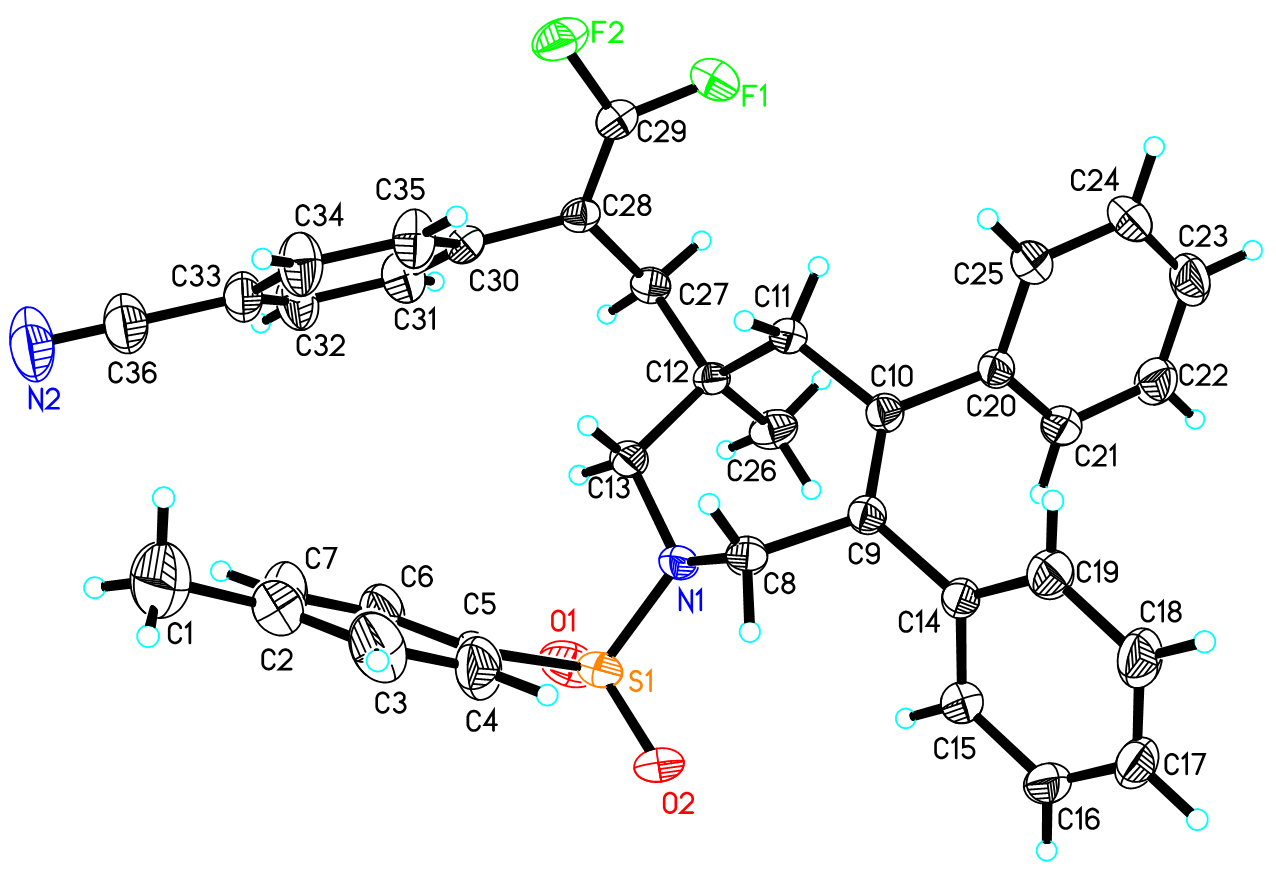


**Supplementary Table 1**. Crystal data and structure refinement for **7**.

Empirical formula C_36_H_32_F_2_N_2_O_2_S

Formula weight 594.69

Temperature 296(2) K

Wavelength 0.71073 Å

Crystal system Monoclinic

Space group P 1 21/c 1

Unit cell dimensions a = 27.772(7) Å α= 90°.

b = 6.2689(16) Å β= 100.470(7)°.

c = 18.093(5) Å γ = 90°.

Volume 3097.6(14) Å3

Z 4

Density (calculated) 1.275 Mg/m3

Absorption coefficient 0.151 mm-1

F(000) 1248

Crystal size 0.12 x 0.1 x 0.1 mm3

Theta range for data collection 0.746 to 24.994°.

Index ranges -33<=h<=32, -7<=k<=7, -20<=l<=21

Reflections collected 25900

Independent reflections 5458 [R(int) = 0.0601]

Completeness to theta = 24.994° 100.0 %

Absorption correction Semi-empirical from equivalents

Max. and min. transmission 0.7452 and 0.6988

Refinement method Full-matrix least-squares on F2

Data / restraints / parameters 5458 / 0 / 390

Goodness-of-fit on F2 1.022

Final R indices [I>2sigma(I)] R1 = 0.0510, wR2 = 0.0930

R indices (all data) R1 = 0.1052, wR2 = 0.1098

Extinction coefficient n/a

Largest diff. peak and hole 0.146 and -0.225 e.Å-3

**Supplementary Table 2**. Atomic coordinates (x104) and equivalent isotropic displacement parameters (Å2x103) for **7**. U(eq) is defined as one third of the trace of the orthogonalized Uij tensor.

______________________________________________________________________

x y z U(eq)

___________________________________________________________________________

C(1) 345(1) 2641(7) 565(3) 146(2)

C(2) 774(1) 4207(7) 689(2) 86(1)

C(3) 1164(1) 3949(6) 340(2) 92(1)

C(4) 1555(1) 5328(6) 466(2) 77(1)

C(5) 1564(1) 7024(5) 949(1) 52(1)

C(6) 1173(1) 7318(6) 1302(2) 80(1)

C(7) 782(1) 5914(8) 1162(2) 99(1)

C(8) 2766(1) 5599(4) 1365(1) 45(1)

C(9) 3315(1) 5546(4) 1669(1) 38(1)

C(10) 3486(1) 5132(4) 2392(1) 37(1)

C(11) 3139(1) 4736(4) 2940(1) 39(1)

C(12) 2812(1) 6625(3) 3066(1) 33(1)

C(13) 2371(1) 6772(4) 2426(1) 41(1)

C(14) 3632(1) 5938(4) 1099(1) 41(1)

C(15) 3597(1) 7809(5) 688(1) 54(1)

C(16) 3882(1) 8161(6) 145(2) 66(1)

C(17) 4200(1) 6618(7) -1(2) 73(1)

C(18) 4239(1) 4749(6) 392(2) 70(1)

C(19) 3959(1) 4403(5) 942(2) 57(1)

C(20) 4017(1) 5134(4) 2747(1) 38(1)

C(21) 4318(1) 6865(4) 2694(1) 48(1)

C(22) 4799(1) 6898(5) 3073(2) 59(1)

C(23) 4984(1) 5188(6) 3503(2) 65(1)

C(24) 4693(1) 3441(5) 3557(2) 64(1)

C(25) 4214(1) 3421(4) 3189(1) 52(1)

C(26) 3095(1) 8725(4) 3138(1) 52(1)

C(27) 2621(1) 6309(4) 3807(1) 38(1)

C(28) 2336(1) 4303(4) 3888(1) 34(1)

C(29) 2555(1) 2727(4) 4279(1) 44(1)

C(30) 1798(1) 4201(4) 3611(1) 37(1)

C(31) 1500(1) 5842(4) 3777(2) 56(1)

C(32) 1002(1) 5769(5) 3544(2) 66(1)

C(33) 789(1) 4051(5) 3142(2) 57(1)

C(34) 1076(1) 2413(5) 2969(2) 68(1)

C(35) 1577(1) 2508(4) 3199(2) 58(1)

C(36) 264(1) 3985(5) 2907(2) 77(1)

F(1) 3024(1) 2648(2) 4604(1) 64(1)

F(2) 2347(1) 928(2) 4455(1) 66(1)

N(1) 2505(1) 7314(3) 1698(1) 42(1)

N(2) -151(1) 3939(5) 2730(2) 107(1)

O(1) 1970(1) 10478(3) 1554(1) 76(1)

O(2) 2269(1) 8978(3) 453(1) 78(1)

S(1) 2087(1) 8671(1) 1136(1) 57(1)

___________________________________________________________________________ **Supplementary Table 3**. Bond lengths [Å] and angles [°] for **7**.

_____________________________________________________

C(1)-H(1A) 0.9600

C(1)-H(1B) 0.9600

C(1)-H(1C) 0.9600

C(1)-C(2) 1.528(5)

C(2)-C(3) 1.359(5)

C(2)-C(7) 1.367(5)

C(3)-H(3) 0.9300

C(3)-C(4) 1.376(4)

C(4)-H(4) 0.9300

C(4)-C(5) 1.373(4)

C(5)-C(6) 1.369(3)

C(5)-S(1) 1.764(3)

C(6)-H(6) 0.9300

C(6)-C(7) 1.385(5)

C(7)-H(7) 0.9300

C(8)-H(8A) 0.9700

C(8)-H(8B) 0.9700

C(8)-C(9) 1.523(3)

C(8)-N(1) 1.484(3)

C(9)-C(10) 1.334(3)

C(9)-C(14) 1.494(3)

C(10)-C(11) 1.524(3)

C(10)-C(20) 1.498(3)

C(11)-H(11A) 0.9700

C(11)-H(11B) 0.9700

C(11)-C(12) 1.534(3)

C(12)-C(13) 1.529(3)

C(12)-C(26) 1.526(3)

C(12)-C(27) 1.541(3)

C(13)-H(13A) 0.9700

C(13)-H(13B) 0.9700

C(13)-N(1) 1.472(3)

C(14)-C(15) 1.383(3)

C(14)-C(19) 1.386(3)

C(15)-H(15) 0.9300

C(15)-C(16) 1.386(3)

C(16)-H(16) 0.9300

C(16)-C(17) 1.367(4)

C(17)-H(17) 0.9300

C(17)-C(18) 1.364(4)

C(18)-H(18) 0.9300

C(18)-C(19) 1.388(4)

C(19)-H(19) 0.9300

C(20)-C(21) 1.384(3)

C(20)-C(25) 1.390(3)

C(21)-H(21) 0.9300

C(21)-C(22) 1.385(3)

C(22)-H(22) 0.9300

C(22)-C(23) 1.369(4)

C(23)-H(23) 0.9300

C(23)-C(24) 1.374(4)

C(24)-H(24) 0.9300

C(24)-C(25) 1.374(3)

C(25)-H(25) 0.9300

C(26)-H(26A) 0.9600

C(26)-H(26B) 0.9600

C(26)-H(26C) 0.9600

C(27)-H(27A) 0.9700

C(27)-H(27B) 0.9700

C(27)-C(28) 1.508(3)

C(28)-C(29) 1.301(3)

C(28)-C(30) 1.487(3)

C(29)-F(1) 1.328(3)

C(29)-F(2) 1.332(3)

C(30)-C(31) 1.387(3)

C(30)-C(35) 1.376(3)

C(31)-H(31) 0.9300

C(31)-C(32) 1.372(3)

C(32)-H(32) 0.9300

C(32)-C(33) 1.373(4)

C(33)-C(34) 1.371(4)

C(33)-C(36) 1.441(4)

C(34)-H(34) 0.9300

C(34)-C(35) 1.379(3)

C(35)-H(35) 0.9300

C(36)-N(2) 1.140(3)

N(1)-S(1) 1.6369(19)

O(1)-S(1) 1.432(2)

O(2)-S(1) 1.4303(18)

H(1A)-C(1)-H(1B) 109.5

H(1A)-C(1)-H(1C) 109.5

H(1B)-C(1)-H(1C) 109.5

C(2)-C(1)-H(1A) 109.5

C(2)-C(1)-H(1B) 109.5

C(2)-C(1)-H(1C) 109.5

C(3)-C(2)-C(1) 121.4(4)

C(3)-C(2)-C(7) 117.4(4)

C(7)-C(2)-C(1) 121.2(4)

C(2)-C(3)-H(3) 119.3

C(2)-C(3)-C(4) 121.4(4)

C(4)-C(3)-H(3) 119.3

C(3)-C(4)-H(4) 119.6

C(5)-C(4)-C(3) 120.9(3)

C(5)-C(4)-H(4) 119.6

C(4)-C(5)-S(1) 119.6(2)

C(6)-C(5)-C(4) 118.5(3)

C(6)-C(5)-S(1) 121.8(3)

C(5)-C(6)-H(6) 120.3

C(5)-C(6)-C(7) 119.4(3)

C(7)-C(6)-H(6) 120.3

C(2)-C(7)-C(6) 122.4(3)

C(2)-C(7)-H(7) 118.8

C(6)-C(7)-H(7) 118.8

H(8A)-C(8)-H(8B) 107.7

C(9)-C(8)-H(8A) 108.9

C(9)-C(8)-H(8B) 108.9

N(1)-C(8)-H(8A) 108.9

N(1)-C(8)-H(8B) 108.9

N(1)-C(8)-C(9) 113.55(19)

C(10)-C(9)-C(8) 120.9(2)

C(10)-C(9)-C(14) 123.9(2)

C(14)-C(9)-C(8) 115.14(19)

C(9)-C(10)-C(11) 121.0(2)

C(9)-C(10)-C(20) 124.7(2)

C(20)-C(10)-C(11) 114.24(19)

C(10)-C(11)-H(11A) 108.4

C(10)-C(11)-H(11B) 108.4

C(10)-C(11)-C(12) 115.62(19)

H(11A)-C(11)-H(11B) 107.4

C(12)-C(11)-H(11A) 108.4

C(12)-C(11)-H(11B) 108.4

C(11)-C(12)-C(27) 109.65(18)

C(13)-C(12)-C(11) 110.40(18)

C(13)-C(12)-C(27) 108.13(18)

C(26)-C(12)-C(11) 111.68(19)

C(26)-C(12)-C(13) 110.02(19)

C(26)-C(12)-C(27) 106.84(18)

C(12)-C(13)-H(13A) 109.0

C(12)-C(13)-H(13B) 109.0

H(13A)-C(13)-H(13B) 107.8

N(1)-C(13)-C(12) 113.14(18)

N(1)-C(13)-H(13A) 109.0

N(1)-C(13)-H(13B) 109.0

C(15)-C(14)-C(9) 121.1(2)

C(15)-C(14)-C(19) 117.7(2)

C(19)-C(14)-C(9) 121.1(2)

C(14)-C(15)-H(15) 119.3

C(14)-C(15)-C(16) 121.4(3)

C(16)-C(15)-H(15) 119.3

C(15)-C(16)-H(16) 120.1

C(17)-C(16)-C(15) 119.7(3)

C(17)-C(16)-H(16) 120.1

C(16)-C(17)-H(17) 120.0

C(18)-C(17)-C(16) 120.1(3)

C(18)-C(17)-H(17) 120.0

C(17)-C(18)-H(18) 119.8

C(17)-C(18)-C(19) 120.3(3)

C(19)-C(18)-H(18) 119.8

C(14)-C(19)-C(18) 120.7(3)

C(14)-C(19)-H(19) 119.6

C(18)-C(19)-H(19) 119.6

C(21)-C(20)-C(10) 122.0(2)

C(21)-C(20)-C(25) 117.7(2)

C(25)-C(20)-C(10) 120.1(2)

C(20)-C(21)-H(21) 119.4

C(20)-C(21)-C(22) 121.1(2)

C(22)-C(21)-H(21) 119.4

C(21)-C(22)-H(22) 120.1

C(23)-C(22)-C(21) 119.9(3)

C(23)-C(22)-H(22) 120.1

C(22)-C(23)-H(23) 120.0

C(22)-C(23)-C(24) 120.0(3)

C(24)-C(23)-H(23) 120.0

C(23)-C(24)-H(24) 119.9

C(25)-C(24)-C(23) 120.1(3)

C(25)-C(24)-H(24) 119.9

C(20)-C(25)-H(25) 119.4

C(24)-C(25)-C(20) 121.1(3)

C(24)-C(25)-H(25) 119.4

C(12)-C(26)-H(26A) 109.5

C(12)-C(26)-H(26B) 109.5

C(12)-C(26)-H(26C) 109.5

H(26A)-C(26)-H(26B) 109.5

H(26A)-C(26)-H(26C) 109.5

H(26B)-C(26)-H(26C) 109.5

C(12)-C(27)-H(27A) 107.9

C(12)-C(27)-H(27B) 107.9

H(27A)-C(27)-H(27B) 107.2

C(28)-C(27)-C(12) 117.58(18)

C(28)-C(27)-H(27A) 107.9

C(28)-C(27)-H(27B) 107.9

C(29)-C(28)-C(27) 119.0(2)

C(29)-C(28)-C(30) 119.6(2)

C(30)-C(28)-C(27) 121.1(2)

C(28)-C(29)-F(1) 126.4(2)

C(28)-C(29)-F(2) 126.3(2)

F(1)-C(29)-F(2) 107.2(2)

C(31)-C(30)-C(28) 119.8(2)

C(35)-C(30)-C(28) 122.6(2)

C(35)-C(30)-C(31) 117.6(2)

C(30)-C(31)-H(31) 119.4

C(32)-C(31)-C(30) 121.1(3)

C(32)-C(31)-H(31) 119.4

C(31)-C(32)-H(32) 119.9

C(31)-C(32)-C(33) 120.2(3)

C(33)-C(32)-H(32) 119.9

C(32)-C(33)-C(36) 119.6(3)

C(34)-C(33)-C(32) 119.7(3)

C(34)-C(33)-C(36) 120.7(3)

C(33)-C(34)-H(34) 120.1

C(33)-C(34)-C(35) 119.8(3)

C(35)-C(34)-H(34) 120.1

C(30)-C(35)-C(34) 121.6(3)

C(30)-C(35)-H(35) 119.2

C(34)-C(35)-H(35) 119.2

N(2)-C(36)-C(33) 179.3(4)

C(8)-N(1)-S(1) 117.94(15)

C(13)-N(1)-C(8) 114.67(18)

C(13)-N(1)-S(1) 114.12(15)

N(1)-S(1)-C(5) 106.39(11)

O(1)-S(1)-C(5) 107.85(13)

O(1)-S(1)-N(1) 106.66(11)

O(2)-S(1)-C(5) 108.73(13)

O(2)-S(1)-N(1) 106.49(11)

O(2)-S(1)-O(1) 119.96(13)

_____________________________________________________________

Symmetry transformations used to generate equivalent atoms:

**Supplementary Table 4**. Anisotropic displacement parameters (Å2x 103) for **7**. The anisotropic displacement factor exponent takes the form: -2π2[ h2 a*2U11 + ... + 2 h k a* b* U12 ]

___________________________________________________________________________

U11 U22 U33 U23 U13 U12

___________________________________________________________________________

C(1) 89(3) 165(4) 171(5) 49(4) -11(3) -50(3)

C(2) 57(2) 113(3) 86(3) 28(2) 1(2) -1(2)

C(3) 67(2) 111(3) 90(3) -15(2) -2(2) 6(2)

C(4) 48(2) 113(3) 72(2) -10(2) 17(2) 9(2)

C(5) 35(2) 83(2) 37(2) 12(2) 3(1) 19(2)

C(6) 50(2) 124(3) 68(2) -12(2) 18(2) 17(2)

C(7) 49(2) 164(4) 90(3) 7(3) 26(2) 7(3)

C(8) 41(2) 64(2) 28(1) 1(1) 5(1) -2(1)

C(9) 35(1) 44(2) 34(1) 0(1) 5(1) 1(1)

C(10) 35(1) 36(1) 41(2) 2(1) 8(1) 1(1)

C(11) 35(1) 44(2) 36(1) 7(1) 4(1) -1(1)

C(12) 36(1) 31(1) 31(1) 4(1) 5(1) -1(1)

C(13) 38(1) 52(2) 35(1) 6(1) 10(1) 9(1)

C(14) 33(1) 60(2) 30(1) -1(1) 4(1) -4(1)

C(15) 45(2) 71(2) 46(2) 11(2) 9(1) 2(2)

C(16) 50(2) 101(3) 44(2) 21(2) 4(2) -11(2)

C(17) 43(2) 135(3) 42(2) 4(2) 12(1) -11(2)

C(18) 52(2) 107(3) 57(2) -11(2) 22(2) 8(2)

C(19) 52(2) 71(2) 49(2) -1(2) 14(1) 6(2)

C(20) 34(1) 47(2) 34(1) 1(1) 7(1) 2(1)

C(21) 42(2) 59(2) 42(2) 6(1) 5(1) -2(1)

C(22) 44(2) 78(2) 52(2) 1(2) 5(1) -14(2)

C(23) 37(2) 102(3) 54(2) -1(2) -2(1) 4(2)

C(24) 48(2) 80(2) 62(2) 17(2) 2(2) 18(2)

C(25) 42(2) 55(2) 59(2) 11(2) 7(1) 5(1)

C(26) 66(2) 42(2) 49(2) 2(1) 12(1) -9(1)

C(27) 44(1) 39(2) 32(1) -2(1) 5(1) 0(1)

C(28) 42(2) 36(2) 27(1) -1(1) 11(1) 2(1)

C(29) 48(2) 42(2) 43(2) 3(1) 14(1) -3(1)

C(30) 41(2) 37(2) 36(1) -1(1) 13(1) -3(1)

C(31) 51(2) 52(2) 63(2) -14(1) 7(2) 3(2)

C(32) 47(2) 63(2) 87(2) -6(2) 12(2) 12(2)

C(33) 38(2) 58(2) 75(2) 4(2) 9(2) -2(2)

C(34) 50(2) 63(2) 90(2) -20(2) 8(2) -12(2)

C(35) 45(2) 49(2) 81(2) -18(2) 15(2) -3(2)

C(36) 48(2) 66(2) 113(3) 5(2) 7(2) 0(2)

F(1) 58(1) 71(1) 58(1) 17(1) 2(1) 11(1)

F(2) 84(1) 47(1) 72(1) 16(1) 30(1) -2(1)

N(1) 39(1) 58(1) 30(1) 9(1) 6(1) 10(1)

N(2) 46(2) 86(2) 182(3) 7(2) 2(2) 0(2)

O(1) 92(2) 58(1) 76(1) 12(1) 4(1) 25(1)

O(2) 64(1) 123(2) 48(1) 43(1) 13(1) 5(1)

S(1) 52(1) 74(1) 45(1) 23(1) 5(1) 11(1)

___________________________________________________________________________**Supplementary Table 5.**Hydrogen coordinates ( x 104) and isotropic displacement parameters (Å2x 103) for **7**.

___________________________________________________________________________

x y z U(eq)

H(1A) 289 2150 54 219

H(1B) 56 3344 665 219

H(1C) 420 1448 898 219

H(3) 1166 2816 9 110

H(4) 1818 5107 222 92

H(6) 1170 8452 1632 96

H(7) 515 6140 1398 119

H(8A) 2714 5793 825 54

H(8B) 2626 4233 1463 54

H(11A) 3332 4329 3421 46

H(11B) 2930 3537 2758 46

H(13A) 2200 5416 2379 50

H(13B) 2147 7847 2550 50

H(15) 3377 8855 778 65

H(16) 3856 9441 -119 79

H(17) 4389 6844 -368 87

H(18) 4455 3701 289 84

H(19) 3991 3128 1209 68

H(21) 4196 8026 2398 58

H(22) 4995 8079 3035 71

H(23) 5306 5208 3760 79

H(24) 4821 2270 3842 77

H(25) 4019 2242 3237 63

H(26A) 3369 8626 3546 78

H(26B) 2883 9862 3235 78

H(26C) 3211 9008 2680 78

H(27A) 2900 6348 4216 46

H(27B) 2415 7518 3871 46

H(31) 1640 7012 4051 67

H(32) 808 6887 3659 79

H(34) 934 1243 2698 82

H(35) 1770 1400 3073 69

___________________________________________________________________________ **Supplementary Table 6**. Torsion angles [°] for **7**.

________________________________________________________________

C(1)-C(2)-C(3)-C(4) -178.4(3)

C(1)-C(2)-C(7)-C(6) 178.0(3)

C(2)-C(3)-C(4)-C(5) -0.3(5)

C(3)-C(2)-C(7)-C(6) -1.4(6)

C(3)-C(4)-C(5)-C(6) -0.1(5)

C(3)-C(4)-C(5)-S(1) 176.5(2)

C(4)-C(5)-C(6)-C(7) -0.2(4)

C(4)-C(5)-S(1)-N(1) -74.8(2)

C(4)-C(5)-S(1)-O(1) 171.1(2)

C(4)-C(5)-S(1)-O(2) 39.5(3)

C(5)-C(6)-C(7)-C(2) 1.0(5)

C(6)-C(5)-S(1)-N(1) 101.7(2)

C(6)-C(5)-S(1)-O(1) -12.4(3)

C(6)-C(5)-S(1)-O(2) -143.9(2)

C(7)-C(2)-C(3)-C(4) 1.1(5)

C(8)-C(9)-C(10)-C(11) -1.0(4)

C(8)-C(9)-C(10)-C(20) -177.4(2)

C(8)-C(9)-C(14)-C(15) 58.8(3)

C(8)-C(9)-C(14)-C(19) -118.6(3)

C(8)-N(1)-S(1)-C(5) 77.13(19)

C(8)-N(1)-S(1)-O(1) -167.94(17)

C(8)-N(1)-S(1)-O(2) -38.7(2)

C(9)-C(8)-N(1)-C(13) -83.5(2)

C(9)-C(8)-N(1)-S(1) 137.72(17)

C(9)-C(10)-C(11)-C(12) -63.7(3)

C(9)-C(10)-C(20)-C(21) 53.9(3)

C(9)-C(10)-C(20)-C(25) -130.5(3)

C(9)-C(14)-C(15)-C(16) -178.4(2)

C(9)-C(14)-C(19)-C(18) 177.5(2)

C(10)-C(9)-C(14)-C(15) -122.5(3)

C(10)-C(9)-C(14)-C(19) 60.1(3)

C(10)-C(11)-C(12)-C(13) 80.6(2)

C(10)-C(11)-C(12)-C(26) -42.2(3)

C(10)-C(11)-C(12)-C(27) -160.40(19)

C(10)-C(20)-C(21)-C(22) 175.1(2)

C(10)-C(20)-C(25)-C(24) -176.2(2)

C(11)-C(10)-C(20)-C(21) -122.7(2)

C(11)-C(10)-C(20)-C(25) 52.9(3)

C(11)-C(12)-C(13)-N(1) -66.4(2)

C(11)-C(12)-C(27)-C(28) -57.5(3)

C(12)-C(13)-N(1)-C(8) 70.2(3)

C(12)-C(13)-N(1)-S(1) -149.45(17)

C(12)-C(27)-C(28)-C(29) 100.8(3)

C(12)-C(27)-C(28)-C(30) -86.7(3)

C(13)-C(12)-C(27)-C(28) 63.0(3)

C(13)-N(1)-S(1)-C(5) -61.9(2)

C(13)-N(1)-S(1)-O(1) 53.0(2)

C(13)-N(1)-S(1)-O(2) -177.76(18)

C(14)-C(9)-C(10)-C(11) -179.7(2)

C(14)-C(9)-C(10)-C(20) 4.0(4)

C(14)-C(15)-C(16)-C(17) 1.2(4)

C(15)-C(14)-C(19)-C(18) 0.1(4)

C(15)-C(16)-C(17)-C(18) -0.6(4)

C(16)-C(17)-C(18)-C(19) -0.3(5)

C(17)-C(18)-C(19)-C(14) 0.5(4)

C(19)-C(14)-C(15)-C(16) -0.9(4)

C(20)-C(10)-C(11)-C(12) 113.0(2)

C(20)-C(21)-C(22)-C(23) 0.6(4)

C(21)-C(20)-C(25)-C(24) -0.4(4)

C(21)-C(22)-C(23)-C(24) 0.2(4)

C(22)-C(23)-C(24)-C(25) -1.1(4)

C(23)-C(24)-C(25)-C(20) 1.2(4)

C(25)-C(20)-C(21)-C(22) -0.5(4)

C(26)-C(12)-C(13)-N(1) 57.3(3)

C(26)-C(12)-C(27)-C(28) -178.7(2)

C(27)-C(12)-C(13)-N(1) 173.62(18)

C(27)-C(28)-C(29)-F(1) -2.7(4)

C(27)-C(28)-C(29)-F(2) 174.2(2)

C(27)-C(28)-C(30)-C(31) -46.9(3)

C(27)-C(28)-C(30)-C(35) 134.3(2)

C(28)-C(30)-C(31)-C(32) -178.4(2)

C(28)-C(30)-C(35)-C(34) 177.8(2)

C(29)-C(28)-C(30)-C(31) 125.6(3)

C(29)-C(28)-C(30)-C(35) -53.2(3)

C(30)-C(28)-C(29)-F(1) -175.3(2)

C(30)-C(28)-C(29)-F(2) 1.5(4)

C(30)-C(31)-C(32)-C(33) 0.3(4)

C(31)-C(30)-C(35)-C(34) -1.1(4)

C(31)-C(32)-C(33)-C(34) -0.4(5)

C(31)-C(32)-C(33)-C(36) 179.3(3)

C(32)-C(33)-C(34)-C(35) -0.2(5)

C(33)-C(34)-C(35)-C(30) 1.0(4)

C(35)-C(30)-C(31)-C(32) 0.5(4)

C(36)-C(33)-C(34)-C(35) -180.0(3)

N(1)-C(8)-C(9)-C(10) 63.7(3)

N(1)-C(8)-C(9)-C(14) -117.6(2)

S(1)-C(5)-C(6)-C(7) -176.8(2)

________________________________________________________________

Symmetry transformations used to generate equivalent atoms:

**Supplementary Table 7.** Hydrogen bonds for **7** [Å and °].

___________________________________________________________________________

D-H...A d(D-H) d(H...A) d(D...A) <(DHA)

___________________________________________________________________________

**X-Ray Crystallographic Analysis of Complex 47**

**(CCDC number: 2220555)**


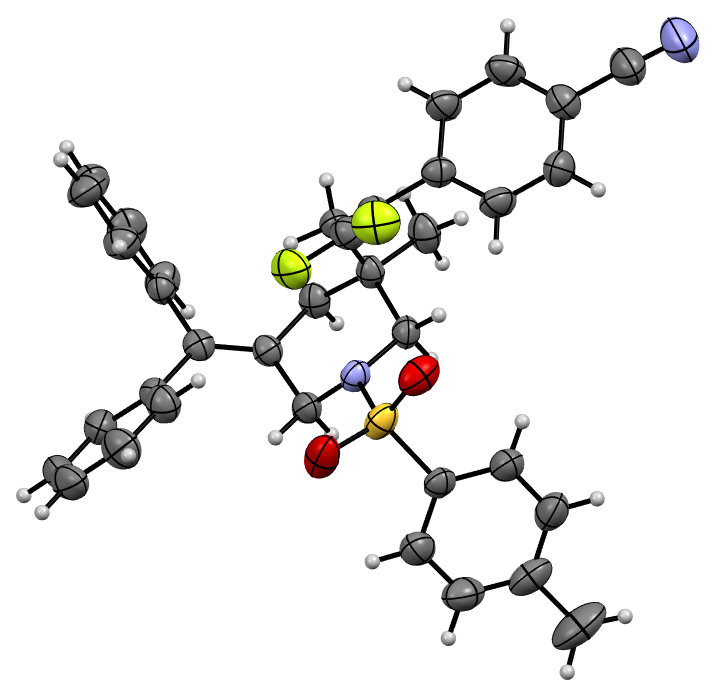


**Supplementary Table 8**. Crystal data and structure refinement for **47.**

| Empirical formula | C_36_H_32_N_2_O_4_ClSF |
| --- | --- |
| Formula weight | 592.08 |
| Temperature/K | 296(2) |
| Crystal system | triclinic |
| Space group | P-1 |
| a/Å | 10.348(6) |
| b/Å | 11.311(7) |
| c/Å | 14.602(9) |
| α/° | 82.493(6) |
| β/° | 79.028(6) |
| γ/° | 67.809(6) |
| Volume/Å^3^ | 1550.3(16) |
| Z | 2 |
| ρ_calc_g/cm^3^ | 1.268 |
| μ/mm^‑1^ | 0.165 |
| F(000) | 622.0 |
| Crystal size/mm^3^ | 0.21 × 0.18 × 0.13 |
| Radiation | MoKα (λ = 0.71073) |
| 2Θ range for data collection/° | 3.896 to 55.248 |
| Index ranges | -13 ≤ h ≤ 13, -14 ≤ k ≤ 14, -18 ≤ l ≤ 19 |
| Reflections collected | 12448 |
| Independent reflections | 6960 [R_int_ = 0.0554, R_sigma_ = 0.1088] |
| Data/restraints/parameters | 6960/0/390 |
| Goodness-of-fit on F^2^ | 0.977 |
| Final R indexes [I>=2σ (I)] | R_1_ = 0.0637, wR_2_ = 0.1316 |
| Final R indexes [all data] | R_1_ = 0.1472, wR_2_ = 0.1609 |
| Largest diff. peak/hole / e Å^-3^ | 0.25/-0.28 |

**Supplementary Table 9**. Fractional Atomic Coordinates (×10^4^) and Equivalent Isotropic Displacement Parameters (Å^2^×10^3^) for **47**. U_eq_ is defined as 1/3 of the trace of the orthogonalized U_IJ_ tensor.

| Atom | *x* | *y* | *z* | U(eq) |
| --- | --- | --- | --- | --- |
| S1 | 6178.5(9) | 7279.0(8) | 6828.2(5) | 47.9(2) |
| F1 | 2225(2) | 9206(2) | 8593.7(13) | 70.7(6) |
| O3 | 7211(2) | 6850(2) | 7447.0(15) | 66.2(7) |
| O4 | 5013(2) | 8474(2) | 6956.4(15) | 59.9(6) |
| N1 | 5502(2) | 6169(2) | 6903.7(15) | 39.6(6) |
| N2 | -3123(3) | 11661(4) | 5539(2) | 95.8(12) |
| F2 | 3697.9(19) | 7322(2) | 8899.8(12) | 69.0(6) |
| C1 | 9097(5) | 7274(4) | 2823(3) | 103.1(15) |
| C2 | 8398(4) | 7333(3) | 3829(2) | 61.8(10) |
| C3 | 6971(4) | 8023(3) | 4064(2) | 62.5(10) |
| C4 | 6308(3) | 8037(3) | 4978(2) | 53.7(9) |
| C5 | 7058(3) | 7352(3) | 5674(2) | 41.2(7) |
| C6 | 4365(3) | 6392(3) | 6358.3(19) | 40.0(7) |
| C7 | 3424(3) | 5636(3) | 6817.3(19) | 38.5(7) |
| C8 | 2656(3) | 6088(3) | 7791.0(19) | 42.2(7) |
| C9 | 2098(3) | 7511(3) | 7896.2(19) | 39.9(7) |
| C10 | 946(3) | 8411(3) | 7402(2) | 41.8(7) |
| C11 | 1164(3) | 9392(3) | 6782(2) | 51.1(8) |
| C12 | 117(3) | 10214(3) | 6308(2) | 54.1(9) |
| C13 | -1189(3) | 10091(3) | 6443(2) | 49.3(8) |
| C14 | -2285(4) | 10959(4) | 5945(2) | 65.7(10) |
| C15 | 6464(3) | 4817(3) | 6932(2) | 42.5(7) |
| C16 | 5611(3) | 4022(3) | 7416.5(19) | 38.5(7) |
| C17 | 4372(3) | 4210(3) | 6939(2) | 42.7(7) |
| C18 | -368(3) | 8298(3) | 7523(2) | 50.5(8) |
| C19 | -1431(3) | 9129(3) | 7055(2) | 54.4(9) |
| C20 | 2655(3) | 7983(3) | 8427(2) | 49.4(8) |
| C21 | 2373(3) | 5773(3) | 6156(2) | 53.1(9) |
| C22 | 5917(3) | 3270(3) | 8200.1(19) | 38.3(7) |
| C23 | 5070(3) | 2480(3) | 8655(2) | 37.8(7) |
| C24 | 4975(3) | 1511(3) | 8214(2) | 52.2(8) |
| C25 | 4196(4) | 788(3) | 8655(3) | 66.3(10) |
| C26 | 3492(4) | 1019(4) | 9541(3) | 69.2(11) |
| C27 | 3589(3) | 1947(4) | 9998(2) | 64.3(10) |
| C28 | 4374(3) | 2685(3) | 9567(2) | 50.6(8) |
| C29 | 7102(3) | 3178(3) | 8693(2) | 42.2(7) |
| C30 | 7199(3) | 4245(3) | 9002(2) | 55.1(9) |
| C31 | 8304(4) | 4133(4) | 9456(2) | 68.6(10) |
| C32 | 9307(4) | 2969(5) | 9611(2) | 70.7(11) |
| C33 | 9231(3) | 1894(4) | 9323(2) | 65.7(11) |
| C34 | 8125(3) | 1988(3) | 8872(2) | 53.3(9) |
| C35 | 8499(3) | 6677(3) | 5461(2) | 55.2(9) |
| C36 | 9138(4) | 6685(3) | 4546(3) | 65.6(10) |

**Supplementary Table 10.** Anisotropic Displacement Parameters (Å^2^×10^3^) for **47**. The Anisotropic displacement factor exponent takes the form: -2π^2^[h^2^a*^2^U_11_+2hka*b*U_12_+…].

| Atom | U_11_ | U_22_ | U_33_ | U_23_ | U_13_ | U_12_ |
| --- | --- | --- | --- | --- | --- | --- |
| S1 | 60.9(5) | 55.5(6) | 39.5(5) | 0.3(4) | -5.7(4) | -36.8(5) |
| F1 | 79.4(14) | 71.1(15) | 76.7(14) | -25.3(11) | -10.4(11) | -38.1(12) |
| O3 | 84.4(16) | 86.9(18) | 53.3(14) | 12.8(12) | -27.7(12) | -57.6(14) |
| O4 | 74.5(15) | 45.0(14) | 60.2(15) | -10.2(11) | 7.2(12) | -27.9(13) |
| N1 | 44.7(14) | 40.5(15) | 38.6(14) | 4.1(11) | -7.5(11) | -22.6(12) |
| N2 | 73(2) | 111(3) | 81(3) | 15(2) | -26(2) | -8(2) |
| F2 | 61.9(12) | 98.2(16) | 54.8(12) | -6.8(11) | -21.6(10) | -31.4(12) |
| C1 | 125(4) | 122(4) | 65(3) | -13(3) | 33(3) | -69(3) |
| C2 | 74(3) | 67(2) | 53(2) | -7.1(19) | 11.8(19) | -44(2) |
| C3 | 69(2) | 85(3) | 46(2) | 13.5(18) | -14.3(18) | -44(2) |
| C4 | 48.3(19) | 67(2) | 48(2) | 5.8(17) | -5.9(16) | -27.0(18) |
| C5 | 42.4(17) | 45.9(19) | 41.9(18) | 3.7(14) | -5.8(14) | -25.9(15) |
| C6 | 44.0(17) | 41.6(18) | 34.5(16) | 3.9(13) | -10.8(13) | -15.5(14) |
| C7 | 42.4(17) | 40.0(18) | 39.4(17) | 3.4(13) | -16.7(14) | -18.8(15) |
| C8 | 38.1(16) | 47.0(19) | 43.3(18) | 6.8(14) | -9.4(14) | -19.1(15) |
| C9 | 35.7(16) | 49(2) | 39.1(17) | 0.7(14) | -4.4(13) | -21.1(15) |
| C10 | 41.2(17) | 41.7(18) | 46.7(18) | -4.0(14) | -5.5(14) | -20.1(15) |
| C11 | 43.6(18) | 51(2) | 61(2) | 3.4(17) | -0.7(16) | -25.2(17) |
| C12 | 54(2) | 48(2) | 57(2) | 11.1(16) | -9.1(17) | -19.7(17) |
| C13 | 48.0(19) | 44(2) | 56(2) | -2.3(16) | -14.5(16) | -13.7(16) |
| C14 | 55(2) | 77(3) | 56(2) | 1(2) | -13.9(19) | -13(2) |
| C15 | 40.3(16) | 53(2) | 37.6(17) | 3.1(14) | -10.9(13) | -19.8(16) |
| C16 | 39.0(16) | 39.3(17) | 38.8(17) | -3.4(14) | -9.8(13) | -14.0(14) |
| C17 | 48.6(18) | 40.8(18) | 45.0(18) | 0.0(14) | -15.9(14) | -20.0(15) |
| C18 | 42.6(18) | 47(2) | 66(2) | 6.0(17) | -7.8(16) | -23.3(16) |
| C19 | 41.3(18) | 58(2) | 71(2) | -4.0(18) | -11.6(17) | -24.5(17) |
| C20 | 44.9(19) | 59(2) | 50(2) | -4.7(17) | -9.7(16) | -23.5(17) |
| C21 | 60(2) | 56(2) | 53(2) | 1.2(16) | -27.6(16) | -24.3(17) |
| C22 | 40.2(16) | 34.4(17) | 39.1(17) | -6.7(13) | -5.9(13) | -10.8(14) |
| C23 | 38.3(16) | 34.4(17) | 41.8(17) | 4.4(13) | -12.0(14) | -13.9(14) |
| C24 | 63(2) | 43(2) | 53(2) | -3.3(16) | -7.4(17) | -22.5(17) |
| C25 | 76(2) | 57(2) | 79(3) | 4(2) | -20(2) | -38(2) |
| C26 | 69(2) | 75(3) | 76(3) | 19(2) | -17(2) | -46(2) |
| C27 | 59(2) | 88(3) | 49(2) | 3(2) | 1.7(17) | -38(2) |
| C28 | 49.9(19) | 58(2) | 50(2) | -5.9(16) | -7.8(16) | -25.4(17) |
| C29 | 39.7(17) | 49(2) | 37.3(17) | 6.0(15) | -7.6(14) | -17.8(16) |
| C30 | 57(2) | 59(2) | 54(2) | -0.8(17) | -18.2(17) | -22.0(18) |
| C31 | 67(2) | 89(3) | 62(2) | -3(2) | -25(2) | -36(2) |
| C32 | 49(2) | 116(4) | 50(2) | 5(2) | -17.6(17) | -33(2) |
| C33 | 42(2) | 85(3) | 53(2) | 15(2) | -10.1(17) | -9(2) |
| C34 | 49.5(19) | 57(2) | 48(2) | 7.8(16) | -6.4(16) | -17.4(17) |
| C35 | 48(2) | 54(2) | 64(2) | 7.4(17) | -8.1(17) | -22.6(17) |
| C36 | 51(2) | 60(2) | 76(3) | -4(2) | 9(2) | -18.7(19) |

**Supplementary Table 11.** Bond Lengths for **47**.

| Atom | Atom | Length/Å | Atom | Atom | Length/Å |
| --- | --- | --- | --- | --- | --- |
| S1 | O3 | 1.433(2) | C11 | C12 | 1.369(4) |
| S1 | O4 | 1.437(2) | C12 | C13 | 1.384(4) |
| S1 | N1 | 1.636(2) | C13 | C14 | 1.437(4) |
| S1 | C5 | 1.765(3) | C13 | C19 | 1.383(4) |
| F1 | C20 | 1.324(4) | C15 | C16 | 1.510(4) |
| N1 | C6 | 1.470(3) | C16 | C17 | 1.508(4) |
| N1 | C15 | 1.477(3) | C16 | C22 | 1.346(4) |
| N2 | C14 | 1.134(4) | C18 | C19 | 1.378(4) |
| F2 | C20 | 1.319(3) | C22 | C23 | 1.491(4) |
| C1 | C2 | 1.507(5) | C22 | C29 | 1.500(4) |
| C2 | C3 | 1.383(5) | C23 | C24 | 1.385(4) |
| C2 | C36 | 1.378(5) | C23 | C28 | 1.390(4) |
| C3 | C4 | 1.378(4) | C24 | C25 | 1.377(4) |
| C4 | C5 | 1.369(4) | C25 | C26 | 1.364(5) |
| C5 | C35 | 1.388(4) | C26 | C27 | 1.358(5) |
| C6 | C7 | 1.537(4) | C27 | C28 | 1.390(4) |
| C7 | C8 | 1.539(4) | C29 | C30 | 1.386(4) |
| C7 | C17 | 1.547(4) | C29 | C34 | 1.392(4) |
| C7 | C21 | 1.540(4) | C30 | C31 | 1.386(4) |
| C8 | C9 | 1.509(4) | C31 | C32 | 1.358(5) |
| C9 | C10 | 1.482(4) | C32 | C33 | 1.370(5) |
| C9 | C20 | 1.313(4) | C33 | C34 | 1.389(4) |
| C10 | C11 | 1.396(4) | C35 | C36 | 1.374(5) |
| C10 | C18 | 1.390(4) |  |  |  |

**Supplementary Table 12**. Bond Angles for **47**.

| Atom | Atom | Atom | Angle/˚ | Atom | Atom | Atom | Angle/˚ |
| --- | --- | --- | --- | --- | --- | --- | --- |
| O3 | S1 | O4 | 120.01(15) | C19 | C13 | C12 | 119.8(3) |
| O3 | S1 | N1 | 106.60(13) | C19 | C13 | C14 | 120.2(3) |
| O3 | S1 | C5 | 108.30(14) | N2 | C14 | C13 | 178.0(4) |
| O4 | S1 | N1 | 106.79(14) | N1 | C15 | C16 | 107.7(2) |
| O4 | S1 | C5 | 107.24(14) | C17 | C16 | C15 | 112.1(2) |
| N1 | S1 | C5 | 107.30(13) | C22 | C16 | C15 | 123.0(3) |
| C6 | N1 | S1 | 117.87(18) | C22 | C16 | C17 | 124.8(3) |
| C6 | N1 | C15 | 112.1(2) | C16 | C17 | C7 | 112.3(2) |
| C15 | N1 | S1 | 118.36(18) | C19 | C18 | C10 | 121.4(3) |
| C3 | C2 | C1 | 120.8(4) | C18 | C19 | C13 | 119.7(3) |
| C36 | C2 | C1 | 121.7(4) | F2 | C20 | F1 | 108.8(3) |
| C36 | C2 | C3 | 117.5(3) | C9 | C20 | F1 | 125.1(3) |
| C4 | C3 | C2 | 121.2(3) | C9 | C20 | F2 | 126.1(3) |
| C5 | C4 | C3 | 120.1(3) | C16 | C22 | C23 | 121.8(3) |
| C4 | C5 | S1 | 119.9(2) | C16 | C22 | C29 | 123.3(3) |
| C4 | C5 | C35 | 119.8(3) | C23 | C22 | C29 | 114.9(2) |
| C35 | C5 | S1 | 120.3(2) | C24 | C23 | C22 | 122.5(3) |
| N1 | C6 | C7 | 110.5(2) | C24 | C23 | C28 | 117.8(3) |
| C6 | C7 | C8 | 111.8(2) | C28 | C23 | C22 | 119.7(3) |
| C6 | C7 | C17 | 108.4(2) | C25 | C24 | C23 | 121.2(3) |
| C6 | C7 | C21 | 108.1(2) | C26 | C25 | C24 | 120.3(4) |
| C8 | C7 | C17 | 107.8(2) | C27 | C26 | C25 | 119.7(3) |
| C21 | C7 | C8 | 111.6(2) | C26 | C27 | C28 | 120.8(3) |
| C21 | C7 | C17 | 109.1(2) | C27 | C28 | C23 | 120.1(3) |
| C9 | C8 | C7 | 116.5(2) | C30 | C29 | C22 | 122.0(3) |
| C10 | C9 | C8 | 121.9(2) | C30 | C29 | C34 | 118.1(3) |
| C20 | C9 | C8 | 119.8(3) | C34 | C29 | C22 | 119.9(3) |
| C20 | C9 | C10 | 118.3(3) | C31 | C30 | C29 | 120.9(3) |
| C11 | C10 | C9 | 120.5(3) | C32 | C31 | C30 | 120.2(4) |
| C18 | C10 | C9 | 121.6(3) | C31 | C32 | C33 | 120.3(3) |
| C18 | C10 | C11 | 117.9(3) | C32 | C33 | C34 | 120.3(3) |
| C12 | C11 | C10 | 121.0(3) | C33 | C34 | C29 | 120.2(3) |
| C11 | C12 | C13 | 120.3(3) | C36 | C35 | C5 | 118.9(3) |
| C12 | C13 | C14 | 120.0(3) | C35 | C36 | C2 | 122.3(3) |

**Supplementary Table 13**. Torsion Angles for **47**.

| A | B | C | D | Angle/˚ | A | B | C | D | Angle/˚ |
| --- | --- | --- | --- | --- | --- | --- | --- | --- | --- |
| S1 | N1 | C6 | C7 | -154.20(19) | C11 | C10 | C18 | C19 | -0.6(5) |
| S1 | N1 | C15 | C16 | 155.24(18) | C11 | C12 | C13 | C14 | 179.8(3) |
| S1 | C5 | C35 | C36 | 175.8(3) | C11 | C12 | C13 | C19 | -0.4(5) |
| O3 | S1 | N1 | C6 | 177.8(2) | C12 | C13 | C19 | C18 | -0.3(5) |
| O3 | S1 | N1 | C15 | -42.0(2) | C14 | C13 | C19 | C18 | 179.5(3) |
| O3 | S1 | C5 | C4 | -163.0(2) | C15 | N1 | C6 | C7 | 63.2(3) |
| O3 | S1 | C5 | C35 | 19.5(3) | C15 | C16 | C17 | C7 | -53.8(3) |
| O4 | S1 | N1 | C6 | 48.4(2) | C15 | C16 | C22 | C23 | -179.0(3) |
| O4 | S1 | N1 | C15 | -171.39(19) | C15 | C16 | C22 | C29 | 2.2(4) |
| O4 | S1 | C5 | C4 | -32.2(3) | C16 | C22 | C23 | C24 | 62.5(4) |
| O4 | S1 | C5 | C35 | 150.3(2) | C16 | C22 | C23 | C28 | -119.4(3) |
| N1 | S1 | C5 | C4 | 82.2(3) | C16 | C22 | C29 | C30 | 56.5(4) |
| N1 | S1 | C5 | C35 | -95.2(3) | C16 | C22 | C29 | C34 | -125.2(3) |
| N1 | C6 | C7 | C8 | 63.7(3) | C17 | C7 | C8 | C9 | 158.9(2) |
| N1 | C6 | C7 | C17 | -55.0(3) | C17 | C16 | C22 | C23 | 3.1(4) |
| N1 | C6 | C7 | C21 | -173.1(2) | C17 | C16 | C22 | C29 | -175.6(3) |
| N1 | C15 | C16 | C17 | 57.1(3) | C18 | C10 | C11 | C12 | -0.1(5) |
| N1 | C15 | C16 | C22 | -121.0(3) | C20 | C9 | C10 | C11 | 58.3(4) |
| C1 | C2 | C3 | C4 | 177.1(3) | C20 | C9 | C10 | C18 | -122.9(3) |
| C1 | C2 | C36 | C35 | -176.6(3) | C21 | C7 | C8 | C9 | -81.4(3) |
| C2 | C3 | C4 | C5 | -0.4(5) | C21 | C7 | C17 | C16 | 168.6(2) |
| C3 | C2 | C36 | C35 | 2.0(5) | C22 | C16 | C17 | C7 | 124.2(3) |
| C3 | C4 | C5 | S1 | -175.4(3) | C22 | C23 | C24 | C25 | 179.3(3) |
| C3 | C4 | C5 | C35 | 2.1(5) | C22 | C23 | C28 | C27 | -179.6(3) |
| C4 | C5 | C35 | C36 | -1.7(5) | C22 | C29 | C30 | C31 | 179.9(3) |
| C5 | S1 | N1 | C6 | -66.3(2) | C22 | C29 | C34 | C33 | 179.6(3) |
| C5 | S1 | N1 | C15 | 73.9(2) | C23 | C22 | C29 | C30 | -122.3(3) |
| C5 | C35 | C36 | C2 | -0.4(5) | C23 | C22 | C29 | C34 | 56.0(4) |
| C6 | N1 | C15 | C16 | -62.4(3) | C23 | C24 | C25 | C26 | 0.3(5) |
| C6 | C7 | C8 | C9 | 39.8(3) | C24 | C23 | C28 | C27 | -1.4(4) |
| C6 | C7 | C17 | C16 | 51.1(3) | C24 | C25 | C26 | C27 | -1.5(5) |
| C7 | C8 | C9 | C10 | 66.0(3) | C25 | C26 | C27 | C28 | 1.3(5) |
| C7 | C8 | C9 | C20 | -112.9(3) | C26 | C27 | C28 | C23 | 0.2(5) |
| C8 | C7 | C17 | C16 | -70.1(3) | C28 | C23 | C24 | C25 | 1.2(4) |
| C8 | C9 | C10 | C11 | -120.6(3) | C29 | C22 | C23 | C24 | -118.6(3) |
| C8 | C9 | C10 | C18 | 58.2(4) | C29 | C22 | C23 | C28 | 59.4(3) |
| C8 | C9 | C20 | F1 | -177.8(3) | C29 | C30 | C31 | C32 | -0.4(5) |
| C8 | C9 | C20 | F2 | 0.1(5) | C30 | C29 | C34 | C33 | -2.0(4) |
| C9 | C10 | C11 | C12 | 178.7(3) | C30 | C31 | C32 | C33 | -0.3(5) |
| C9 | C10 | C18 | C19 | -179.4(3) | C31 | C32 | C33 | C34 | -0.1(5) |
| C10 | C9 | C20 | F1 | 3.3(5) | C32 | C33 | C34 | C29 | 1.3(5) |
| C10 | C9 | C20 | F2 | -178.8(3) | C34 | C29 | C30 | C31 | 1.6(5) |
| C10 | C11 | C12 | C13 | 0.6(5) | C36 | C2 | C3 | C4 | -1.6(5) |
| C10 | C18 | C19 | C13 | 0.7(5) |  |  |  |  |  |

**Supplementary Table 14**. Hydrogen Atom Coordinates (Å×10^4^) and Isotropic Displacement Parameters (Å^2^×10^3^) for **47**.

| Atom | *x* | *y* | *z* | U(eq) |
| --- | --- | --- | --- | --- |
| H1 | 10065.77 | 7182.17 | 2790.23 | 155 |
| H2 | 8614.61 | 8047.27 | 2476.14 | 155 |
| H3 | 9057.48 | 6554.88 | 2561.34 | 155 |
| H4 | 6449.34 | 8487.63 | 3597.08 | 75 |
| H32 | 5349.37 | 8511.62 | 5122.27 | 64 |
| H15 | 4772.15 | 6130.2 | 5728.31 | 48 |
| H16 | 3798.26 | 7298.25 | 6319.06 | 48 |
| H9 | 1869.51 | 5793.98 | 7959.23 | 51 |
| H14 | 3301.62 | 5673.53 | 8237.81 | 51 |
| H11 | 2033.92 | 9488.9 | 6689.98 | 61 |
| H12 | 283.14 | 10857.43 | 5894.06 | 65 |
| H6 | 7225.12 | 4706.43 | 7272.47 | 51 |
| H5 | 6871.47 | 4552.15 | 6303.08 | 51 |
| H7 | 3811.44 | 3742.02 | 7303.65 | 51 |
| H8 | 4717.9 | 3859.68 | 6329.14 | 51 |
| H13 | -533.53 | 7646.37 | 7928.51 | 61 |
| H10 | -2306.87 | 9044.4 | 7150.27 | 65 |
| H18 | 1749.05 | 5335.15 | 6440.84 | 80 |
| H17 | 2883.29 | 5407.15 | 5576.8 | 80 |
| H19 | 1832.72 | 6663 | 6037.82 | 80 |
| H24 | 5445.92 | 1345.6 | 7607.88 | 63 |
| H23 | 4149.36 | 139.32 | 8346.32 | 80 |
| H22 | 2948.86 | 542.44 | 9831.76 | 83 |
| H20 | 3123.82 | 2090.22 | 10607.13 | 77 |
| H21 | 4433.1 | 3315.34 | 9888.1 | 61 |
| H29 | 6513.33 | 5046.17 | 8903 | 66 |
| H28 | 8358.01 | 4858.72 | 9655.7 | 82 |
| H25 | 10049.42 | 2899.57 | 9912.68 | 85 |
| H27 | 9922.54 | 1099.21 | 9430.12 | 79 |
| H26 | 8068.95 | 1253.6 | 8690.28 | 64 |
| H31 | 9023.06 | 6226.9 | 5930.29 | 66 |
| H30 | 10103.7 | 6236.49 | 4404.78 | 79 |

**X-Ray Crystallographic Analysis of Complex 76**

**(CCDC number: 2240774)**


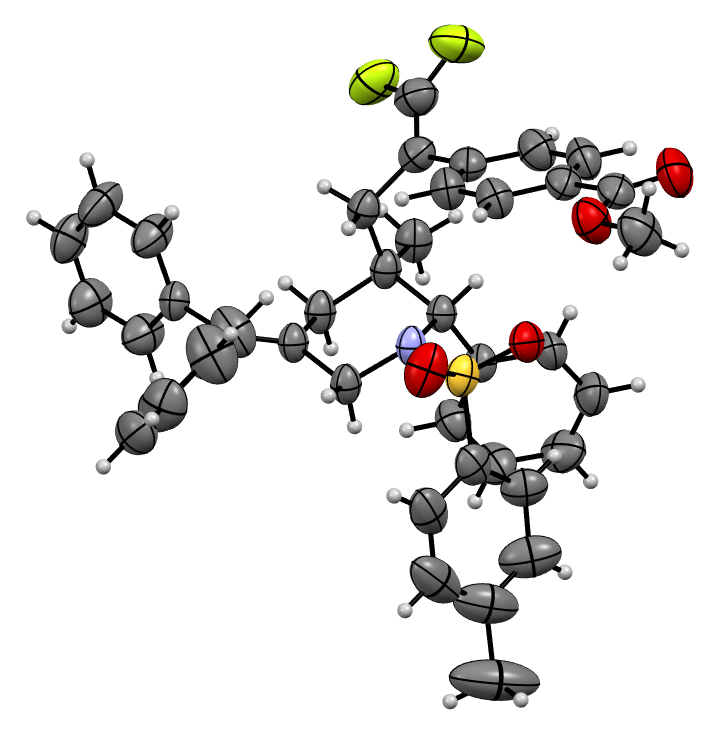


**Supplementary Table 15.** Crystal data and structure refinement for **76**.

| Empirical formula | C_43_H_39_F_2_NO_4_S |
| --- | --- |
| Formula weight | 703.81 |
| Temperature/K | 298.99(10) |
| Crystal system | monoclinic |
| Space group | P21 |
| a/Å | 10.0128(3) |
| b/Å | 16.3243(5) |
| c/Å | 11.4417(2) |
| α/° | 90 |
| β/° | 100.765(2) |
| γ/° | 90 |
| Volume/Å3 | 1837.26(8) |
| Z | 2 |
| ρcalcg/cm3 | 1.272 |
| μ/mm‑1 | 1.224 |
| F(000) | 740.0 |
| Crystal size/mm3 | 0.1 × 0.1 × 0.05 |
| Radiation | Cu Kα (λ = 1.54184) |
| 2Θ range for data collection/° | 7.866 to 151.226 |
| Index ranges | -12 ≤ h ≤ 12, -20 ≤ k ≤ 20, -8 ≤ l ≤ 13 |
| Reflections collected | 28947 |
| Independent reflections | 7271 [Rint = 0.0271, Rsigma = 0.0233] |
| Data/restraints/parameters | 7271/1/464 |
| Goodness-of-fit on F2 | 1.072 |
| Final R indexes [I>=2σ (I)] | R1 = 0.0331, wR2 = 0.0910 |
| Final R indexes [all data] | R1 = 0.0362, wR2 = 0.0937 |
| Largest diff. peak/hole / e Å-3 | 0.12/-0.24 |
| Flack parameter | 0.003(5) |

**Supplementary Table 16**. Fractional Atomic Coordinates (×10^4^) and Equivalent Isotropic Displacement Parameters (Å^2^×10^3^) for **76**. Ueq is defined as 1/3 of the trace of the orthogonalized UIJ tensor.

| Atom | x | y | z | U(eq) |
| --- | --- | --- | --- | --- |
| S26 | 4380.5(6) | 4815.2(4) | 5320.7(5) | 52.83(16) |
| O28 | 4096.1(19) | 4283.8(13) | 4316.6(14) | 61.3(5) |
| O42 | 4485(2) | 5509.9(15) | 863.2(16) | 75.3(6) |
| F32 | -2181(2) | 4743.5(18) | 2195.3(17) | 99.5(6) |
| F33 | -2658.4(17) | 4865.2(15) | 3933.3(19) | 91.1(5) |
| O27 | 4771(2) | 5641.6(14) | 5173(2) | 77.5(6) |
| O44 | 3504(3) | 4425.9(15) | -118.9(17) | 82.1(6) |
| C8 | 356(3) | 6467.0(15) | 8223.3(19) | 48.3(5) |
| N3 | 3000.1(18) | 4847.3(13) | 5907.2(15) | 46.3(4) |
| C1 | 1845(2) | 5499.0(15) | 7388.0(19) | 47.8(5) |
| C20 | 2489(3) | 3348.4(15) | 6176.9(19) | 48.0(5) |
| C38 | 2557(3) | 4965.7(17) | 1473.9(19) | 55.9(6) |
| C7 | 1631(2) | 6251.3(15) | 7789.8(19) | 48.1(5) |
| C41 | 3549(3) | 4929(2) | 655.7(19) | 60.7(7) |
| C34 | -405(3) | 3776.5(18) | 5771(3) | 60.2(6) |
| C29 | 48(3) | 5197.3(17) | 5080(2) | 54.6(5) |
| C35 | 646(3) | 4988.3(16) | 2969(2) | 54.8(6) |
| C14 | 2627(3) | 6931.3(16) | 7820(2) | 51.3(5) |
| C2 | 3183(2) | 5248.2(16) | 7087(2) | 50.3(5) |
| C5 | 614(2) | 4483.1(15) | 5919(2) | 48.3(5) |
| C45 | 5682(2) | 4379.2(19) | 6400(2) | 57.3(6) |
| C6 | 808(2) | 4825.9(18) | 7206.0(19) | 52.3(5) |
| C13 | 146(3) | 6227(2) | 9329(2) | 70.1(8) |
| C4 | 1993(2) | 4175.5(14) | 5643.6(18) | 44.8(5) |
| C31 | -1655(3) | 4871(2) | 3327(3) | 71.5(7) |
| C39 | 2559(3) | 5600.0(18) | 2287(2) | 58.7(6) |
| C30 | -374(3) | 4998.8(16) | 3769(2) | 57.0(6) |
| C37 | 1614(3) | 4348.6(19) | 1428(2) | 66.8(7) |
| C46 | 5853(3) | 3538(2) | 6470(3) | 66.7(7) |
| C21 | 2568(3) | 2709.5(17) | 5388(2) | 57.3(6) |
| C50 | 6502(3) | 4881(3) | 7207(3) | 77.8(9) |
| C12 | -1016(4) | 6465(3) | 9739(3) | 81.4(10) |
| C11 | -1978(3) | 6933(2) | 9055(3) | 75.1(8) |
| C17 | 4497(4) | 8208(2) | 7861(3) | 84.1(9) |
| C23 | 3502(4) | 1802(2) | 6967(3) | 76.8(8) |
| C19 | 3259(3) | 7288(2) | 8887(2) | 65.4(7) |
| C36 | 674(3) | 4352.6(19) | 2167(2) | 66.1(7) |
| C24 | 3411(4) | 2420(2) | 7762(3) | 72.8(8) |
| C40 | 1604(3) | 5606.0(18) | 3018(2) | 60.2(6) |
| C15 | 2962(4) | 7243(2) | 6791(3) | 79.4(9) |
| C10 | -1800(4) | 7167(2) | 7956(3) | 84.5(10) |
| C9 | -633(3) | 6944(2) | 7552(3) | 75.6(9) |
| C18 | 4187(4) | 7921(2) | 8905(3) | 81.0(9) |
| C25 | 2921(3) | 3188.8(18) | 7383(2) | 60.9(6) |
| C22 | 3066(3) | 1947.6(19) | 5770(3) | 69.6(7) |
| C49 | 7476(3) | 4540(4) | 8071(3) | 100.1(17) |
| C48 | 7665(4) | 3705(4) | 8157(3) | 101.3(16) |
| C43 | 5499(4) | 5512(3) | 117(3) | 85.9(10) |
| C16 | 3880(5) | 7876(3) | 6823(4) | 99.0(13) |
| C47 | 6852(4) | 3215(3) | 7343(3) | 94.1(12) |
| C51 | 8790(5) | 3338(5) | 9090(4) | 166(3) |

**Supplementary Table 17**. Anisotropic Displacement Parameters (Å2×10^3^) for **76**. The Anisotropic displacement factor exponent takes the form: -2π^2^[h^2^a*^2^U^11^+2hka*b*U^12^+…].

| Atom | U11 | U22 | U33 | U23 | U13 | U12 |
| --- | --- | --- | --- | --- | --- | --- |
| S26 | 53.5(3) | 55.4(3) | 57.3(3) | 0.7(3) | 30.4(2) | -1.1(3) |
| O28 | 61.9(10) | 78.9(13) | 48.6(8) | -3.9(8) | 24.3(7) | 8.0(9) |
| O42 | 78.6(14) | 90.7(17) | 61.8(10) | -11.7(10) | 26.9(9) | -8.9(12) |
| F32 | 69.7(11) | 126.3(18) | 91.2(11) | 0.2(12) | -13.7(9) | -5.8(13) |
| F33 | 50.9(9) | 99.2(14) | 125.0(14) | 2.8(13) | 21.2(8) | 2.4(11) |
| O27 | 82.9(14) | 60.2(12) | 103.9(14) | 11.0(11) | 55.0(12) | -8.5(10) |
| O44 | 110.2(18) | 82.9(15) | 57.9(10) | -14.6(10) | 28.0(11) | -0.3(13) |
| C8 | 54.5(13) | 44.0(12) | 50.7(11) | -4.5(9) | 20.6(9) | 0.8(9) |
| N3 | 47.6(9) | 45.7(10) | 51.6(9) | -5.6(9) | 24.5(7) | -3.2(9) |
| C1 | 50.9(12) | 48.3(13) | 49.2(11) | -5.8(9) | 21.9(9) | -1.3(10) |
| C20 | 51.7(13) | 44.4(13) | 51.0(11) | -4.5(9) | 17.3(9) | -3.1(9) |
| C38 | 66.0(14) | 57.6(16) | 43.9(10) | 2.5(10) | 9.2(9) | 7.6(12) |
| C7 | 52.5(12) | 48.4(13) | 47.0(10) | -3.2(9) | 18.8(9) | 0.3(10) |
| C41 | 72.8(16) | 65.5(18) | 43.1(11) | 4.8(11) | 9.3(10) | 10.5(15) |
| C34 | 57.4(15) | 55.4(16) | 72.9(15) | -8.1(12) | 25.4(12) | -11.0(12) |
| C29 | 50.8(13) | 52.0(14) | 64.4(13) | -5.2(11) | 19.5(10) | 2.7(11) |
| C35 | 57.7(13) | 55.4(16) | 50.1(11) | 2.3(10) | 7.0(9) | 4.8(11) |
| C14 | 53.2(13) | 44.5(13) | 59.4(12) | -3.2(10) | 18.6(10) | 1.8(10) |
| C2 | 49.5(13) | 49.4(13) | 56.9(12) | -10.2(10) | 22.5(10) | -4.6(10) |
| C5 | 46.6(11) | 47.5(12) | 55.7(11) | -6.0(10) | 22.6(9) | -2.3(10) |
| C45 | 46.8(12) | 74.4(18) | 57.2(12) | -6.8(12) | 26.5(10) | -7.8(12) |
| C6 | 54.6(12) | 50.9(12) | 59.2(11) | -7.3(11) | 31.0(9) | -4.9(12) |
| C13 | 66.8(17) | 91(2) | 58.1(14) | 16.5(14) | 26.5(13) | 11.9(16) |
| C4 | 47.4(11) | 45.0(12) | 45.7(10) | -4.0(9) | 18.0(8) | 0.2(9) |
| C31 | 57.7(14) | 74.1(19) | 80.0(16) | 2.6(16) | 5.7(12) | 2.9(16) |
| C39 | 69.3(16) | 54.3(15) | 54.3(12) | -1.4(11) | 16.1(11) | -6.3(13) |
| C30 | 51.9(13) | 53.4(16) | 65.2(13) | 1.9(11) | 9.8(10) | 4.3(11) |
| C37 | 88(2) | 59.4(17) | 52.8(12) | -10.7(12) | 12.9(12) | -2.1(15) |
| C46 | 49.9(14) | 76(2) | 74.3(16) | 12.5(14) | 11.9(12) | -7.6(13) |
| C21 | 61.6(14) | 53.2(15) | 56.3(12) | -12.6(10) | 9.2(10) | 1.3(12) |
| C50 | 50.6(13) | 108(3) | 81.0(17) | -32.7(18) | 27.6(12) | -15.9(17) |
| C12 | 82(2) | 106(3) | 66.4(16) | 10.3(17) | 41.3(15) | 7.2(19) |
| C11 | 72.4(19) | 74(2) | 91(2) | 1.5(16) | 46.3(16) | 10.6(16) |
| C17 | 79(2) | 64(2) | 113(3) | -6.6(18) | 28.2(19) | -21.2(17) |
| C23 | 83(2) | 52.5(17) | 91(2) | 7.9(15) | 5.6(16) | 1.3(15) |
| C19 | 70.3(17) | 65.2(18) | 61.7(14) | -9.4(12) | 14.4(12) | -7.8(14) |
| C36 | 74.6(18) | 58.9(17) | 64.3(14) | -6.7(12) | 12.2(12) | -9.3(14) |
| C24 | 94(2) | 58.7(18) | 63.9(15) | 11.4(13) | 10.6(15) | -0.9(16) |
| C40 | 68.8(16) | 56.5(15) | 57.9(13) | -8.1(11) | 18.1(11) | -1.4(13) |
| C15 | 104(3) | 74(2) | 64.4(16) | -2.2(14) | 27.0(16) | -30.3(19) |
| C10 | 78(2) | 91(3) | 93(2) | 23.4(18) | 37.0(17) | 36.8(19) |
| C9 | 81(2) | 89(2) | 65.4(15) | 22.8(15) | 34.8(14) | 29.7(18) |
| C18 | 77(2) | 67(2) | 94(2) | -17.7(17) | 3.7(16) | -10.9(16) |
| C25 | 78.4(18) | 56.4(16) | 50.1(12) | -1.8(11) | 17.3(11) | -4.0(13) |
| C22 | 77.1(19) | 49.2(15) | 81.6(17) | -15.0(13) | 12.4(14) | 4.9(14) |
| C49 | 56.6(18) | 183(6) | 65.1(17) | -30(2) | 23.8(14) | -29(2) |
| C48 | 54.6(18) | 179(5) | 70.7(19) | 36(2) | 12.2(14) | -24(2) |
| C43 | 84(2) | 116(3) | 65.2(16) | -7.1(18) | 31.2(16) | -8(2) |
| C16 | 129(3) | 85(3) | 92(2) | 3(2) | 44(2) | -42(2) |
| C47 | 59.4(19) | 112(3) | 110(3) | 47(2) | 14.7(17) | -10.0(19) |
| C51 | 79(3) | 301(10) | 110(3) | 97(5) | -9(2) | -35(4) |

**Supplementary Table 18**. Bond Lengths for **76**.

| Atom | Atom | Length/Å | Atom | Atom | Length/Å |
| --- | --- | --- | --- | --- | --- |
| S26 | O28 | 1.4253(19) | C35 | C36 | 1.389(4) |
| S26 | O27 | 1.423(2) | C35 | C40 | 1.386(4) |
| S26 | N3 | 1.6462(17) | C14 | C19 | 1.394(4) |
| S26 | C45 | 1.769(3) | C14 | C15 | 1.380(4) |
| O42 | C41 | 1.323(4) | C5 | C6 | 1.553(3) |
| O42 | C43 | 1.444(4) | C5 | C4 | 1.556(3) |
| F32 | C31 | 1.320(3) | C45 | C46 | 1.384(4) |
| F33 | C31 | 1.323(3) | C45 | C50 | 1.384(4) |
| O44 | C41 | 1.203(3) | C13 | C12 | 1.389(4) |
| C8 | C7 | 1.496(3) | C31 | C30 | 1.305(4) |
| C8 | C13 | 1.377(3) | C39 | C40 | 1.383(4) |
| C8 | C9 | 1.376(4) | C37 | C36 | 1.377(4) |
| N3 | C2 | 1.480(3) | C46 | C47 | 1.379(5) |
| N3 | C4 | 1.482(3) | C21 | C22 | 1.380(4) |
| C1 | C7 | 1.342(3) | C50 | C49 | 1.370(6) |
| C1 | C2 | 1.501(3) | C12 | C11 | 1.358(5) |
| C1 | C6 | 1.499(3) | C11 | C10 | 1.358(4) |
| C20 | C4 | 1.526(3) | C17 | C18 | 1.372(5) |
| C20 | C21 | 1.391(3) | C17 | C16 | 1.345(5) |
| C20 | C25 | 1.392(3) | C23 | C24 | 1.372(5) |
| C38 | C41 | 1.487(4) | C23 | C22 | 1.378(5) |
| C38 | C39 | 1.392(4) | C19 | C18 | 1.387(5) |
| C38 | C37 | 1.375(4) | C24 | C25 | 1.387(4) |
| C7 | C14 | 1.488(4) | C15 | C16 | 1.380(5) |
| C34 | C5 | 1.528(3) | C10 | C9 | 1.383(4) |
| C29 | C5 | 1.549(4) | C49 | C48 | 1.379(7) |
| C29 | C30 | 1.515(4) | C48 | C47 | 1.373(6) |
| C35 | C30 | 1.494(4) | C48 | C51 | 1.522(6) |

**Supplementary Table 19**. Bond Angles for **76**.

| Atom | Atom | Atom | Angle/˚ | Atom | Atom | Atom | Angle/˚ |
| --- | --- | --- | --- | --- | --- | --- | --- |
| O28 | S26 | N3 | 106.94(10) | C6 | C5 | C4 | 110.45(19) |
| O28 | S26 | C45 | 109.15(12) | C46 | C45 | S26 | 120.5(2) |
| O27 | S26 | O28 | 119.96(13) | C50 | C45 | S26 | 119.7(3) |
| O27 | S26 | N3 | 106.74(12) | C50 | C45 | C46 | 119.8(3) |
| O27 | S26 | C45 | 106.35(15) | C1 | C6 | C5 | 110.91(17) |
| N3 | S26 | C45 | 107.04(10) | C8 | C13 | C12 | 120.8(3) |
| C41 | O42 | C43 | 116.7(2) | N3 | C4 | C20 | 114.53(19) |
| C13 | C8 | C7 | 121.6(2) | N3 | C4 | C5 | 108.40(18) |
| C9 | C8 | C7 | 121.2(2) | C20 | C4 | C5 | 116.06(18) |
| C9 | C8 | C13 | 117.2(2) | F32 | C31 | F33 | 108.1(2) |
| C2 | N3 | S26 | 114.56(14) | C30 | C31 | F32 | 126.0(3) |
| C2 | N3 | C4 | 118.44(16) | C30 | C31 | F33 | 125.8(3) |
| C4 | N3 | S26 | 118.98(14) | C40 | C39 | C38 | 119.7(3) |
| C7 | C1 | C2 | 122.3(2) | C35 | C30 | C29 | 120.9(2) |
| C7 | C1 | C6 | 124.6(2) | C31 | C30 | C29 | 119.4(3) |
| C6 | C1 | C2 | 113.1(2) | C31 | C30 | C35 | 119.7(2) |
| C21 | C20 | C4 | 117.2(2) | C38 | C37 | C36 | 121.1(2) |
| C21 | C20 | C25 | 117.3(2) | C47 | C46 | C45 | 119.1(3) |
| C25 | C20 | C4 | 125.4(2) | C22 | C21 | C20 | 122.1(2) |
| C39 | C38 | C41 | 122.2(3) | C49 | C50 | C45 | 119.7(4) |
| C37 | C38 | C41 | 118.8(2) | C11 | C12 | C13 | 120.7(3) |
| C37 | C38 | C39 | 119.0(2) | C10 | C11 | C12 | 119.4(3) |
| C1 | C7 | C8 | 122.0(2) | C16 | C17 | C18 | 119.4(3) |
| C1 | C7 | C14 | 122.8(2) | C24 | C23 | C22 | 119.0(3) |
| C14 | C7 | C8 | 115.2(2) | C18 | C19 | C14 | 121.2(3) |
| O42 | C41 | C38 | 113.1(2) | C37 | C36 | C35 | 120.6(3) |
| O44 | C41 | O42 | 123.3(3) | C23 | C24 | C25 | 121.4(3) |
| O44 | C41 | C38 | 123.6(3) | C39 | C40 | C35 | 121.4(2) |
| C30 | C29 | C5 | 117.1(2) | C16 | C15 | C14 | 121.4(3) |
| C36 | C35 | C30 | 120.9(2) | C11 | C10 | C9 | 120.1(3) |
| C40 | C35 | C30 | 120.9(2) | C8 | C9 | C10 | 121.8(3) |
| C40 | C35 | C36 | 118.1(2) | C17 | C18 | C19 | 120.1(3) |
| C19 | C14 | C7 | 121.7(2) | C24 | C25 | C20 | 120.4(3) |
| C15 | C14 | C7 | 121.6(2) | C23 | C22 | C21 | 119.9(3) |
| C15 | C14 | C19 | 116.7(3) | C50 | C49 | C48 | 121.5(4) |
| N3 | C2 | C1 | 111.49(19) | C49 | C48 | C51 | 120.8(5) |
| C34 | C5 | C29 | 109.9(2) | C47 | C48 | C49 | 118.1(4) |
| C34 | C5 | C6 | 109.87(19) | C47 | C48 | C51 | 121.0(6) |
| C34 | C5 | C4 | 109.5(2) | C17 | C16 | C15 | 121.2(3) |
| C29 | C5 | C6 | 106.5(2) | C48 | C47 | C46 | 121.8(4) |
| C29 | C5 | C4 | 110.66(18) |  |  |  |  |

**Supplementary Table 20**. Hydrogen Atom Coordinates (Å×10^4^) and Isotropic Displacement Parameters (Å^2^×10^3^) for **76**.

| Atom | x | y | z | U(eq) |
| --- | --- | --- | --- | --- |
| H34A | -1275.21 | 3975.75 | 5880.24 | 90 |
| H34B | -94.4 | 3361.28 | 6352.84 | 90 |
| H34C | -484.51 | 3548.64 | 4987.79 | 90 |
| H29A | -734.05 | 5423.76 | 5354.45 | 66 |
| H29B | 734.83 | 5623.21 | 5160.51 | 66 |
| H2A | 3754.37 | 5728.08 | 7088.32 | 60 |
| H2B | 3639.23 | 4873.48 | 7689.59 | 60 |
| H6A | -52.1 | 5034.31 | 7353.48 | 63 |
| H6B | 1100.06 | 4388.12 | 7769.12 | 63 |
| H13 | 789.47 | 5901.05 | 9805.56 | 84 |
| H4 | 1842.17 | 4099.91 | 4779.2 | 54 |
| H39 | 3198.27 | 6017.85 | 2337.79 | 70 |
| H37 | 1610.55 | 3921.53 | 889.52 | 80 |
| H46 | 5302.17 | 3195.59 | 5935.9 | 80 |
| H21 | 2276.72 | 2798.6 | 4577.72 | 69 |
| H50 | 6391.67 | 5446.38 | 7163.92 | 93 |
| H12 | -1135.64 | 6301.66 | 10491.73 | 98 |
| H11 | -2751.6 | 7092.56 | 9336.84 | 90 |
| H17 | 5128.23 | 8626.6 | 7869.88 | 101 |
| H23 | 3851.58 | 1293.55 | 7232.58 | 92 |
| H19 | 3055.01 | 7098.72 | 9599.84 | 79 |
| H36 | 52.08 | 3925.73 | 2128.06 | 79 |
| H24 | 3684.36 | 2321.37 | 8571.94 | 87 |
| H40 | 1605.56 | 6033.78 | 3555.38 | 72 |
| H15 | 2560.74 | 7020.95 | 6061.11 | 95 |
| H10 | -2463.22 | 7477.14 | 7473.78 | 101 |
| H9 | -513.31 | 7122.16 | 6806.04 | 91 |
| H18 | 4598.51 | 8151.66 | 9625.75 | 97 |
| H25 | 2881.3 | 3599.86 | 7938.1 | 73 |
| H22 | 3107.33 | 1532.87 | 5220.77 | 84 |
| H49 | 8023.03 | 4881.12 | 8611.39 | 120 |
| H43A | 5104.97 | 5717.78 | -657.05 | 129 |
| H43B | 5821.61 | 4964.3 | 44.94 | 129 |
| H43C | 6244.4 | 5856.67 | 466.45 | 129 |
| H16 | 4076.15 | 8077.93 | 6113.93 | 119 |
| H47 | 6978.03 | 2650.95 | 7381.44 | 113 |
| H51A | 9631.53 | 3352.26 | 8803.08 | 250 |
| H51B | 8567.12 | 2781.34 | 9242.61 | 250 |
| H51C | 8880.24 | 3650.27 | 9811.35 | 250 |
